# Polyhalonitrobutadienes as Versatile Building Blocks for the Biotargeted Synthesis of Substituted N-Heterocyclic Compounds †

**DOI:** 10.3390/molecules25122863

**Published:** 2020-06-21

**Authors:** Viktor A. Zapol’skii, Ursula Bilitewski, Sören R. Kupiec, Isabell Ramming, Dieter E. Kaufmann

**Affiliations:** 1 Institute of Organic Chemistry, Clausthal University of Technology, Leibnizstraße 6, 38678 Clausthal-Zellerfeld, Germany; viktor.zapolskii@tu-clausthal.de (V.A.Z.); SOEREN104@gmail.com (S.R.K.); 2 Helmholtz Centre for Infection Research (HZI), Inhoffenstr. 7, 38124 Braunschweig, Germany; ursula.bilitewski@helmholtz-hzi.de (U.B.); Isabell.Ramming@helmholtz-hzi.de (I.R.)

**Keywords:** polyhalonitrobutadienes, nucleophilic substitution, heterocyclization, nitrogen heterocycles, medicinal chemistry

## Abstract

Substituted nitrogen heterocycles are structural key units in many important pharmaceuticals. A new synthetic approach towards heterocyclic compounds displaying antibacterial activity against *Staphylococcus aureus* or cytotoxic activity has been developed. The selective synthesis of a series of 64 new N-heterocycles from the three nitrobutadienes 2-nitroperchloro-1,3-butadiene, 4-bromotetrachloro-2-nitro-1,3-butadiene and (*Z*)-1,1,4-trichloro-2,4-dinitrobuta-1,3-diene proved feasible. Their reactions with N-, O- and S-nucleophiles provide rapid access to push-pull substituted benzoxazolines, benzimidazolines, imidazolidines, thiazolidinones, pyrazoles, pyrimidines, pyridopyrimidines, benzoquinolines, isothiazoles, dihydroisoxazoles, and thiophenes with unique substitution patterns. Antibacterial activities of 64 synthesized compounds were examined. Additionally, seven compounds (thiazolidinone, nitropyrimidine, indole, pyridopyrimidine, and thiophene derivatives) exhibited a significant cytotoxicity with IC_50_-values from 1.05 to 20.1 µM. In conclusion, it was demonstrated that polyhalonitrobutadienes have an interesting potential as structural backbones for a variety of highly functionalized, pharmaceutically active heterocycles.

## 1. Introduction

**Scheme 1:**
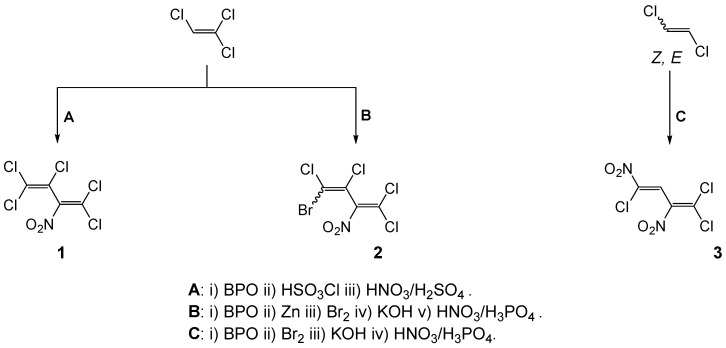
Synthesis of the polyhalonitrobutadienes **1**–**3**.

Halogenated nitrobutadienes are part of a relatively small group of selectively reactive aliphatic nitro compounds [1]. Representatives with one or two nitro and three to five halogen groups are easily accessible by introduction of an activating and directing nitro group into polyhalo-1,3-butadienes [2]. These can be easily obtained in high purity and multigram scale by radical dimerization of industrial solvents such as trichloroethene and 1,2-dichloroethene with subsequent dehydrohalogenation-halogenation, followed by nitration reactions. 2-Nitroperchlorobutadiene (**1**) has been synthesized in three steps from trichloroethene [3,4] (Scheme 1). 4-Bromotetrachloro-2-nitrobutadiene (**2**) could be obtained from trichloroethene in five steps [5]. (*Z*)-1,1,4-Trichloro-2,4-dinitrobutadiene (**3**) was made accessible in four steps from a *Z*,*E*-mixture of 1,2-dichloroethene [6].

Due to their graded reactivity in S_N_ reactions, nitro-substituted polyhalo-1,3-butadienes have proven to be valuable synthetic precursors for a variety of four- to six-membered, often pharmaceutically active heterocycles. They bear a unique substitution pattern that cannot be accessed easily on an alternative pathway. This structural overview for medicinal chemists demonstrates the broad synthetic potential of the nitrodienes **1**, **2,** and **3** as a backbone for specifically substituted heterocycles, owing to the additional potential for predictable successive molecular fine tuning.

## 2. Results and Discussion

### 2.1. Chemistry

#### 2.1.1. Benzoxazolines and Benzimidazolines

**Scheme 2:**
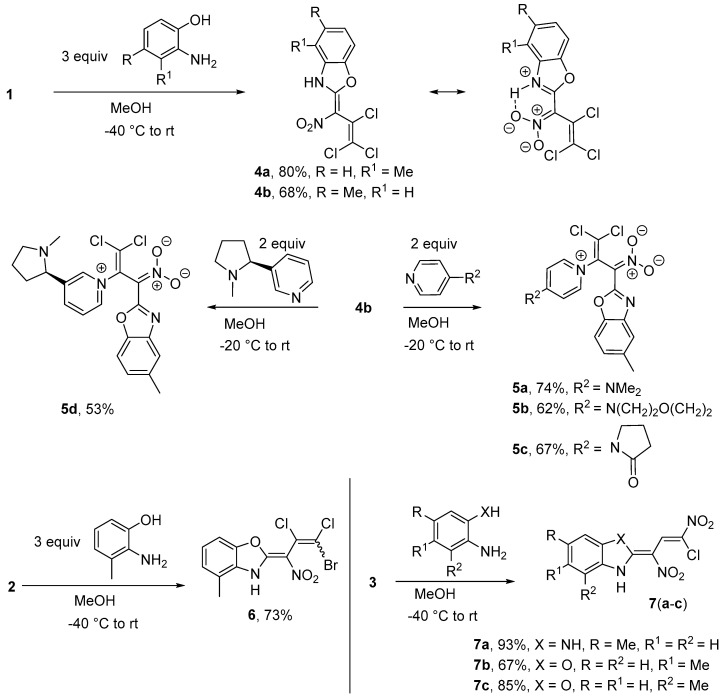
Synthesis of benzoxazolines **4**, **5**, **6**, **7 b**,**c** and benzimidazoline **7a**.

Due to the high electrophilicity of its nitrodichlorovinyl group, **1** reacts readily with various amines which, according to Pearson’s scale, are hard nucleophiles [7]. Thus, reactions of 2-aminophenol derivatives with **1** occur under mild conditions and lead to substitution of both Cl groups of the nitrodichlorovinyl unit with formation of the corresponding (*E*)-2,3-di-hydrobenzoxazoles **4a**,**b** in reasonable yields [8]. The substructure of a ß-nitro-substituted enamine within compounds **4a,b** should enable a stabilization caused by a strong hydrogen bond between an oxygen atom of the nitro group and the proton at the oxazoline nitrogen atom. The downfield-shifted ^1^H nmr signal for the NH-group between 11.8 ppm in CDCl_3_ and 13.6 ppm in DMSO-*d_6_* points out that H-bonding (Scheme 2) must be important and azoles **4a**,**b** are obtained hereby exclusively as *E*-isomers. Under mild reaction conditions, benzoxazoline **4b** reacts with activated pyridine derivatives, forming betaines **5a**–**c** in 62–74% yield. Treatment of **4b** with nicotine under the same reaction conditions furnishes azinate **5d** in 53% yield. Synthesis of the novel and structurally interesting cross-conjugated inner salts **5a**–**d** demonstrates the broad synthetic applicability of 2-nitroperchlorobutadiene [9]. Benzoxazoline **6** was obtained in 73% yield as a 1:1 mixture of two isomers by the action of 2-amino-3-methylphenol on the bromonitrodiene **2** at −40 °C in methanol. The reaction of dinitrodiene **3** with 4-methylbenzene-1,2-diamine at −40 °C results almost quantitatively in the formation of benzimidazoline **7a**. By treatment of **3** with 2-aminophenol derivatives, the corresponding benzoxazolines **7b**,**c** were obtained in acceptable yields (Scheme 2). Similar benzoxazolines and benzimidazolines exhibit herbicidal activity and act as a model compound when exploring caseinolytic protease as target for herbicides or growth regulators [10].

#### 2.1.2. Imidazolidines

**Scheme 3:**
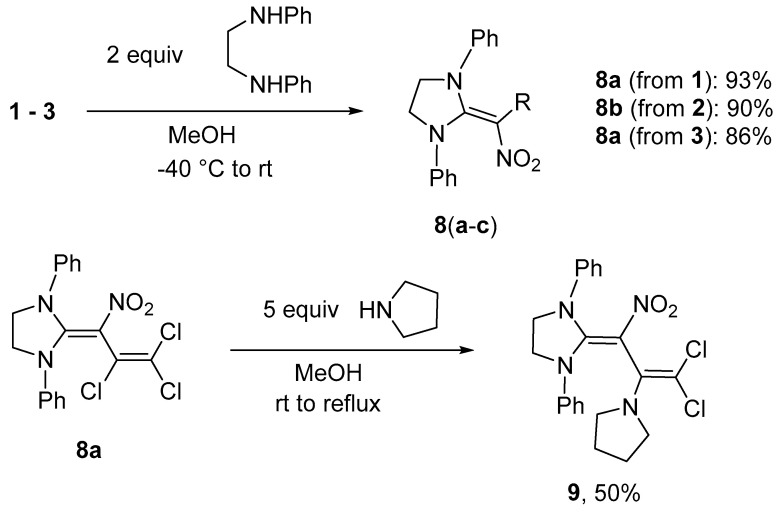
Synthesis of imidazolidines **8**, **9**.

Imidazolidines **8** have been prepared by reaction of nitrodienes **1**–**3** with *N^1^, N^2^*-diphenylethane-1,2-diamine in methanol. Under optimized conditions, yields of the products **8** reached 86–93% (Scheme 3). Only compound **8a** was previously prepared [11] and isolated in 25% yield by reaction of (1,3,4,4-tetrachloro-2-nitrobuta-1,3-dien-1-yl)(*p*-tolyl)sulfane with *N*^1^,*N*^2^-diphenylethane-1,2-di-amine. Treatment of imidazolidine **8a** with a fivefold excess of pyrrolidine in refluxing methanol led to the formation of a S_N_Vin product, the 2-(3,3-dichloro-1-nitro-2-(pyrrolidin-1-yl)allylidene)-1,3-diphenylimidazolidine (**9**) in 50% yield.

#### 2.1.3. Imidacloprid Analogues

**Scheme 4:**
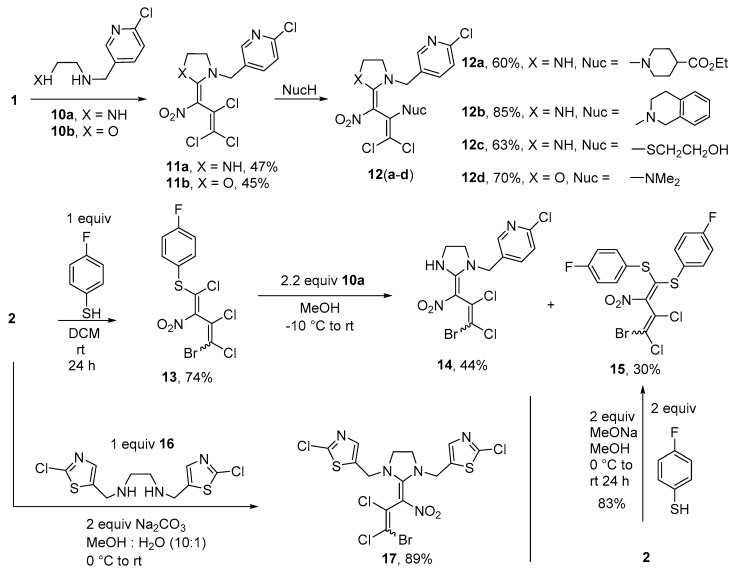
Synthesis of Imidacloprid analogues **11**, **12**, **14**, **17**.

The imidazolidine Imidacloprid (*N*-{1-[(6-chloro-3-pyridyl)methyl]-4,5-dihydroimidazol-2-yl}-nitramide) has been the most widely used systemic insecticide worldwide. A first synthesis of ana-logues from nitropolychloroalkenes has been reported [12,13], new types of derivatives are presented here. For instance, **11a** and **11b** were obtained from nitrodiene **1** and chloropyridines **10a** and **10b**, respectively [13]. Reaction of imidazolidine **11a** with a 2.5-fold excess of N-nucleophiles such as ethyl piperidine-4-carboxylate and 1,2,3,4-tetrahydroisoquinoline in methanol at 35–50 °C leads to compounds **12a** and **12b** in 60–85% yield, respectively. By using 2-mercaptoethan-1-ol as S-nucleophile for the reaction with **11a** in the presence of sodium ethanolate, the corresponding sulfane **12c** was obtained in 63% yield. Treatment of **11b** with a fivefold excess of dimethylamine at rt led to the formation of oxazolidine **12d** (70%). By the reaction of bromonitrodiene **2** with an equimolar amount of 4-fluorobenzenethiol in DCM at rt, sulfane **13** was obtained as mixture of two isomers in a total yield of 74%. The subsequent vinylic substitution of the monothio compound **13** by means of **10a** gave imidazolidine **14** (44%) as well as ketene dithioacetal **15** (30% yield). Arylthiols are known to be both, good nucleophiles as well as good leaving groups. Compound **14** was previously syn-thesized in 40% yield directly from nitrodiene **2** and diamine **10a [12]**. 1,1-Dithio compound **15** could be obtained in 83% yield from diene **2** and two equivalents of 4-fluorobenzenethiol using sodium methanolate as a base. The reaction of diene **2** with diamine **16** [13] at optimized conditions furnished the imidazolidine **17** as a mixture of two isomers in a total yield of 89% (Scheme 4).

#### 2.1.4. Thiazolidinones

Thiazolidin-4-ones represent a class of compounds that has proven to exhibit distinctive bio-activity, e.g. antifungal, antibacterial, antitubercular, and anticonvulsant properties [14,15,16,17]. Our research in this area is presented through an efficient synthesis of functionalized (*Z*)-2-allylidene-thiazolidin-4-ones [18]. Nitrodiene **1** reacts with ethyl 2-mercaptoacetate to give the sulfane **18** as single *E*-isomer [19]. For the subsequent reactions of sulfane **18,** we used two aniline derivatives, an activated (ERG) and an desactivated (EWG) one. In both cases, the expected thiazolidinones **19a**,**b** were obtained in good yields (73–76%). Treatment of **19a**,**b** with a 5– to 8–fold excess of hydrazine led to pyrazoles **20a**,**b**. The assumed mechanism for this ring-opening and subsequent ring-closure transformation forming **20a**,**b** has been presented [18]. Heating of thiazolidinones **19a**,**b** with five membered 2-formyl heterocycles in acetic acid in the presence of trimethylamine furnished hetarylmethylidenethiazolidinones **21a**,**f** in good to excellent yields, as single diastereomers. The *Z*-configuration was assigned according to literature data. The presence of only one signal for the methylidene proton at 7.72–8.15 ppm in the ^1^H nmr spectra of compounds **21a**,**f** suggested the formation of a single isomer, which was assigned to the *Z*-configuration according to the literature for similar compounds [20,21]. Interestingly, close analogues of these structures, i.e., the 5-arylmethylidene rhodanines, possess photosynthesis-inhibiting and antialgae properties [22], show anticancer activity [23,24], and are inhibitors of bacterial enzyme synthetase MurD with *E. coli* [25].

**Scheme 5:**
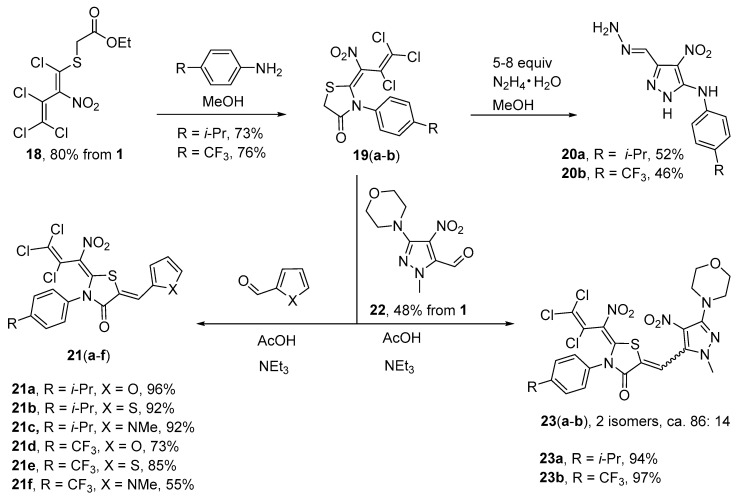
Synthesis of thiazolidinones **19**, **21**, **23** and pyrazoles **20** and **23**.

Other thiazolidinone derivatives can be obtained by treatment of **19a** and **19b** with 5-formyl-pyrazole **22**, previously obtained from **1** in 48% yield, as already reported [26]. Under standard reaction conditions, the corresponding pyrazolyl-thiazolidinones **23a**,**b** were obtained as a mixture of two isomers in relation of 86:14. Formation of a mixture of isomers can be explained in this case by the bulky structure of pyrazole **22** compared with 2-formyl derivatives of furan, thiophene or 1-methylpyrrole (Scheme 5).

#### 2.1.5. Benzazetines

**Scheme 6:**
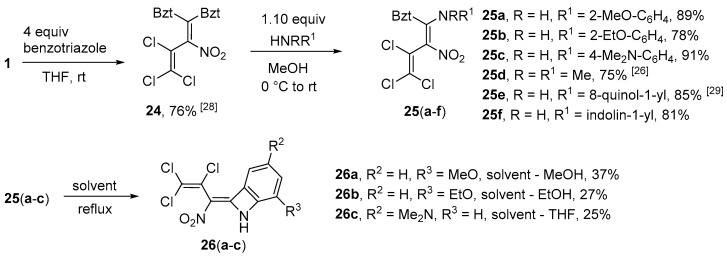
Synthesis of benzazetines **26** and benzotriazoles **25**.

Functionally substituted benzazetines have also been made accessible starting from **1** [27]. The derived bis(benzotriazole) **24** was obtained in 76% yield according to the literature [28]. Single amine exchange of azole **24** is feasible with aromatic, aliphatic, and heteroaromatic amines in methanol at mild reactions conditions and led to 1-benzotriazolyl-1-(organylamino)-2-nitro-3,4,4-trichloro-1,3-butadienes **25a**–**f** in good yields (75–91%). Dienes **25d** and **25e** have been described, already [26,29]. Heating of nitrodienes **25a**–**c** in different solvents (MeOH for **26a**, EtOH for **26b,** and THF for **26c**) gave the corresponding benzazetines **26a**–**c** in 25-37% yield (Scheme 6). No reaction occurred in diethyl ether, while in benzene, strong tarring was observed [30]. Some details on the synthesis and chemical transformations of benzazetines have been published, already [30]. N-Unsubstituted derivatives are unstable. Stable substituted representatives have become accessible for the first time in recent years. The substructure of a ß-nitro-substituted enamine within compounds **26a**–**c** should enable a stabilization caused by a strong hydrogen bond between an oxygen atom of the nitro group and the single proton at the azetine nitrogen atom. The wide-ranging synthetic possibilities of benzazetines and benzazetes have also been discussed.

4-Ethoxy-2-(2,3,3-trichloro-1-nitro-2-propenylidene)-benzazetine (a regioisomer of compound **26b**) can modulate RNA binding proteins [31]. A new prenylated indole alkaloid taichunamide A contains a benzazetidine unit, too [32]. It was isolated from the fungus *Aspergillus taichungensis*.

#### 2.1.6. Pyrimidines

**Scheme 7:**
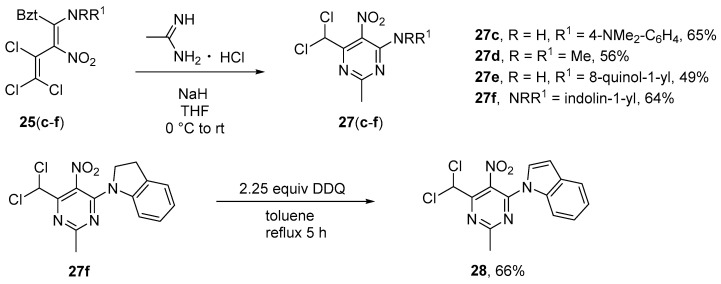
Synthesis of pyrimidines **27**, **28**.

In the course of the studies concerning polyhalogenated nitrobutadienes, a new ring closure approach to perfunctionalized 5-nitropyrimidines was also developed [33]. Using this protocol starting from **25c**–**f**, four new nitropyrimidines **27c**–**f** were obtained. Even under optimum conditions, yields of the products **27c**–**f** remained moderate, reaching 49–65%. The assumed mechanism for the formation of pyrimidines **27** has been presented in the literature [33]. 5-Nitro-substituted pyrimidines are interesting precursors for the synthesis of a wide range of poly-substituted pyrimidines and other heterocyclic systems with potential biological activity [34]. Among numerous applications, some examples are noteworthy: cytotoxic activity is documented [35,36] as well as the potential to inactivate the human DNA repair process [37]. The broad variety of medicinal applications is further illustrated, e.g. with the activity against chronic obstructive pulmonary disease [38], applicability against herpes simplex [39], and other viral diseases [40]. Furthermore, one field of application of 5-nitropyrimidines uses their positive modulating effect of the GABAB receptor [41,42]. Pyrimidin-4-yl-1*H*-indoles are a very rare class of organic compounds; to the best of our knowledge, only 4 representatives are known till today [43,44,45]. With the aim to synthesize a new pyrimidin-4-yl-1*H*-indole with potent biological activity, we made an attempt to oxidize the 2,3-dihydroindole **27f**. Indeed, by using DDQ as oxidizing agent (ratio **27f**: DDQ 1: 2.25, toluene, reflux 5 h), the expected indole **28** was obtained in 66% yield (Scheme 7).

#### 2.1.7. Pyrazoles

**Scheme 8:**
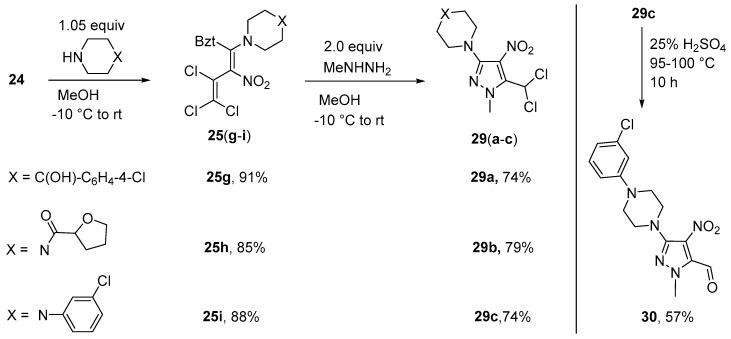
Synthesis of pyrazoles **29**, **30** and benzotriazoles **25**.

In the past ten years, different ways to synthesize persubstituted 4-nitropyrazoles have been developed [9,18,26,28,46]. Some of these compounds show high biological activities: they can modulate the biological activity of IFNs-I [47], are active against mycobacterial infections including tuberculosis [48], and are able to reduce prime virulence factors of *Vibrio cholerae* [49,50]. In the course of our recent studies, the three 1-amino-1-benzotriazolyldienes **25g**–**i** were synthesized in good yields (85–91%) from bis(benzotriazole) **24** using 4-(4-chlorophenyl)piperidin-4-ol, piperazin-1-yl-(tetrahydrofuran-2-yl)methanone and 1-(3-chlorophenyl)piperazine, respectively. The treatment of dienes **25g**–**i** with a twofold excess of methylhydrazine in methanol at −10 °C to rt led to the formation of new nitropyrazoles **29a**–**c** in good yields (74–79%). By saponification of the dichloromethyl group in compound **29c** by means of 25% aqueous sulfuric acid at 95–100 °C, the aldehyde **30** was obtained (57%) (Scheme 8) [26]. Other 4-nitropyrazoles are known as hypoxia-selective cytotoxins and radiosensitizers [51]; they can be useful as herbicides [52,53] or show psychosedative actions [54].

#### 2.1.8. *4H*-Pyrido [1,2-*a*]Pyrimidines

**Scheme 9:**
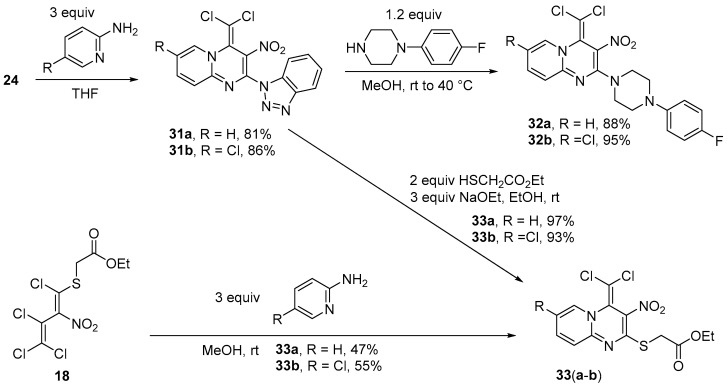
Synthesis of 4*H*-pyrido[1,2-*a*]pyrimidines **31**–**33**.

Recently, a new pathway for the synthesis of 4-(dichloromethylene)-3-nitro-4*H*-pyrido[1,2-*a*]pyrimidines with a unique substitution pattern at the pyrimidine ring was developed [28]. Starting with bis(benzotriazole) **24**, using a threefold excess of 2-aminopyridines two enamines **31a**,**b** were formed in THF in 81–86% yield. The benzotriazole unit in pyridopyrimidines **31**, activated through the neighboring nitro group, acts as a very good leaving group and can be replaced with different nucleophiles at mild reaction conditions. Thus, treatment of compounds **31** with 1-(4-fluoro-phenyl)piperazine in methanol at 40 °C led to the piperazino-substituted pyridopyrimidines **32** in excellent yields. A S_N_Vin process in pyridopyrimidines **31** under action of S-nucleophiles such as ethyl 2-mercaptoacetate using sodium ethanolate as base furnished sulfane **33a,b** in 93–97% yield. Another possibility to form compounds **33a**,**b** is reaction of sulfane **18** with 2-aminopyridines. At optimum conditions (MeOH, threefold excess of amino-pyridines, rt), the yields of pyridopyrimidines **33** reached 47–55% (Scheme 9). Similar pyrido[1,2-*a*]pyrimidines show antiviral [55], antithrombotic [56], and antibacterial [57,58,59,60] activities.

#### 2.1.9. Benzo[*h*]quinolines

**Scheme 10:**
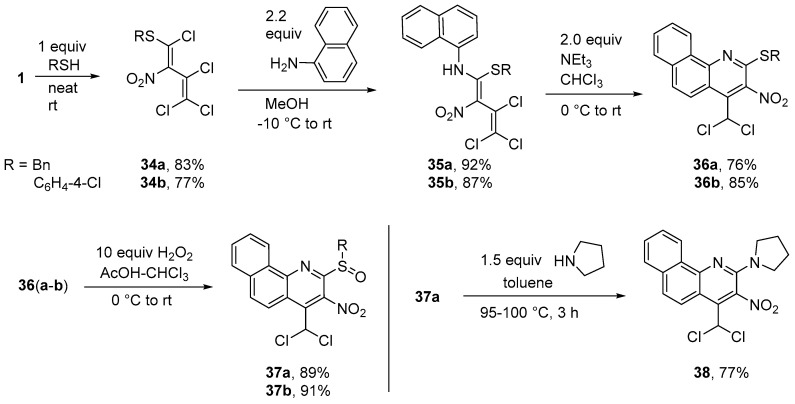
Synthesis of benzo[*h*]quinolines **36**–**38** and nitrodienes **34**, **35**.

In the course of our studies on polyhalogenated nitrobutadienes, a new ring closure approach to benzo[*h*]quinolines was also developed [61]. Starting from nitrodiene **1** in three steps, the target benzo[*h*]quinolines with a unique substitution pattern at the pyridine ring were obtained in good yields. In detail, after mono substitution of one chlorine group in diene **1** sulfanes **34a**,**b** were formed as single isomers each in yields of about 80% according to the literature [62] for the benzyl derivative **34a** and literature [9] for the 4-chlorophenyl derivative **34b**. In a second step, we synthesized the aminothiobutadienes **35a**,**b** by interaction of sulfane **34a**,**b** with an excess of 1-naphthylamine in methanol at −10 °C to rt. Dienes **35a**,**b** were also formed (76–85% yield) as single *E–*isomers due to the stable six membered hydrogen bridge between the amino and nitro group. Finally, the ring closure at optimum conditions (twofold excess of triethylamine as a base) proceeded under formation of the expected benzo[*h*]quinolines **36a**,**b** in good yields (76–85%). The assumed mechanism for the formation of benzo[*h*]quinolines is depicted in the literature [61]. Benzo[*h*]quinolines are a precious class of organic compounds and show interesting biological properties [63,64,65,66,67]. Oxidation of quinolines **36a**,**b** with excess of hydrogen peroxide in a mixture of acetic acid and chloroform lead to the formation of sulfoxides **37a**,**b** in 89–91% yield. The sulfinyl group is known to be a good leaving group [68,69,70]. Indeed, treatment of sulfoxide **37a** with an excess of pyrrolidine in toluene at 100 °C furnished amino derivative **38** in 77% yield (Scheme 10).

#### 2.1.10. Isothiazoles

**Scheme 11:**
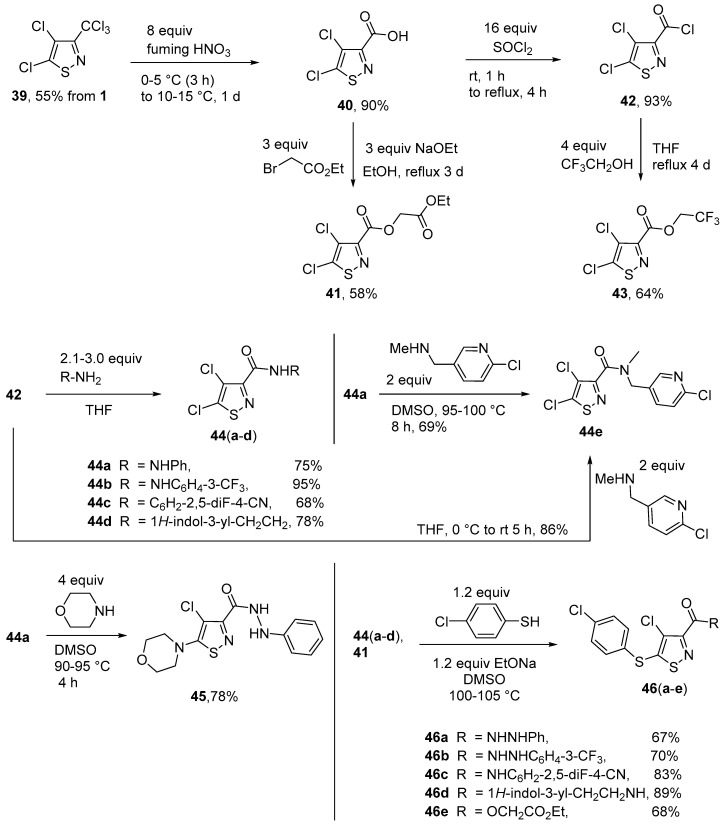
Synthesis of isothiazoles **39**–**46**.

The isothiazole **39** was obtained from nitrodiene **1** upon treatment with elemental sulfur at 200 °C [71]. Subsequent reaction with fuming nitric acid provided the 4,5-dichloroisothiazole-3-carboxylic acid (**40**) **[72]**, which could be easily converted into the corresponding acid chloride **42** with thionyl chloride (93% yield) [28]. Acid **40** reacts with ethyl 2-bromoacetate in the presence of sodium ethanolate under reflux conditions to ester **41** in 58% yield. The esterification of acid chloride **42** with a fourfold excess of 2,2,2-trifluoroethan-1-ol in refluxing THF resulted in the formation of a 2,2,2-trifluoroethyl 4,5-dichloroisothiazole-3-carboxylate (**43**) (64% yield). Acid chloride **42** smoothly reacted with aromatic and aliphatic amines to provide the corresponding amides **44a**–**d** in 68–95% yield. The reaction of chloride **42** with 2 equivalents of 1-(6-chloropyridin-3-yl)-*N*-methyl-methanamine in THF furnished amide **44e** as mixture of two rotamers in relation 10: 6 with a total yield of 86%. An alternative way to obtain amide **44e** is the interaction of carbohydrazide **44a** with 1-(6-chloropyridin-3-yl)-*N*-methylmethanamine at harder reactions conditions (DMSO, 95–100 °C). In this case, amide **44e** is formed as a mixture of two rotamers (relation 10:6), with a total yield of 69%. Reacting hydrazide **44a** with a fourfold excess of morpholine (DMSO, 90–95 °C) did not lead to the formation of a product similar to amide **44e**. Instead, upon substitution of a chlorine group in 5 position of the heterocyclic ring, an isothiazole **45** was formed (78% yield). These amides **44a**–**e** and **45** are interesting candidates for biological testing, as amides of 4-chloroisothiazol-3-carboxylic acid have been shown to exhibit high antibacterial activity [73,74,75]. Finally, we investigated the interaction of amides **44a**–**d** and ester **41** with S*–*nucleophiles such as 4-chlorobenzenethiol. In all cases, reaction products at 5 position of the heterocyclic ring were formed. Under optimized reaction conditions, the yields of the 5-((4-chlorophenyl)thio)isothiazoles **46a**–**e** were in the range of 67–89% (Scheme 11).

#### 2.1.11. *4*,5-Dihydroisoxazoles

**Scheme 12:**
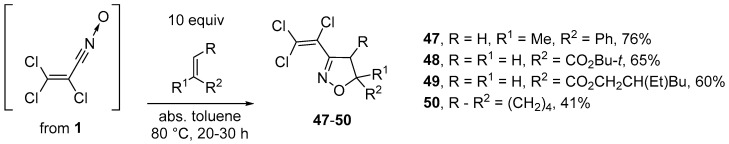
Synthesis of 4,5-dihydroisoxazoles **47**–**50**.

Recently, we developed a novel, fairly general method for the synthesis of dihydroisoxazoles with a chlorinated side chain in 3-position, starting from *gem*-dichloronitroalkenes [76]. In particular, reaction of nitrodiene **1** with a base leads to the formation of 2,3,3-trichloroprop-2-enenitrile oxide. Trapping of this nitrile oxide with an alkene resulted in 4,5-dihydroisoxazole derivatives **47**–**50**, due to a 1,3-dipolar cycloaddition. In detail, reaction of nitrodiene **1** with prop-1-en-2-ylbenzene at optimized conditions furnished 4,5-dihydroisoxazole **47** in 76% yield. The interaction of the acrylonitrile oxide **1** with *tert*-butyl acrylate and 2-ethylhexyl acrylate at similar reactions conditions provided alkyl 3-(1,2,2-trichlorovinyl)-4,5-dihydroisoxazole-5-carboxylates **48** and **49** (65 and 60% yield). Upon treatment of diene **1** with cyclohexene, the hexahydrobenzo-[*d*]isoxazole **50** was isolated in 41% yield (Scheme 12). The yields of **47**–**49** are quite similar to certain compounds in [76], whereas for **50** (in analogy to the reaction with cyclopentene [76]), the yield is lower as expected. 4,5-Dihydroisoxazoles were found to exhibit potent cytotoxic, antineoplastic [77], and antimalarial activity [78]. Additionally, they can be used as androgenic or antiandrogenic agents or androgen receptor modulators [79] and show antibacterial and antifungal activities [80].

#### 2.1.12. Thiophenes

In the course of studying nitroperchlorobutadiene **1** as a versatile building block for the directed synthesis of a range of persubstituted heterocycles, we also developed a three-step synthesis to persubstituted 3-amino-4-nitrothiophenes [81]. Incorporating both, an enamine and a thioketene unit, these thiophenes are very electron-rich heterocycles with a unique substitution pattern. Starting from **1**, the piperazine derivative **52** was obtained in 90% yield via the dithiolane **51**. The push-pull substituted thiophene **53** was efficiently accessible in 85% yield upon treatment of dithiolane **52** with sodium hydroxide using DMSO as solvent. The regioselective *ipso*-formylation of the 2-chloro-thiophene **53** under Vilsmeier-Haack conditions led to the carbaldehyde **54** (64% yield), according to [82].

**Scheme 13:**
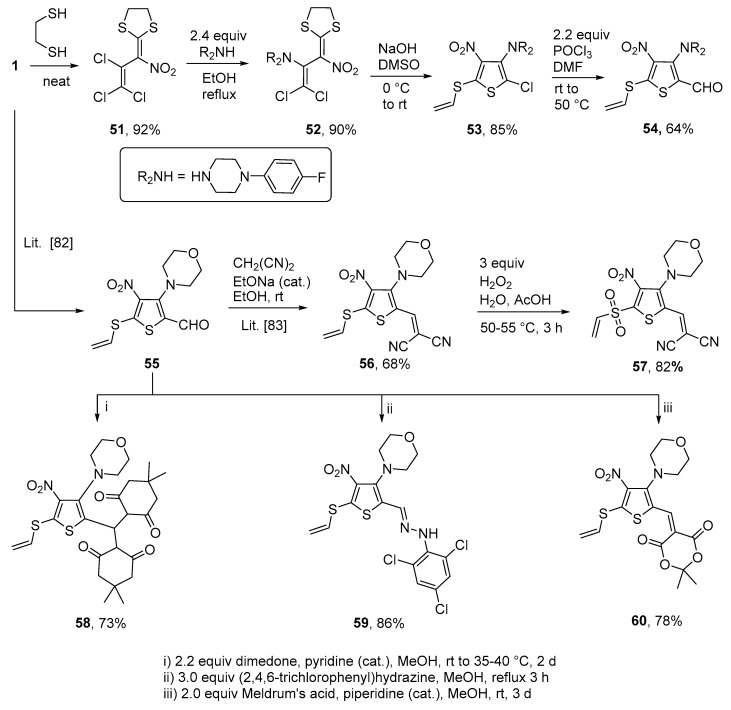
Synthesis of thiophenes **53**–**60** and dithiolanes **51**, **52**.

The next successive steps were performed with the most stable derivative 3-morpholino-4-nitro-5-(vinylthio)thiophene-2-carbaldehyde (**55**) [82]. Knoevenagel condensation of thiophene **55** with malononitrile in ethanol in the presence of a catalytic amount of sodium ethanolate gave the *gem*-dicyanovinylthiophene **56** in 68% yield [83]. Oxidation of the vinylsulfanylthiophene **56** with threefold excess of hydrogen peroxide in acetic acid at 50–55 °C furnished sulfone **57** in 82% yield. Interaction of carbaldehyde **55** with an excess of dimedone, (2,4,6-trichlorophenyl)hydrazine and Meldrum’s acid in methanol led to the formation of bis(5,5-dimethylcyclohexane-1,3-dione) **58**, vinylsulfanyl-thiophene **59** and 1,3-dioxane-4,6-dione **60**, respectively (Scheme 13).

2-Chloro-3-amino-4-nitro-5-(vinylsulfanyl)thiophenes similarly to compound **53** were identified as anti-HIV compounds to treat drug-resistant retroviral infections [84] and show antiviral activity [85]. Other 3(4)-nitrothiophenes can be used in fungicidal and/or bactericidal compositions [86], and show insecticidal and acaricidal activity [87].

### 2.2. Biological Activity of the Polyhalonitrobutadiene Derivatives

Evaluation of the biological activity of the chosen polyhalonitrobutadiene derivatives showed that most of them did not display antibacterial or cytotoxic effects, i.e., residual growth or viability after incubation for 1 and 3 days, respectively, were higher than 50%. Tables with all primary screening data are shown in Supplementary Figures S204–S205. None of the derivatives showed an antibacterial activity against the uropathogenic *Escherichis coli* strain UPEC 796, whereas some had antibacterial activity against *Staphylococcus aureus*. The cytotoxic activity of nine compounds could be proven, as these compounds had IC_50_–values < 50 µM in the viability assay. Among those compounds was the “conjugate” **23b** of the pyrazole **22** and the thiazolidinone **19b**. Whereas the compound series **21** was more or less completely inactive, introduction of the pyrazole group proved successful. In particular, the introduction of a CF_3_ substituent resulted in a compound with significant cytotoxicity (IC_50_ = 6.2 ± 1.8 µM). Similarly, among the pyrimidines **27**, **28,** the most potent derivatives were those with the aromatic residues at the nitropyrimidine–core, namely **27c** and **28** with IC_50_-values of 1.5 ± 0.4 µM and 1.05 ± 0.2 µM, respectively. The non-aromatic nature of the ring next to the pyrimidine core in **27f** prevented the cytotoxic activity. Following the synthesis route of the pyridopyrimidines **32** and **33** revealed that the precursor with the leaving group benzotriazole was the only cytotoxic compound (IC_50_ = 6.0 and 5.7 ± 1.0 µM), and that cytotoxicity was lost, when the benzotriazole group was replaced. In addition, the benzo[*h*]quinolines **36**, **38** lost the cytotoxic activity, which was still observed for the intermediate naphthalene-aminothiobutadiene **35**. All tested derivatives of both the groups of isothiazoles and dihydroisoxazoles were inactive, whereas among the thiophenes, the derivatives with a cyclic dione residue **58** and **60** represented cytotoxic compounds (IC_50_ = 3.1 ± 0.4 µM and 20.1 ± 3.9 µM). Obviously, the morpholino-nitrothiophene structure was not sufficient for biological activity as compound **59** was completely inactive.

## 3. Experimental

### 3.1. General Information

General Remarks: Solvents and reagents were used as received from commercial sources without further purification. TLC was performed with Merck aluminum-backed TLC plates with silica gel 60, F254. Flash column chromatography was performed with Macherey–Nagel silica gel 60 M (0.040–0.063 mm) with appropriate mixtures of petroleum ether (PE, boiling range 60–70 °C) and ethyl acetate as eluents. Melting points (m.p.) were determined in capillary tubes with a Büchi B-520 instrument and were not corrected. FTIR spectra were recorded with a Bruker “Alpha-T” spectro-meter with solid compounds measured as KBr pellets. ATR-IR spectra were measured on the same instrument with a Bruker “Alpha Platinum ATR” single reflection diamond ATR module. ^1^H NMR and ^13^C NMR spectra at 600 and 150 MHz, respectively, were recorded with an “Avance III” 600 MHz FT–NMR spectrometer (Bruker, Rheinstetten, Germany). ^1^H NMR and ^13^C NMR spectra at 400 and 100 MHz, respectively, were recorded with an “Avance” 400 MHz FT–NMR spectrometer (also Bruker). ^1^H NMR and ^13^C NMR spectra at 200 and 50 MHz, respectively, were recorded with an DPX 200 spectrometer (also Bruker). ^14^N and ^15^N NMR spectra were measured at their appropriate resonance frequency on the aforementioned spectrometers; ^15^N measurements were taken as gs-^1^H,^15^N–HSQC or –HMBC experiments with inverse detection. ^1^H and ^13^C NMR spectra were referenced to the residual solvent peak: CDCl_3_, δ = 7.26 (^1^H) and 77.0 ppm (^13^C); DMSO-d_6_, δ = 2.50 (^1^H), and 39.7 ppm (^13^C). Mass spectra were obtained with a Hewlett–Packard MS 5989B spectrometer, usually in direct mode with electron impact (70 eV). For chlorinated and brominated compounds, all peak values of molecular ions and fragments refer to the isotope ^35^Cl and ^79^Br. High resolution mass spectra were recorded with a Waters mass spectrometer “VG Autospec” (EI), with a WATERS mass spectrometer “Q-Tof Premier” coupled with a Waters “Acquity UPLC” (ESI), or with a Micromass mass spectrometer “LCT” coupled with a Waters “Alliance 2965 HPLC” (ESI) at the Institute of Organic Chemistry, Leibniz University of Hannover and at the Georg-August University of Göttingen.

### 3.2. Synthesis

Pentachloro-2-nitro-1,3-butadiene (**1**) was prepared from 2*H*-pentachloro-1,3-butadiene in 53% yield (b.p. 69–71 °C/1 mbar) according to the literature [3,4]. (*Z*)- and (*E*)*-*4-Bromotetrachloro-2-nitrobuta-1,3-diene (**2**) was obtained from (*Z*)- and (*E*)*-*1-bromotetrachlorobuta-1,3-diene in 56% yield (b.p. 84–86 °C/1.3 mbar) [5]. (*Z*)-1,1,4-Trichloro-2,4-dinitrobuta-1,3-diene (**3**) was synthetized from the (*Z*)- and (*E*)*-*1-bromo-1,4,4-trichlorobuta-1,3-diene in 18% yield, m.p. 70–71 °C [6].

*Synthesis of 4-methyl-2-(2,3,3-trichloro-1-nitroprop-2-en-1-ylidene)-2,3-dihydro-1,3-benzoxazole* (**4a**) *(General method).* At −40 °C, a solution of nitrodiene **1** (2.71 g, 10.0 mmol) in 5 mL methanol (MeOH) was added dropwise to a suspension of 2-amino-3-methylphenol (3.94 g, 32.0 mmol) in 30 mL MeOH within 5 min. The resulting mixture was kept for 1 h at this temperature, and was then allowed to reach room temperature (r.t.). After 5 h stirring, the mixture was poured into a cold solution (0 °C) of 5 mL conc. HCl in 250 mL of water. After 20 min, the precipitate was filtered off, washed with cold water (3 × 40 mL) and diethyl ether (2 × 10 mL). Drying in vacuo gave 2.57 g of oxazole **4a**, yield 80%, yellowish solid m.p. 149–151 °C; IR (KBr) ν_max_ = 3089, 1612, 1382, 1079, 968, 520 cm^−1^; ^1^H NMR (200 MHz, CDCl_3_), δ = 2.61 (3H, s, CH_3_ Ph), 7.21–7.33 (2H, m, H Ph), 7.36–7.41 (1H, m, H Ph), 11.78 (1H, s, NH) ppm; ^13^C NMR (50 MHz, CDCl_3_) δ = 16.6 (CH_3_), 107.0 (C NO_2_), 108.7 (CH), 120.2 (CCl_2_), 123.4 (C Me), 125.4 (CH), 127.4, 127.6 (CH), 128.2, 146.6, 159.0 ppm; MS *m/z* (*I*_rel_, %): 320 [M^+^] (4), 285 [M − Cl]^+^ (10), 274 [M-NO_2_]^+^ (65), 239 [M-Cl-NO_2_]^+^ (100); HRMS (ESI^−^) *m/z* calcd for C_11_H_6_N_2_O_3_Cl_3_ [M − H]^−^: 318.9444; found: 318.9446.

*5-Methyl-2-(2,3,3-trichloro-1-nitroprop-2-en-1-ylidene)-2,3-dihydro-1,3-benzoxazole* (**4b**). Same procedure as for **4a**, **b**ut using 2-amino-4-methylphenol (3.94 g, 32.0 mmol). Yield 2.18 g (68%), yellowish solid, m.p. 168–170 °C. IR (KBr) ν_max_ = 3380, 1612, 1434, 1375, 1056, 922 cm^−1^. ^1^H NMR (400 MHz, DMSO-d_6_) δ = 2.42 (3H, s, CH_3_ Ph), 7.20 (1H, d, *J* = 8.3 Hz, H Ph), 7.36 (1H, s, H Ph), 7.69 (1H, d, *J* = 8.3 Hz, H Ph), 13.64 (1H, s, NH) ppm; ^13^C NMR (100 MHz, DMSO-d_6_) δ = 21.2 (CH_3_), 105.4 (C NO_2_), 111.0 (CH), 113.6 (CH), 122.1 (CCl_2_), 126.0 (CH), 126.6, 129.8, 136.4, 144.8, 158.0 (NCO) ppm. MS *m/z* (*I*_rel_, %): 320 [M^+^] (2), 285 [M-Cl]^+^ (4), 274 [M-NO_2_]^+^ (55), 239 [M-Cl-NO_2_]^+^ (100), 204 [M-2Cl-NO_2_]^+^ (14); HRMS (ESI^−^) *m/z* calcd for C_11_H_6_N_2_O_3_Cl_3_ [M − H]^−^: 318.9453; found: 318.9444.

*Synthesis of [{1-(1,3-benzoxazol-2-yl)-3,3-dichloro-2-[4-(dimethylamino)pyridinium-1-yl]prop-2-en-1-yli- dene}(oxido)-l5-azanyl]oxidanide* (**5a**) *(General method)*. To a suspension of benzoxazole **4b** (0.322 g, 1.00 mmol) in 15 mL MeOH at 0 °C, a solution of 4-dimethylaminopyridine (DMAP) (0.257 g, 2.1 mmol) in 3 mL MeOH was added dropwise. The mixture was stirred for 1 h at 0 °C and at r.t. for 12 h. Subsequently, the precipitate was filtered off at 0 °C and washed with water (2 × 10 mL) and cold MeOH (5 mL). Finally, the product **5a** was dried in vacuo. Yield 0.301 g (74%), yellowish solid, m.p. 189–190 °C. IR (ATR) ν_max_ = 1641, 1531, 1355, 1139, 1055, 790 cm^−1^; ^1^H NMR (400 MHz, DMSO-d_6_) δ = 2.38 (3H, s, CH_3_ Ph), 3.23 (6H, s, N(CH_3_)_2_), 6.99 (1H, d, *J* = 7.8 Hz, H Ph), 7.06 (2H, d, *J* = 6.7 Hz, H pyr), 7.33 (1H, s, H Ph), 7.43 (1H, d, *J* = 7.9 Hz, H Ph), 8.29 (2H, d, *J* = 6.7 Hz, H pyr) ppm; ^13^C NMR (100 MHz, DMSO-d_6_) δ = 39.7 (CH_3_), 40.2 (NCH_3_), 103.7, 107.3 (CH), 109.3 (CH), 117.8 (CH), 122.7, 123.6 (CH), 133.2, 135.8, 142.3 (CH), 142.5, 147.8, 156.4, 160.9 ppm; MS *m/z* (*I*_rel_, %): 406 [M^+^] (1), 273 [M-benzoxazole-H]^+^ (5), 238 [M-benzoxazole-HCl]^+^ (3), 122 [DMAP]^+^ (73), 100 (100); HRMS (ESI^+^) *m/z* calcd for C_18_H_17_N_4_O_3_Cl_2_ [M + H]^+^: 407.0672; found: 407.0675.

*[{1-(1,3-Benzoxazol-2-yl)-3,3-dichloro-2-[4-(morpholin-4-yl)pyridinium-1-yl]prop-2-en-1-ylidene} (oxido)-l5-azanyl]oxidanide* (**5b**). Following the typical procedure for **5a**, using **4b** (0.322 g, 1.00 mmol) and 4-(4-morpholinyl)pyridine (0.345 g, 2.1 mmol) at −18 °C and holding at this temperature for 3 h. Yield 0.270 g (62%), yellowish solid, m.p. 180–181 °C. IR (ATR) ν_max_ = 1640, 1548, 1348, 1253, 1156, 792 cm^−1^. ^1^H NMR (400 MHz, DMSO-d_6_) δ = 2.38 (3H, s, CH_3_ Ph), 3.73 (8H, s, H morph), 7.00 (1H, dd, *J* = 8.1 Hz, *J* = 1.3 Hz, H Ph), 7.25 (2H, d, *J* = 7.8, H pyr), 7.34 (1H, s, H Ph), 7.43 (1H, d, *J* = 8.2, H Ph), 8.34 (2H, d, *J* = 7.6, H pyr) ppm; ^13^C NMR (100 MHz, DMSO-d_6_) δ = 21.3 (CH_3_), 46.6 (N(CH_2_)_2_), 65.6 (O(CH_2_)_2_), 103.8, 107.5 (CH), 109.3 (CH), 117.8 (CH), 122.9, 123.9 (CH), 133.2, 135.7, 142.5, 143.0 (CH), 147.8, 156.1, 160.8 ppm; MS *m/z* (*I*_rel_, %): 448 [M^+^] (1), 325 [M-HCl-morpholine]^+^ (1), 316 [M-methylbenzoxazole]^+^ (1), 269 [M-pyridine+H]^+^ (4), 165 (100), 132 [methylbenzoxazole]^+^ (12); HRMS (ESI^+^) *m/z* calcd for C_20_H_19_N_4_O_4_Cl_2_ [M + H]^+^: 449.0778; found: 449.0780.

*1-(Pyridin-4-yl)pyrrolidin-2-one* as starting material for the synthesis of azinate **5c** was obtained from 4-aminopyridine and 4-chlorobutanoyl chloride according to the literature [88]. Yield 65%, colorless liquid. ^1^H NMR (600 MHz, CDCl_3_) δ = 2.17 (2H, t, *J* = 7.7 Hz, CH_2_ pyrro), 2.61 (2H, t, *J* = 8.2 Hz, CH_2_ pyrro), 3.82 (2H, t, *J* = 7.1 Hz, CH_2_ pyrro), 7.57 (2H, d, *J* = 4.9 Hz, H pyr), 8.50 (2H, d, *J* = 4.9 Hz, H pyr) ppm; ^13^C NMR (150 MHz, CDCl_3_) δ = 17.6 (CH_2_), 32.8 (CH_2_), 47.4 (CH_2_), 112.8 (CH), 145.8, 150.4 (CH), 175.2 (CO) ppm; MS *m/z* (*I*_rel_, %): 162 [M^+^] (31), 147 [M-OH]^+^ (25), 119 [M-CH_2_CHO]^+^ (10), 107 [M-CH_2_CH_2_CO+H]^+^ (100).

*[{1-(1,3-Benzoxazol-2-yl)-3,3-dichloro-2-[4-(2-oxopyrrolidin-1-yl)pyridinium-1-yl]prop-2-en-1-ylidene}- (oxido)-l5-azanyl]oxidanide* (**5c**). Same procedure as for **5a**, using 1-(pyridin-4-yl)pyrrolidin-2-one (0.341 g, 2.1 mmol) at −18 °C and holding this temperature for 2 h. Stirring was continued for 24 h at r.t. Yield 67%, yellow solid, m.p. 157–158 °C. IR (ATR) ν_max_ = 1733, 1627, 1550, 1349, 1257, 1157 cm^−1^. ^1^H NMR (600 MHz, DMSO-d_6_) δ = 2.13 (2H, t, *J* = 7.6 Hz, CH_2_ pyrro), 2.39 (3H, s, CH_3_ Ph), 2.67 (2H, t, *J* = 8.0 Hz, CH_2_ pyrro), 3.99 (2H, t, *J* = 7.2 Hz, CH_2_ pyrro), 7.01 (1H, dd, *J* = 8.2 Hz, *J* = 0.8 Hz, H Ph), 7.35 (1H, s, H Ph), 7.45 (1H, d, *J* = 8.2 Hz, H Ph), 8.25 (2H, d, *J* = 7.0, H pyr), 8.99 (2H, d, *J* = 7.5 Hz, H pyr) ppm; ^13^C NMR (150 MHz, DMSO-d_6_) δ = 17.1 (CH_2_), 21.2 (CH_3_), 32.7 (CH_2_), 48.1 (CH_2_), 104.0, 109.4 (CH), 113.9 (CH), 117.9 (CH), 123.8 (CH), 124.0, 133.3, 136.2, 142.5, 145.7 (CH), 147.8, 152.6, 160.5, 177.5 ppm; ^15^N NMR (43.4 MHz, DMSO-d_6_, doped with nitromethane (0.0 ppm)) δ = −187.0 (N-pyridine), -235.2 (N-pyrrolidine) ppm, other N-atoms could not be detected; MS *m/z* (*I*_rel_, %): 446 [M^+^] (1), 411 [M-Cl]^+^ (1), 162 [pyridinyl–pyrrolidinone]^+^ (45), 133 [methylbenzoxazole+H]^+^ (6), 107 (100); HRMS (ESI^+^) *m/z* calcd for C_20_H_17_N_4_O_4_Cl_2_ [M + H]^+^: 447.0621; found: 447.0623.

*[{1-(1,3-Benzoxazol-2-yl)-3,3-dichloro-2-[3-(1-methylpyrrolidin-2-yl)pyridinium-1-yl]prop-2-en-1-ylidene}- (oxido)-λ^5^-azanyl]oxidanide* (**5d**). To a suspension of benzoxazole **4b** (0.322 g, 1.0 mmol) in MeOH (10 mL) at −18 °C, a solution of (-)-nicotine (0.324 g, 2.0 mmol) in MeOH (5 mL) was added dropwise. Subsequently, the mixture was stirred at −18 °C for 2 h and at r.t. for 18 h. After completion of the reaction water (10 mL), NaHCO_3_ (0.184 g, 2.2 mmol) were added and the mixture was stirred for 10 min, following extraction with chloroform (3 × 15 mL). The combined organic phases were dried over calcium chloride and purified by column chromatography using DCM–petroleum ether (1:1). The product **5d** was dried in vacuo. Yield 0.237 g (53%), yellow solid, m.p. 96–98 °C. IR (ATR) ν_max_ = 1622, 1516, 1354, 1259, 1141, 1062 cm^−1^. ^1^H NMR (400 MHz, CDCl_3_) δ = 1.64–1.77 (1H, m, CH_2_ pyrro), 1.83–2.01 (2H, m, NCH_2_), 2.26 (3H, s, NCH_3_ pyrro), 2.43 (3H, s, Ph-CH_3_), 2.43–2.49 (1H, m, CH_2_ pyrro), 3.24–3.34 (1H, m, CH_2_ pyrro), 3.42–3.57 (1H, m, CH_2_ pyrro), 3.63–3.89 (1H, m, CH_2_ pyrro), 7.00 (1H, dd, *J* = 1.0 Hz, *J* = 8.2 Hz, CH_3_C-CH), 7.37 (1H, s, H Ph), 7.39 (1H, d, *J* = 8.2 Hz, OCCH), 7.96 (1H, dd, *J* = 5.6 Hz, *J* = 7.8 Hz, H pyr), 8.48 (1H, d, *J* = 7.8 Hz, H pyr), 9.03 (1H, d, *J* = 5.6 Hz, H pyr), 9.15 (1H, s, H pyr) ppm; ^13^C NMR (100 MHz, CDCl_3_) δ = 21.5 (CH_3_ Ph), 23.1 (CH_2_), 35.8 (CH_2_), 40.3 (CH_3_ pyrro), 56.6 (CH_2_), 67.1 (CH pyrro), 104.6 (CNO_2_), 109.8 (CH Ph), 118.0 (CH Ph), 124.0 (CH Ph), 127.0 (CH pyr), 128.5 (CCl_2_), 133.7 (CCH_3_), 136.3, 142.2, 143.6 (CH pyr), 145.2 (CH pyr), 145.5 (CH pyr), 145.7, 148.3 (C-pyrro), 160.1 (NCO) ppm; MS (ESI^+^) *m/z* calcd for C_21_H_21_N_4_O_3_Cl_2_ [M + H]^+^: 447.1; found 447.1.

*2-[3-Bromo-2,3-dichloro-1-nitroprop-2-en-1-ylidene]-4-methyl-2,3-dihydro-1,3-benzoxazole* (**6**). Same proce-dure as for **4a**, but starting from **2** (316 mg, 1.00 mmol, a 47: 53 mixture of isomers) using 2-amino-3-methylphenol (394 mg, 3.20 mmol). A 1:1 mixture of isomers was obtained. Yield 73%, yellowish solid, m.p. 145–146 °C. IR (KBr) ν_max_ = 3329, 1620, 1571 (NO_2_), 1376, 1320, 1047 cm^−1^; ^1^H NMR (200 MHz, CDCl_3_) δ = 2.61 (3H, s, CH_3_ Ph), 7.22–7.41 (3H, m, H Ph), 11.77 (1H, s, NH) ppm; ^13^C NMR (50 MHz,CDCl_3_) δ = 16.6 (CH_3_); 106.9 (CNO_2_); 108.7 (CH); 114.9, 115.8 (CClBr); 121.8, 123.7 (CCl); 123.4 (C Me); 125.4 (CH), 127.4, 127.6 (CH), 146.7, 158.7, 158.9 (NCO) ppm; MS *m/z* (*I*_rel_, %): 364 [M^+^] (2), 329 [M − Cl]^+^ (4), 318 [M-NO_2_]^+^ (19), 285 [M − Br]^+^ (25), 283 [M-Cl-NO_2_]^+^ (50), 239 [M-Br-NO_2_]^+^ (100); HRMS (ESI^−^) *m/z* calcd for C_11_H_6_N_2_O_3_Cl_2_Br [M − H]^−^: 362.8944; found: 362.8948.

*2-((Z)-3-chloro-1,3-dinitroallylidene)-5-methyl-2,3-dihydro-1H-benzo[d]imidazole* (**7a**). Same procedure as for **4a**, but starting from **3** (247 mg, 1.00 mmol, only *Z*-isomer) using 4-methylbenzene-1,2-diamine (257 mg, 2.10 mmol). Yield 93%, red solid, m.p. 163–165 °C. IR (KBr) ν_max_ = 3330, 1602, 1577 (NO_2_), 1410, 1268, 987 cm^−1^. ^1^H NMR spectrum (200 MHz, DMSO-d_6_) δ = 2.49 (3H, s, CH_3_ Ph), 7.36 (1H, d, *J* = 8.5 Hz, H Ph), 7.58 (1H, s, H Ph), 7.67 (1H, d, *J* = 8.5 Hz, H Ph), 9.14 (1H, s, CH), 14.45 (2H, s, NH) ppm; ^13^C NMR (50 MHz, DMSO-d_6_) δ = 21.4 (CH_3_), 106.9 (C NO_2_), 113.4 (CH), 113.6 (CH), 120.1 (CClNO_2_), 127.5 (CH), 129.1 (C Me), 129.2 (CH), 131.2, 136.1, 142.3 (NCN) ppm; MS *m/z* (*I*_rel_, %): 296 [M^+^] (5), 250 [M-NO_2_]^+^ (55), 233 [M-NO_2_-OH]^+^ (12), 204 [M-2NO_2_]^+^ (60), 169 [M-2NO_2_-Cl]^+^ (48), 157 (100); HRMS (ESI^−^) *m/z* calcd for C_11_H_8_N_4_O_4_Cl [M − H]^−^: 295.0240; found: 295.0248.

*2-(3-Chloro-1,3-dinitroprop-2-en-1-ylidene)-5-methyl-2,3-dihydro-1,3-benzoxazole* (**7b**). Same procedure as for **4a**, but using **3** (247 mg, 1.00 mmol, only Z-isomer) and 2-amino-4-methylphenol (394 mg, 3.20 mmol). Yield 67%, yellow solid, m.p. 110–112 °C. IR (KBr) ν_max_ = 3250, 1602, 1545 (NO_2_), 1289, 1065, 871 cm^−1^. ^1^H NMR (200 MHz, DMSO-d_6_) δ = 2.45 (3H, s, CH_3_ Ph), 7.25 (1H, d, *J* = 8.3 Hz, H Ph), 7.55 (1H, s, H Ph), 7.63 (1H, d, *J* = 8.5 Hz, H Ph), 9.09 (1H, s, CH), 10.66 (1H, s, NH); ^13^C NMR (50 MHz, DMSO-d_6_) δ = 21.2 (CH_3_), 110.8 (CH), 112.5 (CNO_2_), 118.5 (CClNO_2_), 119.1 (CH), 126.8 (CH), 129.5 (CH), 134.5 (C Me), 139.5, 148.2, 157.2 (NCO) ppm; MS *m/z* (*I*_rel_, %): 297 [M^+^] (3), 251 [M − NO_2_]^+^ (45), 234 [M-NO_2_-OH]^+^ (13), 158 (100); HRMS (ESI^−^) *m/z* calcd for C_11_H_7_N_3_O_5_Cl [M − H]^−^: 296.0072; found: 296.0070.

*2-(3-Chloro-1,3-dinitroprop-2-en-1-ylidene)-4-methyl-2,3-dihydro-1,3-benzoxazole* (**7c**). Same procedure as for **4a**, using **3** (247 mg, 1.00 mmol, only *Z*-isomer) and 2-amino-3-methylphenol (394 mg, 3.20 mmol). Yield 85%, yellow solid, m.p. 102–103 °C. IR (KBr) ν_max_ = 3254, 1625, 1579 (NO_2_), 1520, 1077, 646 cm^−1^. ^1^H NMR (200 MHz, DMSO-d_6_) δ = 2.53 (3H, s, CH_3_ Ph), 7.19 (1H, d, *J* = 7.5 Hz, H Ph), 7.30 (1H, t, *J* = 7.5 Hz, H Ph), 7.52 (1H, d, *J* = 8.0 Hz, H Ph), 9.20 (1H, s, CH), 10.19 (1H, s, NH) ppm; ^13^C NMR (50 MHz, DMSO-d_6_) δ = 16.5 (CH_3_), 108.5 (CH), 113.6 (CNO_2_), 115.9 (CClNO_2_), 125.0 (CH), 125.5 (CH), 130.3 (CH), 140.3, 150.3, 156.3 (NCO) ppm; MS *m/z* (*I*_rel_, %): 297 [M^+^] (3), 251 [M-NO_2_]^+^ (40), 234 [M-NO_2_-OH]^+^ (12), 158 (100); MS (ESI^−^) *m/z* calcd for C_11_H_7_N_3_O_5_Cl [M − H]^−^: 296.0; found: 296.0.

*Synthesis of 2-(3-bromo-2,3-dichloro-1-nitroprop-2-en-1-ylidene)-1,3-diphenylimidazolidine* (**8b**) *(General method).* To a solution of *N*,*N*’-diphenylethane-1,2-diamine (0.446 g, 2.1 mmol) in 10 mL MeOH at −40 °C a solution of nitrodiene **2** (0.316 g, 1.0 mmol) in 5 mL MeOH was added dropwise. After 1 h of stirring at −40 °C, the solution was allowed to reach r.t. and stirred for another 5 h. The precipitate was filtered off, washed with water (3 × 20 mL), MeOH (1 × 10 mL), diethyl ether (2 × 10 mL) and dried in vacuo. A 1:1 mixture of isomers was obtained. Yield 0.410 g (90%), yellow solid, m.p. 222–223 °C. IR (KBr) ν_max_ = 3455, 3059, 1594, 1523, 1295, 761 cm^−1^. ^1^H NMR (200 MHz, DMSO-d_6_) δ = 4.37 (4H, s, CH_2_), 7.25–7.47 (10H, m, H Ph) ppm; ^13^C NMR (50 MHz, DMSO-d_6_) δ = 51.0, 50.9 (CH_2_), 104.4, 105.9 (CNO_2_), 107.2, 107.7 (CBrCl), 122.4, 122.5 (CH), 127.0 (CH), 127.8, 129.5, 129.6 (CH), 139.9, 140.0 (NC Ph), 155.6, 155.7 (NCN) ppm; MS *m/z* (*I*_rel_, %): 453 [M^+^] (3), 418 [M − Cl]^+^ (3), 374 [M-Br]^+^ (45), 339 [M-Cl-Br]^+^ (8), 279 [M-C_2_Cl_2_Br]^+^ (100); HRMS (ESI^+^) *m/z* calcd for C_18_H_15_N_3_O_2_Cl_2_Br [M + H]^+^: 453.9719; found: 453.9728.

*2-(3-Chloro-1,3-dinitroprop-2-en-1-ylidene)-1,3-diphenylimidazolidine* (**8c**). Same procedure as for **8b**, using dinitrodiene **3** (0.247 g, 1.0 mmol). Yield 86%, yellow solid, m.p. 236–237 °C. IR (KBr) ν_max_ = 3054, 1570, 1491, 1349, 1193, 999 cm^−1^. ^1^H NMR (200 MHz, CDCl_3_) δ = 4.58 (4H, s, CH_2_), 7.29–7.33 (4H, m, H Ph), 7.39–7.45 (6H, m, H Ph), 8.69 (1H, s, CH) ppm; ^13^C NMR (50 MHz, DMSO-d_6_) δ = 50.6 (CH_2_), 105.1, 117.8 (CClNO_2_), 123.2 (CH), 128.4 (CH), 129.0 (CH), 130.0 (CH), 136.7 (NC Ph), 158.7 (NCN) ppm; MS *m/z* (*I*_rel_, %): 386 [M^+^] (20), 340 [M − NO_2_]^+^ (60), 305 [M-NO_2_-Cl]^+^ (3), 281 [M-(CH=CClNO_2_)+H]^+^ (20), 247 [M-(CH=CClNO_2_)-2OH]^+^ (100); HRMS (ESI^+^) *m/z* calcd for C_18_H_16_N_4_O_4_Cl [M + H]^+^: 387.0855; found: 387.0865.

*Synthesis of 2-[3,3-dichloro-2-nitro-1-(pyrrolidin-1-yl)prop-2-en-1-ylidene]-1,3-diphenylimidazolidine* (**9**). To a suspension of imidazolidine **8a** (0.387 g, 1.0 mmol) in 10 mL MeOH at r.t. a solution of pyrrolidine (0.356 g, 5.0 mmol) in 5 mL MeOH was added. Subsequently, the mixture was stirred for 3 h at r.t. and 4 h at reflux. After cooling to 10 °C, the pH was adjusted to 6–7 with HCl (5%). The resulting precipitate was filtered off and washed with water (3 × 20 mL), MeOH (2 × 10 mL) and dried in vacuo. Yield 0.223 g (50%), yellow solid, m.p. 156–159 °C. IR (KBr) ν_max_ = 2968, 2868, 1597, 1497, 1347, 908 cm^−1^. ^1^H NMR (200 MHz, DMSO-d_6_) δ = 1.52–1.56 (4H, m, CH_2_ pyr), 2.68–2.73 (4H, m, CH_2_ pyr), 4.29 (4H, s, CH_2_ im), 7.21–7.44 (10H, m, H Ph) ppm; ^13^C NMR (50 MHz, DMSO-d_6_) δ = 25.0 (CH_2_), 50.1 (CH_2_), 51.0 (CH_2_), 98.6 (CCl_2_), 104.7 (CNO_2_), 122.8 (CH), 126.7 (CH), 129.2 (CH), 140.2, 140.6, 157.1 (NCN) ppm; MS *m/z* (*I*_rel_, %): 444 [M^+^] (2), 427 [M-OH]^+^ (2), 408 [M-HCl]^+^ (3), 373 [M-pyrrolidine]^+^ (22), 281 [M-(CCl_2_=C)-pyrrolidine+H]^+^ (20), 264 [M-(CCl_2_=C)-pyrrolidine-O]^+^ (85), 248 [M-(CCl_2_=C)-pyrrolidine-2O]^+^ (100); HRMS (ESI^+^) *m/z* calcd for C_22_H_23_N_4_O_2_Cl_2_ [M + H]^+^: 445.1193; found: 445.1190.

*Synthesis of ethyl 1-(1,1-dichloro-3-{1-[(6-chloropyridin-3-yl)methyl]imidazolidin-2-ylidene}-3- nitroprop-1-en-2-yl)piperidine-4-carboxylate* (**12a**) *(General method)*. To a suspension of compound **11a** (0.384 g, 1.0 mmol) and piperidine-4-carboxylic acid ethyl ester (0.393 g, 2.5 mmol) in 15 mL MeOH at r.t., a solution of sodium ethanolate (0.177 g, 2.6 mmol) in 5 mL MeOH was added, and stirred for 24 h at 35 °C. At r.t. 3 drops of conc. HCl were added before adding 50 mL cold water. The resulting precipitate was filtered off and washed with cold MeOH (2 × 7 mL) and water (3 × 10 mL). The product was dried in vacuo. Yield 0.303 g (60%), yellow solid, m.p. 60–62 °C. IR (KBr) ν_max_ = 3319, 1726, 1561, 1319, 1177, 1043 cm^−1^. ^1^H NMR (200 MHz, DMSO-d_6_) δ = 1.16 (3H, t, *J* = 6.9 Hz, CH_3_ Et), 1.53–1.76 (4H, m, CH_2_ pip), 2.28–2.42 (1H, m, CH pip), 2.73 (4H, s, CH_2_ pip), 3.35 (2H, s, CH_2_ imi), 3.67 (2H, s, CH_2_ imi), 4.06 (2H, q, *J* = 6.9 Hz, CH_2_ Et), 4.37–4.47 (2H, m, pyr-CH_2_-imi), 7.56 (1H, d, *J* = 8.1 Hz, H pyr), 7.81 (1H, d, *J* = 8.2 Hz, H pyr), 8.36 (1H, s, H pyr), 9.43 (1H, s, NH) ppm; ^13^C NMR (50 MHz, DMSO-d_6_) δ = 14.3 (CH_3_), 28.4 (CH_2_), 40.5 (CH), 42.2 (CH_2_), 48.0 (CH_2_ pip), 48.4 (CH_2_), 49.8 (CH_2_), 60.1 (CH_2_), 102.8 (CNO_2_), 103.6 (CCl_2_), 124.4 (CH), 131.4, 138.8 (CH), 141.3, 148.9 (CH), 149.7, 160.1, 174.4 ppm; MS *m/z* (*I*_rel_, %): 503 [M^+^] (3), 467 [M − HCl]^+^ (3), 377 [M-CH_2_pyridine]^+^ (2), 254 [M-C_2_Cl_2_-piperidine]^+^ (18), 126 [CH_2_pyridine]^+^ (100); HRMS (ESI^−^) *m/z* calcd for C_20_H_23_N_5_O_4_Cl_3_ [M − H]^−^: 502.0821; found: 502.0818.

*2-(1,1-Dichloro-3-{1-[(6-chloropyridin-3-yl)methyl]imidazolidin-2-ylidene}-3-nitroprop-1-en-2-yl)- 1,2,3,4-tetrahydroisoquinoline* (**12b**). Same procedure as for **12a**, using **11a** (0.384 g, 1.00 mmol) and 1,2,3,4-tetrahydroisoquinoline (0.368 g, 2.50 mmol) and stirring for 4 h at 50 °C. Yield 85%, orange solid, m.p. 146–148 °C. IR (KBr) ν_max_ = 3318, 2895, 1559, 1460, 1320, 749 cm^−1^. ^1^H NMR spectrum (200 MHz, CDCl_3_) δ = 2.70–3.16 (2H, m, CH_2_ pip), 3.30–3.55 (2H, m, CH_2_ pip), 3.59–3.90 (4H, m, CH_2_ imi), 4.08 (1H, d, J = 15.2 Hz, NCH_2_); 4.26–4.38 (2H, m, CH_2_ pip), 4.67 (1H, d, J = 15.2 Hz, NCH_2_), 6.91–6.97 (1H, m, H Ph), 7.07–7.15 (3H, m, H Ph), 7.32 (1H, d, J = 8.2 Hz, H pyr), 7.60 (1H, dd, J = 8.3 Hz, J = 2.5 Hz, H pyr), 8.30 (1H, d, J = 2.4 Hz, H pyr), 9.56 (1H, s, NH) ppm. ^13^C NMR (50 MHz, CDCl_3_) δ = 29.7 (CH_2_), 41.7 (CH_2_), 48.7 (CH_2_), 49.6 (CH_2_), 49.9 (CH_2_), 51.3 (CH_2_), 105.1, 105.7, 124.7 (CH), 125.7 (CH), 126.7 (CH), 126.3 (CH), 128.8 (CH), 129.6, 133.9, 134.9, 138.2 (CH), 139.7, 148.9 (CH), 151.6, 160.2 ppm; MS *m/z* (I_rel_, %): 479 [M^+^] (2), 443 [M − HCl]^+^ (3), 407 [M-2(HCl)]^+^ (5), 254 [M-C_2_Cl_2_-isoquinoline + H]^+^ (7), 126 [Cl-pyr-CH_2_]^+^ (100); HRMS (ESI^−^) *m/z* calcd for C_21_H_19_N_5_O_2_Cl_3_ [M − H]^−^: 478.0610; found: 478.0606.

*2-[(1,1-Dichloro-3-{1-[(6-chloropyridin-3-yl)methyl]imidazolidin-2-ylidene}-3-nitroprop-1-en-2-yl)sulfanyl]- ethanol* (**12c**). Same procedure as for **12a**, using **11a** (0.384 g, 1.00 mmol), 2-mercaptoethanol (0.086 g, 1.1 mmol) and sodium ethanolate (0.082 g, 1.2 mmol) in EtOH. The mixture was stirred for 12 h at 40 °C. Yield 0.268 g (63%), orange solid, m.p. 185–187 °C. IR (KBr) ν_max_ = 3220, 2974, 1575, 1299, 1125, 844 cm^−1^. ^1^H NMR spectrum (200 MHz, DMSO-d_6_) δ = 2.77 (2H, t, *J* = 6.4 Hz, SCH_2_), 3.53 (2H, t, *J* = 6.4 Hz, CH_2_OH), 3.73 (4H, s, CH_2_), 4.30 (2H, s, NCH_2_), 4.70 (1H, s, OH), 7.53 (1H, d, *J* = 8.3 Hz), 7.75 (1H, dd, *J* = 8.3 Hz, *J* = 2.5 Hz), 8.33 (1H, d, *J* = 2.3 Hz), 9.55 (1H, s, NH) ppm; ^13^C NMR (50 MHz, DMSO-d_6_) δ = 34.9 (CH_2_), 42.4 (CH_2_), 48.9 (CH_2_), 50.8 (CH_2_), 60.1 (CH_2_), 102.8 (CNO_2_), 115.1 (CCl_2_), 124.3 (CH), 131.6, 131.9, 138.1 (CH), 148.0 (CH), 149.5, 159.7 ppm; MS *m/z* (*I*_rel_, %): 424 [M^+^] (1), 254 [M-C_2_Cl_2_-mercaptoethanol + H]^+^ (27), 235 [M − C_2_Cl_2_S-H_2_O]^+^ (12), 126 [Cl-pyr-CH_2_]^+^ (100); HRMS (ESI^−^) *m/z* calcd for C_14_H_14_N_4_O_3_SCl_3_ [M − H]^−^: 422.9858; found: 422.9854.

*1,1-Dichloro-3-{3-[(6-chloropyridin-3-yl)methyl]-1,3-oxazolidin-2-ylidene}-N,N-dimethyl-3-nitroprop-1-en-2-amine* (**12d**). Same procedure as for **12a**, using pyridine **11b** (0.385 g, 1.00 mmol) and dimethylamine (0.366 g, 5.00 mmol) and stirring at r.t. for 5 h. Yield 70%, yellow solid, m.p. 127–129 °C. IR (KBr) ν_max_ = 2868, 1597, 1561, 1298, 1118, 903 cm^−1^. ^1^H NMR (400 MHz, DMSO-d_6_) δ = 2.59 (6H, s, NCH_3_), 3.96–4.10 (2H, m, CH_2_ oxa), 4.52–4.81 (4H, m, CH_2_ oxa, NCH_2_), 7.59 (1H, d, *J* = 7.7 Hz, H pyr), 7.91 (1H, d, *J* = 7.5 Hz, H pyr), 8.41 (1H, s, H pyr) ppm; ^13^C NMR (100 MHz, DMSO-d_6_) δ = 42.0 (CH_3_), 49.3 (CH_2_), 50.8 (CH_2_), 68.2 (CH_2_), 104.0, 105.1, 124.4 (CH), 130.0, 139.8 (CH), 142.1, 149.8 (CH), 150.1, 165.4 ppm; MS *m/z* (*I*_rel_, %): 392 [M^+^] (1), 311 [M − Cl-NO_2_]^+^ (1), 254 [M − C_2_Cl_2_-N(CH_3_)_2_]^+^ (2), 126 [Cl-pyr-CH_2_]^+^ (100); HRMS (ESI^+^) *m/z* calcd for C_14_H_16_N_4_O_3_Cl_3_ [M + H]^+^: 393.0283; found: 393.0280.

*Synthesis of 1-[(4-bromo-1,3,4-trichloro-2-nitrobuta-1,3-dien-1-yl)sulfanyl]-4-fluorobenzene* (**13**). To a solution of nitrodiene **2** (0.316 g, 1.0 mmol) in dry DCM (15 mL) at 0 °C, a solution of 4-fluorothiophenole (0.128 g, 1.0 mmol) in 5 mL of dry DCM was added and stirred for 3 h. After reaching r.t. and further stirring for 24 h the mixture was concentrated and water (20 mL) was added before extraction with chloroform (3 × 10 mL). The product was purified by column chromatography using petroleum ether-ethyl acetate (10:1) and dried in vacuo. A 1:1 mixture of isomers was obtained. Yield 0.301 g (74%), yellow solid, m.p. 98–99 °C. IR (ATR) ν_max_ = 1587, 1527, 1292, 1223, 815, 526 cm^−1^. ^1^H NMR (400 MHz, CDCl_3_) δ = 7.11–7.25 (2H, m, H Ph), 7.48–7.64 (2H, m, H Ph) ppm; ^13^C NMR (101 MHz, CDCl_3_) δ = 115.2, 116.4 (CClBr), 117.0, 117.2 (CH), 124.0, 124.1, 124.2, 124.5, 138.3, 138.4 (CH), 157.3, 157.8 (CClS), 163.4, 165.9 (CF) ppm, CNO_2_ could not be detected; MS *m/z* (*I*_rel_, %): 405 [M^+^] (1), 370 [M − Cl]^+^ (1), 326 [M − Br]^+^ (6), 291 [M − Br-Cl]^+^ (3), 127 [S-Ph-F]^+^ (100); HRMS (ESI^+^) *m/z* calcd for C_10_H_5_NO_2_Cl_3_SBrF [M + H]^+^: 405.8269; found: 405.8286.

*Synthesis of 5-(((2E)-2-(3-bromo-2,3-dichloro-1-nitroallylidene)imidazolidin-1-yl)methyl)-2-chloropyridine* (**14**) and *1,1’-[(4-bromo-3,4-dichloro-2-nitrobuta-1,3-diene-1,1-diyl)disulfanediyl]bis(4-fluorobenzene)* (**15**). A solution of *N*-[(6-chloropyridin-3-yl)methyl]ethane-1,2-diamine **10a** (0.515 g, 3.0 mmol) in MeOH (5 mL) was added to a suspension of diene **13** (0.407 g, 1.0 mmol, a 1: 1 mixture of isomers ) in MeOH (10 mL) at –10 °C and stirred for 1 h at the same temperature. The precipitated bisthiodiene **15** was filtered off, washed with water and cold MeOH (2 × 3 mL), and dried under reduced pressure to yield diene **15**. The collected filtrates were carefully neutralized by means of hydrochloric acid and stirred with additional 50 mL of water. Again, the solid was filtered off, and then washed with water and diethyl ether (3 × 5 mL). Recrystallization from methanol gave imidazolidine **14.**

*Imidazolidine***14**. A 1: 1 mixture of isomers was obtained. Yield 0.189 g (44%), white solid, m.p. 172–173 °C. IR (KBr) ν_max_ = 3312, 3055, 2917, 1588, 1420, 1137, 824 cm^−1^. ^1^H NMR (200 MHz, DMSO-d_6_) δ = 3.67–3.90 (4H, m, CH_2_ imi), 4.46-4.60 (2H, m, NCH_2_-pyr), 7.48–7.60 (1H, m, H pyr), 7.71–7.81 (1H, m, H pyr), 8.24–8.39 (1H, m, H pyr), 9.41 (1H, s, NH) ppm; ^13^C NMR (50 MHz, DMSO-d_6_) δ = 42.7 (CH_2_ imi), 49.4 (NCH_2_-pyr), 50.8 (CH_2_ imi), 103.4, 105.2 (CNO_2_), 113.0, 113.8 (CClBr), 124.3, 124.4 (CH), 126.6, 128.7, 131.4, 131.5, 137.9, 138.0 (CH), 147.9, 148.0, 149.6, 159.6, 159.7 (NCN) ppm; MS *m/z* (*I*_rel_, %): 426 [M^+^] (1), 380 [M-NO_2_]^+^ (1), 347 [M-Br]^+^ (20), 314 [M-pyr]^+^ (3), 312 [M-Br-Cl]^+^ (3), 254 [M-C_2_Cl_2_Br+H]^+^ (35), 126 [Cl-pyr-CH_2_]^+^ (100); HRMS (ESI^+^) *m/z* calcd for C_12_H_9_N_4_O_2_Cl_3_Br [M-H]^−^: 424.8980; found: 424.8967.

*Bisthiodiene***15**. A 1: 1 mixture of isomers was obtained. Yield 0.150 g (30%), yellow solid, m.p. 141–142 °C. IR (ATR) ν_max_ = 1589, 1489, 1293, 1230, 823, 505 cm^−1^. ^1^H NMR (400 MHz, CDCl_3_) δ = 6.84–7.01 (6H, m, H Ph), 7.05–7.17 (2H, m, H Ph) ppm; ^13^C NMR (101 MHz, CDCl_3_) δ = 115.1, 117.2 (CClBr), 116.3–116.7 (CH Ph), 123.4, 125.5, 126.1 (C_Ph_-S), 133.4, 133.5, 136.5, 136.6, 136.7 (C Ph), 138.3, 138.4 (CNO_2_), 159.1, 159.5 (CSS), 163.1 (CF, *J*_C,F_ = 251.5 Hz), 163.1 (CF, *J*_C,F_ = 252.3 Hz) ppm; MS, *m/z* (*I*_rel_, %): 497 [M^+^] (2), 462 [M − Cl]^+^ (2), 418 [M − Br]^+^ (30), 383 [M − Br-Cl]^+^ (3), 372 [M − Br-NO_2_]^+^ (4), 127 [S-Ph-F]^+^ (100); HRMS (ESI^+^) *m/z* calcd for C_16_H_9_NO_2_BrCl_2_F_2_S_2_ [M + H]^+^: 497.8598; found 497.8588.

*Alternative synthesis of diene***15***:* To a solution of bromonitrodiene **2** (0.316 g, 1.0 mmol, a 47: 53 mixture of isomers) and 4-fluorobenzenethiol (0.256 g, 2.0 mmol) in MeOH (10 mL) at 0 °C, a solution of sodium methanolate (0.108 g, 2.0 mmol) in MeOH (5 mL) was added. The solution was stirred for 3 h at 0 °C and at r.t. for 1 d. Subsequently, the solution was concentrated and the precipitate filtered off and washed with diluted HCl (5 mL) and cold MeOH (2 × 2 mL). A mixture of both isomers was obtained. Yield of bisthiodiene **15** is 0.414 g (83%, a 1:1 mixture of isomers).

*N,N’-Bis[(2-chloro-1,3-thiazol-5-yl)methyl]ethane-1,2-diamine* (**16**) was prepared according to the literature [13] in 70% yield.

*Synthesis of 5,5’-{[2-(3-bromo-2,3-dichloro-1-nitroprop-2-en-1-ylidene)imidazolidine-1,3-diyl] dimethanediyl}-bis(2-chloro-1,3-thiazole)* (**17**). To a solution of diene **2** (0.166 g, 0.5 mmol) in a mixture of MeOH and water (10 mL, 10: 1) at 0 °C *N*,*N*’-bis[(2-chloro-1,3-thiazol-5-yl)methyl]ethane-1,2-diamine (**16**) (0.170 g, 0.5 mmol) and Na_2_CO_3_ (0.112 g, 2.0 mmol) were added carefully. After 1 h at 0 °C, the mixture was allowed to reach r.t. and stirred for another 5 h. Subsequently, the mixture was concentrated in vacuo and the resulting precipitate was filtered off and washed with cold MeOH (2 × 5 mL), water (2 × 5 mL) and again MeOH (1 × 5 mL). A 1: 1 mixture of isomers was obtained. Yield 0.252 g (89%), white solid, mp 162–163 °C. IR (ATR) ν_max_ = 1569, 1533, 1325, 1143, 1046, 770 cm^−1^. ^1^H NMR (400 MHz, DMSO-d_6_) δ = 3.73–3.96 (4H, m, CH_2_), 4.58–4.80 (4H, m, imi-CH_2_), 7.69 (2H, s, CH) ppm; ^13^C NMR (100 MHz, DMSO-d_6_) δ = 45.2, 45.4 (CH_2_ imi), 45.2, 45.4 (CH_2_ imi), 47.2, 47.3 (imi-CH_2_), 98.1, 99.7 (CNO_2_), 109.7, 110.6 (CClBr), 127.0, 128.8 (CCl), 133.6, 133.7 (SC thiaz), 141.8, 141.9 (NC thiaz), 151.7, 151.8 (NCS), 161.6, 161.8 (NCN) ppm; MS *m/z* (*I*_rel_, %): 563 [M^+^] (1), 527 [M − Cl]^+^ (1), 484 [M − Br]^+^ (1), 132 [thiazole-Cl-CH_2_]^+^ (100); HRMS (ESI^+^) *m/z* calcd for C_14_H_11_N_5_O_2_Cl_4_BrS_2_ [M + H]^+^: 563.8286; found: 563.8284.

*Ethyl [(1,3,4,4-tetrachloro-2-nitrobuta-1,3-dien-1-yl)sulfanyl]acetate* (**18**) was prepared according to the literature [18] in 80% yield.

*Synthesis of 3-[4-(propan-2-yl)phenyl]-2-(2,3,3-trichloro-1-nitroprop-2-en-1-ylidene)-1,3-thiazolidin-4-one* (**19a**) *(General method).* To a suspension of the acetate **18** (0.355 g, 1.0 mmol) in MeOH (3 mL) at −18 °C a solution of 4-(propan-2-yl)aniline (0.297 g, 2.2 mmol) in MeOH (3 mL) was added dropwise within 10 min. The mixture was stirred for 3 h at −18 °C and 12 h at r.t. before it was concentrated. The resulting precipitate was filtered off and washed with cold MeOH (1 × 5 mL), water (2 × 5 mL) and again MeOH (2 × 5 mL). The product was dried in vacuo. Yield 0.298 g (73%), beige solid, m.p. 208–209 °C. IR (ATR) ν_max_ = 2962, 1758, 1520, 1289, 1167, 686 cm^−1^. ^1^H NMR (400 MHz, DMSO-d_6_) δ = 1.22 (3H, d, *J* = 6.9 Hz, *i*-Pr), 1.23 (3H, d, *J* = 6.9 Hz, *i*-Pr), 2.95 (1H, sep, *J* = 6.8 Hz, *i*-Pr), 4.13 (1H, d, *J* = 18.7 Hz, SCH_2_), 4.17 (1H, d, *J* = 18.7 Hz, SCH_2_), 7.28–7.33 (2H, m, H Ar), 7.34–7.40 (2H, m, H Ar) ppm; ^13^C NMR (100 MHz, DMSO-d_6_) δ = 23.6, 23.8 (CH_3_), 32.5 (CH_2_), 33.6 (CH *i*-Pr), 121.3 (CCl_2_), 121.4 (CNO_2_), 126.9 (CH), 127.3 (CH), 127.4 (CH), 128.4, 128.8 (CH), 132.1, 150.6, 165.8 (NCS), 174.1 (C=O) ppm; MS *m/z* (*I*_rel_, %): 406 [M^+^] (10), 389 [M-OH]^+^ (5), 371 [M-Cl]^+^ (5), 278 [M-C_2_Cl_3_+H]^+^ (15), 261 [M-C_2_Cl_3_-O]^+^ (100); MS (ESI^−^) *m/z* calcd for C_15_H_12_N_2_O_3_Cl_3_S 405.0; found: 405.0.

*2-(2,3,3-Trichloro-1-nitroprop-2-en-1-ylidene)-3-[4-(trifluoromethyl)phenyl]-1,3-thiazolidin-4-one* (**19b**). Same procedure as for **19a**, using 4-(trifluoromethyl)aniline (0.355 g, 2.2 mmol) at 0 °C for 3 h and 15 h at r.t.. Yield 0.330 g (76%), yellowish solid, m.p. 228–229 °C. IR (ATR) ν_max_ = 3058, 1737, 1515, 1285, 1170, 691 cm^−1^. ^1^H NMR (400 MHz, DMSO-d_6_) δ = 4.13 (1H, d, *J* = 18.7 Hz, SCH_2_), 4.20 (1H, d, *J* = 18.7 Hz, SCH_2_), 7.67–7.74 (2H, m, H Ar), 7.91–7.98 (2H, m, H Ar) ppm; ^13^C NMR (400 MHz, DMSO-d_6_) δ = 32.8 (CH_2_), 121.2 (CNO_2_), 121.3 (CCl_2_), 123.9 (*J*_C,F_ = 272.4 Hz, CF_3_), 126.2 (*J*_C,F_ = 3.9 Hz, CH), 126.6 (*J*_C,F_ = 4.2 Hz, CH), 128.9 (CH), 129.1, 130.2 (CH), 130.7 (*J*_C,F_ = 32.3 Hz, CF_3_-C), 138.1, 165.4 (NCS), 173.9 (C=O) ppm; MS *m/z* (*I*_rel_, %): 432 [M^+^] (3); 397 [M − Cl]^+^ (12); 304 [M-C_2_Cl_3_+H]^+^ (30); 287 [M-C_2_Cl_3_-O]^+^ (93); 145 (100); HRMS (ESI^−^) *m/z* calcd for C_13_H_5_N_2_O_3_Cl_3_SF_3_ [M − H]^−^: 430.9044; found: 430.9062.

*Synthesis of 3-(hydrazinylidenemethyl)-4-nitro-N-[4-(propan-2-yl)phenyl]-1H-pyrazol-5-amine* (**20a**). To a stirred suspension of thiazolidinone **19a** (0.408 g, 1.0 mmol) in MeOH (10 mL) at −18 °C, a solution of hydrazine hydrate (0.400 g, 8.0 mmol) in MeOH (5 mL) was added dropwise within 5 min. Subsequently, the mixture was allowed to reach 0 °C. After 5 h, and 18 h at r.t., the solution was concentrated and neutralized to pH 7 with HCl (5%), then extracted with ethyl acetate (3 × 10 mL). The combined organic phases were purified over a short column chromatography using ethyl acetate-petroleum ether (1:1). Subsequently, the obtained solution was concentrated and the resulting precipitate was filtered and washed with diethyl ether (2 × 3 mL). Yield 0.150 g (52%), red solid, m.p. 194–195 °C. IR (ATR) ν_max_ = 3365, 2956, 1597, 1562, 1461, 1350, 579 cm^−1^. ^1^H NMR (400 MHz, DMSO-d_6_) δ = 1.18 (6H, d, *J* = 6.9 Hz, CH_3_ *i*-Pr), 2.83 (1H, sep, *J* = 6.8 Hz, CH *i*-Pr), 7.16 (2H, d, *J* = 8.5 Hz, H Ar), 7.61 (2H, d, *J* = 8.4 Hz, H Ar), 8.07 (1H, s, NCH), 8.15 (2H, s, N-NH_2_), 8.53 (1H, s, NH anil), 13.29 (1H, s, NH pyr) ppm; ^13^C NMR (100 MHz, DMSO-d_6_) δ =24.2 (CH_3_), 32.9 (CH *i*-Pr), 117.4 (CNO_2_), 118.0 (2 × CH Ph), 122.7 (NCH), 126.7 (2 × CH Ph), 138.3, 138.7, 141.4, 147.4 (NNHC) ppm; MS *m/z* (*I*_rel_, %): 288 [M^+^] (60), 273 [M − CH_3_]^+^ (100), 256 [M-CH_3_-OH]^+^ (5), 245 [M-*i*-Pr]^+^ (8), 169 [M-NH-Ph-*i*-Pr]^+^ (5); HRMS (ESI^−^) *m/z* calcd for C_13_H_15_N_6_O_2_ [M − H]^−^: 287.1261; found: 287.1272.

*Synthesis of 3-(hydrazinylidenemethyl)-4-nitro-N-[4-(trifluoromethyl)phenyl]-1H-pyrazol-5-amine* (**20b**). To a stirred suspension of thiazolidinone **19b** (0.434 g, 1.0 mmol) in MeOH (10 mL) at −18 °C, a solution of hydrazine hydrate (0.250 g, 5.0 mmol) in MeOH (5 mL) was added dropwise within 5 min. Subsequently, the mixture was allowed to reach 0 °C. After 5 h with stirring at 0 °C and 18 h at r.t., the solution was concentrated and the resulting precipitate filtered off. Washing the product with cold MeOH (2 × 5 mL) and drying in vacuo yielded the pyrazole **20b**. Yield 0.145 g (46%), yellow solid, m.p. 248–250 °C. IR (ATR) ν_max_ = 3440, 1602, 1516, 1327, 1160, 832 cm^−1^. ^1^H NMR (400 MHz, DMSO-d_6_) δ = 7.63 (2H, d, *J* = 8.7 Hz, H Ph), 7.91 (2H, d, *J* = 8.6 Hz, H Ph), 8.07 (1H, s, NCH), 8.21 (2H, s, NH_2_), 8.97 (1H, s, Ph-NH), 13.45 (1H, s, NH Pyr) ppm; ^13^C NMR (100 MHz, DMSO-d_6_) δ = 117.6 (2 × CH Ph), 117.8 (CNO_2_), 121.0 (*J*_C,F_ = 32.1 Hz, CCF_3_), 122.2 (*J*_C,F_ = 270.7 Hz, CF_3_), 122.5 (NCH), 126.2 (*J*_C,F_ = 3.7 Hz, 2 × CH Ph), 139.0, 144.2, 146.4 (NNHC) ppm;. MS *m/z* (*I*_rel_, %): 314 [M^+^] (2), 270 [M-CH_2_=N-NH_2_]^+^ (3), 254 [M-CH_2_=N-NH_2_-O]^+^ (14), 161 [CF_3_-Ph-NH_2_]^+^ (60), 142 [CF_2_-Ph-NH_2_]^+^ (27), 96 (100); HRMS (ESI^−^) *m/z* calcd for C_11_H_8_N_6_O_2_F_3_ [M − H]^−^: 313.0665; found: 313.0666.

*Synthesis of 5-(furan-2-ylmethylidene)-3-[4-(propan-2-yl)phenyl]-2-(2,3,3-trichloro-1-nitroprop-2-en-1- ylidene)-1,3-thiazolidin-4-one* (**21a**) *(General method)*. To a suspension of thiazolidinone **19a** (0.408 g, 1.0 mmol) in acetic acid (15 mL) furan-2-carbaldehyde (0.115 g, 1.2 mmol) and triethylamine (0.152 g, 1.5 mmol) were added. The resulting mixture was refluxed for 4 h. After concentration of this mixture in vacuo to a volume of about 3 mL and cooling to r.t., the resulting precipitate was filtered off and washed with water (2 × 10 mL) and cold MeOH (1 × 2 mL). The product was dried in vacuo. Yield 0.466 g (96%), orange solid, m.p. 228–229 °C. IR (ATR) ν_max_ = 2958, 1718, 1598, 1519, 1167, 764 cm^−1^. ^1^H NMR (400 MHz, DMSO-d_6_) δ = 1.24 (3H, d, *J* = 6.9 Hz, CH_3_ *i*-Pr), 1.25 (3H, d, *J* = 6.9 Hz, CH_3_ *i*-Pr), 2.97 (1H, sep, *J* = 6.8 Hz, CH *i*-Pr), 6.86 (1H, dd, *J* = 1.8 Hz, *J* = 3.6 Hz, H fur), 7.34 (1H, d, *J* = 3.5 Hz, H fur), 7.37–7.45 (4H, m, H Ar), 7.90 (1H, s, =CH), 8.30 (1H, d, *J* = 1.7 Hz, H fur) ppm; ^13^C NMR (100 MHz, DMSO-d_6_) δ = 23.6 (CH_3_), 23.9 (CH_3_), 33.6 (CH *i*-Pr), 114.4 (CH), 116.7, 120.8 (CNO_2_), 121.0 (CCl_2_), 121.8 (CH), 122.8 (CH), 126.9 (CH), 127.3 (CH), 127.4 (CH), 128.8 (CH), 128.8 (CCl-CCl_2_), 132.1, 149.4 (CH), 149.9, 150.8, 158.5 (NCS), 166.5 (C=O) ppm; MS *m/z* (*I*_rel_, %): 484 [M^+^] (6), 449 [M-Cl]^+^ (2), 339 [M-C_2_Cl_3_-O]^+^ (75), 325 [M-furan-CH=C=S-Cl]^+^ (7), 124 [furan-CH=C=S]^+^ (100). HRMS (ESI^+^) *m/z* calcd for C_20_H_15_N_2_O_4_SCl_3_Na [M + Na]^+^: 506.9716; found: 506.9712.

*3-[4-(Propan-2-yl)phenyl]-5-(thiophen-2-ylmethylidene)-2-(2,3,3-trichloro-1-nitroprop-2-en-1-ylidene) -1,3-thiazolidin-4-one* (**21b**). Same procedure as for **21a**, using thiophene-2-carbaldehyde (0.135 g, 1.2 mmol). After filtration of the product, it was further purified by column chromatography using chloroform. Yield 0.462 g (92%), dark yellow solid, m.p. 221–223 °C. IR (ATR) ν_max_ = 2963, 1717, 1586, 1525, 1258, 729 cm^−1^. ^1^H NMR (400 MHz, CDCl_3_) δ = 1.29 (6H, d, *J* = 6.7 Hz, CH_3_ *i*-Pr), 2.99 (1H, sep, *J* = 6.9 Hz, CH *i*-Pr), 7.16–7.20 (1H, m, H Ar), 7.24–7.27 (1H, m, H thien), 7.27–7.30 (1H, m, H Ar), 7.35–7.39 (2H, m, H Ar), 7.56 (1H, d, *J* = 3.7 Hz, H thien), 7.78 (1H, d, *J* = 5.0 Hz, H thien), 8.12 (1H, s, =CH) ppm; ^13^C NMR (100 MHz, CDCl_3_) δ = 23.5 (CH_3_), 23.7 (CH_3_), 34.1 (CH *i*-Pr), 117.6, 120.6 (CCl_2_), 122.6 (CNO_2_), 126.8 (CH), 127.4 (CH), 127.8 (CH), 127.9 (CH), 129.1 (CH), 129.6 (CH), 129.7 (CClCCl_2_), 131.6, 133.6 (CH), 134.8 (CH), 137.6, 151.4 (*i*-Pr-C), 155.3 (NCS), 166.7 (C=O) ppm; MS *m/z* (*I*_rel_, %): 500 [M^+^] (3), 465 [M-Cl]^+^ (2), 372 [M-C_2_Cl_3_+H]^+^ (4), 355 [M-C_2_Cl_3_-O]^+^ (73), 140 [thienyl-C=CS]^+^ (100). HRMS (ESI^+^) *m/z* calcd for C_20_H_15_N_2_O_3_Cl_3_S_2_Na [M + Na]^+^: 522.9487; found: 522.9482.

*5-[(1-Methyl-1H-pyrrol-2-yl)methylidene]-3-[4-(propan-2-yl)phenyl]-2-(2,3,3-trichloro-1-nitroprop-2 -en-1-ylidene)-1,3-thiazolidin-4-one* (**21c**). Same procedure as for **21a**, using 1-methyl-1H-pyrrole-2-carbaldehyde (0.328 g, 3.0 mmol) and triethylamine (0.304 g, 3.0 mmol). The mixture was refluxed for 6 h. Yield 0.459 g (92%), red solid, m.p. 244–246 °C. IR (ATR) ν_max_ = 2986, 1700, 1584, 1520, 1273, 745 cm^−1^. ^1^H NMR (400 MHz, DMSO-d_6_) δ = 1.24 (3H, d, J = 6.9 Hz, CH_3_ i-Pr), 1.25 (3H, d, J = 6.9 Hz, CH_3_ i-Pr), 2.98 (1H, sep, J = 6.9 Hz, CH i-Pr), 3.83 (3H, s, NCH_3_), 6.47 (1H, dd, J = 2.6 Hz, J = 4.2 Hz, H pyrr), 6.88 (1H, dd, J = 1.1 Hz, J = 4.2 Hz, H pyrr), 7.36–7.44 (5H, m, 4H Ph, H pyrr), 7.84 (1H, s, =CH) ppm; ^13^C NMR (100 MHz, DMSO-d_6_) δ = 23.6 (CH_3_), 23.9 (CH_3_ i-Pr), 33.6 (NCH_3_), 34.3 (CH i-Pr), 111.5 (CCl_2_), 111.9 (CH), 118.0 (CH), 120.2 (CNO_2_), 121.2, 124.9 (CH), 126.9 (CH), 127.4 (2 × CH), 128.0, 128.6, 128.7 (CH), 131.8 (CH), 132.2, 150.7 (i-Pr-C), 157.5 (NCS), 166.5 (C=O) ppm; MS *m/z* (I_rel_, %): 497 [M^+^] (10), 462 [M-Cl]^+^ (1), 361 [M-pyrrol-CH=C=S+H]^+^ (3), 352 [M-C_2_Cl_3_-CH_4_]^+^ (25), 137 [Me-pyrrol-CH=C=S]^+^ (100); HRMS (ESI^+^) *m/z* calcd for C_21_H_18_N_3_O_3_Cl_3_SNa [M + Na]^+^: 520.0032; found: 520.0018.

*5-(Furan-2-ylmethylidene)-2-(2,3,3-trichloro-1-nitroprop-2-en-1-ylidene)-3-[4-(trifluoromethyl) phenyl]-1,3-thiazolidin-4-one* (**21d**). Same procedure as for **21a**, using thiazolidinone **21b** (0.433 g, 1.0 mmol), furan-2-carbaldehyde (0.115 g, 1.2 mmol) and triethylamine (0.152 g, 1.5 mmol). Yield 0.374 g (73%), orange solid, m.p. 227–229 °C. IR (ATR) ν_max_ = 3130, 1720, 1525, 1286, 1162, 622 cm^−1^. ^1^H NMR (400 MHz, CDCl_3_) δ = 6.67 (1H, dd, J = 1.8 Hz, J = 3.6 Hz, H fur), 6.98 (1H, d, J = 3.6 Hz, H fur), 7.41–7.45 (1H, m, H Ar), 7.50–7.55 (1H, m, H Ar), 7.72 (1H, s, =CH), 7.78–7.84 (2H, m, H Ar), 7.85 (1H, d, J = 1.8 Hz, H fur) ppm; ^13^C NMR (100 MHz, CDCl_3_) δ = 113.9 (CH), 116.9, 120.4 (CH), 120.6 (CCl_2_), 123.4 (J_C,F_ = 272.8 Hz, CF_3_), 122.5 (CNO_2_), 122.9 (CH), 126.4 (J_C,F_ = 3.7 Hz, CH), 126.9 (J_C,F_ = 3.7 Hz, CH), 127.8 (CH), 128.8 (CH), 130.2 (CClCCl_2_), 132.6 (J_C,F_ = 33.0 Hz, CF_3_C), 137.2, 148.0 (CH), 149.9, 156.0, 166.4 (C=O)ppm; MS *m/z* (I_rel_, %): 510 [M^+^] (3), 475 [M-Cl]^+^ (1), 429 [M-Cl-NO_2_]^+^ (1), 382 [M-C_2_Cl_3_+H]^+^ (5), 365 [M-C_2_Cl_3_-O]^+^ (37), 145 [Ph-CF_3_]^+^ (24), 124 [furan-CH=C=S]^+^ (100); HRMS (ESI^+^) *m/z* calcd for C_18_H_8_N_2_O_4_Cl_3_SF_3_Na [M + Na]^+^: 532.9120; found: 532.9117.

*5-(Thiophen-2-ylmethylidene)-2-(2,3,3-trichloro-1-nitroprop-2-en-1-ylidene)-3-[4-(trifluoromethyl)- phenyl]-1,3-thiazolidin-4-one* (**21e**). Same procedure as for **21a**, using thiazolidinone **21b** (0.433 g, 1.0 mmol), thiophene-2-carbaldehyde (0.135 g, 1.2 mmol) and triethylamine (0.152 g, 1.5 mmol). After filtration, the product was purified by column chromatography using chloroform. The fraction containing the product was dried, suspended in MeOH (2 mL), filtered, and the solid washed with cold MeOH (1 × 2 mL). Yield 0.449 g (85%), orange solid, m.p. 245–246 °C. IR (ATR) ν_max_ = 3076, 1699, 1522, 1281, 1126, 730 cm^−1^. ^1^H NMR (400 MHz, CDCl_3_) δ = 7.28 (1H, dd, J = 3.8 Hz, J = 5.0 Hz, H thien), 7.43–7.45 (1H, m, H Ar), 7.52–7.54 (1H, m, H Ar), 7.58 (1H, d, J = 3.7 Hz, H thien), 7.79–7.84 (2H, m, H Ar), 7.84–7.86 (1H, m, H thien), 8.15 (1H, s, =CH) ppm; ^13^C NMR (100 MHz, CDCl_3_) δ = 116.9, 120.4 (CCl_2_), 123.3 (J_C,F_ = 271.9 Hz, CF_3_), 126.4 (J_C,F_ = 3.9 Hz, CH), 126.9 (J_C,F_ = 4.2 Hz, CH), 127.8 (CH), 128.8 (CH), 129.3 (CH), 130.3 (CH), 130.4 (CClCCl_2_), 132.3 (J_C,F_ = 32.0 Hz, CF_3_-C), 134.1 (CH), 135.3 (CH), 137.2, 137.4, 154.5 (NCS), 166.2 (C=O) ppm, CNO_2_ could not be detected; MS *m/z* (I_rel_, %): 526 [M^+^] (4), 491 [M-Cl]^+^ (1), 398 [M-C_2_Cl_3_+H]^+^ (5), 381 [M-C_2_Cl_3_-O]^+^ (48), 140 [thienyl-C=CS]^+^ (100); HRMS (ESI^+^) *m/z* calcd for C_18_H_8_N_2_O_3_Cl_3_S_2_F_3_Na [M + Na]^+^: 548.8892; found, *m/z*: 548.8888.

*5-[(1-Methyl-1H-pyrrol-2-yl)methylidene]-2-(2,3,3-trichloro-1-nitroprop-2-en-1-ylidene)-3- [4-(tri fluoro-methyl)-phenyl]-1,3-thiazolidin-4-one* (**21f**). Same procedure as for **21a**, using thiazolidinone **21b** (0.433 g, 1.0 mmol), 1-methyl-1H-pyrrole-2-carbaldehyde (0.262 g, 2.4 mmol) and triethylamine (0.152 g, 1.5 mmol). The mixture was refluxed for 8 h. Yield 0.289 g (55%), red solid, shows fluorescence, m.p. 241–242 °C. IR (ATR) ν_max_ = 1688, 1589, 1521, 1269, 1064, 620 cm^−1^. ^1^H NMR (400 MHz, DMSO-d_6_) δ = 3.84 (3H, s, NCH_3_), 6.48 (1H, dd, J = 2.3 Hz, J = 4.1 Hz, H pyrr), 6.90 (1H, dd, J = 1.3 Hz, J = 4.1 Hz, H pyrr), 7.39 (1H, dd, J = 1.3 Hz, J = 2.3 Hz, H pyrr), 7.78–7.86 (2H, m, H Ar), 7.87 (1H, s, =CH), 7.94–8.00 (2H, m , H Ph) ppm; ^13^C NMR (100 MHz, DMSO-d_6_) δ = 34.3 (NCH_3_), 111.3, 112.1 (CH), 118.2 (CH), 120.0 (CNO_2_), 121.2 (CCl_2_), 123.9 (J_C,F_ = 273.1 Hz, CF_3_), 125.1 (CH), 126.2 (CH), 126.7 (CH), 128.0, 129.0 (CH), 129.2, 130.1 (CH), 130.8 (J_C,F_ = 32.3 Hz, CCF_3_), 132.0 (CH), 138.2, 157.2 (NCS), 166.2 (C=O) ppm; MS *m/z* (I_rel_, %): 523 [M^+^] (5), 458 [M-F-NO_2_]^+^ (2), 378 [M-Ph-CF_3_]^+^ (12), 287 (100), 145 [Ph-CF_3_]^+^ (90); HRMS (ESI^+^) *m/z* calcd for C_19_H_11_N_3_O_3_Cl_3_SF_3_Na [M + Na]^+^: 545.9436; found: 545.9433.

*1-Methyl-4-nitro-3-(morpholin-4-yl)-1H-pyrazole-5-carbaldehyde* (**22**) was prepared according to the literature [26] in 48% yield.

*Synthesis of 5-{[3-(Morpholin-4-yl)-4-nitro-1H-pyrazol-5-yl]methylidene}-3-[4-(propan-2-yl)phenyl]-2-(2,3,3-trichloro-1-nitropropylidene)-1,3-thiazolidin-4-one* (**23a**) *(General method)*. To a suspension of thiazolidinone **19a** (0.408 g, 1.0 mmol) in acetic acid (10 mL), a solution of 1-methyl-3-(morpholin-4-yl)-4-nitro-1*H*-pyrazole-5-carbaldehyde (0.240 g, 1.0 mmol) in acetic acid (5 mL) and triethylamine (0.152 g, 1.5 mmol) was added. Subsequently, the mixture was refluxed for 4 h. After cooling, the solvent was removed and MeOH (5 mL) added. The resulting precipitate was filtered off and washed with cold MeOH (2 × 5 mL). The product was dried in vacuo. Yield 0.592 g (94%), orange solid, mixture of two isomers (100: 16), m.p. 212–213 °C. IR (ATR) ν_max_ = 2961, 1735, 1535, 1290, 1178, 683 cm^−1^. ^1^H NMR major isomer (400 MHz, DMSO-d_6_) δ = 1.21 (6H, d, *J* = 6.9 Hz, CH_3_ *i*-Pr), 2.99 (1H, sep, *J* = 6.8 Hz, CH *i*-Pr), 3.22–3.28 (4H, m, NCH_2_), 3.72–3.78 (4H, m, OCH_2_), 3.85 (3H, s, NCH_3_), 7.33–7.53 (4H, m, H Ar), 7.90 (1H, s, =CH) ppm; ^13^C NMR major isomer (100 MHz, DMSO-d_6_) δ = 23.5, 23.8 (CH_3_ *i*-Pr), 33.6 (CH *i*-Pr), 39.0 (NCH_3_), 49.9 (NCH_2_), 65.9 (OCH_2_), 120.1, 121.4 (CH), 121.8, 123.5 (CNO_2_), 127.0 (CH), 127.4 (2 × CH), 128.9 (CH), 129.5, 130.9, 131.7, 137.4, 151.1 (C-*i*-Pr), 153.2 (N-N-C-N), 156.5 (NCS), 165.1 (C=O) ppm; MS *m/z* (*I*_rel_, %): 628 [M^+^] (55), 613 [M-CH_3_]^+^ (83), 598 [M-2(CH_3_)]^+^ (22), 582 [M-NO_2_]^+^ (20), 499 [M-C_2_Cl_3_]^+^ (10), 146 (100); HRMS (ESI^+^) *m/z* calcd for C_24_H_23_N_6_O_6_Cl_3_SNa [M + Na]^+^: 651.0363; found: 651.0360.

*5-{[3-(Morpholin-4-yl)-4-nitro-1H-pyrazol-5-yl]methylidene}-2-(2,3,3-trichloro-1-nitropropylidene) -3-[4-(trifluoromethyl)phenyl]-1,3-thiazolidin-4-one* (**23b**). Same procedure as for **23a**, using thiazolidinone **19b** (0.434 g, 1.0 mmol), 1-methyl-3-(morpholin-4-yl)-4-nitro-1*H*-pyrazole-5-carbaldehyde (0.240 g, 1.0 mmol) and triethylamine (0.152 g, 1.5 mmol). The mixture was refluxed for 2 h. Yield 0.636 g (97%), mixture of two isomers (100: 17), orange solid, m.p. 226–227 °C. IR (ATR) ν_max_ = 1733, 1538, 1291, 1171, 1065, 630 cm^−1^. ^1^H NMR major isomer (400 MHz, DMSO-d_6_) δ = 3.22–3.29 (4H, m, NCH_2_), 3.72–3.79 (4H, m, OCH_2_), 3.85 (3H, s, NCH_3_), 7.81–8.04 (4H, m, 4H Ar), 7.93 (1H, s, =CH) ppm; ^13^C NMR major isomer (100 MHz, DMSO-d_6_) δ = 39.1 (NCH_3_), 49.9 (NCH_2_), 65.9 (OCH_2_), 120.1, 121.6 (pyr-CH), 121.7 (CH), 123.9 (J_C,F_ = 272.7 Hz, CF_3_), 123.6 (CNO_2_), 126.3 (J_C,F_ = 3.9 Hz, CH), 126.8 (J_C,F_ = 3.9 Hz, CH), 129.0 (CH), 130.2, 130.3 (CH), 130.8, 131.2 (J_C,F_ = 32.1 Hz, CCF_3_), 137.3, 137.7, 153.2, 156.2 (NCS), 164.9 (C=O) ppm; MS *m/z* (I_rel_, %): 654 [M^+^] (35), 639 [M − CH_3_]^+^ (60), 619 [M − Cl]^+^ (8), 145 [Ph-CF_3_]^+^ (50), 127 (100); HRMS (ESI^+^) *m/z* calcd for C_22_H_16_N_6_O_6_Cl_3_SF_3_Na [M + Na]^+^: 676.9767; found: 676.9769.

*1,1’-(3,4,4-Trichloro-2-nitrobuta-1,3-diene-1,1-diyl)bis(1H-benzotriazole)* (**24**) was prepared according to the literature [28] in 76% yield.

*Synthesis of N-[1-(1H-Benzotriazol-1-yl)-3,4,4-trichloro-2-nitrobuta-1,3-dien-1-yl]-2-methoxyaniline* (**25a**) *(General method).* To a suspension of azole **24** (0.437 g, 1.0 mmol) in MeOH (10 mL) at 0 °C, 2-methoxyaniline (0.129 g, 1.05 mmol) was slowly added. The mixture was allowed to reach r.t. and stirred for another 3 h. After evaporation of the solvent, HCl (10%, 10 mL) was added and the resulting sludge was stirred for 20 min. The precipitate was then filtered off and washed with HCl (10%, 5 mL), cold water (5 mL) and cold Et_2_O (2 × 5 mL). The product was dried in vacuo. Yield 0.392 g (89%), yellow solid, m.p. 128–129 °C. IR (KBr) ν_max_ = 1621, 1580, 1463, 1252, 1023, 753 cm^−1^. ^1^H NMR (200 MHz, CDCl_3_) δ = 3.91 (3H, s, OCH_3_), 6.15 (1H, dd, *J* = 1.3 Hz, *J* = 8.0 Hz, H Ar), 6.46 (1H, ddd *J* = 7.7 Hz, *J* = 1.2 Hz, *J* = 7.5 Hz, H Ar), 6.82 (1H, dd, *J* = 1.5 Hz, *J* = 8.3 Hz, H Ar), 7.00 (1H, ddd, *J* = 8.3 Hz, *J* = 1.4 Hz, *J* = 7.5 Hz, H Ar), 7.27–7.42 (3H, m, H Bzt), 8.02–8.06 (1H, m, H Bzt), 11.65 (1H, s, NH) ppm; ^13^C NMR (50 MHz, CDCl_3_) δ = 55.9 (OCH_3_), 109.8 (CH Bzt), 111.3 (CH Ar), 120.6 (CH Ar), 120.9 (CH Bzt), 122.2 (CH Ar), 123.8 (CNH), 125.3 (CH Bzt), 128.6 (CH Ar), 129.5 (CH Bzt), 131.9, 145.3 (NCN), 145.9, 151.4 (COCH_3_) ppm, three C signals from butadiene-chain due to their low intensity could not be detected; MS *m/z* (*I*_rel_, %): 439 [M^+^] (4), 403 [M-HCl]^+^ (3), 358 [M-Cl-NO_2_]^+^ (3), 331 [M-Ph-OCH_3_]^+^ (6), 122 [H_2_NPhOCH_3_]^+^ (100); HRMS (ESI^+^) *m/z* calcd for C_17_H_13_N_5_O_3_Cl_3_ [M + H]^+^: 440.0079; found: 440.0077.

*N-[1-(1H-Benzotriazol-1-yl)-3,4,4-trichloro-2-nitrobuta-1,3-dien-1-yl]-2-ethoxyaniline* (**25b**). Same procedure as for **25a**, using 2-ethoxyaniline (0.144 g, 1.05 mmol). Yield 0.355 g (78%), orange solid, m.p. 145–146 °C. IR (ATR) ν_max_ = 1620, 1583, 1471, 1241, 1147, 741 cm^−1^. ^1^H NMR (400 MHz, CDCl_3_) δ = 1.56 (3H, t, *J* = 7.0 Hz, OCH_2_CH_3_), 4.15 (2H, q, *J* = 6.7 Hz, OCH_2_CH_3_), 5.99 (1H, dd, *J* = 1.0 Hz, *J* = 8.0 Hz, H Ar), 6.41 (1H, ddd, *J* = 7.6 Hz, *J* = 1.0 Hz, *J* = 7.9 Hz, H Ar), 6.82 (1H, dd, *J* = 8.3 Hz, *J* = 0.9 Hz, H Ar), 6.97 (1H, ddd, *J* = 8.0 Hz, *J* = 1.2 Hz, *J* = 7.8 Hz, H Ar), 7.31 (1H, d, *J* = 8.0 Hz, H Bzt), 7.37 (ddd, *J* = 7.0 Hz, *J* = 1.1 Hz, *J* = 8.1 Hz, H Bzt), 7.43 (1H, t, *J* = 7.36 Hz, H Bzt), 8.06 (1H, d, *J* = 8.2 Hz, H Bzt) ppm; ^13^C NMR (100 MHz, CDCl_3_) δ = 14.7 (CH_3_), 64.7 (OCH_2_), 109.8 (CH), 112.2 (CH), 120.6 (CH), 120.7 (CH), 121.5 (CH), 124.0, 125.3 (CH), 128.3 (CH), 129.5 (CH), 131.9, 145.3, 145.6, 150.6 ppm, three C signals from butadiene-chain due to their low intensity could not be detected; MS *m/z* (*I*_rel_, %): 453 [M^+^] (1), 417 [M − HCl]^+^ (1), 390 [M-Cl-N_2_]^+^ (1), 135 [NPhOEt_2_]^+^ (38), 100 (100); HRMS (ESI^+^) *m/z* calcd for C_18_H_15_N_5_O_3_Cl_3_ [M + H]^+^: 454.0235; found: 454.0234.

*N’-[1-(1H-Benzotriazol-1-yl)-3,4,4-trichloro-2-nitrobuta-1,3-dien-1-yl]-N,N-dimethylbenzene-1,4-di- amine* (**25c**). Same procedure as for **25a**, using *N*,*N*-dimethylbenzene-1,4-diamine (0.143 g, 1.05 mmol) keeping the reaction mixture at 0 °C until completion. Yield 0.413 g (91%), dark red solid, m.p. 147–148 °C. IR (ATR) ν_max_ = 2891, 1620, 1491, 1358, 1168, 818 cm^−1^. ^1^H NMR (400 MHz, CDCl_3_) δ = 2.81 (6H, s, NCH_3_), 6.31 (2H, d, *J* = 9.0 Hz, H Ar), 6.60 (2H, d, *J* = 9.0 Hz, H Ar), 7.35–7.44 (3H, m, H Bzt), 8.04 (1H, d, *J* = 8.4 Hz, H Bzt), 11.88 (1H, s, NH) ppm; ^13^C NMR (100 MHz, CDCl_3_) δ = 40.1 (CH_3_), 109.8 (CH), 112.2 (CH), 117.0, 120.6 (CH), 120.9, 123.8 (CH), 125.2 (CH), 128.0, 129.3 (CH), 129.5, 145.3, 146.8, 149.4 ppm, three C signals from butadiene-chain due to their low intensity could not be detected; MS *m/z* (*I*_rel_, %): 452 [M^+^] (2), 416 [M − Cl]^+^ (1), 388 [M-HCl-N_2_]^+^ (1), 119 [Bzt + H]^+^ (100); HRMS (ESI^+^) *m/z* calcd for C_18_H_16_N_6_O_2_Cl_3_ [M + H]^+^: 453.0395; found: 453.0395.

*1-[3,4,4-Trichloro-1-(2,3-dihydro-1H-indol-1-yl)-2-nitrobuta-1,3-dien-1-yl]-1H-benzotriazole* (**25f**). Same procedure as for **25a**, using 1*H*-indoline (0.125 g, 1.05 mmol). Yield 0.354 g (87%), yellow solid, m.p. 178–179 °C. IR (ATR) ν_max_ = 3065, 1533, 1295, 1023, 905, 655 cm^−1^. ^1^H NMR (400 MHz, CDCl_3_) δ = 3.34–3.46 (2H, m, CH_2_ ind), 3.89–4.25 (2H, m, CH_2_ ind), 5.06–6.38 (1H, s, H ind), 6.66–7.00 (1H, m, H Bzt), 7.06 (1H, s, H ind), 7.29–7.36 (1H, m, H ind), 7.40–7.88 (3H, m, 2H Bzt, 1H ind), 8.16 (1H, d, *J* = 8.1 Hz, H Bzt) ppm; ^13^C NMR (100 MHz, CDCl_3_) δ = 29.2 (CH_2_), 54.5 (CH_2_), 113.9 (CNO_2_), 121.1 (2 × CH), 123.2, 125.9 (2 × CH), 126.0 (CH), 127.9 (CH), 128.0 (CH), 130.0 (CH), 131.8 (CCl_2_), 141.2 (NCN), 146.1, 146.2 ppm; MS *m/z* (*I*_rel_, %): 435 [M^+^] (12), 289 [M-indolin-N_2_]^+^ (34), 271 [M-Bzt-NO_2_]^+^ (79), 118 [benzotriazole]^+^ (100); HRMS (ESI^+^) *m/z* calcd for C_18_H_13_N_5_O_2_Cl_3_ [M + H]^+^: 436.0129; found: 436.0127.

*Synthesis of 5-Methoxy-8-(2,3,3-trichloro-1-nitroprop-2-en-1-ylidene)-7-azabicyclo[4.2.0]octa-1,3,5- triene* (**26a**) *(General method).* A suspension of azole **25a** (0.441 g, 1.0 mmol) in MeOH (10 mL) was stirred at reflux for 5 h. Subsequently, the mixture was concentrated and cooled to 0 °C. The precipitate was filtered off and washed with diethyl ether (2 × 5 mL). Yield 0.119 g (37%), yellow solid, m.p. 168–169 °C. IR (KBr) ν_max_ = 3112, 1637, 1536, 1395, 1083, 859 cm^−1^. ^1^H NMR (200 MHz, DMSO-d_6_) δ = 3.96 (3H, s, OCH_3_), 7.30–7.42 (2H, m, CH), 7.74 (1H, dd, *J* = 2.3 Hz, *J* = 7.6 Hz, CH), 12.40 (1H, s, NH) ppm; ^13^C NMR (50 MHz, DMSO-d_6_) δ = 57.0 (OCH_3_), 110.9 (CH), 114.3 (CH), 119.5, 123.9 (CH), 124.0, 125.9, 130.6, 134.0, 147.3, 153.9 (COCH_3_) ppm; MS *m/z* (*I*_rel_, %): 320 [M^+^] (1), 284 [M − HCl]^+^ (12), 256 [M-NO_2_-NH_2_+H]^+^ (40), 64 (100); HRMS (ESI^+^) *m/z* calcd for C_11_H_8_N_2_O_3_Cl_3_ [M + H]^+^: 320.9595; found: 320.9594.

*5-Ethoxy-8-(2,3,3-trichloro-1-nitroprop-2-en-1-ylidene)-7-azabicyclo[4.2.0]octa-1,3,5-triene* (**26b**). Same procedure as for **26a**, using azole **25b** (0.455, 1.0 mmol) and refluxing in EtOH for 10 h. Yield 0.091 g (27%), yellow solid, m.p. 204–205 °C. IR (KBr) ν_max_ = 2992, 1647, 1533, 1378, 1234, 554 cm^−1^. ^1^H NMR (400 MHz, DMSO-d_6_) δ = 1.42 (3H, t, *J* = 7.0 Hz, OCH_2_CH_3_), 4.16–4.26 (2H, m, OCH_2_CH_3_), 7.30–7.38 (2H, m, CH), 7.71 (1H, dd, *J* = 1.5 Hz, *J* = 8.2 Hz, CH), 12.37 (1H, s, NH) ppm; ^13^C NMR (100 MHz, DMSO-d_6_) δ = 14.5 (CH_3_), 65.4 (OCH_2_), 110.7 (CH), 114.9 (CH), 119.5, 123.9 (CH), 124.0, 125.9, 130.6, 133.9, 146.5, 153.9 ppm; MS *m/z* (*I*_rel_, %): 334 [M^+^] (3), 299 [M − Cl]^+^ (52), 271 [M-Cl-EtO+H]^+^ (100); HRMS (ESI^+^) *m/z* calcd for C_12_H_10_N_2_O_3_Cl_3_ [M + H]^+^: 334.9752; found: 334.9750.

*Synthesis of N,N-Dimethyl-8-(2,3,3-trichloro-1-nitroprop-2-en-1-ylidene)-7-azabicyclo[4.2.0]octa-1,3,5- trien-3-amine* (**26c**). A suspension of azole **25c** (0.454 g, 1.0 mmol) in THF (20 mL) was stirred at reflux for 10 h. After evaporation of the solvent, dilute HCl (10 mL) was added and the mixture stirred for 15 min. Subsequently, the mixture was extracted with chloroform and purified by column chromatography using petroleum ether - ethyl acetate (1:1). Yield 0.084 g (25%), red solid, m.p. 171–173 °C. IR (KBr) ν_max_ = 2856, 1567, 1369, 1063, 846, 550 cm^−1^. ^1^H NMR (400 MHz, DMSO-d_6_) δ = 2.97 (6H, s, N(CH_3_)_2_), 7.22 (1H, d, *J* = 2.0 Hz, CH), 7.32 (1H, d, *J* = 9.0 Hz, CH), 7.36 (1H, dd, *J* = 2.1 Hz, *J* = 9.2 Hz, CH), 12.65 (1H, s, NH) ppm; ^13^C NMR (100 MHz, DMSO-d_6_) δ = 40.3 (Me), 98.6 (CH), 117.8 (CH), 119.9, 121.1 (CH), 124.0, 125.6, 130.9, 133.5, 147.5 (CNMe_2_), 153.1 ppm; MS *m/z* (*I*_rel_, %): 333 [M^+^] (27), 298 [M − Cl]^+^ (40), 270 [M-NO_2_-NH_3_]^+^ (100); HRMS (ESI^+^) *m/z* calcd for C_12_H_11_N_3_O_2_Cl_3_ [M + H]^+^: 333.9917; found: 333.9919.

*Synthesis of N’-[6-(Dichloromethyl)-2-methyl-5-nitropyrimidin-4-yl]-N, N-dimethylbenzene-1,4- diamine* (**27c**). To a solution of the nitrobutadiene **25c** (0.454 g, 1.0 mmol) and acetamidine hydrochloride (0.284 g, 3.0 mmol) in 20 mL dry THF, sodium hydride (0.160 g, 4.0 mmol, 60%) was added at 0 °C. The solution was thoroughly stirred for 1 h at 0 °C and then at r.t. for 2 d. After evaporation of the solvent and addition of 10% HCl (5 mL) the precipitate was filtered off and washed with 10% HCl (2 × 10 mL), cold water (10 mL), cold MeOH (2 × 5 mL) and dried in vacuo. Yield 0.232 g (65%), yellow solid, m.p. 124–125 °C. IR (KBr) ν_max_ = 3321, 1612, 1573 (NO_2_), 1517, 1204, 746 cm^−1^. ^1^H NMR (400 MHz, CDCl_3_) δ = 2.64 (3H, s, CCH_3_), 2.99 (6H, s, NCH_3_), 6.74 (2H, d, *J* = 9.0 Hz, H Ar), 7.40 (2H, d, *J* = 8.9 Hz, H Ar), 7.42 (1H, s, CHCl_2_), 9.73 (1H, s, NH) ppm; ^13^C NMR (100 MHz, CDCl_3_) δ = 26.5 (CH_3_), 40.5 (NCH_3_), 66.8 (CHCl_2_), 112.3 (CH), 122.8, 124.5 (CH), 125.3, 148.9, 153.2, 160.6, 171.0 ppm; MS *m/z* (*I*_rel_, %): 355 [M^+^] (39), 136 [phenylendiamine+H]^+^ (100); HRMS (ESI^+^) *m/z* calcd for C_14_H_16_N_5_O_2_Cl_2_ [M + H]^+^: 356.0676; found: 356.0674.

*6-(Dichloromethyl)-N,N,2-trimethyl-5-nitropyrimidin-4-amine* (**27d**). Same procedure as for **27c**, using diene **25d** (0.363 g, 1.0 mmol). The precipitate was further purified by column chromatography using petroleum ether - ethyl acetate, 5: 1. Yield 0.148 g (56%), yellowish solid, m.p. 82–83 °C. IR (ATR) ν_max_ = 3048, 1579 (NO_2_), 1500, 1338 (NO_2_), 1195, 746 cm^−1^. ^1^H NMR (400 MHz, CDCl_3_) δ = 2.61 (3H, s, CCH_3_), 3.09 (6H, s, NCH_3_), 6.98 (1H, s, CHCl_2_); ^13^C NMR (100 MHz, CDCl_3_) δ = 26.1 (CH_3_), 39.3 (NCH_3_), 65.4 (CHCl_2_), 125.6 (CNO_2_), 154.7, 156.2, 168.1 ppm; MS *m/z* (*I*_rel_, %): 264 [M^+^] (55), 218 [M − NO_2_]^+^ (30), 182 [M-CHCl_2_+H]^+^ (63), 135 [M-CHCl_2_-NO_2_]^+^ (52). HRMS (ESI^+^) *m/z* calcd for C_8_H_11_N_4_O_2_Cl_2_ [M + H]^+^: 265.0254; found: 265.0255.

*N-[6-(Dichloromethyl)-2-methyl-5-nitropyrimidin-4-yl]quinolin-8-amine* (**27e**). Same procedure as for **27c**, using azole **25e** (0.462 g, 1.0 mmol) as starting material. Yield 0.178 g (49%), yellow solid, m.p. 217–219 °C. IR (KBr) ν_max_ = 3262, 1573 (NO_2_), 1298 (NO_2_), 1208, 766, 590 cm^−1^. ^1^H NMR (400 MHz, CDCl_3_) δ = 2.82 (3H, s, CCH_3_), 7.37 (1H, s, CHCl_2_), 7.52 (1H, dd, *J* = 8.3 Hz, *J* = 4.2 Hz, H quin), 7.58–7.63 (2H, m, H quin), 8.21 (1H, dd, *J* = 8.3 Hz, *J* = 1.6 Hz, H quin), 8.94 (1H, dd, *J* = 4.2 Hz, *J* = 1.6 Hz, H quin), 9.04–9.07 (1H, m, H quin), 12.17 (1H, s, NH) ppm; ^13^C NMR (100 MHz, CDCl_3_) δ = 26.6 (CH_3_), 66.4 (CHCl_2_), 118.4 (CH), 122.0 (CH), 122.93 (CH), 124.6, 127.0 (CH), 128.1, 134.0, 136.4 (CH), 139.3, 149.0 (CH), 152.1, 159.7, 170.9 ppm; MS *m/z* (*I*_rel_, %): 363 [M^+^] (15), 317 [M-NO_2_]^+^ (100), 281 [M − CHCl_2_]^+^ (25), 235 [M − quin]^+^ (12), 128 [quinoline]^+^ (45); HRMS (ESI^+^) *m/z* calcd for C_15_H_12_N_5_O_2_Cl_2_ [M + H]^+^: 364.0368; found: 364.0368.

*1-[6-(Dichloromethyl)-2-methyl-5-nitropyrimidin-4-yl]-2,3-dihydro-1H-indole* (**27f**). Same procedure as for **27c**, using diene **25f** (0.437 g, 1.0 mmol), DMSO (25 mL) and NaOH (30%, 4 mmol). Yield 0.216 g (64%), yellow solid, m.p. 132–133 °C. IR (ATR) ν_max_ = 3048, 1562 (NO_2_), 1529, 1340 (NO_2_), 1209, 768 cm^−1^. ^1^H NMR (400 MHz, CDCl_3_) δ = 2.72 (3H, s, CCH_3_), 3.20 (2H, t, *J* = 7.9 Hz, H ind), 3.86 (2H, t, *J* = 7.9 Hz, H ind), 7.04 (1H, s, CHCl_2_), 7.11 (1H, t, *J* = 7.4 Hz, H ind), 7.24–7.29 (2H, m, H ind), 7.93 (1H, d, *J* = 8.1 Hz, H ind) ppm; ^13^C NMR (100 MHz, CDCl_3_) δ = 26.0 (CH), 28.8 (CH_2_), 50.2 (CH_2_), 65.3 (CHCl_2_), 117.6 (CH), 124.8 (CH), 125.1 (CH), 126.4, 127.1 (CH), 132.3, 142.1, 151.6, 156.8, 168.7 ppm; MS *m/z* (*I*_rel_, %): 338 [M^+^] (100), 323 [M-CH_3_]^+^ (13), 257 [M-NO_2_-Cl] (48), 118 [indole]^+^ (24); HRMS (ESI^+^) *m/z* calcd for C_14_H_13_N_4_O_2_Cl_2_ [M + H]^+^: 339.0416; found: 339.0413.

*Synthesis of 1-[6-(Dichloromethyl)-2-methyl-5-nitropyrimidin-4-yl]-1H-indole* (**28**). A solution of pyrimidine **27f** (0.400 g, 1.2 mmol) and DDQ (0.600 g, 2.7 mmol) in toluene (10 mL) was heated to reflux for 5 h, allowed to cool down to r.t., and the precipitate filtered off. The filtrate was purified by column chromatography using petroleum ether–ethyl acetate, 25: 1. The product was dried in vacuo. Yield 0.267 g (66%), m.p. 103–104 °C. IR (ATR) ν_max_ = 3022, 1589 (NO_2_), 1342, 1261 (NO_2_), 1099, 745 cm^−1^. ^1^H NMR (600 MHz, CDCl_3_) δ = 2.92 (3H, s, CCH_3_), 6.79 (1H, dd, *J* = 3.7 Hz, J = 0.7 Hz, H ind), 7.01 (1H, s, CHCl_2_), 7.12 (1H, d, *J* = 3.7 Hz, H ind), 7.30 (1H, t, *J* = 8.0 Hz, H ind), 7.36 (1H, t, *J* = 8.4 Hz, H ind), 7.63 (1H, d, *J* = 8.2 Hz, H ind), 8.10 (1H, d, *J* = 9.1 Hz, H ind) ppm; ^13^C NMR (150 MHz, CDCl_3_) δ = 26.2 (CH_3_), 64.8 (CHCl_2_), 110.9 (CH), 114.1 (CH), 121.6 (CH), 123.8 (CH), 124.5 (CH), 124.9 (CH), 128.3, 130.4, 135.4, 150.5, 157.6, 170.5 ppm; MS *m/z* (*I*_rel_, %): 336 [M^+^] (100), 291 [M + H-NO_2_]^+^ (42), 238 [M-CH_3_-CHCl_2_]^+^ (48), 116 [indole]^+^ (62); HRMS (ESI^+^) *m/z* calcd for C_14_H_11_N_4_O_2_Cl_2_ [M + H]^+^: 337.0259; found: 337.0261.

*Synthesis of 1-[1-(1H-Benzotriazol-1-yl)-3,4,4-trichloro-2-nitrobuta-1,3-dien-1-yl]-4-(4-chlorophenyl)*- *piperidin-4-ol* (**25g**). Same procedure as for **25a**, using 4-(4-chlorophenyl)piperidin-4-ol (0.223 g, 1.05 mmol). Yield 0.482 g (91%), yellowish solid, m.p. 149–150 °C. IR (ATR) ν_max_ = 2931, 1565, 1498, 1290, 1005, 749 cm^−1^. ^1^H NMR (400 MHz, CDCl_3_) δ = 1.83–2.03 (2H, m, H pip), 2.33–2.61 (2H, m, H pip), 2.95–3.25 (2H, m, NCH_2_), 3.76–4.08 (3H, m, OH + NCH_2_), 7.35–7.38 (2H, m, H Ar), 7.45–7.47 (2H, m, H Ar), 7.47–7.79 (3H, m, H Bzt), 8.10–8.13 (1H, m, H Bzt) ppm; ^13^C NMR (100 MHz, CDCl_3_) δ = 38.3 (CH_2_), 47.3 (CH_2_), 70.4 (COH), 110.4 (CH), 111.89 (CNO_2_), 120.9 (CH), 124.8 (CCl), 125.9 (2 × CH), 126.0 (CH), 126.2 (CCl_2_), 128.8 (2 × CH), 130.6 (CH), 132.4, 133.6 (CCl), 144.8, 146.1, 148.0 (CNN) ppm; MS *m/z* (*I*_rel_, %): 527 [M^+^] (1), 499 [M-N_2_]^+^ (1), 453 [M-N_2_-NO_2_]^+^ (1), 416 [M-Ph-Cl]^+^ (1), 111 [PhCl]^+^ (20), 91 (100); HRMS (ESI^+^) *m/z* calcd for C_21_H_18_N_5_O_3_Cl_4_ [M + H]^+^: 538.0158; found: 528.0156.

*{4-[1-(1H-Benzotriazol-1-yl)-3,4,4-trichloro-2-nitrobuta-1,3-dien-1-yl]piperazin-1-yl}(tetrahydrofuran-2-yl)-methanone* (**25h**). Same procedure as for **25a**, using piperazin-1-yl(tetrahydrofuran-2-yl)methanone (0.193 g, 1.05 mmol). Yield 0.426 g (85%), yellow solid, m.p. 170–172 °C. IR (KBr) ν_max_ = 2871, 1657, 1560, 1290, 1006, 747 cm^−1^. ^1^H NMR (200 MHz, CDCl_3_) δ = 1.80–2.11 (3H, m, CH_2_ fur), 2.30–2.49 (1H, m, CH_2_ fur), 3.18–3.60 (4H, m, NCH_2_), 3.72–4.41 (6H, m, 2NCH_2_, OCH_2_), 4.52–4.67 (1H, m, OCH), 7.48–7.80 (3H, m, H Bzt), 8.17–8.20 (1H, m, H Bzt) ppm; ^13^C NMR (50 MHz, CDCl_3_) δ 25.7 (CH_2_), 27.7 (CH_2_), 41.8 (NCH_2_), 45.2 (NCH_2_), 50.6 (NCH_2_), 69.2 (OCH_2_), 76.1 (OCH), 110.2 (CH), 116.2 (CCl_2_), 120.6 (CNO_2_), 121.1 (CH), 125.5, 126.1 (CH), 129.9, 130.7 (CH), 146.3, 147.5 (CNN), 169.7 (C=O) ppm; MS *m/z* (*I*_rel_, %): 500 [M^+^] (1), 465 [M-Cl]^+^ (1), 426 [M-NO_2_-N_2_]^+^ (1), 337 [M-Bzt-NO_2_+H]^+^ (4), 119 [Bzt + H]^+^ (16), 92 (100); HRMS (ESI^+^) *m/z* calcd for C_19_H_20_N_6_O_4_Cl_3_ [M + H]^+^: 501.0606; found: 501.0605.

*1-{3,4,4-Trichloro-1-[4-(3-chlorophenyl)piperazin-1-yl]-2-nitrobuta-1,3-dien-1-yl}-1H-benzotriazole* (**25i**). Same procedure as for **25a**, using 1-(3-methylphenyl)piperazine (0.207 g, 1.05 mmol). Yield 0.452 g (88%), yellow solid, m.p. 68–70 °C. IR (KBr) ν_max_ = 2916, 1594, 1560, 1291, 942, 768 cm^−1^. ^1^H NMR (200 MHz, CDCl_3_) δ = 3.41–3.61 (8H, m, NCH_2_), 6.75–6.90 (1H, m, H Ar), 6.91–6.98 (2H, m, H Ar), 7.18–7.24 (1H, m, H Ar), 7.40–7.68 (3H, m, H Bzt), 8.16 (1H, d, *J* = 8.1 Hz, H Bzt) ppm; ^13^C NMR (50 MHz, CDCl_3_) δ = 49.2 (NCH_2_), 49.4 (NCH_2_), 110.3 (CH), 115.1 (CH), 117.2 (CH), 121.1 (CH), 121.6 (CH), 124.3 (CCl_2_), 125.3 (CCl), 126.1 (CH), 130.4 (CH), 130.7 (CH), 132.4, 135.2 (CCl), 146.3, 147.6 (CNN), 150.7 ppm, CNO_2_ could not be detected; MS *m/z* (*I*_rel_, %): 512 [M^+^] (2), 449 [M-Cl-N_2_]^+^ (1), 401 [M-PhCl]^+^ (2), 138 [PhClNCH]^+^ (100); HRMS (ESI^+^) *m/z* calcd for C_20_H_17_N_6_O_2_Cl_4_ [M + H]^+^: 512.0078; found: 512.0075.

*Synthesis of 4-(4-Chlorophenyl)-1-[5-(dichloromethyl)-1-methyl-4-nitro-1H-pyrazol-3-yl]piperidin-4-ol* (**29a**) *(General method).* To a suspension of azole **25g** (0.529 g, 1.0 mmol) in MeOH (10 mL) at −10 °C, methylhydrazine (0.921 g, 2.0 mmol) was added dropwise. After 2 h, the solution was allowed reach r.t. and stirred for 1 d. The solution was then concentrated and 10% HCl (5 mL) was added. The resulting precipitate was filtered off, washed with water, and purified by column chromatography using chloroform. Yield 0.311 g (74%), yellow solid, m.p. 95–96 °C. IR (ATR) ν_max_ = 2951, 1553, 1484, 1348, 1024, 746 cm^−1^. ^1^H NMR (400 MHz, CDCl_3_) δ = 1.65 (1H, s, OH), 1.78–1.85 (2H, m, CCH_2_), 2.28 (2H, ddd, *J* = 13.1 Hz, *J* = 4.2 Hz, *J* = 13.1 Hz, CCH_2_), 3.33 (2H, ddd, *J* = 12.5 Hz, *J* = 2.5 Hz, *J* = 12.5 Hz, NCH_2_), 3.51–3.59 (2H, m, NCH_2_), 4.12 (3H, s, NCH_3_), 7.34 (2H, d, *J* = 8.7 Hz, H Ar), 7.47 (2H, d, *J* = 8.7 Hz, H Ar), 7.88 (1H, s, CHCl_2_) ppm; ^13^C NMR (100 MHz, CDCl_3_) δ = 38.0 (2 × CH_2_), 39.7 (NCH_3_), 46.0 (2 × NCH_2_), 57.8 (CHCl_2_), 71.1 (HO-C), 120.4 (CNO_2_), 126.1 (CH), 129.2 (CH), 133.0 (CCl), 137.9, 146.5, 152.8 (NC-pyr) ppm; MS *m/z* (*I*_rel_, %): 418 [M^+^] (3), 401 [M-OH]^+^ (6), 383 [M − Cl]^+^ (5), 367 [M-Cl-O]^+^ (4), 347 [M-Cl-HCl]^+^ (4), 100 (100); HRMS (ESI^+^) *m/z* calcd for C_16_H_18_N_4_O_3_Cl_3_ [M + H]^+^: 419.0439; found: 419.0437.

*{4-[5-(Dichloromethyl)-1-methyl-4-nitro-1H-pyrazol-3-yl]piperazin-1-yl}(tetrahydrofuran-2-yl)methanone* (**29b**). Same procedure as for **29a**, using diene **25h** (0.502 g, 1.0 mmol) and DCM as the eluent. Yield 0.310 g (79%), yellowish oil. IR (ATR) ν_max_ = 2954, 1647, 1551, 1341, 1198, 745 cm^−1^. ^1^H NMR (400 MHz, CDCl_3_) δ = 1.86–2.12 (3H, m, CH_2_ Fur), 2.25–2.35 (1H, m, CH_2_ Fur), 3.17–3.31 (4H, m, NCH_2_), 3.63–3.73 (2H, m, NCH_2_), 3.79–3.98 (4H, m, NCH_2_, OCH_2_), 4.10 (3H, s, NCH_3_), 4.64 (1H, dd, *J* = 5.6 Hz, *J* = 7.5 Hz, COCHO), 7.84 (1H, s, CHCl_2_) ppm; ^13^C NMR (100 MHz, CDCl_3_) δ = 25.7 (CH_2_), 28.4 (CH_2_), 39.7 (NCH_3_), 41.6 (NCH_2_), 45.0 (NCH_2_), 49.6 (NCH_2_), 50.0 (NCH_2_), 57.6 (CH), 69.1 (OCH_2_), 75.9 (OCH), 120.5 (CNO_2_), 138.1, 152.1, 170.1 (C=O) ppm; MS *m/z* (*I*_rel_, %): 391 [M^+^] (100), 374 [M − OH]^+^ (20), 356 [M − Cl]^+^ (65), 320 [M − furan]^+^ (20), 292 [M-CO-furan]^+^ (35); HRMS (ESI^+^) *m/z* calcd for C_14_H_20_N_5_O_4_Cl_2_ [M + H]^+^: 392.0887; found: 392.0889.

*1-(3-Chlorophenyl)-4-[5-(dichloromethyl)-1-methyl-4-nitro-1H-pyrazol-3-yl]piperazine* (**29c**). Same proce-dure as for **29a**, using diene **25i** (0.514 g, 1.0 mmol) and a mixture of petroleum ether and ethyl acetate (5: 1) as the eluent. Yield 0.299 g (74%), yellow solid, m.p. 162–163 °C. IR (ATR) ν_max_ = 2832, 1552, 1475, 1338, 936, 737 cm^−1^. ^1^H NMR (400 MHz, CDCl_3_) δ = 3.31–3.36 (4H, m, NCH_2_), 3.38–3.43 (4H, m, NCH_2_), 4.13 (3H, s, NCH_3_), 6.81–6.86 (2H, m, H Ar), 6.93 (1H, t, *J* = 2.1 Hz, H Ar), 7.18 (1H, t, *J* = 8.0 Hz, H Ar), 7.87 (1H, s, CHCl_2_) ppm; ^13^C NMR (100 MHz, CDCl_3_) δ = 39.7 (CH_3_), 48.5 (NCH_2_), 49.5 (NCH_2_), 57.7 (CHCl_2_), 114.1 (CH), 116.1 (CH), 119.7 (CH), 120.5 (CNO_2_), 130.1 (CH), 135.0 (CCl), 138.0, 152.2 (CN), 152.3 (CN) ppm; MS *m/z* (*I*_rel_, %): 403 [M^+^] (45), 357 [M − NO_2_]^+^ (6), 322 [M-NO_2_-Cl]^+^ (22), 138 [PhClNCH]^+^ (100); HRMS (ESI^+^) *m/z* calcd for C_15_H_17_N_5_O_2_Cl_3_ [M + H]^+^: 404.0442; found: 404.0444.

*Synthesis of 3-[4-(3-Chlorophenyl)piperazin-1-yl]-1-methyl-4-nitro-1H-pyrazole-5-carbaldehyde* (**30**). Pyrazole **29c** (0.405 g, 1.0 mmol) was suspended in 10 mL sulfuric acid (25%) and heated to 95–100 °C for 10 h. After cooling to r.t., the solution was extracted with chloroform (3 × 10 mL) and purified by column chromatography using petroleum ether - ethyl acetate (5: 1). The product was dried in vacuo. Yield 0.199 g (57%), yellow solid, m.p. 159–160 °C. IR (ATR) ν_max_ = 2827, 1683, 1546, 1474, 1236, 775 cm^−1^. ^1^H NMR (400 MHz, CDCl_3_) δ = 3.32–3.38 (4H, m, NCH_2_), 3.45–3.5 (4H, m, NCH_2_), 6.81–6.87 (2H, m, H Ph), 6.93 (1H, t, *J* = 2.0 Hz, H Ph), 7.19 (1H, t, *J* = 8.1 Hz, H Ph), 10.43 (1H, s, CHO) ppm; ^13^C NMR (100 MHz, CDCl_3_) δ = 40.9 (NCH_3_), 48.5 (2 × NCH_2_), 49.4 (2 × NCH_2_), 114.2 (CH), 116.1 (CH), 119.8 (CH), 125.7 (CNO_2_), 130.1 (CH), 135.0 (CCl), 135.2 (CCHO), 151.9, 152.1, 181.8 (CHO) ppm; MS *m/z* (*I*_rel_, %): 349 [M^+^] (100), 332 [M − OH]^+^ (5), 314 [M − Cl]^+^ (4), 304 [M-NO_2_+H]^+^ (20), 138 [ClPhNCH]^+^ (75); HRMS (ESI^+^) *m/z* calcd for C_15_H_17_N_5_O_3_Cl [M + H]^+^: 350.1014; found: 350.1016.

*Synthesis of 2-(1H-Benzotriazol-1-yl)-4-(dichloromethylidene)-3-nitro-4H-pyrido[1,2-a]pyrimidine* (**31a**) *(General method).* To a solution of azole **24** (0.437 g, 1.0 mmol) in THF (10 m), 2-aminopyridine (0.282 g, 3.0 mmol) was added at r.t. The solution was stirred for 8 h, then concentrated, and the residue treated with dilute HCl for 1 h, filtered off and washed with water (2 × 10 mL) and cold MeOH (5 mL). The product was dried in vacuo. Yield 0.304 g (81%), red solid, m.p. 158–160 °C. IR (KBr) ν_max_ = 3082, 1628, 1552, 1434, 1307, 1215 cm^−1^. ^1^H NMR (200 MHz, DMSO-d_6_) δ = 7.50–7.64 (2H, m, H Ar), 7.64–7.75 (1H, m, H Bzt), 7.87 (1H, d, *J* = 8.3 Hz, H Ar), 7.90–7.95 (1H, m, H Bzt), 8.21 (1H, d, *J* = 7.8 Hz, H Bzt), 8.22–8.32 (1H, m, H Bzt), 8.99 (1H, dd, *J* = 6.8 Hz, 0.9 Hz, H Ar) ppm; ^13^C NMR (50 MHz, DMSO-d_6_) δ = 102.3 (CCl_2_), 113.0 (CH), 119.2 (CH), 120.0 (CH), 121.3 (CNO_2_), 123.7 (CH), 125.5 (CH), 126.3, 129.6 (CH), 132.3, 138.8 (CH), 143.6 (CH), 145.6, 148.0, 151.3 ppm; MS *m/z* (*I*_rel_, %): *m/z* (%) = 374 [M^+^] (1), 256 [M–benzotriazole]^+^ (14), 219 (18), 119 [benzotriazole] (14), 92 (100); HRMS (ESI^+^) *m/z* calcd for C_15_H_9_N_6_O_2_Cl_2_ [M + H]^+^: 375.0164; found: 375.0164.

*2-(1H-Benzotriazol-1-yl)-7-chloro-4-(dichloromethylidene)-3-nitro-4H-pyrido[1,2-a]pyrimidine* (**31b**). Same procedure as for **31a**, using 2-amino-5-chloropyridine (0.386 g, 3.0 mmol) and heating the reaction mixture to 45 °C. Yield 0.352 g (86%), orange solid, m.p. 182–184 °C. IR (KBr) ν_max_ = 3066, 1505, 1437, 1318, 1238, 1222 cm^−1^. ^1^H NMR (200 MHz, DMSO-d_6_) δ = 7.55 (1H, t, *J* = 7.9 Hz, H Bzt), 7.71 (1H, t, *J* = 8.1 Hz, H Bzt), 7.86 (1H, d, *J* = 9.3 Hz, H pyr), 7.92 (1H, d, *J* = 8.2 Hz, H Bzt), 8.23 (1H, d, *J* = 8.2 Hz, H Bzt), 8.35 (1H, dd, *J* = 9.4 Hz, *J* = 2.3 Hz, H pyr), 9.35 (1H, d, *J* = 2.3 Hz, H pyr) ppm; ^13^C NMR (50 MHz, DMSO-d_6_) δ = 102.8 (CCl_2_), 112.9 (CH), 119.9 (CH), 122.2 (CNO_2_), 124.7 (CCl), 124.8 (CH), 125.6 (CH), 129.6 (CH), 132.3, 136.6 (CH), 143.1 (CH), 145.6, 147.7 (NC), 150.6 ppm; MS *m/z* (*I*_rel_, %): 408 [M^+^] (1), 380 [M − N_2_]^+^ (1), 373 [M − Cl]^+^ (2), 334 [M-N_2_-NO_2_]^+^ (7), 112 (100); HRMS (ESI^+^) *m/z* calcd for C_15_H_8_N_6_O_2_Cl_3_ [M + H]^+^: 408.9769; found: 408.9768.

*Synthesis of 4-(dichloromethylidene)-2-[4-(4-fluorophenyl)piperazin-1-yl]-3-nitro-4H-pyrido[1,2-a]pyrimi-dine* (**32a**) *(General method)*. To a solution of pyrimidine **31a** (0.375 g, 1 mmol) in MeOH (10 mL) 1-(4-fluorophenyl)piperazine (0.216 g, 1.2 mmol) was added and the mixture stirred at 40 °C for 5 h. Subsequently, the mixture was cooled to 0 °C and treated with dilute HCl for 1 h. The precipitate was filtered off and washed with water (2 × 5 mL) and cold MeOH (5 mL). The product was dried in vacuo. Yield 0.423 g (97%), yellow solid, m.p. 126–127 °C. IR (KBr) ν_max_ = 1639, 1551, 1504, 1253, 1145, 933 cm^−1^. ^1^H NMR (200 MHz, DMSO-d_6_) δ = 3.15–3.50 (4H, m, NCH_2_), 3.78–4.31 (4H, m, NCH_2_), 6.78–7.18 (5H, m, H Pyr, 4H Ph-F), 7.31 (1H, d, *J* = 8.6 Hz, H pyr), 7.94 (1H, ddd, *J* = 8.6 Hz, *J* = 1.3 Hz, *J* = 7.3 Hz, H pyr), 8.60 (1H, dd, *J* = 7.2 Hz, *J* = 1.3 Hz, H pyr) ppm; ^13^C NMR (50 MHz, DMSO-d_6_) δ = 49.5 (4 × NCH_2_), 94.9 (CCl_2_), 114.7 (CH), 115.6 (*J*_C,F_ = 21.6 Hz, 2 × CH Ph), 118.1 (*J*_C,F_ = 7.9 Hz, 2 × CH Ph), 120.5 (CNO_2_), 122.7 (CH), 128.2 (CCCl_2_), 137.1 (CH), 142.0 (CH), 147.3 (NC Ph), 151.5, 156.6 (*J*_C,F_ = 235.9 Hz, CF), 156.1 (NCN) ppm; MS *m/z* (*I*_rel_, %): 435 [M^+^] (7), 256 [M-morph-Ph-F]^+^ (10), 179 [Morph-Ph-F]^+^ (15), 95 (100); HRMS (ESI^+^) *m/z* calcd for C_19_H_17_N_5_O_2_Cl_2_F [M + H]^+^: 436.0738; found: 436.0736.

*7-Chloro-4-(dichloromethylidene)-2-[4-(4-fluorophenyl)piperazin-1-yl]-3-nitro-4H-pyrido[1,2-a] pyrimidine* (**32b**). Same procedure as for **32a**, using compound **31b** (0.410 g, 1.0 mmol) but without adding HCl. Yield 0.447 g (95%), yellow solid, m.p. 141–142 °C. IR (KBr) ν_max_ = 1635, 1563, 1493, 1373, 1237, 827 cm^−1^. ^1^H NMR (200 MHz, DMSO-d_6_) δ = 3.14–3.29 (4H, m, NCH_2_), 3.50–4.33 (4H, m, NCH_2_), 6.95–7.14 (4H, m, H Ph), 7.31 (1H, d, *J* = 9.4 Hz, H pyr), 7.99 (1H, dd, *J* = 9.5 Hz, *J* = 2.3 Hz, H pyr); 8.92 (1H, d, *J* = 8.9 Hz, H pyr) ppm; ^13^C NMR (50 MHz, DMSO-d_6_) δ = 49.4 (4 × NCH_2_), 94.7 (CCl_2_), 115.3 (*J*_C,F_ = 21.9 Hz, 2 × FCCH), 117.6 (*J*_C,F_ = 7.7 Hz, 2 × CH Ph), 120.1 (CCl), 121.1 (CNO_2_), 123.9 (CH), 127.6, 134.7 (NCH), 141.8 (CH), 147.3 (*J*_C,F_ = 1.8 Hz, NC Ph), 150.4, 156.2 (*J*_C,F_ = 236.4 Hz, CF), 156.0 ppm; MS *m/z* (*I*_rel_, %): 469 [M^+^] (8), 290 [M-piperazine-Ph-F]^+^ (3), 179 [piperazine-Ph-F]^+^ (20), 112 (100); HRMS (ESI^+^) *m/z* calcd for C_19_H_16_N_5_O_2_Cl_3_F [M + H]^+^: 470.0348; found: 470.0350.

*Synthesis of Ethyl {[4-(dichloromethylidene)-3-nitro-4H-pyrido[1,2-a]pyrimidin-2-yl]sulfanyl}acetate* (**33a**) *(General method).* To a solution of pyrimidine **31a** (0.375 g, 1.0 mmol) in EtOH (10 mL), ethyl 2-mercaptoacetate (0.240 g, 2.0 mmol) and sodium ethanolate (0.204 g, 3.0 mmol) were added. The solution was stirred at r.t. for 3 d. Subsequently, dilute HCl (5 mL) was added under stirring for 30 min. The resulting precipitate was filtered off, washed with water (2 × 5 mL) and cold MeOH (3 mL), and dried in vacuo. Yield 0.365 g (97%).

*Alternative synthesis of pyrimidine***33a***from nitrodiene***18**. To a solution of nitrobutadiene **18** (0.355 g, 1.0 mmol) in MeOH (10 mL) 2-aminopyridine (0.282 g, 3.0 mmol) was added. After stirring at r.t. for 1 d, the solution was concentrated and the resulting precipitate filtered off, washed with water (2 × 3 mL), cold MeOH (3 mL) and dried in vacuo. Yield 0.177 g (47%) of **33a**, orange solid, m.p. 147–148 °C. IR (ATR) ν_max_ = 1731, 1597, 1451, 1202, 1141, 764 cm^−1^. ^1^H NMR (400 MHz, CDCl_3_) δ = 1.27 (3H, t, *J* = 7.1 Hz, OCH_2_CH_3_); 3.85 (2H, s, SCH_2_), 4.20 (2H, q, *J* = 7.1 Hz, OCH_2_), 7.00 (1H, ddd, *J* = 1.3 Hz, *J* = 6.9 Hz, *J* = 6.9 Hz, CH), 7.23 (1H, ddd, *J* = 1.2 Hz, *J* = 0.6 Hz, *J* = 9.0 Hz, CH), 7.72 (1H, ddd, *J* = 1.7 Hz, *J* = 7.1 Hz, *J* = 8.8 Hz, CH), 8.04 (1H, ddd, *J* = 1.6 Hz, *J* = 0.7 Hz, *J* = 6.9 Hz, NCH) ppm; ^13^C NMR (100 MHz, CDCl_3_) δ = 14.2 (CH_3_), 33.7 (SCH_2_), 61.5 (OCH_2_), 114.5 (CH), 117.1 (CCl_2_), 123.0 (CH), 125.1 (CNO_2_), 135.6 (NCH), 140.0 (CH), 149.7 (NCN), 164.1 (SCN), 169.3 (C=O) ppm, NCCHCl_2_ could not be detected; MS *m/z* (*I*_rel_, %): 375 [M^+^] (10), 340 [M-Cl]^+^ (9), 288 [M-CH_2_CO_2_Et]^+^ (25), 209 [M-HNO_2_-SCH_2_CO_2_Et]^+^ (95), 149 (100); HRMS (ESI^+^) *m/z* calcd for C_13_H_11_N_3_O_4_Cl_2_SNa [M + Na]^+^: 397.9745; found: 397.9746.

*Ethyl {[7-chloro-4-(dichloromethylidene)-3-nitro-4H-pyrido[1,2-a]pyrimidin-2-yl]sulfanyl}acetate* (**33b**). Same procedure as for **33a,b**ut starting from compounds **31b** (Yield 0.382 g, 93%), or **18** (Yield 0.226 g, 55%), orange solid, m.p. 171–172 °C. IR (ATR) ν_max_ = 3074, 1727, 1626, 1489, 1235, 741 cm^−1^. ^1^H NMR (200 MHz, CDCl_3_) δ = 1.27 (3H, t, *J* = 7.1 Hz, CH_3_), 3.83 (2H, s, SCH_2_), 4.19 (2H, q, *J* = 7.2 Hz, OCH_2_), 7.17 (1H, dd, *J* = 9.4 Hz, *J* = 0.5 Hz, CH), 7.63 (1H, dd, *J* = 9.5 Hz, *J* = 2.3 Hz, CH), 8.05 (1H, dd, *J* = 2.3 Hz, *J* = 0.6 Hz, NCH) ppm; ^13^C NMR (50 MHz, CDCl_3_) δ = 14.3 (CH_3_), 33.6 (SCH_2_), 61.6 (OCH_2_), 117.5 (CCl_2_), 121.4 (CNO_2_), 122.2 (CCl), 123.2 (CH), 124.4 (NC), 133.0 (CH), 140.5 (CH), 148.2, 163.7 (SC), 169.2 (C=O) ppm; MS *m/z* (*I*_rel_, %): 409 [M^+^] (10), 373 [M-HCl]^+^ (18), 363 [M-NO_2_]^+^ (15), 321 [M-CH_3_CO_2_Et]^+^ (45), 242 (100); HRMS (ESI^+^) *m/z* calcd for C_13_H_10_N_3_O_4_Cl_3_SNa [M + Na]^+^: 431.9355; found: 431.9357.

*Synthesis of N-[1-(benzylsulfanyl)-3,4,4-trichloro-2-nitrobuta-1,3-dien-1-yl]naphthalen-1-amine* (**35a**) *(General method).* To a solution of diene **34a** (0.359 g, 1.0 mmol) in MeOH (10 mL) at −10 °C, naphthalen-1-amine (0.315 g, 2.2 mmol) was added. The solution was kept at −10 °C for 2 h and then allowed to warm up to r.t. for 8 h. Subsequently, the solution was concentrated and the resulting precipitate was filtered off, washed with water (2 × 5 mL) and cold MeOH (5 mL), and dried in vacuo. Yield 0.428 g (92%), green-yellow solid, m.p. 132–133 °C. IR (ATR) ν_max_ = 1536, 1332, 1149, 939, 768, 702 cm^−1^. ^1^H NMR (400 MHz, CDCl_3_) δ = 3.47 (2H, q, *J* = 12.4 Hz, SCH_2_), 6.86 (2H, dd, *J* = 7.7 Hz, *J* = 1.7 Hz, H Ph), 7.14–7.25 (3H, m, H Ar), 7.57 (1H, dd, *J* = 7.8 Hz, *J* = 7.8 Hz, H Ph), 7.59–7.65 (2H, m, H Ph), 7.73 (1H, d, *J* = 7.4 Hz, H Ar), 7.93 (1H, d, *J* = 8.2 Hz, H Ar), 7.94–7.99 (2H, m, H Ar), 13.32 (1H, s, NH) ppm; ^13^C NMR (100 MHz, CDCl_3_) δ = 38.5 (CH_2_), 121.5 (CH), 122.0 (CNO_2_), 122.9 (CH), 123.9 (CCl_2_), 125.4 (CH), 127.2 (CH), 127.8 (CH), 128.0 (CCl), 128.2 (CH), 128.5, 128.6 (CH), 128.7 (2 × CH), 128.7 (CH), 128.9 (2 × CH), 133.4, 134.1, 134.2, 159.7 (NCS) ppm; MS *m/z* (*I*_rel_, %): 464 [M^+^] (1), 447 [M − OH]^+^ (1), 418 [M − NO_2_]^+^ (1), 127 [naphthalene]^+^ (11), 91 [PhCH_2_]^+^ (100); HRMS (ESI^+^) *m/z* calcd for C_21_H_16_N_2_O_2_Cl_3_S [M + H]^+^: 464.9993; found: 464.9991.

*N-{3,4,4-Trichloro-1-[(4-chlorophenyl)sulfanyl]-2-nitrobuta-1,3-dien-1-yl}naphthalen-1-amine* (**35b**). Same procedure as for **33a**, but starting from **34b**. Yield 0.423 g (87%), green-yellow solid, m.p. 185–186 °C. IR (ATR) ν_max_ = 1538, 1472, 1345, 1161, 824, 767 cm^−1^. ^1^H NMR (400 MHz, CDCl_3_) δ = 6.68 (2H, d, *J* = 8.8 Hz, H Ar), 6.71 (2H, d, *J* = 8.8 Hz, H Ar), 7.33–7.43 (3H, m, H Ar), 7.48 (1H, ddd, *J* = 6.8 Hz, *J* = 1.2 Hz, *J* = 8.1 Hz, H Ar), 7.55 (1H, d, *J* = 8.5 Hz, H Ar), 7.71–7.80 (2H, m, H Ar), 11.96 (1H, s, NH) ppm; ^13^C NMR (100 MHz, CDCl_3_) δ = 121.4, 121.3 (CH), 123.8 (CNO_2_), 124.6 (CH), 124.9 (CH), 125.6, 126.8 (CH), 126.9 (CH), 128.2 (CH), 128.4, 128.5, 128.7 (CH), 129.1 (2 × CH), 133.2, 133.6, 134.3 (2 × CH), 135.4, 160.8 (NCS) ppm; MS *m/z* (*I*_rel_, %): 484 [M^+^] (5), 449 [M-Cl]^+^ (5), 438 [M-NO_2_]^+^ (3), 356 [M − naphthalene]^+^ (2), 143 [naphthylamine]^+^ (60), 127 [naphthalene]^+^ (100); HRMS (ESI^+^) *m/z* calcd for C_20_H_13_N_2_O_2_Cl_4_S [M + H]^+^: 484.9446; found: 484.9443.

*Synthesis of 2-(Benzylsulfanyl)-4-(dichloromethyl)-3-nitrobenzo[h]quinoline* (**36a**) *(General method).* To a solution of diene **35a** (0.466 g, 1.0 mmol) in chloroform (10 mL) at 0 °C, triethylamine (0.202 g, 2.0 mmol) was added. The solution was kept at 0 °C for 2 h and then allowed to warm to r.t. for 3 h. Subsequently, the solution was concentrated and diluted with 5 mL MeOH. The resulting precipitate was filtered off, washed with 10% aq. HCl (2 × 5 mL), water (2 × 5 mL) and cold MeOH (5 mL), and dried in vacuo to give benzoquinoline **36a**. Yield 0.326 g (76%), yellow solid, m.p. 173–174 °C. IR (ATR) ν_max_ = 3033, 1541, 1336, 1198, 828, 711 cm^−1^. ^1^H NMR (400 MHz, CDCl_3_) δ = 4.78 (2H, s, SCH_2_), 7.12 (1H, s, CHCl_2_), 7.27–7.36 (3H, m, H Ph), 7.48–7.52 (2H, m, H Ph), 7.57 (1H, ddd, *J* = 7.2 Hz, *J* = 1.1 Hz, *J* = 7.9 Hz, H Ar), 7.79 (1H, ddd, *J* = 7.3 Hz, *J* = 1.4 Hz, *J* = 7.3 Hz, H Ar), 7.95 (2H, d, *J* = 9.0 Hz, H Ar), 8.64 (1H, d, *J* = 9.4 Hz, H Ar), 9.18 (1H, dd, *J* = 7.6 Hz, *J* = 0.8 Hz, H Ar) ppm; ^13^C NMR (100 MHz, CDCl_3_) δ = 35.8 (SCH_2_), 63.4 (CHCl_2_), 119.2, 122.3 (CH), 125.4 (CH), 127.7 (CH), 127.9 (CH), 128.0 (CH), 128.5 (CH), 128.7 (2 × CH), 129.0 (2 × CH), 130.1 (CH), 130.4, 134.2, 134.8, 136.1, 139.4, 147.7, 149.8 ppm; MS *m/z* (*I*_rel_, %): 428 [M^+^] (2), 344 [M-CH_2_Cl_2_]^+^ (2), 91 [PhCH_2_]^+^ (100); HRMS (ESI^+^) *m/z* calcd for C_21_H_14_N_2_O_2_Cl_2_SNa [M + Na]^+^: 451.0051; found: 451.0050.

*2-[(4-Chlorophenyl)sulfanyl]-4-(dichloromethyl)-3-nitrobenzo[h]quinoline* (**36b**). Same procedure as for **36a**, but starting from **35b**. Yield 0.382 g (85%), yellow solid, m.p. 162–163 °C. IR (ATR) ν_max_ = 3073, 1572, 1541, 1388, 1028, 739 cm^−1^. ^1^H NMR (400 MHz, CDCl_3_) δ = 7.19 (1H, s, CHCl_2_), 7.53 (2H, d, *J* = 8.6 Hz, H Ph), 7.58 (1H, ddd, *J* = 7.1 Hz, *J* = 1.3 Hz, *J* = 8.3 Hz, H Ph), 7.63 (2H, d, *J* = 8.6 Hz, H Ph), 7.72 (1H, ddd, *J* = 7.0 Hz, *J* = 1.1 Hz, *J* = 8.1 Hz, H Ph), 7.88 (1H, d, *J* = 7.9 Hz, H Ph), 7.92 (1H, d, *J* = 9.3 Hz, H Ph), 8.32 (1H, d, *J* = 8.3 Hz, H Ph), 8.61 (1H, d, *J* = 9.3 Hz, H Ph) ppm; ^13^C NMR (100 MHz, CDCl_3_) δ = 63.4 (CHCl_2_), 119.8, 122.0 (CH), 125.3 (CH), 127.2, 127.8 (CH), 128.0 (CH), 129.1 (CH), 129.6 (2 × CH), 130.1 (CH), 130.4 (SC Ph), 134.0 (CCl), 135.4, 136.4 (Cl_2_CHC), 137.5 (2 × CH), 138.9 (CNO_2_), 147.8 (NC), 149.8 (NCS) ppm; MS *m/z* (*I*_rel_, %): 448 [M^+^] (28), 413 [M − Cl]^+^ (7), 364 [M-CHCl_2_-NO_2_+H]^+^ (15), 175 (100), 111 [PhCl]^+^ (55); HRMS (ESI^+^) *m/z* calcd for C_20_H_11_N_2_O_2_Cl_3_SNa [M + Na]^+^: 470.9505; found: 470.9504.

*Synthesis of 2-(Benzylsulfinyl)-4-(dichloromethyl)-3-nitrobenzo[h]quinoline* (**37a**) *(General method).* To a solution of quinoline **36a** (0.429 g, 1.0 mmol) in chloroform (5 mL) and glacial acetic acid (2 mL) at 0 °C, hydrogen peroxide (1.13 g, 10.0 mmol, 30% aq.) was added dropwise. After 3 h at 0 °C, the solution was allowed to reach r.t. and stirred additionally for 2 d. The solution was then extracted with chloroform (3 × 30 mL), washed with water (2 × 50 mL), dried with CaCl_2_ and dried in vacuo. Column chromatography was carried out with a solvent ratio of 2: 1 (petroleum ether-ethyl acetate). Yield 0.396 g (89%), orange solid, m.p. 138–139 °C. IR (ATR) ν_max_ = 1697, 1533, 1353, 1076, 830, 695 cm^−1^. ^1^H NMR (400 MHz, CDCl_3_) δ = 4.57 (2H, q, *J* = 12.7 Hz, SCH_2_), 7.25–7.31 (6H in all, m, CHCl_2_ and 5H Ph overlapped), 7.81–7.92 (2H, m, H Ar), 8.02 (1H, dd, *J* = 7.5 Hz, *J* = 1.5 Hz, H Ar), 8.15 (1H, d, *J* = 9.4 Hz, H Ar), 8.76 (1H, d, *J* = 9.4 Hz, H Ar), 9.31 (1H, dd, *J* = 7.7 Hz, *J* = 1.0 Hz, H Ar) ppm; ^13^C NMR (100 MHz, CDCl_3_) δ = 62.2 (CH_2_), 62.5 (CHCl_2_), 122.0 (CH), 123.3, 126.2 (CH), 128.0 (CH), 128.6 (CH), 128.7 (CH), 128.8 (2 × CH), 129.5, 130.4 (2 × CH), 130.5, 131.1 (CH), 131.9 (CH), 134.0, 135.9, 138.8 (CNO_2_), 148.3, 153.2 ppm; MS *m/z* (*I*_rel_, %): 444 [M^+^] (10), 428 [M − O]^+^ (5), 360 [M − CH_2_Cl_2_] (5), 96 (100); HRMS (ESI^+^) *m/z* calcd for C_21_H_14_N_2_O_3_Cl_2_SNa [M + Na]^+^: 467.0000; found: 466.9998.

*2-[(4-Chlorophenyl)sulfinyl]-4-(dichloromethyl)-3-nitrobenzo[h]quinoline* (**37b**). Same procedure as for **37a**, but starting from **36b** (0.450 g, 1.0 mmol). Yield 0.424 g (91%), yellow solid, m.p. 166–168 °C. IR (ATR) ν_max_ = 3082, 1546, 1342, 1092, 828, 750 cm^−1^. ^1^H NMR (400 MHz, CDCl_3_) δ = 7.20 (1H, s, CHCl_2_), 7.50 (2H, d, *J* = 8.6 Hz, H Ar), 7.84–7.91 (2H, m, H Ar), 7.95–8.03 (3H, m, H Ar), 8.12 (1H, d, *J* = 9.4 Hz, H Ar), 8.71 (1H, d, *J* = 9.4 Hz, H Ar), 9.28–9.33 (1H, m, H Ar) ppm; ^13^C NMR (100 MHz, CDCl_3_) δ = 62.4 (CHCl_2_), 122.0 (CH), 123.3, 125.8 (CH), 127.0 (2 × CH), 128.2 (CH), 128.8 (CH), 129.7 (2 × CH), 130.5, 131.0 (CH), 132.0 (CH), 134.0, 136.0, 138.0 (CNO_2_), 138.3, 141.1, 148.2, 153.4 (NCS) ppm; MS *m/z* (*I*_rel_, %): 464 [M^+^] (2), 418 [M-NO_2_]^+^ (2), 365 [M-O-CHCl_2_]^+^ (38), 335 [M-CHCl_2_-NO_2_]^+^ (12), 111 [PhCl]^+^ (50), 100 (100); HRMS (ESI^+^) *m/z* calcd for C_20_H_11_N_2_O_3_Cl_3_SNa [M + Na]^+^: 486.9454; found: 486.9454.

*Synthesis of 4-(Dichloromethyl)-3-nitro-2-(pyrrolidin-1-yl)benzo[h]quinoline* (**38**). To a solution of quinoline **37a** (0.445 g, 1.0 mmol) in toluene (10 mL), pyrrolidine (0.107 g, 1.5 mmol) was added and the mixture heated to 100 °C for 3 h. After cooling, the crude product was purified by column chromatography using petroleum ether - ethyl acetate (10: 1). Yield 0.290 g (77%), red solid, m.p. 169–170 °C. IR (ATR) ν_max_ = 2884, 1584, 1506, 1359, 821, 734 cm^−1^. ^1^H NMR (400 MHz, CDCl_3_) δ = 1.99–2.09 (4H, m, NCCH_2_), 3.61–3.71 (4H, m, NCH_2_), 7.07 (1H, s, CHCl_2_), 7.61–7.71 (3H, m, H Ar), 7.85 (1H, dd, *J* = 7.6 Hz, *J* = 1.3 Hz, H Ar), 8.50 (1H, d, *J* = 9.3 Hz, H Ar), 9.03 (1H, d, *J* = 7.9 Hz, H Ar) ppm; ^13^C NMR (100 MHz, CDCl_3_) δ = 25.5 (2 × NCCH_2_), 48.1 (2 × NCH_2_), 64.0 (CHCl_2_), 113.9, 122.6 (CH), 124.1 (CH), 125.5 (CH), 126.6 (CH), 127.6 (CH), 129.2 (CH), 130.2, 130.4 (CNO_2_), 134.3, 135.6, 145.5, 147.5 ppm; MS *m/z* (*I*_rel_, %): 375 [M^+^] (100), 358 [M − OH]^+^ (15), 340 [M − Cl]^+^ (5), 329 [M − NO_2_]^+^ (12), 259 [M-NO_2_-Pyr]^+^ (30); HRMS (ESI^+^) *m/z* calcd for C_18_H_15_N_3_O_2_Cl_2_Na [M + Na]^+^: 398.0439; found: 398.0440.

*Synthesis of 2-ethoxy-2-oxoethyl 4,5-dichloro-1,2-thiazole-3-carboxylate* (**41**). To a solution of isothiazole **40** (0.198 g, 1.0 mmol) and ethyl bromoacetate (0.501 g, 3.0 mmol) in EtOH (10 mL), sodium ethanolate (0.204 g, 3.0 mmol) was added at r.t. The mixture was heated to reflux for 3 d. The product was extracted with chloroform (3 × 10 mL) and purified through a short column chromatography using petroleum ether – ethyl acetate (10:1). Yield 0.165 g (58%), yellowish solid, m.p. 31–33 °C. IR (KBr) ν_max_ = 2983, 1743, 1427, 1193, 1038, 947 cm^−1^. ^1^H NMR (200 MHz, CDCl_3_) δ = 1.31 (3H, t, *J* = 7.1 Hz, OCH_2_CH_3_), 4.28 (2H, q, *J* = 7.1 Hz, OCH_2_CH_3_), 4.91 (2H, s, OCH_2_C=O) ppm; ^13^C NMR (50 MHz, CDCl_3_) δ = 14.0 (CH_3_), 61.7 (OCH_2_CH_3,_ OCH_2_C=O), 126.1 (CCl), 150.8 (SCCl), 153.1 (C=N), 158.2, 166.7; MS *m/z* (*I*_rel_, %): 283 [M^+^] (18), 238 [M − OEt]^+^ (15), 210 [M − CO_2_Et]^+^ (12), 180 [M − OCH_2_CO_2_Et]^+^ (100); HRMS (ESI^+^) *m/z* calcd for C_8_H_7_NO_4_Cl_2_SNa [M + Na]^+^: 305.9371; found: 305.9370.

*Synthesis of 2,2,2-Trifluoroethyl 4,5-dichloro-1,2-thiazole-3-carboxylate* (**43**). A solution of isothiazole **42** (0.217 g, 1.0 mmol) and 2,2,2-trifluoroethanol (0.300 g, 4.0 mmol) in 20 mL dry THF was refluxed for 4 d. After cooling to r.t., the solution was concentrated, diluted with water (20 mL), extracted with hexane (3 × 20 mL), and washed with water (2 × 20 mL). Subsequently, the product was purified by column chromatography (hexane). Yield 0.179 g (64%), white solid, m.p. 51–52 °C. IR (KBr) ν_max_ = cm^−1^: 1741, 1430, 1358, 1167, 1091, 745. ^1^H NMR (200 MHz, CDCl_3_) δ = 4.61 (2H, q, *J* = 8.2 Hz, OCH_2_); ^13^C NMR (50 MHz, CDCl_3_) δ = 61.3 (*J*_C,F_ = 37.1 Hz, CH_2_), 122.6 (*J*_C,F_ = 276.0 Hz, CF_3_), 126.3 (CCl), 151.3 (C=N), 152.3 (SCCl), 157.3 (C=O) ppm; MS *m/z* (*I*_rel_, %): 279 [M^+^] (15), 244 [M − Cl]^+^ (2), 197 [M-CH_2_CF_3_+H]^+^ (12), 180 [M-OCH_2_CF_3_]^+^ (100), 153 [M-CO_2_CH_2_CF_3_+H]^+^ (40); HRMS (ESI^+^) *m/z* calcd for C_6_H_2_NO_2_Cl_2_SF_3_Na [M + Na]^+^: 301.9033; found: 301.9035.

*Synthesis of 4,5-Dichloro-N’-phenyl-1,2-thiazole-3-carbohydrazide* (**44a**) *(General method).* To a suspension of phenylhydrazine (0.324 g, 3.0 mmol) in dry THF (10 mL), the isothiazole **42** (0.216 g, 1.0 mmol) was added at r.t. The mixture was stirred for 1 d. After removal of the solvent, the residue was treated with cold HCl (10%) and the resulting precipitate filtered off. Subsequently, it was washed with cold water (3 × 5 mL) and Et_2_O (2 × 3 mL). The product was dried in vacuo. Yield 0.216 g (75%), light brown solid, m.p. 145–147 °C. IR (KBr) ν_max_ = 3254, 1678, 1605, 1351, 884, 746 cm^−1^. ^1^H NMR (200 MHz, CDCl_3_) δ = 6.88–6.96 (3H, m, H Ph), 7.20–7.26 (3H, m, NH, 2H Ph), 8.81 (1H, s, NH) ppm; ^13^C NMR (50 MHz, CDCl_3_) δ = 113.8 (2 × CH), 121.6 (CH), 125.3 (CCl), 129.2 (2 × CH), 147.3, 150.9 (SCCl), 155.3, 158.9 ppm; MS *m/z* (*I*_rel_, %): 287 [M^+^] (14), 180 [M-CONHNHPh]^+^ (5), 107 [PhNHNH]^+^ (100); HRMS (ESI^+^) *m/z* calcd for C_10_H_7_N_3_OCl_2_SNa [M + Na]^+^: 309.9585; found: 309.9585.

*4,5-Dichloro-N’-[3-(trifluoromethyl)phenyl]-1,2-thiazole-3-carbohydrazide* (**44b**). Same procedure as for **44a**, but using 3-trifluoromethylphenylhydrazine (0.370 g, 2.1 mmol) and stirring the mixture at reflux for 4 h. Yield 0.338 g (95%), yellowish solid, m.p. 152–153 °C. IR (KBr) ν_max_ = 3300, 1691, 1497, 1340, 1161, 798 cm^−1^. ^1^H NMR (200 MHz, DMSO-d_6_) δ = 7.03–7.09 (3H, m, H Ar), 7.34–7.49 (1H, m, H Ar), 8.57 (1H, s, NH), 10.79 (1H, s, NH) ppm; ^13^C NMR (50 MHz, DMSO-d_6_) δ = 108.2 (*J*_C,F_ = 3.9 Hz, CH), 115.2 (*J*_C,F_ = 4.0 Hz, CH), 116.1 (CH), 124.6 (*J*_C,F_ = 271.5 Hz, CF_3_), 123.0 (CCl), 129.7 (*J*_C,F_ = 31.3 Hz, CCF_3_), 130.2 (CH), 149.5 (CCl), 149.9, 157.8, 160.1 (C=O) ppm; MS *m/z* (*I*_rel_, %): 355 [M^+^] (25), 336 [M-F]^+^ (2), 180 [M-CF_3_-Ph-NHNH]^+^ (60), 175 [CF_3_-Ph-NHNH]^+^ (100), 152 [M-CONHNH-Ph-CF_3_]^+^ (10); HRMS (ESI^+^) *m/z* calcd for C_11_H_6_N_3_OCl_2_SF_3_Na [M + Na]^+^: 377.9458; found: 377.9459.

*4,5-Dichloro-N-(4-cyano-2,5-difluorophenyl)-1,2-thiazole-3-carboxamide* (**44c**). Same procedure as for **44a**, but using 4-cyano-2,5-difluoro-aniline (0.354 g, 2.3 mmol) and stirring the mixture at reflux for 6 h. Yield 0.227 g (68%), white solid, m.p. 164–166 °C. IR (KBr) ν_max_ = 3361, 2237 (CN), 1715, 1528, 1190, 673 cm^−1^. ^1^H NMR (200 MHz, CDCl_3_) δ = 7.41 (1H, dd, *J* = 9.8 Hz, *J* = 5.4 Hz, H Ar), 8.57 (1H, dd, *J* = 10.6 Hz, *J* = 6.2 Hz, H Ar), 9.49 (1H, s, NH) ppm; ^13^C NMR (50 MHz, CDCl_3_) δ = 95.6 (*J*_C,F_ = 18.8 Hz, 9.6 Hz, C(CN)), 108.6 (*J*_C,F_ = 28.7 Hz, 1.5 Hz, CH), 112.9 (*J*_C,F_ = 2.3 Hz, C(CN)), 118.3 (*J*_C,F_ = 24.5 Hz, 2.3 Hz, CH), 125.7 (CCl), 131.7 (*J*_C,F_ = 11.9 Hz, NC Ph), 147.5 (*J*_C,F_ = 244.0 Hz, 2.8 Hz, CF), 152.1 (CCl), 154.6, 156.3 (C=O), 160.1 (*J*_C,F_ = 255.3 Hz, 2.3 Hz, CF) ppm; MS *m/z* (*I*_rel_, %): 333 [M^+^] (22), 180 [M-NH-Ph-F_2_-(CN)]^+^ (100), 152 [M-CONH-Ph-F_2_-(CN)]^+^ (15); HRMS (ESI^+^) *m/z* calcd for C_11_H_3_N_3_OCl_2_SF_2_Na [M + Na]^+^: 355.9240; found: 355.9241.

*4,5-Dichloro-N-[2-(1H-indol-3-yl)ethyl]-1,2-thiazole-3-carboxamide* (**44d**). Same procedure as for **44a**, but using tryptamine (0.352 g, 2.2 mmol) and triethylamine (0.223 g, 2.1 mmol). Yield 0.265 g (78%), white solid, m.p. 183–185 °C. IR (KBr) ν_max_ = 3302, 1650, 1537, 1349, 946, 738 cm^−1^. ^1^H NMR (200 MHz, CDCl_3_) δ = 3.08 (2H, t, *J* = 8.1 Hz, Ind-CH_2_), 3.72–3.82 (2H, m, NCH_2_), 7.07 (1H, s, NHC=O), 7.11–7.25 (3H, m, 2H Ar, NCH), 7.37 (1H, d, *J* = 7.7 Hz, H Ar), 7.63 (1H, d, *J* = 7.7 Hz, H Ar), 8.15 (1H, s, NH) ppm; ^13^C NMR (50 MHz, CDCl_3_) δ = 25.3 (CH_2_), 39.6 (CH_2_), 111.2 (CH), 112.6, 118.7 (CH), 119.5 (CH), 122.1 (CH, CCl), 122.2 (CH), 127.2, 136.4, 150.4 (CCl), 156.7, 159.0 (C=O) ppm; MS *m/z* (*I*_rel_, %): 339 [M^+^] (4), 180 [M-NHCH_2_-ind]^+^ (5), 143 [ind-CHCH_2_]^+^ (100), 130 [ind-CH_2_]^+^ (91); HRMS (ESI^+^) *m/z* calcd for C_14_H_11_N_3_OCl_2_SNa [M + Na]^+^: 361.9900; found: 361.9898.

*4,5-Dichloro-N-[(6-chloropyridin-3-yl)methyl]-N-methyl-1,2-thiazole-3-carboxamide* (**44e**). Same proce-dure as for **44a, b**ut using isothiazole **42** and 1-(6-chloropyridin-3-yl)-*N*-methylmethanamine (0.313 g, 2.0 mmol) at 0 °C and then stirring at r.t. for 5 h. Yield 0.290 g (86%).

*Alternative synthesis of thiazole***44e***:* To a suspension of 4,5-dichloro-*N*’-phenyl-1,2-thiazole-3-carbohydrazide (**44a**) (0.288 g, 1.0 mmol) in DMSO (10 mL), 1-(6-chloropyridin-3-yl)-*N*-methylmethanamine (0.313 g, 2.0 mmol) was added. The mixture was heated to 100 °C for 8 h. After cooling to r.t., water (50 mL) was added and the mixture extracted with chloroform (3 × 15 mL). Subsequently, the product was purified by column chromatography using petroleum ether-ethyl acetate (3: 1). The product was dried in vacuo. Yield 0.232 g (69%), a mixture of two rotamers in relation 10: 6, yellowish solid, m.p. 43–45 °C. IR (KBr) ν_max_ = 2934, 1651, 1460, 1349, 1106, 834 cm^−1^. ^1^H NMR (600 MHz, CDCl_3_) for major isomer δ = 2.90 (3H, s, NCH_3_), 4.73 (2H, s, NCH_2_), 7.34 (1H, d, *J* = 8.2 Hz, H pyr), 7.71 (1H, dd, *J* = 2.5 Hz, *J* = 8.3 Hz, H pyr), 8.37 (1H, d, *J* = 2.3 Hz, H pyr) ppm; minor isomer δ = 3.02 (1.8H, s, NCH_3_), 4.48 (1.2H, s, NCH_2_), 7.34 (0.6H, d, *J* = 8.2 Hz, H pyr), 7.72 (0.6H, dd, *J* = 2.5 Hz, *J* = 8.3 Hz, H pyr), 8.28 (0.6H, d, *J* = 2.3 Hz, H pyr) ppm; ^13^C NMR (150 MHz, CDCl_3_) for major isomer δ = 35.7 (NCH_3_), 47.8 (NCH_2_), 122.5 (CCl), 124.6 (CH), 130.7 (CH_2_C), 138.8 (CH), 149.2 (CH), 149.6 (CCl), 151.2 (NCCl), 159.6, 162.8 (C=O) ppm; minor isomer δ = 32.8 (NCH_3_), 51.2 (NCH_2_), 123.2 (CCl), 124.5 (CH), 130.3 (CH_2_C), 138.1 (CH), 148.9 (CH), 149.8 (CCl), 151.5 (NCCl), 159.5, 162.4 (C=O) ppm; ^15^N NMR (43.4 MHz, CDCl_3_, doped with nitromethane (0.0 ppm)) δ = -267.5 (NMe, minor isomer), -266.3 (NMe, major isomer), -75.7 (NCCl, major isomer), -75.5 (NCCl, minor isomer) ppm, NS not detected; MS *m/z* (*I*_rel_, %): 337 [M^+^] (3), 319 [M-CH_3_]^+^ (2), 300 [M-Cl]^+^ (6), 180 [M-CH_3_-N-CH_2_-isothiazole]^+^ (14), 155 [isothiazole-N-CH_2_-CH_3_]^+^ (100); HRMS (ESI^+^) *m/z* calcd for C_11_H_8_N_3_OCl_3_SNa [M + Na]^+^: 357.9351; found: 357.9352.

*4-Chloro-5-(morpholin-4-yl)-N’-phenyl-1,2-thiazole-3-carbohydrazide* (**45**). Following the alternative procedure for azole **44e** using morpholine (0.348 g, 4.0 mmol). The mixture was stirred at 90–95 °C for 4 h. Yield 0.264 g (78%), yellowish solid, m.p. 163–164 °C. IR (ATR) ν_max_ = 3294, 1695, 1494, 1403, 1115, 688 cm^−1^. ^1^H NMR (400 MHz, CDCl_3_) δ = 3.32–3.41 (4H, m, NCH_2_), 3.82–3.91 (4H, m, OCH_2_), 6.24 (1H, s, Ph-NH), 6.88–6.95 (3H, m, H Ph), 7.21–7.26 (2H, m, H Ph), 8.78 (1H, s, CONH) ppm; ^13^C NMR (100 MHz, CDCl_3_) δ = 50.8 (2 × NCH_2_), 66.0 (2 × OCH_2_), 108.9, 113.8 (2 × CH), 121.4 (CH), 129.2 (2 × CH), 147.7, 156.0 (NCC=O), 160.1 (C=O), 173.1 (morph-C) ppm; MS *m/z* (*I*_rel_, %): 338 [M^+^] (75), 303 [M-Cl]^+^ (8), 231 [M-PhNHNH]^+^ (35), 197 [M-PhNHNH-Cl+H]^+^ (100), 107 [PhNHNH]^+^ (60); HRMS (ESI^+^) *m/z* calcd for C_14_H_15_N_4_O_2_ClSNa [M + Na]^+^: 361.0502; found: 361.0502.

*Synthesis of 4-chloro-5-[(4-chlorophenyl)sulfanyl]-N’-phenyl-1,2-thiazole-3-carbohydrazide* (**46a**) *(General method).* To a solution of isothiazole **44a** (0.288 g, 1.0 mmol) in DMSO (10 mL), 4-chlorothiophenol (0.173 g, 1.2 mmol) and sodium ethanolate (0.082 g, 1.2 mmol) were added. The mixture was stirred at 110 °C for 8 h. After concentration at 100 °C, it was poured into 50 mL diluted HCl (5%) and extracted with chloroform (3 × 20 mL). The combined organic phases were washed with water, dried over calcium chloride, and purified by column chromatography using petroleum ether-ethyl acetate (2:1). Subsequently, the dried product was washed with cold methanol (1 × 3 mL). Yield 0.266 g (67%), white solid, m.p. 169–170 °C. IR (ATR) ν_max_ = 3250, 1655, 1493, 1094, 896, 695 cm^−1^. ^1^H NMR (400 MHz, CDCl_3_) δ = 5.11 (1H, s br, Ph-NH), 6.88–6.95 (3H, m, H Ph), 7.20–7.26 (2H, m, H Ph), 7.43 (2H, d, *J* = 8.5 Hz, H Ar), 7.53 (2H, d, *J* = 8.5 Hz, H Ar), 8.7 (1H, s, CONH) ppm; ^13^C NMR (100 MHz, CDCl_3_) δ = 113.8 (2 × CH), 121.6 (CH), 123.3, 128.2 (CCl), 129.2 (2 × CH), 130.5 (2 × CH), 134.7 (2 × CH), 136.7 (CS-Thiaz), 147.5, 155.5 (CC=O), 159.3, 160.9 (C=O) ppm; MS *m/z* (*I*_rel_, %): 395 [M^+^] (20), 288 [M-PhNHNH]^+^ (10), 261 [M-PhNHNHCO+H]^+^ (4), 225 [M-PhNHNHCO-Cl]^+^ (15), 107 [NHNHPh]^+^ (100); HRMS (ESI^+^) *m/z* calcd for C_16_H_11_N_3_OCl_2_S_2_Na [M + Na]^+^: 417.9618; found: 417.9617.

*4-Chloro-5-[(4-chlorophenyl)sulfanyl]-N’-[3-(trifluoromethyl)phenyl]-1,2-thiazole-3-carbohydrazide* (**46b**). Same procedure as for **46a,** but using **44b** (0.356 g, 1.0 mmol). The product was purified using petroleum ether-ethyl acetate (10: 1). Yield 0.325 g (70%), white solid, m.p. 144–145 °C. IR (ATR) ν_max_ = 3246, 1662, 1476, 1338, 1123, 694 cm^−1^. ^1^H NMR (400 MHz, CDCl_3_) δ = 6.39 (1H, s br, NH), 7.05 (1H, dd, *J* = 8.0 Hz, *J* = 2.1 Hz, H Ar), 7.11 (1H, s, H Ar), 7.15 (1H, d, *J* = 7.9 Hz, H Ar), 7.32 (1H, t, *J* = 8.0 Hz, H Ar), 7.44 (2H, d, *J* = 8.6 Hz, H Ar), 7.54 (2H, d, *J* = 8.6 Hz, H Ar), 8.77 (1H, s, NH). ^13^C (100 MHz, CDCl_3_) δ = 110.2 (*J*_C,F_ = 4.1 Hz, CH), 116.8 (CH), 118.1 (*J*_C,F_ = 3.9 Hz, CH), 123.9 (*J*_C,F_ = 272.5 Hz, CF_3_), 123.1 (CCl), 128.0 (CCl), 129.7 (CH), 130.9 (2 × CH), 131.6 (*J*_C,F_ = 32.2 Hz, CCF_3_), 134.8 (2 × CH), 136.9 (CS), 148.1 (N_2_H_2_C), 155.1 (SCS), 159.5 (CCO), 161.5 (C=O) ppm; MS *m/z* (*I*_rel_, %): 463 [M^+^] (35), 288 [M-N_2_H_2_PhCF_3_]^+^ (93), 225 [M-CON_2_H_2_PhCF_3_]^+^ (80), 145 [PhCF_3_]^+^ (100); HRMS (ESI^+^) *m/z* calcd for C_17_H_10_N_3_OCl_2_S_2_F_3_Na [M + Na]^+^: 485.9492; found: 485.9494.

*4-Chloro-5-[(4-chlorophenyl)sulfanyl]-N-(4-cyano-2,5-difluorophenyl)-1,2-thiazole-3-carboxamide* (**46c**). Same procedure as for **46a,** but using **44c** (0.334 g, 1.0 mmol). The product was purified by using petroleum ether-ethyl acetate (10: 1). Yield 0.367 g (83%), white solid, m.p. 152–153 °C. IR (ATR) ν_max_ = 3384, 2241 (CN), 1703, 1473, 1196, 617 cm^−1^. ^1^H NMR (400 MHz, CDCl_3_) δ = 7.24–7.66 (5H, m, H Ar), 8.57 (1H, s, H Ar), 9.47 (1H, s, NH) ppm; ^13^C NMR (101 MHz, CDCl_3_) δ = 95.4 (*J*_C,F_ = 19.8 Hz, 11.0 Hz, Ar CCN), 108.6 (*J*_C,F_ = 28.6 Hz, CH), 112.9 (Ph-CN), 118.3 (*J*_C,F_ = 24.2 Hz, CH), 123.0 (CCl), 127.6 (CCl), 130.7 (2 × CH), 131.9 (*J*_C,F_ = 11.7 Hz, CNH), 135.0 (2 × CH), 137.1 (SC), 147.5 (*J*_C,F_ = 244.3 Hz, CF), 154.9 (SCS), 156.9 (SNC), 160.2 (*J*_C,F_ = 253.8 Hz, CF), 163.0 (C=O) ppm; MS *m/z* (*I*_rel_, %): 441 [M^+^] (40), 288 [M-NHPhF_2_CN]^+^ (100), 225 [M-ClCOPhF_2_CN]^+^ (80), 143 [SPhCl]^+^ (53); HRMS (ESI^+^) *m/z* calcd for C_17_H_7_N_3_OCl_2_S_2_F_2_Na [M + Na]^+^: 463.9273; found: 463.9271.

*4-Chloro-5-[(4-chlorophenyl)sulfanyl]-N-[2-(1H-indol-3-yl)ethyl]-1,2-thiazole-3-carboxamide* (**46d**). Same procedure as for **46a,** but using **44d** (0.340 g, 1.0 mmol). After addition of HCl the crude product precipitated and was filtered off, washed with diluted HCl (3 mL), water (2 × 5 mL), cold MeOH (3 mL) and dried in vacuo. Yield 0.399 g (89%), white solid, m.p. 176–177 °C. IR (ATR) ν_max_ = 3280, 1667, 1525, 1338, 821, 740 cm^−1^. ^1^H NMR (400 MHz, DMSO-d_6_) δ = 2.88–2.99 (2H, m, Ind-CH_2_), 3.47–3.58 (2H, m, NCH_2_), 6.98 (1H, t, *J* = 7.0 Hz, H ind), 7.06 (1H, t, *J* = 7.0 Hz, H ind), 7.19 (1H, s, H ind), 7.34 (1H, d, *J* = 7.7 Hz, H ind), 7.51–7.73 (5H, m, H ind, 4H Ar), 8.84 (1H, s, CONH), 10.82 (1H, s, NH ind); ^13^C NMR (101 MHz, DMSO-d_6_) δ = 25.1 (ind-CH_2_), 39.7 (NCH_2_), 111.5, 111.6 (CH), 118.4 (CH), 118.5 (CH), 121.1 (CH), 121.5, 122.8 (NCH), 127.4, 128.8, 130.6 (2 × CH), 134.5 (2 × CH), 135.3 (SC), 136.4, 158.6 (CCO), 159.4 (SCS), 159.8 (C=O) ppm; MS *m/z* (*I*_rel_, %): 447 [M^+^] (3), 288 [M-NHC_2_H_4_-ind]^+^ (1), 143 [SPhCl]^+^ (100), 130 [I ndCH_2_]^+^ (53); HRMS (ESI^+^) *m/z* calcd for C_20_H_15_N_3_OCl_2_S_2_Na [M + Na]^+^: 469.9931; found: 469.9931.

*2-Ethoxy-2-oxoethyl 4-chloro-5-[(4-chlorophenyl)sulfanyl]-1,2-thiazole-3-carboxylate* (**46e**). Same proce-dure as for **46a,** but using **41** (0.284 g, 1.0 mmol). The product was purified by using petroleum ether-ethyl acetate (10: 1). Yield 0.267 g (68%), yellowish solid, m.p. 72–73 °C. IR (ATR) ν_max_ = 1755, 1722, 1344, 1198, 1084, 505 cm^−1^. ^1^H NMR (400 MHz, CDCl_3_) δ = 1.28 (3H, t, *J* = 7.0 Hz, OCH_2_CH_3_), 4.25 (2H, q, *J* = 7.0 Hz, OCH_2_), 4.87 (2H, s, OCH_2_), 7.42 (2H, d, *J* = 8.7 Hz, H Ar), 7.51 (2H, d, *J* = 8.6 Hz, H Ar) ppm; ^13^C NMR (101 MHz, CDCl_3_) δ = 14.1 (CH_3_), 61.6 (OCH_2_CH_3_), 61.7 (COOCH_2_), 124.0 (CCl), 128.1 (CCl), 130.5 (2 × CH), 134.6 (2 × CH), 136.7 (SC Ar), 153.5 (NC), 158.6, 160.9 (C=O), 166.8 (COOEt); MS *m/z* (*I*_rel_, %): 391 [M^+^] (48), 288 [M-OCH_2_CO_2_Et]^+^ (50), 225 [M-CO_2_CH_2_CO_2_Et-Cl]^+^ (70), 144 [ClPhSH]^+^ (100); HRMS (ESI^+^) *m/z* calcd for C_14_H_11_NO_4_Cl_2_S_2_Na [M + Na]^+^: 413.9404; found: 413.9405.

*Synthesis of 5-methyl-5-phenyl-3-(trichloroethenyl)-4,5-dihydro-1,2-oxazole* (**47**) *(General method).* To a solution of nitrodiene **1** (0.271 g, 1.0 mmol) in dry toluene (10 mL) prop-1-en-2-ylbenzene (1.182 g, 10.0 mmol), 18-crown-6 (0.066 g, 0.25 mmol), activated molar sieves 4 Å (0.250 g), and powdered NaOH (0.120 g, 3.0 mmol) were added under nitrogen atmosphere. The mixture was then stirred at 60 °C for 16 h, and at 80 °C for another 16 h. After completion of the reaction, the solvent was evaporated, 10% aq. HCl (10 mL) was added, and the mixture was extracted with DCM (3 × 10 mL). The crude product was purified by column chromatography using petroleum ether-ethyl acetate (10: 1). Yield 0.221 g (76%), yellow viscous oil. IR (ATR) ν_max_ = 2977, 1684, 1446, 1265, 859, 697 cm^−1^. ^1^H NMR (400 MHz, CDCl_3_) δ = 1.77 (3H, s, CH_3_), 3.43 (1H, d, *J* = 16.9 Hz, ONCCH_2_), 3.50 (1H, d, *J* = 16.9 Hz, ONCH_2_), 7.37–7.48 (5H, m, H Ph) ppm; ^13^C NMR (100 MHz, CDCl_3_) δ = 27.8 (CH_3_), 50.1 (CH_2_), 90.3 (OCCH_3_), 121.7, 124.5 (2 × CH), 127.7 (CH), 128.6 (2 × CH), 137.1 (CCl_2_), 144.2, 152.5 (NCCCl) ppm; MS *m/z* (*I*_rel_, %): 289 [M^+^] (4), 274 [M-CH_3_]^+^ (4), 117 [PhC_2_H_5_]^+^ (60), 105 (100); HRMS (ESI^+^) *m/z* calcd for C_12_H_10_NOCl_3_Na [M + Na]^+^: 311.9726; found: 311.9725.

*tert-Butyl 3-(trichloroethenyl)-4,5-dihydro-1,2-oxazole-5-carboxylate* (**48**). Same procedure as for **47**, but using *tert*-butyl prop-2-enoate (1.282 g, 10.0 mmol). Yield 0.195 g (65%), yellowish oil. IR (ATR) ν_max_ = 2981, 1732, 1368, 1232, 1150, 838 cm^−1^. ^1^H NMR (400 MHz, CDCl_3_) δ = 1.47 (9H, s, CCH_3_), 3.53 (2H, d, *J* = 9.4 Hz, ONCCH_2_), 5.03 (1H, t, *J* = 9.4 Hz, OCH) ppm; ^13^C NMR (100 MHz, CDCl_3_) δ = 27.8 (3 × CH_3_), 40.0 (CH_2_), 80.2 (OCH), 83.1 (OCMe_3_), 120.5 (CCl), 125.2 (CCl_2_), 152.3 (ONC), 168.0 (C=O) ppm; ^15^N NMR (43.4 MHz, CDCl_3_, doped with nitromethane (0.0 ppm)) δ = 7.8 ppm; MS *m/z* (*I*_rel_, %): 299 [M^+^] (12), 200 [M-CO_2_CMe_3_]^+^ (100), 170 [M-C_2_Cl_3_]^+^ (12), 129 [C_2_Cl_3_]^+^ (52), 100 [CO_2_CMe_3_-H]^+^ (95); HRMS (ESI^+^) *m/z* calcd for C_10_H_12_NO_3_Cl_3_Na [M + Na]^+^: 321.9781; found: 321.9781.

*2-Ethylhexyl 3-(trichloroethenyl)-4,5-dihydro-1,2-oxazole-5-carboxylate* (**49**). Same procedure as for **47**, but using 2-ethylhexyl prop-2-enoate (1.843 g, 10.0 mmol). Yield 0.216 g (60%), yellowish oil. IR (ATR) ν_max_ = 2958, 2860, 1732, 1461, 1162, 851 cm^−1^. ^1^H NMR (400 MHz, CDCl_3_) δ = 0.86–0.88 (6H, m, 2 × CH_3_), 1.19–1.42 (8H, m, 4 × CH_2_), 1.60–1.64 (1H, m, CH), 3.60 (1H, d, *J* = 10.7 Hz, ONCCH_2_), 3.61 (1H, d, *J* = 7.8 Hz, ONCCH_2_), 4.06–4.18 (2H, m, OCH_2_), 5.17 (1H, dd, *J* = 10.7 Hz, *J* = 7.7 Hz, OCH) ppm; ^13^C NMR (100 MHz, CDCl_3_) δ = 10.9 (CH_3_), 14.0 (CH_3_), 22.9 (CH_2_), 23.6 (CH_2_), 28.8 (CH_2_), 30.2 (CH_2_), 28.6 (CH), 40.2 (ONCCH_2_), 68.5 (OCH_2_), 79.6 (OCH), 120.4 (CCl), 125.5 (CCl_2_), 152.4 (ONC), 169.2 (C=O) ppm; MS *m/z* (*I*_rel_, %): 355 [M^+^] (10), 320 [M-Cl]^+^ (22), 198 [M-CO_2_C_8_H_17_]^+^ (100); HRMS (ESI^+^) *m/z* calcd for C_14_H_20_NO_3_Cl_3_Na [M + Na]^+^: 378.0407; found: 378.0406.

*3-(Trichloroethenyl)-3a,4,5,6,7,7a-hexahydro-1,2-benzoxazole* (**50**). Same procedure as for **47**, but using cyclohexene (0.821 g, 10.0 mmol). Yield 0.104 g (41%), yellowish oil. IR (ATR) ν_max_ = 2935, 2863, 1448, 912, 838, 739 cm^−1^. ^1^H NMR (400 MHz, CDCl_3_) δ = 1.23–1.43 (2H, m, CH_2_), 1.48–1.64 (3H, m, CH_2_), 1.72–1.82 (1H, m, CH_2_), 1.83–1.93 (1H, m, CH_2_), 2.00–2.09 (1H, m, CH_2_), 3.32–3.41 (1H, m, CH), 4.58 (1H, ddd, *J* = 8.2 Hz, *J* = 4.2 Hz, *J* = 4.2 Hz, CH) ppm; ^13^C (100 MHz, CDCl_3_) δ = 19.9 (CH_2_), 21.7 (CH_2_), 24.8 (CH_2_), 25.3 (CH_2_), 46.1 (CH), 81.4 (CH), 121.0 (CCl), 124.4 (CCl_2_), 159.1 (ONC) ppm; MS *m/z* (*I*_rel_, %): 253 [M^+^] (75), 236 [M-OH]^+^ (5), 218 [M-Cl]^+^ (7), 182 [M-Cl-HCl]^+^ (16), 91 (100); HRMS (ESI^+^) *m/z* calcd for C_9_H_10_NOCl_3_Na [M + Na]^+^: 275.9726; found: 275.9725.

*Synthesis of 1-[1,1-dichloro-3-(1,3-dithiolan-2-ylidene)-3-nitroprop-1-en-2-yl]-4-(4-fluorophenyl)pipera- zine* (**52**). A solution of dithiolane **51** (0.292 g, 1.0 mmol) in MeOH (10 mL) and 1-(4-fluorophenyl)piperazine (0.433 g, 2.4 mmol) was stirred at reflux for 7 d. After cooling, the solution was concentrated and treated with water for 30 min. The resulting precipitate was filtered off and washed with water (2 × 5 mL). Yield 0.393 g (90%), orange solid, m.p. 47–50 °C. IR (KBr) ν_max_ = 2825, 1509, 1272, 1226, 954, 816 cm^−1^. ^1^H NMR (200 MHz, CDCl_3_) δ = 3.08–3.39 (8H, m, NCH_2_), 3.48–3.61 (4H, m, SCH_2_), 6.80–7.11 (4H, m, H Ar) ppm; ^13^C NMR (50 MHz, CDCl_3_) δ = 37.6 (SCH_2_), 40.1 (SCH_2_), 49.2 (2 × NCH_2_), 50.6 (2 × NCH_2_), 113.3 (CCl_2_), 115.5 (*J*_C,F_ = 21.9 Hz, 2 × CH), 118.2 (*J*_C,F_ = 7.7 Hz, 2 × CH), 130.9 (CNO_2_), 139.0 (CN), 147.9 (*J*_C,F_ = 2.2 Hz, NC Ar), 157.3 (*J*_C,F_ = 238.9 Hz, CF), 170.4 (SCS) ppm; MS *m/z* (*I*_rel_, %): 439 [M^+^] (25), 418 [M − OH]^+^ (4), 389 [M − NO_2_]^+^ (7), 179 [F-Ph-piperazine]^+^ (15), 122 (100); HRMS *m/z* calcd for C_16_H_16_N_3_O_2_FCl_2_S_2_ [M]^+^: 435.0045; found: 435.0047.

*Synthesis of 1-[2-Chloro-5-(ethenylsulfanyl)-4-nitrothiophen-3-yl]-4-(4-fluorophenyl)piperazine* (**53**). An aqueous solution of NaOH (3.0 mmol, 40%) was added dropwise to a solution of dithiolane **52** (0.436 g, 1.0 mmol) in DMSO (10 mL) at 0 °C. After 1 h at 0 °C, the mixture was stirred at r.t. for an additional 3 h. Subsequently, cold water was added and the mixture was treated dropwise with diluted HCl until a precipitate formed. The precipitate was filtered off and washed with water (2 × 5 mL) and cold MeOH (3 mL). Yield 0.340 g (85%), yellowish solid, m.p. 83–86 °C. IR (KBr) ν_max_ = 2829, 1543, 1511, 1322, 1233, 831 cm^−1^. ^1^H NMR (200 MHz, CDCl_3_) δ = 3.16–3.26 (4H, m, NCH_2_), 3.31–3.47 (4H, m, NCH_2_), 5.83 (1H, d, *J* = 9.1 Hz, SCHCH_2_), 8.55 (1H, d, *J* = 5.9 Hz, SCHCH_2_), 6.55 (1H, dd, *J* = 16.4 Hz, *J* = 9.2 Hz, SCH), 6.86–7.09 (4H, m, H Ar) ppm; ^13^C NMR (50 MHz, CDCl_3_) δ = 50.0 (2 × NCH_2_), 51.5 (2 × NCH_2_), 115.6 (*J*_C,F_ = 21,9 Hz, 2 × CH), 118.6 (*J*_C,F_ = 8.1 Hz, 2 × CH), 119.1 (CCl), 126.2 (CH_2_ vin), 126.8 (CH vin), 139.4 (CNO_2_), 141.0 (CN), 143.3 (SCS), 148.5 (*J*_C,F_ = 3.7 Hz, NC Ar), 157.7 (*J*_C,F_ = 234.5 Hz, CF) ppm; MS *m/z* (*I*_rel_, %): 399 [M^+^] (85), 352 [M-HNO_2_]^+^ (17), 317 [M-HNO_2_-Cl]^+^ (4), 122 (100); HRMS (ESI^+^) *m/z* calcd for C_16_H_15_N_3_O_2_ClFS_2_ [M + H]^+^: 399.0278; found: 399.0278.

*Synthesis of 5-(Ethenylsulfanyl)-3-[4-(4-fluorophenyl)piperazin-1-yl]-4-nitrothiophene-2-carbaldehyde* (**54**). A solution of thiophene **53** (0.400 g, 1.0 mmol) and POCl_3_ (0.307 g, 2.0 mmol) in dry DMF was stirred at r.t. for 1 d and then at 55 °C for 2 d. After cooling down to 0 °C, cold water (15 mL) was added and the mixture extracted with chloroform (3 × 10 mL). After drying over CaCl_2_, the crude product was purified by column chromatography using petroleum ether-ethyl acetate (2: 1). Yield 0.252 g (64%), orange solid, m.p. 104–105 °C. IR (ATR) ν_max_ = 2833, 1621, 1531, 1505, 1223, 997 cm^−1^. ^1^H NMR (400 MHz, CDCl_3_) δ = 3.25–3.33 (4H, m, NCH_2_), 3.50–3.59 (4H, m, OCH_2_), 5.99 (2H, dd, *J* = 16.7 Hz, *J* = 9.4 Hz, CH_2_ vin), 6.65 (1H, dd, *J* = 16.6 Hz, *J* = 9.3 Hz, SCH vin), 6.88–7.03 (4H, m, H Ar), 10.03 (1H, s, CHO) ppm; ^13^C NMR (100 MHz, CDCl_3_) δ = 50.7 (2 × NCH_2_), 53.5 (2 × OCH_2_), 115.6, 115.7 (*J*_C,F_ = 22.2 Hz, 2 × CH Ar), 118.6 (*J*_C,F_ = 7.7 Hz, 2 × CH Ar), 124.7 (CN), 125.6 (SCH), 128.7 (CH_2_ vin), 136.2 (CNO_2_), 147.6 (*J*_C,F_ = 2.2 Hz, NC Ar), 150.5 (SCS), 157.6 (*J*_C,F_ = 239.9 Hz, CF), 159.8 (SC), 180.2 (CHO) ppm; MS *m/z* (*I*_rel_, %): 393 [M^+^] (15), 376 [M − OH]^+^ (5), 346 [M − HNO_2_]^+^ (3), 179 [piperazine-Ph-F]^+^ (7), 122 (100); HRMS (ESI^+^) *m/z* calcd for C_17_H_16_N_3_FO_3_S_2_Na [M + Na]^+^: 416.0515 ; found: 416.0514.

*Synthesis of {[5-(Ethenylsulfonyl)-3-(morpholin-4-yl)-4-nitrothiophen-2-yl]methylidene}propanedinitrile* (**57**). A solution of thiophene **56** (0.348 g, 1.0 mmol) and hydrogen peroxide (3.0 mmol, 35%) in acetic acid was stirred at 50–55 °C for 3 h. After cooling, ice was poured into the mixture and the resulting precipitate was filtered off and washed with water (3 × 5 mL). Yield 0.312 g (82%), red solid, m.p. 121–123 °C. IR (KBr) ν_max_ = 2857, 2223 (CN), 1541, 1340, 1111, 857 cm^−1^. ^1^H NMR (200 MHz, CDCl_3_) δ = 3.23–3.30 (4H, m, NCH_2_), 3.84–3.90 (4H, m, OCH_2_), 6.15 (1H, dd, *J* = 9.3, *J* = 1.4, CH_2, cis_ vin), 6.45 (1H, dd, *J* = 16.2, *J* = 1.3, CH_2, trans_ vin), 7.09 (1H, dd, *J* = 16.3, *J* = 9.3, CH vin), 8.02 (1H, s, SCCH). ^13^C NMR (50 MHz, CDCl_3_) δ = 52.0 (2 × NCH_2_), 67.0 (2 × OCH_2_), 80.3 (C(CN)_2_), 112.2 (2 × CN), 113.3, 124.1 (CH_2_ vin), 127.4 (SC), 138.5 (CH vin), 146.8 (SCCH), 151.4 (C-morph), 166.1 (SO_2_C), CNO_2_ could not be detected. Mass spectrum, *m/z* (*I*_rel_, %): 380 [M^+^] (3), 363 [M-OH]^+^ (20), 346 [M-2(OH)]^+^ (100), 289 [M-SO_2_C_2_H_3_]^+^ (2); HRMS (ESI^+^) *m/z* calcd for C_14_H_12_N_4_O_5_S_2_Na [M + Na]^+^: 403.0147; found: 403.0146.

*Synthesis of 2,2’-{[5-(Ethenylsulfanyl)-3-(morpholin-4-yl)-4-nitrothiophen-2-yl]methanediyl}bis(5,5- di-methylcyclohexane-1,3-dione)* (**58**). A solution of thiophene **55** (0.300 g, 1.0 mmol), dimedone (0.308 g, 2.2 mmol) and pyridine (8.0 mg, 0.1 mmol) in MeOH (15 mL) was stirred at r.t. for 1 d and then at 35–40 °C for 2 d. After cooling to r.t., the mixture was treated with diluted HCl and the resulting precipitate was filtered off and washed with water (2 × 5 mL) and cold Et_2_O (3 mL). Yield 0.411 g (73%), yellow solid, m.p. 159–160 °C. IR (KBr) ν_max_ = 3243, 2961, 1717, 1626, 1542, 1068 cm^−1^. ^1^H NMR (200 MHz, CDCl_3_) δ = 1.03 (12H, s, 4CH_3_), 2.34 (8H, s, 4COCH_2_), 2.60–3.80 (10H, m, 2NCH_2_, 2OCH_2_, 2COCH), 5.77 (2H, dd, *J* = 16.4 Hz, *J* = 9.3 Hz, CH_2_ vin), 6.41–6.51 (1H, m, SCH), 6.67 (1H, dd, *J* = 16.4 Hz, *J* = 9.3 Hz, CH vin) ppm; ^13^C NMR (50 MHz, CDCl_3_) δ = 26.7 (SCCH), 28.1 (2 × CH_3_C), 31.6 (4 × CH_3_), 46.7 (2 × NCH_2_), 49.6 (4 × COCH_2_), 67.4 (2 × OCH_2_), 77.2 (2 × CC=O), 116.1 (SC), 124.4 (CH_2_ vin), 127.6 (SCH), 137.2 (CNO_2_), 141.1 (C-morph), 143.2 (SCS), 189.9 (4 × C=O) ppm; MS *m/z* (*I*_rel_, %): 562 [M^+^] (12), 543 [M-H_2_O-H]^+^ (12), 526 [M-2(H_2_O)]^+^ (42), 458 [M-SC_2_H_3_-NO_2_+H]^+^ (10); HRMS (ESI^+^) *m/z* calcd for C_27_H_34_N_2_O_7_S_2_Na [M + Na]^+^: 585.1705; found: 585.1705.

*Synthesis of 4-[5-(Ethenylsulfanyl)-4-nitro-2-{[2-(2,4,6-trichlorophenyl)hydrazinylidene]methyl} thiophen-3-yl]morpholine* (**59**). A solution of thiophene **55** (0.300 g, 1.0 mmol) and (2,4,6-trichlorophenyl)-hydrazine (0.634 g, 3.0 mmol) in MeOH (15 mL) was refluxed for 3 h. After cooling to r.t., the mixture was treated with diluted HCl and the resulting precipitate was filtered off and washed with water (2 × 5 mL) and cold MeOH (3 mL). Yield 0.425 g (86%), orange solid, m.p. 192–194 °C. IR (KBr) ν_max_ = 3228, 1520, 1445, 1332, 1107, 851 cm^−1^. ^1^H NMR (200 MHz, CDCl_3_) δ = 3.05–3.19 (4H, m, NCH_2_), 3.75–3.82 (4H, m, OCH_2_), 5.87 (2H, dd, *J* = 16.7 Hz, *J* = 9.4 Hz, CH_2_ vin), 6.65 (1H, dd, *J* = 16.7 Hz, *J* = 9.4 Hz, SCH vin), 7.36 (2H, s, H Ar), 7.64 (1H, br s, NH), 8.01 (1H, s, NCH) ppm; ^13^C (50 MHz, CDCl_3_) δ = 51.0 (2 × NCH_2_), 67.5 (2 × OCH_2_), 126.4 (CH_2_ vin), 126.8 (SCH vin), 127.6 (2 × CCl), 129.0 (2 × CH), 129.1 (CCl), 130.1 (SC), 133.3 (NCH), 135.8 (NHC), 139.5 (CNO_2_), 142.3 (SCS), 148.5 (C-morph) ppm; MS *m/z* (*I*_rel_, %): 492 [M^+^] (12), 433 [M-SC_2_H_3_]^+^ (2), 298 [M-NHPhCl_3_]^+^ (55), 195 [H_2_NPhCl_3_]^+^ (100); HRMS (ESI^+^) *m/z* calcd for C_17_H_15_N_4_O_3_Cl_3_S_2_Na [M + Na]^+^: 514.9549; found: 514.9547.

*Synthesis of 5-{[5-(Ethenylsulfanyl)-3-(morpholin-4-yl)-4-nitrothiophen-2-yl]methylidene}-2,2-dimethyl- 1,3-dioxane-4,6-dione* (**60**). A solution of thiophene **55** (0.300 g, 1.0 mmol), 2,2-dimethyl-1,3-dioxane-4,6-dione (0.288 g, 2.0 mmol) and piperidine (8.5 mg, 0.1 mmol) in MeOH (15 mL) was stirred at r.t. for 1 d. Then another 0.288 g of 2,2-dimethyl-1,3-dioxane-4,6-dione were added and the mixture was stirred for an additional 2 d. Subsequently, the mixture was treated with diluted HCl and the resulting precipitate was filtered off and washed with water (2 × 5 mL) and cold MeOH (3 mL). Yield 0.333 g (73%), red solid, m.p. 173–174 °C. IR (ATR) ν_max_ = 1683, 1516, 1332, 1197, 1113, 787 cm^−1^. ^1^H NMR (200 MHz, CDCl_3_) δ = 1.76 (6H, s, CH_3_), 3.33–3.39 (4H, m, NCH_2_), 3.93–3.96 (4H, m, OCH_2_), 6.00 (2H, dd, *J* = 16.8 Hz, *J* = 9.5 Hz, CH_2_ vin), 6.75 (1H, dd, *J* = 16.8 Hz, *J* = 9.5 Hz, SCH vin), 8.68 (1H, s, SCCH) ppm; ^13^C (200 MHz, CDCl_3_) δ = 27.5 (2 × CH_3_), 53.2 (2 × NCH_2_), 67.1 (2 × OCH_2_), 103.6 (CMe_2_), 104.7 (CC=O), 119.5 (SC), 124.9 (SCH vin), 128.7 (CH_2_ vin), 137.2 (CNO_2_), 144.1 (SCCH), 156.9 (SCS), 161.4 (C-morph), 162.3 (C=O), 163.4 (C=O) ppm; MS *m/z* (*I*_rel_, %): 426 [M^+^] (100), 409 [M-OH]^+^ (10), 351 [M-Me_2_CO_2_H]^+^ (24), 295 [M-NO_2_-morph+H]^+^ (27); HRMS (ESI^+^) *m/z* calcd for C_17_H_18_N_2_O_7_S_2_Na [M + Na]^+^: 449.0453; found: 449.0453.

### 3.3. Evaluation of Biological Activity

For a first evaluation of the biological activity of heterocycles, the influence of the compounds on the growth of bacteria, namely of the Gram-positive strain *Staphylococcus aureus* SH−1000 and of the Gram-negative uropathogenic strain *Escherichia coli UPEC796*, and on the viability of mammalian cells, namely the murine fibroblast cell line L929, was evaluated.

Bacteria were cultivated in the complex Lysogeny broth. An aliquot of an overnight culture was diluted with fresh medium to an OD_600_ (optical density at 600 nm) of 0.1–0.2. This intermediate culture was incubated at 37 °C with shaking until an OD_600_ = 0.5 was reached to generate a culture of exponentially growing cells. The cells were again diluted with fresh medium to an OD_600_ = 0.2 to generate the working culture.

In each well of a 96-well plate, 90 µl Lysogeny broth were placed, to which 1.8 µl of compound solutions were added. The incubation was started after the addition of 90 µl of the working culture, resulting in a total volume of 180 µl. The microtiter plates were incubated at 37 °C. Bacterial growth was followed by determination of the OD_600_ with the microplate spectrophotometer PowerWave^TM^ (BioTek; Bad Friedrichshall, Germany) in regular time intervals, starting 2 h after the inoculation. The final value was obtained after a growth period of 24 h.

L929 cells were cultivated in RPMI cell culture medium, supplemented with 10% FBS (serum) in a cell culture incubator at 37 °C and 10% CO_2_. 60 µl of a cell suspension with 3_*_10^4^ cells/mL were seeded in each well of an assay-ready 96 well microtiter plate. The assay-ready microtiter plates were prepared from the 10 mM DMSO stock solutions of the compounds with the Echo^®^ 525 acoustic liquid handler (Labcyte Inc., USA). The microtiter plates with compounds and cells were incubated at 37 °C, 10% CO_2_ in a cell culture incubator for three days. The remaining viability of cells was assessed with the alamarBlue^®^ assay [88] (Thermo Fisher Scientific (Waltham, MA, USA) according to the instructions given by the manufacturer, i.e., 5 µl of the resazurin-solution were given in each well of the 96-well plate and incubated for up to 4 h. The turnover was determined via the fluorescence of resorufin (λ_ex_ = 540 nm; λ_em_ = 600 nm) with the multi-mode microplate reader Synergy™ 4 (BioTek).

The primary evaluation of the biological activity was done with a single concentration of the compounds, which was 100 µM in the bacterial assays and 10 µM in the cell culture assay. Compounds were considered to show activity in these assays, when the residual growth or viability, respectively, was reduced to 50% or less of the growth or viability of an untreated bacterial or cell culture. The activities of these compounds were validated by investigating the influences of compound concentrations and determinations of the EC_50_ values (concentration resulting in 50% of the observed effect). Diluted compound solutions were prepared either by manual serial dilutions using eight channel pipettes or again with the Echo^®^ 525 transfering varying volumes from the compound plate to the assay plate.

The EC_50_ values were determined by nonlinear regression with a 4-parameter equation using the respective module from GraphPadPrism.

## 4. Conclusions

Starting from three polyhalogenated nitro-1,3-butadienes, we developed an efficient and practical strategy for the multigram synthesis of the following heterocycles with unique substitution patterns: benzoxazolines, benzimidazolines, imidazolidines, Imidacloprid analogues, thia-zolidinones, pyrimidines, pyrazoles, 4*H*-pyrido[1,2-*a*]pyrimidines, benzo[*h*]quinolines, isothiazoles, dihydroisoxazoles, and thiophenes. Quite some of these heterocycles deserve interest as key units in synthesis, chemical biology, and medicinal chemistry. Successive synthetic modifications of the heterocycles are predictable and feasible.

## Supplementary Materials

The following are available online, Figures S1–S203: ^1^H–NMR, ^13^C–NMR, ^15^N, ^1^H–HMBC–NMR, and mass spectra. Figures S204 and S205: Biological profiling of compounds **4a–58**.
