# Supplementary material for: Polyhalonitrobutadienes as Versatile Building Blocks for the Biotargeted Synthesis of Substituted N-Heterocyclic Compounds
Source: Molecules. 2020 Jun 21;25(12):2863. doi: 10.3390/molecules25122863 (PMC7355852; doi:10.3390/molecules25122863)
Supplement: Supplementary file 1 [file molecules-25-02863-s001.zip › molecules-805501-supplementary.pdf]

Supplementary Material

Polyhalonitrobutadienes as versatile building blocks for the targeted synthesis of substituted N-heterocyclic compounds [‡]

Viktor A. Zapol'skii [1], Ursula Bilitewski [2], Sören R. Kupiec [1], Isabell Ramming [2], and Dieter E. Kaufmann [1,*]

[1] Institute of Organic Chemistry, Clausthal University of Technology, Leibnizstraße 6, 38678 Clausthal-Zellerfeld, Germany

[2] Helmholtz Centre for Infection Research (HZI), Inhoffenstr. 7, Braunschweig, 38124, Germany

[‡] Chemistry of Polyhalogenated Nitrobutadienes, 16.
Part 15: V. A. Zapol'skii, J. C. Namyslo, M. Gjikaj, D. E. Kaufmann, Synthesis of Novel 4-Nitro-3-amino-1*H*-pyrazole-5-carbaldehydes and Pyrazolo[3,4-f]indazole-4,8-diones. *Heterocycles*, **2016**, *93*, 2, 628-646; DOI: 10.3987/COM-15-S(T)46.

* Author to whom correspondence should be addressed.
dieter.kaufmann@tu-clausthal.de

Figure S1–S203. ^{1}H–NMR, ^{13}C–NMR, ^{15}N, ^{1}H–HMBC–NMR and mass spectra.
Figure S204. Biological profiling of compounds **4a-32b.**
Figure S205. Biological profiling of compounds **33a-60.**

Figure S1. 200 MHz ^{1}H-NMR spectrum in $CDCl_3$ for **4a**.

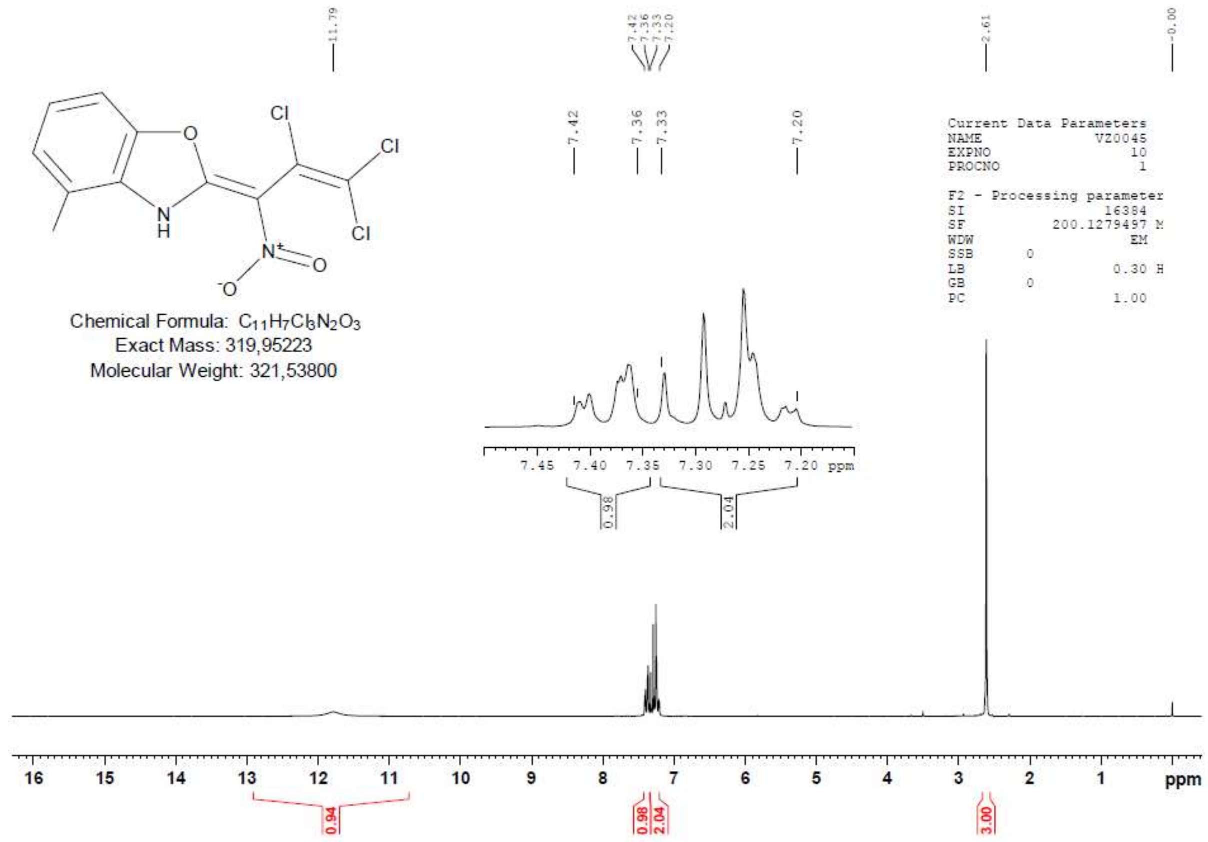

Figure S2. 50 MHz ^{13}C-NMR spectrum in $CDCl_3$ for **4a**.

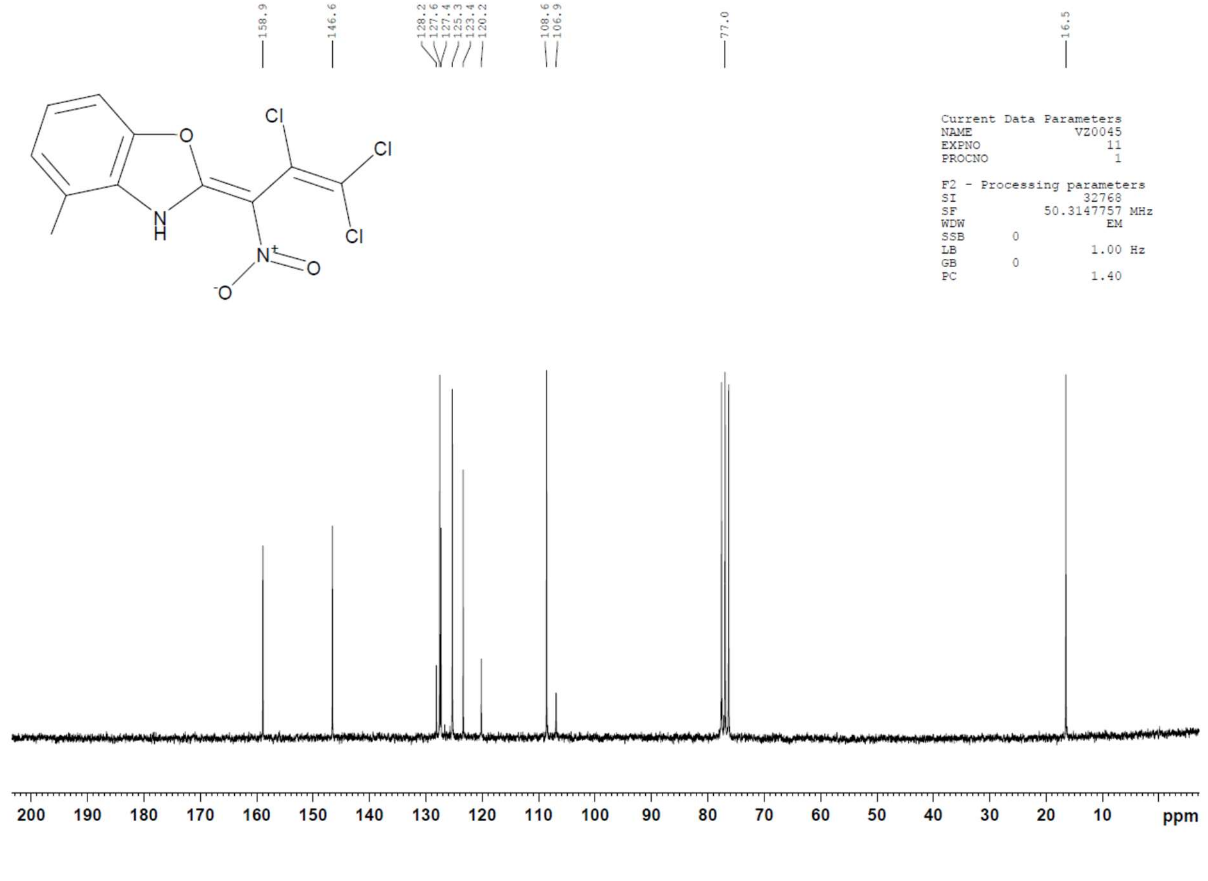

Figure S3. Mass spectrum for 4-methyl-2-(2,3,3-trichloro-1-nitroprop-2-en-1-ylidene)-2,3-dihydro-1,3-benzoxazole (**4a)**.

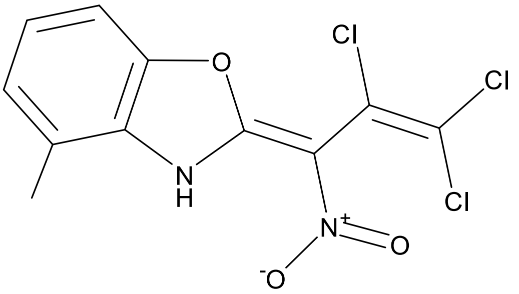

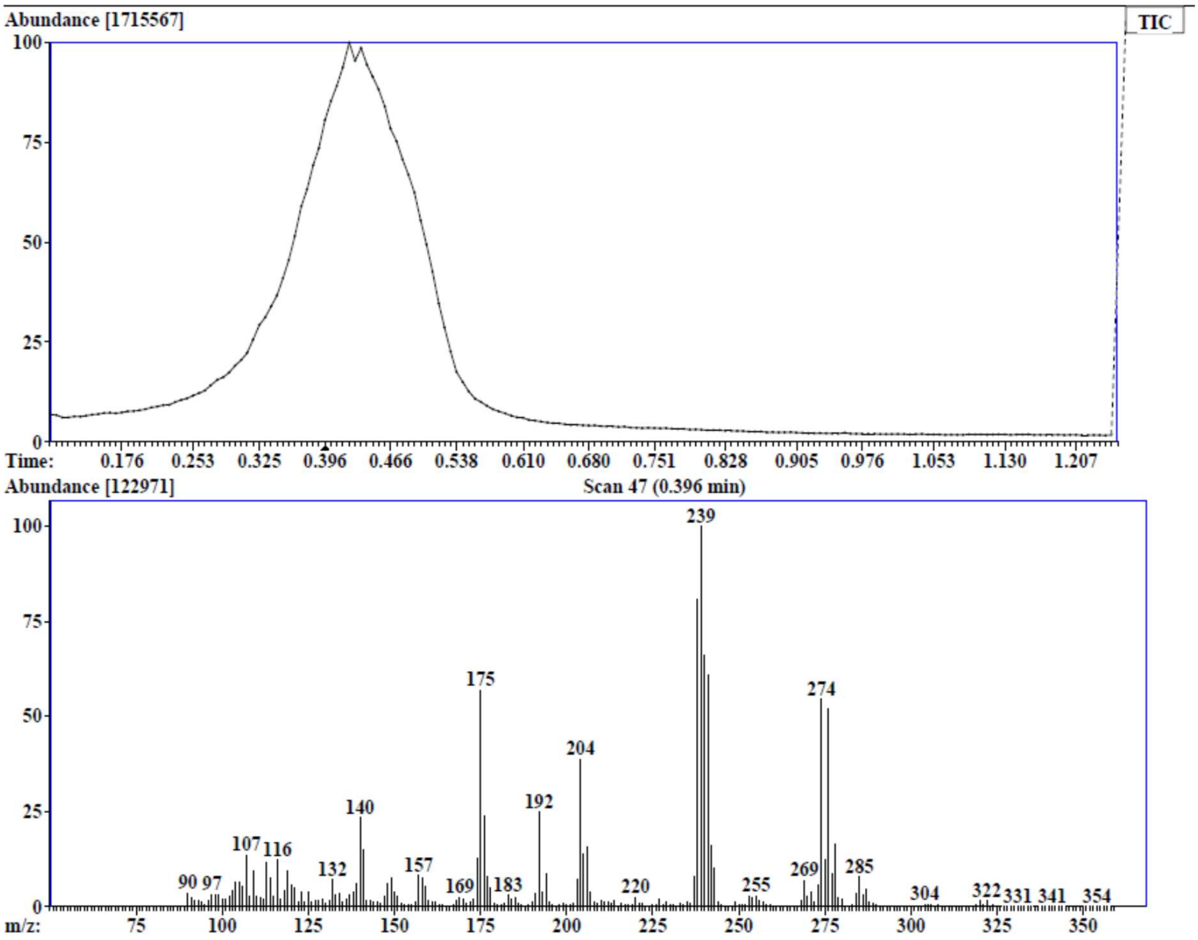

Figure S4. 400 MHz ^{1}H-NMR spectrum in DMSO-*d6* for **4b**.

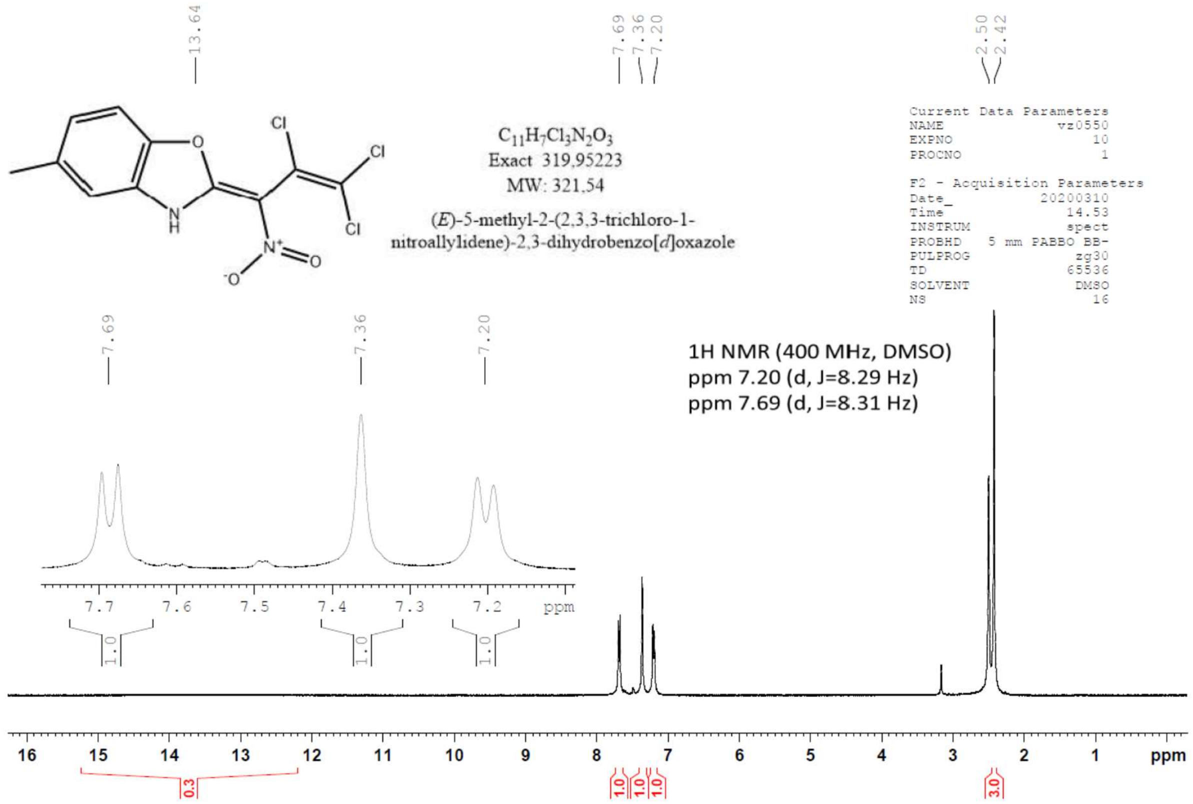

Figure S5. 100 MHz ^{13}C-NMR spectrum in DMSO-*d6* for **4b**.

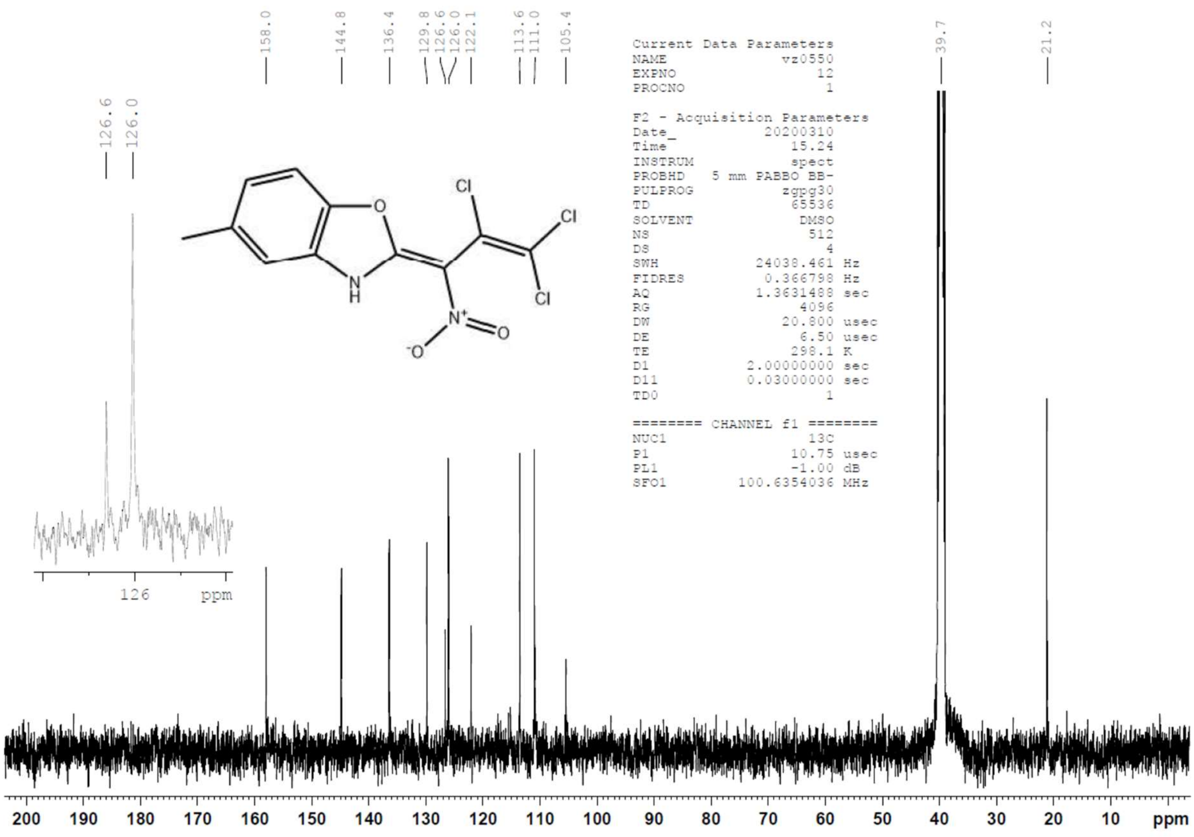

Figure S6. Mass spectrum for 5-methyl-2-(2,3,3-trichloro-1-nitroprop-2-en-1-ylidene)-2,3-dihydro-1,3-benzoxazole (**4b**).

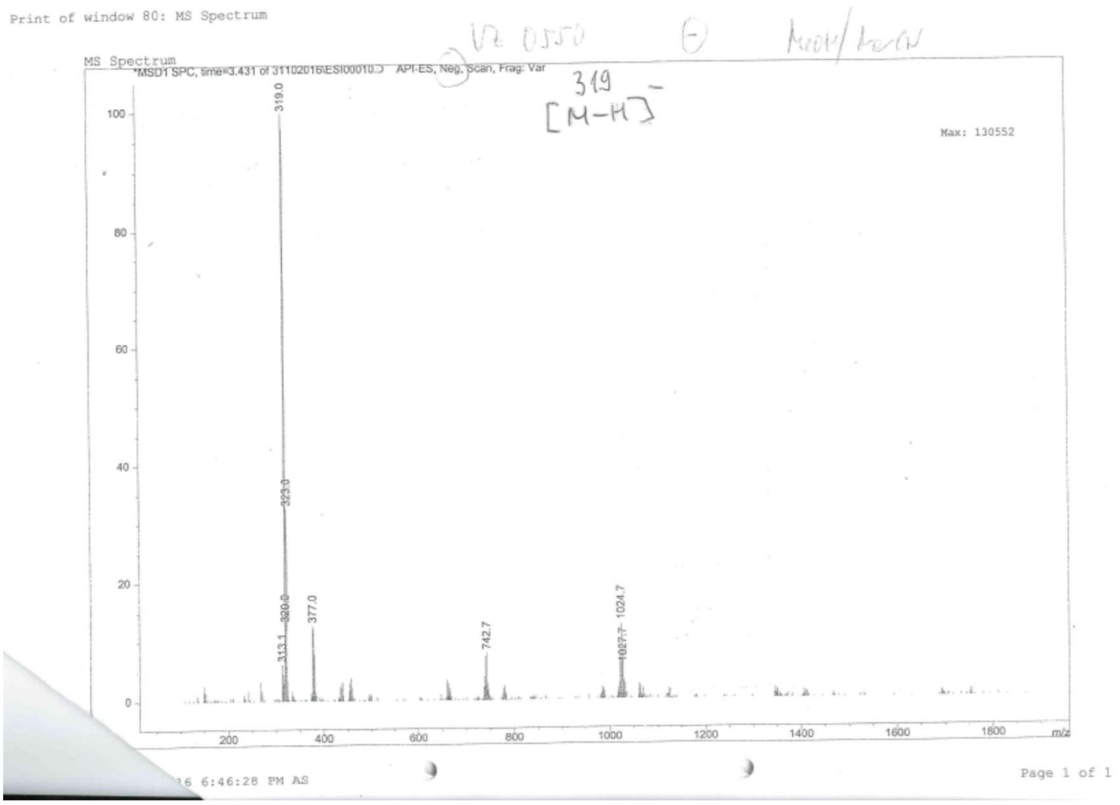

Figure S7. HR-MS spectrum for 5-methyl-2-(2,3,3-trichloro-1-nitroprop-2-en-1-ylidene)-2,3-dihydro-1,3-benzoxazole (**4b**).

Elemental Composition Report **Page 1**

Single Mass Analysis (displaying only valid results)
Tolerance = 10.0 PPM / DBE: min = -1.5, max = 50.0
Selected filters: None

Monoisotopic Mass, Even Electron Ions
603 formula(e) evaluated with 6 results within limits (up to 80 closest results for each mass)
Elements Used:
C: 0-50 H: 0-60 N: 0-2 O: 0-7 Na: 0-1 Cl: 0-3
Zapolski LCT Premier KD070
VZ 0550 8 (0.179) AM (Cen,4, 20.00, Ar,11000.0,554.26,0.70,LS 5); Cm (5:28) 1: TOF MS ES- 1.01e4

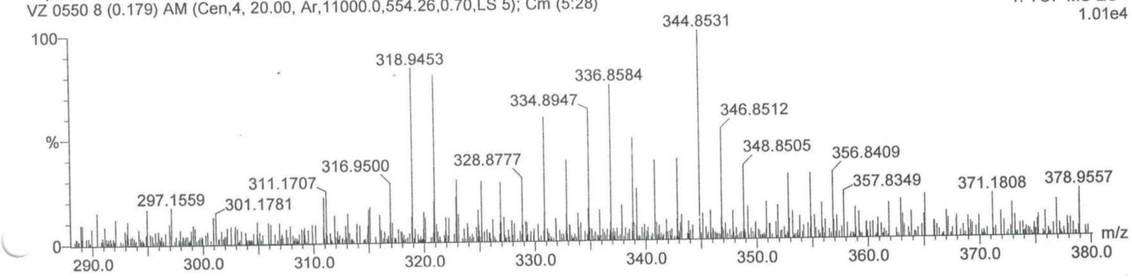

Minimum:				-1.5		
Maximum:		5.0	10.0	50.0		
Mass	Calc. Mass	mDa	PPM	DBE	i-FIT	Formula
318.9453	318.9460	-0.7	-2.2	9.5	5.0	C14 H7 O Na Cl3
	318.9444	0.9	2.8	8.5	37.6	C11 H6 N2 O3 Cl3
	318.9442	1.1	3.4	14.5	303.5	C15 H2 N2 O Na Cl2
	318.9466	-1.3	-4.1	17.5	338.9	C17 H N2 O Cl2
	318.9434	1.9	6.0	17.5	1541.3	C17 O5 Cl
	318.9484	-3.1	-9.7	12.5	5.4	C16 H6 O Cl3

Figure S8. 400 MHz ^{1}H-NMR spectrum in DMSO-*d6* for **5a**.

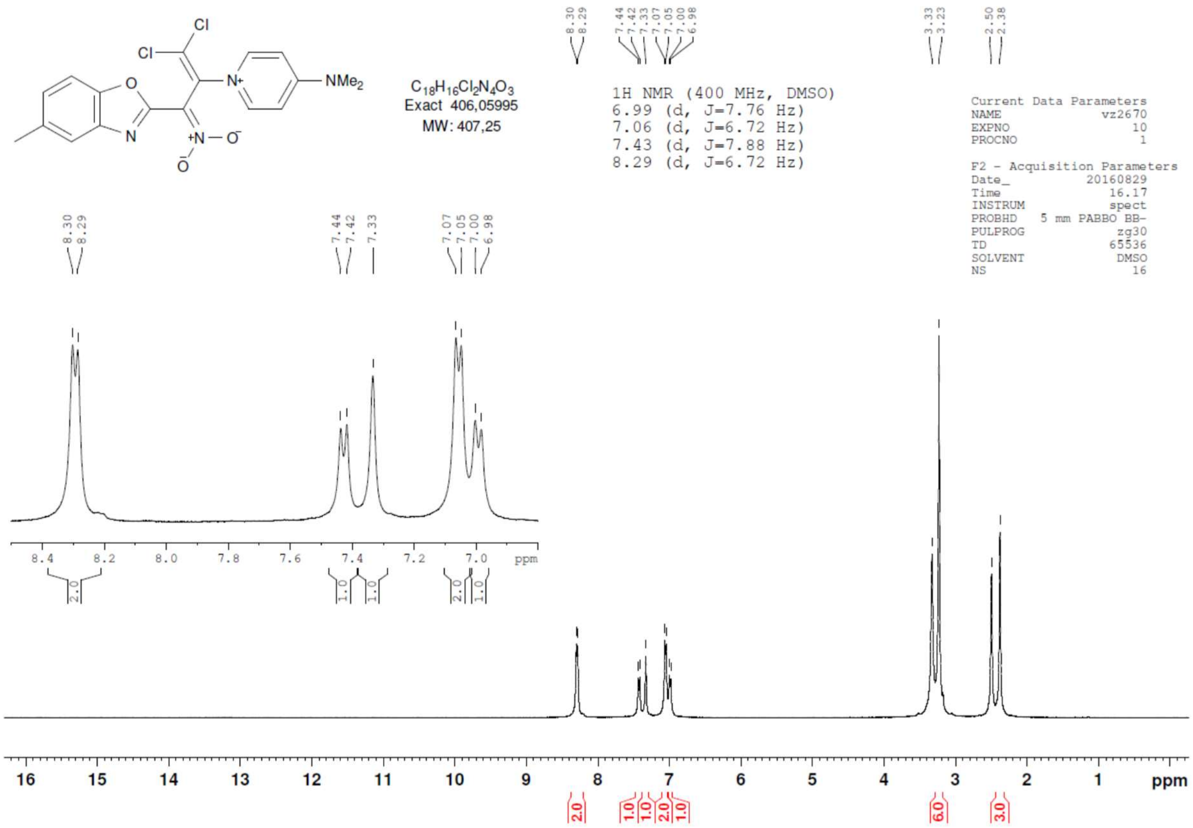

Figure S9. 100 MHz ^{13}C-NMR spectrum in DMSO-*d6* for **5a**.

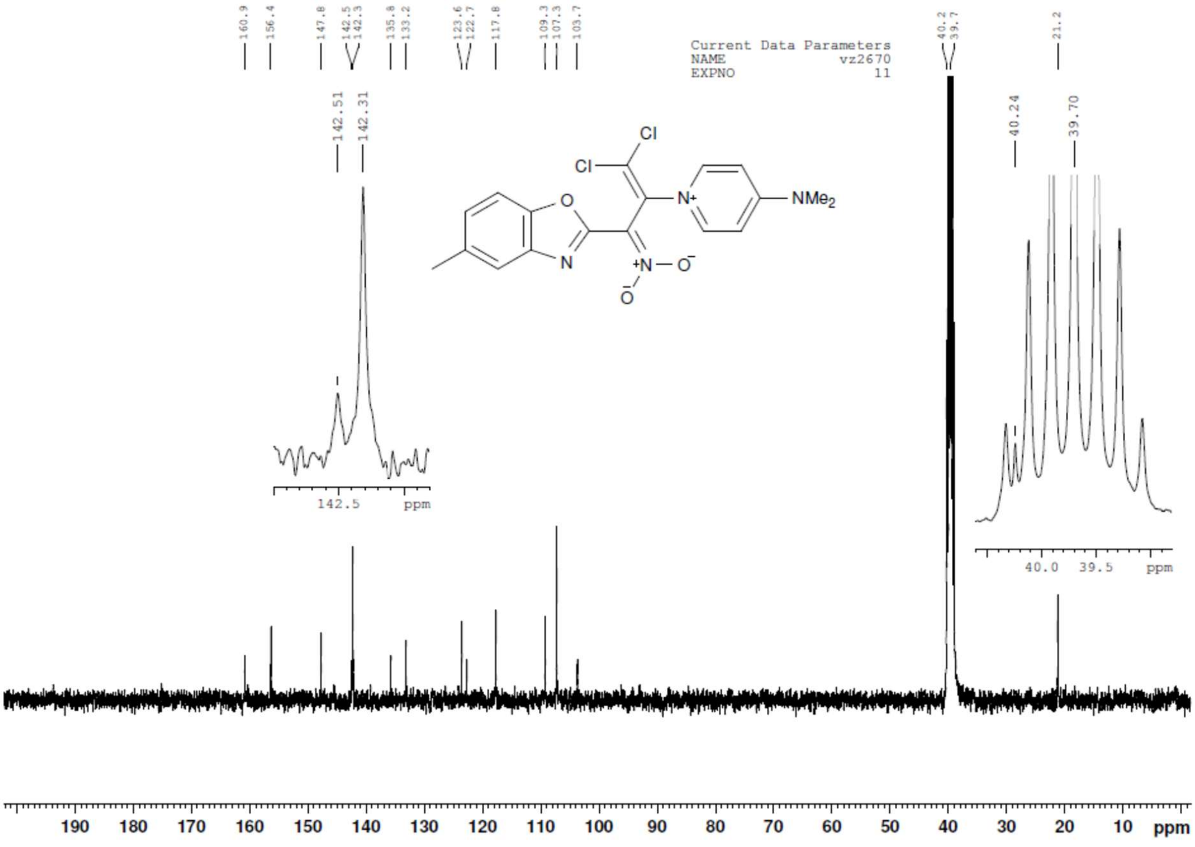

Figure S9. 400 MHz ^{1}H-NMR spectrum in DMSO-*d6* for **5b**.

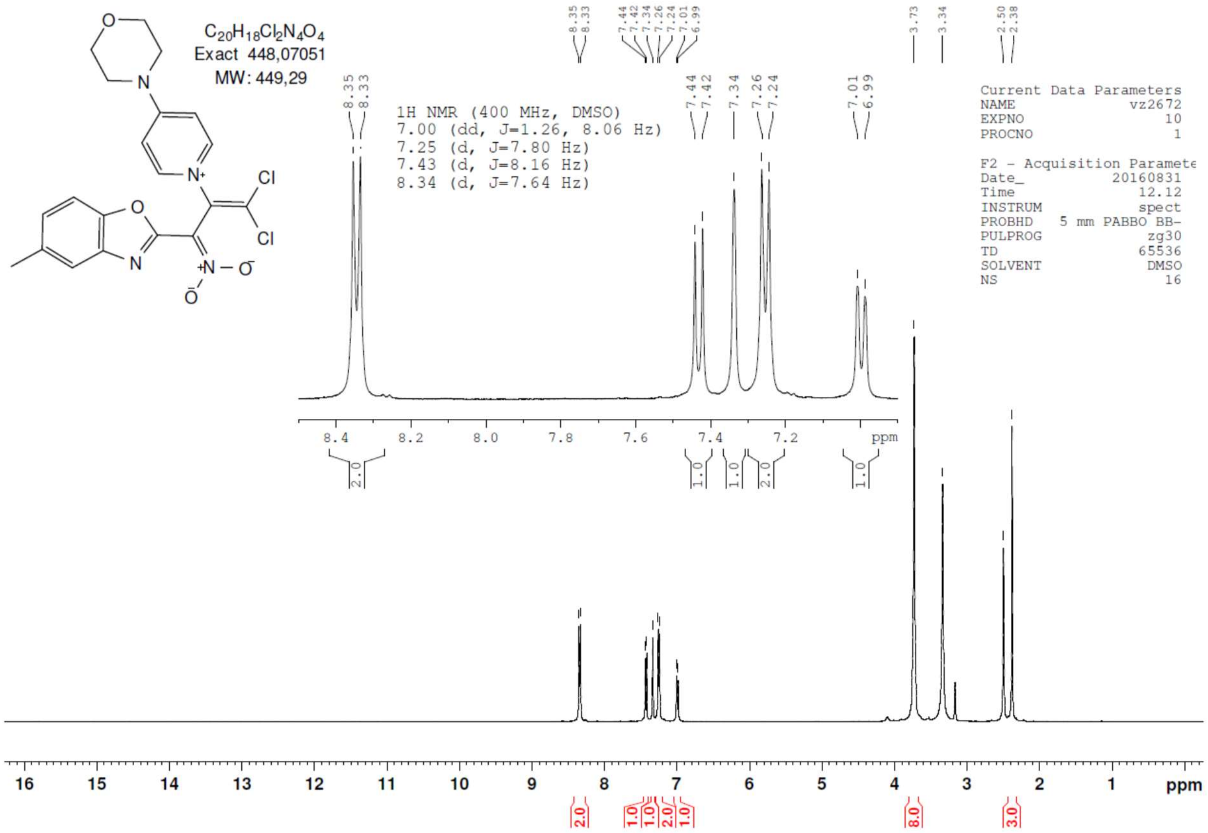

Figure S10. 100 MHz ^{13}C-NMR spectrum in DMSO-*d6* for **5b**.

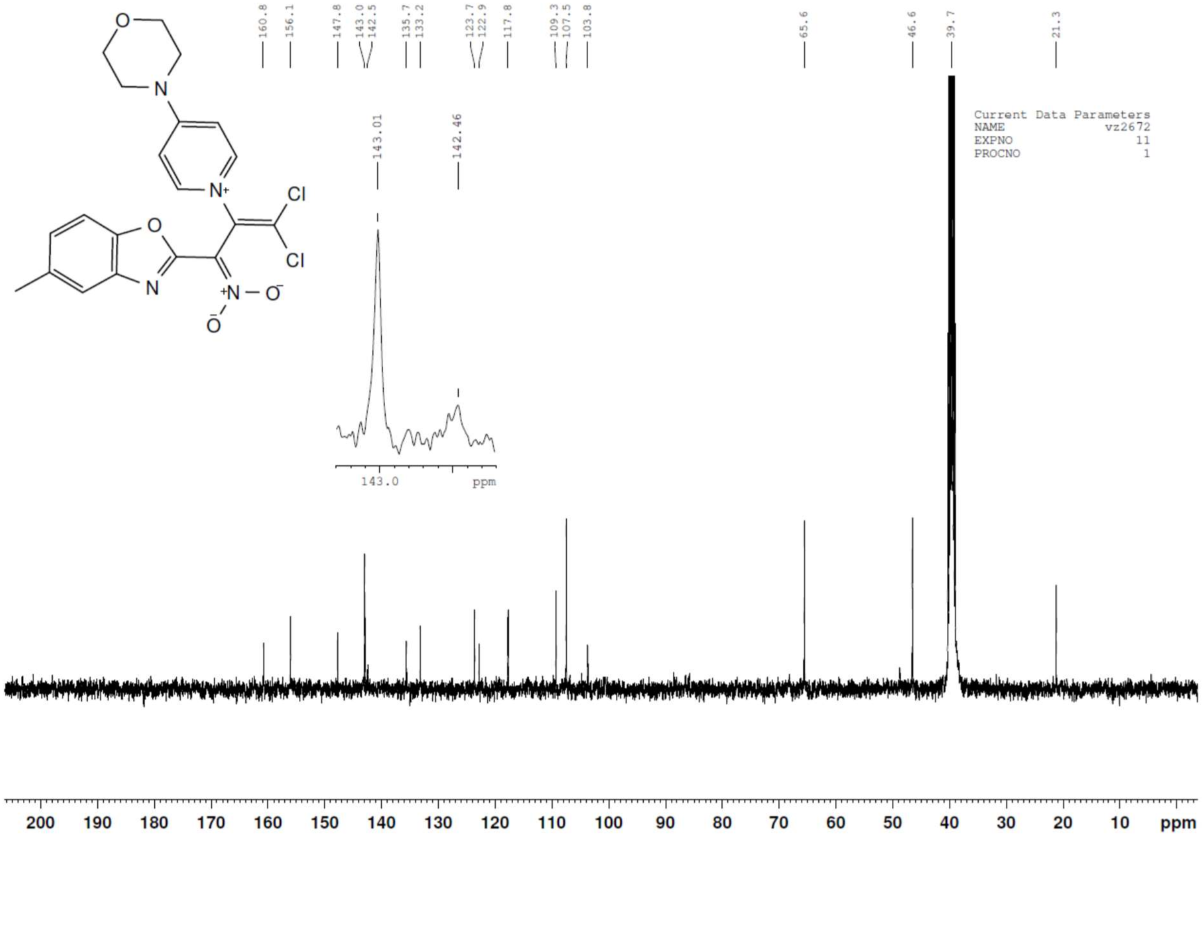

Figure S11. 600 MHz ^{1}H-NMR spectrum in DMSO-*d6* for **5c**.

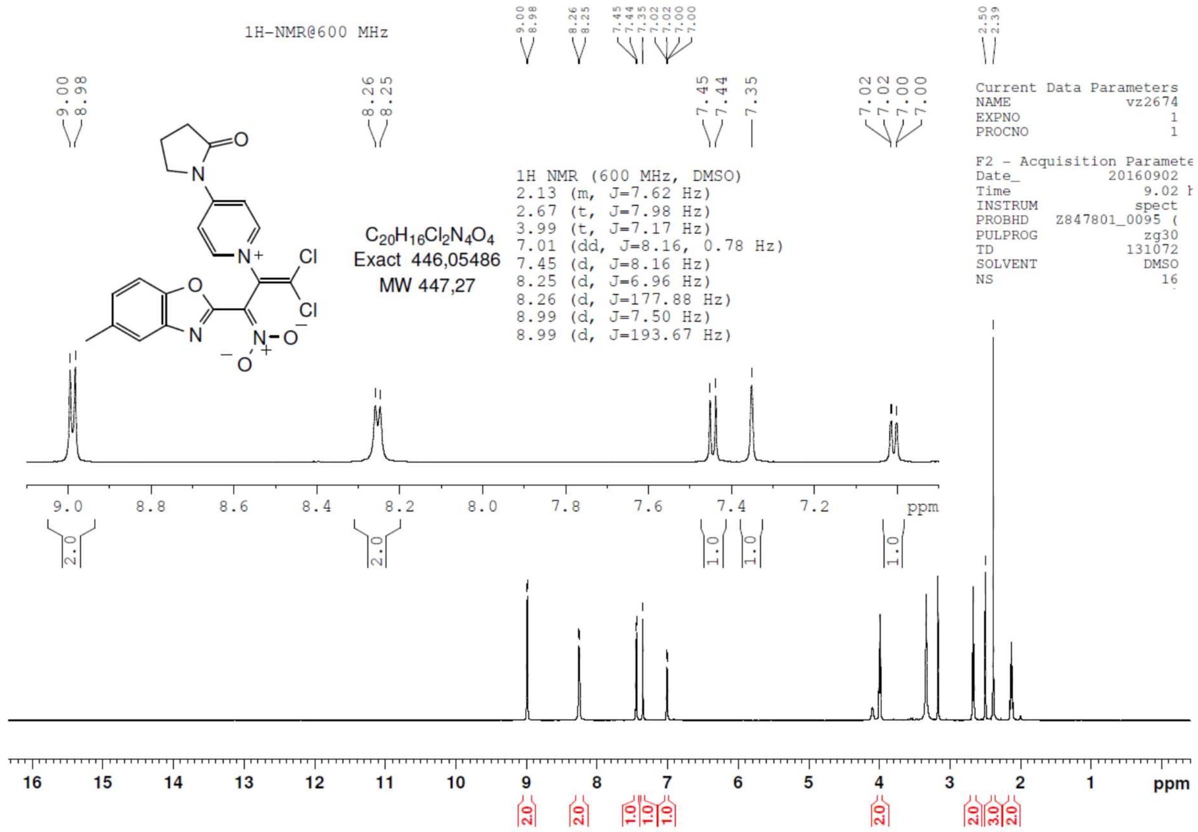

Figure S12. 150 MHz ^{13}C-NMR spectrum in DMSO-*d6* for **5c**.

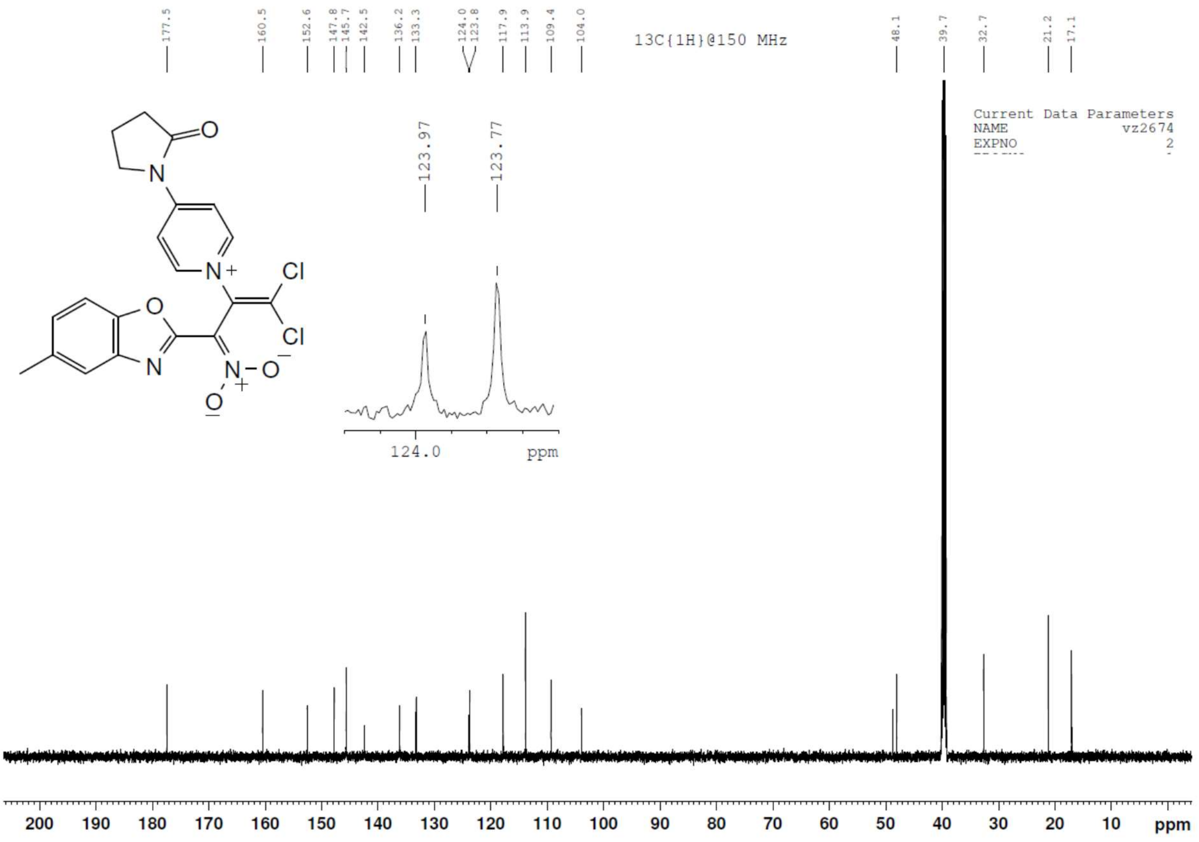

Figure S13. HMBC-NMR spectrum in DMSO-*d6* for **5c**.

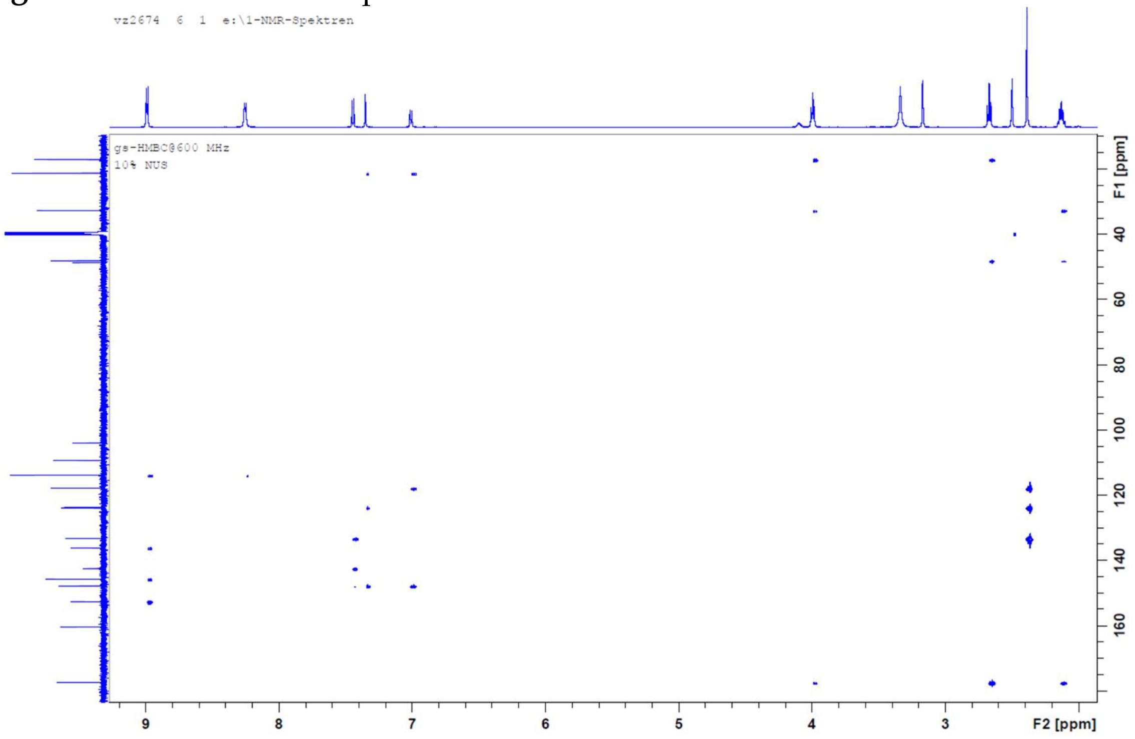

Figure S14. ^{15}N,^{1}H-HMBC-NMR spectrum in DMSO-*d6* for **5c**.

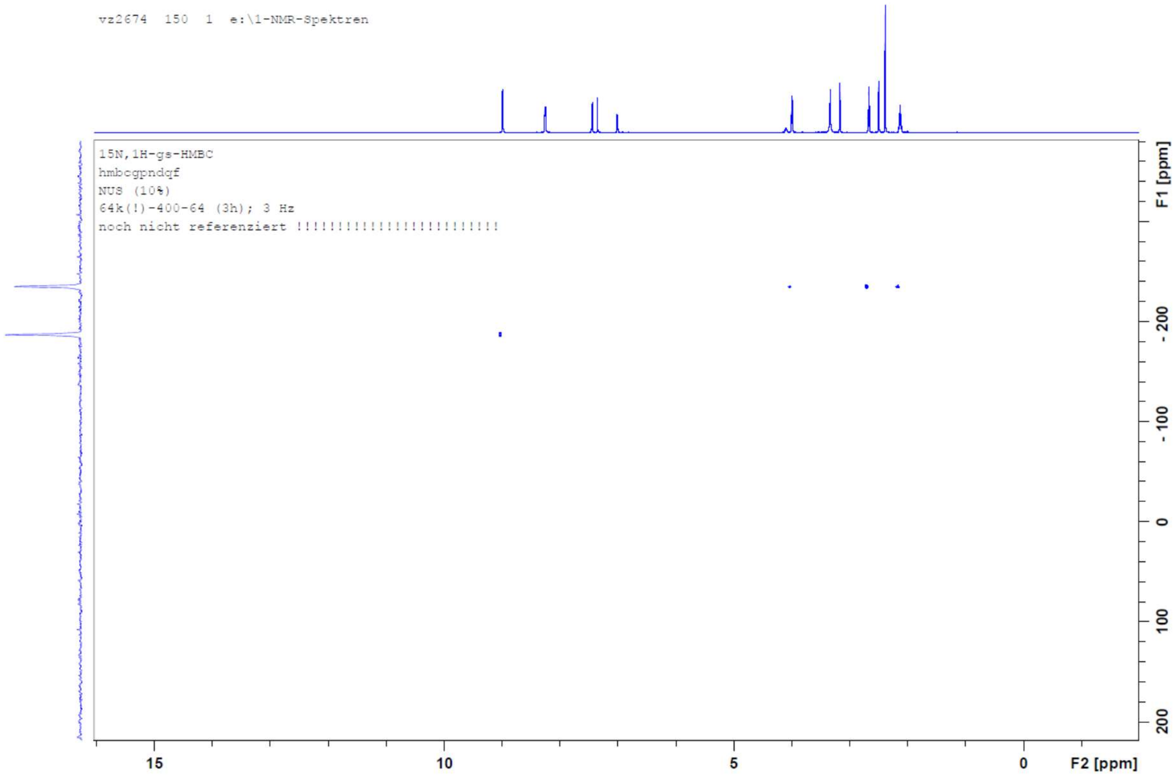

Figure S15. 200 MHz ^{1}H-NMR spectrum in $CDCl_3$ for **6**.

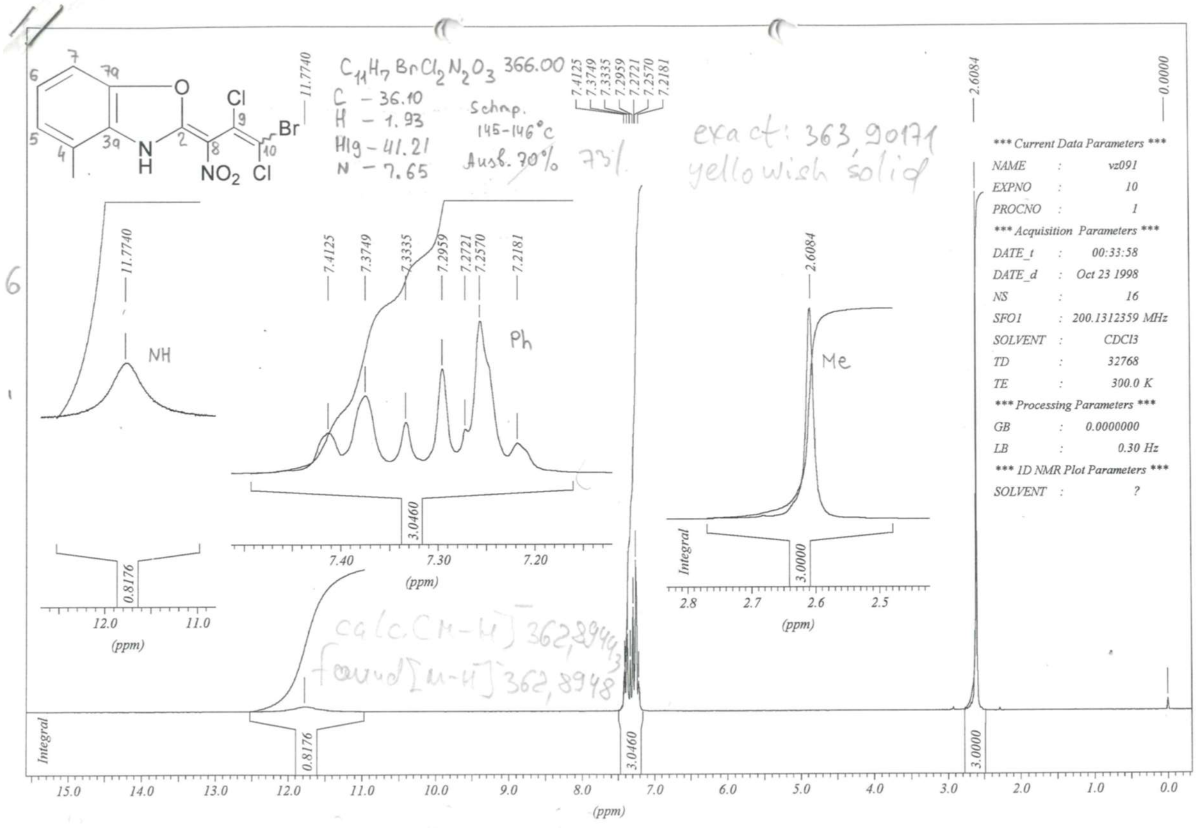

Figure S16. 50 MHz ^{13}C-NMR spectrum in $CDCl_3$ for **6**.

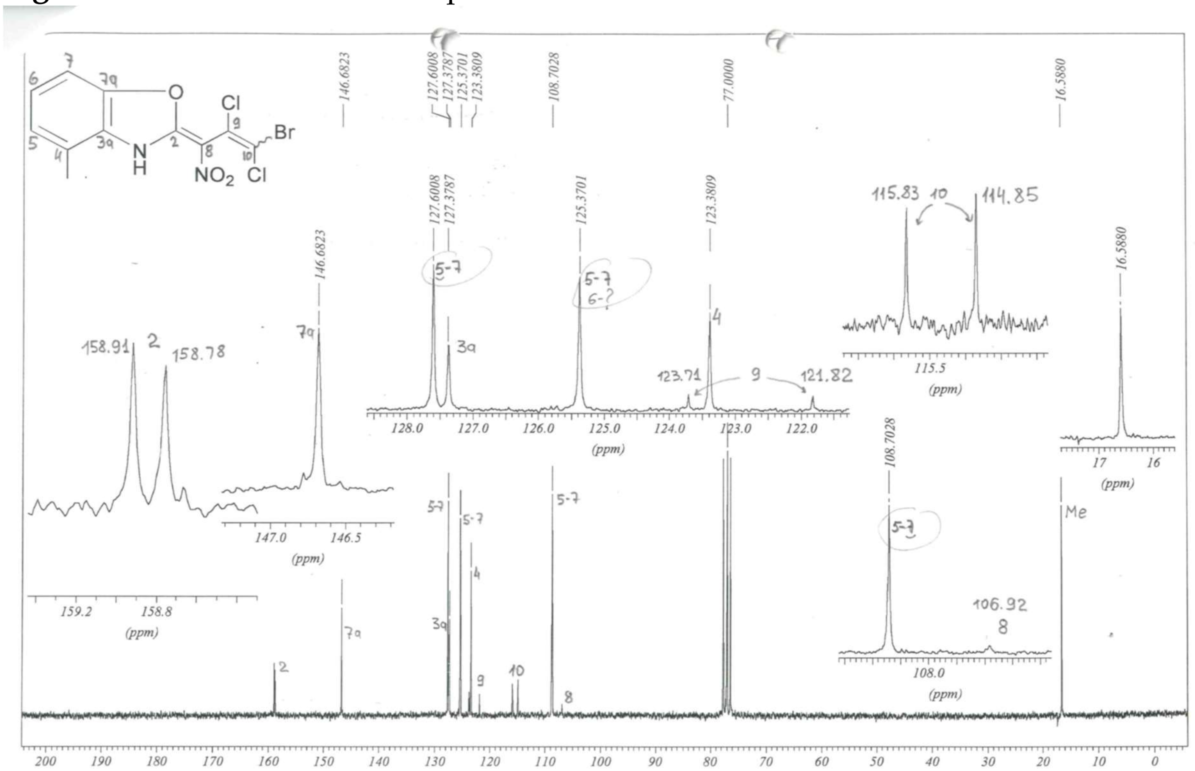

Figure S17. Mass spectrum for 2-[3-bromo-2,3-dichloro-1-nitroprop-2-en-1-ylidene]-4-methyl-2,3-dihydro-1,3-benzoxazole (**6**).

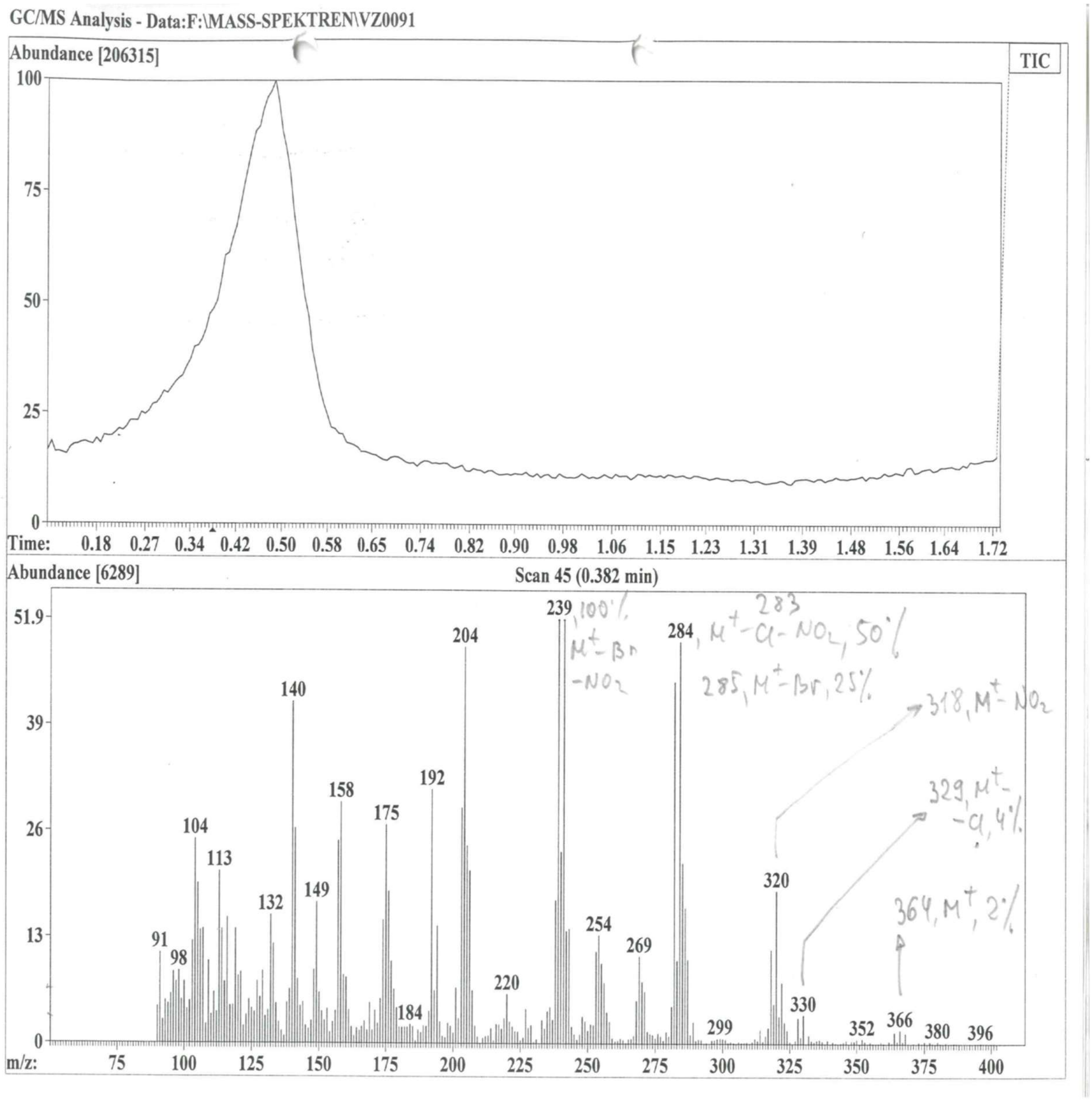

Figure S18. 200 MHz ^{1}H-NMR spectrum in DMSO-*d6* for 2-((Z)-3-chloro-1,3-dinitroallylidene)-5-methyl-2,3-dihydro-1*H*-benzo[*d*]imidazole (**7a**).

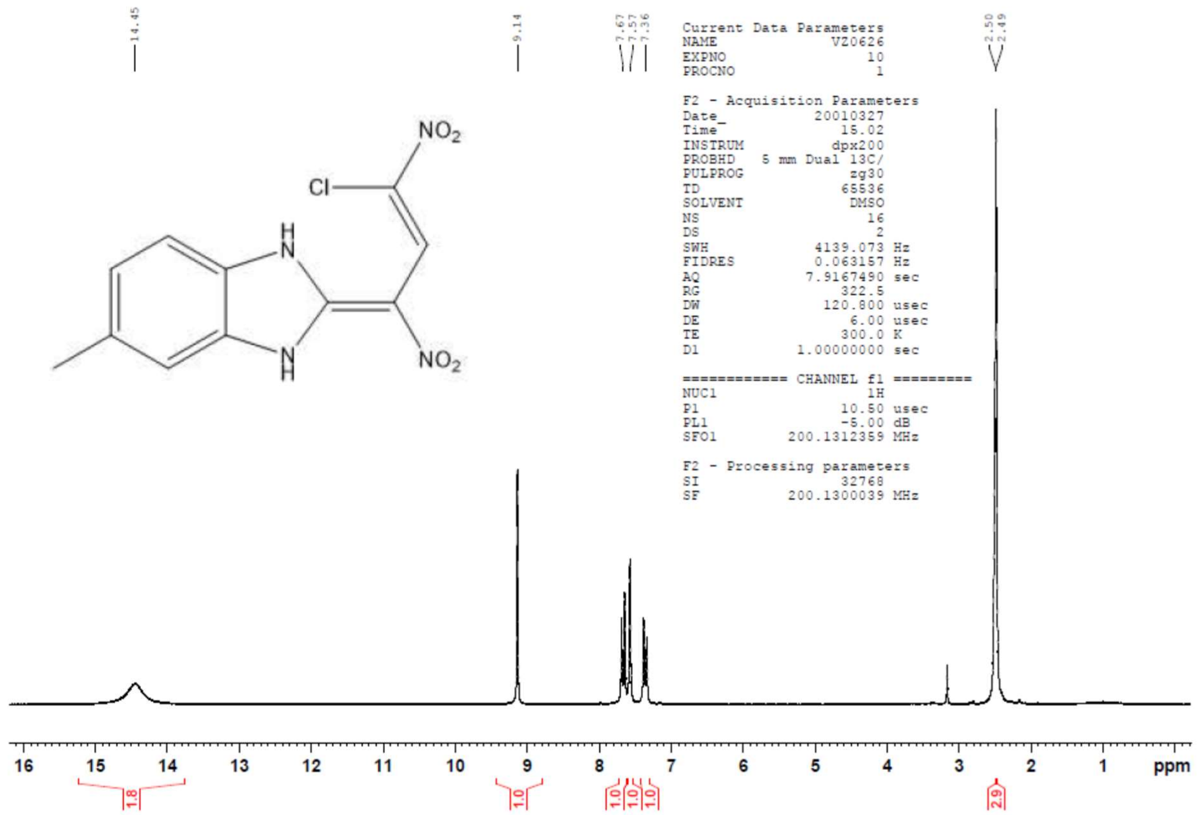

Figure S19. 50 MHz ^{13}C-NMR spectrum in DMSO-*d6* for 2-((Z)-3-chloro-1,3-dinitroallylidene)-5-methyl-2,3-dihydro-1*H*-benzo[*d*]imidazole (**7a**).

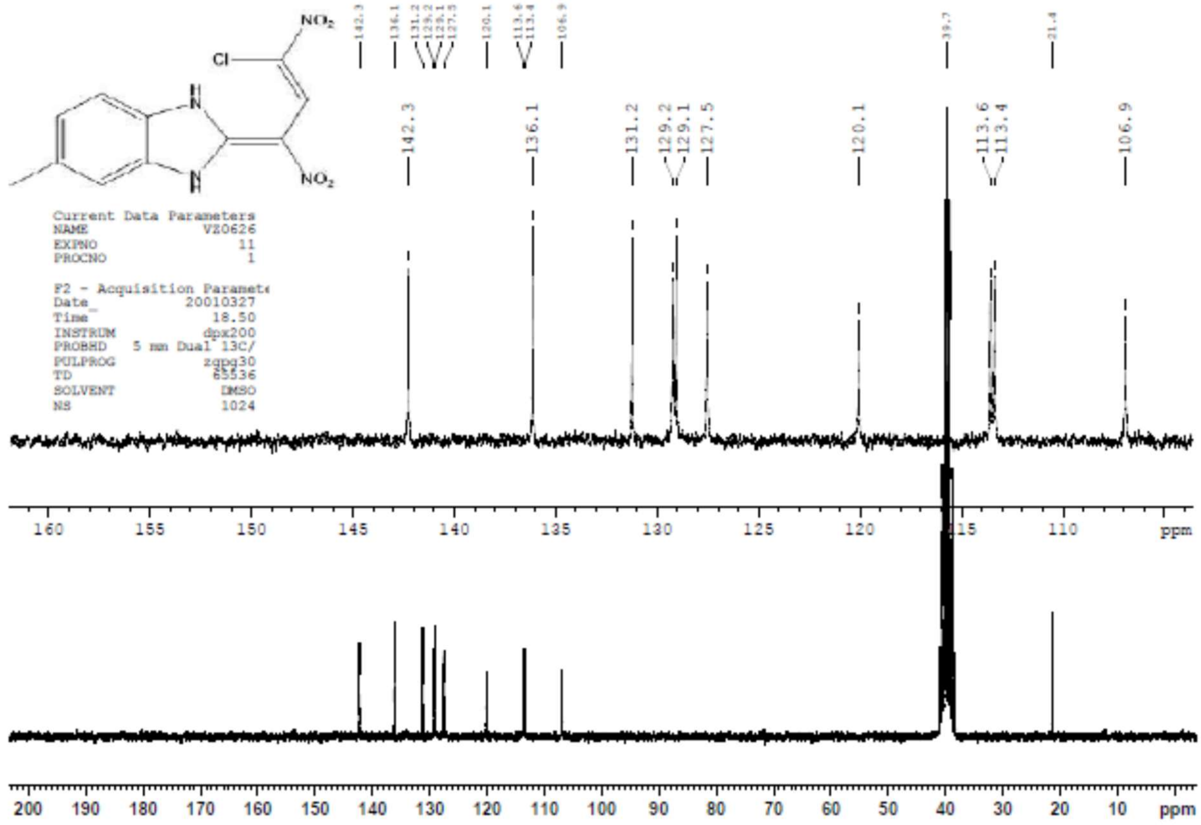

Figure S20. 200 MHz ^{1}H-NMR spectrum in DMSO-*d6* for *2-(3-Chloro-1,3-dinitroprop-2-en-1-ylidene)-5-methyl-2,3-dihydro-1,3-benzoxazole* (**7b**).

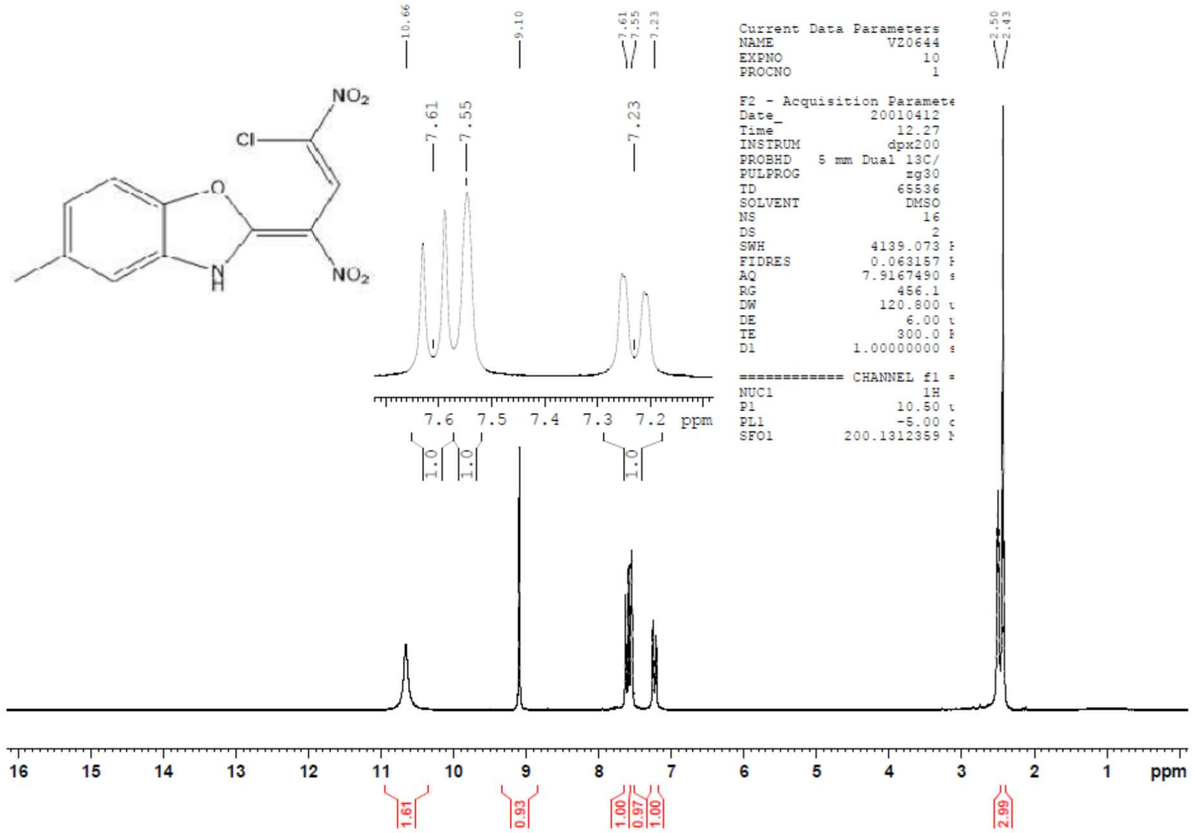

Figure S21. 50 MHz ^{13}C-NMR spectrum in DMSO-*d6* for *2-(3-Chloro-1,3-dinitroprop-2-en-1-ylidene)-5-methyl-2,3-dihydro-1,3-benzoxazole* (**7b**).

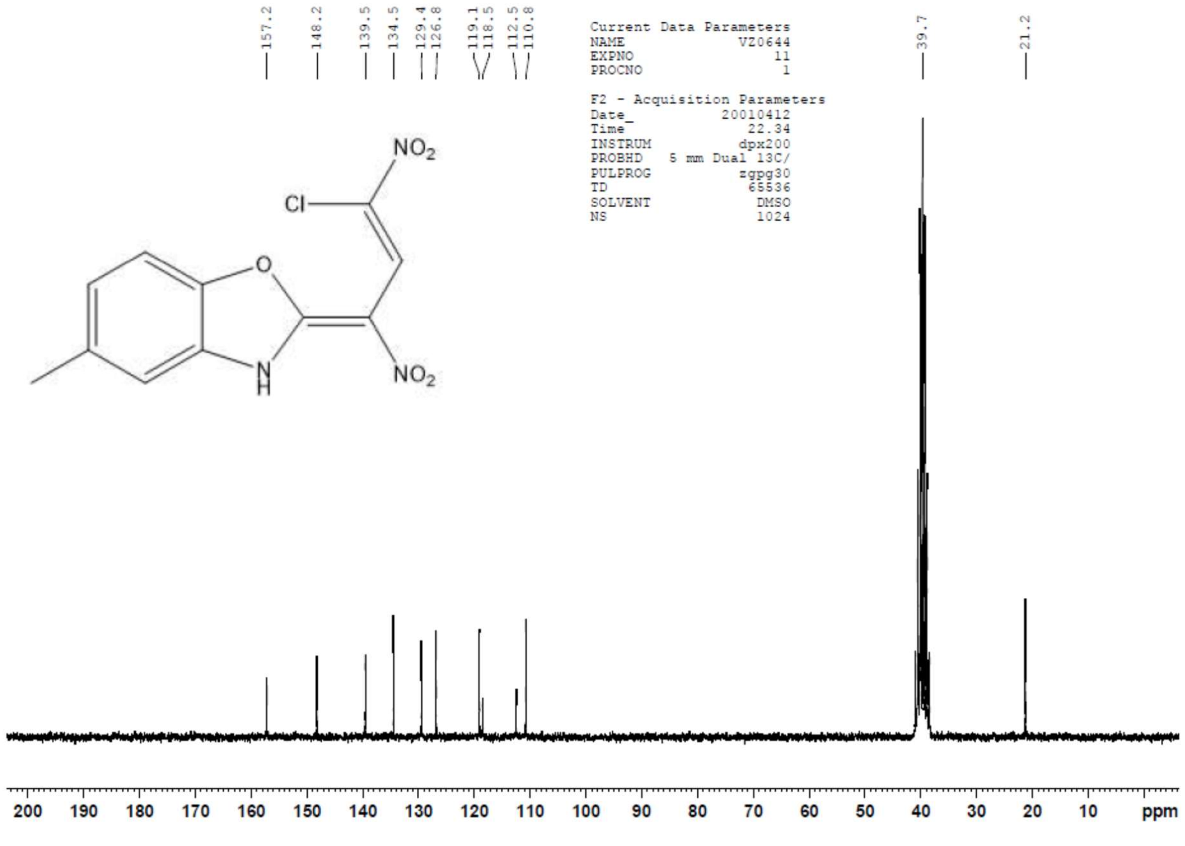

Figure S22. 200 MHz ^{1}H-NMR spectrum in DMSO-*d6* for **7c**.

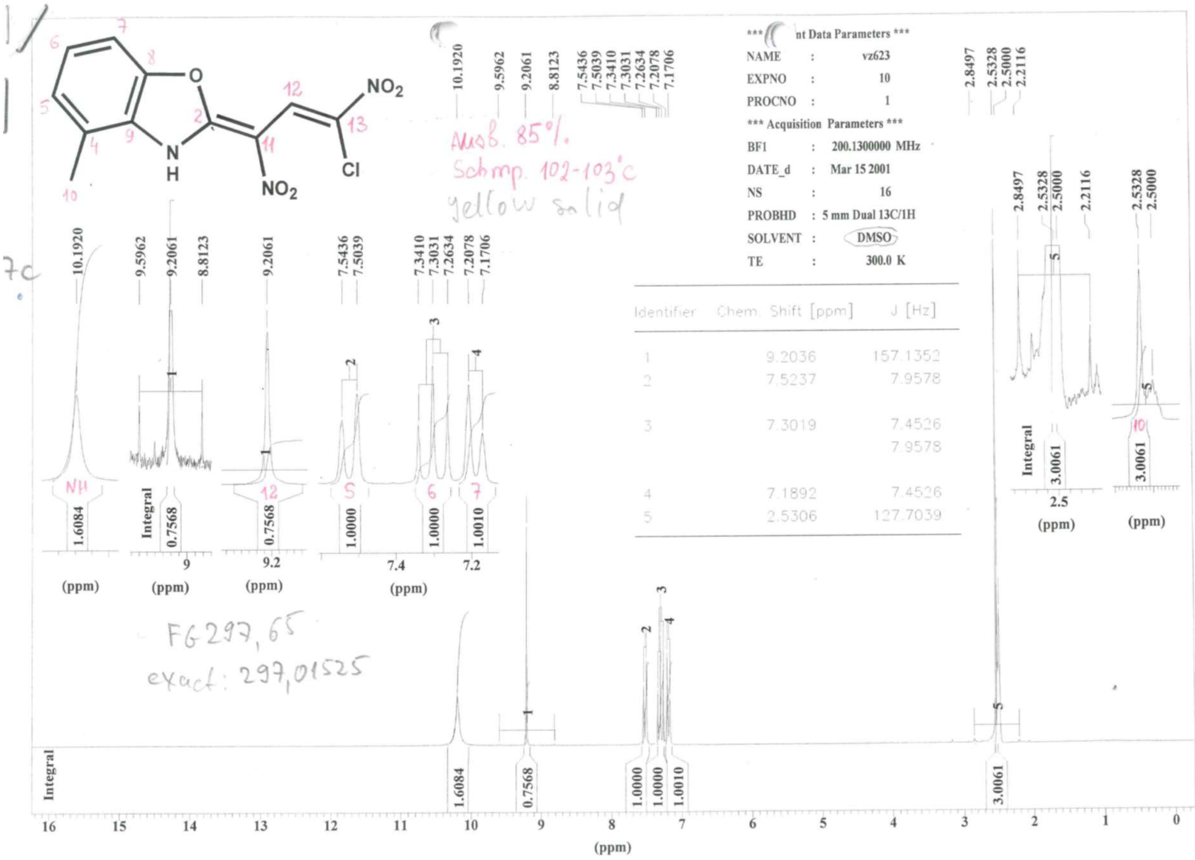

Figure S23. 50 MHz ^{13}C-NMR spectrum in DMSO-*d6* for **7c**.

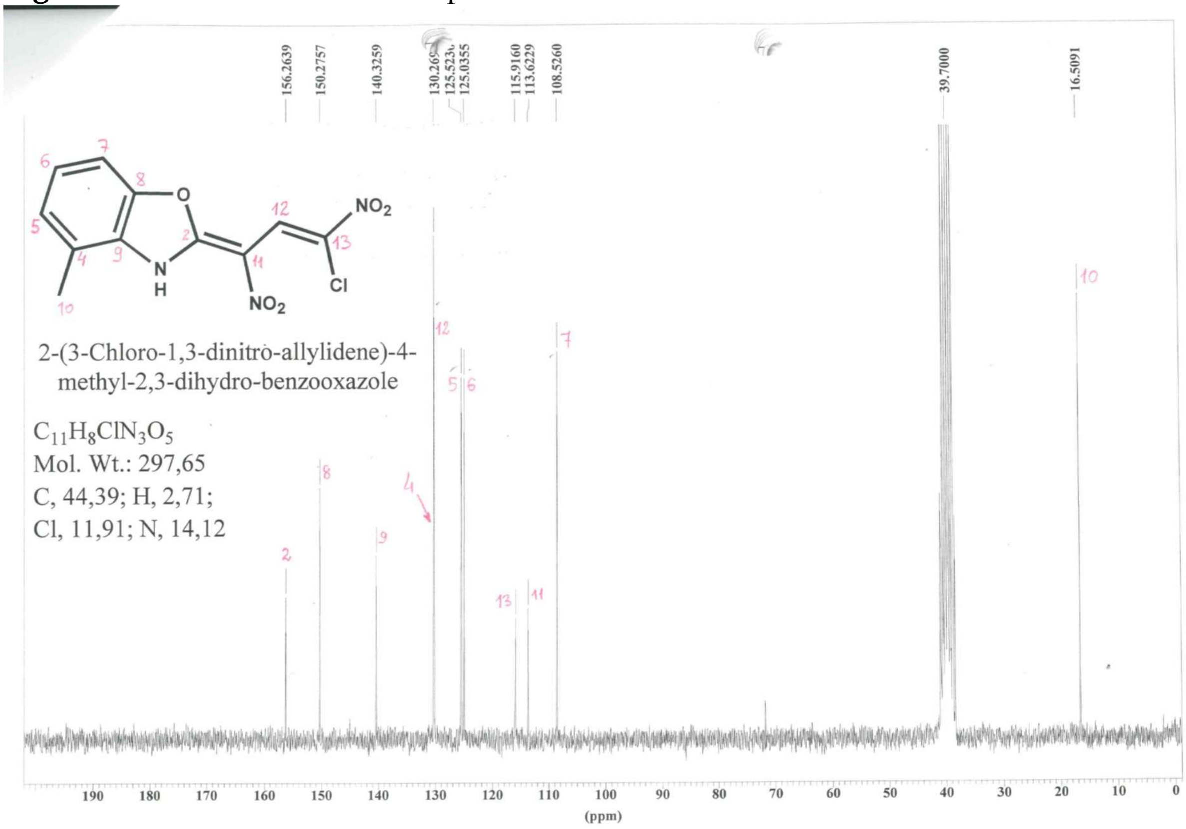

Figure S24. Mass spectrum for 2-(3-chloro-1,3-dinitroprop-2-en-1-ylidene)-4-methyl-2,3-dihydro-1,3-benzoxazole (**7c**).

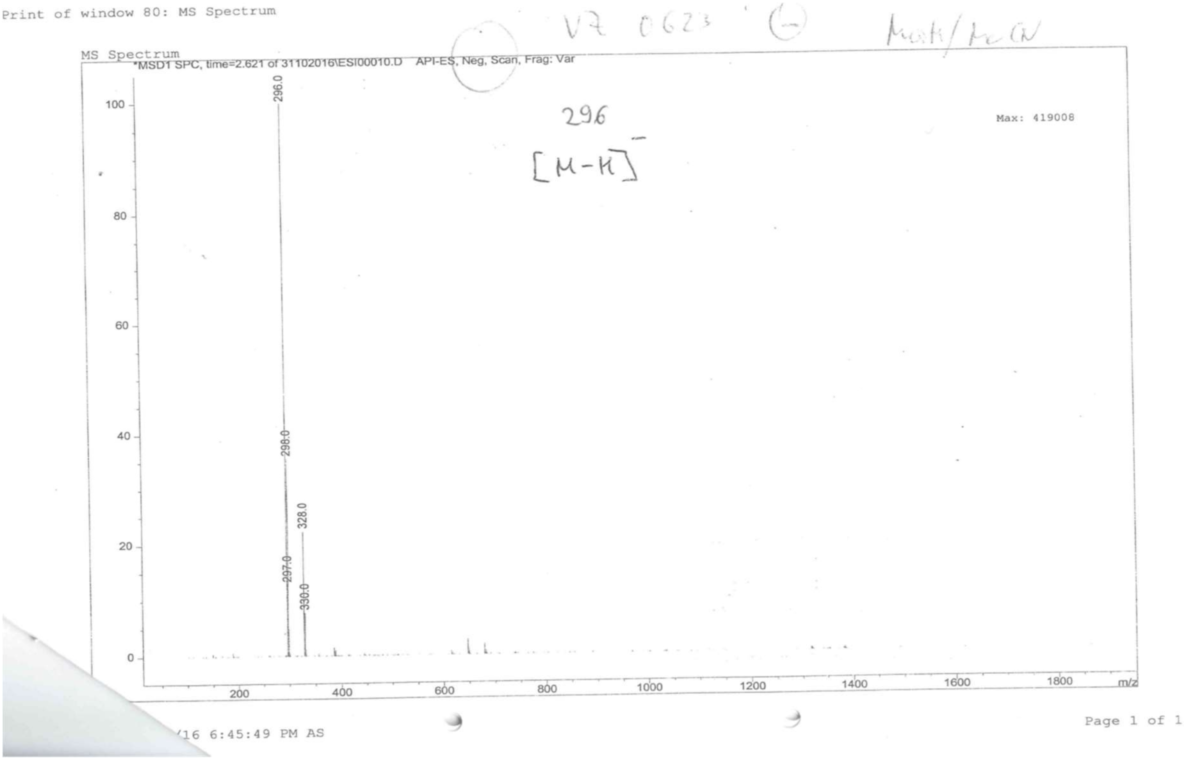

Figure S25. HR-MS spectrum for 2-(3-chloro-1,3-dinitroprop-2-en-1-ylidene)-4-methyl-2,3-dihydro-1,3-benzoxazole (**7c**).

Page 1

…nental Composition Report

Single Mass Analysis (displaying only valid results)
Tolerance = 10.0 PPM / DBE: min = -1.5, max = 50.0
Selected filters: None

Monoisotopic Mass, Even Electron Ions
720 formula(e) evaluated with 9 results within limits (up to 80 closest results for each mass)
Elements Used:
C: 0-50 H: 0-60 N: 0-3 O: 0-7 Na: 0-1 Cl: 0-3

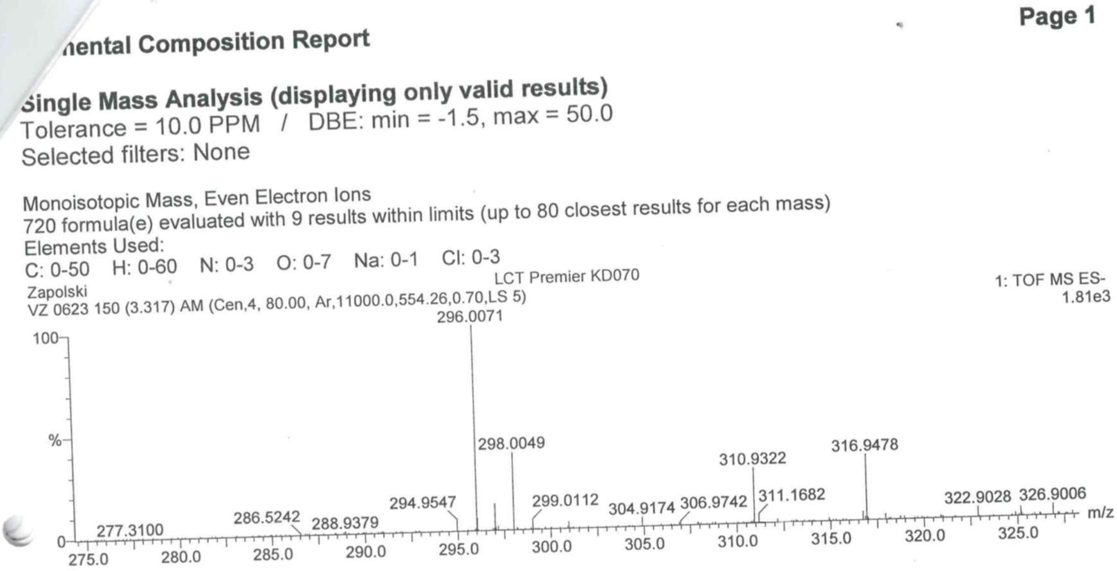

Minimum:				-1.5		
Maximum:		5.0	10.0	50.0		
Mass	Calc. Mass	mDa	PPM	DBE	i-FIT	Formula
296.0071	296.0072	-0.1	-0.3	15.5	320.2	C15 H3 N3 O3 Na
	√296.0074	-0.3	-1.0	9.5	3.4	C11 H7 N3 O5 Cl
	296.0068	0.3	1.0	1.5	106.9	C8 H13 N O5 Na Cl2
	296.0090	-1.9	-6.4	10.5	11.8	C14 H8 N O3 Na Cl
	296.0052	1.9	6.4	0.5	125.9	C5 H12 N3 O7 Cl2
	296.0050	2.1	7.1	6.5	4.0	C9 H8 N3 O5 Na Cl
	296.0093	-2.2	-7.4	4.5	98.0	C10 H12 N O5 Cl2
	296.0096	-2.5	-8.4	18.5	330.4	C17 H2 N3 O3
	296.0100	-2.9	-9.8	1.5	276.2	C8 H14 N3 O Na Cl3

Figure S26. 200 MHz ^{1}H-NMR spectrum in DMSO-*d6* for **8b**.

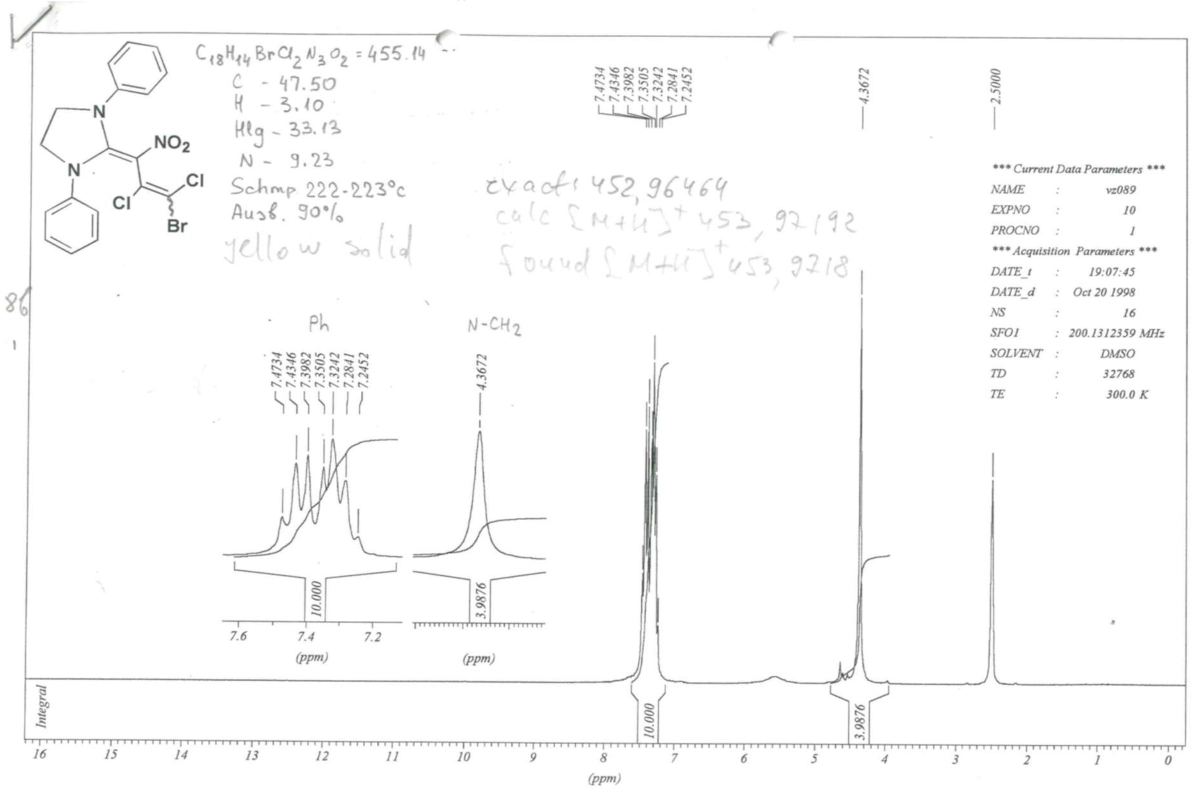

Figure S27. 50 MHz ^{13}C-NMR spectrum in DMSO-*d6* for **8b**.

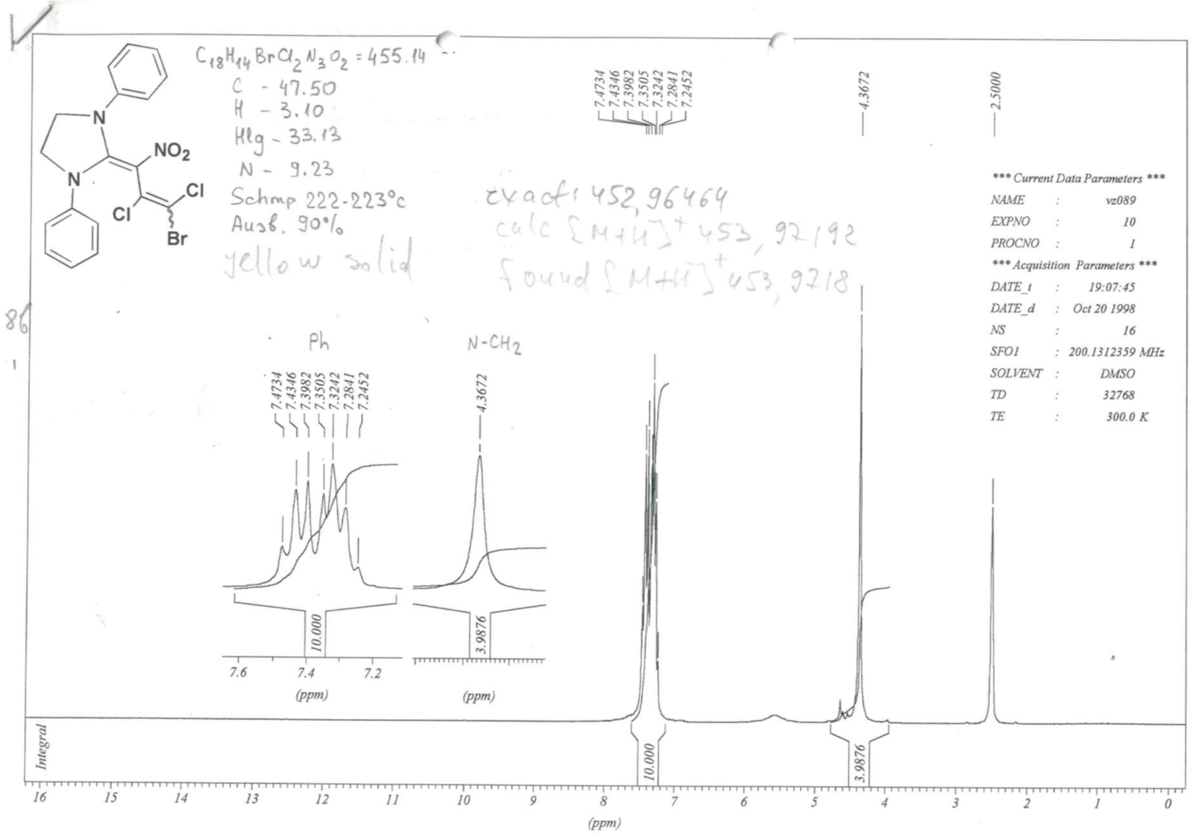

Figure S28. Mass spectrum for 2-(3-bromo-2,3-dichloro-1-nitroprop-2-en-1-ylidene)-1,3-diphenylimidazolidine (**8b**).

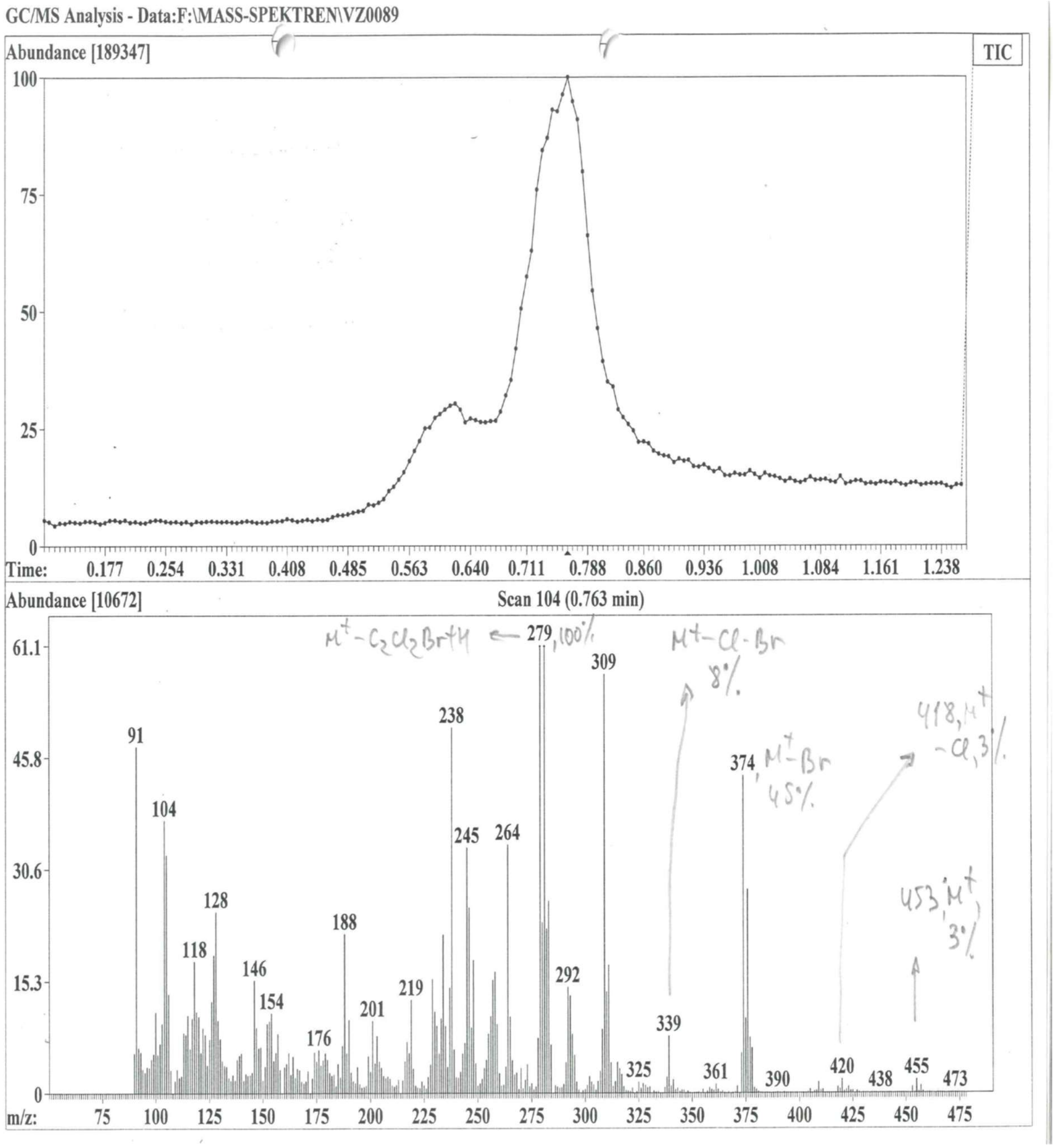

Figure S29. 200 MHz ^{1}H-NMR spectrum in DMSO-*d6* for **8c**.

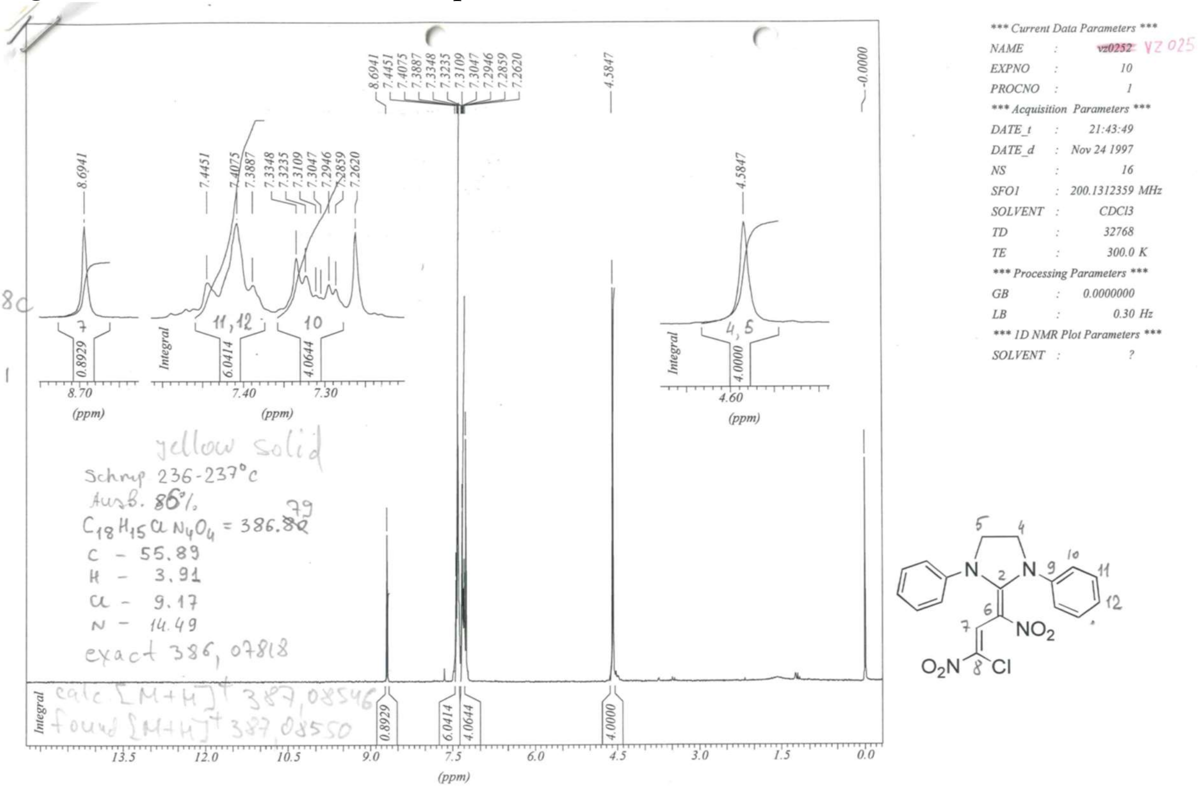

Figure S30. 50 MHz ^{13}C-NMR spectrum in DMSO-*d6* for **8c**.

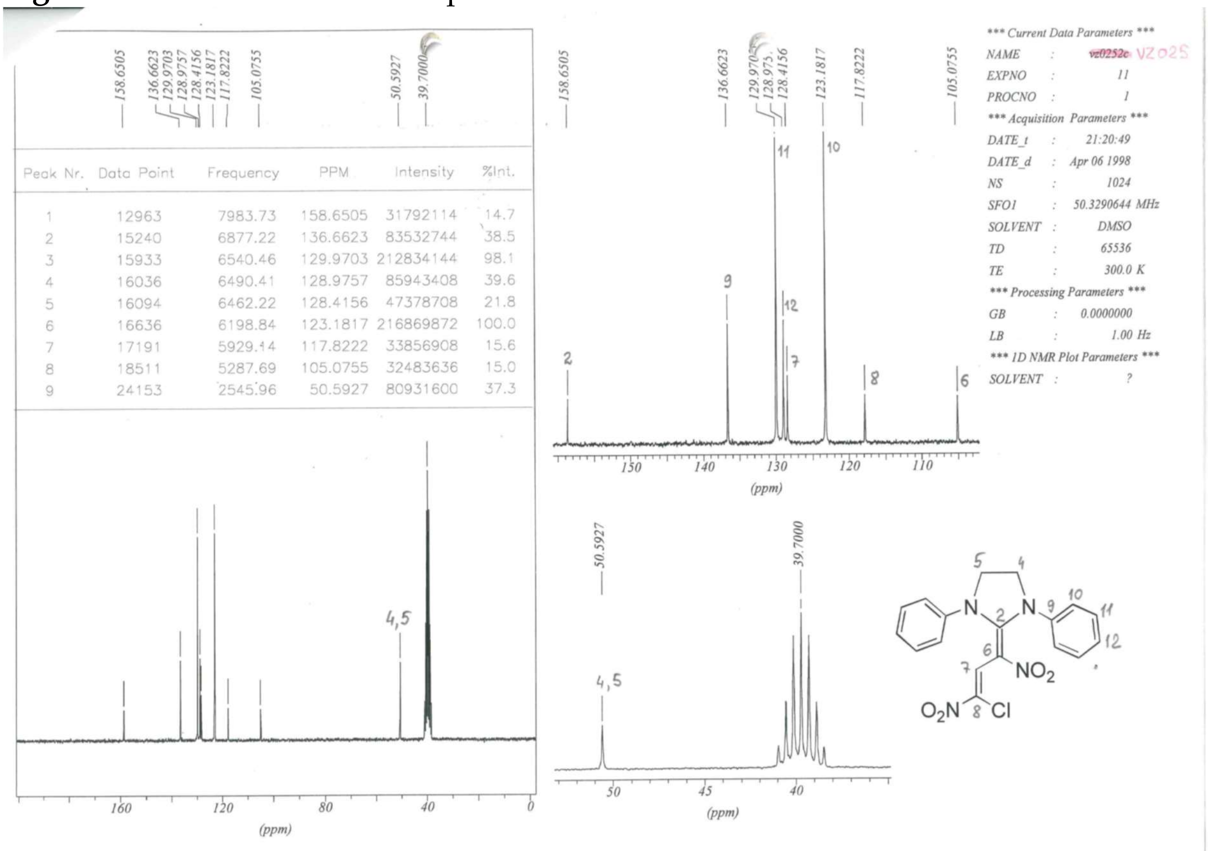

Peak Nr.	Data Point	Frequency	PPM	Intensity	%Int.
1	12963	7983.73	158.6505	31792114	14.7
2	15240	6877.22	136.6623	83532744	38.5
3	15933	6540.46	129.9703	212834144	98.1
4	16036	6490.41	128.9757	85943408	39.6
5	16094	6462.22	128.4156	47378708	21.8
6	16636	6198.84	123.1817	216869872	100.0
7	17191	5929.14	117.8222	33856908	15.6
8	18511	5287.69	105.0755	32483636	15.0
9	24153	2545.96	50.5927	80931600	37.3

Figure S31. Mass spectrum for 2-(3-chloro-1,3-dinitroprop-2-en-1-ylidene)-1,3-diphenylimidazolidine (**8c**).

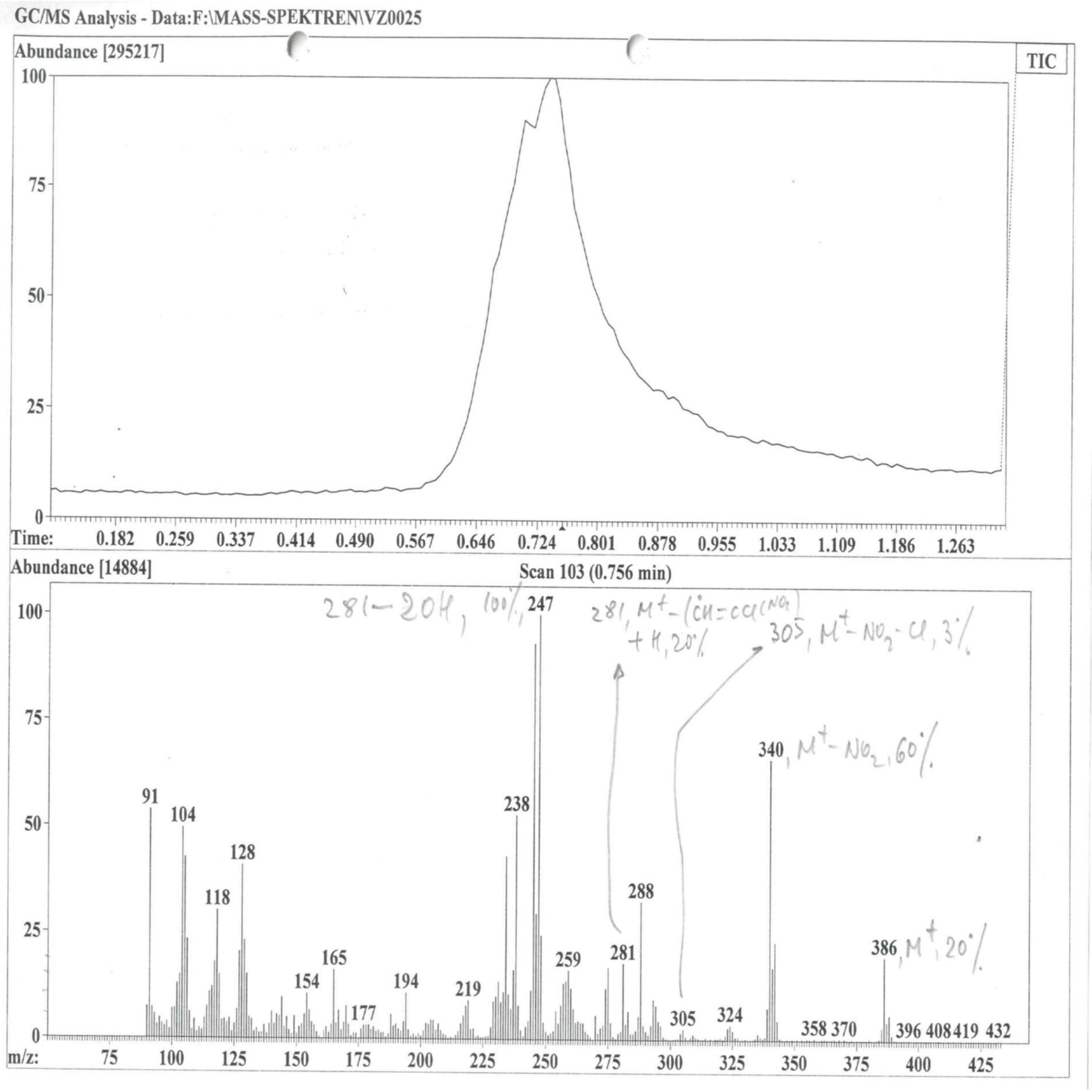

Figure S32. 200 MHz ^{1}H-NMR spectrum in DMSO-*d6* for **9**.

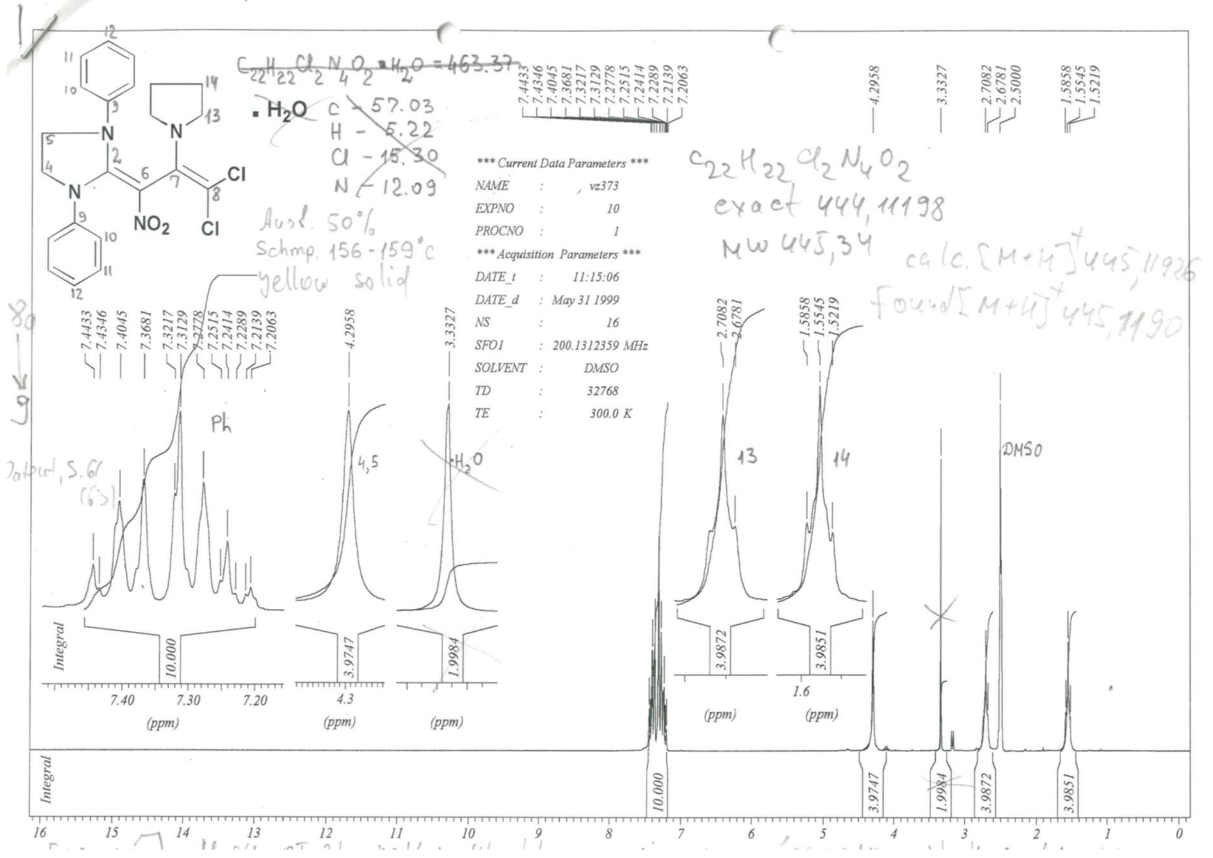

Figure S33. 50 MHz ^{13}C-NMR spectrum in DMSO-*d6* for **9**.

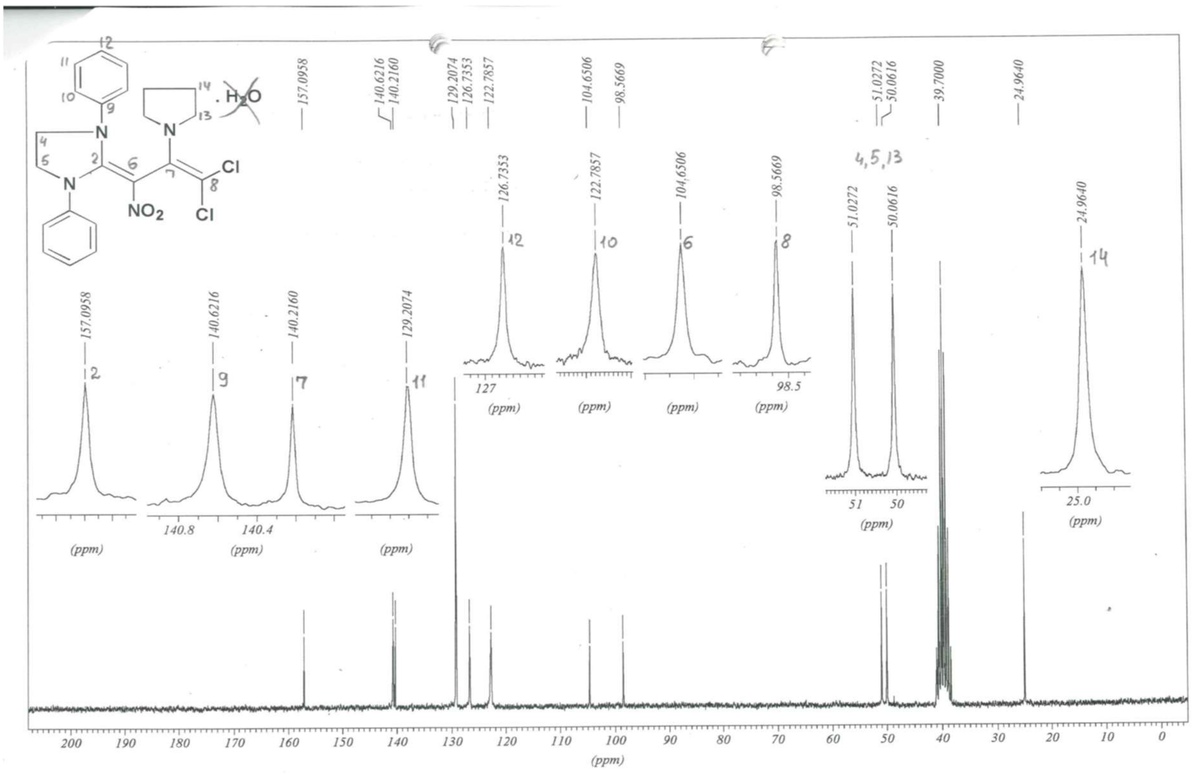

Figure S34. Mass spectrum for 2-[3,3-dichloro-2-nitro-1-(pyrrolidin-1-yl)prop-2-en-1-ylidene]-1,3-diphenylimidazolidine (**9**).

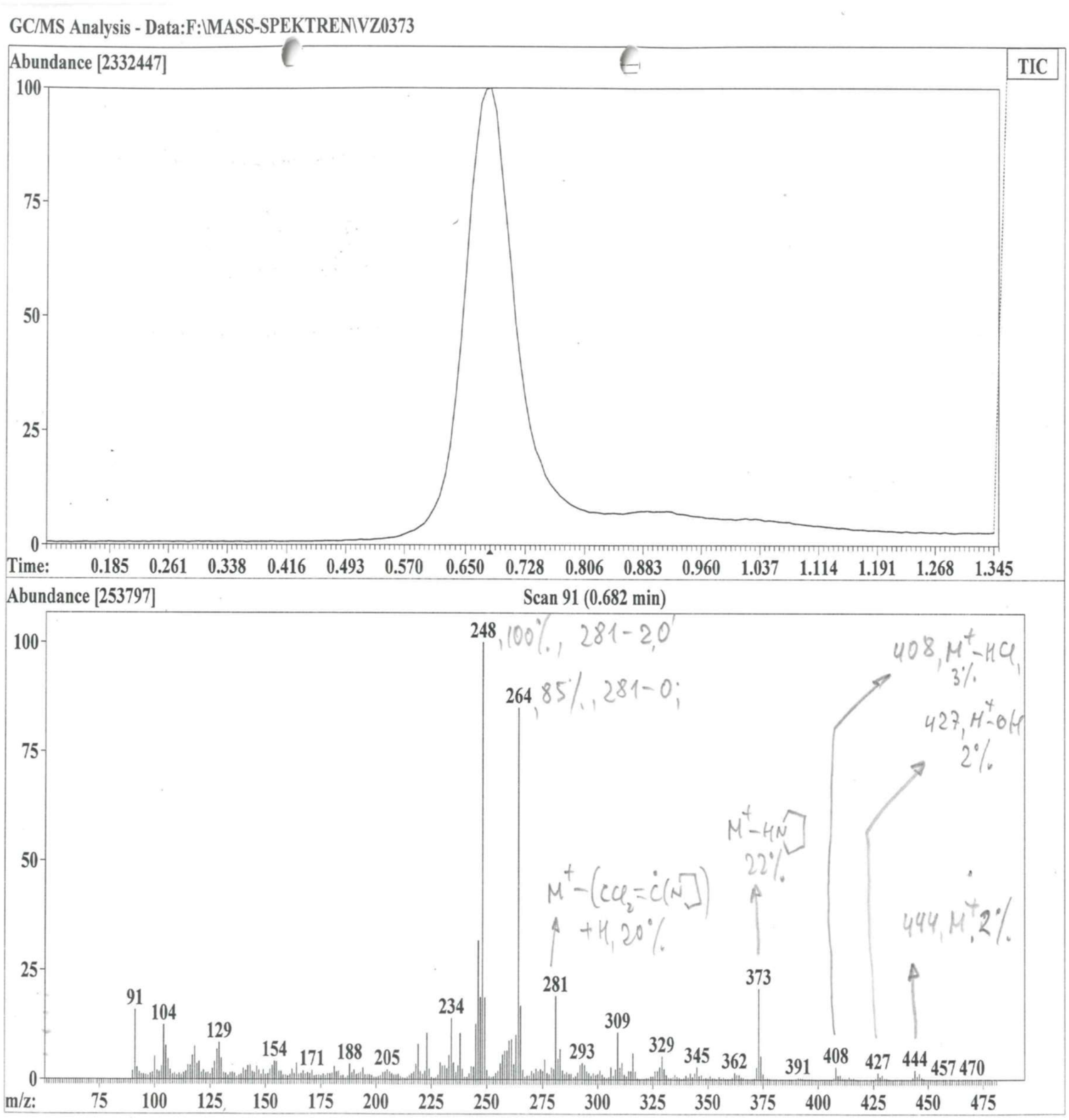

Figure S35. 200 MHz ^{1}H-NMR spectrum in DMSO-*d6* for **12a**.

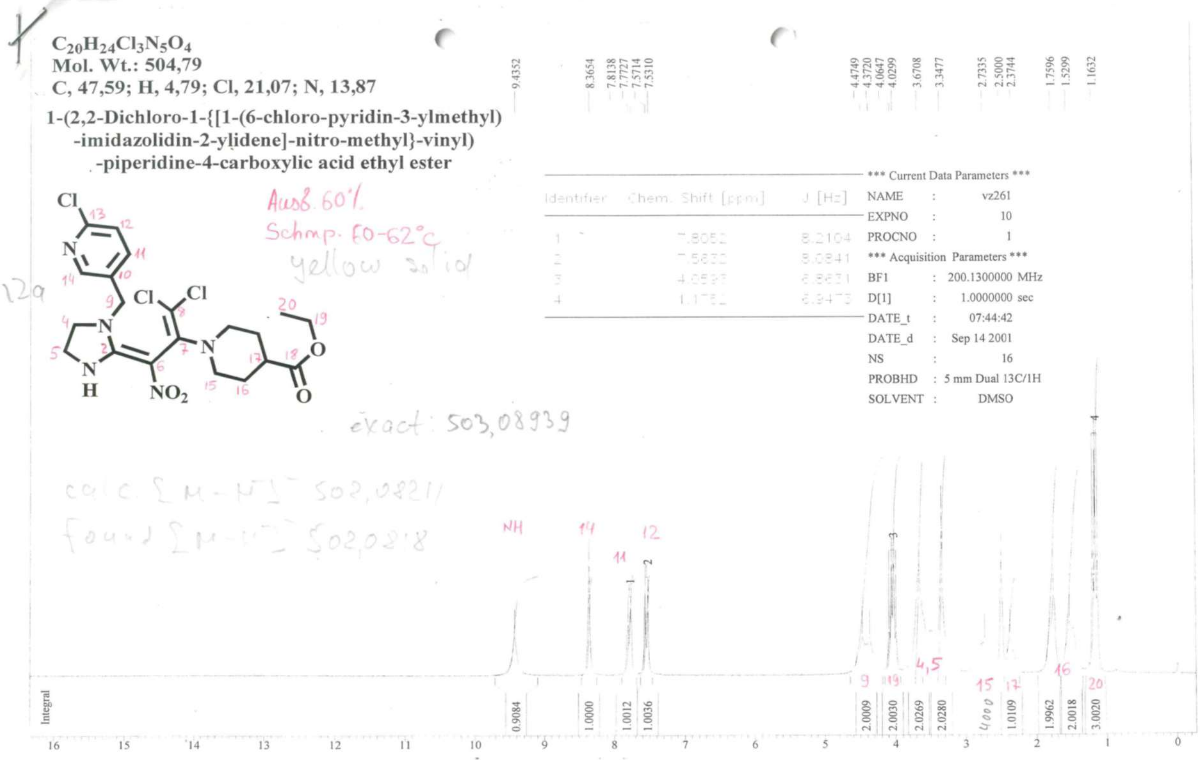

Figure S36. 50 MHz ^{13}C-NMR spectrum in DMSO-*d6* for **12a**.

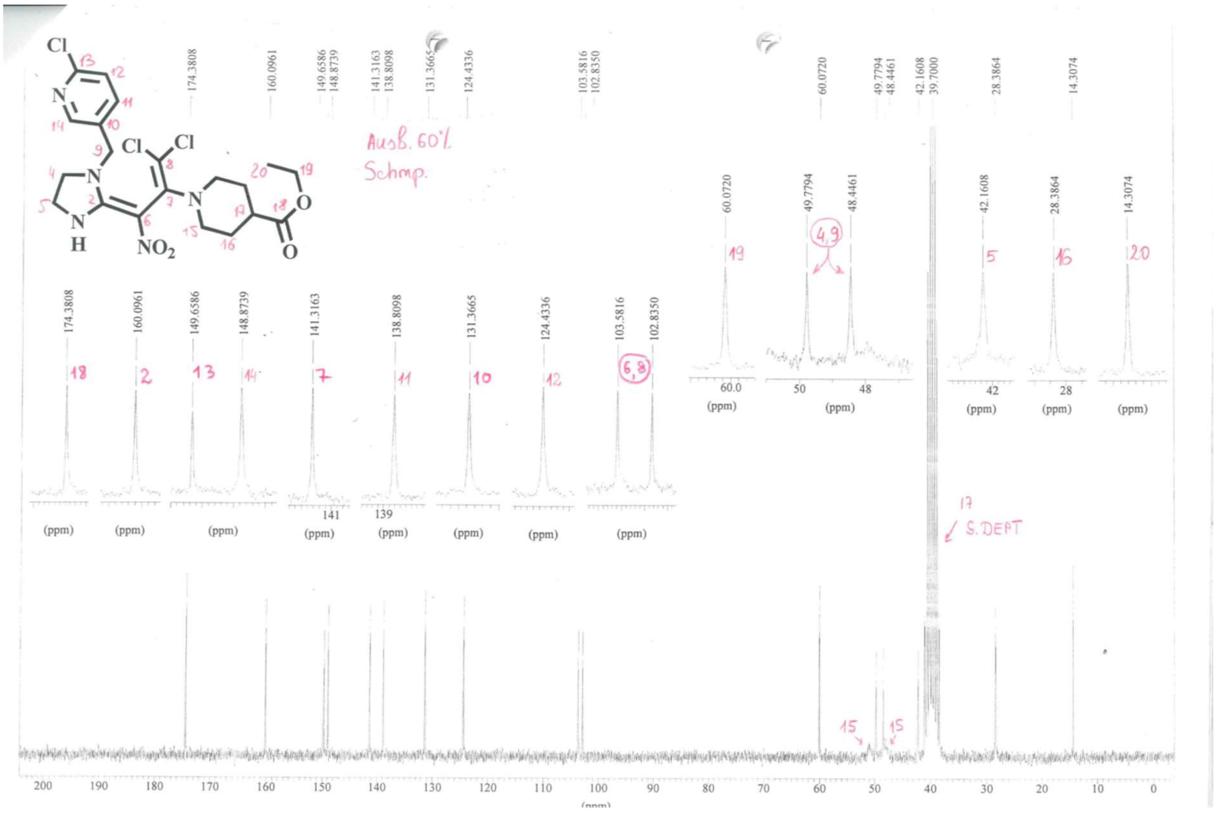

Figure S37. Mass spectrum for ethyl 1-(1,1-dichloro-3-{1-[(6-chloropyridin-3-yl)methyl]imidazolidin-2-ylidene}-3-nitroprop-1-en-2-yl)piperidine-4-carboxylate (**12a**).

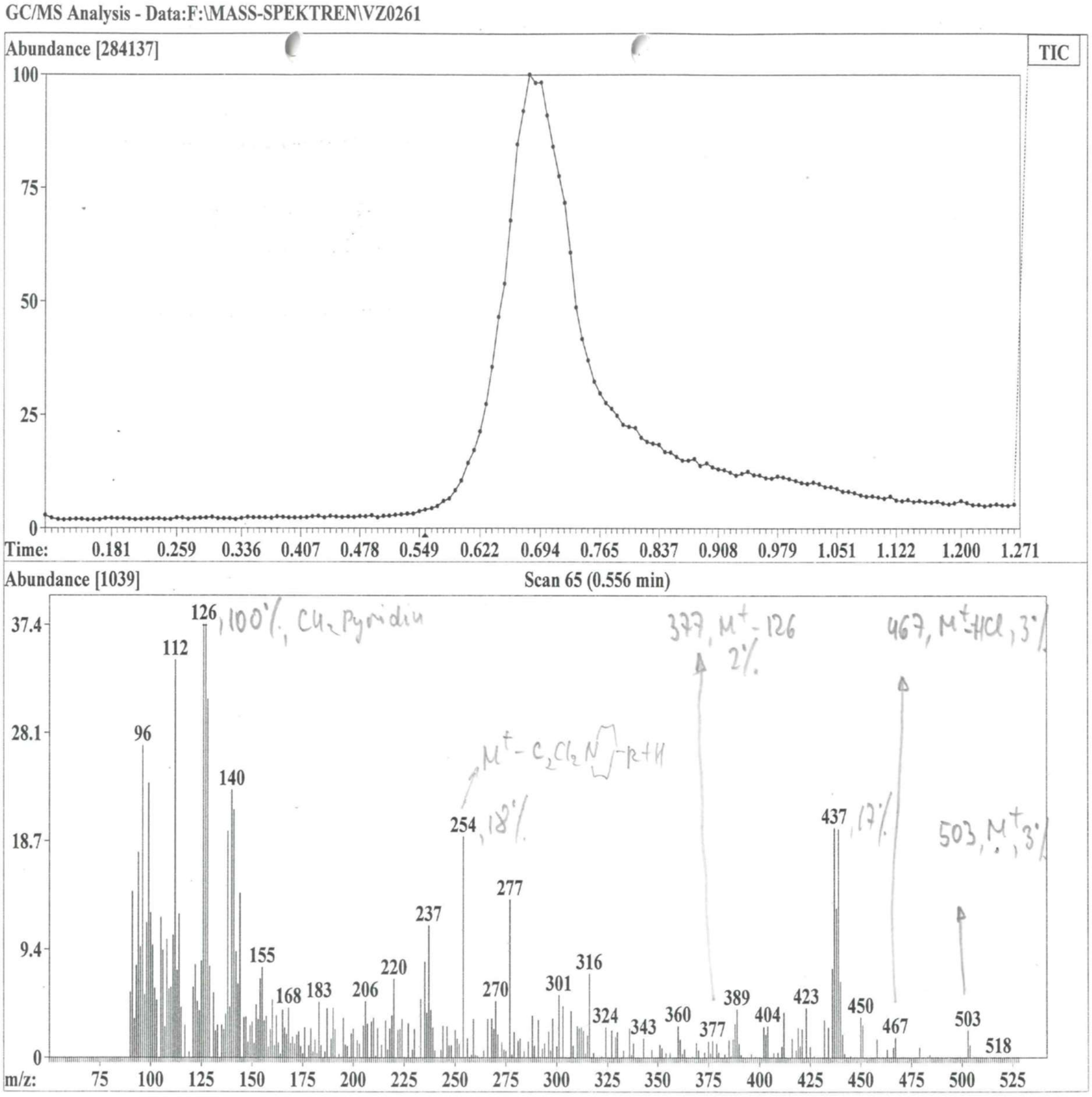

Figure S38. 200 MHz ^{1}H-NMR spectrum in $CDCl_3$ for **12b**.

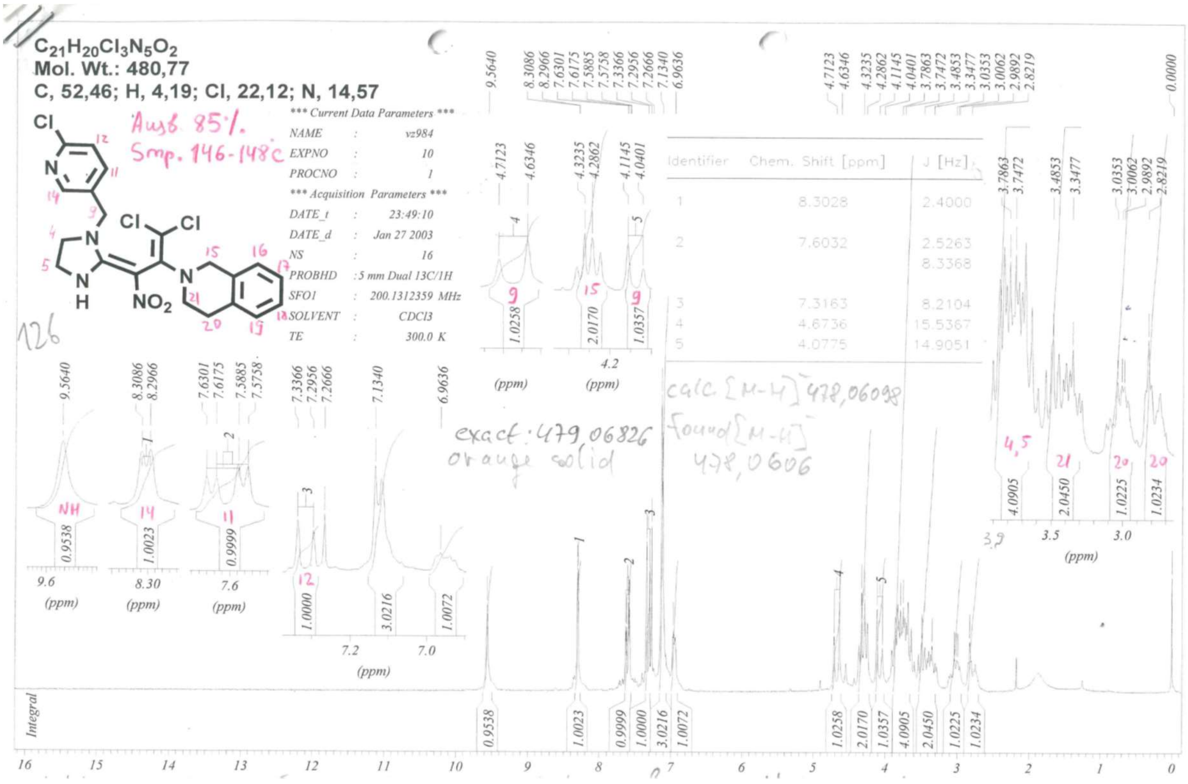

Figure S39. 50 MHz ^{13}C-NMR spectrum in $CDCl_3$ for **12b**.

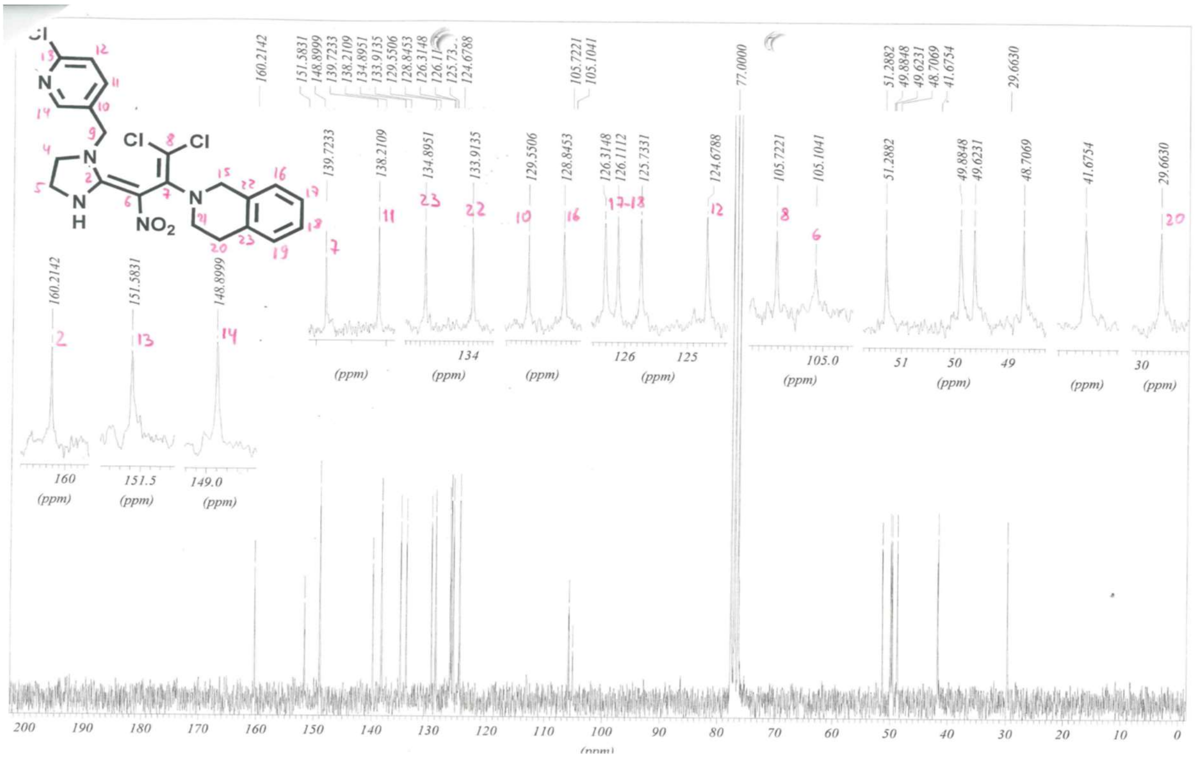

Figure S40. Mass spectrum for 2-(1,1-dichloro-3-{1-[(6-chloropyridin-3-yl)methyl]imidazolidin-2-ylidene}-3-nitroprop-1-en-2-yl)-1,2,3,4-tetrahydroisoquinoline (**12b**).

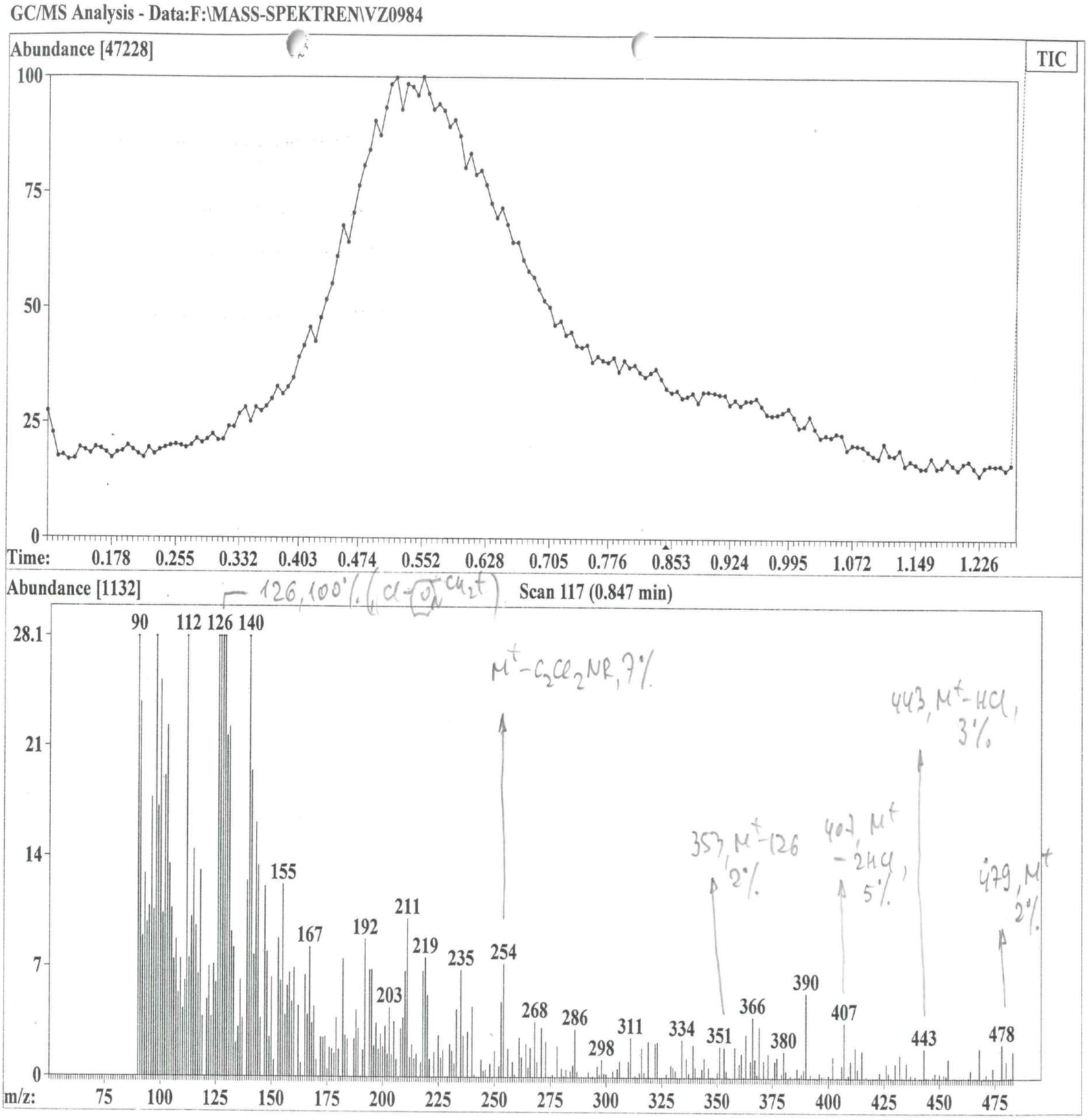

Figure S41. 200 MHz ^{1}H-NMR spectrum in DMSO-*d6* for **12c**.

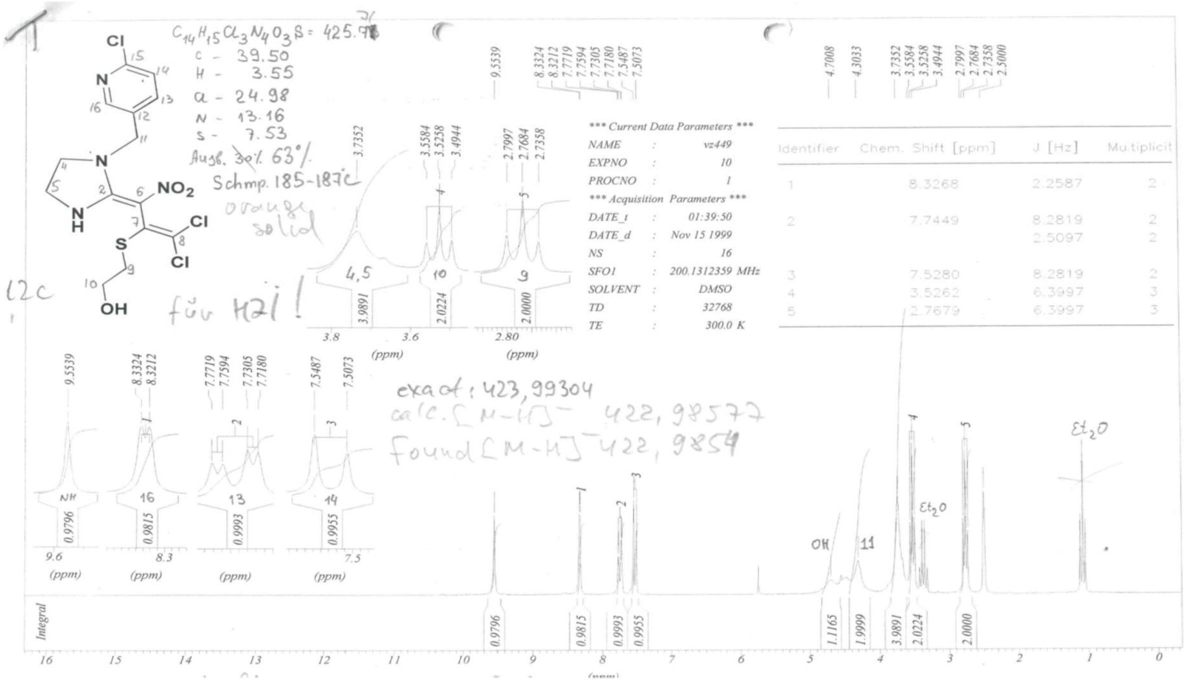

Figure S42. 50 MHz ^{13}C-NMR spectrum in DMSO-*d6* for **12c**.

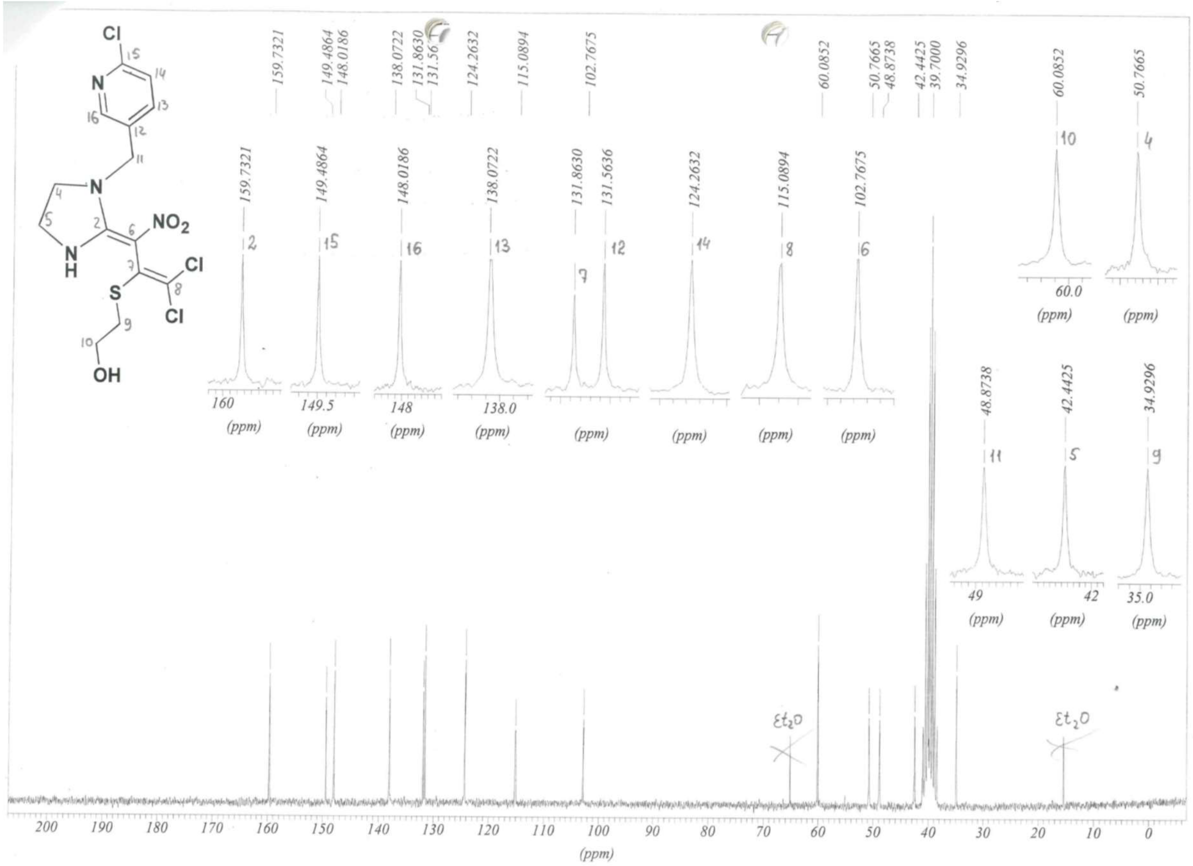

Figure S43. Mass spectrum for 2-[(1,1-dichloro-3-{1-[(6-chloropyridin-3-yl)methyl]imidazolidin-2-ylidene}-3-nitroprop-1-en-2-yl)-sulfanyl]ethanol (**12c**).

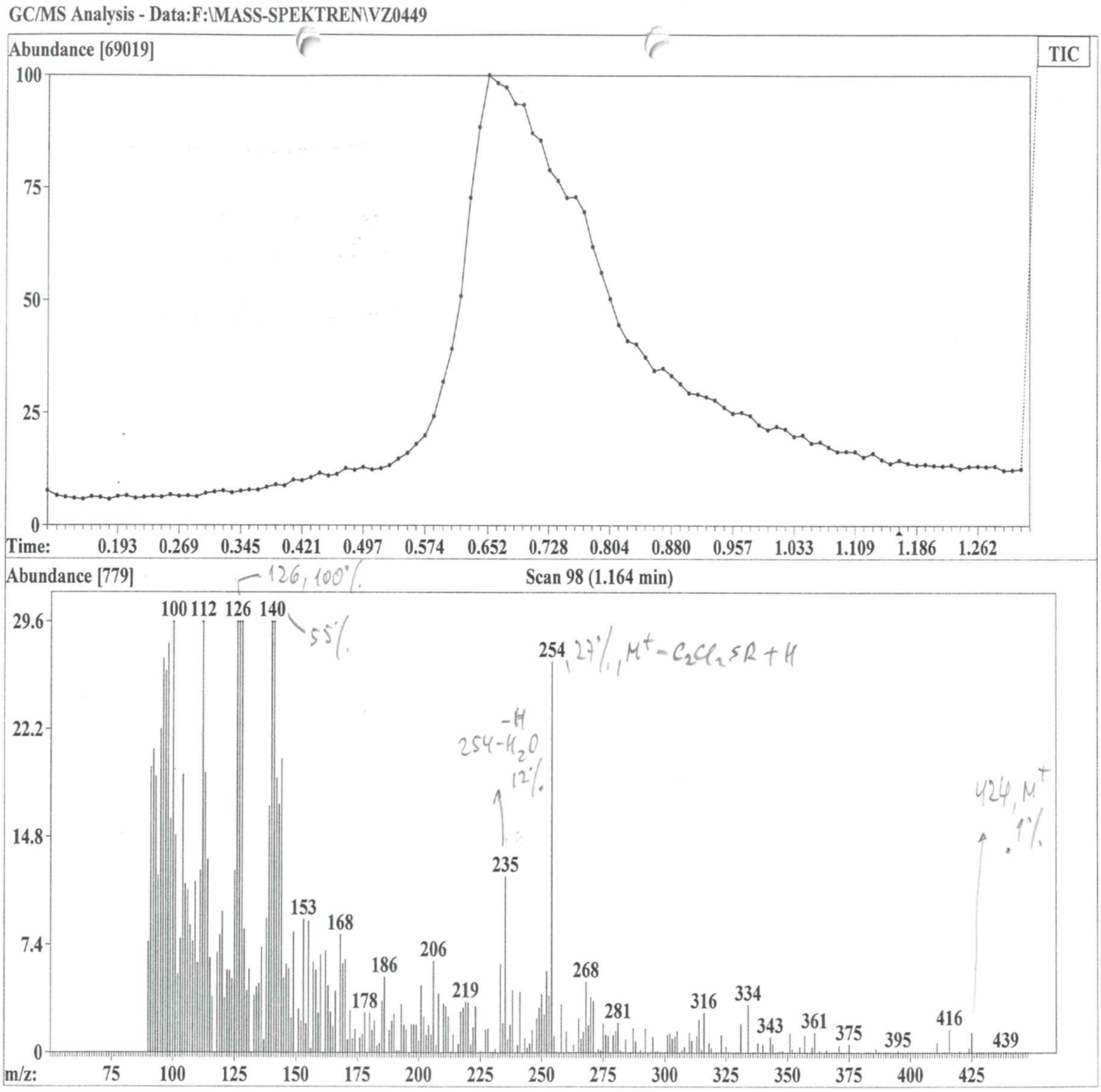

Figure S44. 200 MHz ^{1}H-NMR spectrum in DMSO-*d6* for **12d**.

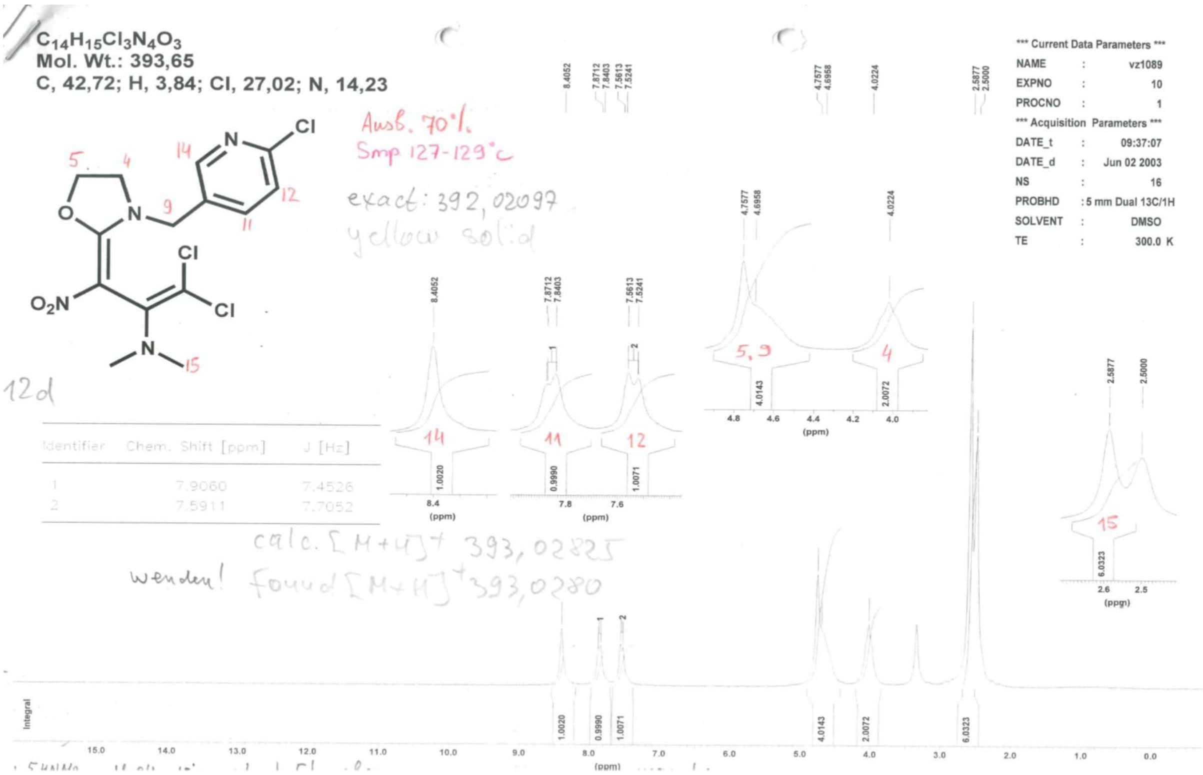

Figure S45. 50 MHz ^{13}C-NMR spectrum in DMSO-*d6* for **12d**.

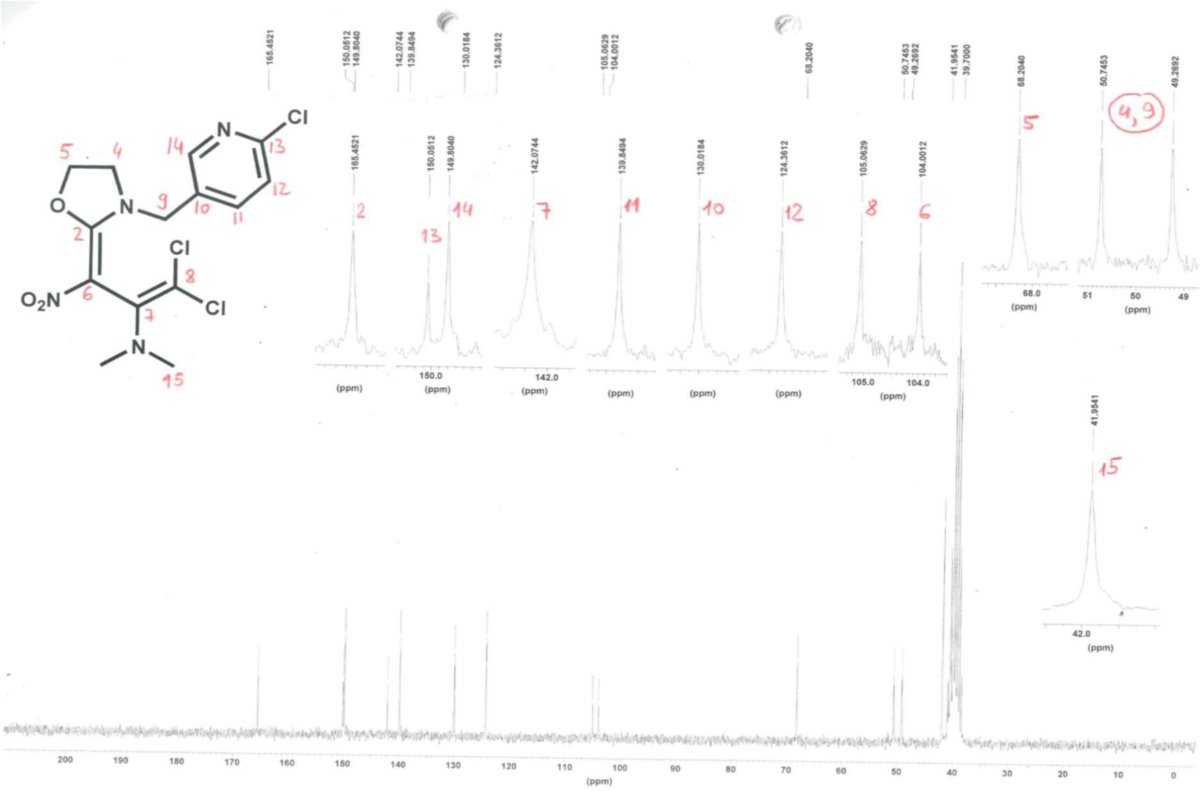

Figure S46. Mass spectrum for 1,1-dichloro-3-{3-[(6-chloropyridin-3-yl)methyl]-1,3-oxazolidin-2-ylidene}-*N*,*N*-dimethyl-3-nitroprop-1-en-2-amine (**12d**).

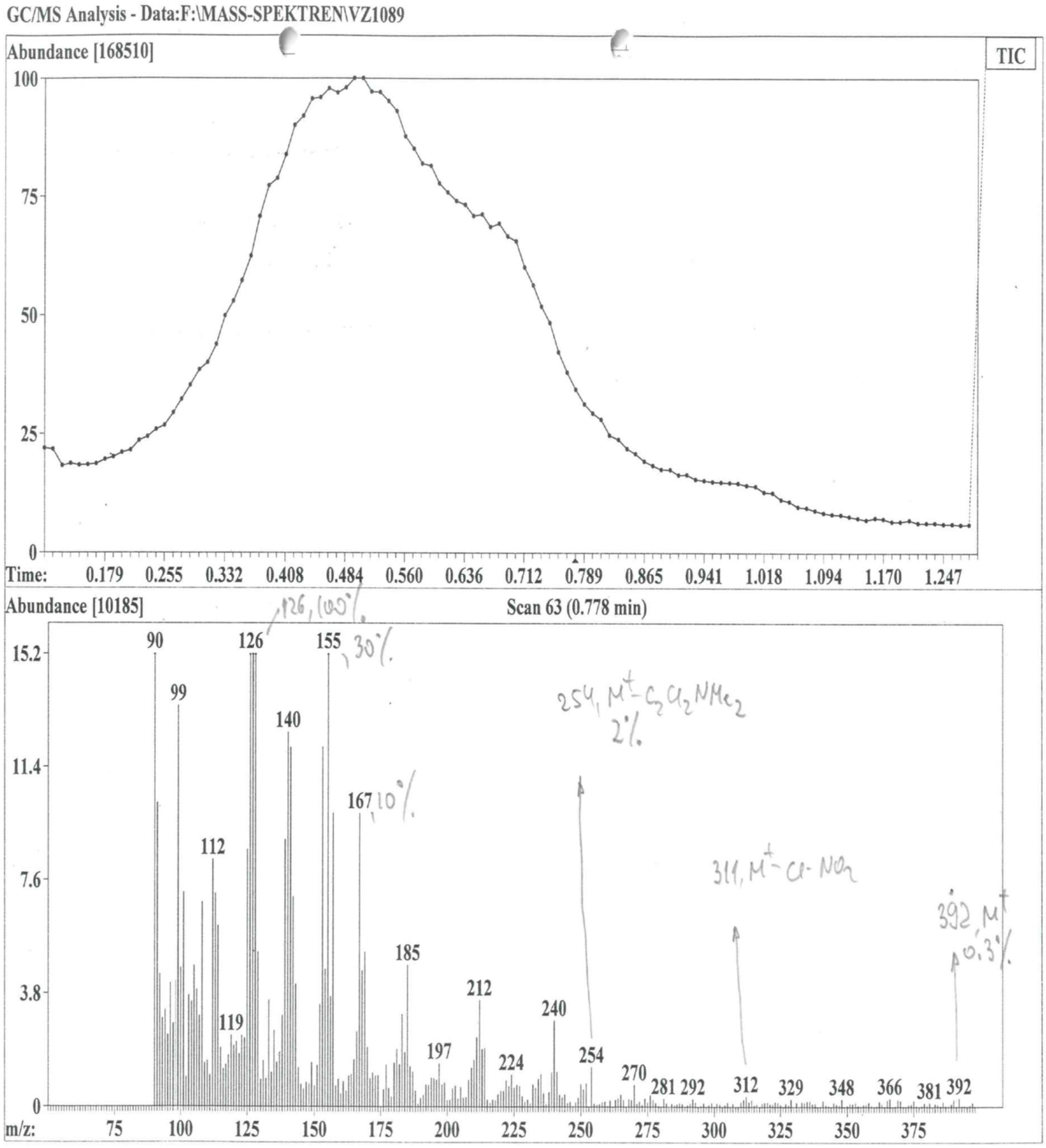

Figure S47. 400 MHz ^{1}H-NMR spectrum in $CDCl_3$ for **13**.

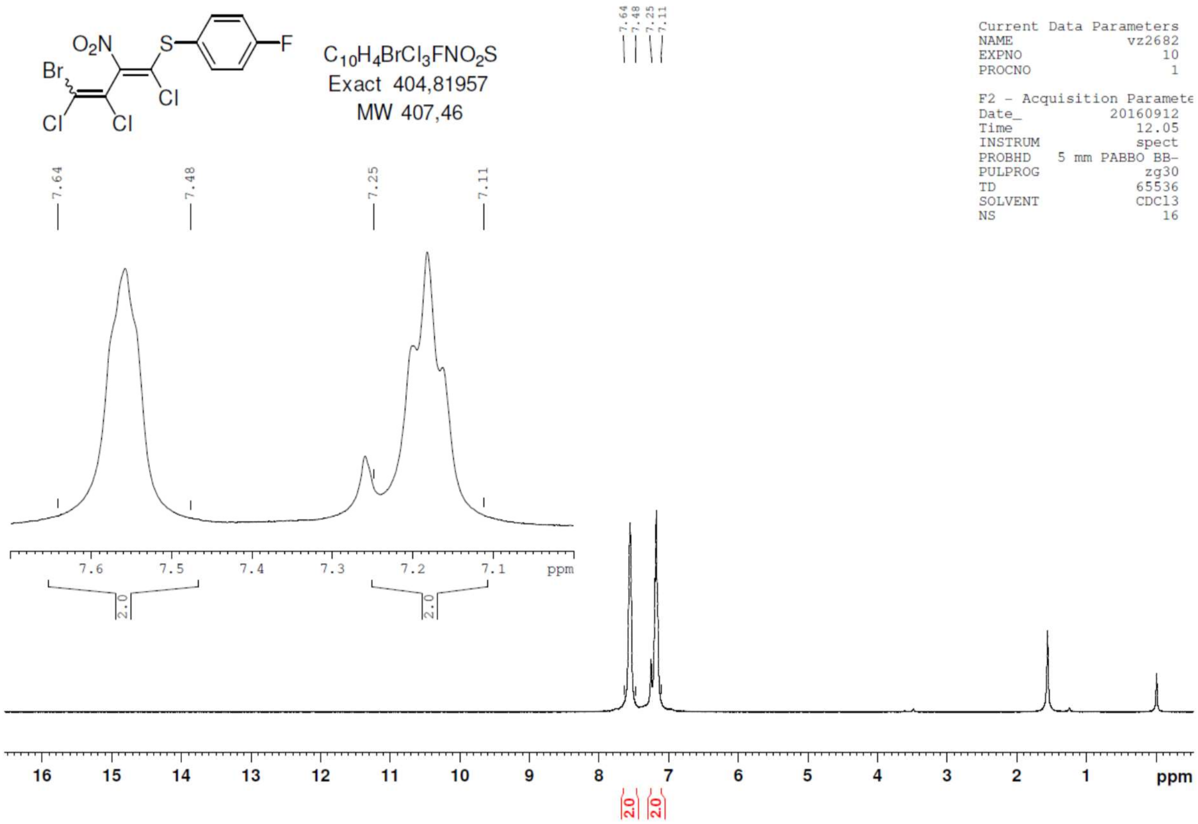

Figure S48. 101 MHz ^{13}C-NMR spectrum in $CDCl_3$ for **13**.

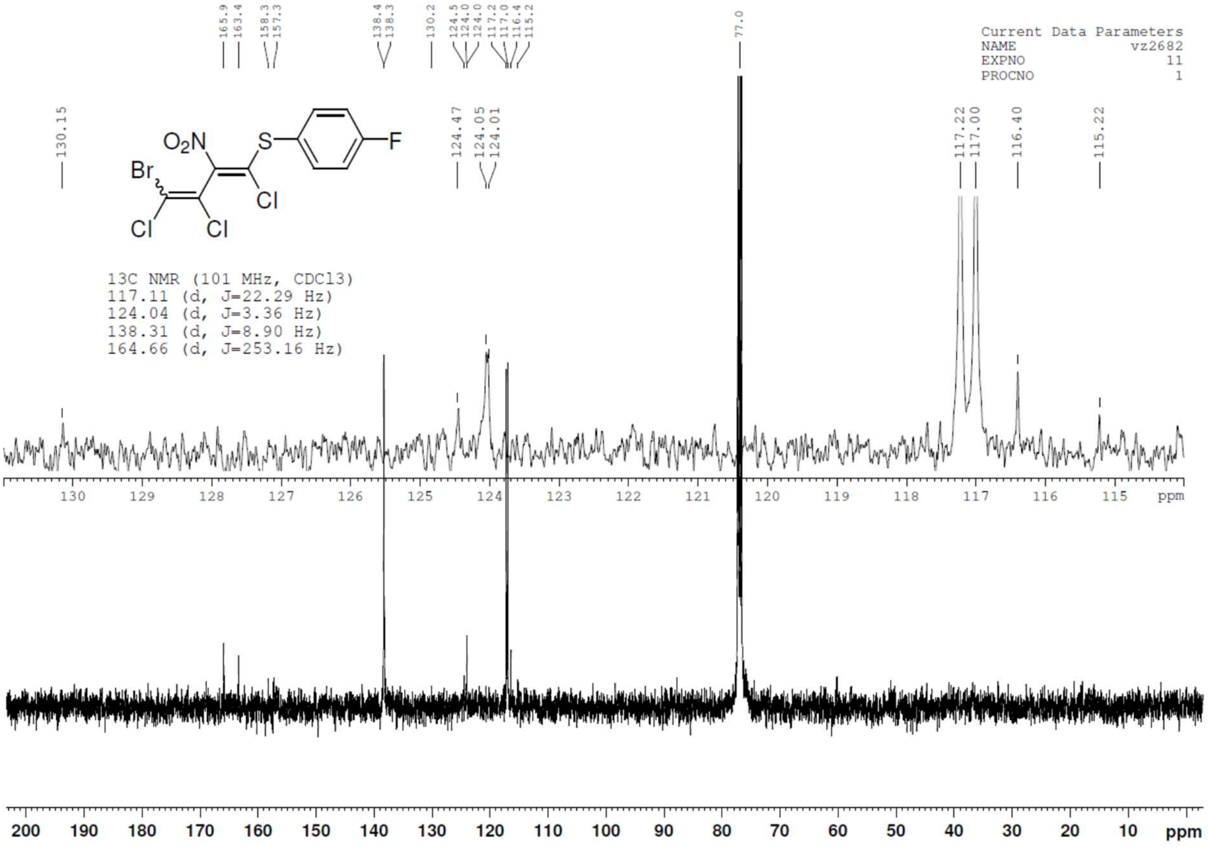

Figure S49. 200 MHz ^{1}H-NMR spectrum in DMSO-*d6* for **14**.

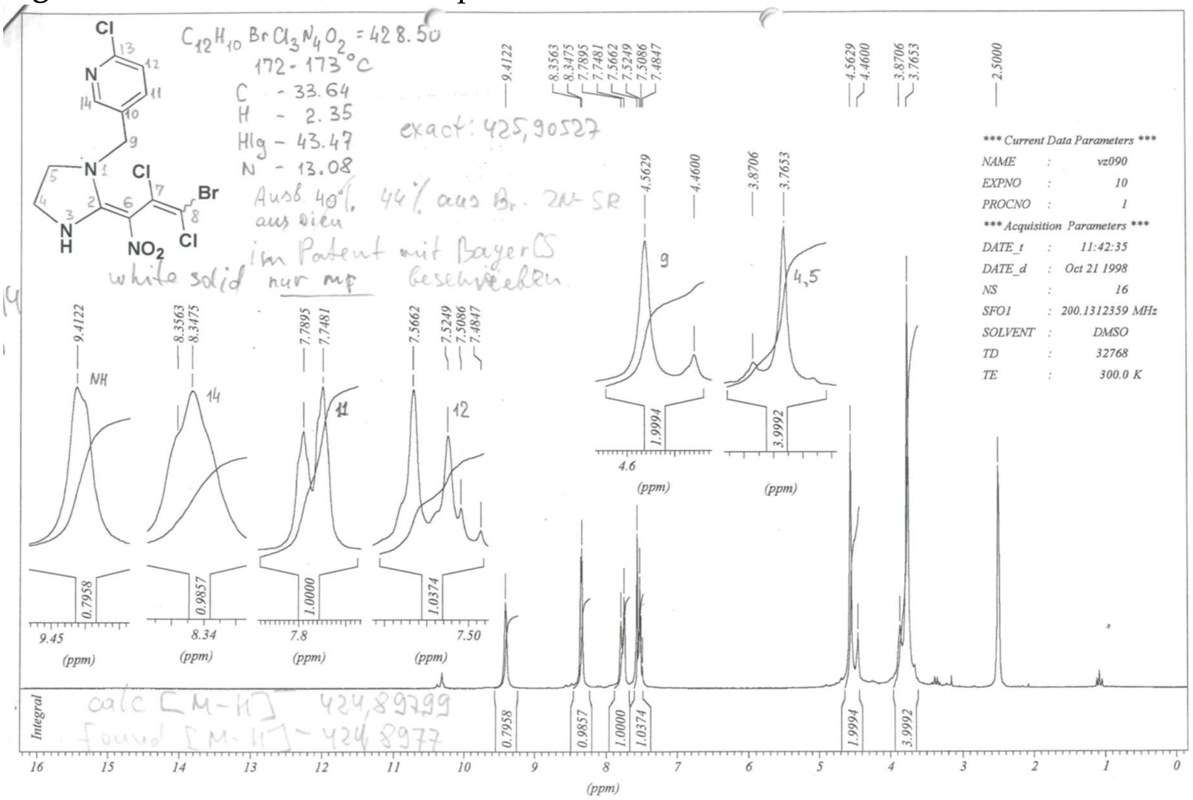

Figure S50. 50 MHz ^{1c}C-NMR spectrum in DMSO-*d6* for **14**.

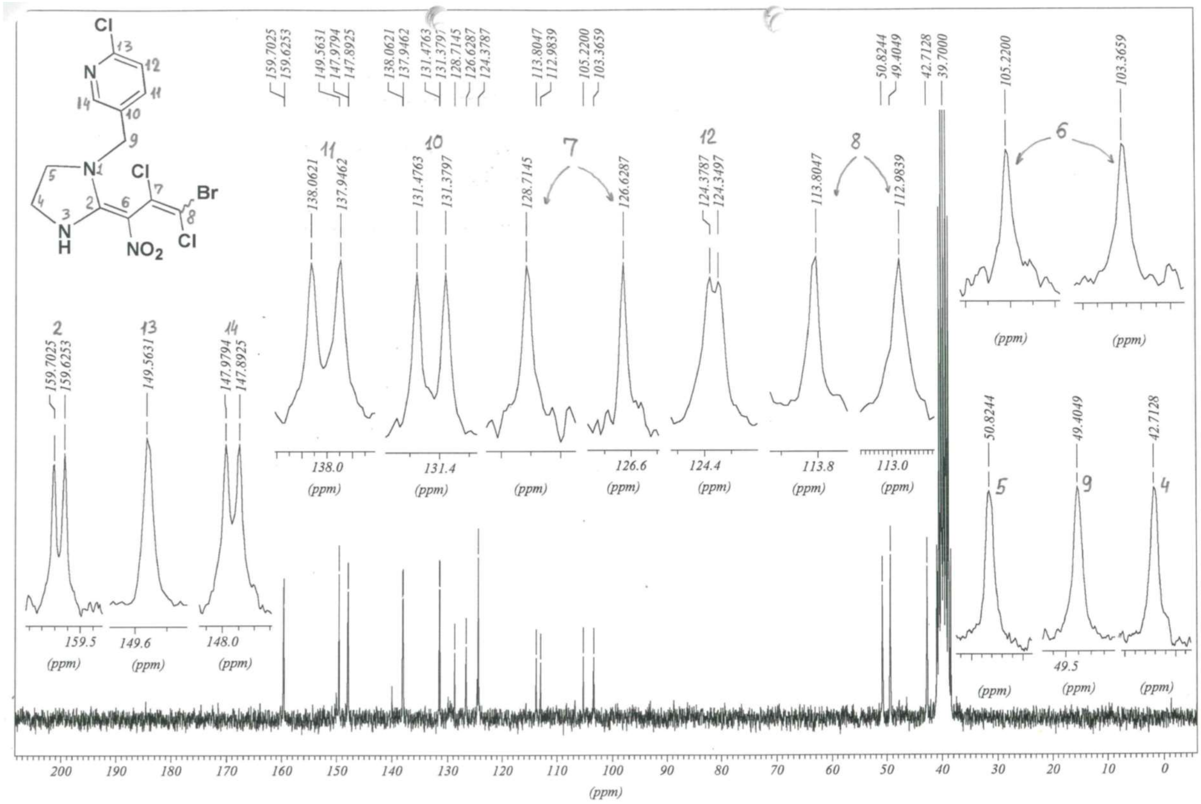

Figure S50. Mass spectrum for 5-(((2*E*)-2-(3-bromo-2,3-dichloro-1-nitroallylidene)imidazolidin-1-yl)methyl)-2-chloropyridine (**14**).

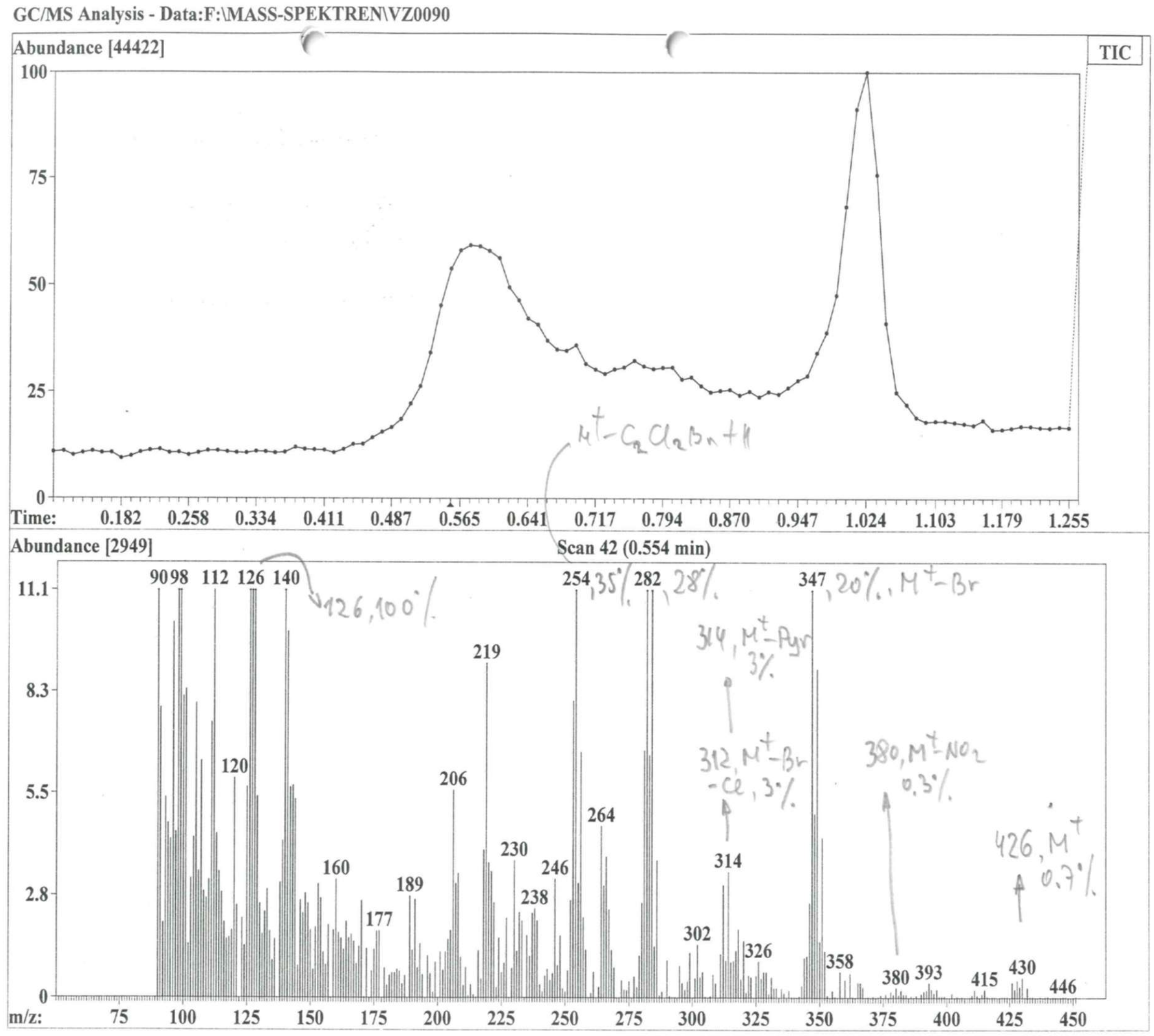

Figure S51. 400 MHz ^{1}H-NMR spectrum in $CDCl_3$ for **15**.

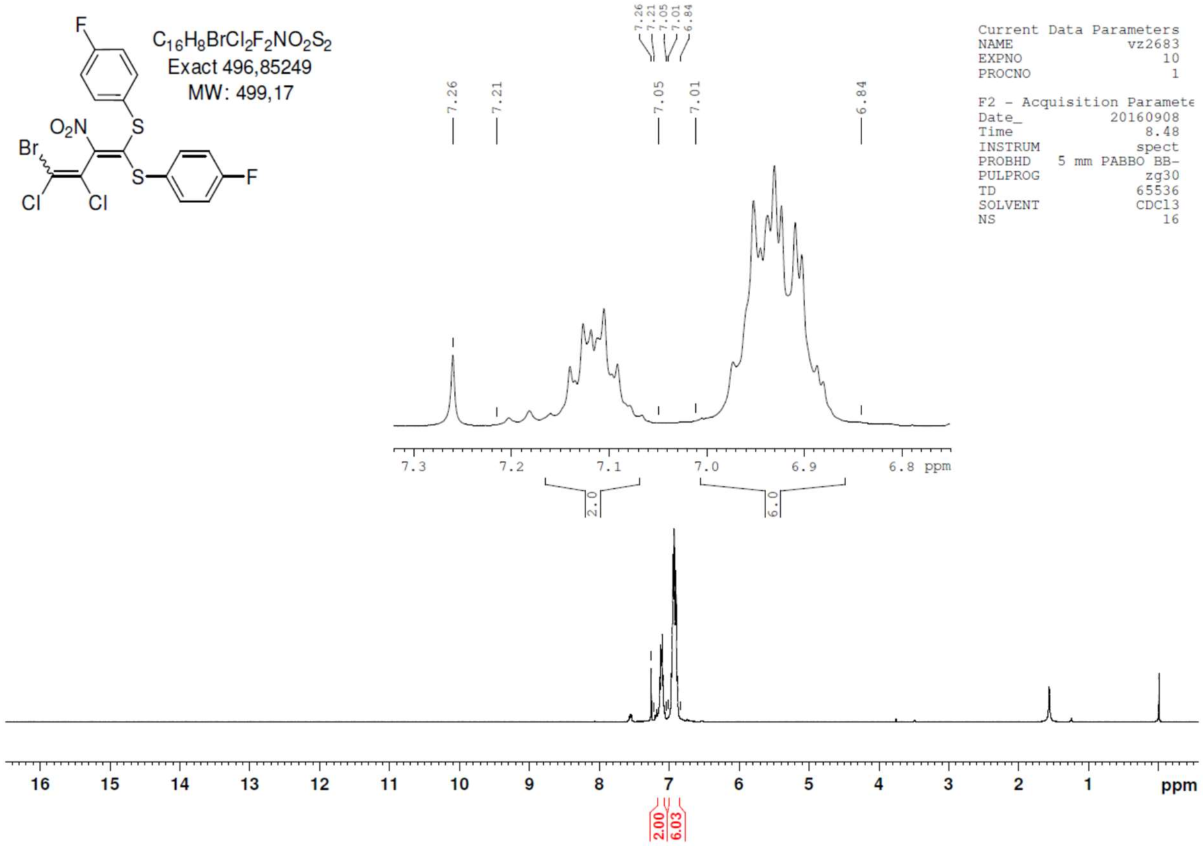

Figure S52. 100 MHz ^{13}C-NMR spectrum in $CDCl_3$ for **15**.

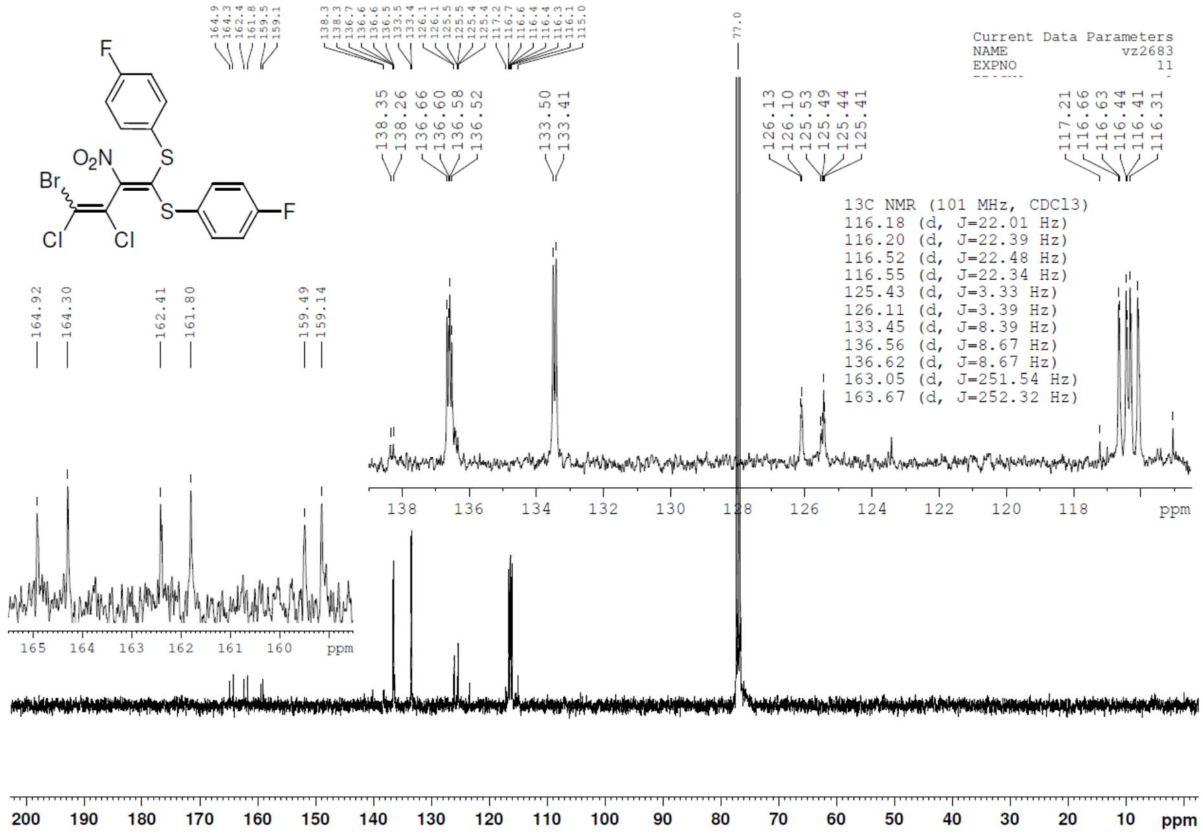

Figure S53. 400 MHz ^{1}H-NMR spectrum in DMSO-*d6* for **17**.

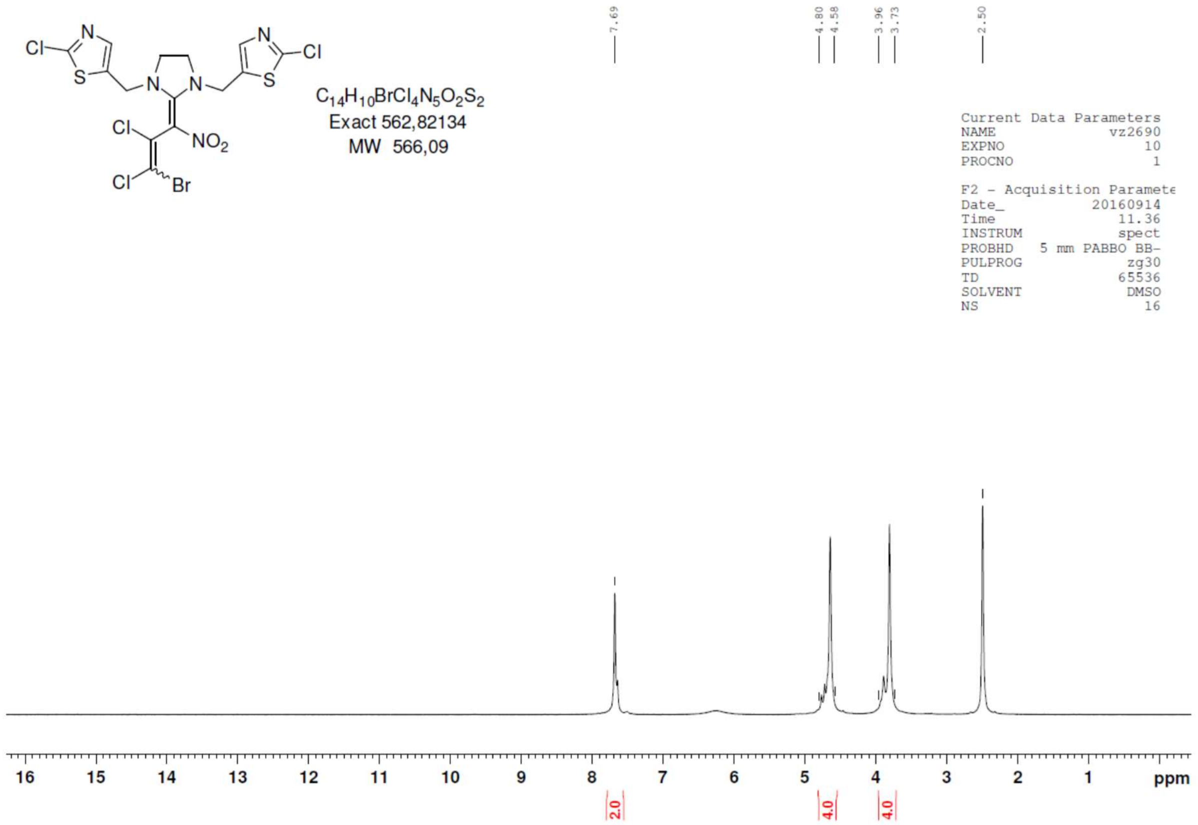

Figure S54. 100 MHz ^{13}C-NMR spectrum in DMSO-*d6* for **17**.

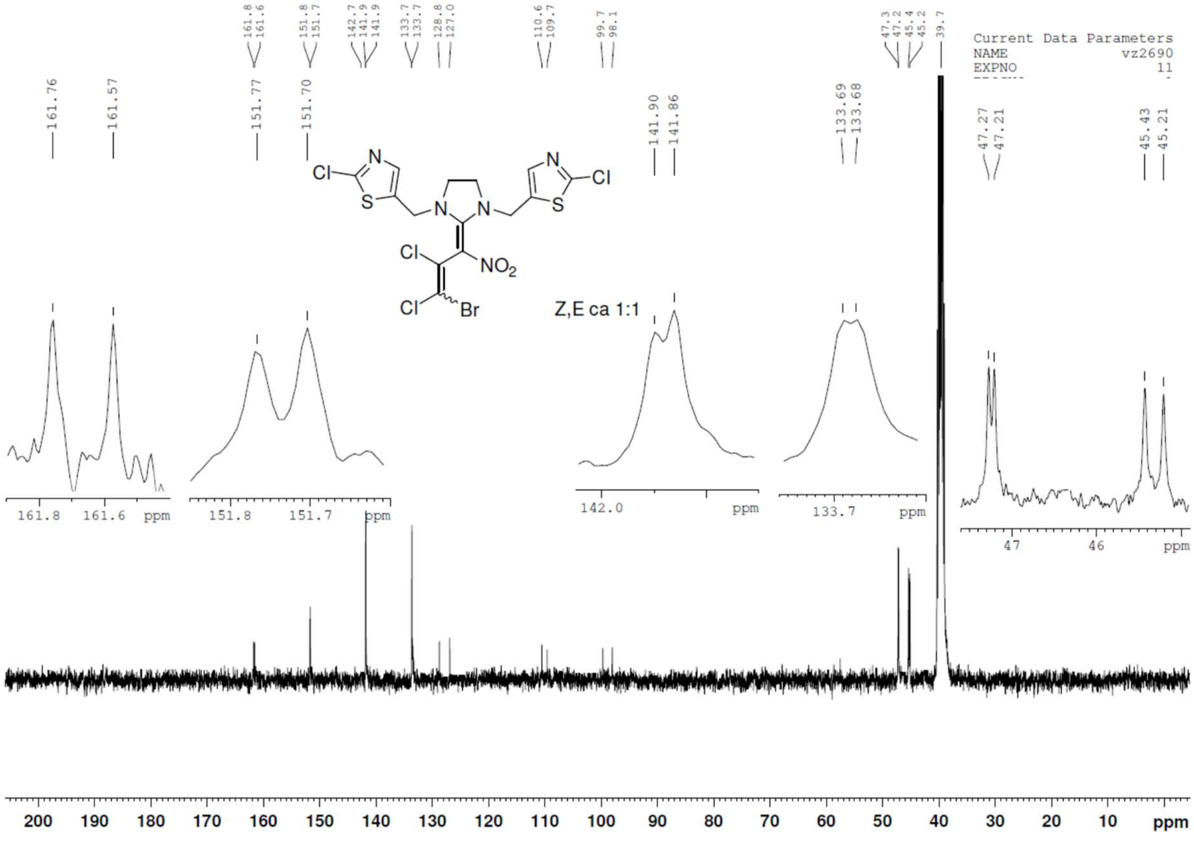

Figure S55. 400 MHz ^{1}H-NMR spectrum in DMSO-*d6* for **19a**.

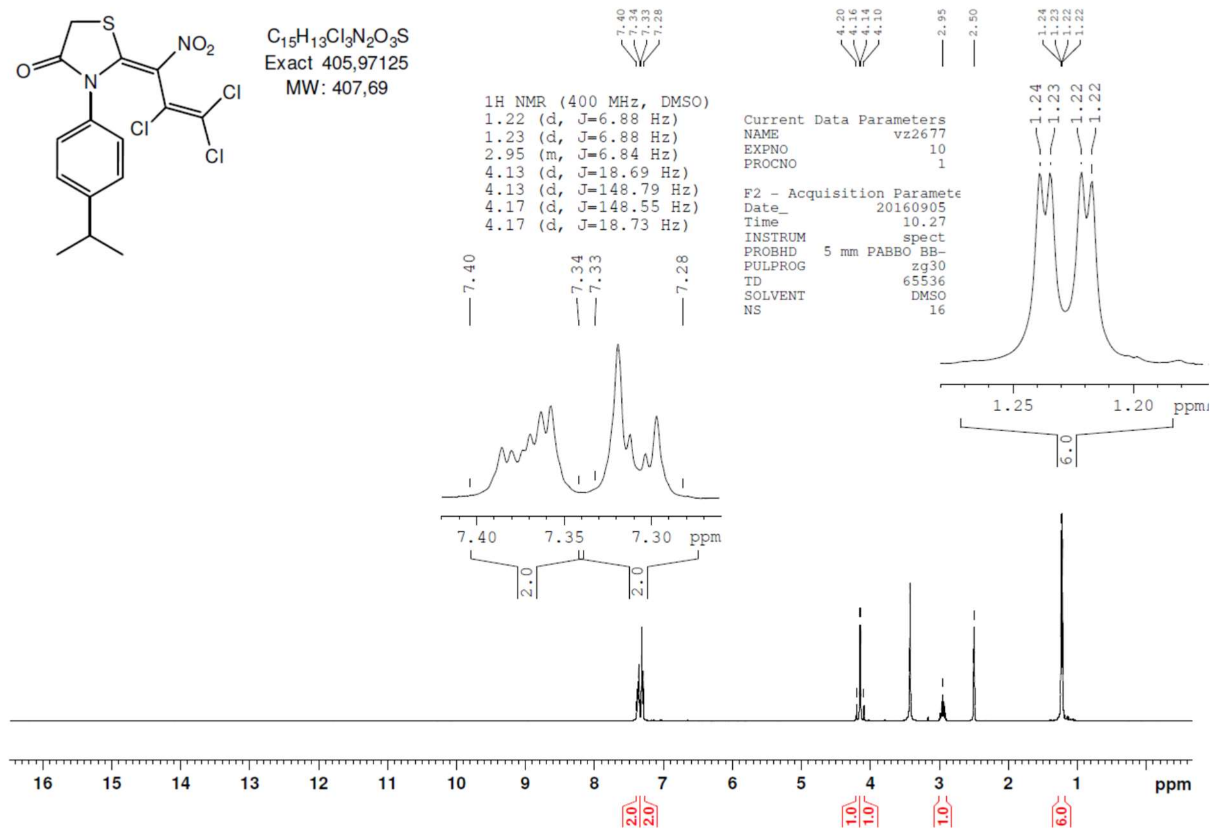

Figure S56. 100 MHz ^{13}C-NMR spectrum in DMSO-*d6* for **19a**.

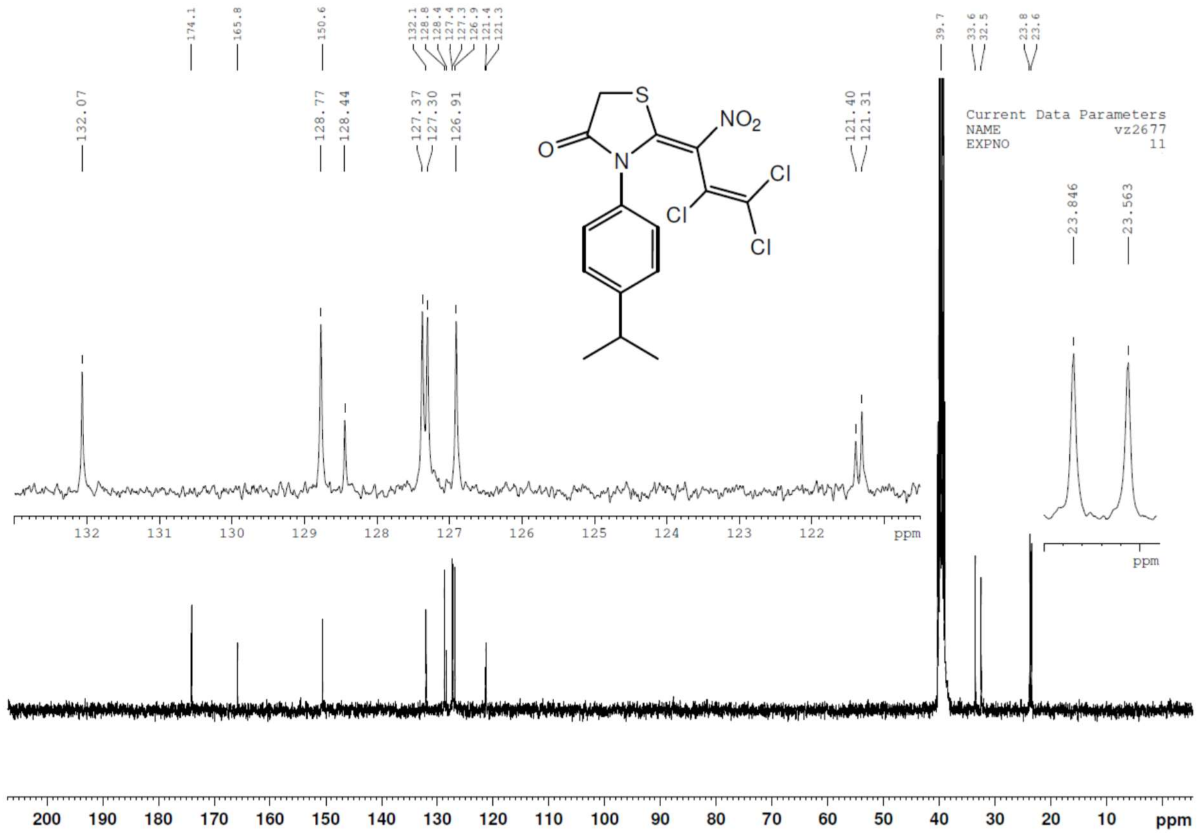

Figure S57. 400 MHz ^{1}H-NMR spectrum in DMSO-*d6* for **19b**.

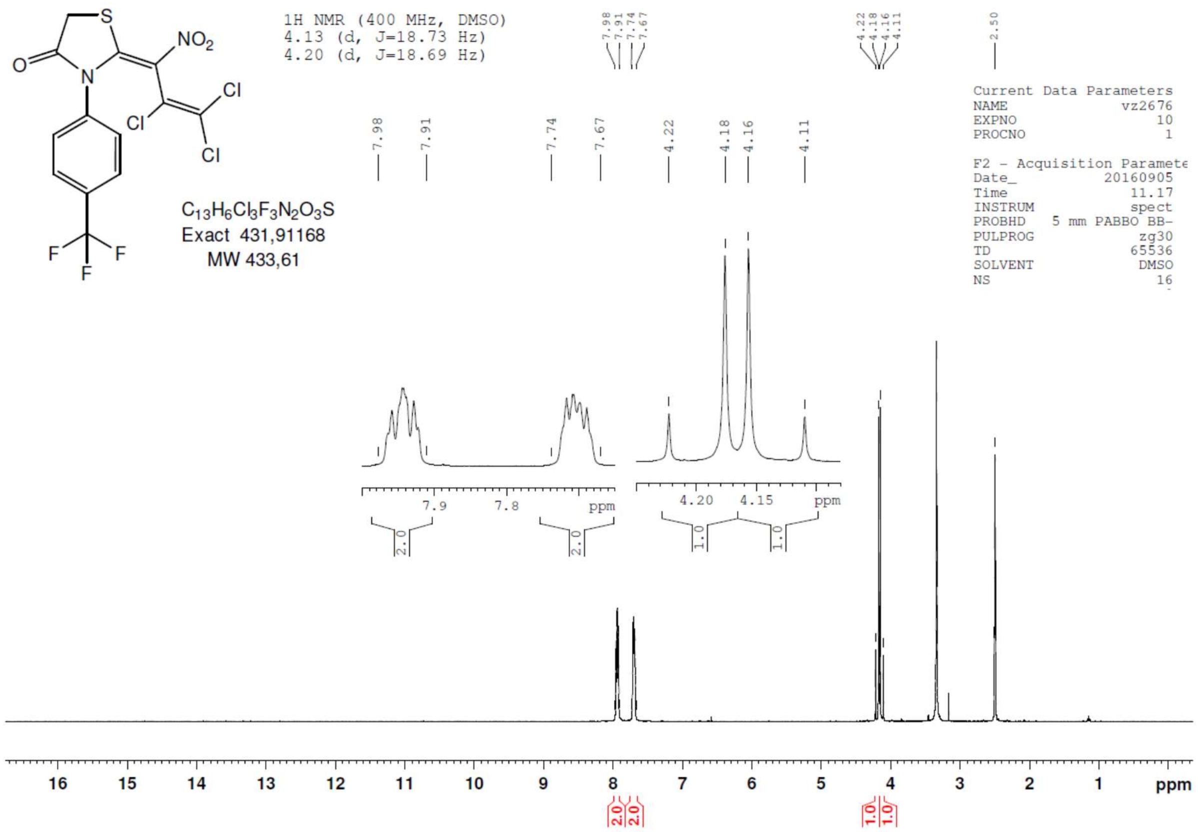

Figure S58. 100 MHz ^{13}C-NMR spectrum in DMSO-*d6* for **19b**.

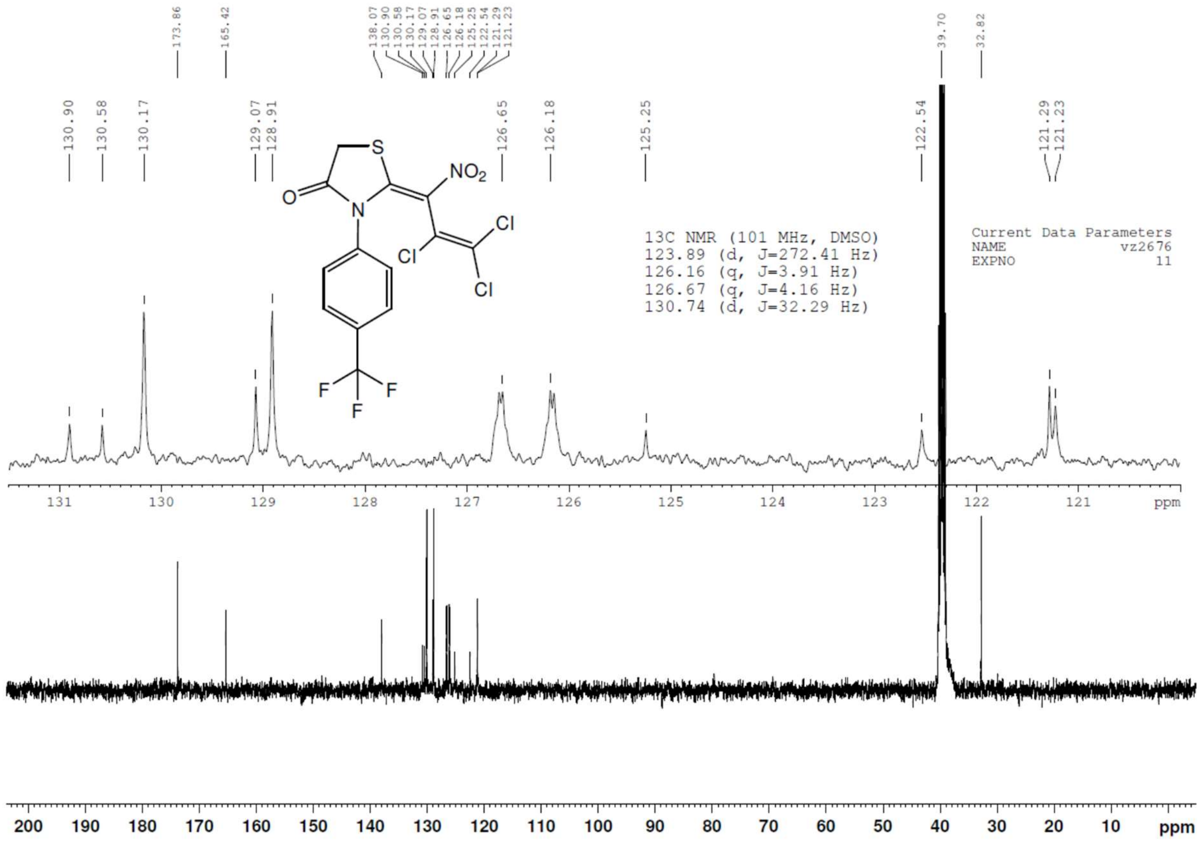

Figure S59. 400 MHz ^{1}H-NMR spectrum in DMSO-*d6* for **20a**.

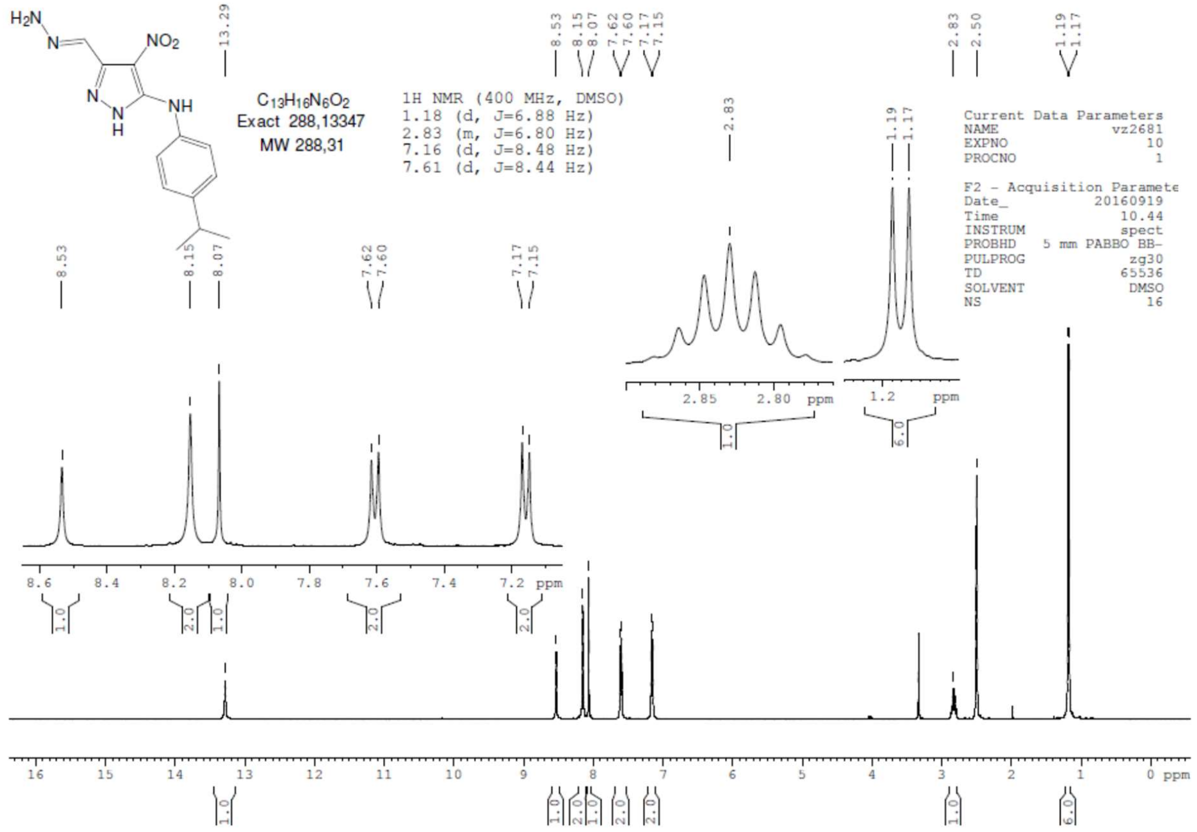

Figure S60. 100 MHz ^{13}C-NMR spectrum in DMSO-*d6* for **20a**.

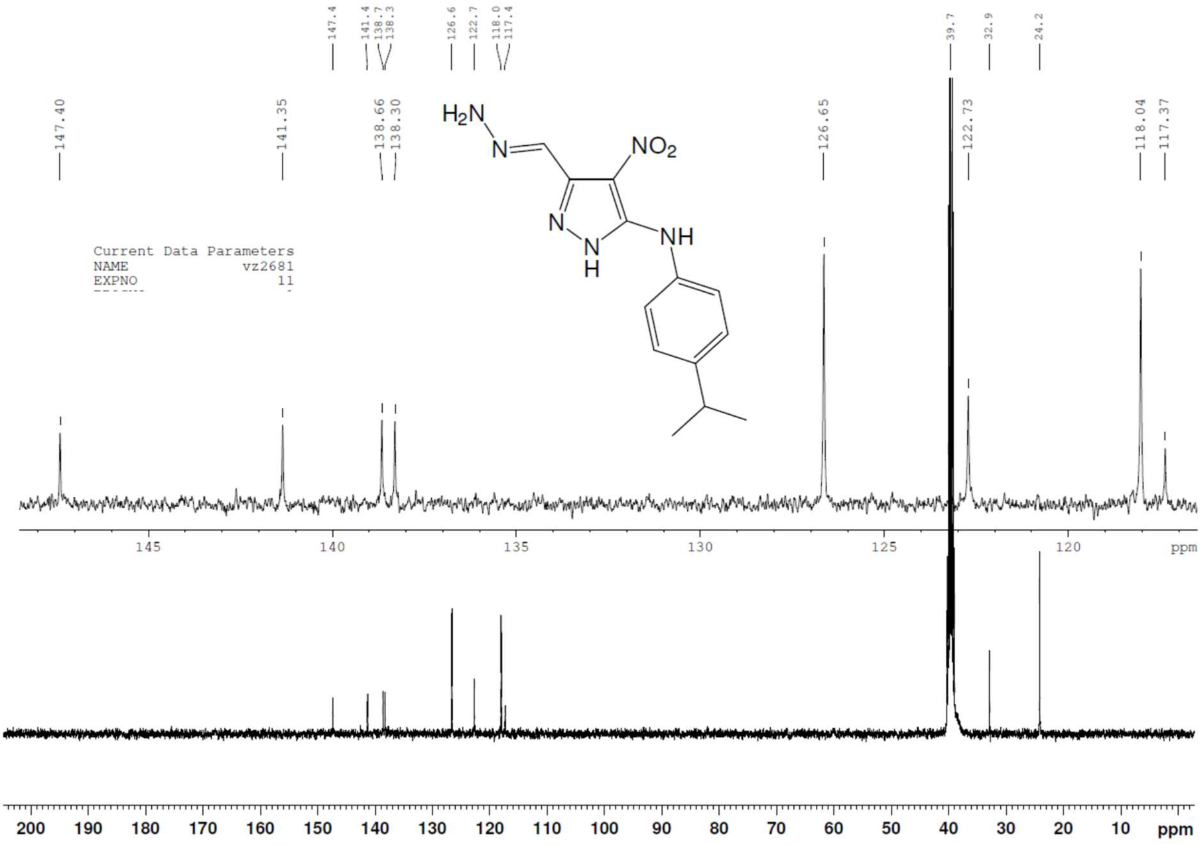

Figure S61. 400 MHz ^{1}H-NMR spectrum in DMSO-*d6* for **20b**.

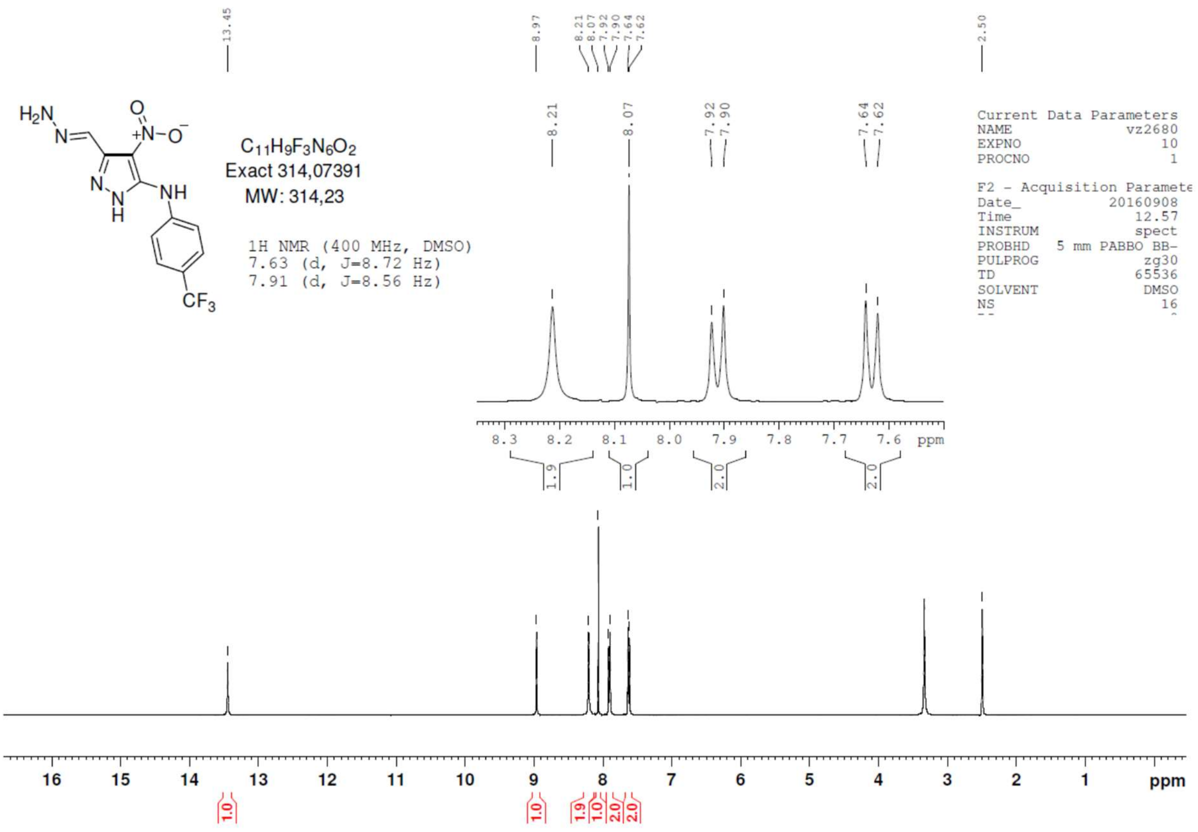

Figure S62. 100 MHz ^{13}C-NMR spectrum in DMSO-*d6* for **20b**.

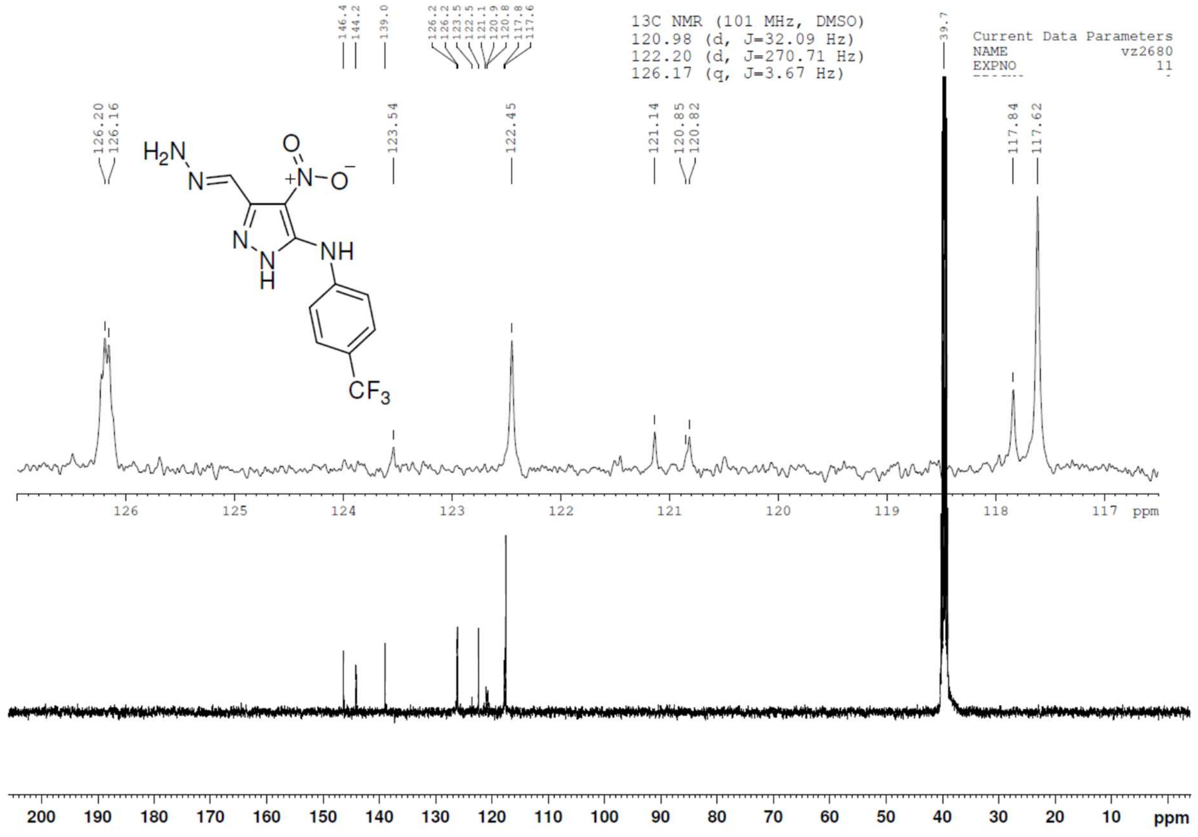

Figure S63. 400 MHz ^{1}H-NMR spectrum in DMSO-*d6* for **21a**.

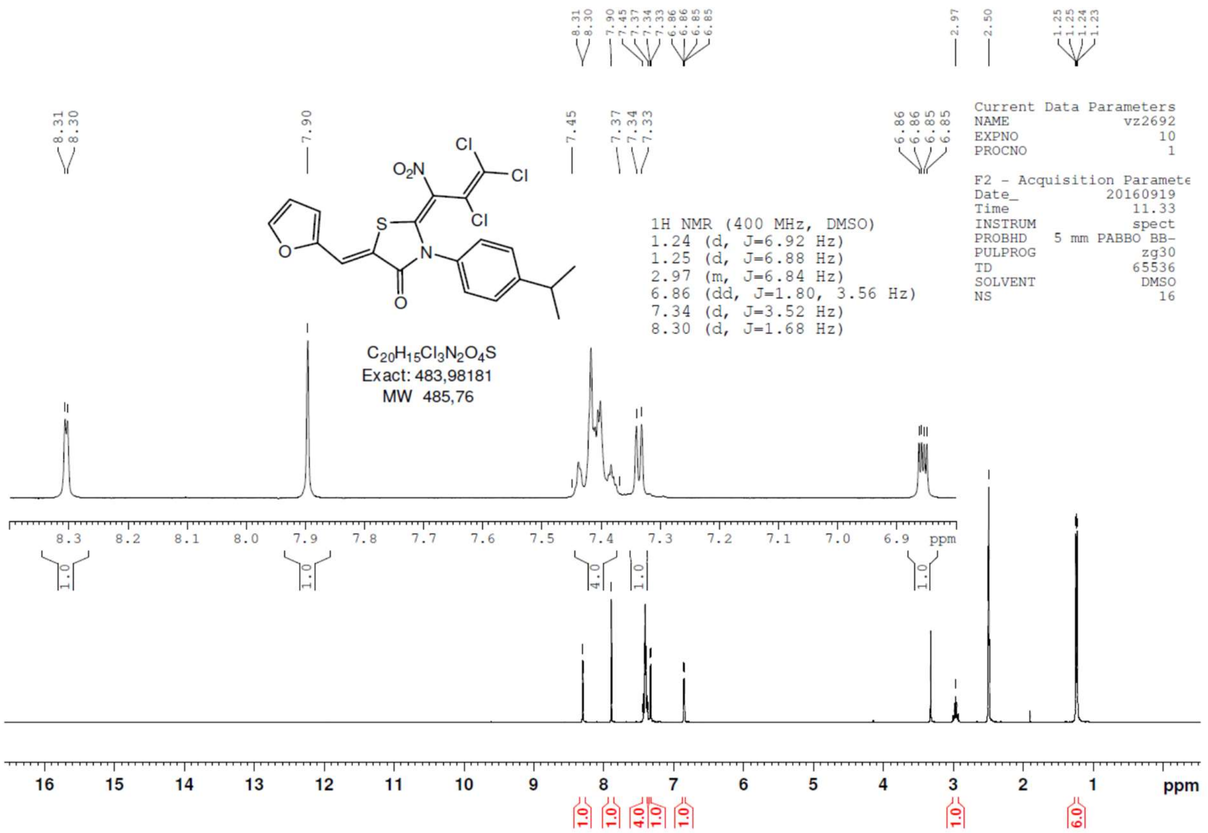

Figure S64. 100 MHz ^{13}C-NMR spectrum in DMSO-*d6* for **21a**.

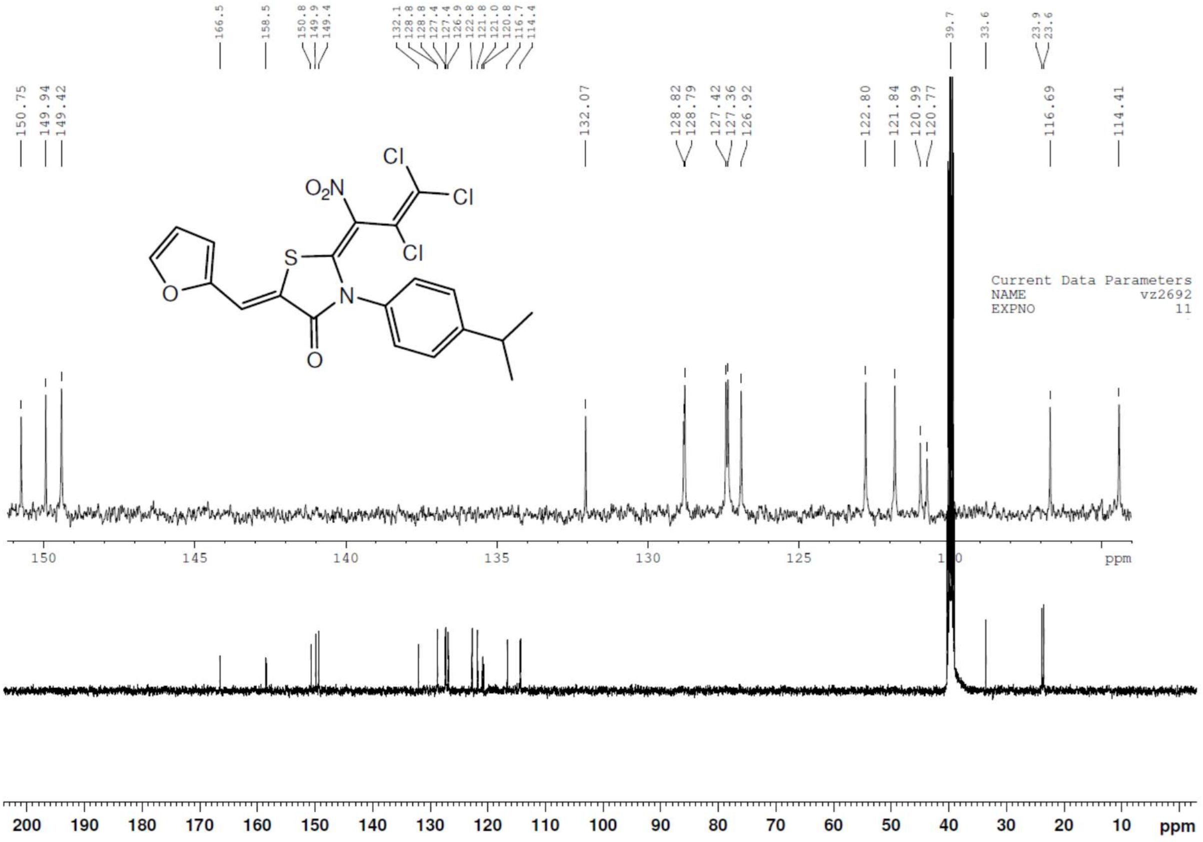

Figure S65. 400 MHz ^{1}H-NMR spectrum in $CDCl_3$ for **21b**.

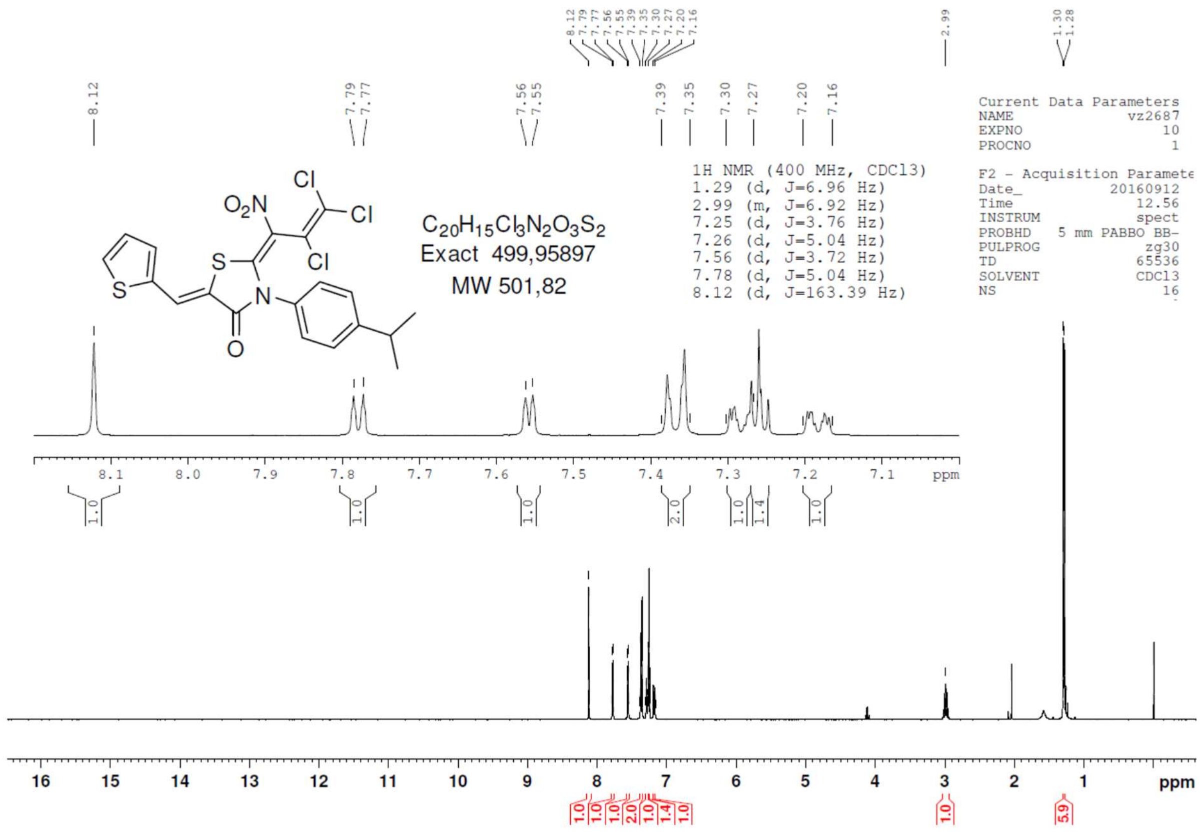

Figure S66. 100 MHz ^{13}C-NMR spectrum in $CDCl_3$ for **21b.**

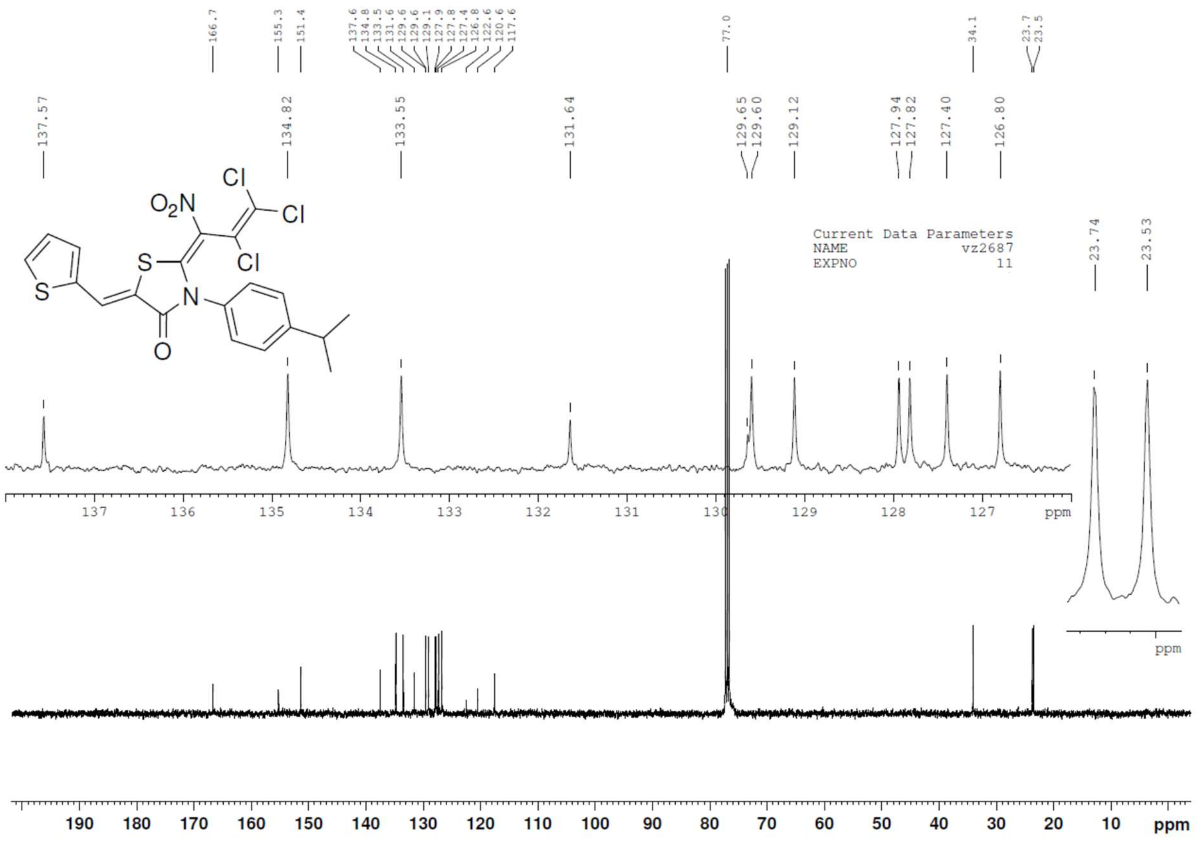

Figure S67. 400 MHz ^{1}H-NMR spectrum in DMSO-*d6* for **21c**.

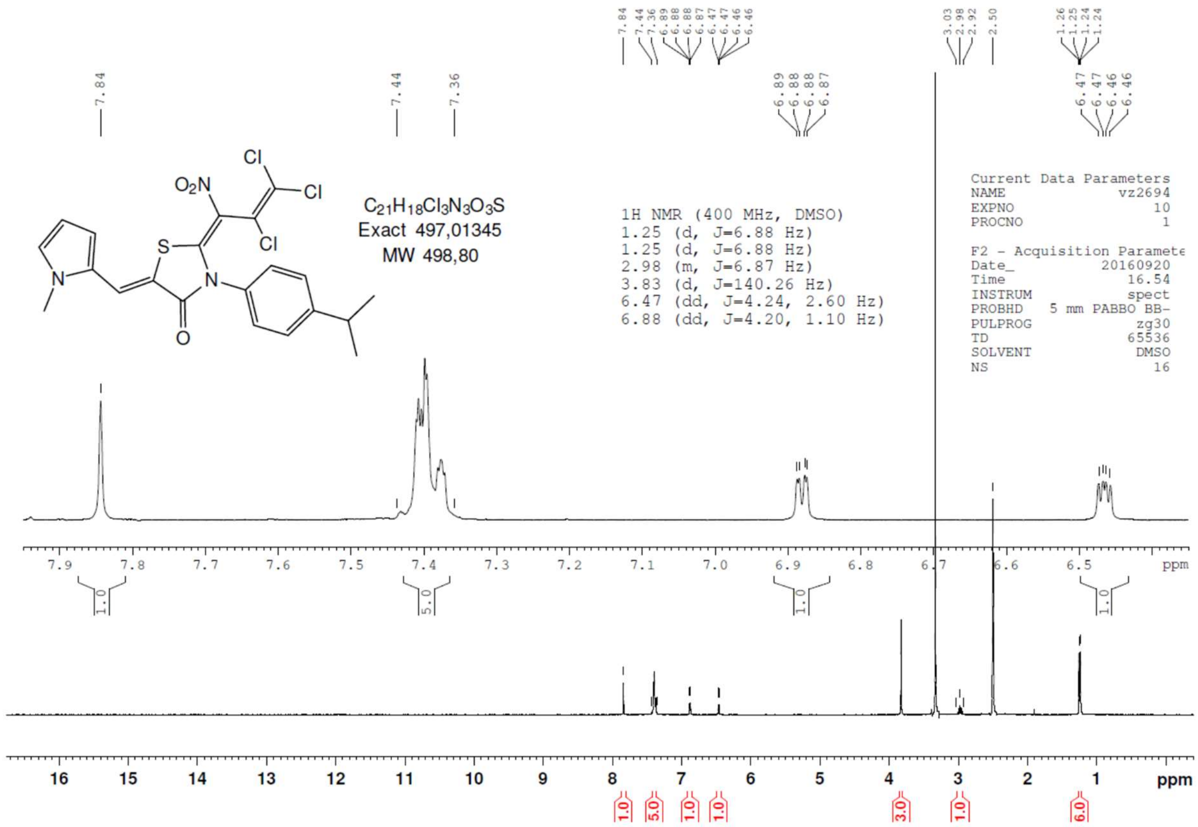

Figure S68. 100 MHz ^{13}C-NMR spectrum in DMSO-*d6* for **21c**.

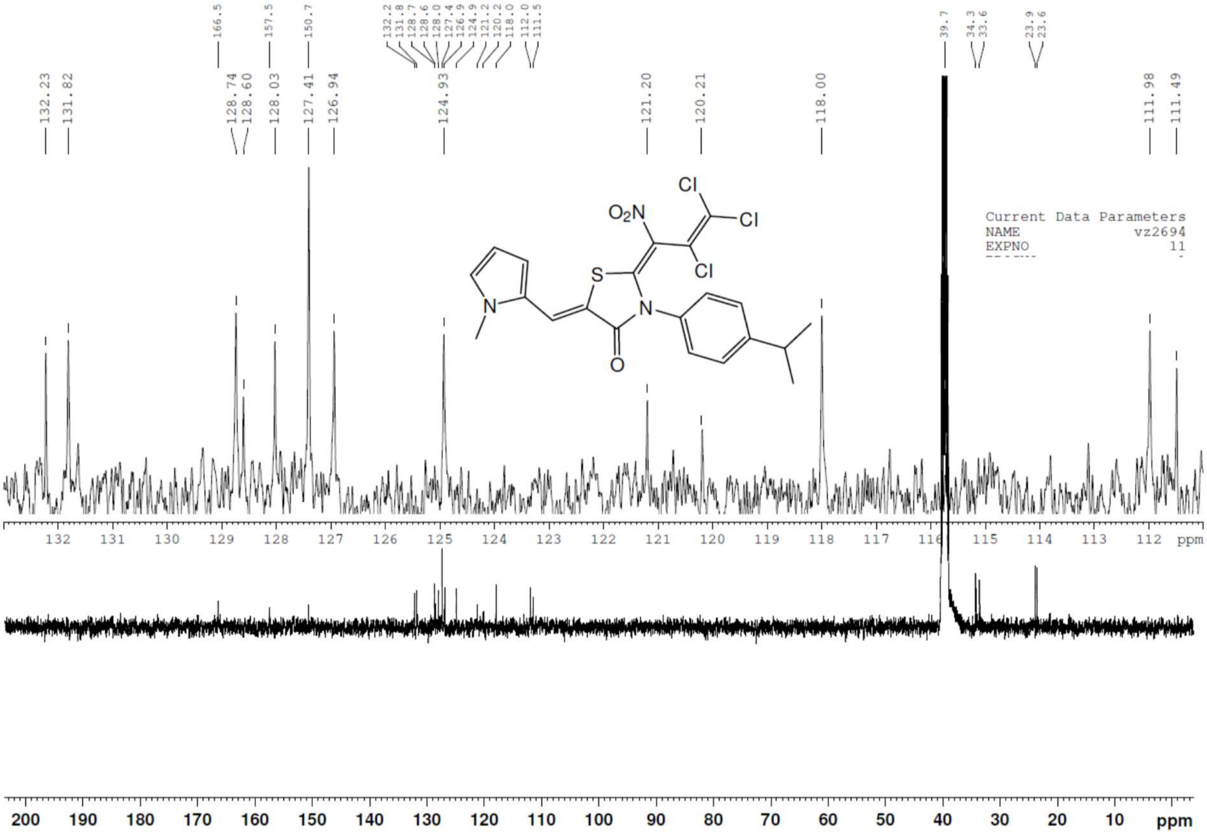

Figure S69. 400 MHz ^{1}H-NMR spectrum in CDCl₃ for **21d**.

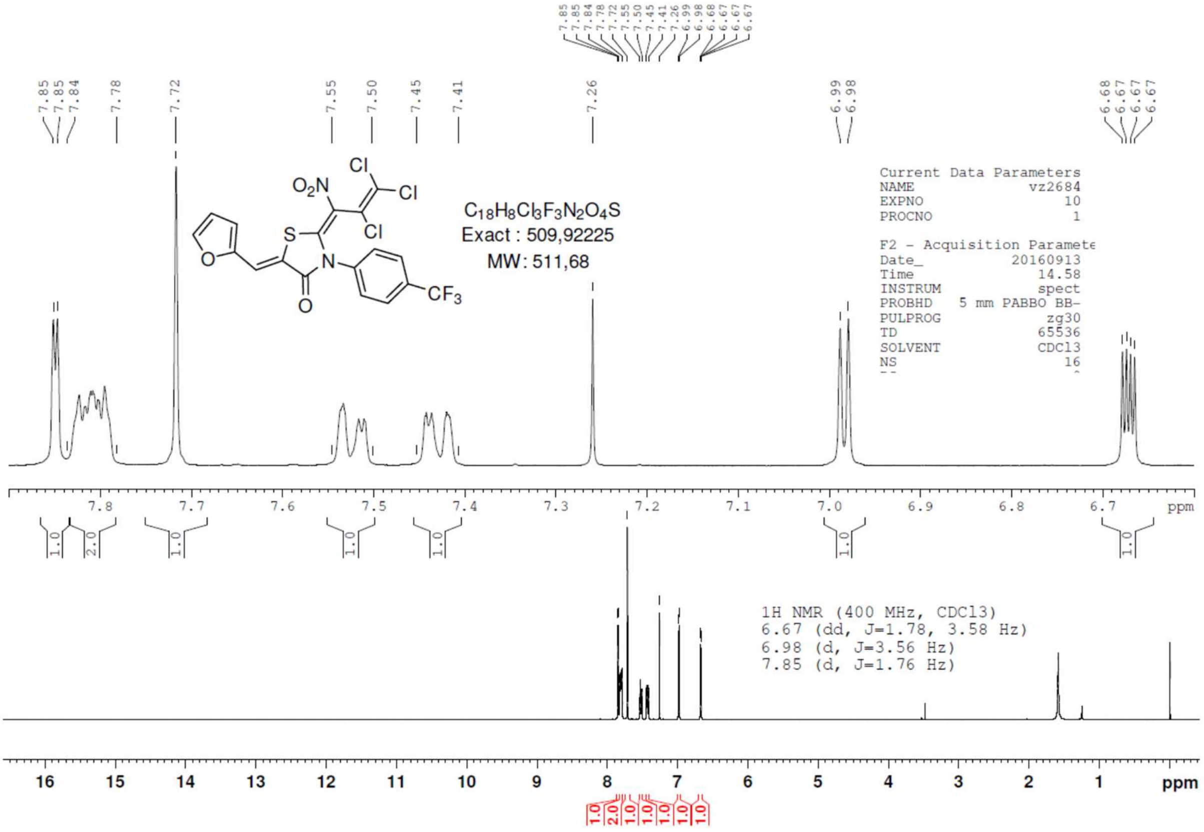

Figure S70. 100 MHz ^{13}C-NMR spectrum in $CDCl_3$ for **21d**.

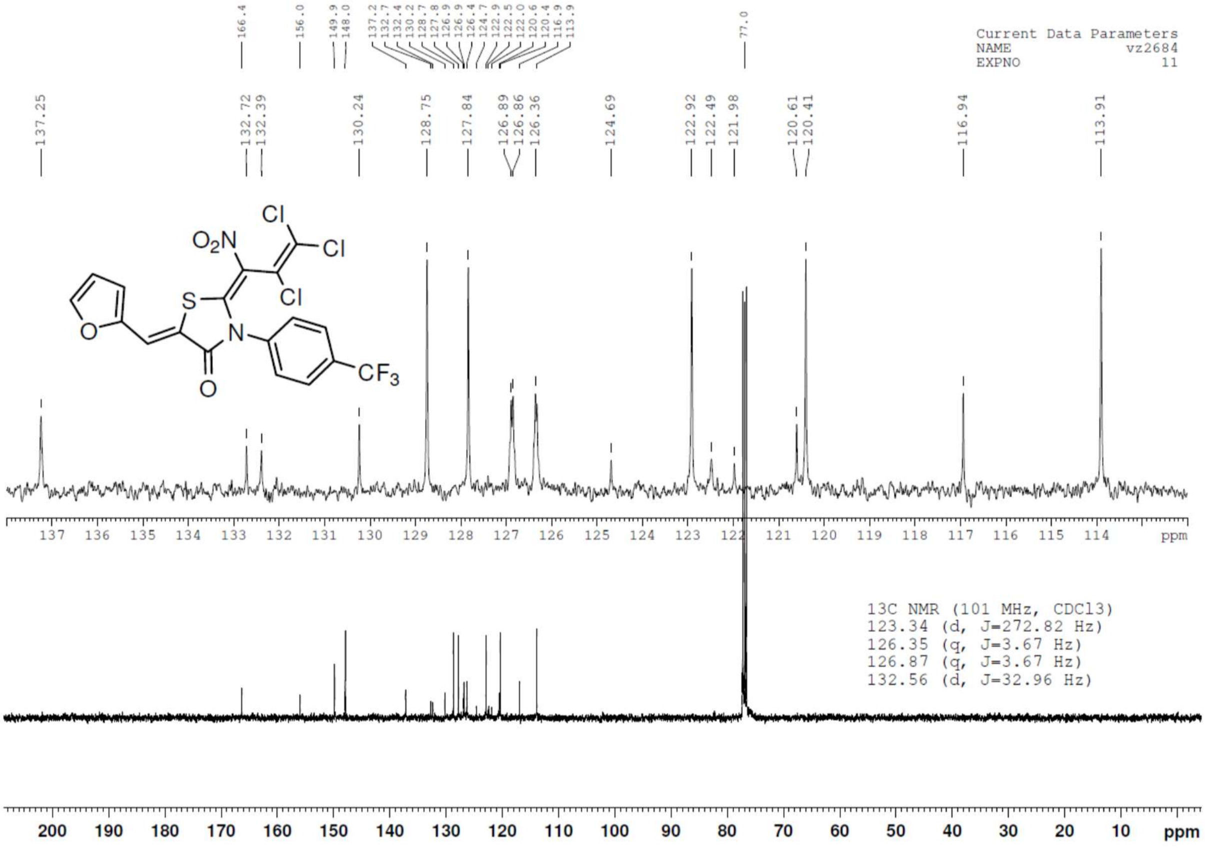

Figure S71. 400 MHz ^{1}H-NMR spectrum in $CDCl_3$ for **21e.**

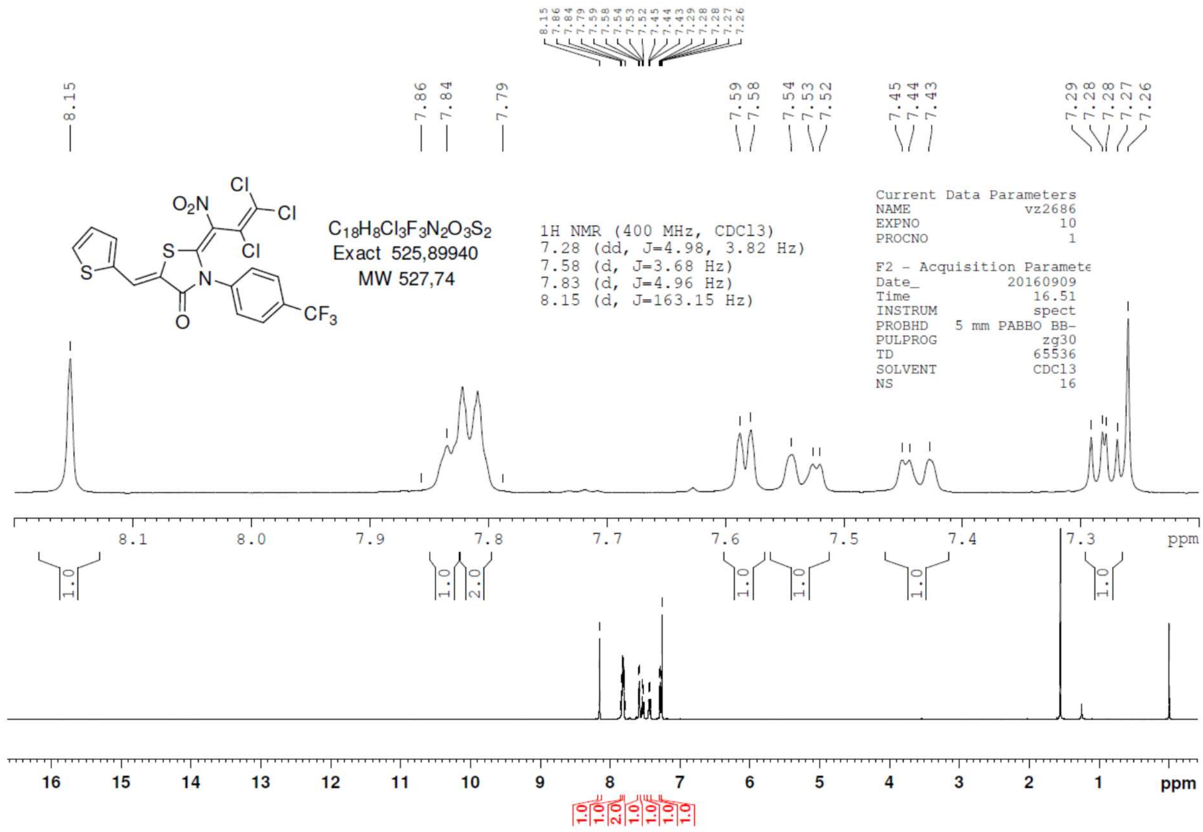

Figure S72. 100 MHz ^{13}C-NMR spectrum in $CDCl_3$ for **21e**.

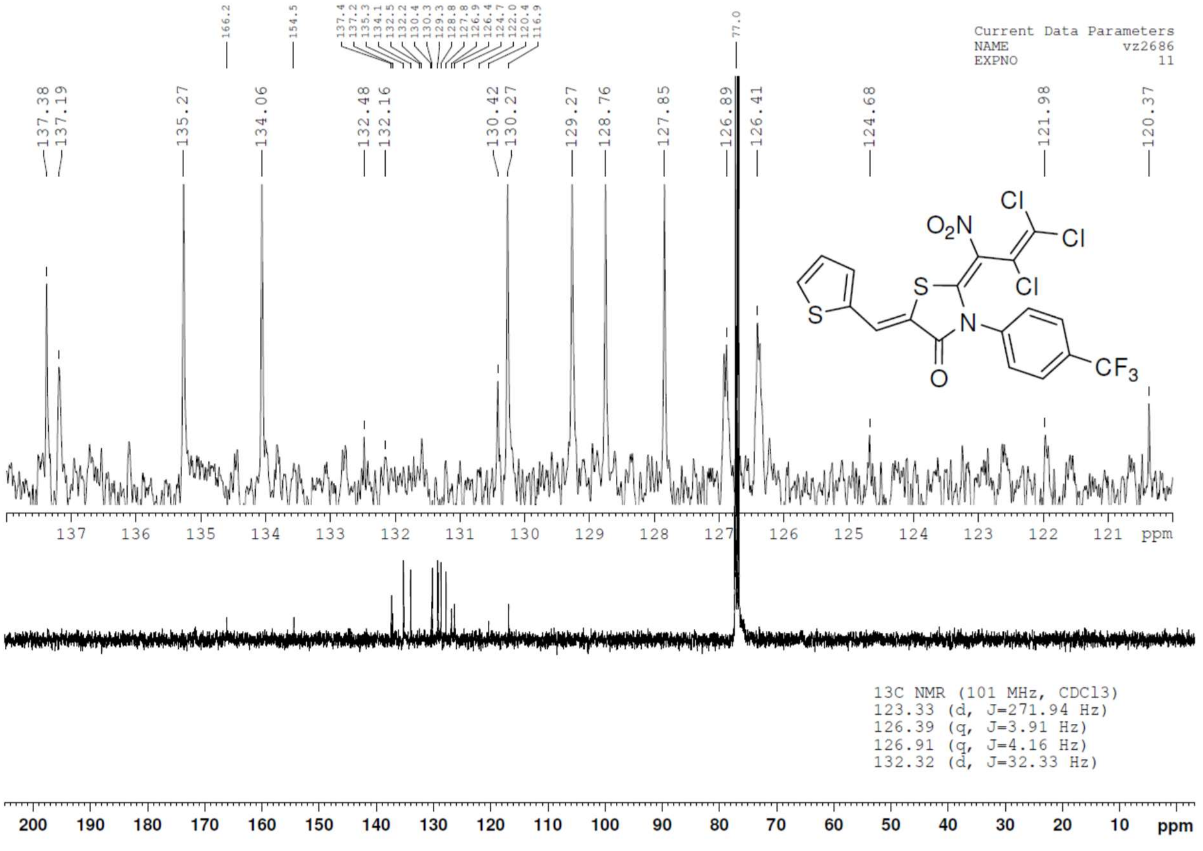

Figure S73. 400 MHz ^{1}H-NMR spectrum in DMSO-*d6* for **21f**.

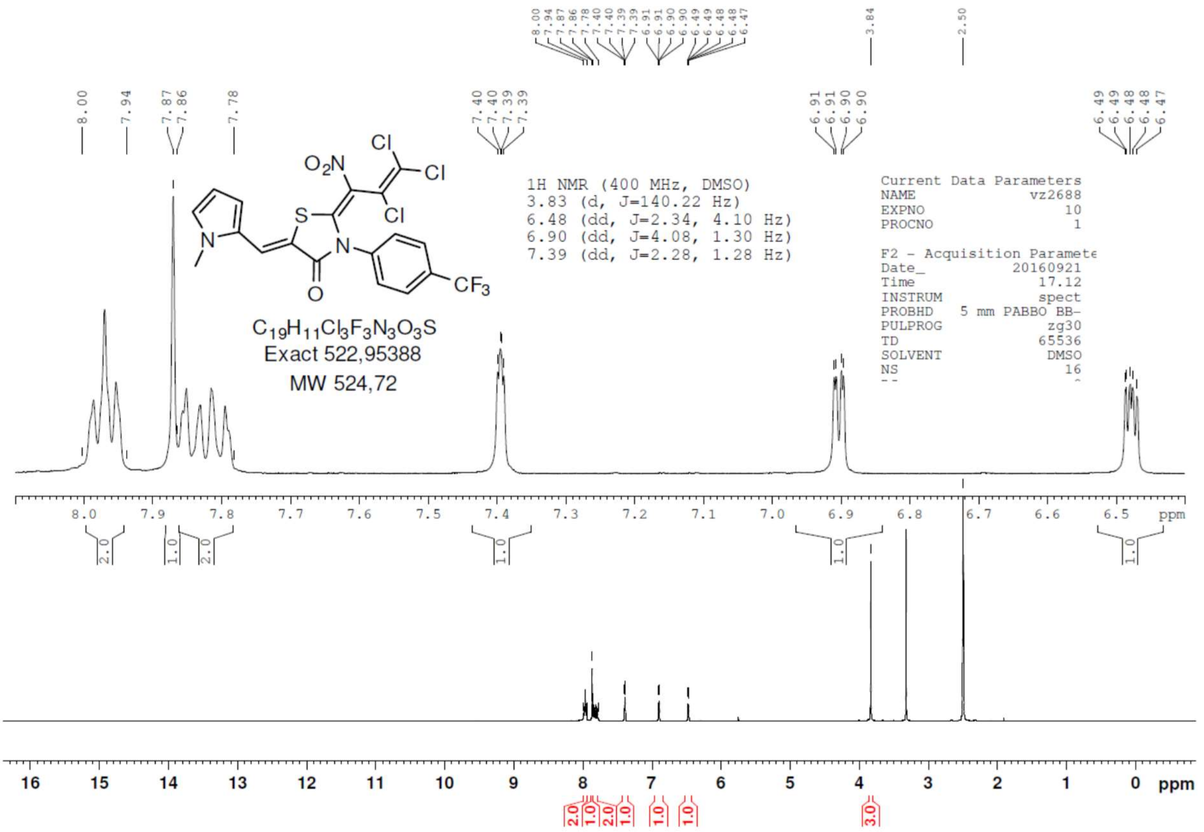

Figure S74. 100 MHz ^{13}C-NMR spectrum in DMSO-*d6* for **21f**.

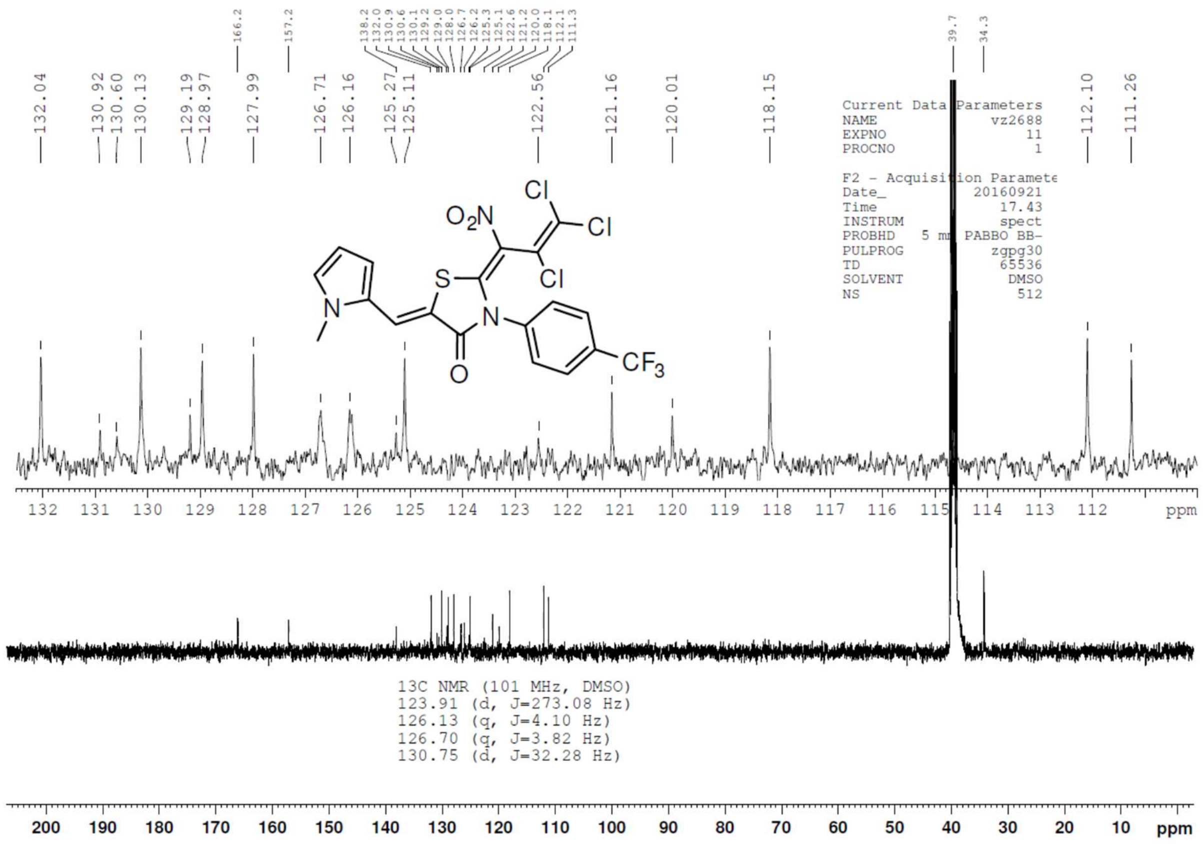

Figure S75. 400 MHz ^{1}H-NMR spectrum in DMSO-*d6* for **23a**.

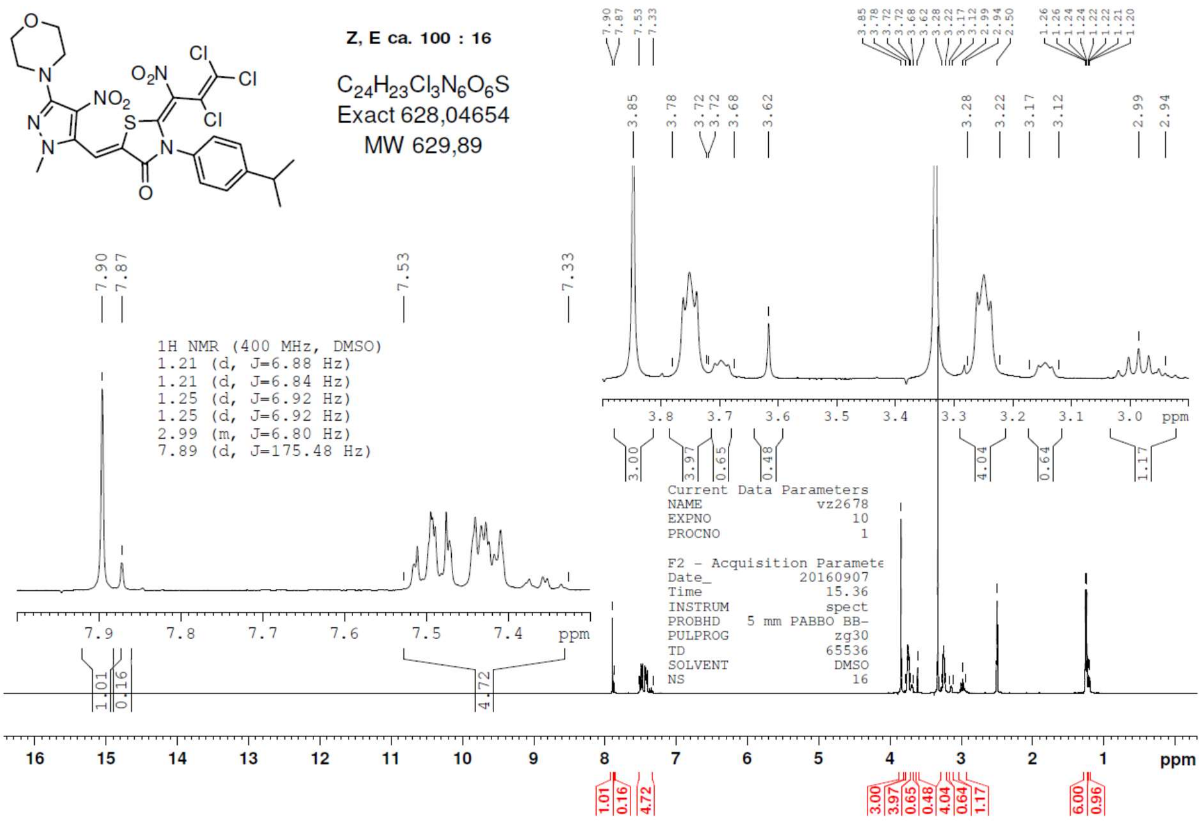

Figure S76. 100 MHz ^{13}C-NMR spectrum in DMSO-*d6* for **23a**.

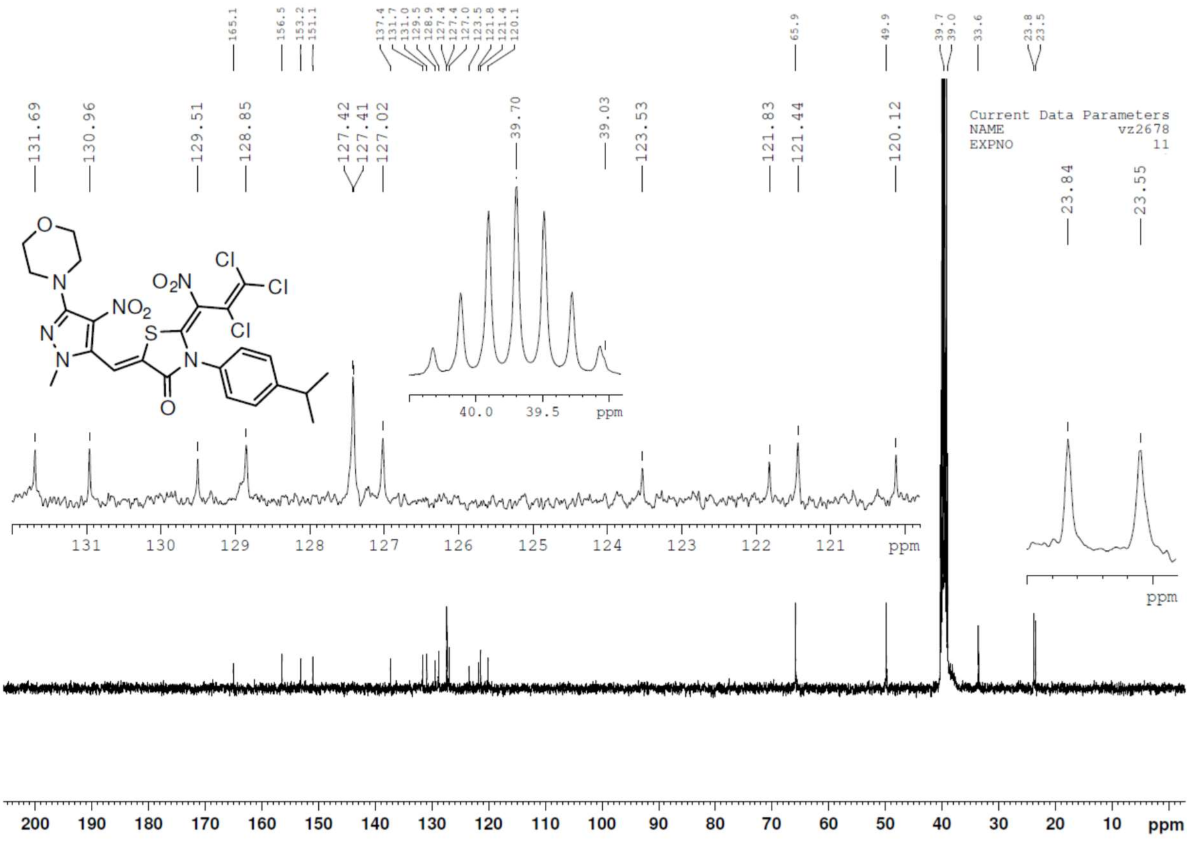

Figure S77. 400 MHz ^{1}H-NMR spectrum in DMSO-*d6* for **23b**.

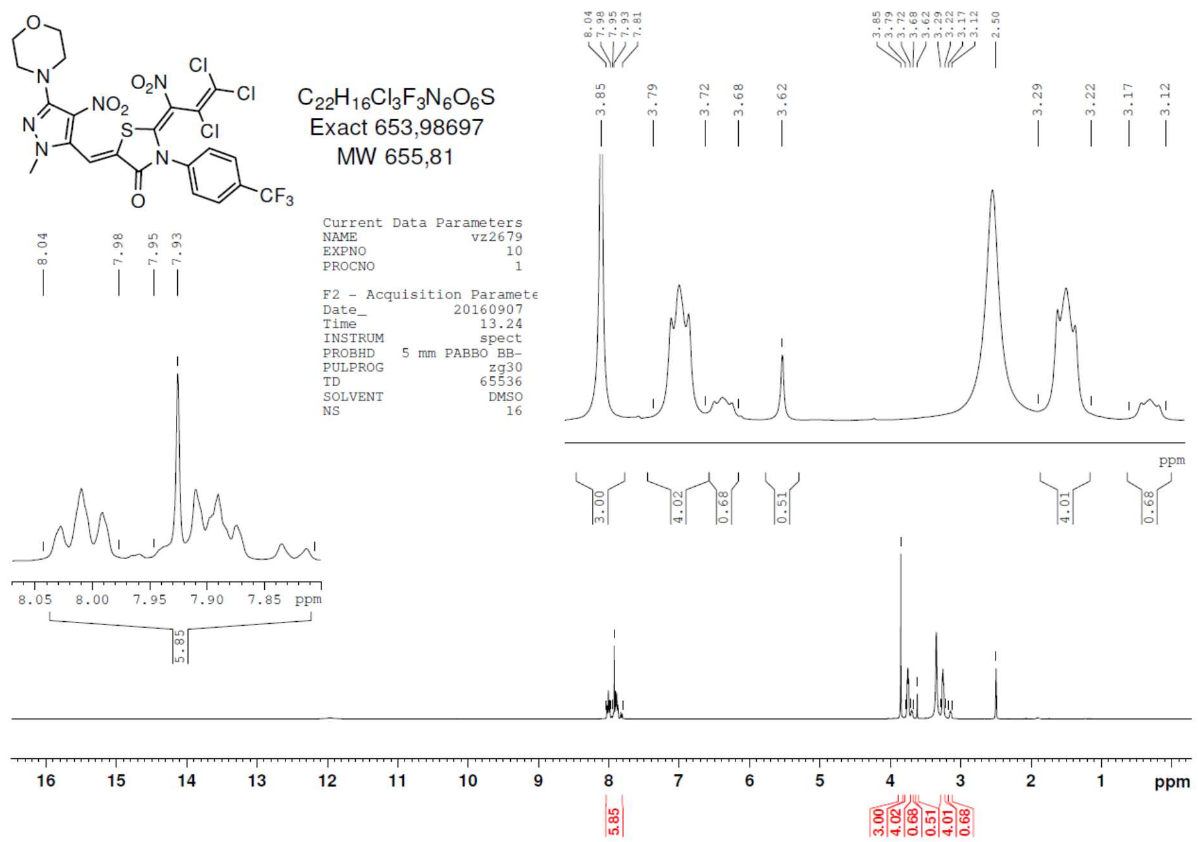

Figure S78. 100 MHz ^{13}C-NMR spectrum in DMSO-*d6* for **23b**.

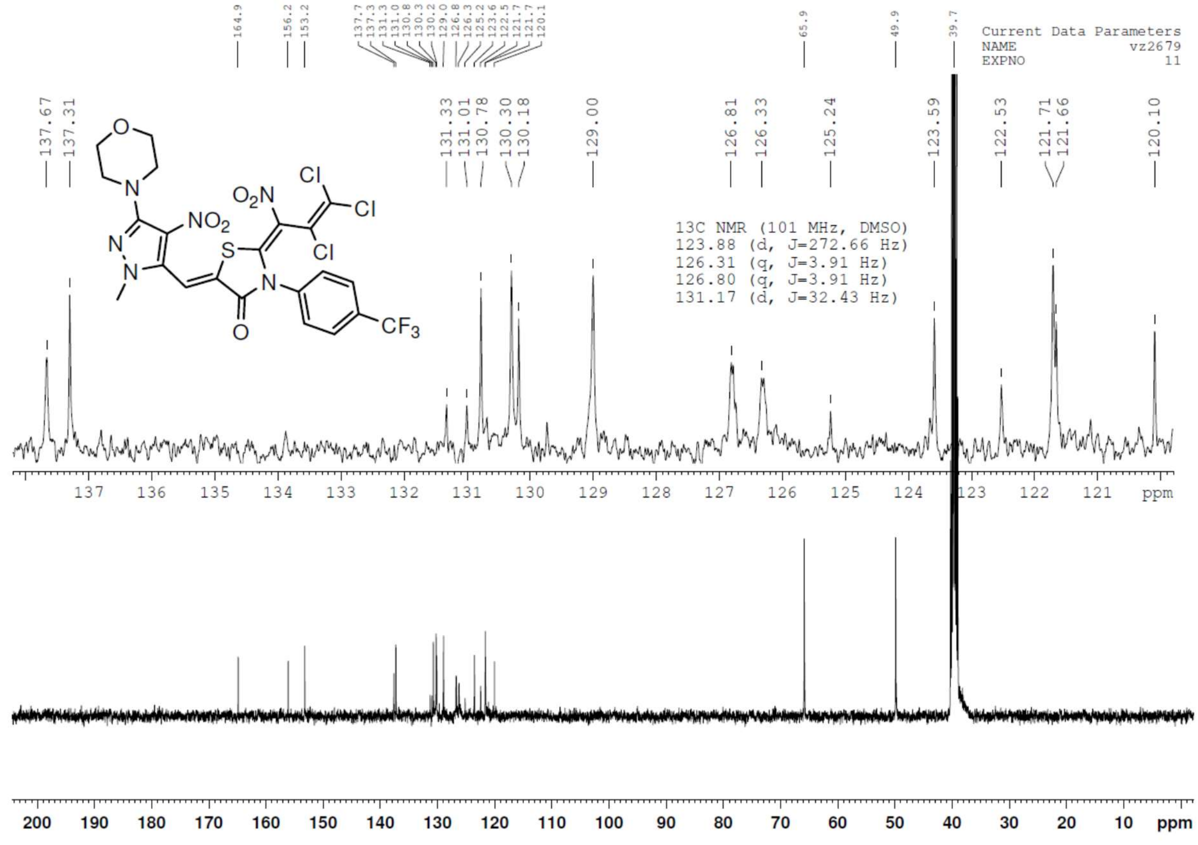

Figure S79. 200 MHz ^{1}H-NMR spectrum in $CDCl_3$ for **25a.**

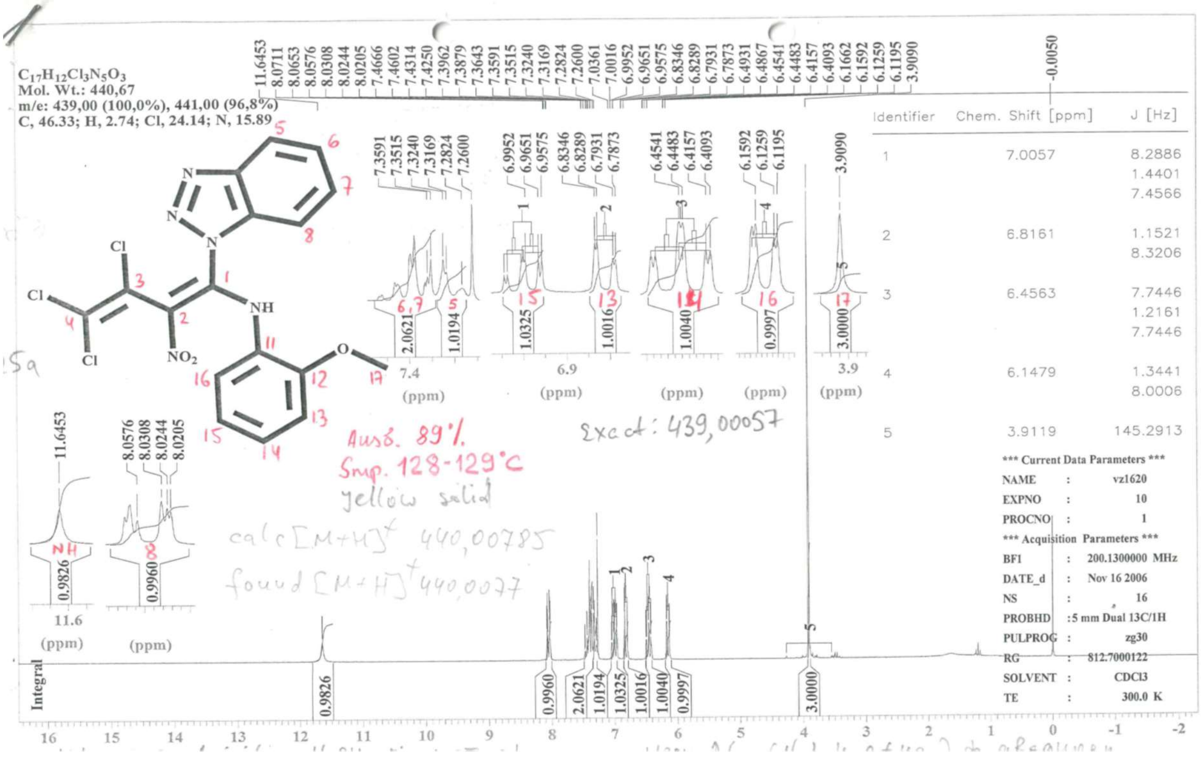

Figure S80. 50 MHz ^{13}C-NMR spectrum in $CDCl_3$ for **25a.**

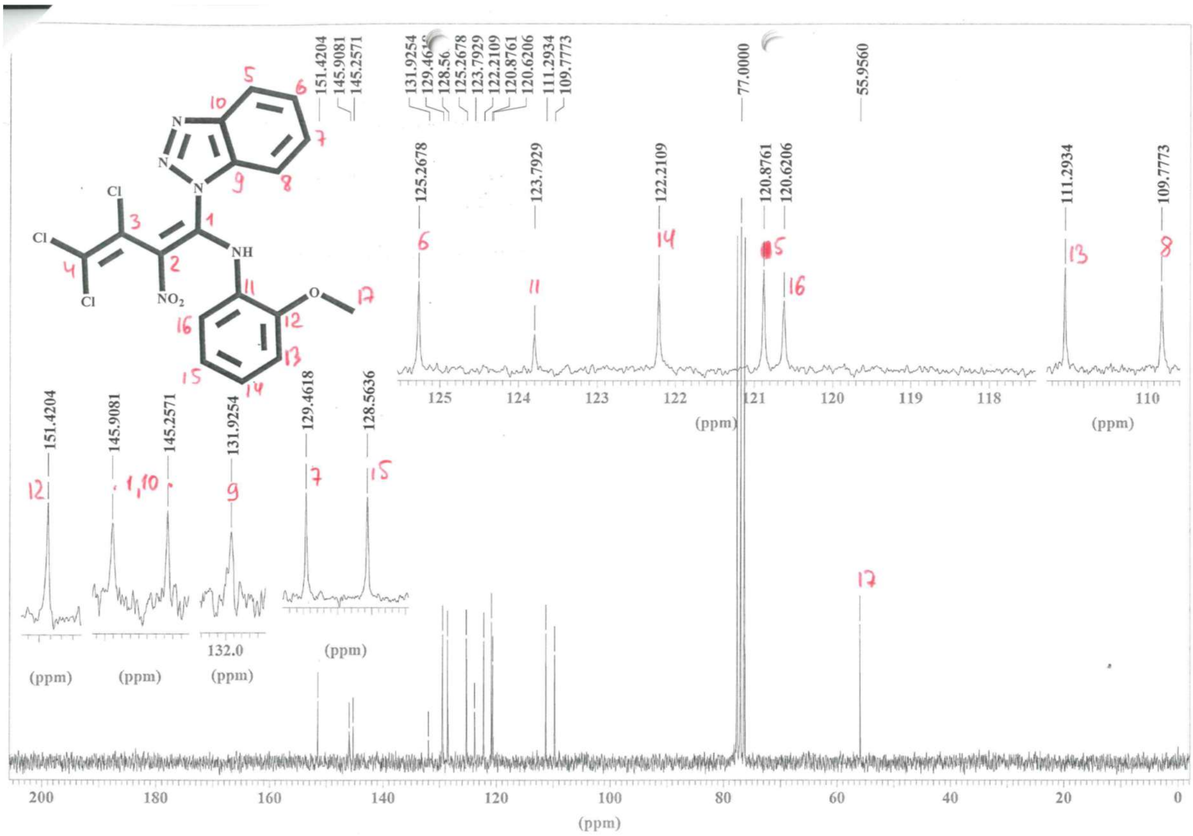

Figure S81. 400 MHz ^{1}H-NMR spectrum in $CDCl_3$ for **25b.**

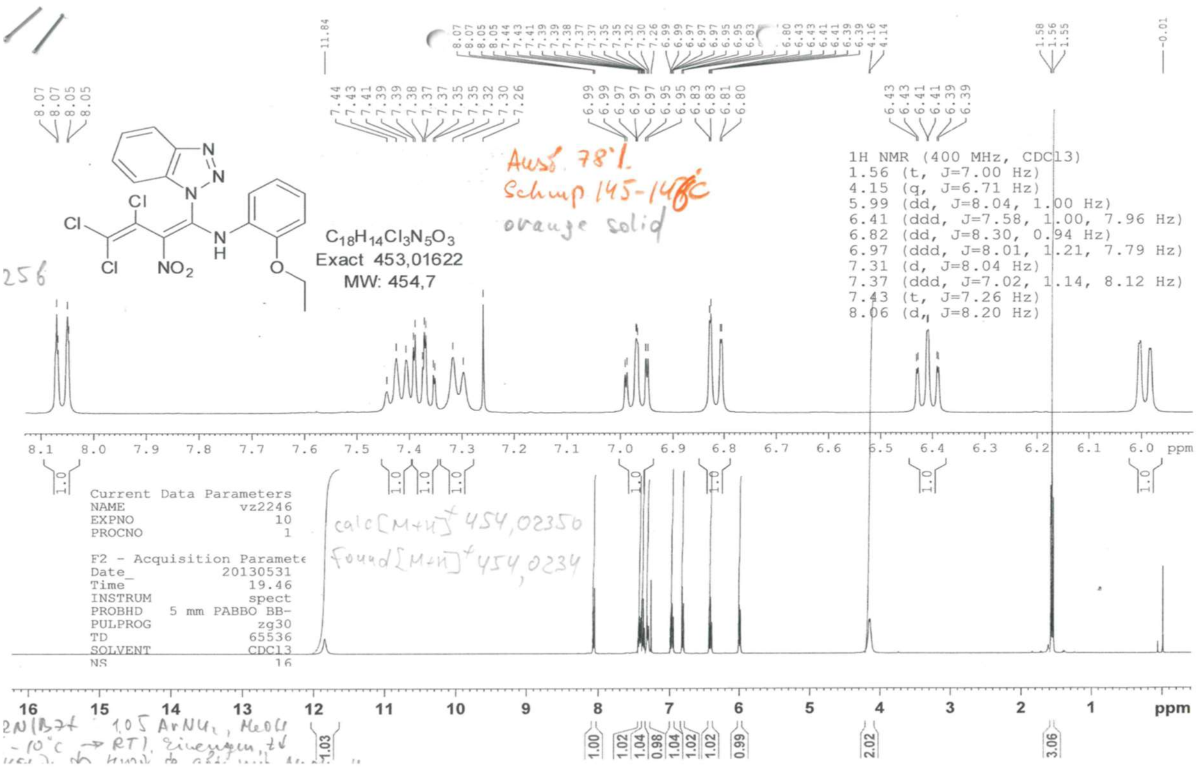

Figure S82. 100 MHz ^{13}C-NMR spectrum in $CDCl_3$ for **25b.**

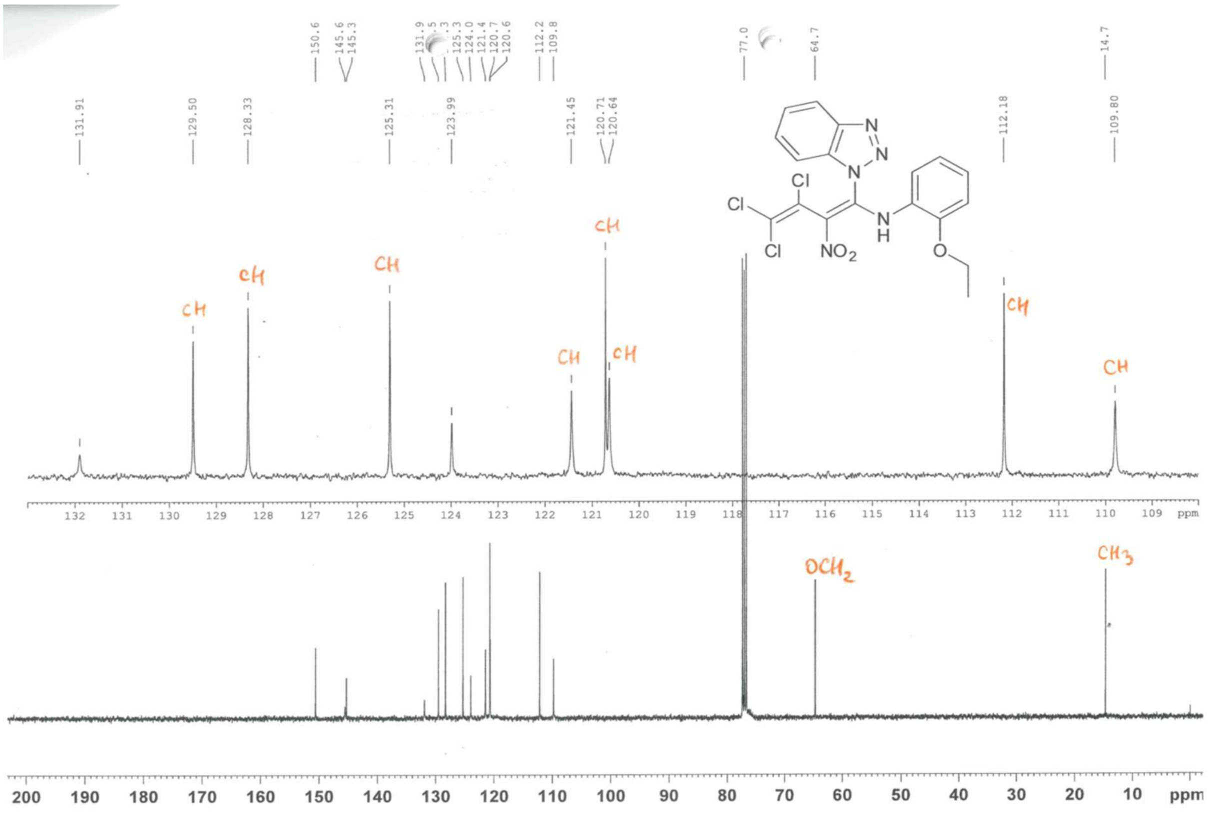

Figure S83. 400 MHz ^{1}H-NMR spectrum in $CDCl_3$ for **25c.**

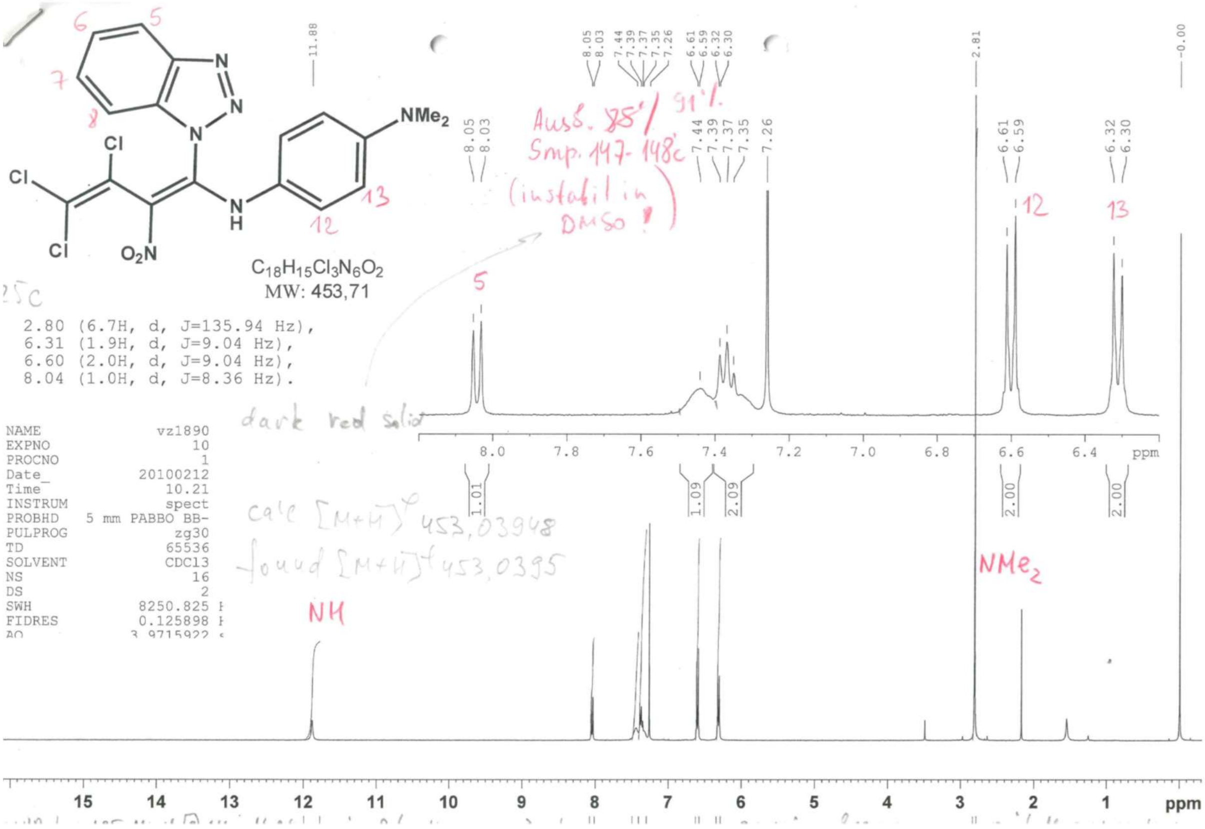

Figure S84. 100 MHz ^{13}C-NMR spectrum in $CDCl_3$ for **25c.**

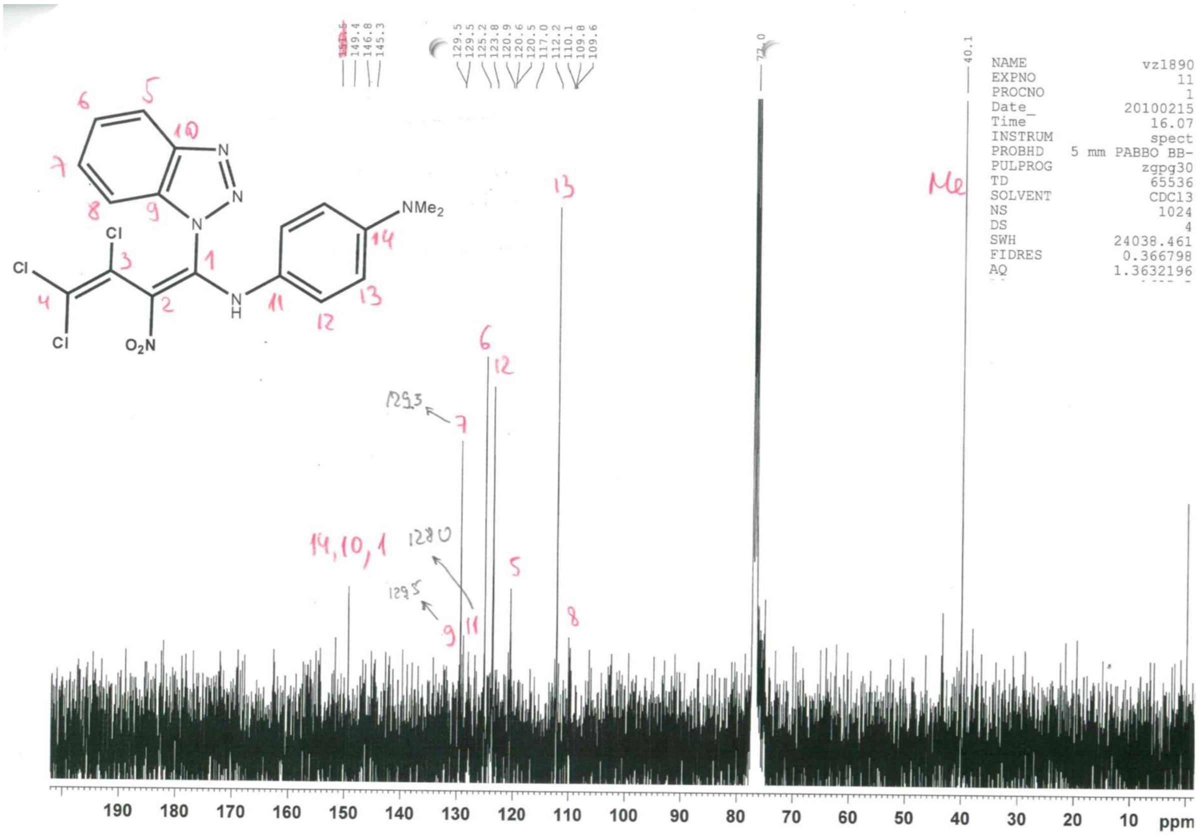

Figure S85. 400 MHz ^{1}H-NMR spectrum in $CDCl_3$ for **25f**.

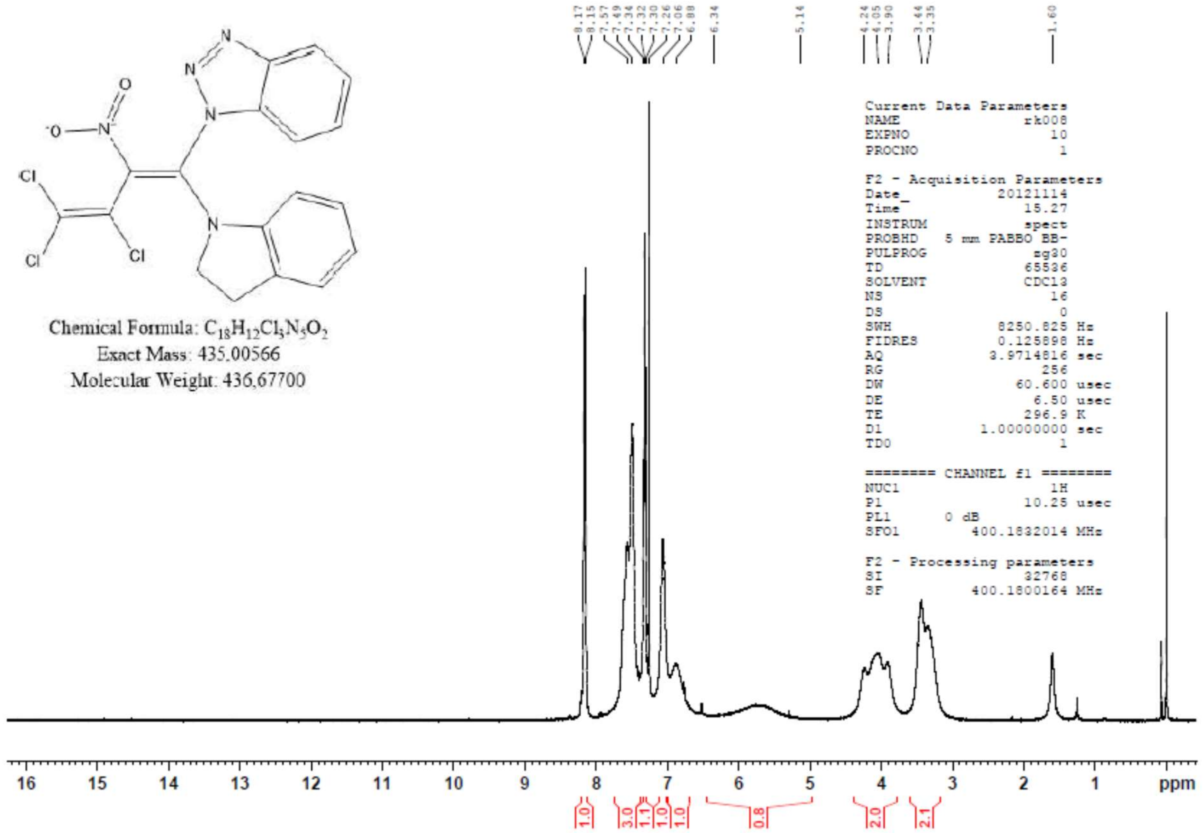

Figure S86. 100 MHz ^{13}C-NMR spectrum in $CDCl_3$ for **25f**.

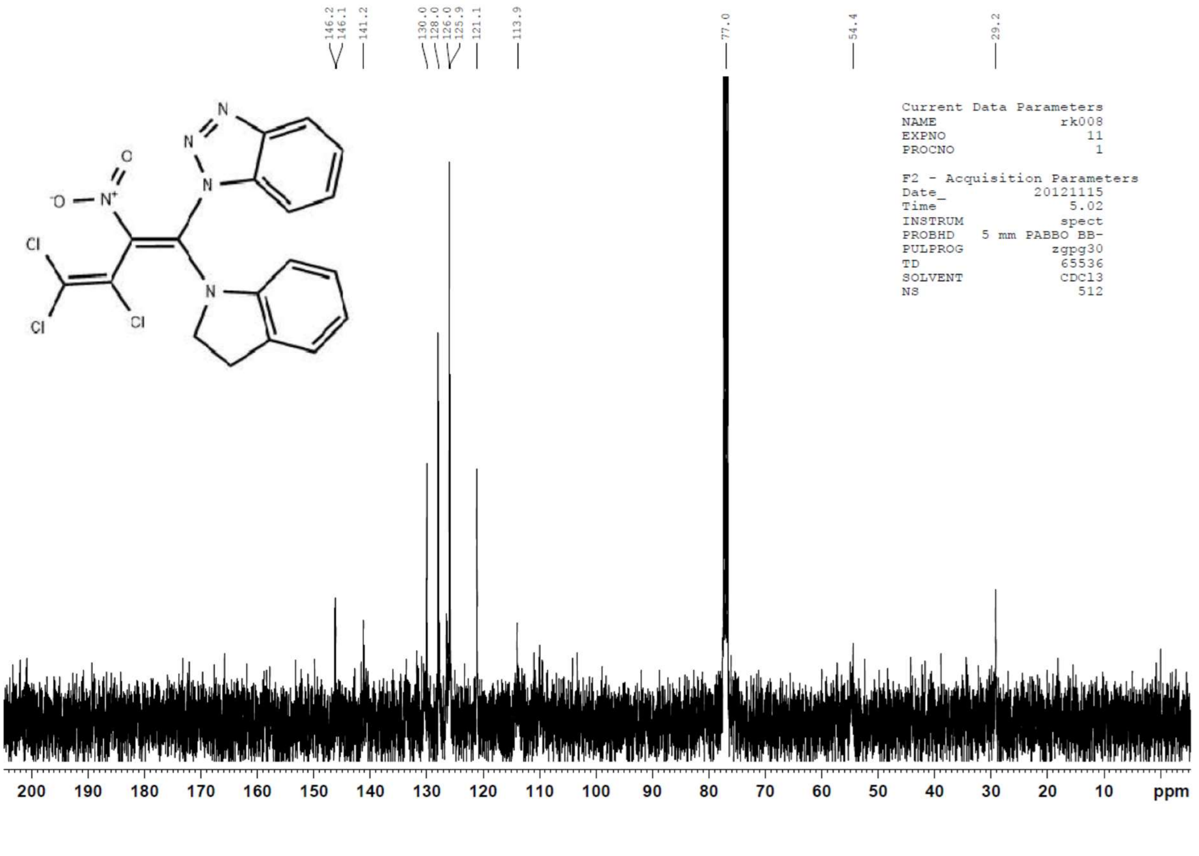

Figure S87. 200 MHz ^{1}H-NMR spectrum in DMSO-*d6* for **26a**.

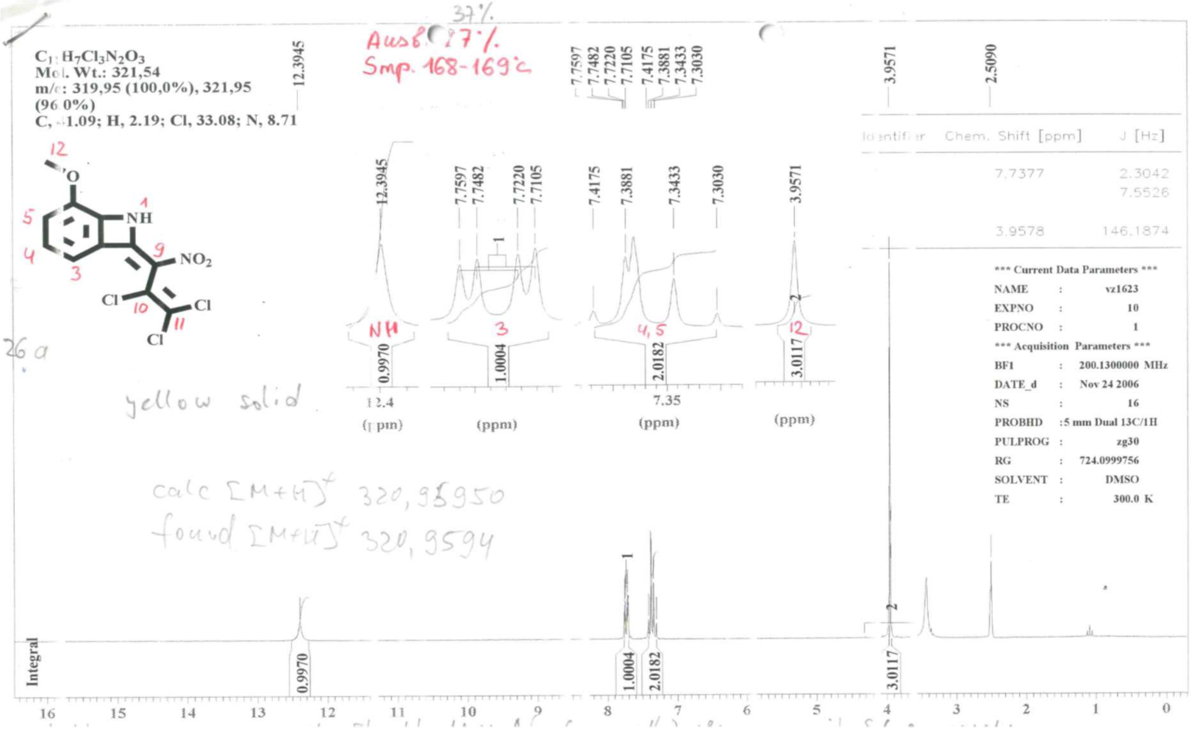

Figure S88. 50 MHz ^{13}C-NMR spectrum in DMSO-*d6* for **26a**.

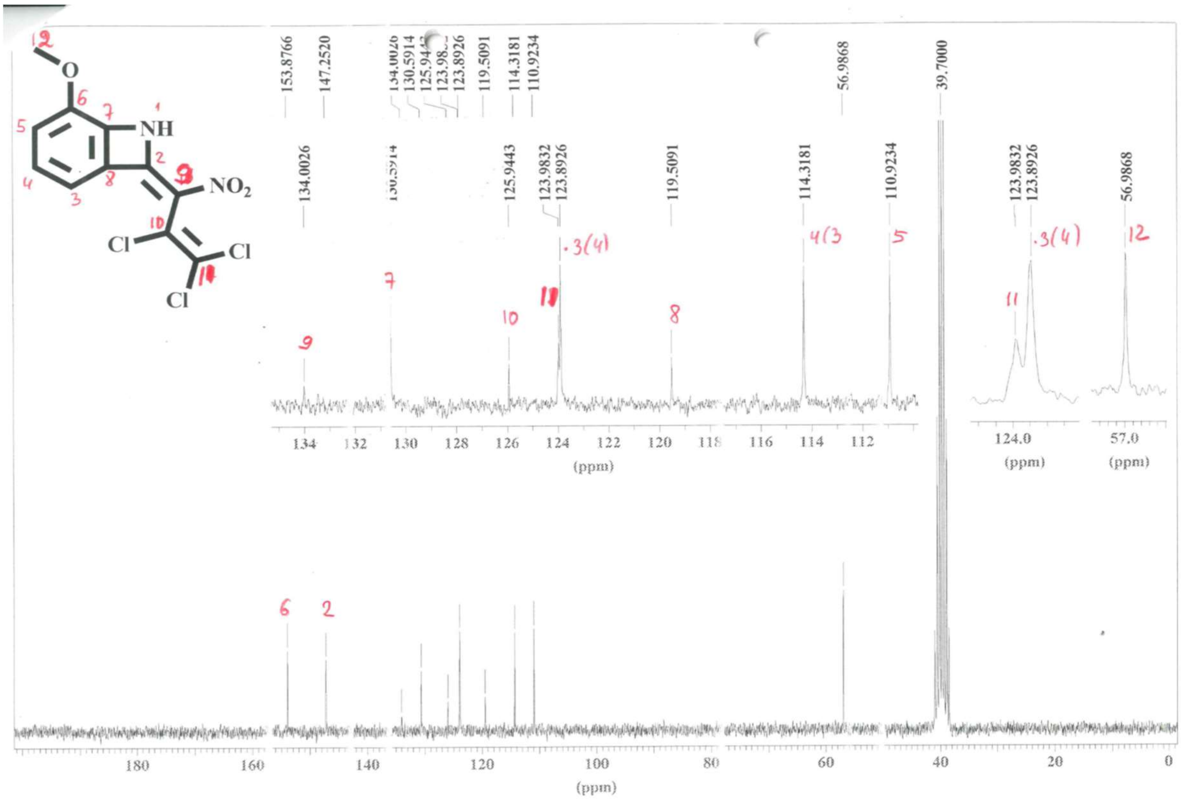

Figure S89. 400 MHz ^{1}H-NMR spectrum in DMSO-*d6* for **26b**.

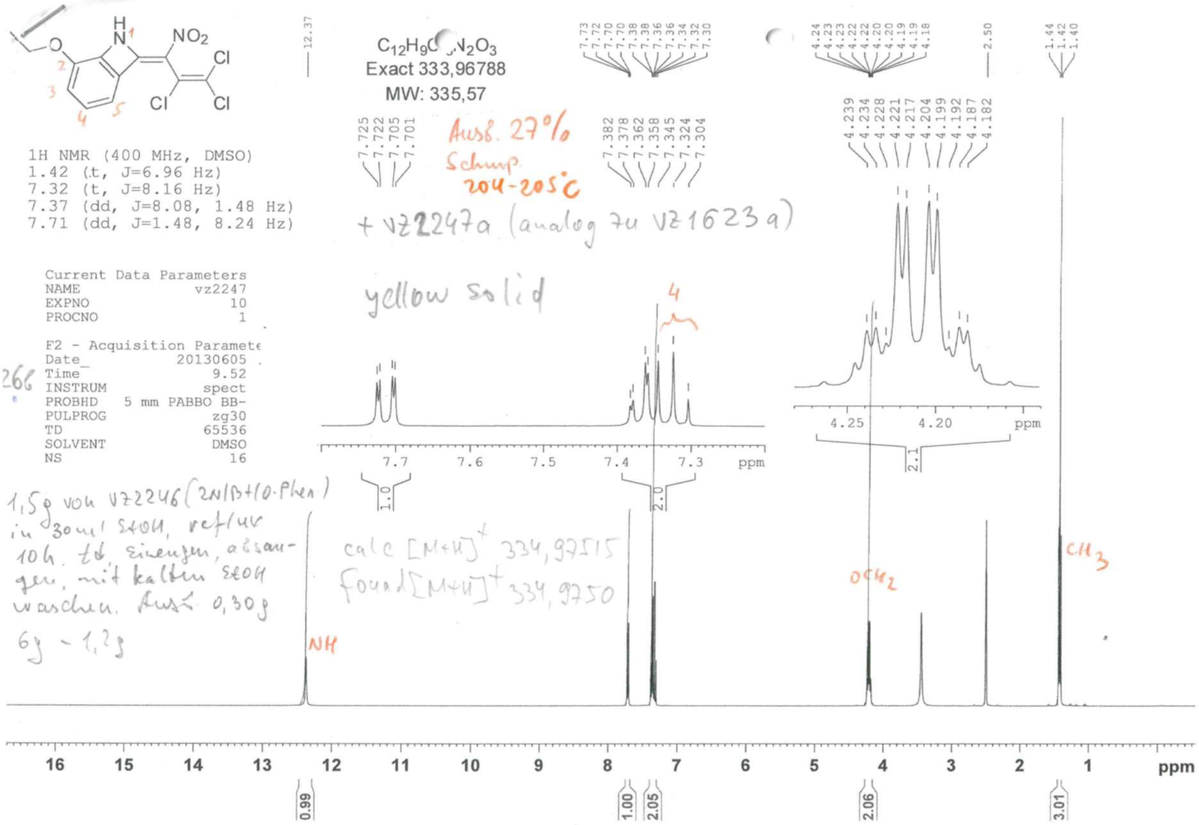

Figure S90. 100 MHz ^{13}C-NMR spectrum in DMSO-*d6* for **26b**.

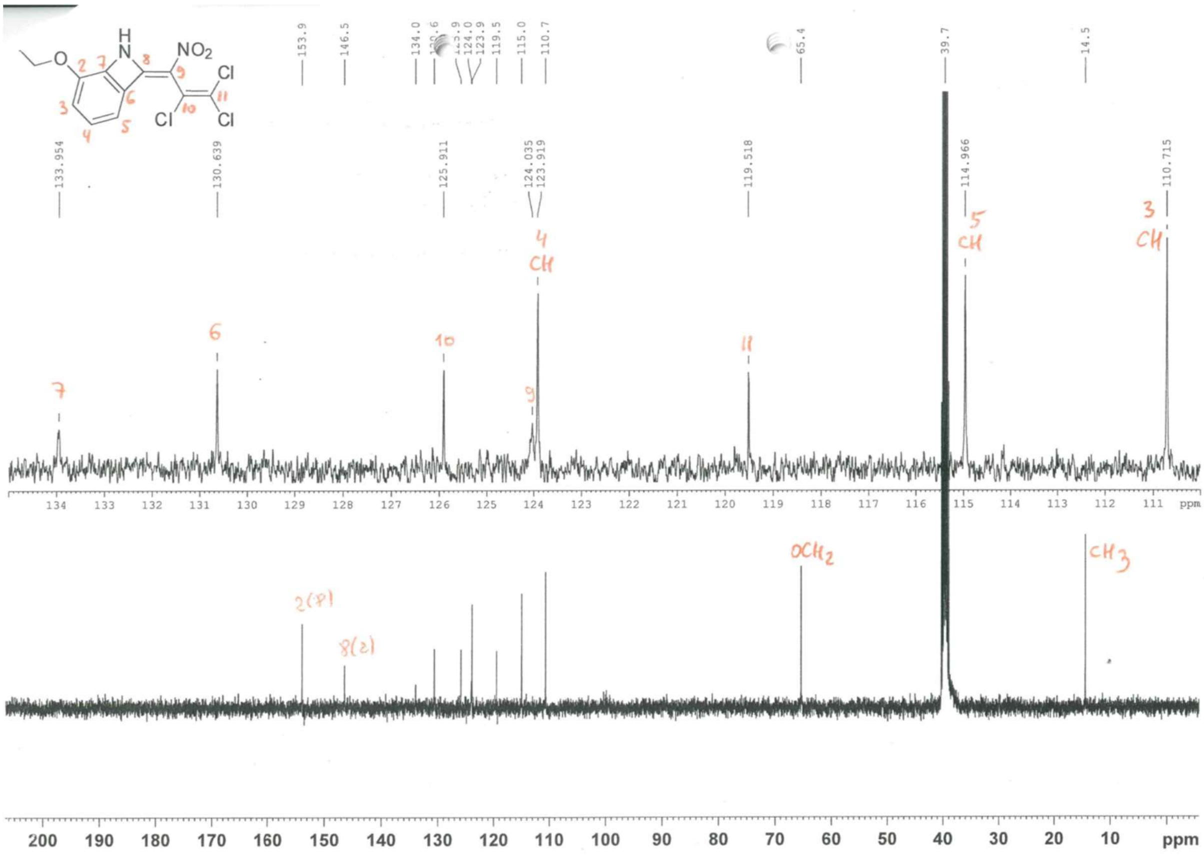

Figure S91. 400 MHz ^{1}H-NMR spectrum in DMSO-*d6* for **26c**.

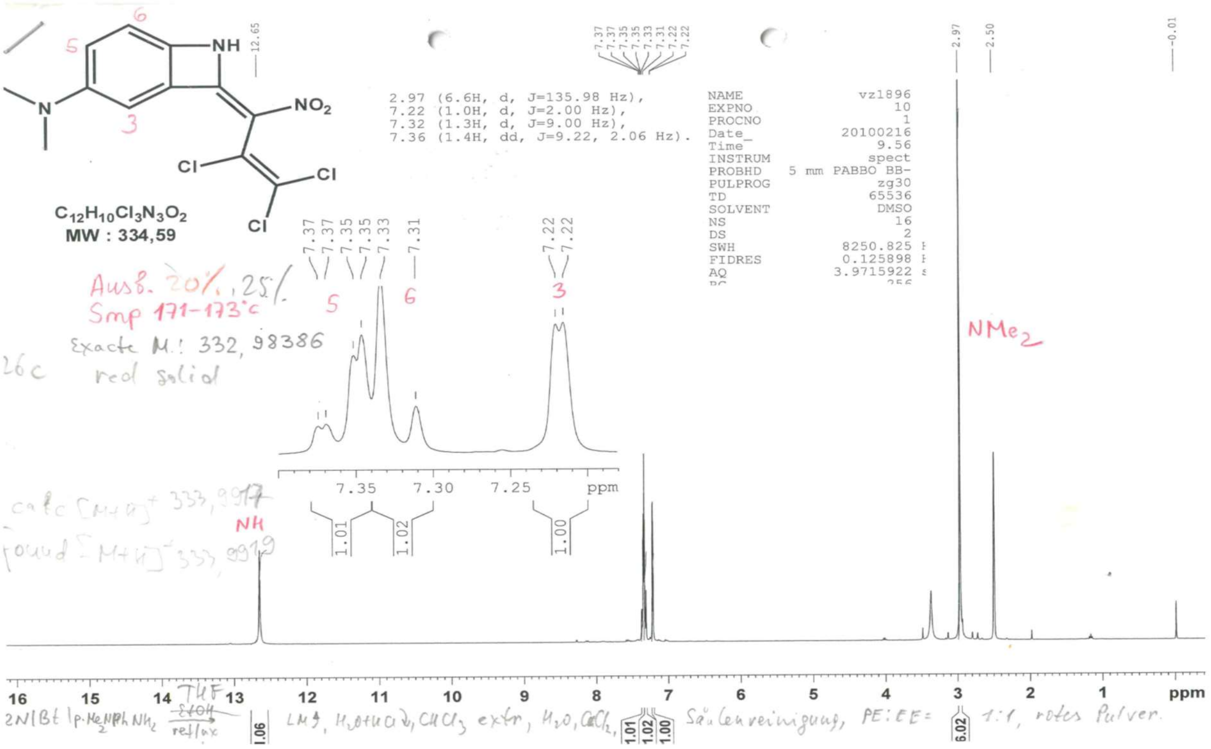

Figure S92. 100 MHz ^{13}C-NMR spectrum in DMSO-*d6* for **26c**.

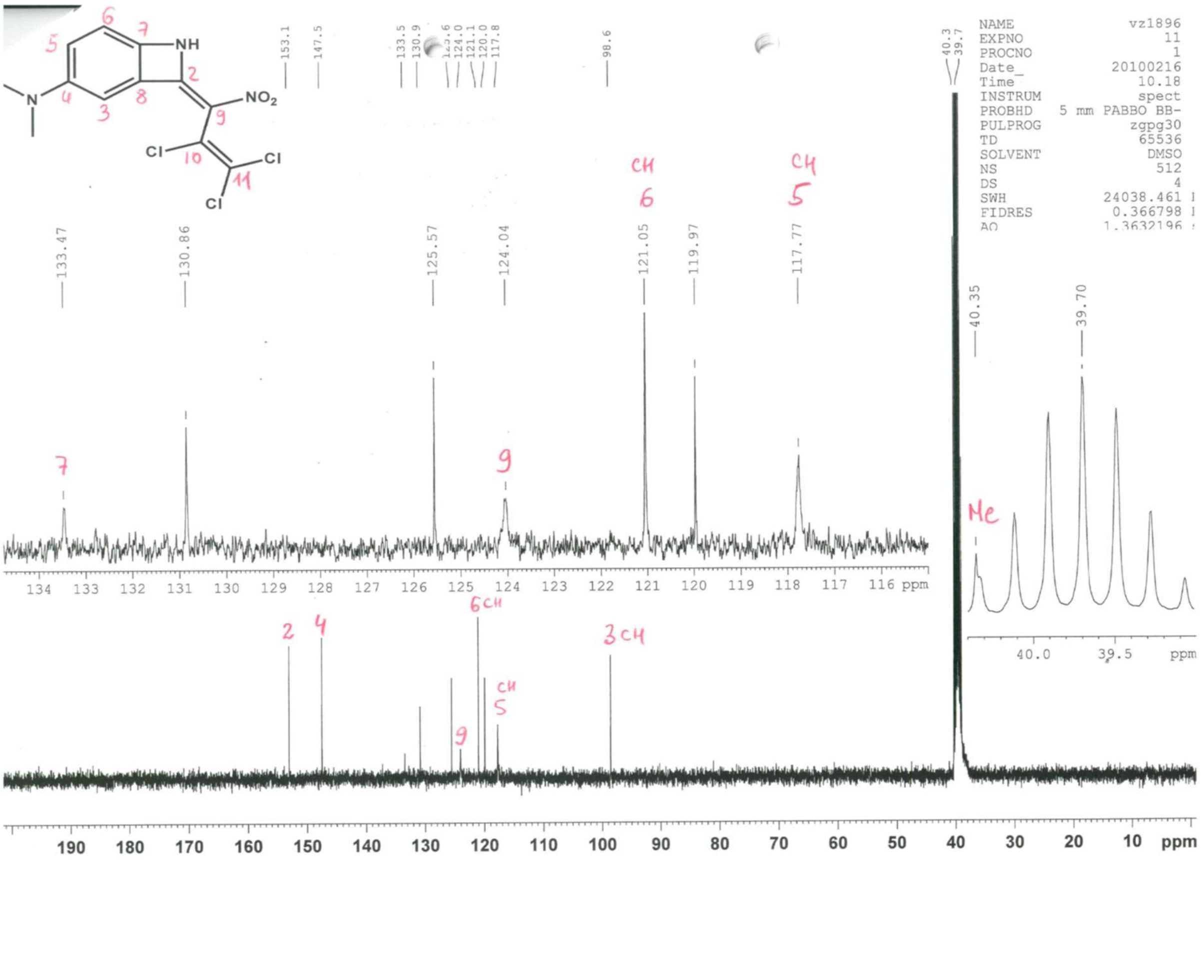

Figure S93. HR-MS spectrum for *N*,*N*-Dimethyl-8-(2,3,3-trichloro-1-nitroprop-2-en-1-ylidene)-7-azabicyclo[4.2.0]octa-1,3,5- trien-3-amine (**26c**).

Elemental Composition Report **Page 1**

Single Mass Analysis
Tolerance = 6.0 mDa / DBE: min = -1.5, max = 50.0
Element prediction: Off
Number of isotope peaks used for i-FIT = 3

Monoisotopic Mass, Odd and Even Electron Ions
686 formula(e) evaluated with 23 results within limits (up to 30 closest results for each mass)
Elements Used:
C: 0-70 H: 0-90 N: 0-5 O: 0-3 Na: 0-1 Cl: 0-3
Zapolski,TUC Q-Tof Premier UPLC-MS 24-Jun-2010
10:22:39
VZ1896 241 (2.480) AM (Cen,5, 70.00, Ar,10000.0,556.28,0.70,LS 15) 1: TOF MS ES+
1.56e+003

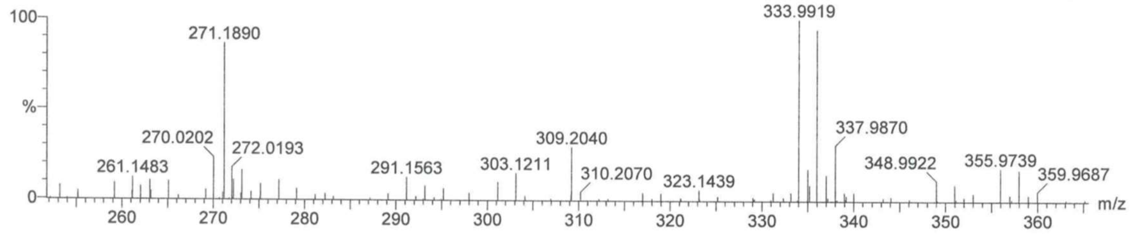

Minimum:				-1.5		
Maximum:		6.0	100.0	50.0		
Mass	Calc. Mass	mDa	PPM	DBE	i-FIT	Formula
333.9919	333.9920	-0.1	-0.3	21.5	300.9	C19 H N5 Cl
	333.9917	0.2	0.6	7.5	3.6	C12 H11 N3 O2 Cl3
	333.9915	0.4	1.2	13.5	54.7	C16 H7 N3 Na Cl2
	333.9912	0.7	2.1	12.0	49.0	C15 H8 N2 O3 Cl2
	333.9928	-0.9	-2.7	13.0	58.4	C18 H9 O Na Cl2
	333.9910	0.9	2.7	18.0	293.4	C19 H4 N2 O Na Cl
	333.9929	-1.0	-3.0	25.5	717.7	C24 N O2
	333.9930	-1.1	-3.3	7.0	1.7	C14 H13 O3 Cl3
	333.9906	1.3	3.9	4.0	6.6	C12 H14 O3 Na Cl3
	333.9933	-1.4	-4.2	8.5	0.6	C15 H12 N Na Cl3
	333.9905	1.4	4.2	22.5	711.0	C22 H N O2 Na
	333.9934	-1.5	-4.5	21.0	306.4	C21 H3 N2 O Cl
	333.9939	-2.0	-6.0	16.5	64.0	C18 H6 N3 Cl2
	333.9899	2.0	6.0	12.5	48.4	C13 H6 N5 O2 Cl2
	333.9896	2.3	6.9	18.5	290.1	C17 H2 N5 Na Cl
	333.9894	2.5	7.5	17.0	279.0	C16 H3 N4 O3 Cl
	333.9893	2.6	7.8	4.5	11.2	C10 H12 N3 O2 Na Cl3
	333.9888	3.1	9.3	9.0	47.7	C13 H9 N2 O3 Na Cl2
	333.9952	-3.3	-9.9	16.0	70.2	C20 H8 O Cl2
	333.9957	-3.8	-11.4	11.5	3.3	C17 H11 N Cl3
	333.9874	4.5	13.5	9.5	50.3	C11 H7 N5 O2 Na Cl2
	333.9870	4.9	14.7	14.0	275.2	C14 H4 N4 O3 Na Cl
	333.9977	-5.8	-17.4	18.5	711.0	C16 H N5 O3 Na

Figure S94. 400 MHz ^{1}H-NMR spectrum in $CDCl_3$ for **27c.**

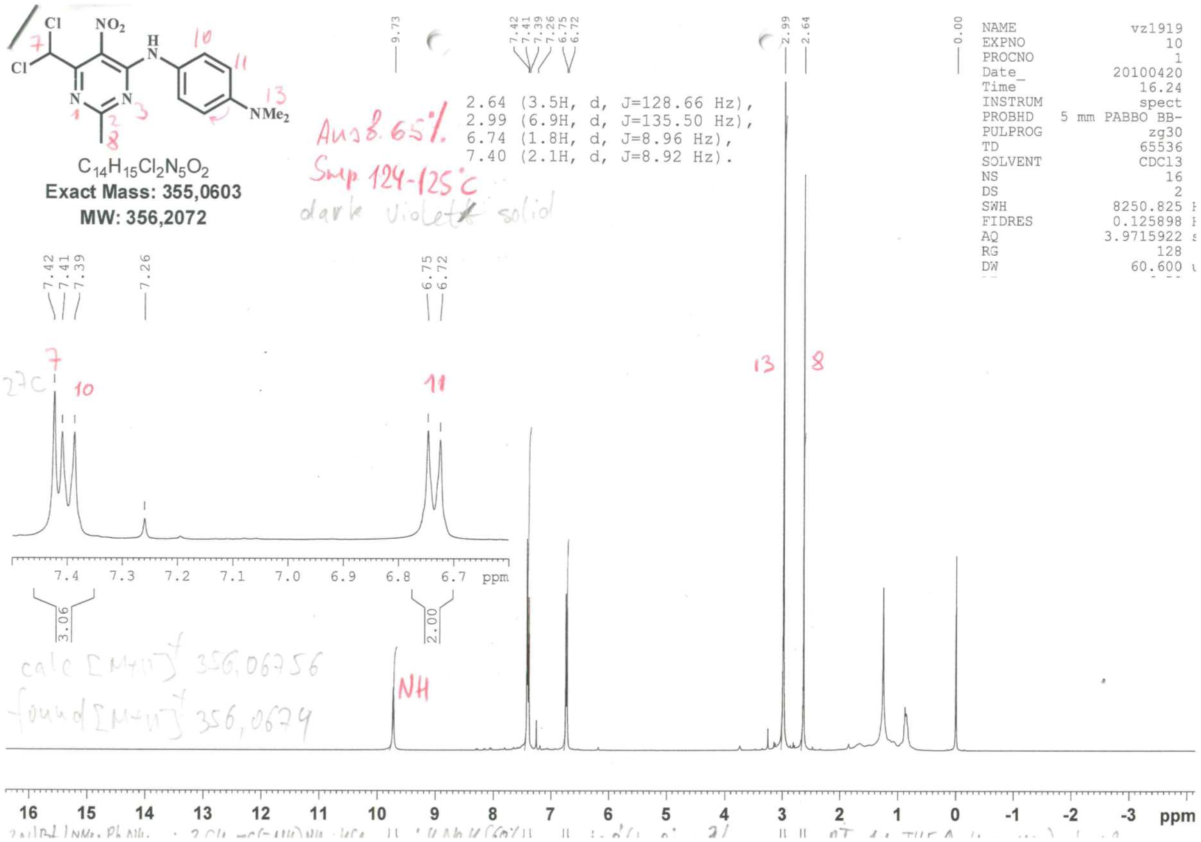

Figure S95. 100 MHz ^{13}C-NMR spectrum in $CDCl_3$ for **27c.**

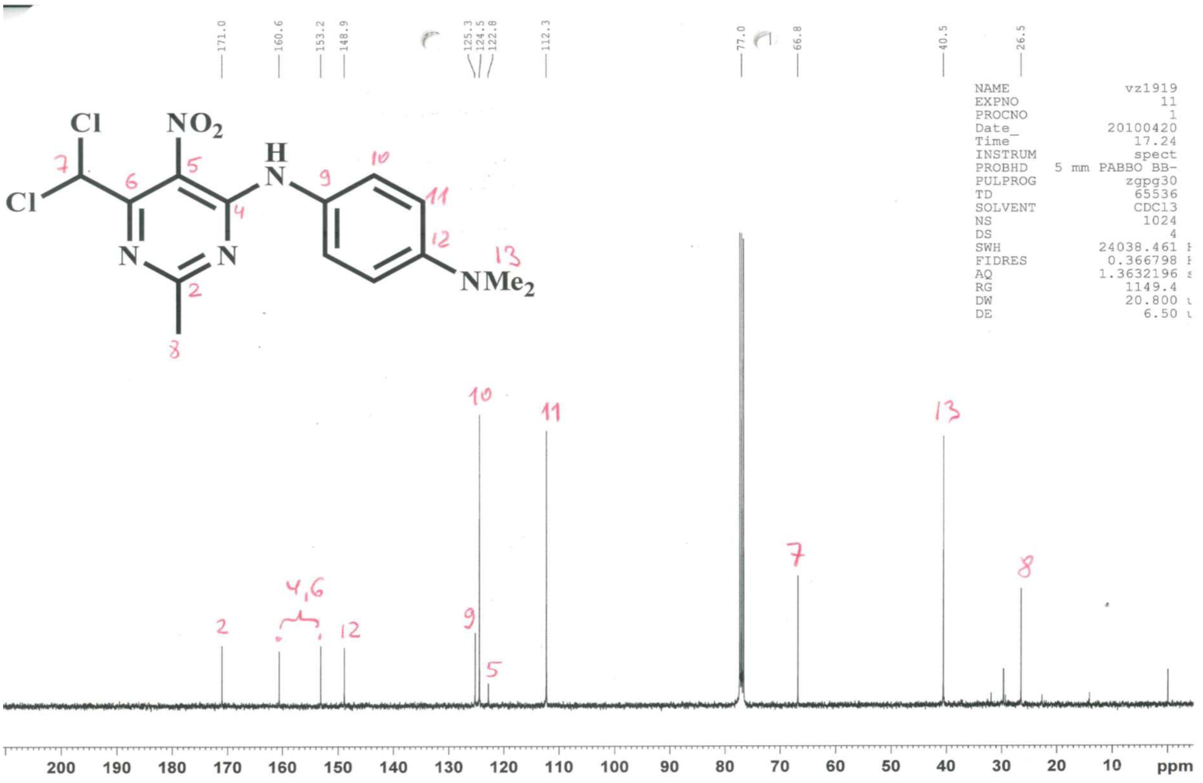

Figure S96. 400 MHz ^{1}H-NMR spectrum in $CDCl_3$ for **27d.**

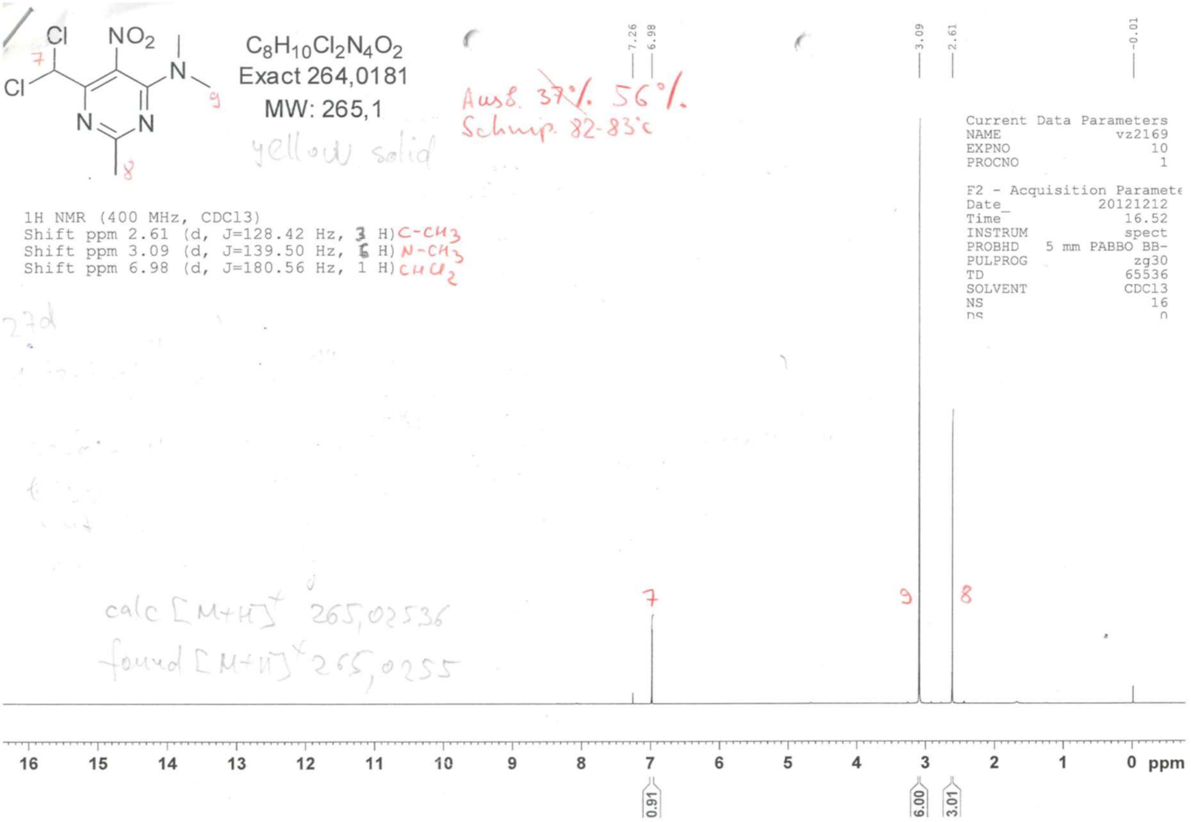

Figure S97. 100 MHz ^{13}C-NMR spectrum in $CDCl_3$ for **27d.**

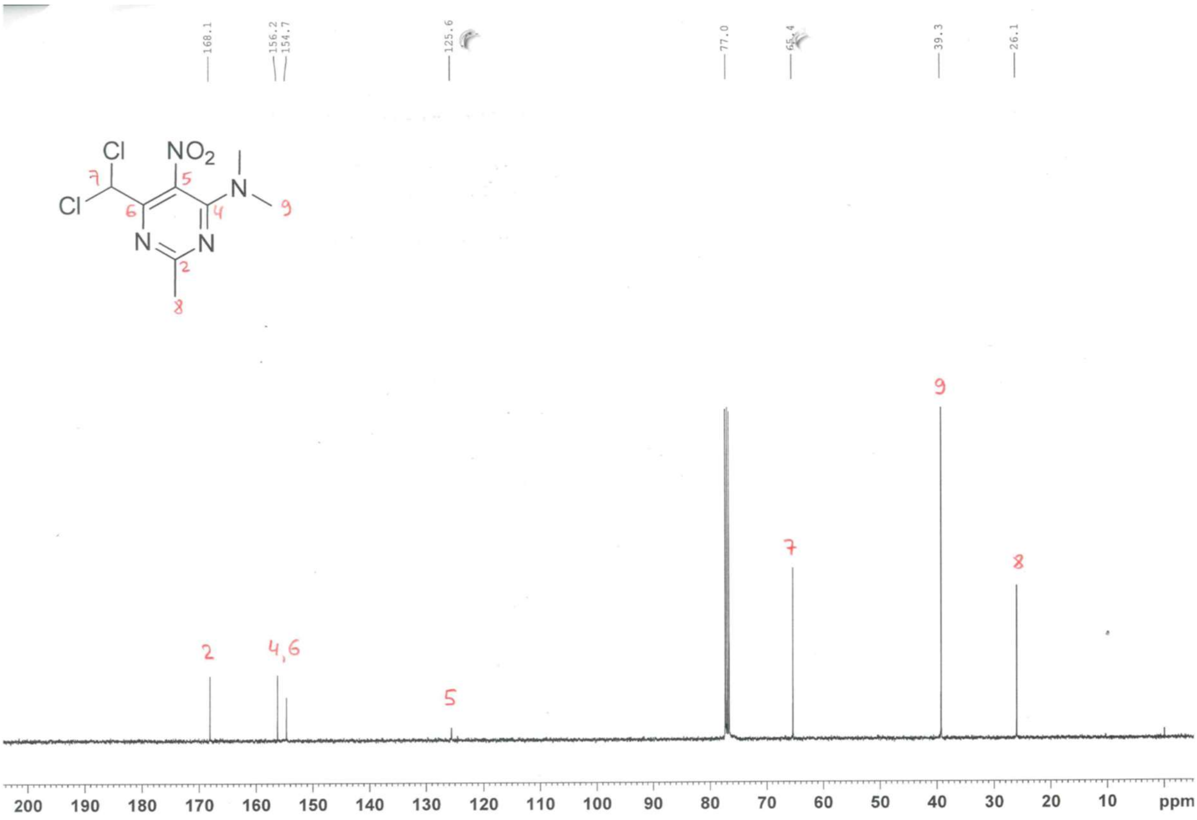

Figure S98. 400 MHz ^{1}H-NMR spectrum in $CDCl_3$ for **27e.**

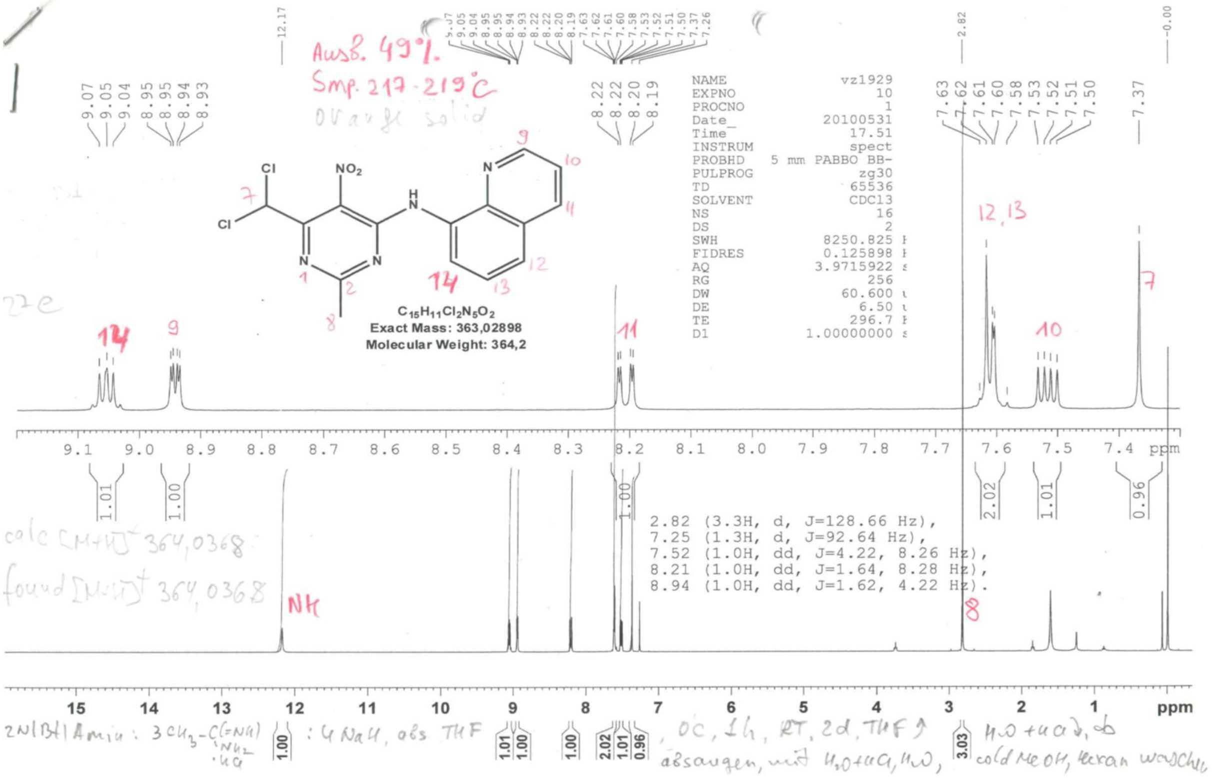

Figure S99. 100 MHz ^{13}C-NMR spectrum in $CDCl_3$ for **27e.**

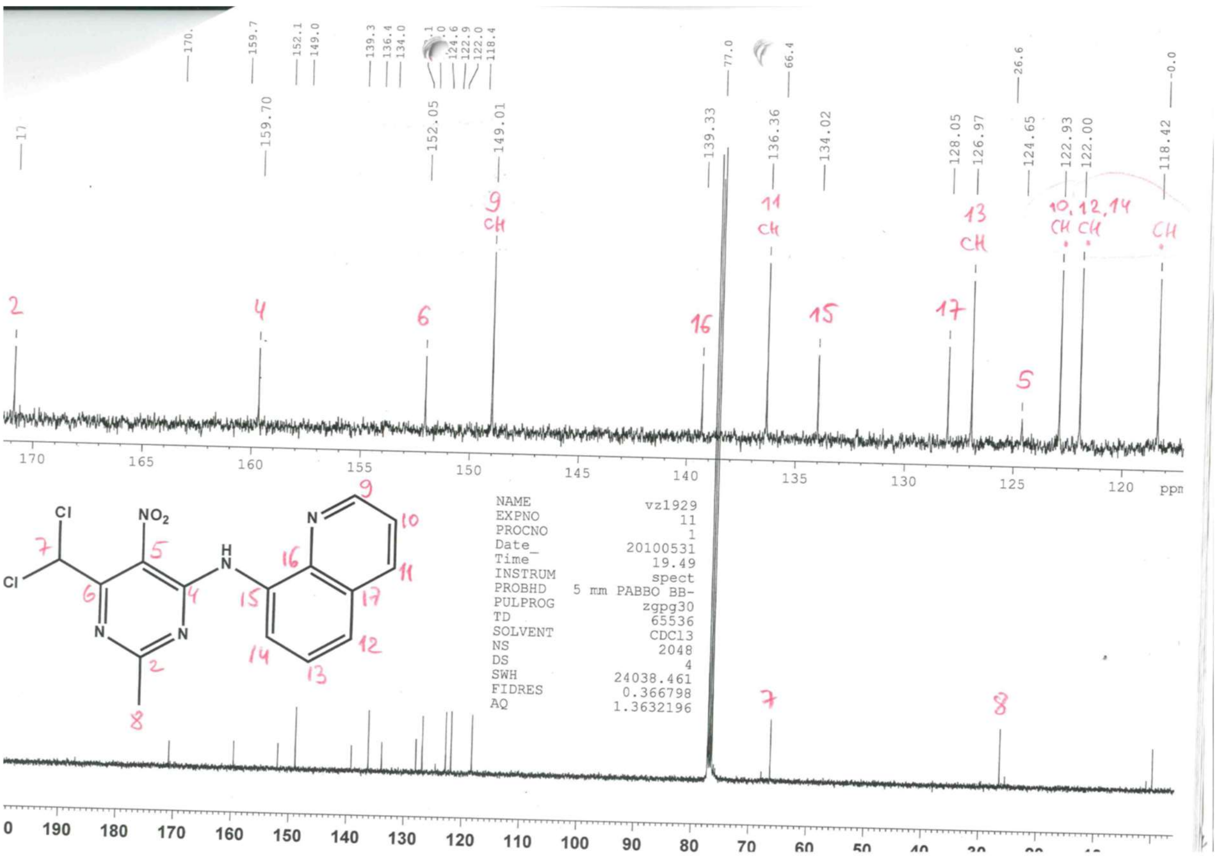

Figure S100. HR-MS spectrum for *N*-[6-(Dichloromethyl)-2-methyl-5-nitropyrimidin-4-yl]quinolin-8-amine (**27e**).

Elemental Composition Report

Page 1

Single Mass Analysis (displaying only valid results)

Tolerance = 10.0 PPM / DBE: min = -1.5, max = 50.0
Selected filters: None

Monoisotopic Mass, Even Electron Ions
1973 formula(e) evaluated with 19 results within limits (up to 80 closest results for each mass)
Elements Used:
C: 0-50 H: 0-100 N: 0-6 O: 0-10 Na: 0-1 Cl: 0-3

Zapolski LCT Premier KD070
VZ 1929, pos 23 (0.510) AM (Cen,4, 90.00, Ar,11000.0,556.28,0.70,LS 5) 1: TOF MS ES+ 518

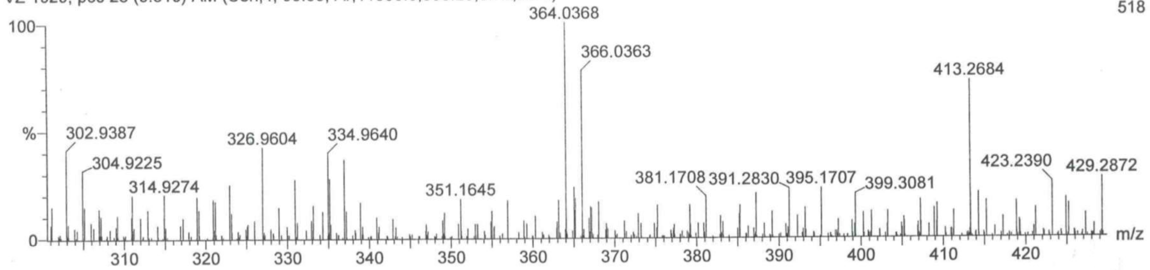

Mass	Calc. Mass	mDa	PPM	DBE	i-FIT	Formula
Minimum:				-1.5		
Maximum:		5.0	10.0	50.0		
364.0368	364.0368	0.0	0.0	11.5	4.1	C15 H12 N5 O2 Cl2
	364.0366	0.2	0.5	17.5	54.6	C19 H8 N5 Na Cl
	364.0374	-0.6	-1.6	21.5	182.7	C24 H7 N O2 Na
	364.0362	0.6	1.6	3.5	17.3	C12 H18 N3 O2 Na Cl3
	364.0377	-0.9	-2.5	15.5	52.3	C20 H11 N O4 Cl
	364.0358	1.0	2.7	20.5	182.2	C21 H6 N3 O4
	364.0355	1.3	3.6	6.5	6.5	C14 H16 N O6 Cl2
	364.0353	1.5	4.1	12.5	52.8	C18 H12 N O4 Na Cl
	364.0384	-1.6	-4.4	12.5	2.9	C18 H13 N3 Na Cl2
	364.0386	-1.8	-4.9	6.5	13.5	C14 H17 N3 O2 Cl3
	364.0390	-2.2	-6.0	20.5	55.3	C21 H7 N5 Cl
	364.0346	2.2	6.0	2.5	23.2	C9 H17 N5 O4 Cl3
	364.0344	2.4	6.6	8.5	6.8	C13 H13 N5 O2 Na Cl2
	364.0393	-2.5	-6.9	8.5	193.2	C12 H11 N3 O9 Na
	364.0399	-3.1	-8.5	24.5	182.4	C26 H6 N O2
	364.0336	3.2	8.8	11.5	53.7	C15 H11 N3 O6 Cl
	364.0334	3.4	9.3	17.5	184.4	C19 H7 N3 O4 Na
	364.0403	-3.5	-9.6	-0.5	23.8	C6 H17 N5 O7 Na Cl2
	364.0403	-3.5	-9.6	7.5	10.2	C17 H18 N Na Cl3

Figure S101. 400 MHz ^{1}H-NMR spectrum in $CDCl_3$ for **27f.**

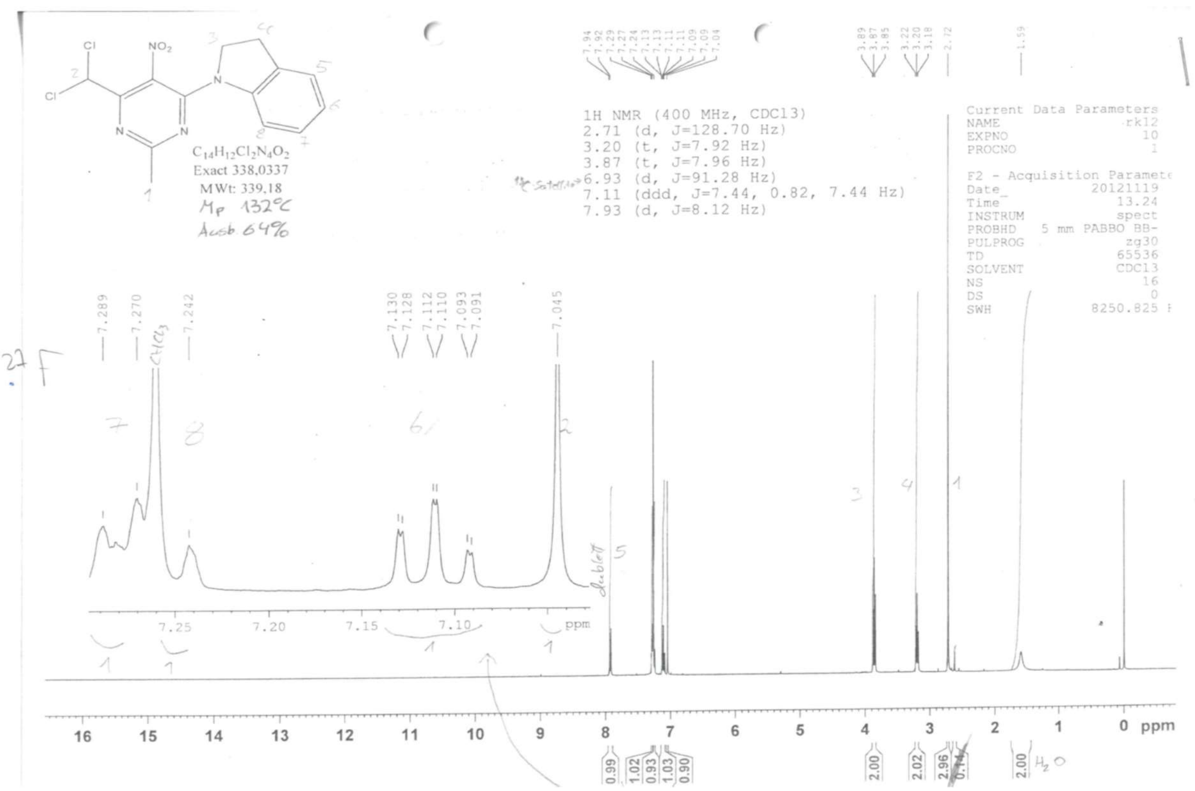

Figure S102. 100 MHz ^{13}C-NMR spectrum in $CDCl_3$ for **27f.**

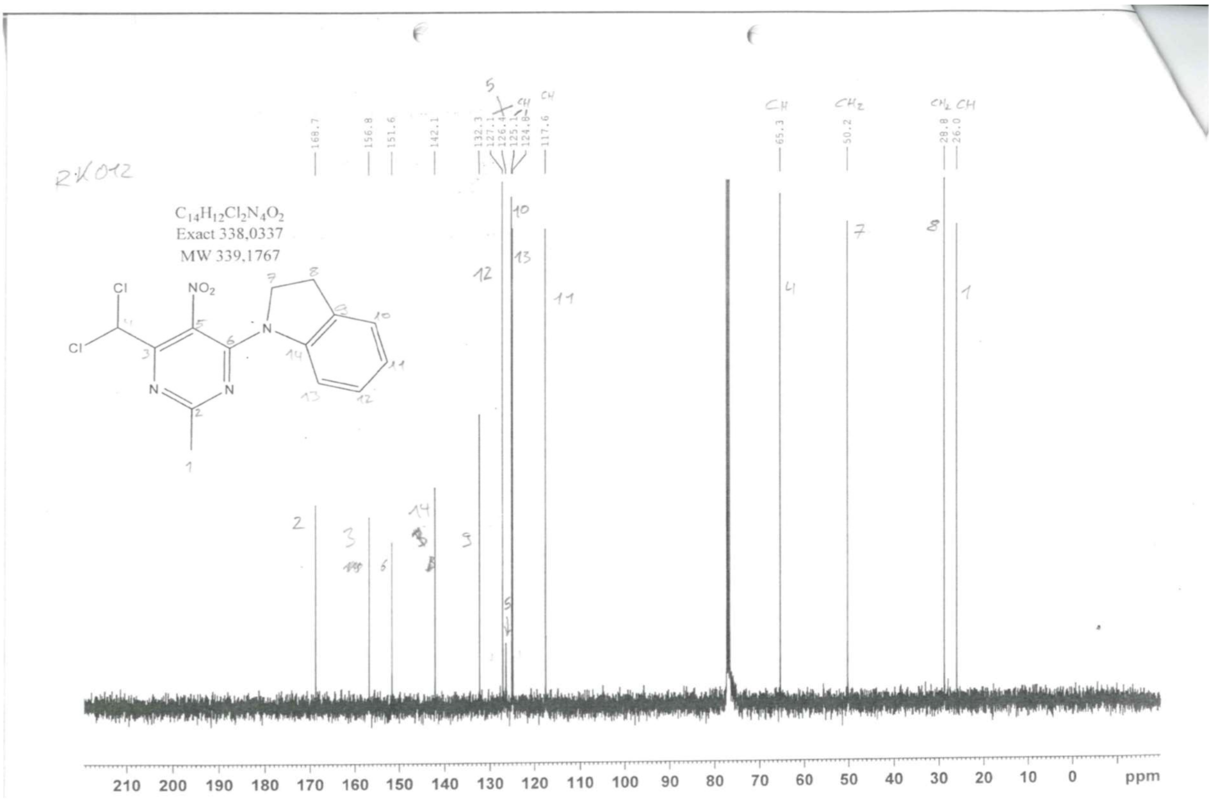

Figure S103. HR-MS spectrum for 1-[6-(dichloromethyl)-2-methyl-5-nitropyrimidin-4-yl]-2,3-dihydro-1H-indole (**27f**).

.lemental Composition Report

Page 1

Single Mass Analysis

Tolerance = 5.0 PPM / DBE: min = -1.5, max = 50.0
Element prediction: Off
Number of isotope peaks used for i-FIT = 3

Monoisotopic Mass, Odd and Even Electron Ions
418 formula(e) evaluated with 5 results within limits (up to 50 closest results for each mass)
Elements Used:
C: 0-100 H: 0-100 N: 0-5 O: 0-5 Cl: 0-2

Kupiec LCT 30-Apr-2013
SK RK-012 182 (2.053) AM (Cen,5, 32.00, Ar,0.0,196.96,1.00,LS 5) 1: TOF MS ES+
7.75e+002

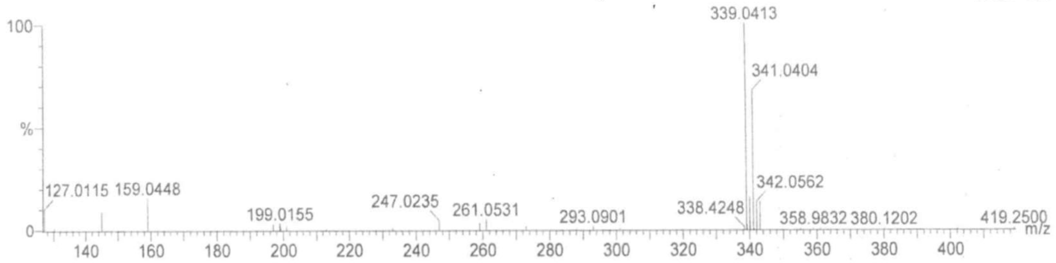

Mass	Calc. Mass	mDa	PPM	DBE	i-FIT	Formula
Minimum:				-1.5		
Maximum:		6.0	5.0	50.0		
339.0413	339.0411	0.2	0.6	14.0	63.7	C17 H10 N3 O3 Cl
	√ 339.0416	-0.3	-0.9	9.5	0.7	C14 H13 N4 O2 Cl2
	339.0406	0.7	2.1	18.5	248.0	C20 H7 N2 O4
	339.0424	-1.1	-3.2	13.5	66.1	C19 H12 O4 Cl
	339.0429	-1.6	-4.7	9.0	1.8	C16 H15 N O3 Cl2

Figure S104. 600 MHz ^{1}H-NMR spectrum in $CDCl_3$ for **28.**

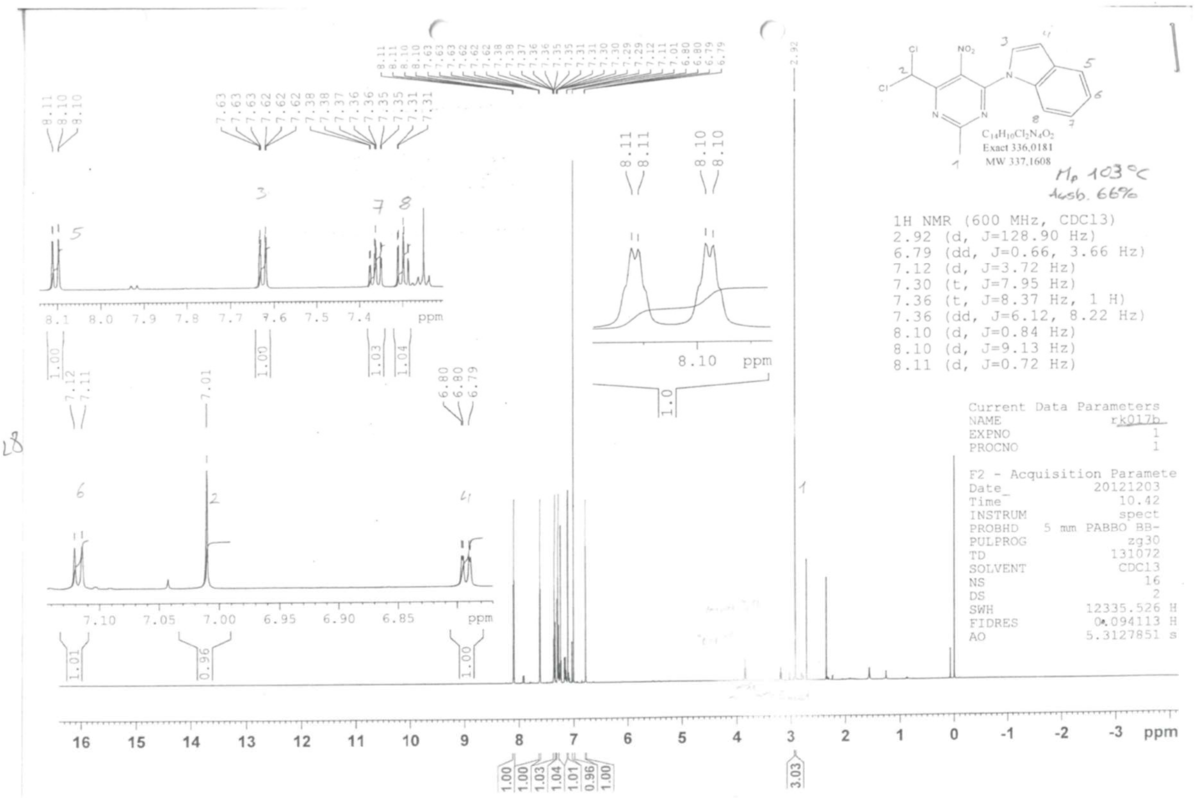

Figure S105. 150 MHz ^{13}C-NMR spectrum in $CDCl_3$ for **28.**

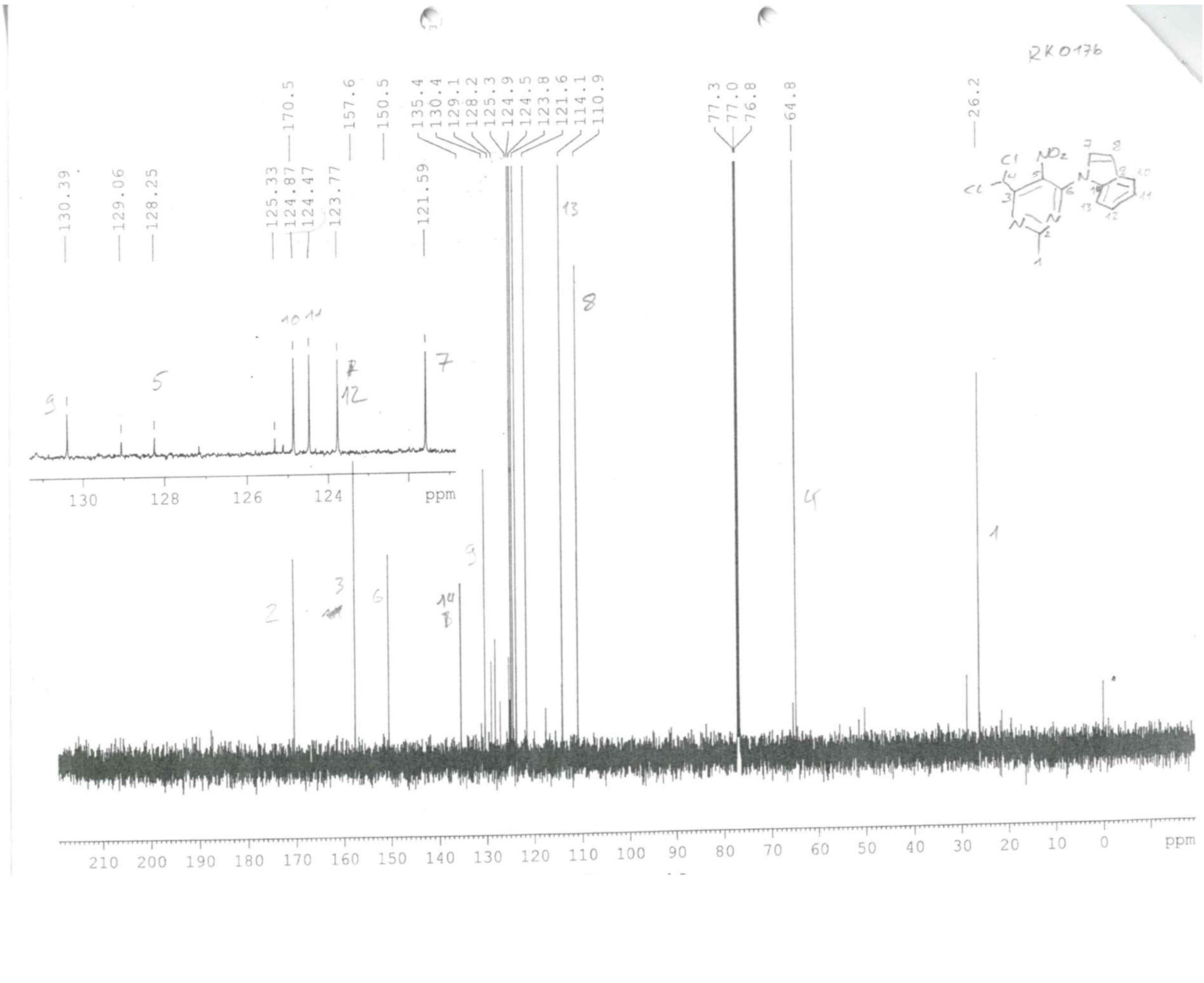

Figure S106. 400 MHz ^{1}H-NMR spectrum in $CDCl_3$ for **25g.**

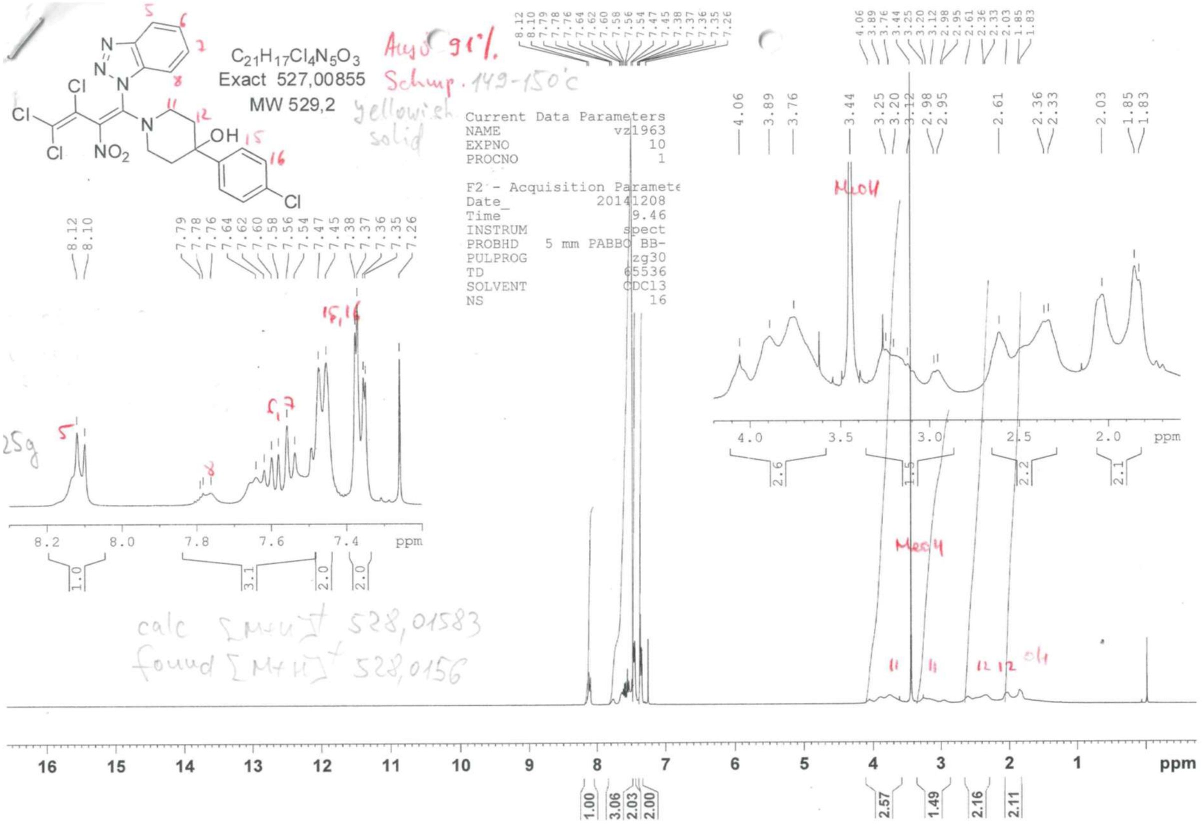

Figure S107. 100 MHz ^{13}C-NMR spectrum in $CDCl_3$ for **25g.**

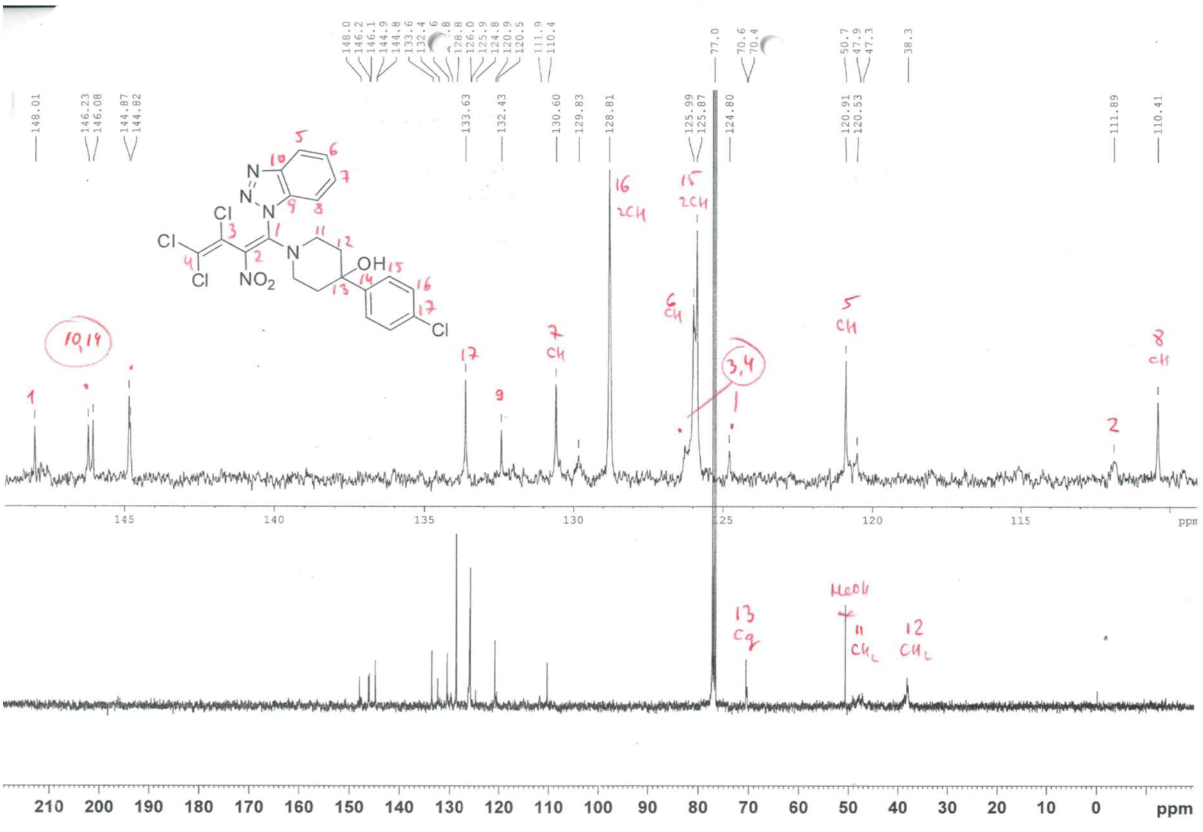

Figure S108. 200 MHz ^{1}H-NMR spectrum in $CDCl_3$ for **25h.**

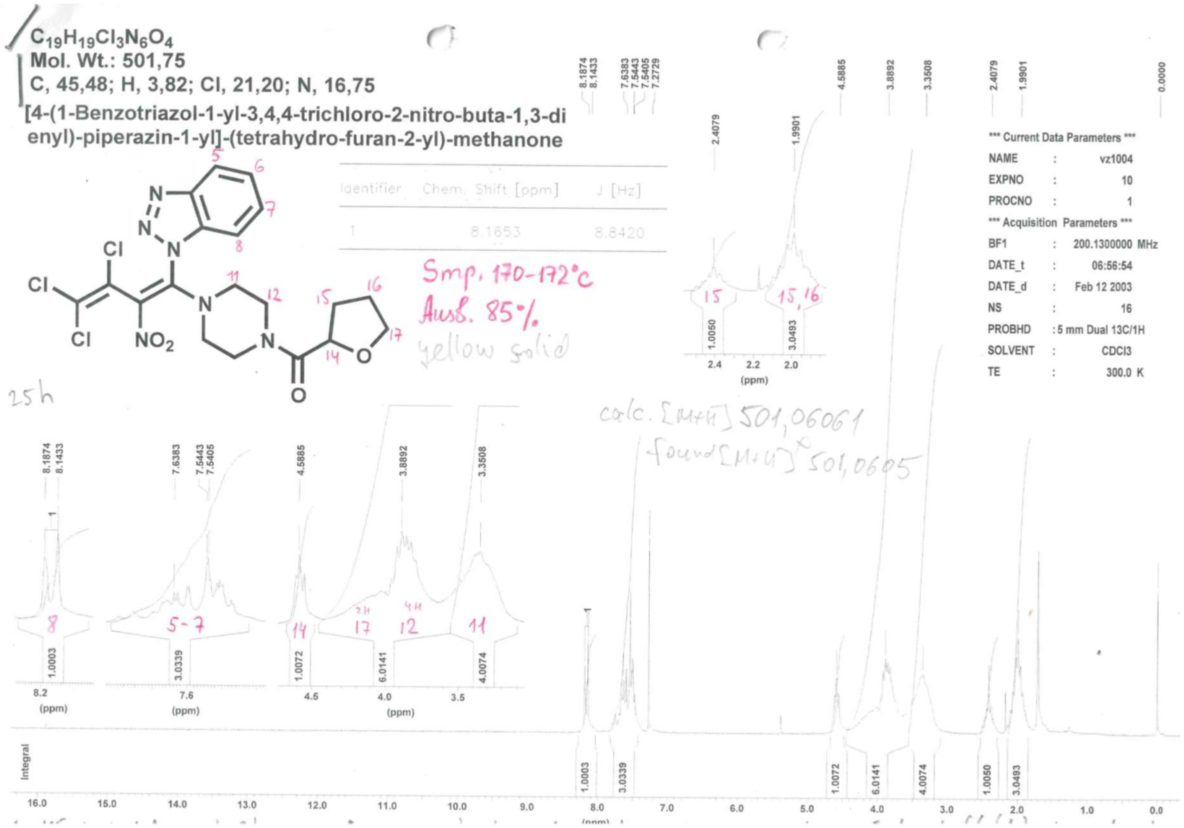

Figure S109. 50 MHz ^{13}C-NMR spectrum in $CDCl_3$ for **25h.**

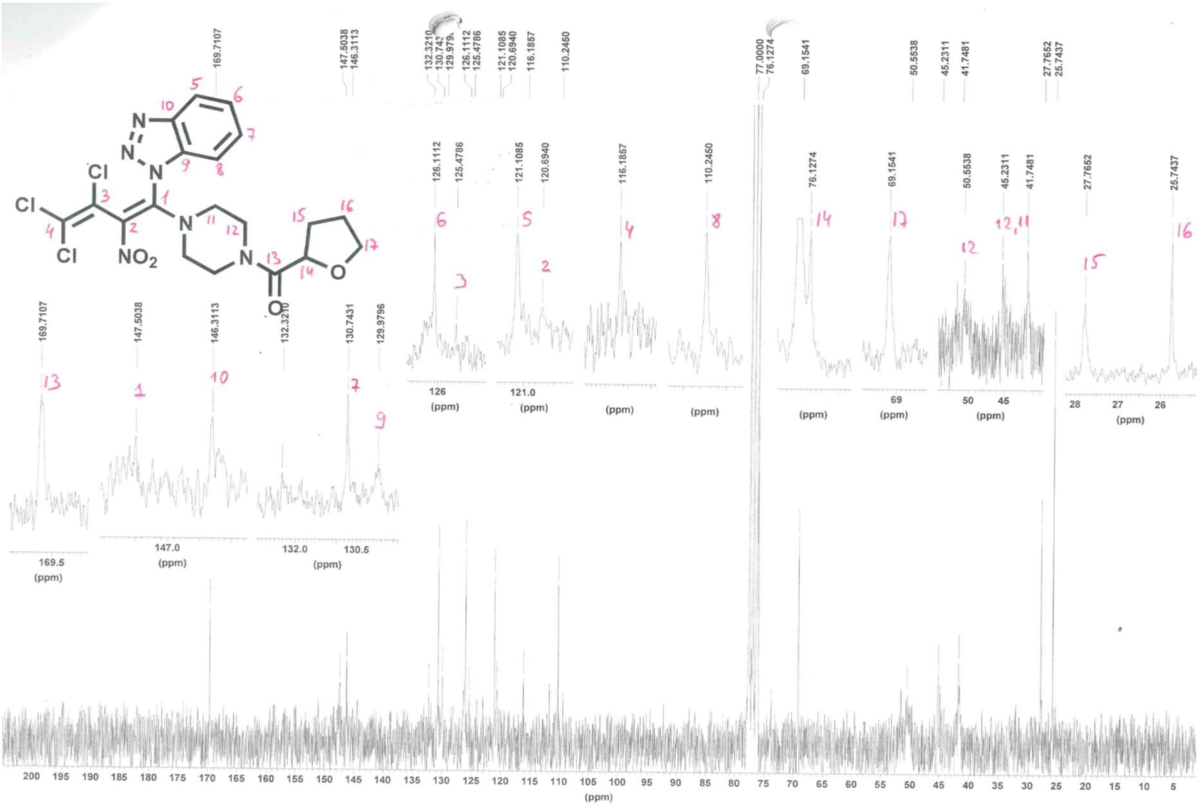

Figure S110. 200 MHz ^{1}H-NMR spectrum in $CDCl_3$ for **25i.**

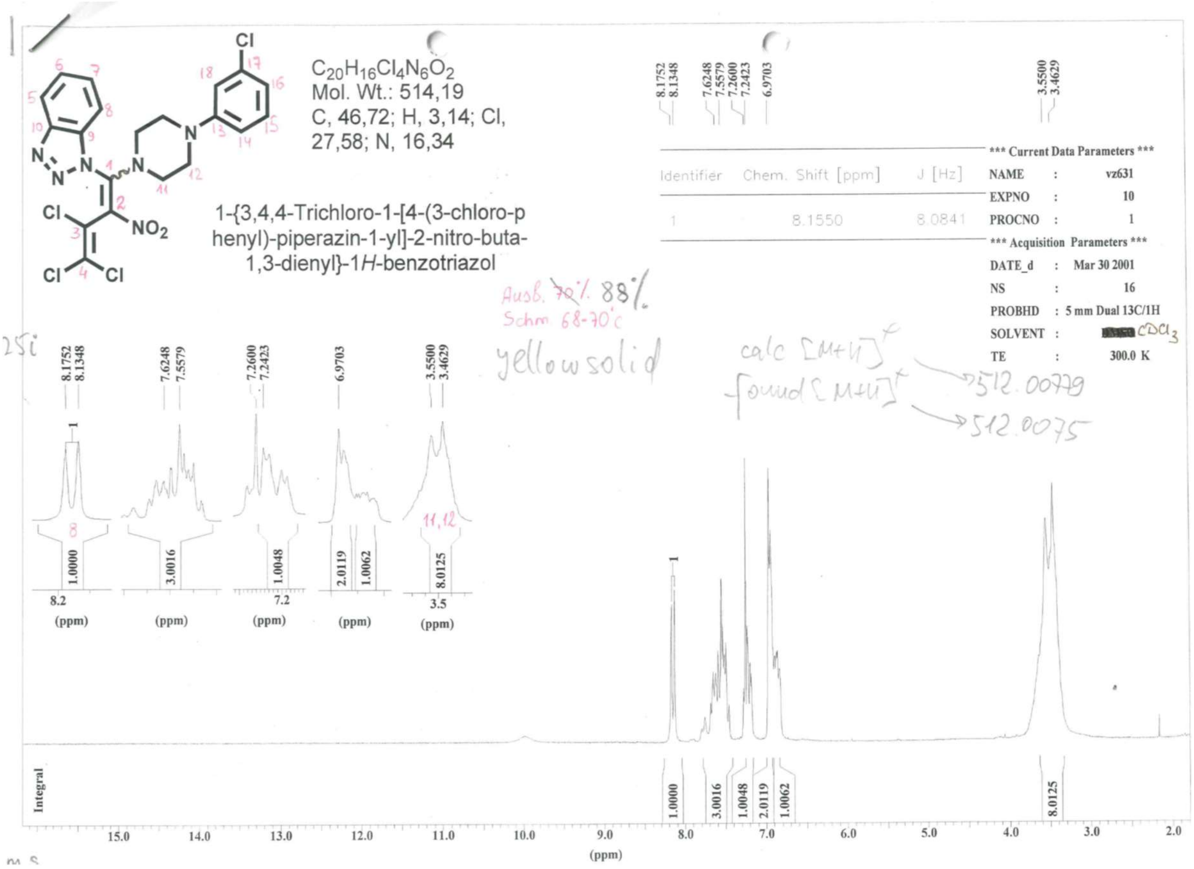

Figure S111. 50 MHz ^{13}C-NMR spectrum in $CDCl_3$ for **25i.**

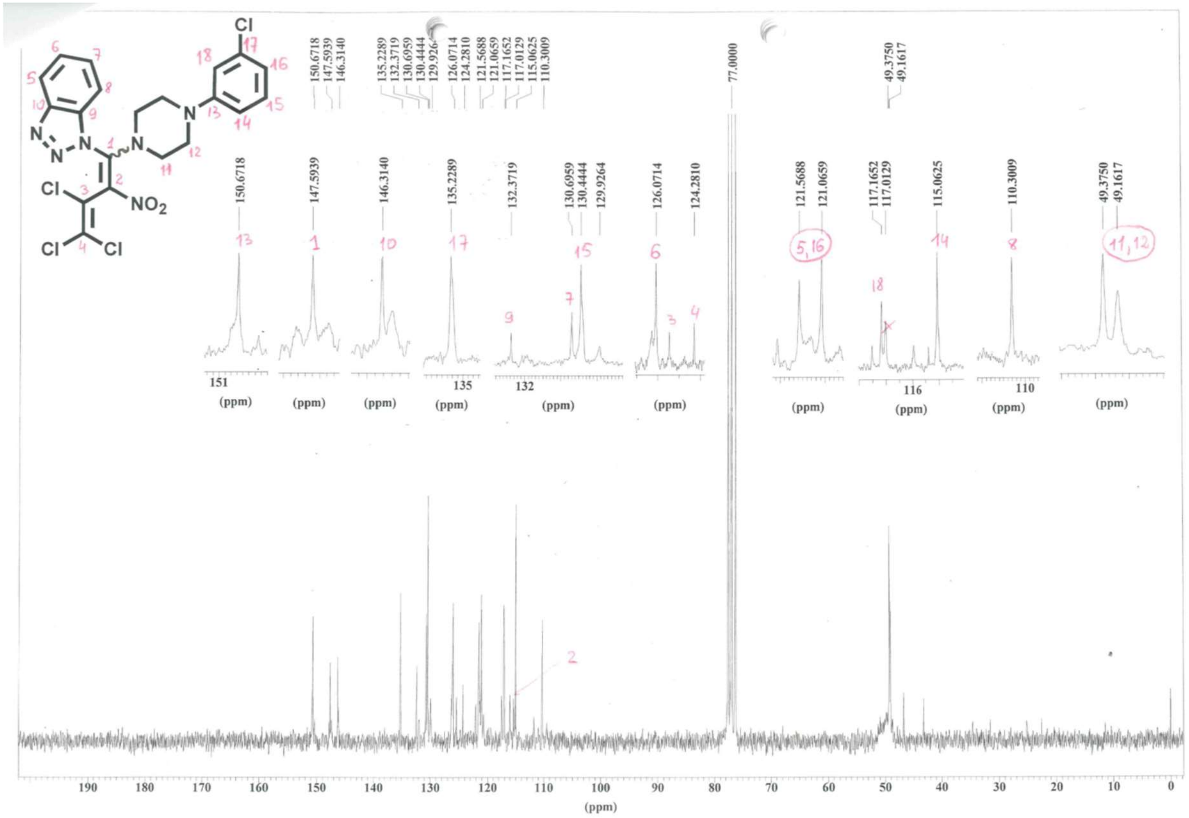

Figure S112. 400 MHz ^{1}H-NMR spectrum in $CDCl_3$ for **29a.**

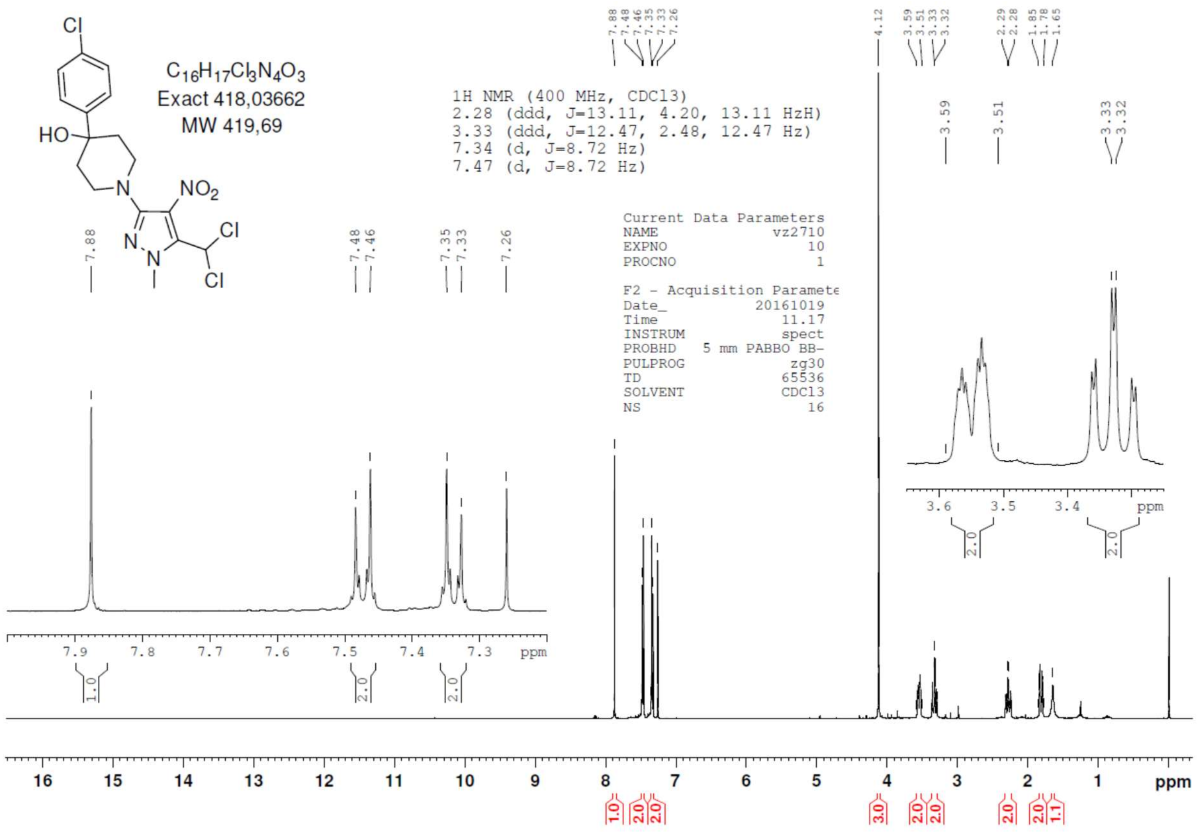

Figure S113. 100 MHz ^{13}C-NMR spectrum in $CDCl_3$ for **29a.**

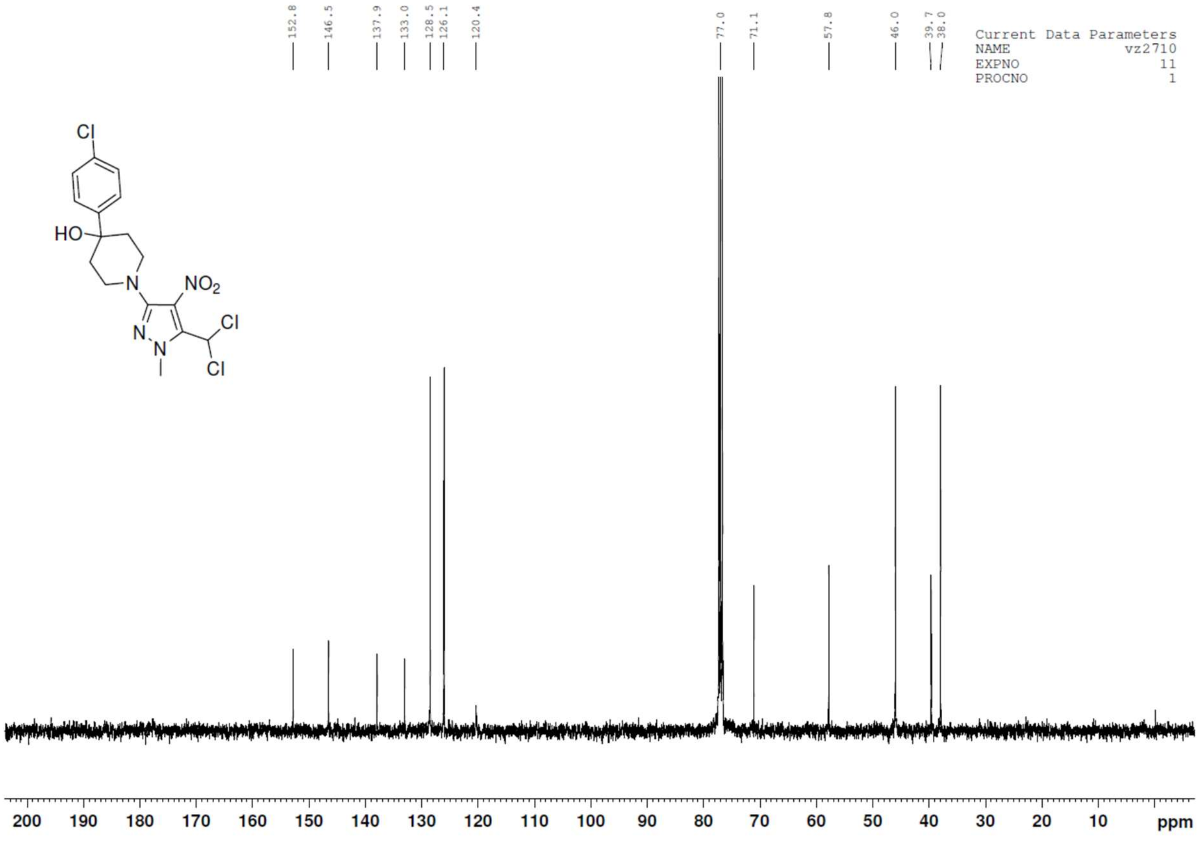

Figure S114. 400 MHz ^{1}H-NMR spectrum in $CDCl_3$ for **29b.**

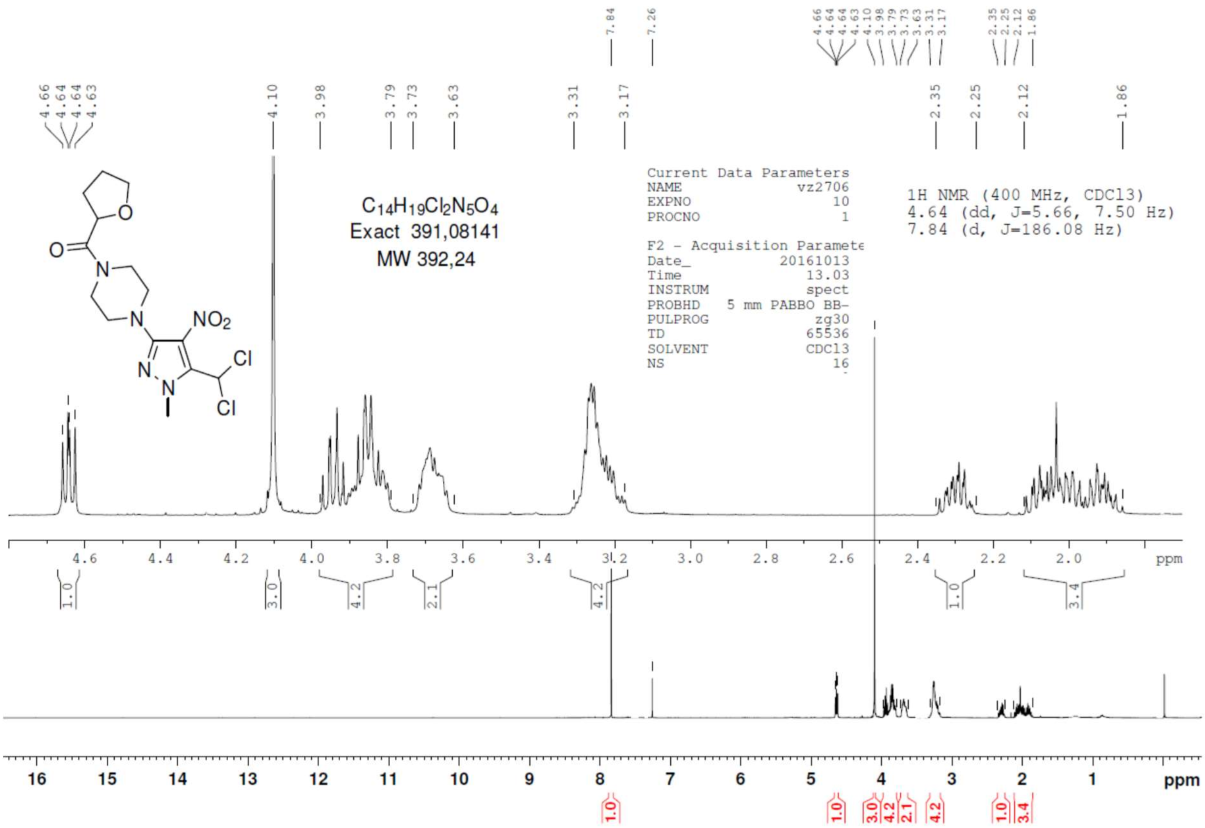

Figure S115. 100 MHz ^{13}C-NMR spectrum in $CDCl_3$ for **29b.**

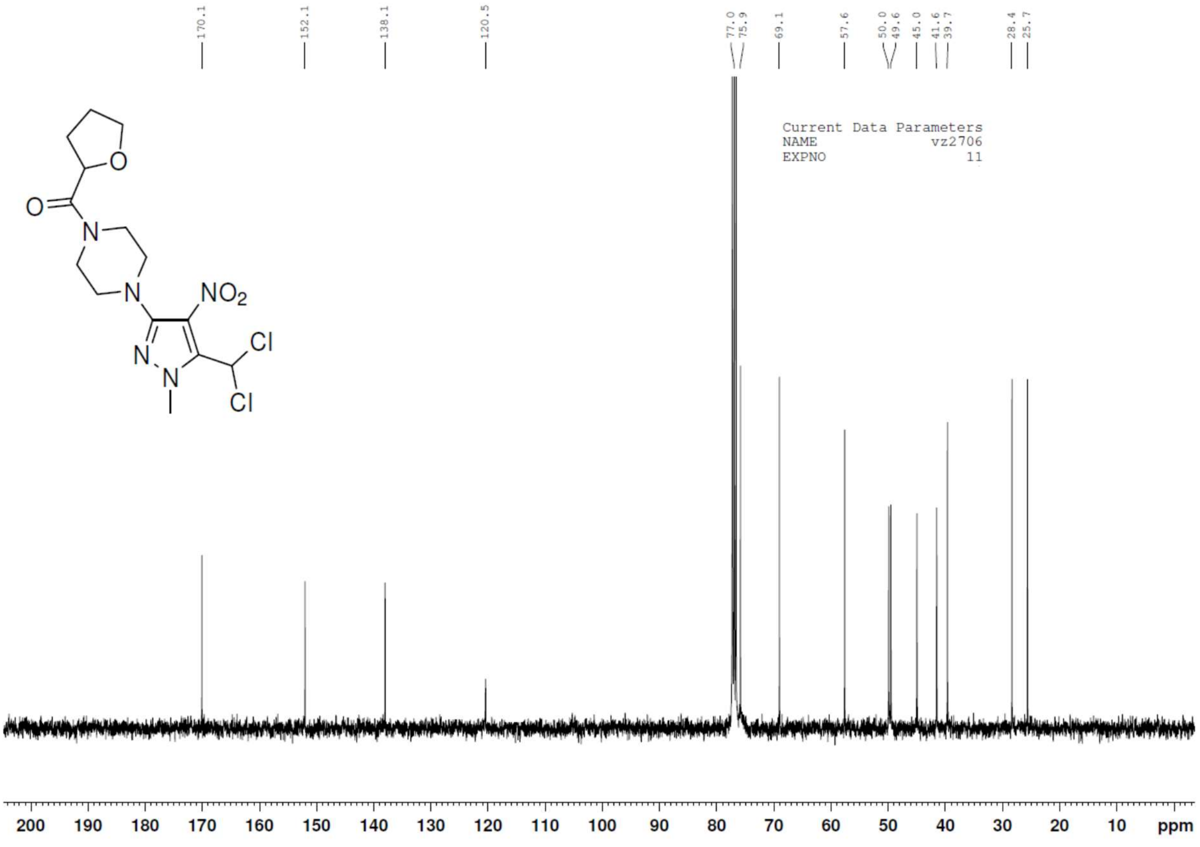

Figure S116. 400 MHz ^{1}H-NMR spectrum in $CDCl_3$ for **29c.**

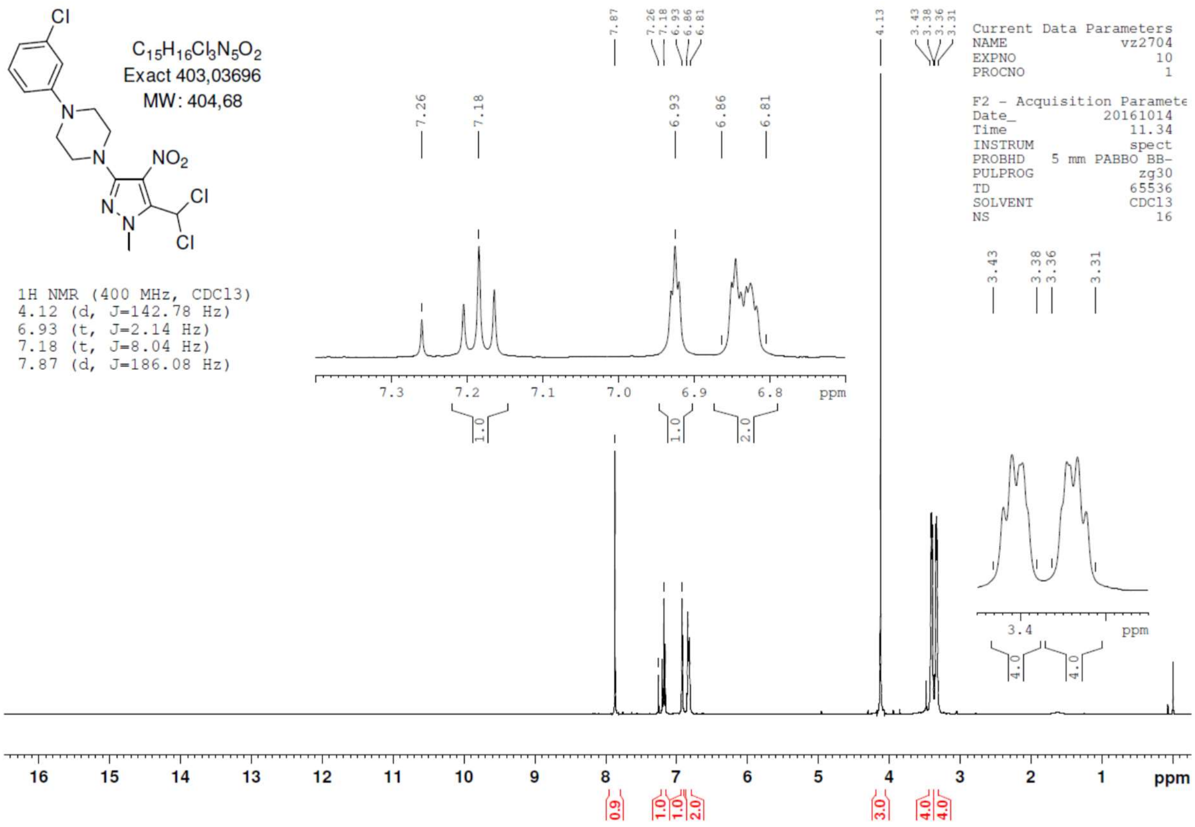

Figure S117. 100 MHz ^{13}C-NMR spectrum in $CDCl_3$ for **29c.**

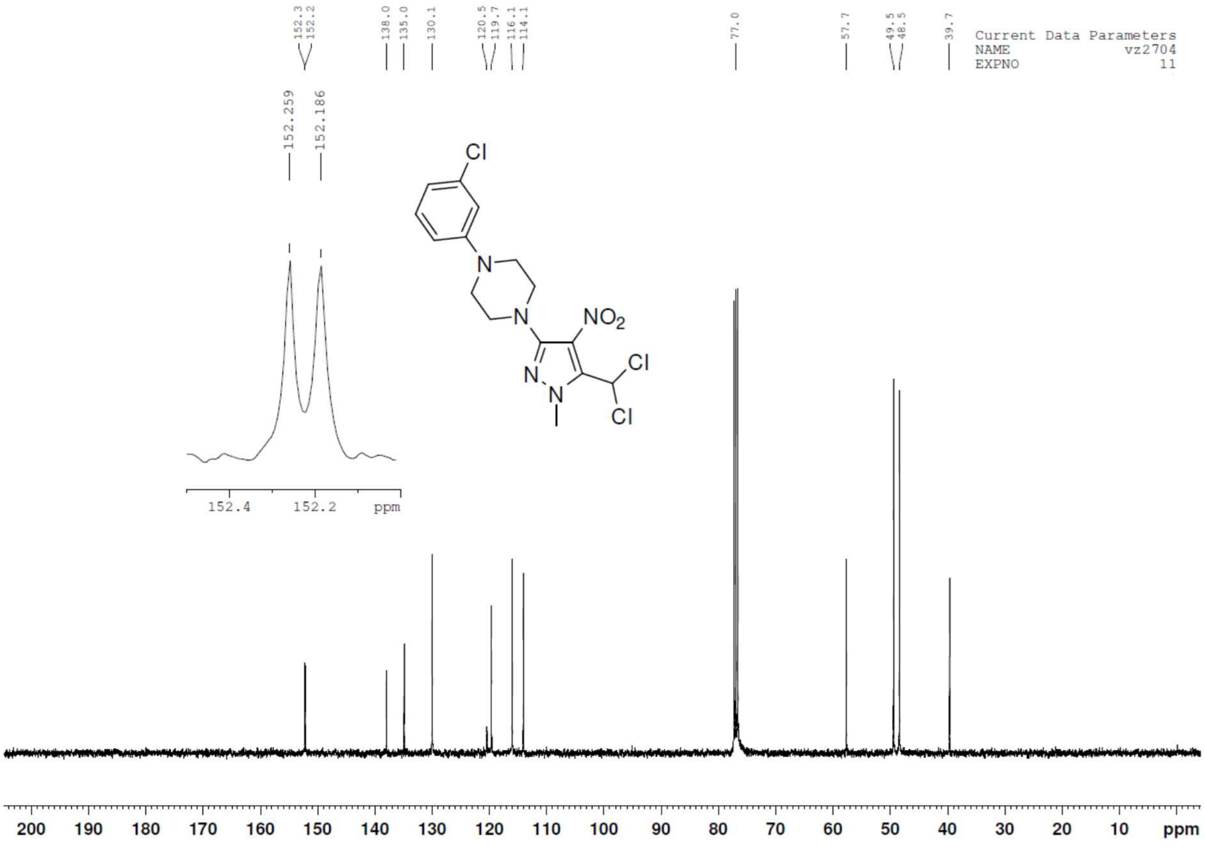

Figure S118. 400 MHz ^{1}H-NMR spectrum in $CDCl_3$ for **30.**

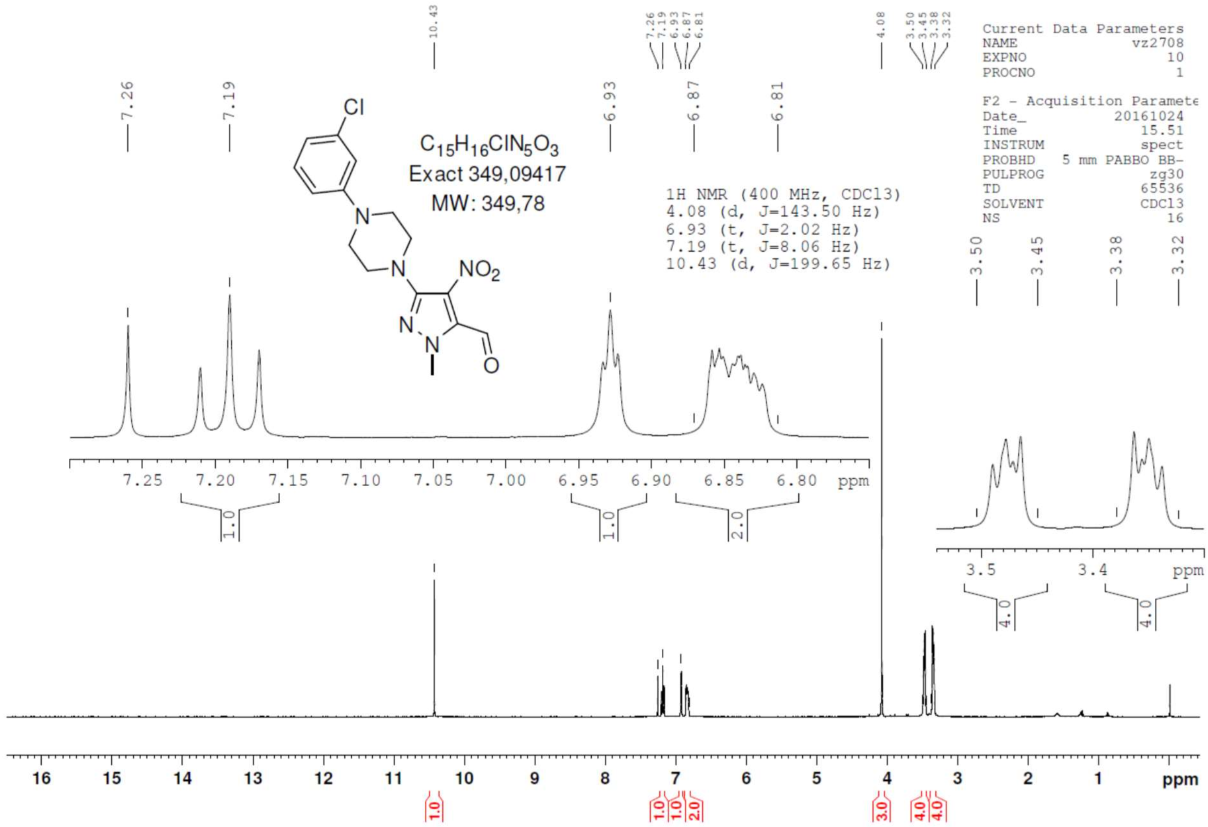

Figure S119. 100 MHz ^{13}C-NMR spectrum in $CDCl_3$ for **30.**

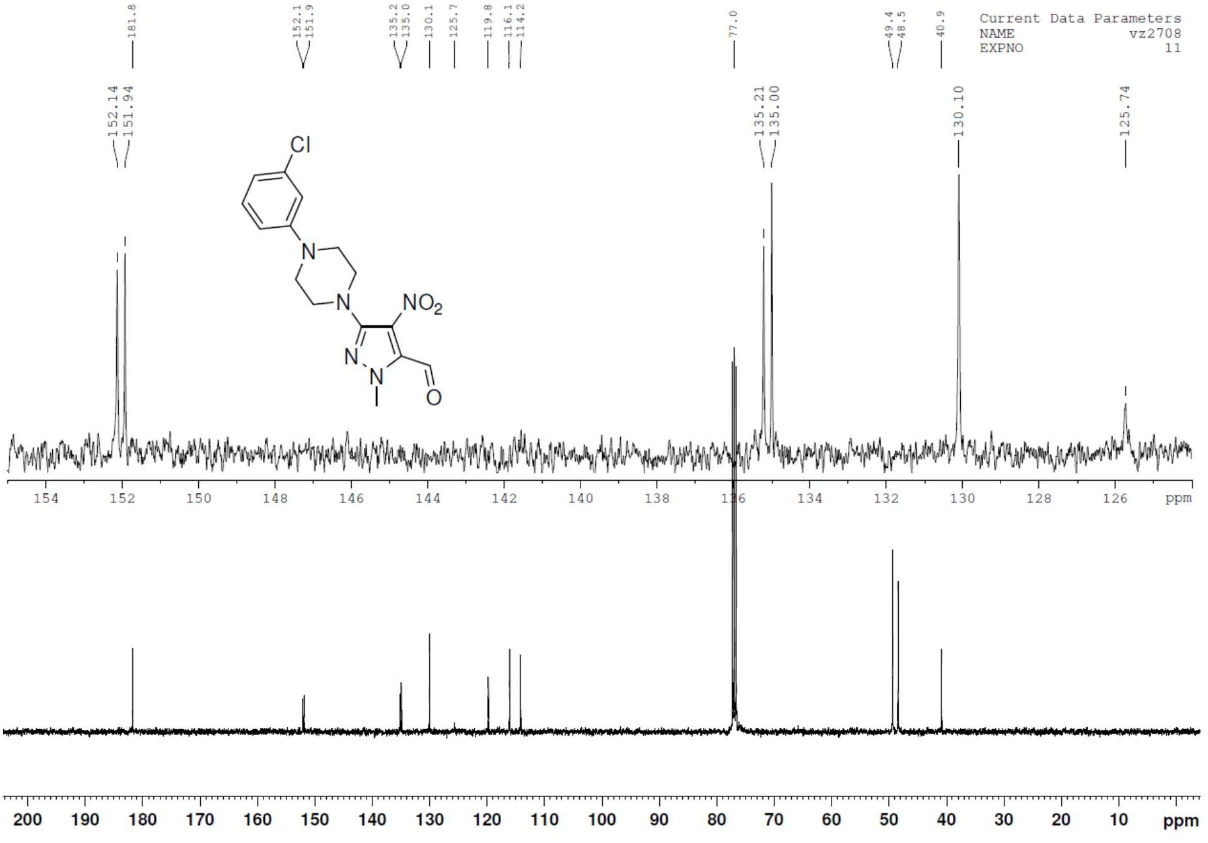

Figure S120. 200 MHz ^{1}H-NMR spectrum in DMSO-*d6* for **31a.**

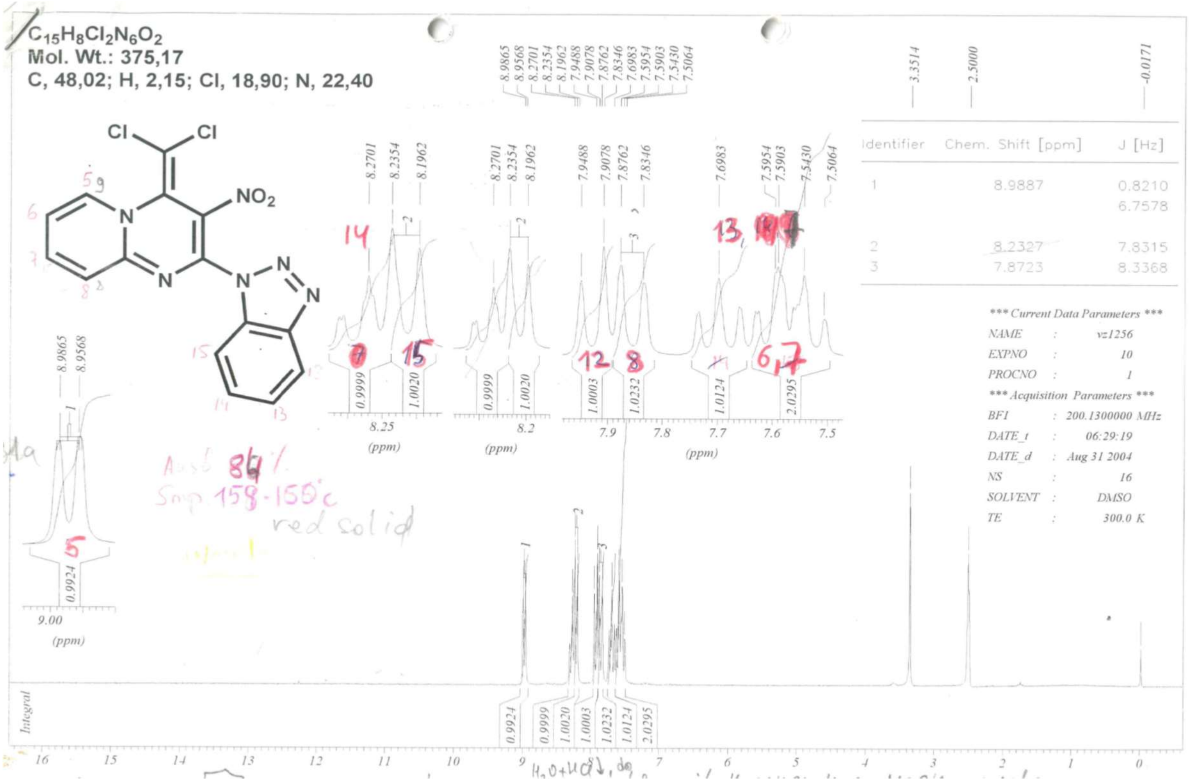

Figure S121. 50 MHz ^{13}C-NMR spectrum in DMSO-*d6* for **31a.**

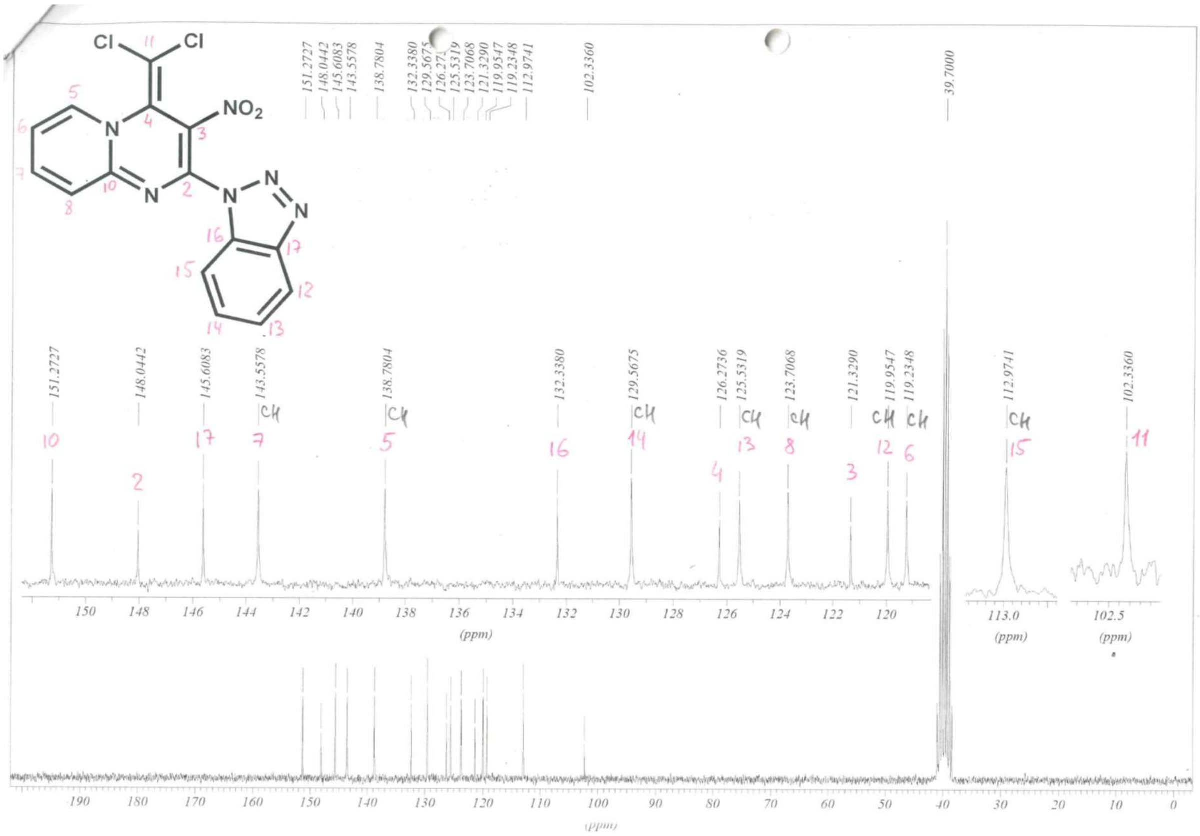

Figure S122. HR-MS spectrum for 2-(1*H*-benzotriazol-1-yl)-4-(dichloromethylidene)-3-nitro-4*H*-pyrido[1,2-*a*]pyrimidine (**31a**).

Elemental Composition Report

Single Mass Analysis
Tolerance = 6.0 mDa / DBE: min = -1.5, max = 50.0
Element prediction: Off
Number of isotope peaks used for i-FIT = 3

Monoisotopic Mass, Odd and Even Electron Ions
548 formula(e) evaluated with 18 results within limits (up to 30 closest results for each mass)
Elements Used:
C: 0-60 H: 0-90 N: 0-6 O: 0-2 Na: 0-1 Cl: 0-2
Zapolski,TUC Q-Tof Premier UPLC-MS 05-May-2010 13:10:47

VZ 1256 217 (2.229) AM (Cen,5, 85.00, Ar,10000.0,556.28,0.70,LS 10) 1: TOF MS ES+ 1.20e+003

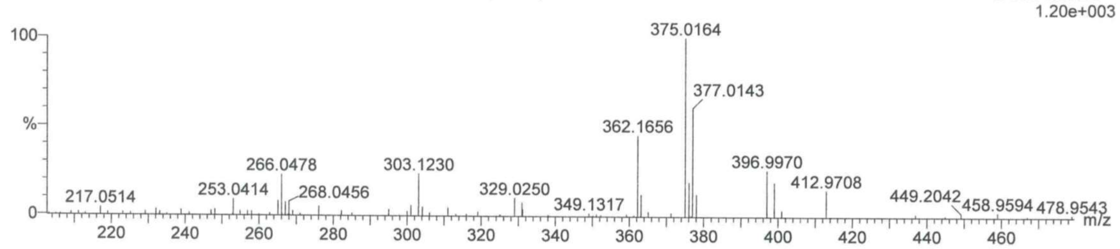

Mass	Calc. Mass	mDa	PPM	DBE	i-FIT	Formula
Minimum:				-1.5		
Maximum:		6.0	40.0	50.0		
375.0164	375.0164	0.0	0.0	13.5	1.9	C15 H9 N6 O2 Cl2
	375.0162	0.2	0.5	19.5	69.6	C19 H5 N6 Na Cl
	375.0170	-0.6	-1.6	23.5	338.3	C24 H4 N2 O2 Na
	375.0157	0.7	1.9	24.0	338.7	C22 H2 N5 O Na
	375.0175	-1.1	-2.9	19.0	72.6	C21 H7 N3 O Na Cl
	375.0180	-1.6	-4.3	14.5	3.1	C18 H10 N4 Na Cl2
	375.0181	-1.7	-4.5	27.0	342.1	C24 H N5 O
	375.0186	-2.2	-5.9	22.5	77.3	C21 H4 N6 Cl
	375.0140	2.4	6.4	10.5	3.8	C13 H10 N6 O2 Na Cl2
	375.0189	-2.5	-6.7	18.5	76.1	C23 H9 O2 Na Cl
	375.0194	-3.0	-8.0	14.0	5.1	C20 H12 N O Na Cl2
	375.0195	-3.1	-8.3	26.5	342.8	C26 H3 N2 O2
	375.0199	-3.5	-9.3	22.0	81.4	C23 H6 N3 O Cl
	375.0204	-4.0	-10.7	17.5	7.2	C20 H9 N4 Cl2
	375.0211	-4.7	-12.5	27.5	353.2	C29 H4 Na
	375.0213	-4.9	-13.1	21.5	86.3	C25 H8 O2 Cl
	375.0218	-5.4	-14.4	17.0	10.6	C22 H11 N O Cl2
	375.0109	5.5	14.7	31.0	354.4	C30 H N

Figure S123. 200 MHz ^{1}H-NMR spectrum in DMSO-*d6* for **31b.**

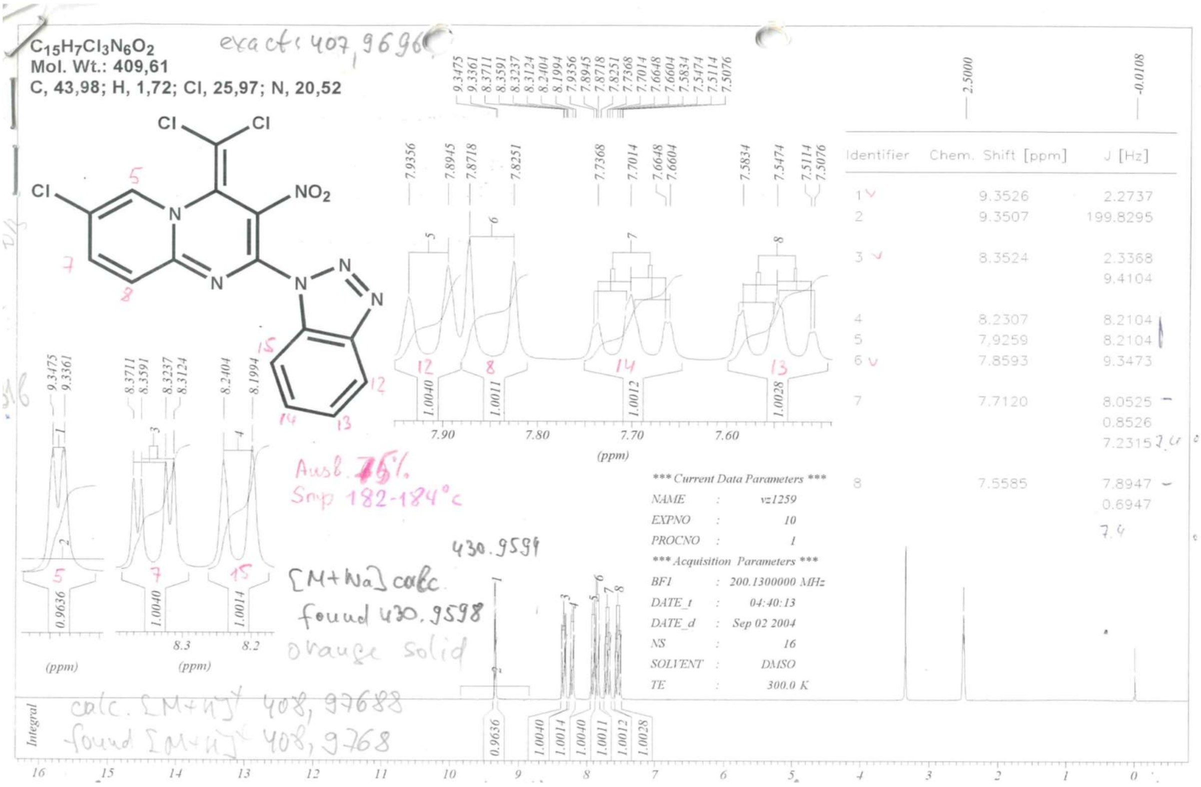

Figure S124. 50 MHz ^{13}C-NMR spectrum in DMSO-*d6* for **31b.**

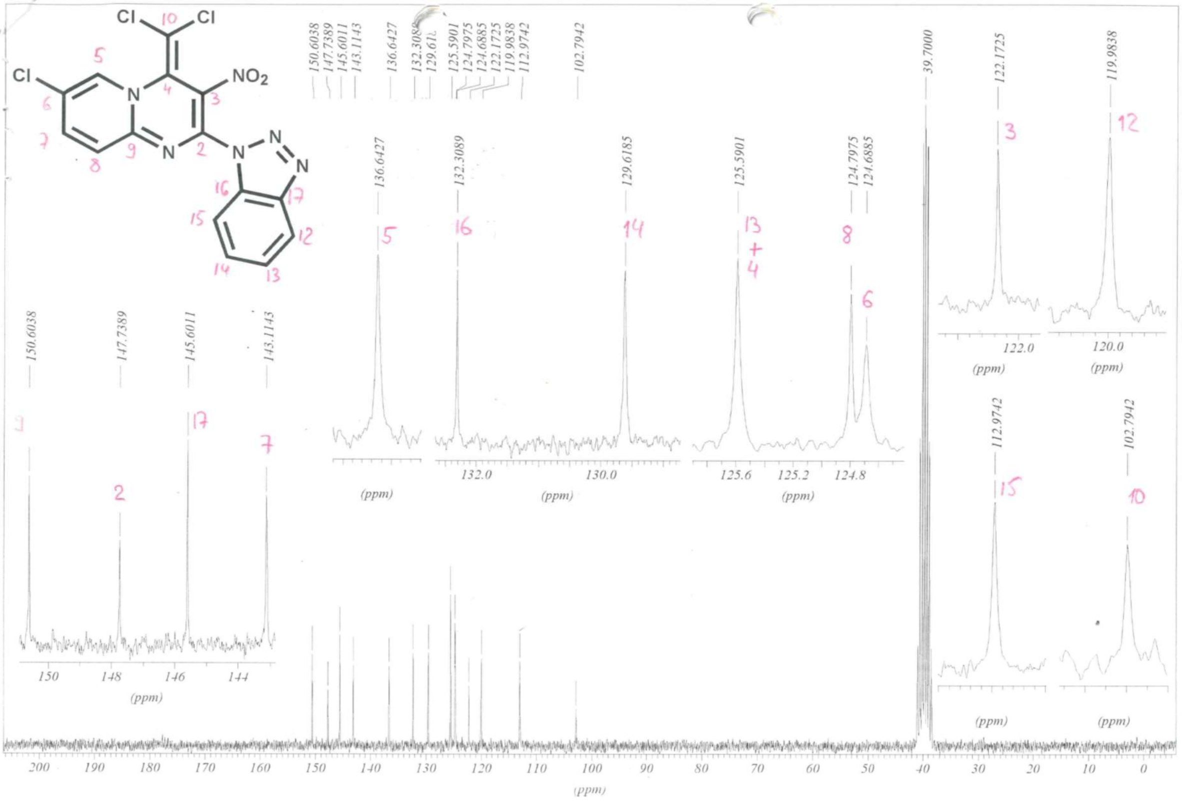

Figure S125. 200 MHz ¹H-NMR spectrum in DMSO-*d6* for **32a.**

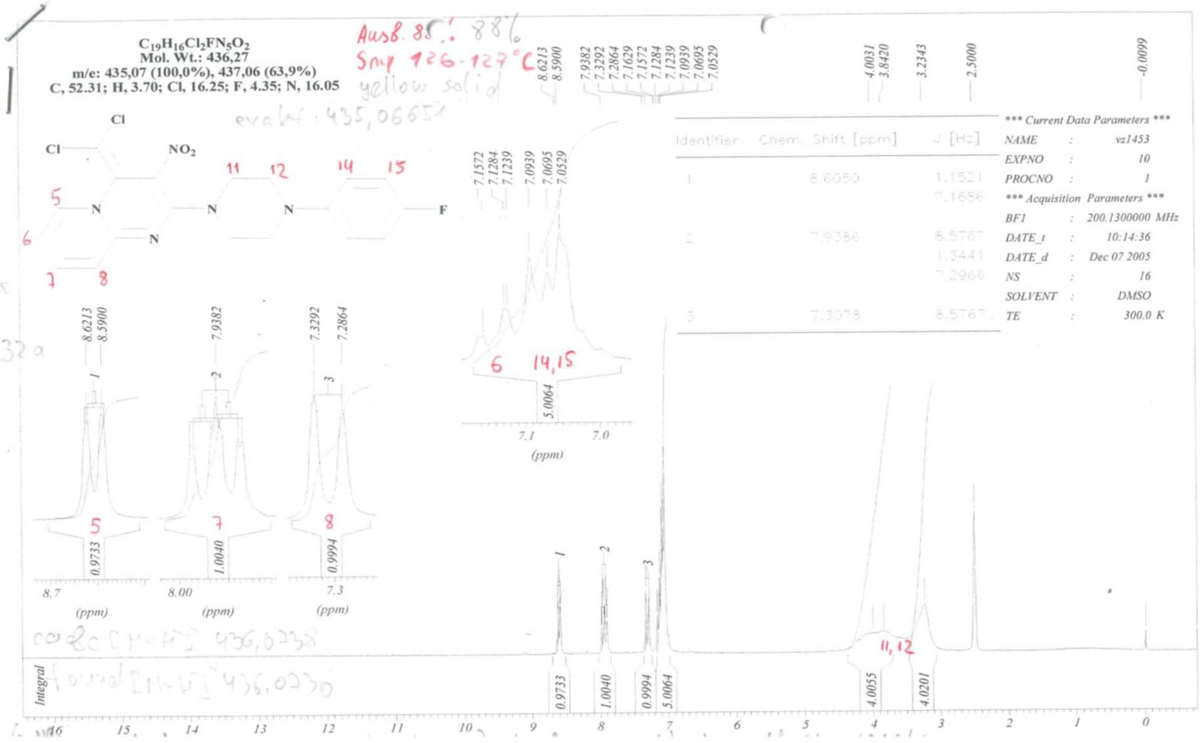

Figure S126. 50 MHz ¹³C-NMR spectrum in DMSO-*d6* for **32a.**

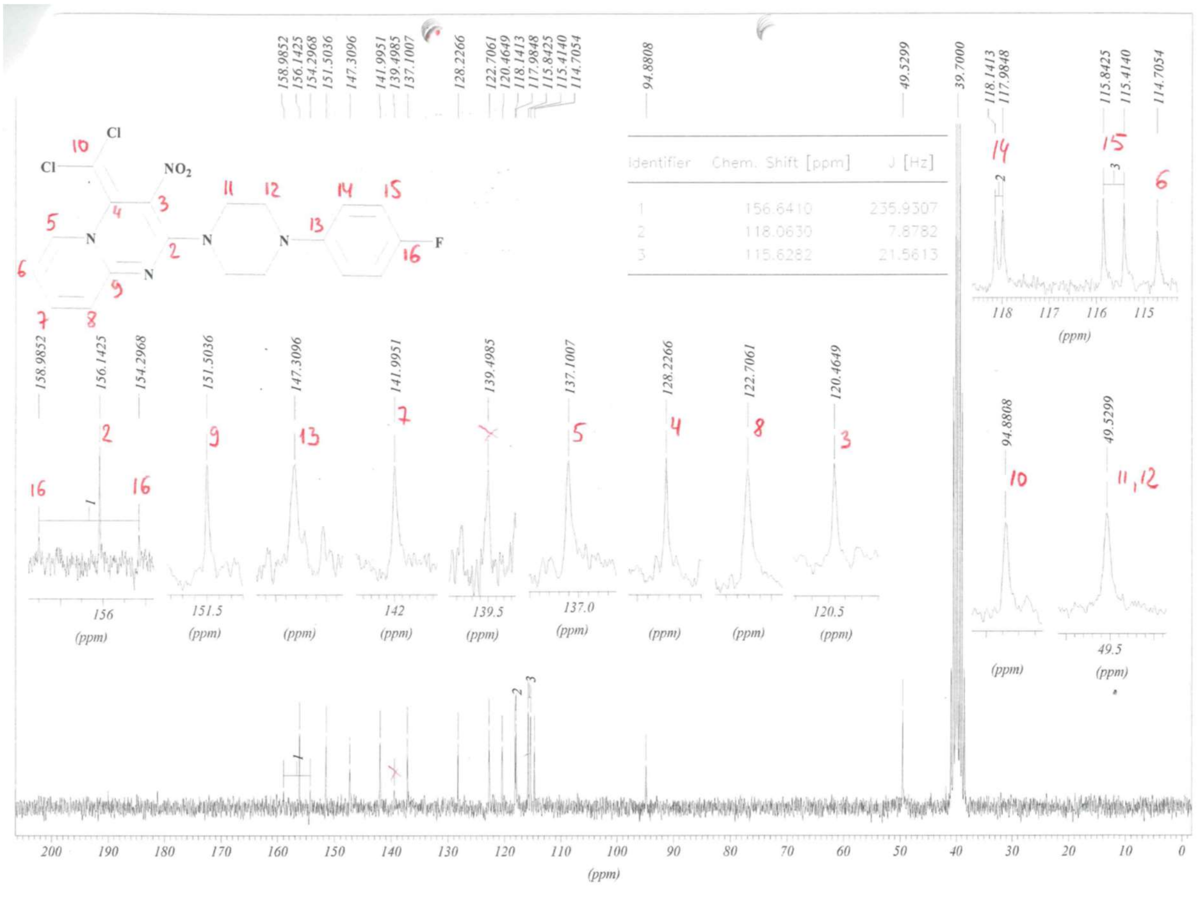

Figure S127. 200 MHz ^{1}H-NMR spectrum in DMSO-*d6* for **32b.**

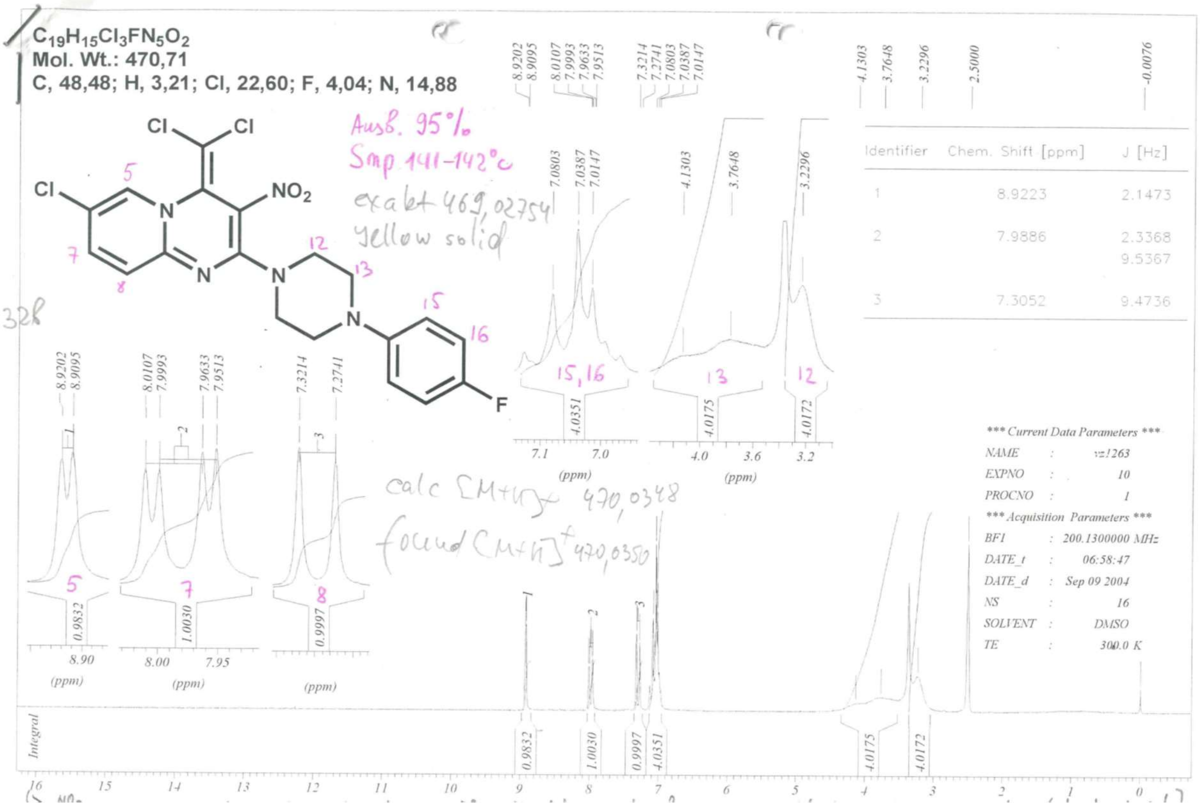

Figure S128. 50 MHz ^{13}C-NMR spectrum in DMSO-*d6* for **32b.**

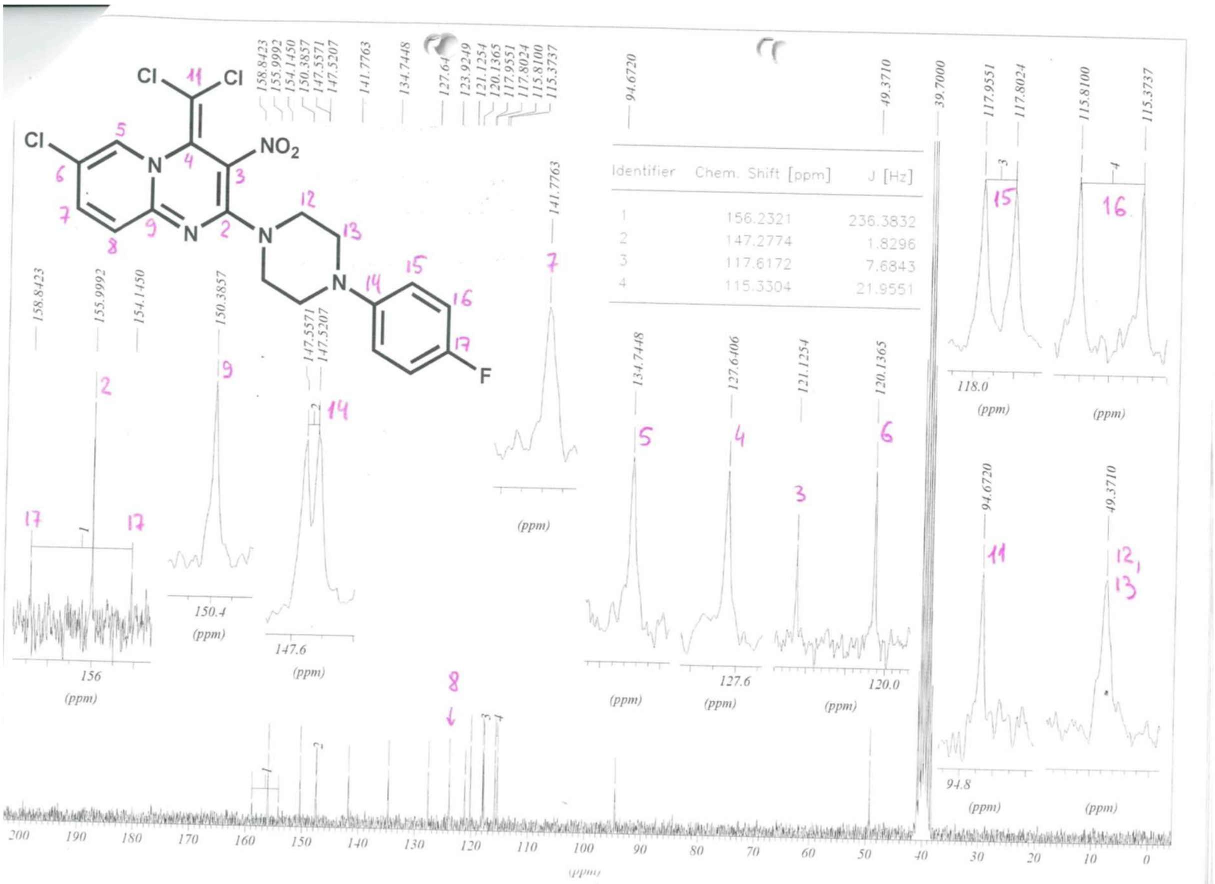

Figure S129. 400 MHz ^{1}H-NMR spectrum in $CDCl_3$ for **33a.**

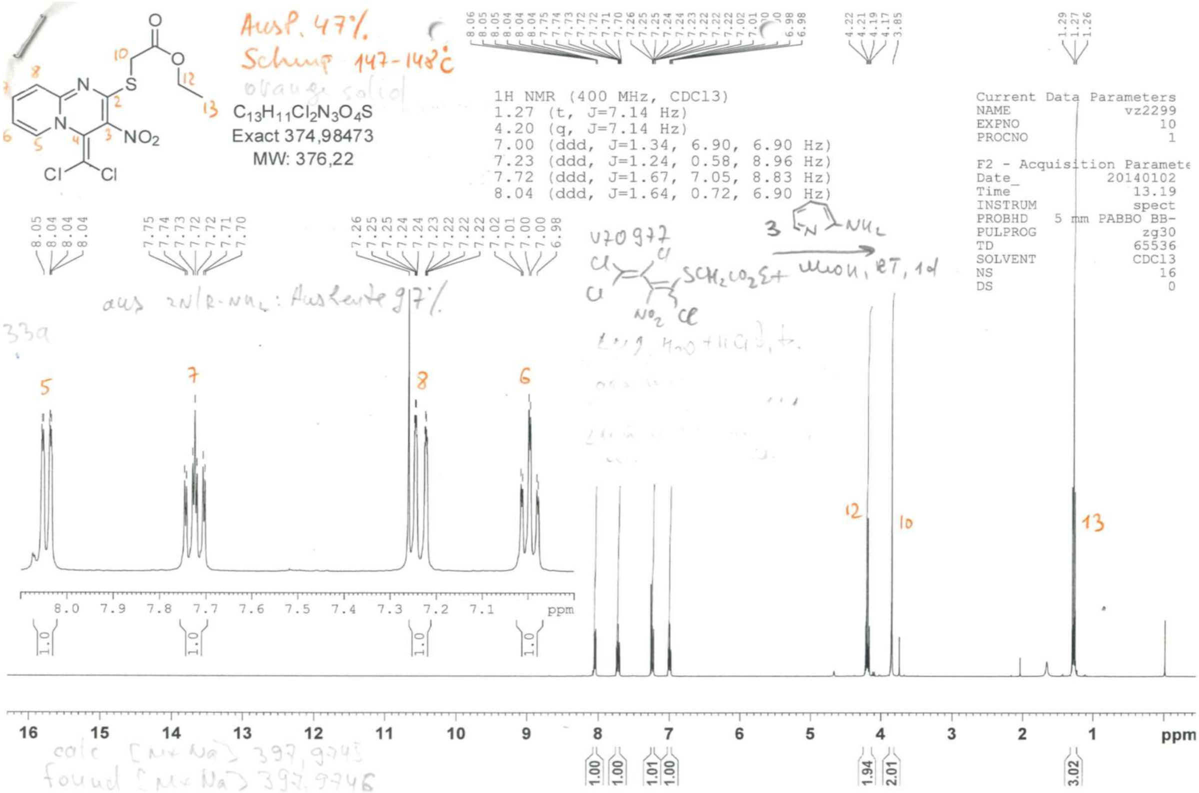

Figure S130. 100 MHz ^{13}C-NMR spectrum in $CDCl_3$ for **33a.**

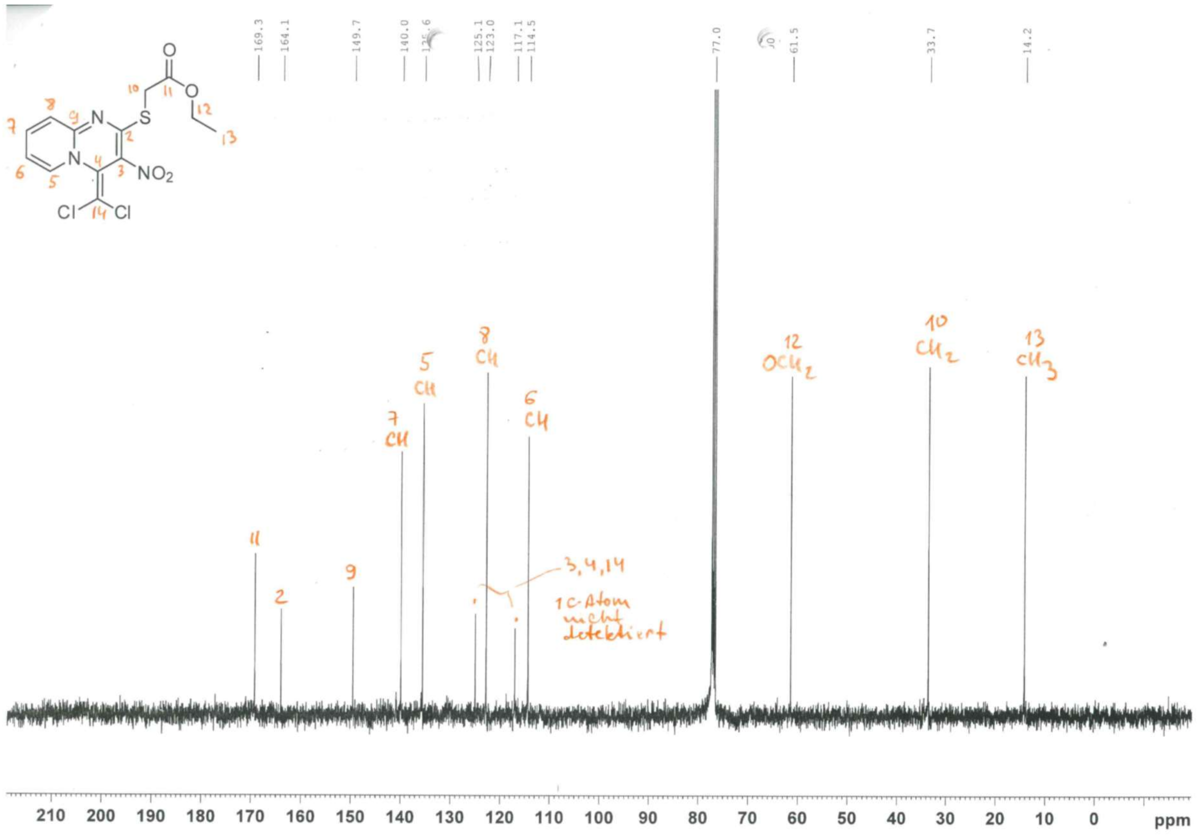

Figure S131. 200 MHz ^{1}H-NMR spectrum in $CDCl_3$ for **33b.**

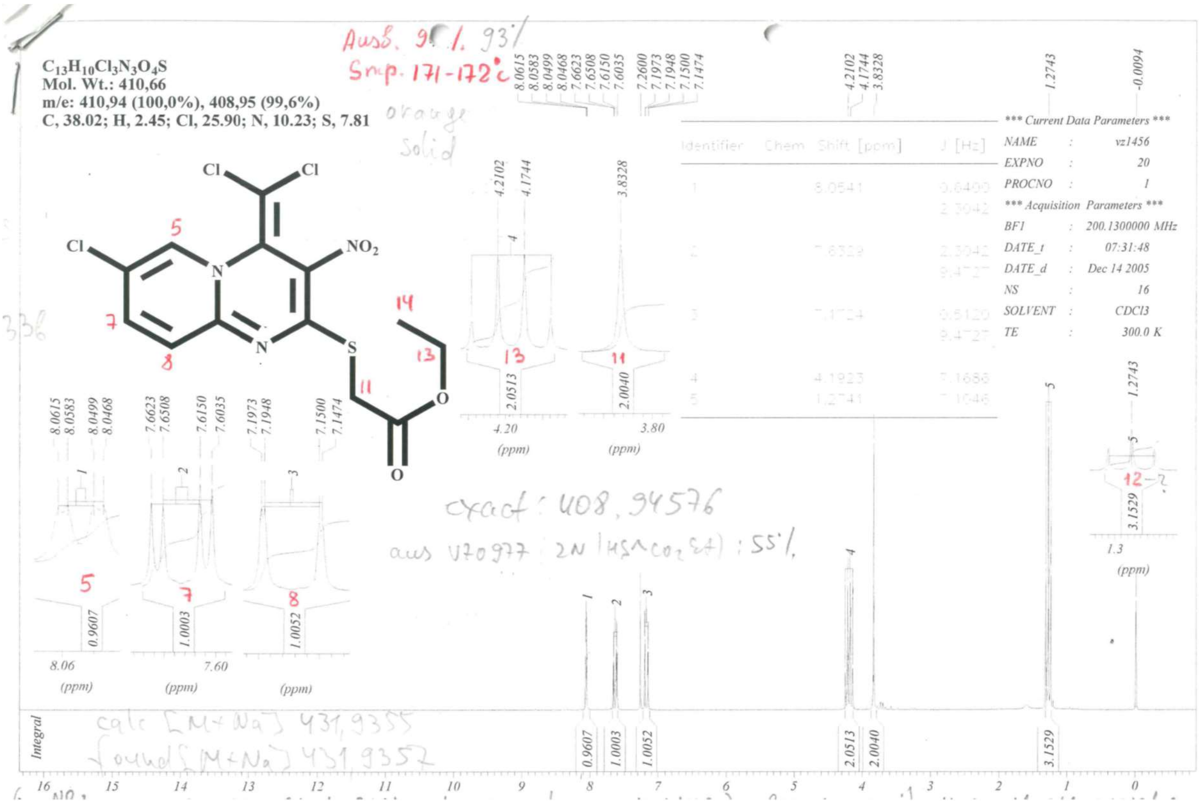

Figure S132. 50 MHz ^{13}C-NMR spectrum in $CDCl_3$ for **33b.**

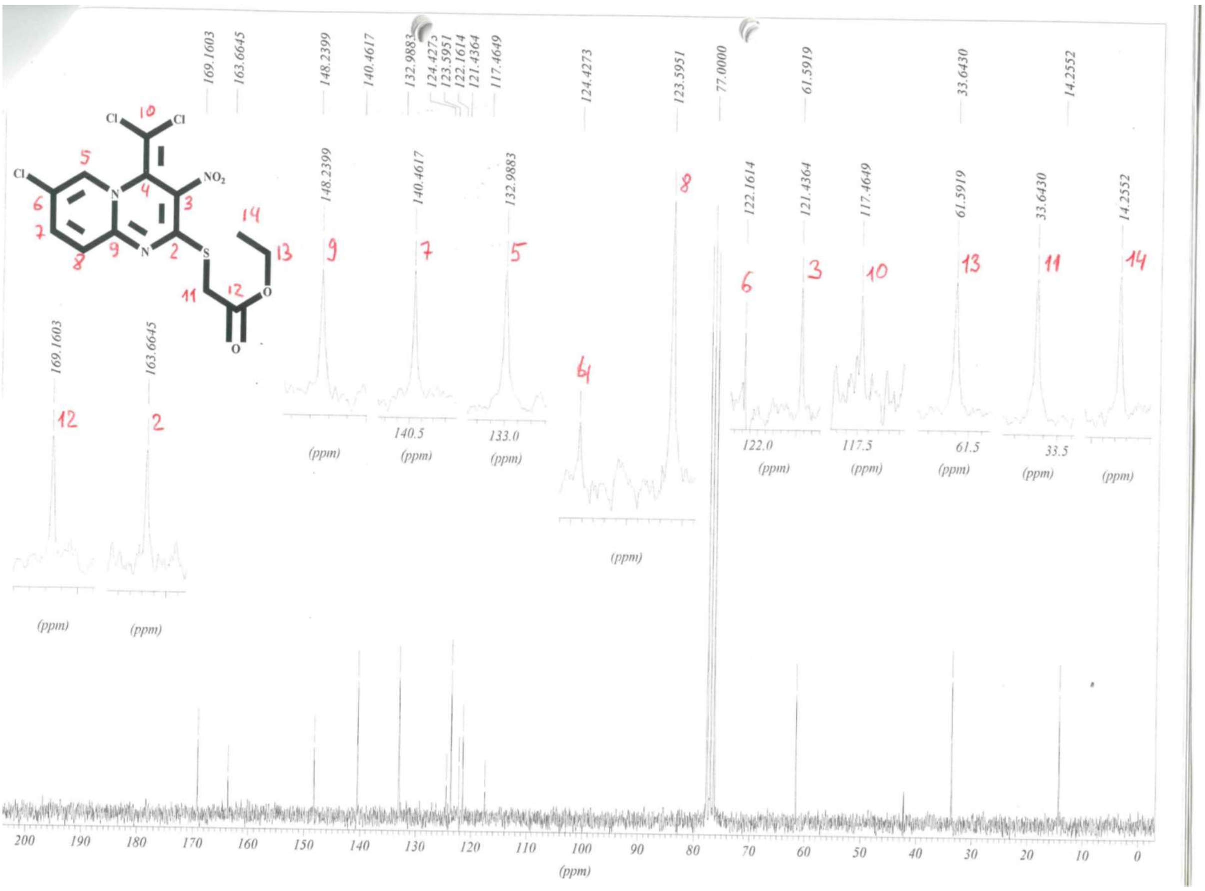

Figure S133. HR-MS spectrum for ethyl {[7-chloro-4-(dichloromethylidene)-3-nitro-4*H*-pyrido[1,2-*a*]pyrimidin-2-yl]sulfanyl}acetate (**33b**).

Elemental Composition Report

Page 1

Single Mass Analysis (displaying only valid results)

Tolerance = 10.0 PPM / DBE: min = -1.5, max = 50.0
Selected filters: None

Monoisotopic Mass, Even Electron Ions
3110 formula(e) evaluated with 21 results within limits (up to 80 closest results for each mass)
Elements Used:
C: 0-50 H: 0-60 N: 0-3 O: 0-7 Na: 0-1 S: 0-2 Cl: 0-3

Zapolski LCT Premier KD070
VZ 1456 22 (0.492) AM (Cen,4, 70.00, Ar,11000.0,556.28,0.70,LS 5) 1: TOF MS ES+ 808

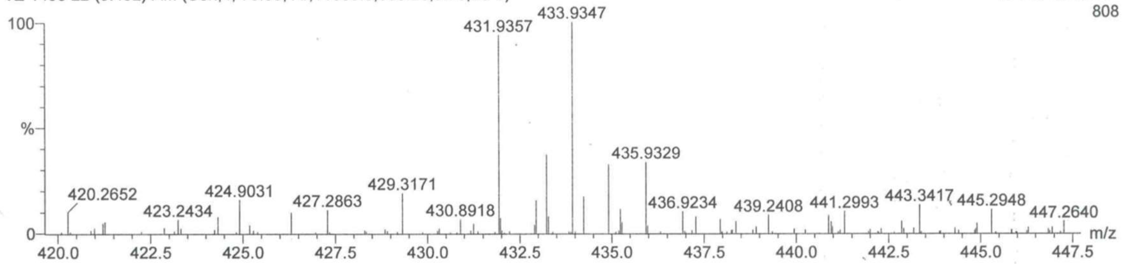

Minimum:				-1.5		
Maximum:		5.0	10.0	50.0		

Mass	Calc. Mass	mDa	PPM	DBE	i-FIT	Formula
431.9357	431.9355	0.2	0.5	8.5	0.5	C13 H10 N3 O4 Na S Cl3
	431.9361	-0.4	-0.9	17.5	322.7	C17 H3 N3 O6 Na S2
	431.9362	-0.5	-1.2	17.5	15.2	C21 H6 N O2 Na Cl3
	431.9346	1.1	2.5	17.5	160.0	C19 H4 N O6 Na S Cl
	431.9346	1.1	2.5	16.5	8.2	C18 H5 N3 O4 Cl3
	431.9368	-1.1	-2.5	25.5	83.5	C24 N3 O2 Cl2
	431.9345	1.2	2.8	24.5	176.1	C25 H3 N O S2 Cl
	431.9344	1.3	3.0	22.5	72.8	C22 H N3 O2 Na Cl2
	431.9370	-1.3	-3.0	20.5	168.2	C21 H3 N O6 S Cl
	431.9377	-2.0	-4.6	17.5	51.3	C19 H5 N3 O2 Na S Cl2
	431.9379	-2.2	-5.1	11.5	2.1	C15 H9 N3 O4 S Cl3
	431.9379	-2.2	-5.1	12.5	131.2	C16 H8 N O6 Na S2 Cl
	431.9384	-2.7	-6.3	26.5	98.1	C27 H N Na Cl2
	431.9385	-2.8	-6.5	20.5	326.8	C19 H2 N3 O6 S2
	431.9386	-2.9	-6.7	20.5	22.8	C23 H5 N O2 Cl3
	431.9389	-3.2	-7.4	3.5	1.1	C10 H14 N3 O4 Na S2 Cl3
	431.9323	3.4	7.9	15.5	42.4	C19 H8 N O3 S2 Cl2
	431.9322	3.5	8.1	13.5	4.4	C16 H6 N3 O4 Na Cl3
	431.9321	3.6	8.3	21.5	165.5	C23 H4 N O Na S2 Cl
	431.9396	-3.9	-9.0	12.5	6.6	C18 H10 N O2 Na S Cl3
	431.9399	-4.2	-9.7	26.5	196.7	C25 N3 Na S Cl

Figure S134. 400 MHz ¹H-NMR spectrum in $CDCl_3$ for **35a**.

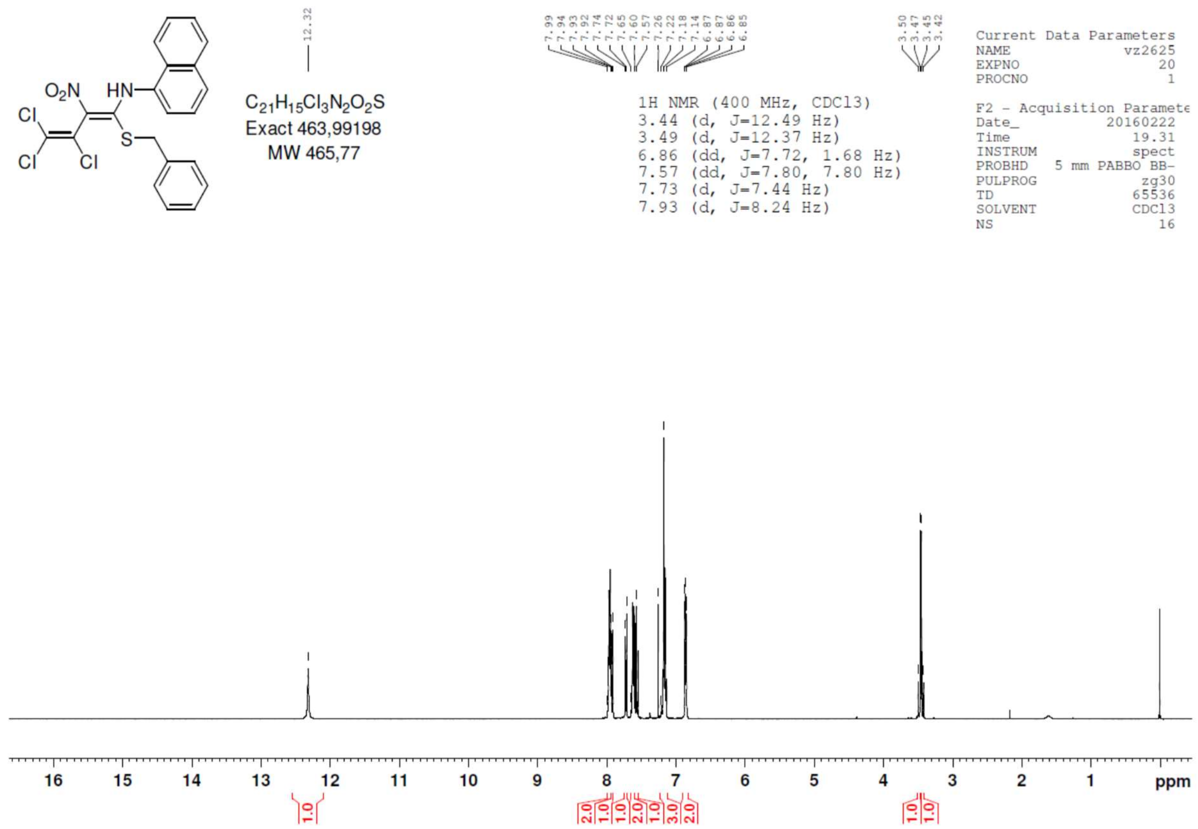

Figure S135. 100 MHz ¹³C-NMR spectrum in $CDCl_3$ for **35a**.

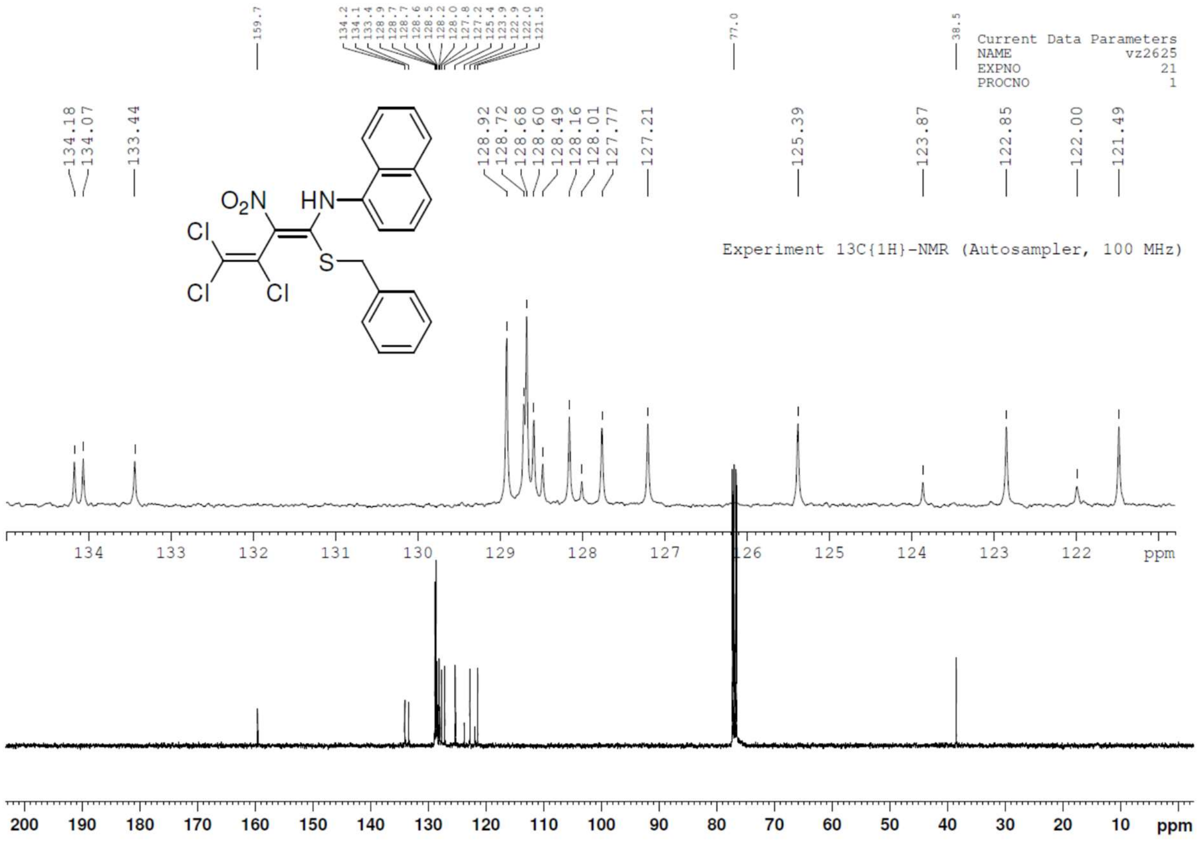

Figure S136. 400 MHz ^{1}H-NMR spectrum in $CDCl_3$ for **35b**.

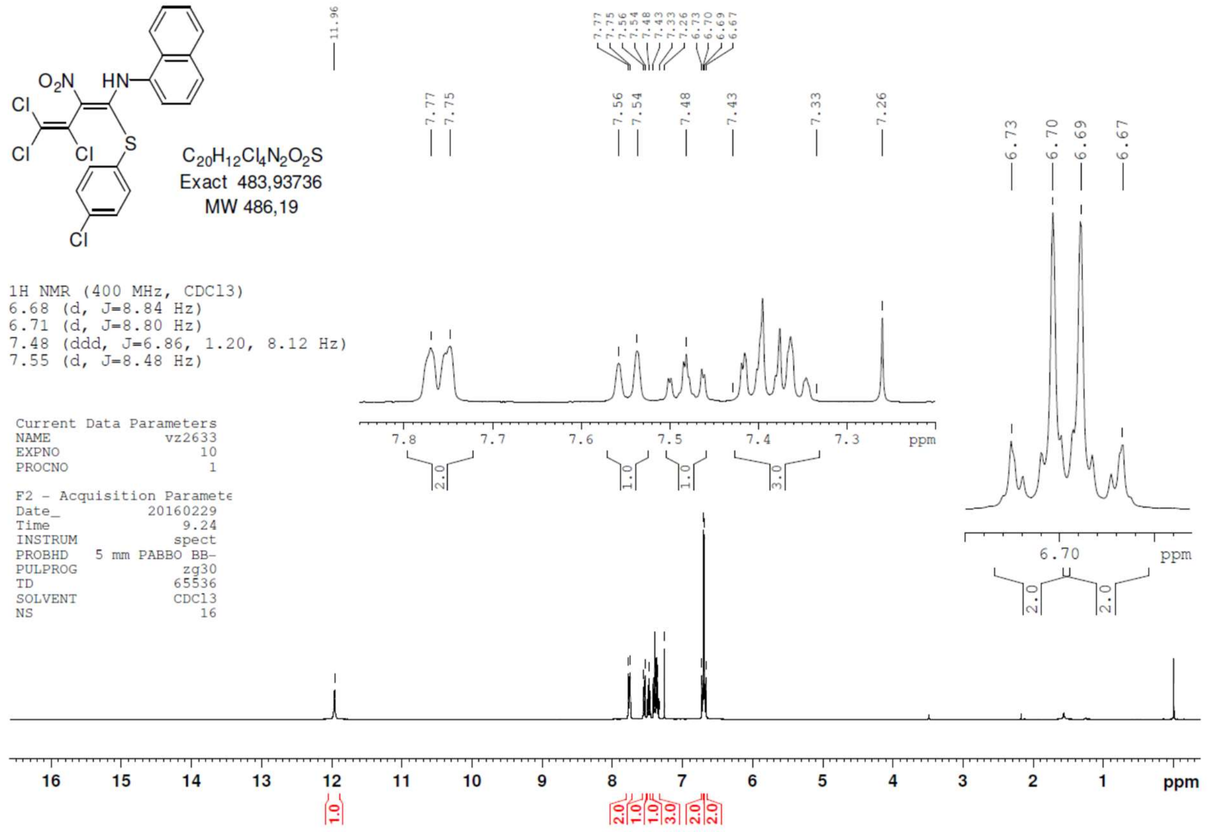

Figure S137. 100 MHz ^{13}C-NMR spectrum in $CDCl_3$ for **35b**.

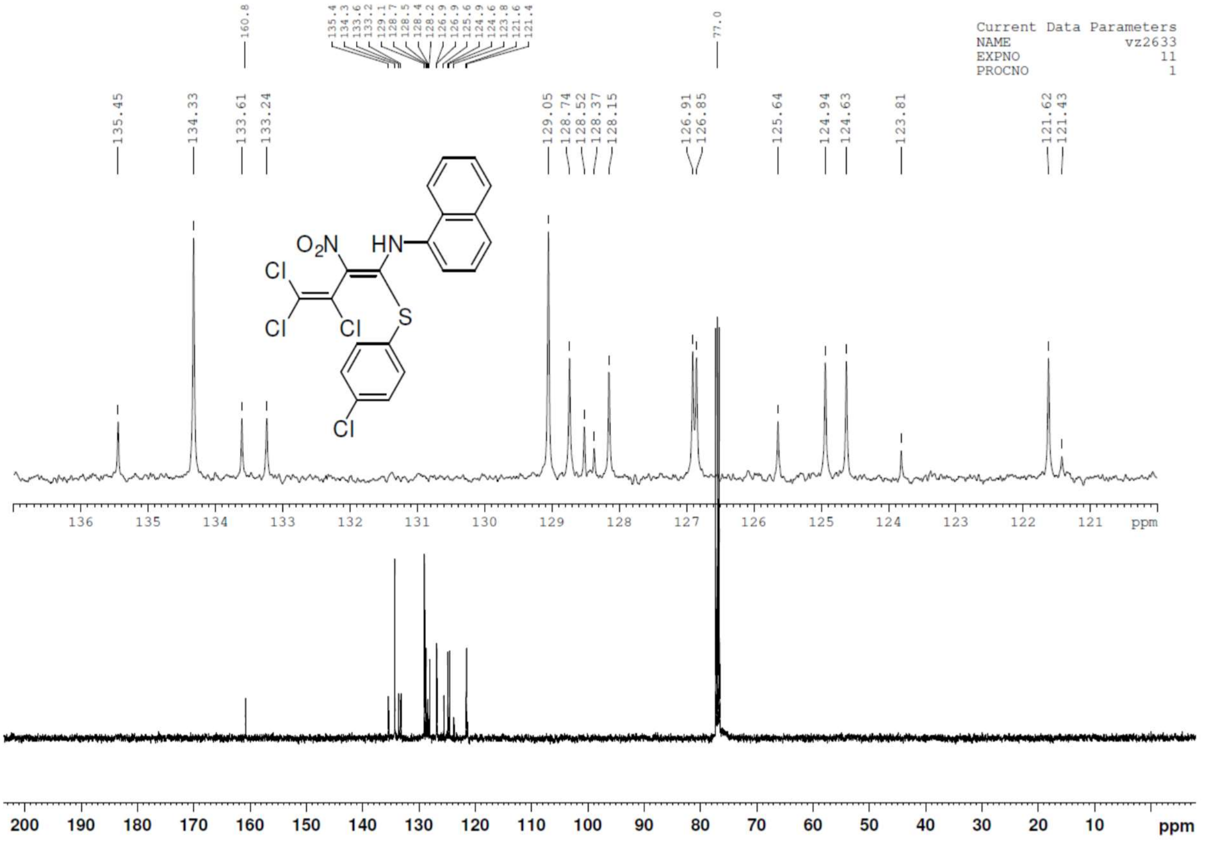

Figure S138. 400 MHz ^{1}H-NMR spectrum in $CDCl_3$ for **36a.**

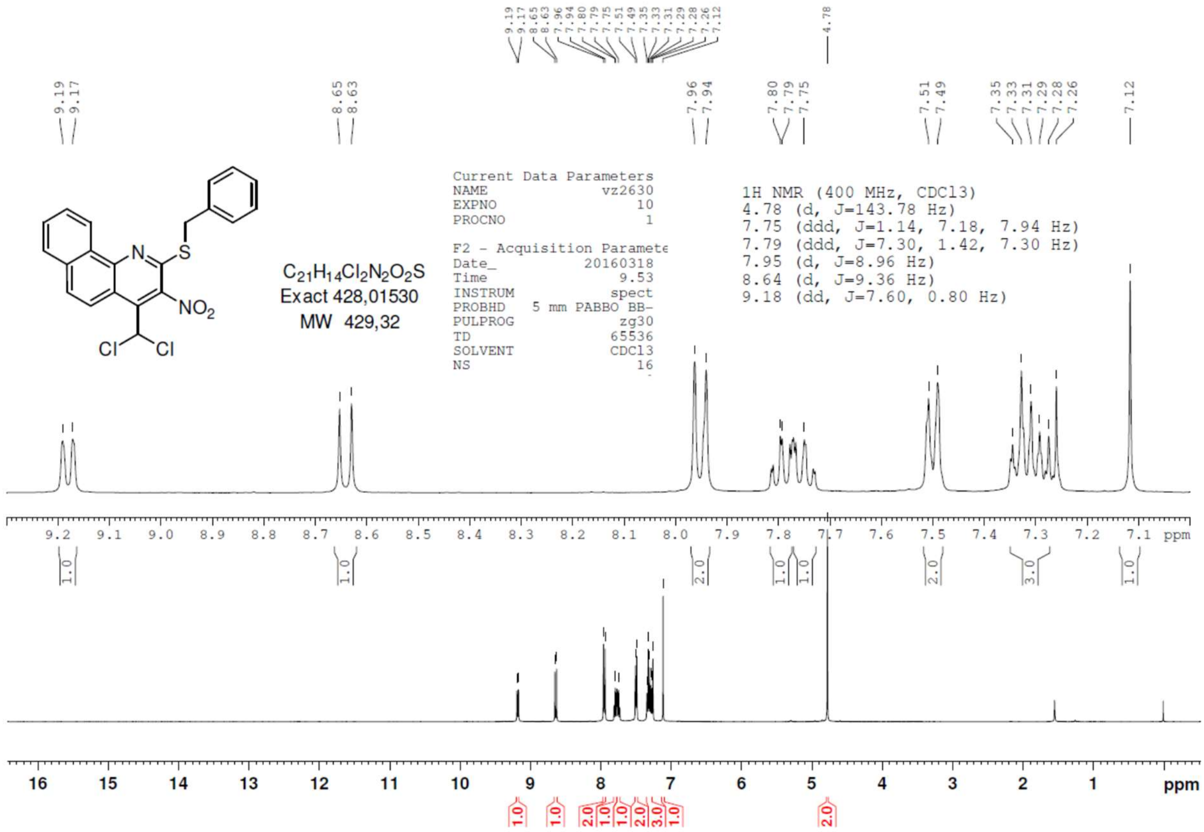

Figure S139. 100 MHz ^{13}C-NMR spectrum in $CDCl_3$ for **36a.**

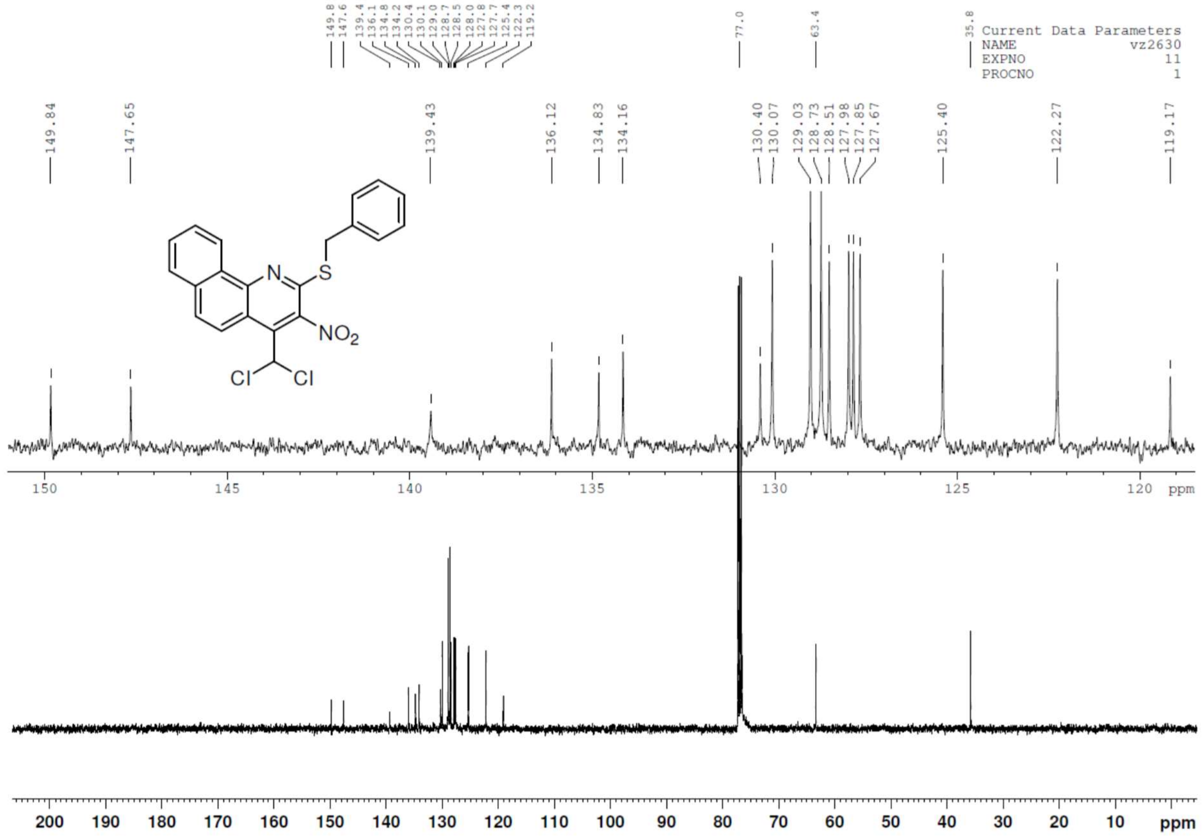

Figure S140. 400 MHz ^{1}H-NMR spectrum in $CDCl_3$ for **36b**.

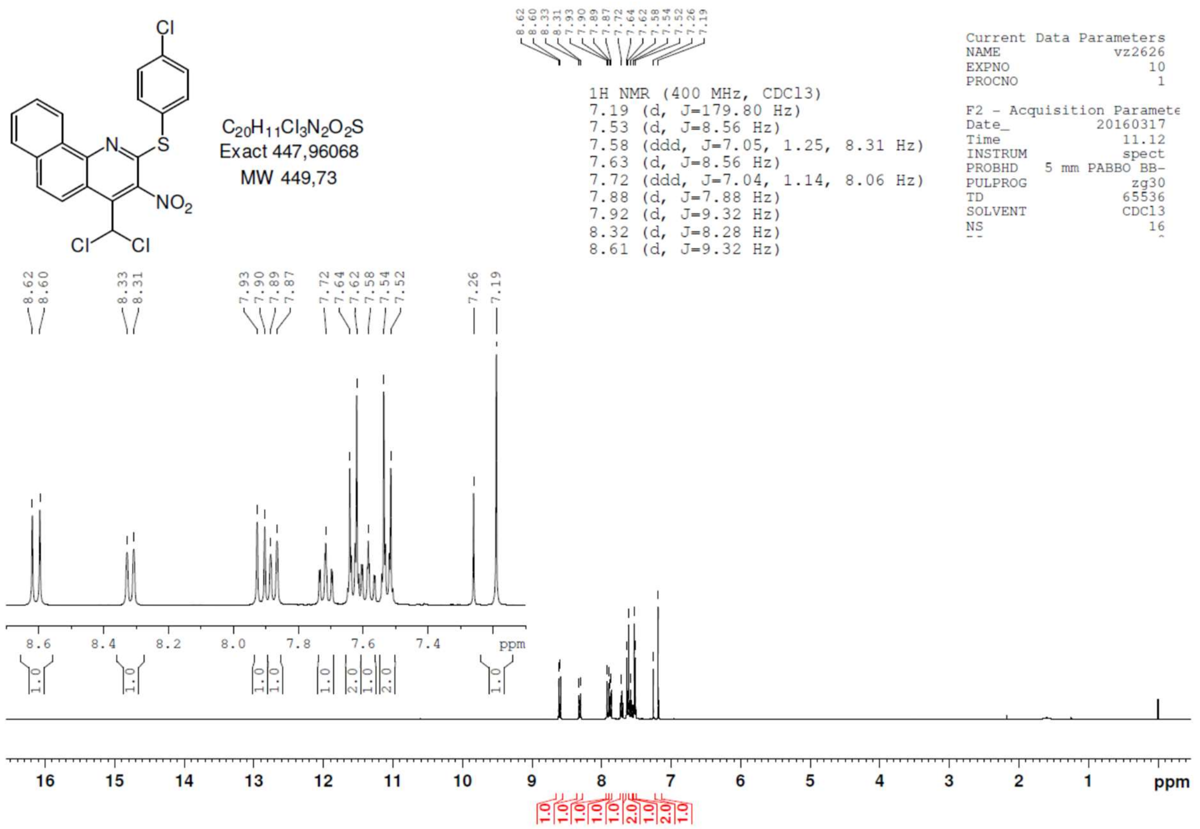

Figure S141. 100 MHz ^{13}C-NMR spectrum in $CDCl_3$ for **36b**.

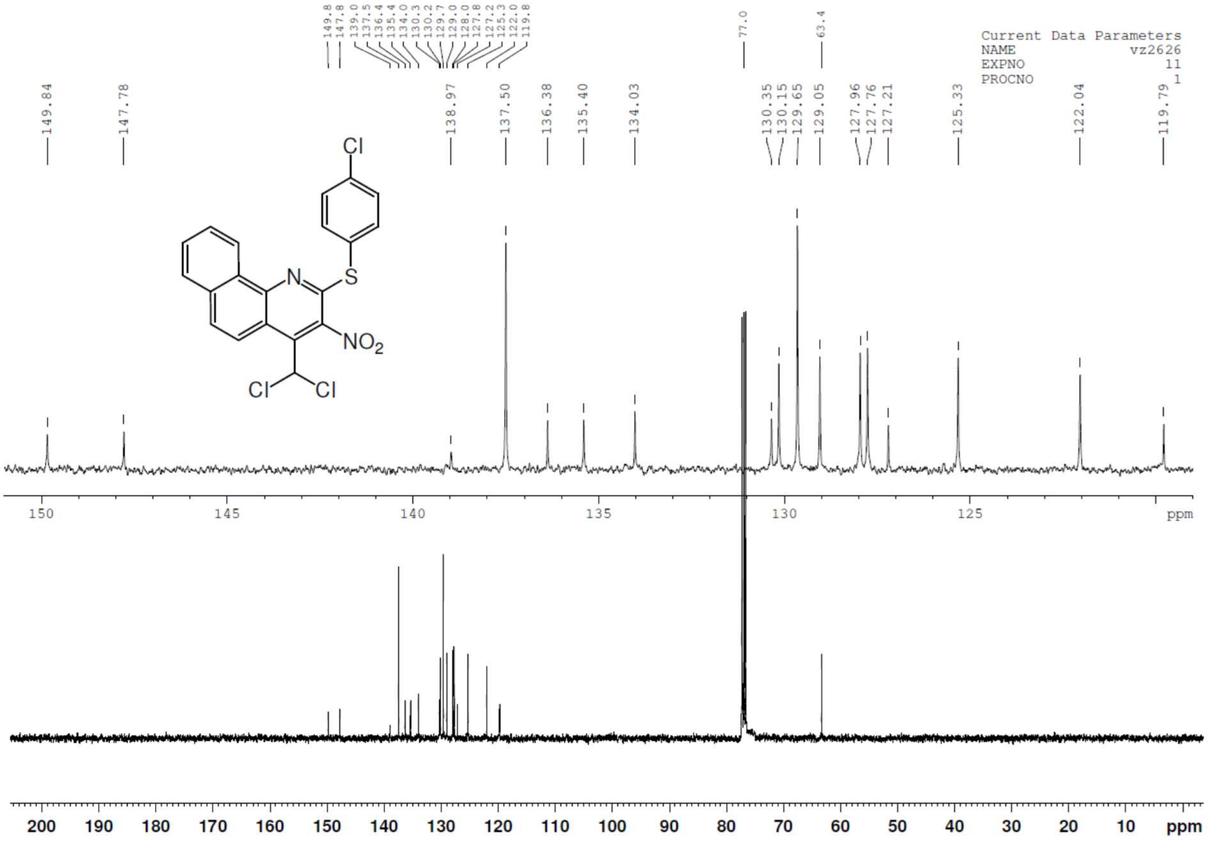

Figure S142. 400 MHz ^{1}H-NMR spectrum in $CDCl_3$ for **37a**.

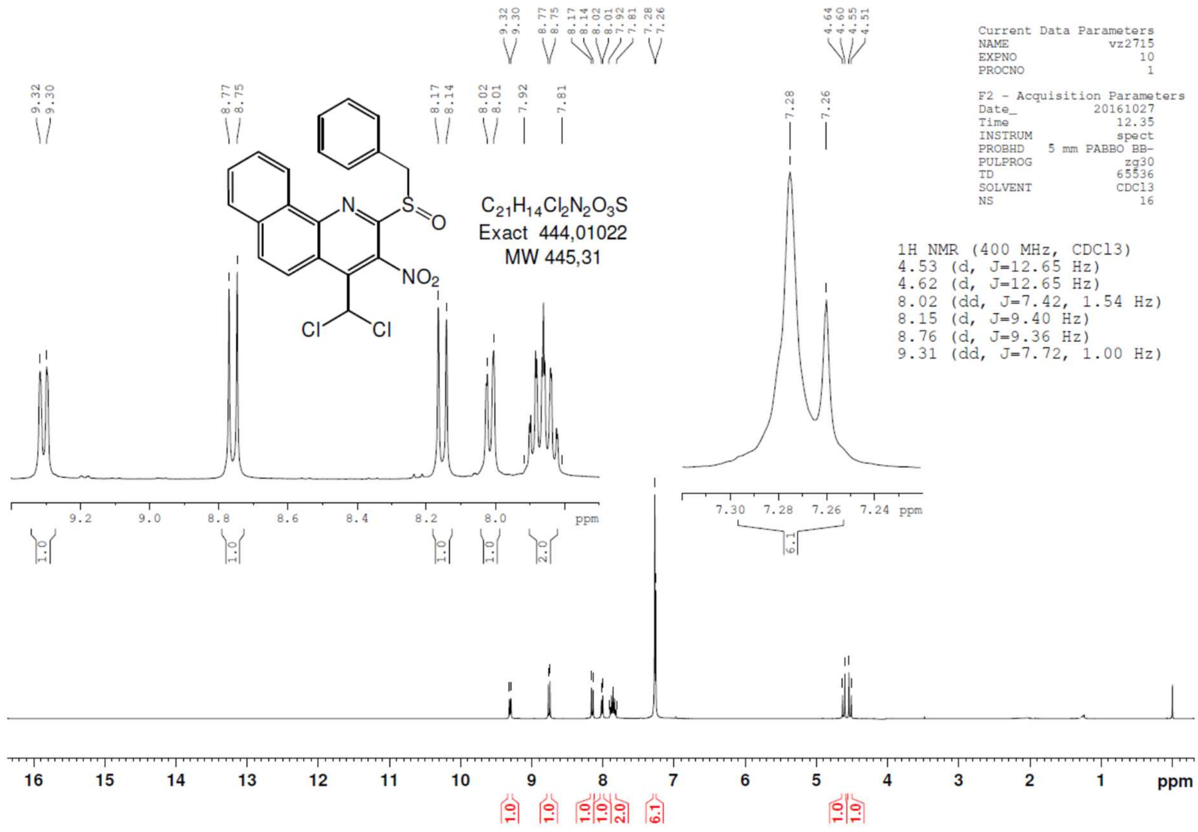

Figure S143. 100 MHz ^{13}C-NMR spectrum in $CDCl_3$ for **37a**.

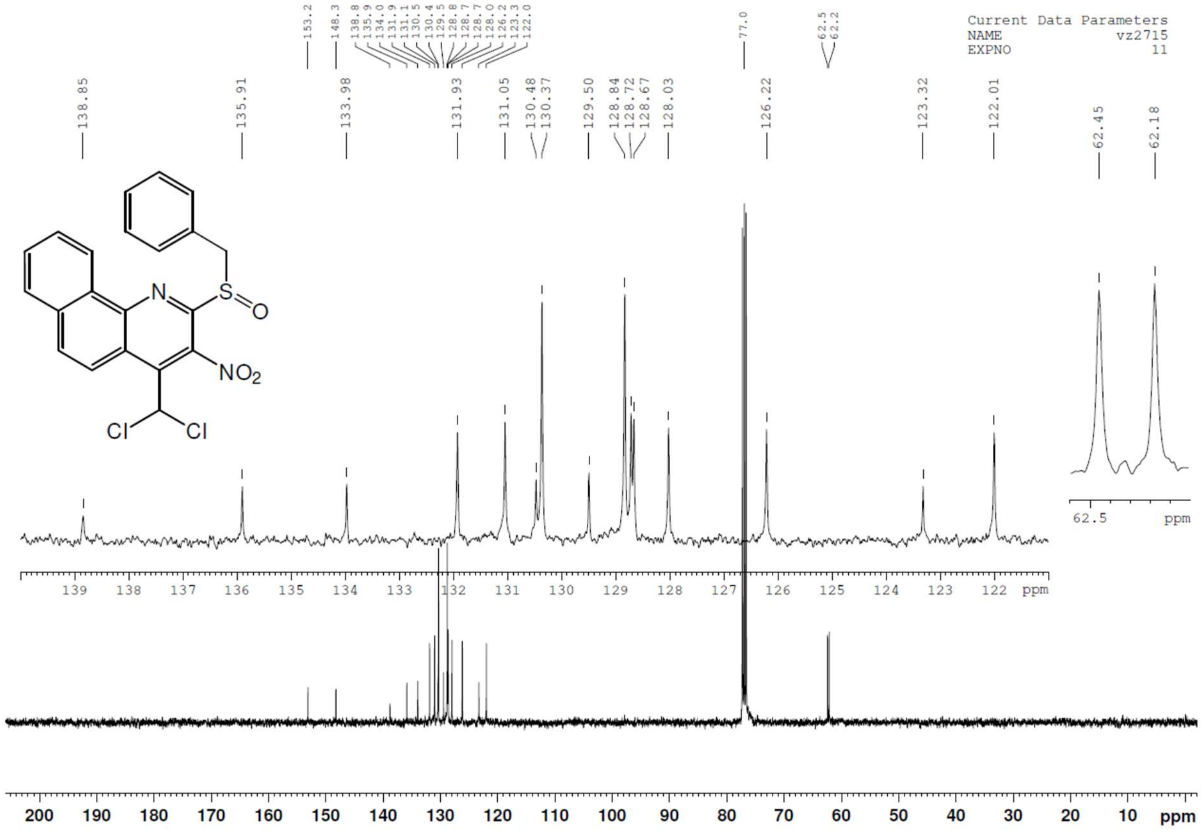

Figure S144. 400 MHz ^{1}H-NMR spectrum in $CDCl_3$ for **37b**.

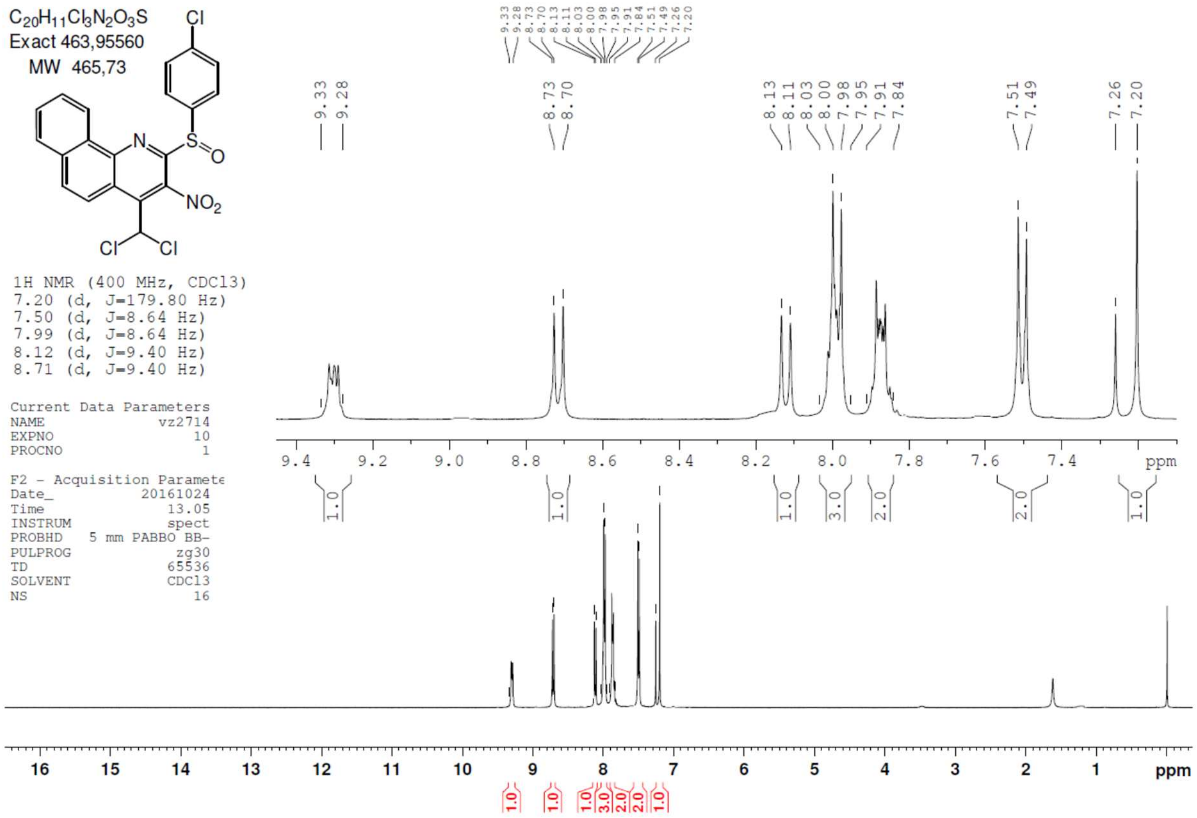

Figure S145. 100 MHz ^{13}C-NMR spectrum in $CDCl_3$ for **37b**.

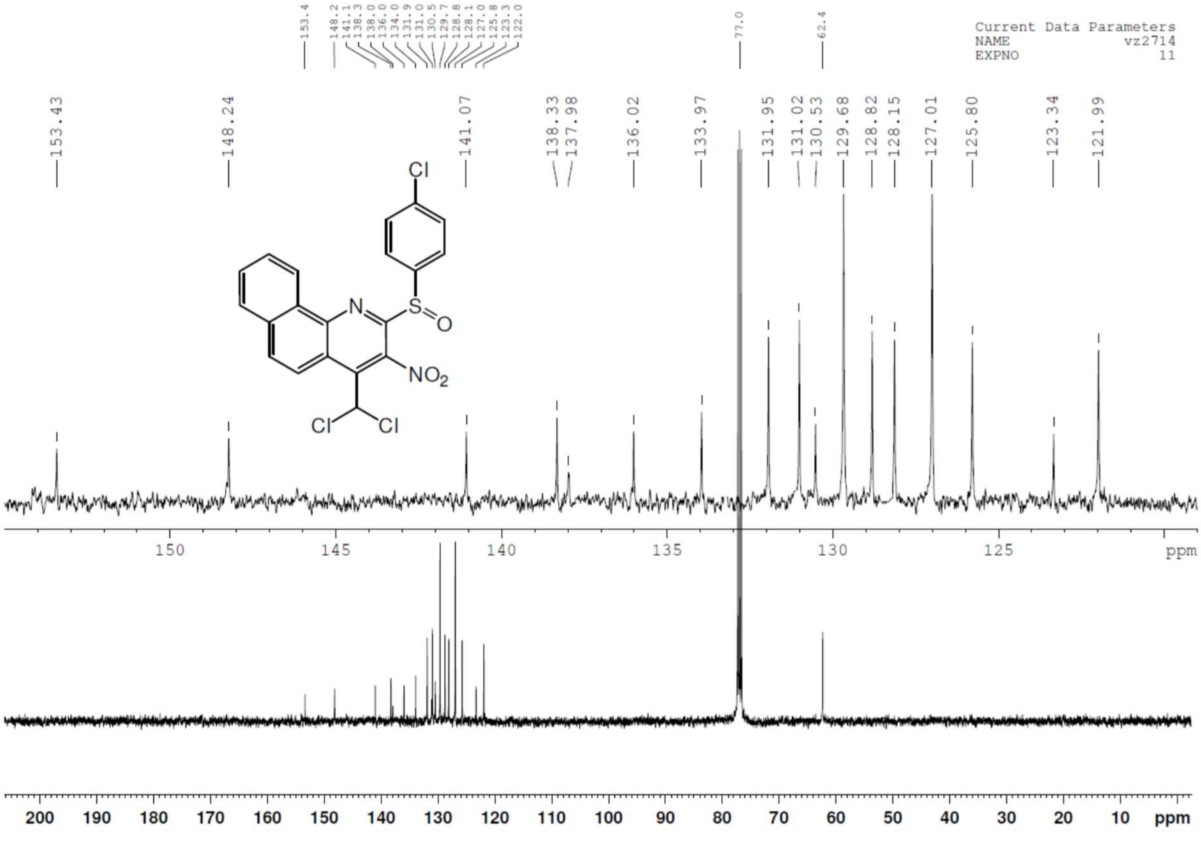

Figure S146. 400 MHz ^{1}H-NMR spectrum in $CDCl_3$ for **38**.

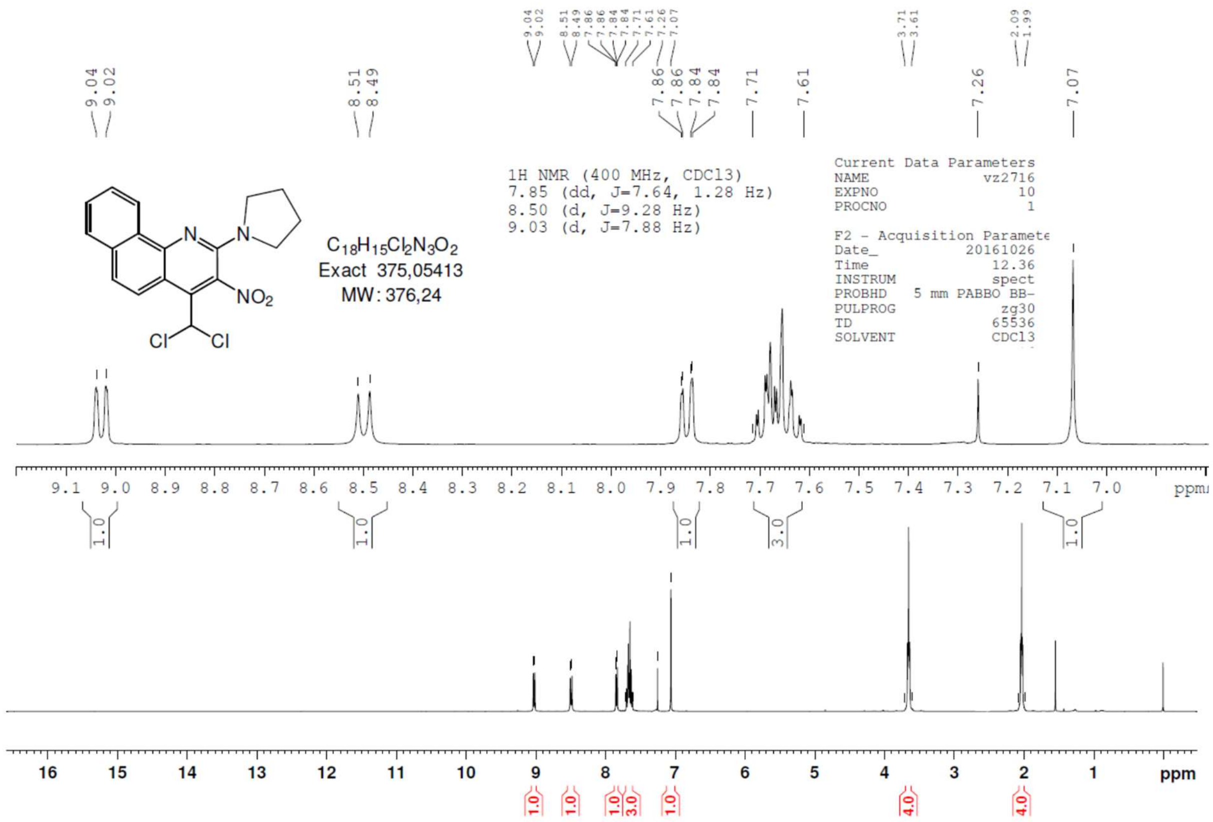

Figure S147. 100 MHz ^{13}C-NMR spectrum in $CDCl_3$ for **38**.

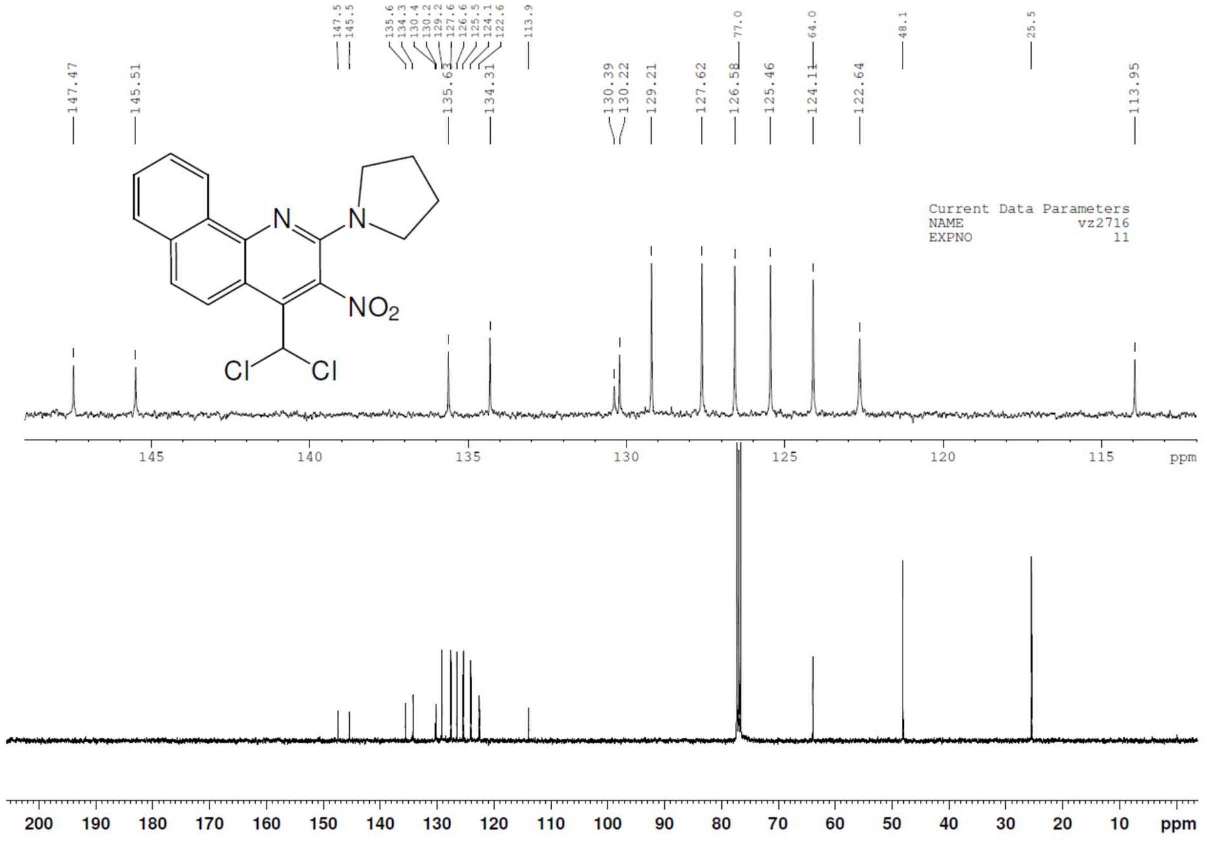

Figure S148. 200 MHz ¹H-NMR spectrum in $CDCl_3$ for **41**.

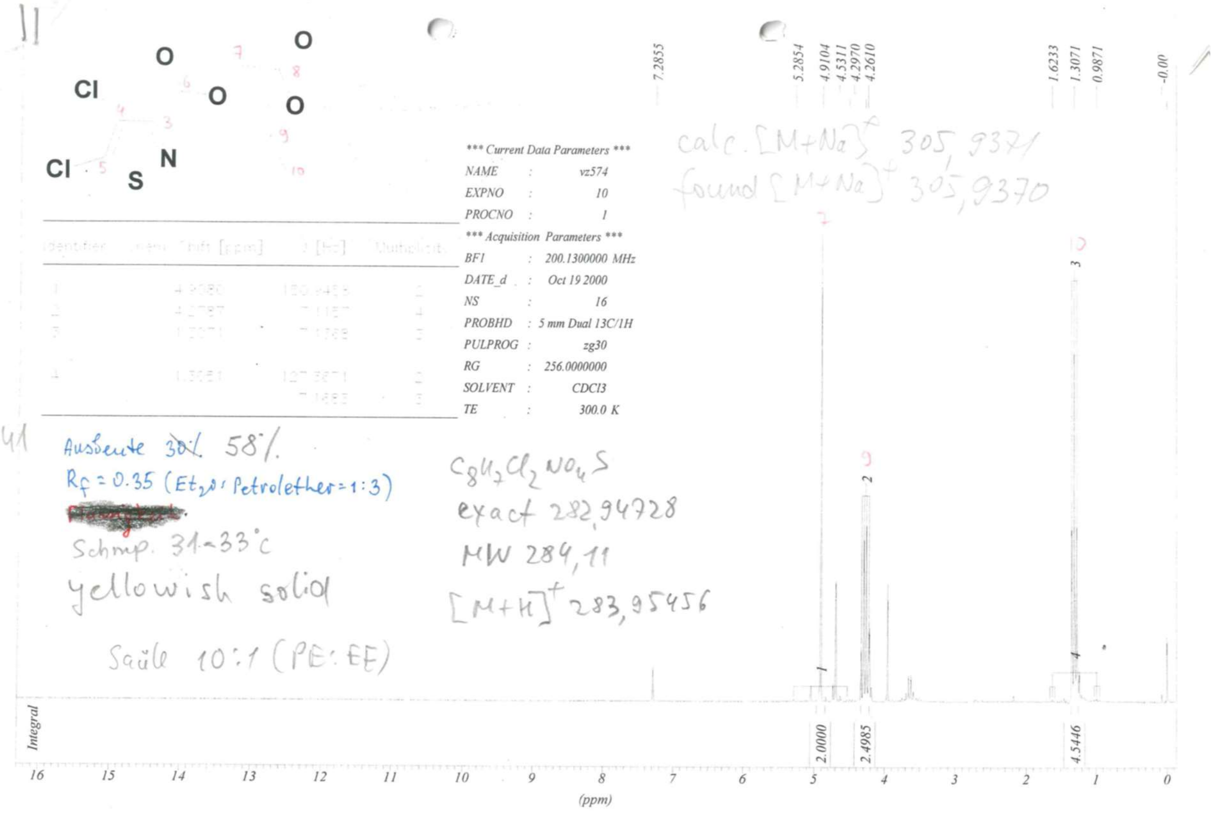

Figure S149. 50 MHz ¹³C-NMR spectrum in $CDCl_3$ for **41**.

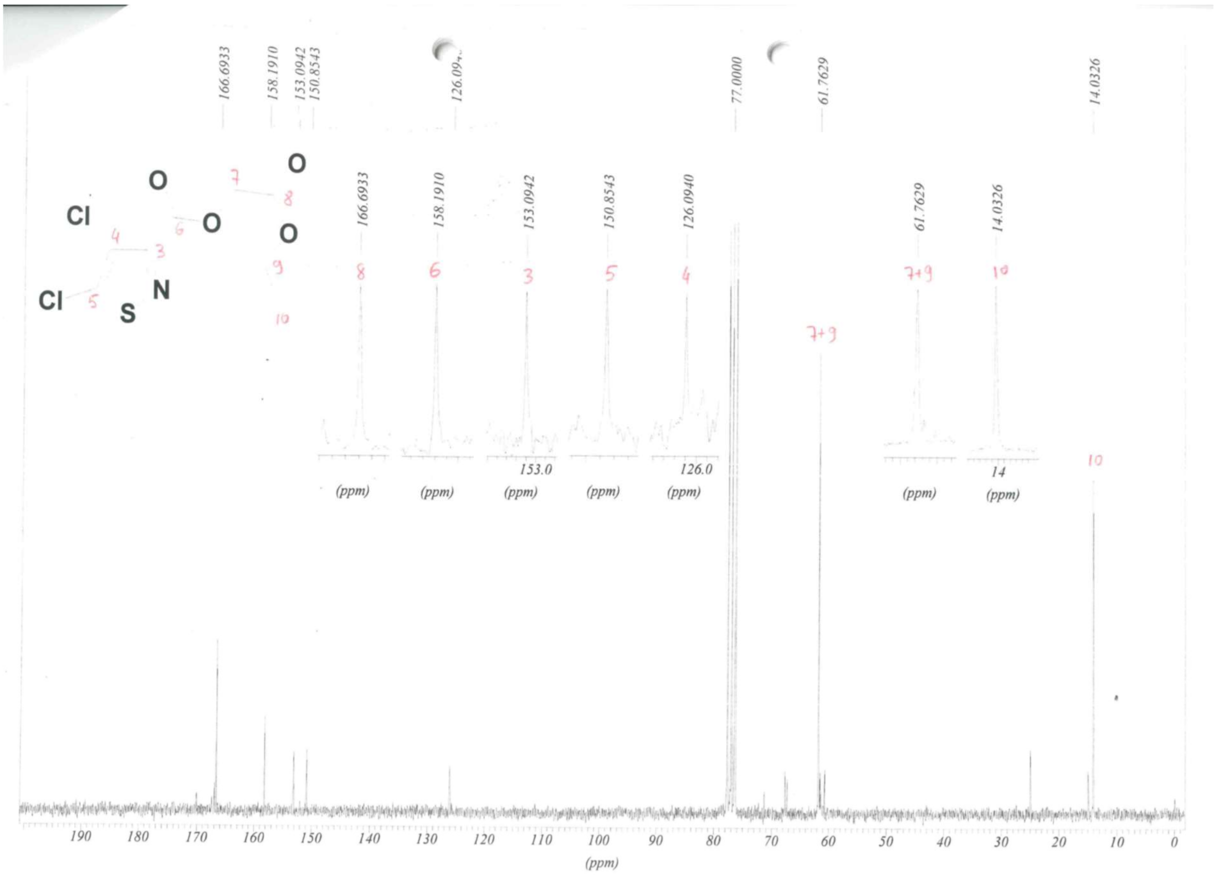

Figure S150. 200 MHz ^{1}H-NMR spectrum in $CDCl_3$ for **43**.

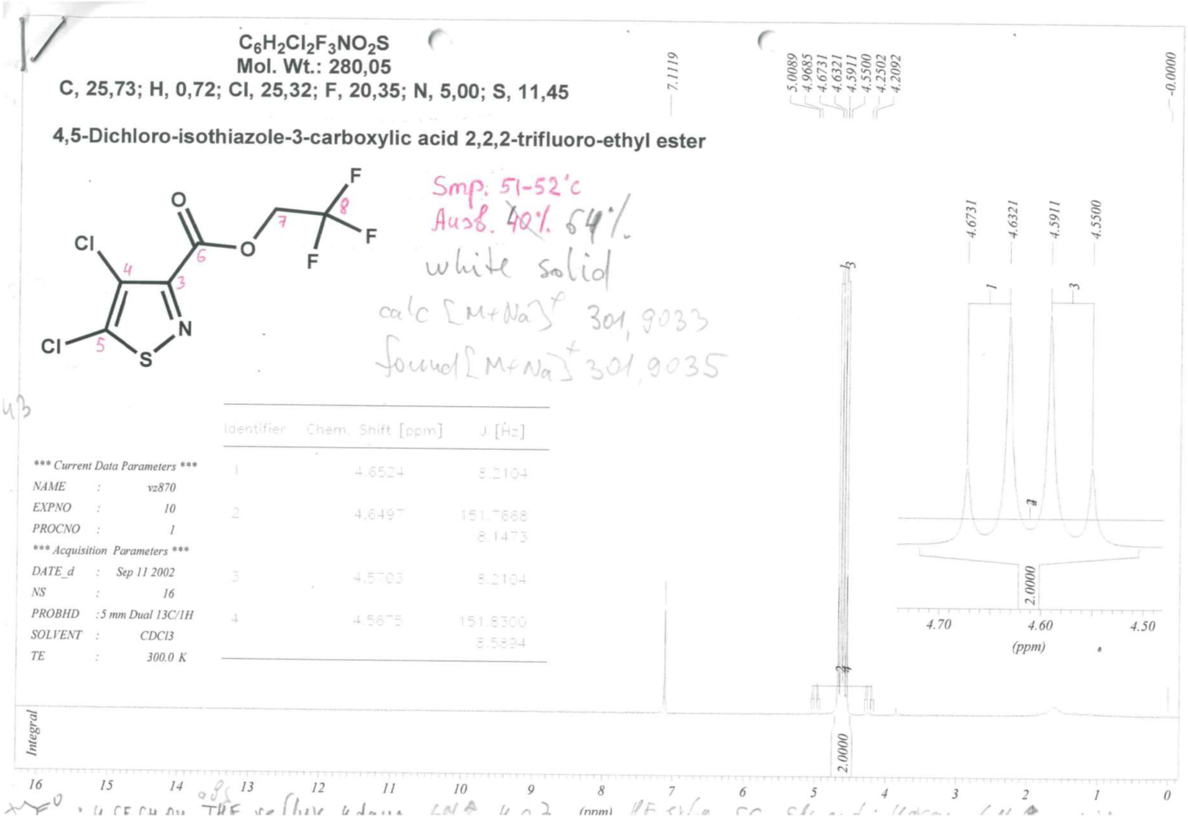

Figure S151. 50 MHz ^{13}C-NMR spectrum in $CDCl_3$ for **43**.

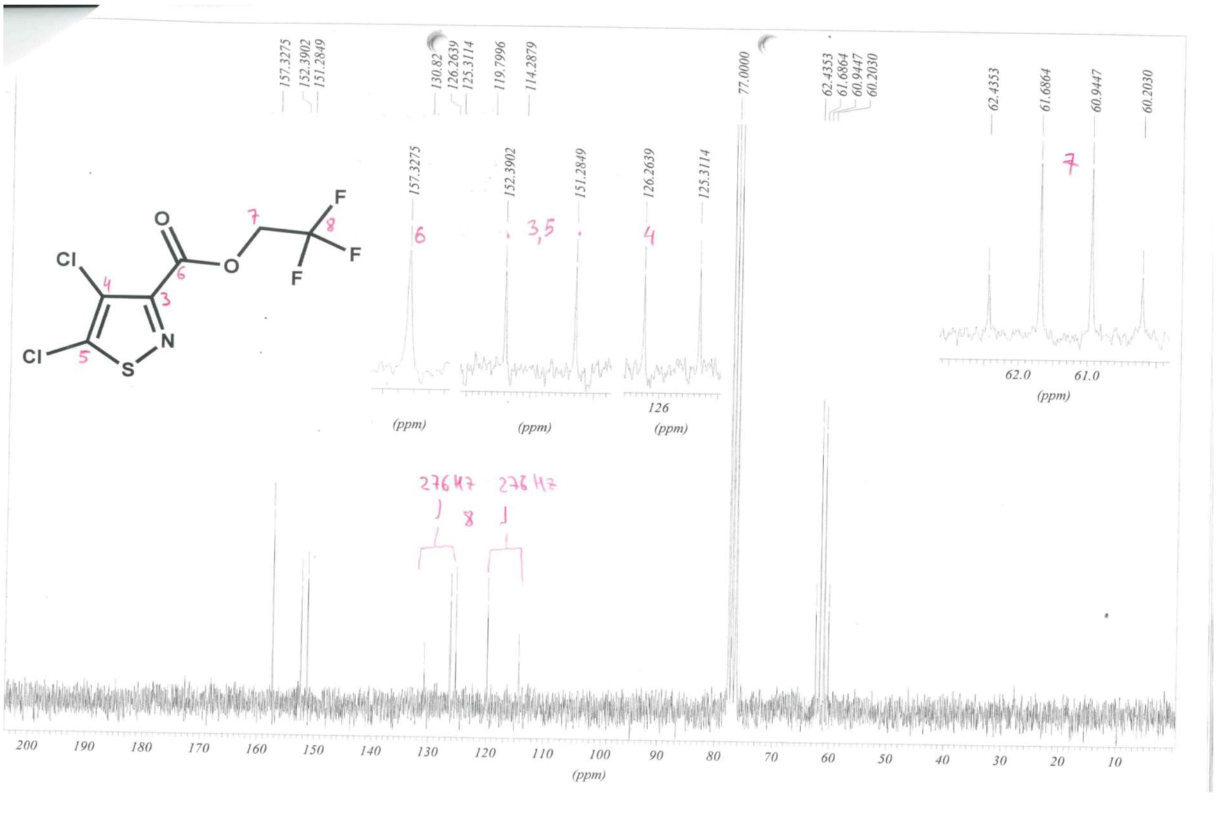

Figure S152. 200 MHz ^{1}H-NMR spectrum in $CDCl_3$ for **44a**.

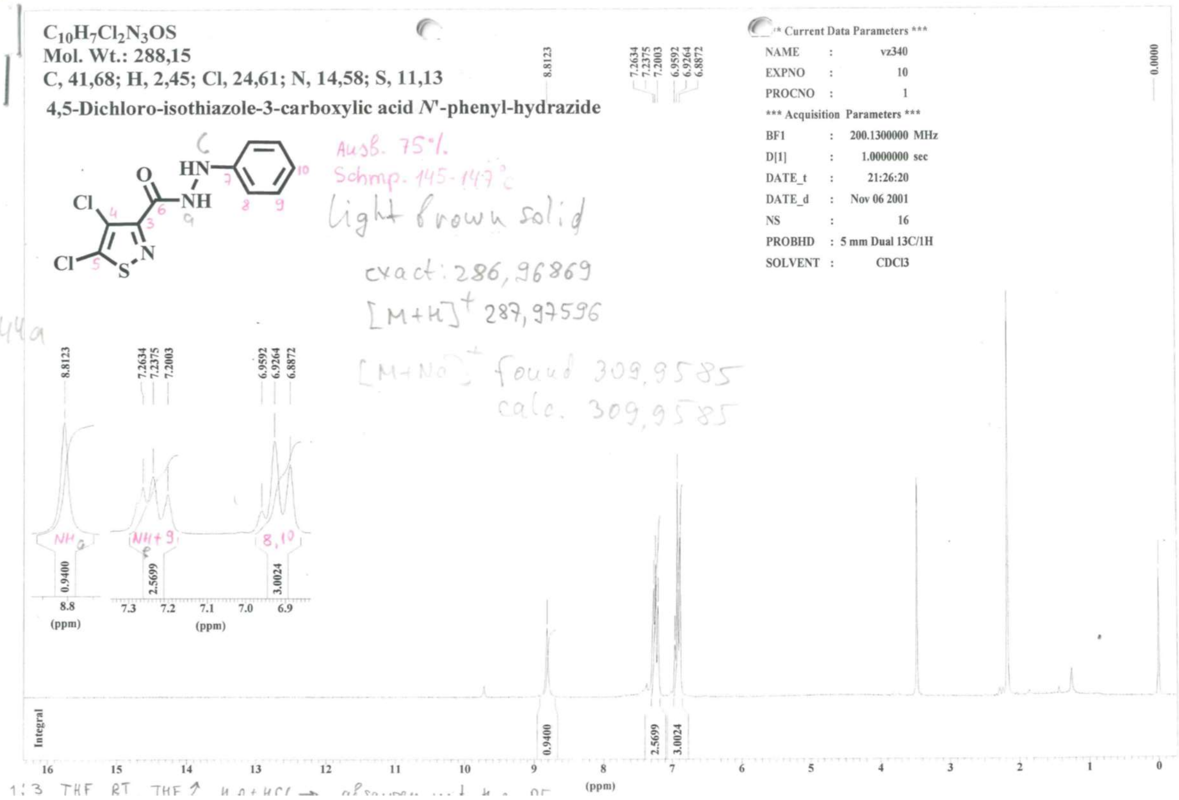

Figure S153. 50 MHz ^{13}C-NMR spectrum in $CDCl_3$ for **44a**.

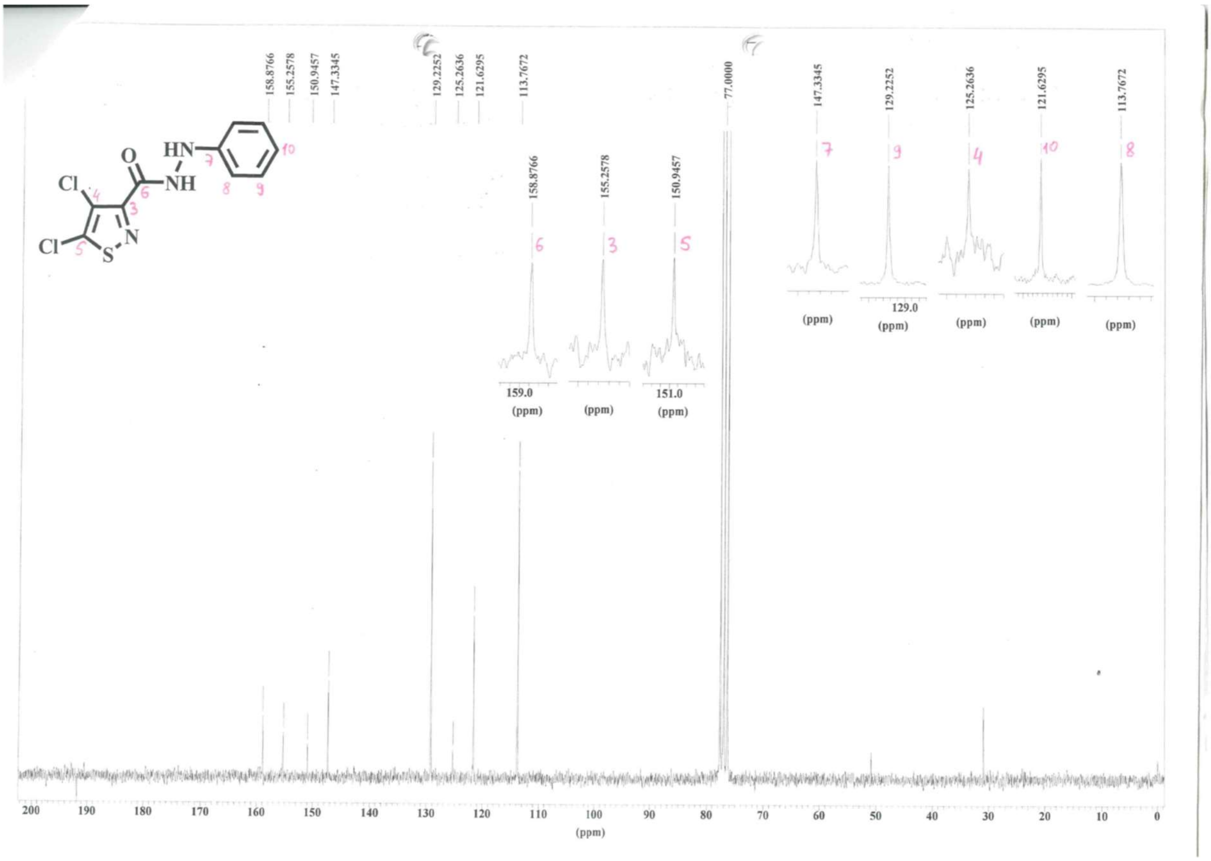

Figure S154. MS spectrum for 4,5-dichloro-N'-phenyl-1,2-thiazole-3-carbohydrazide (**44a**).

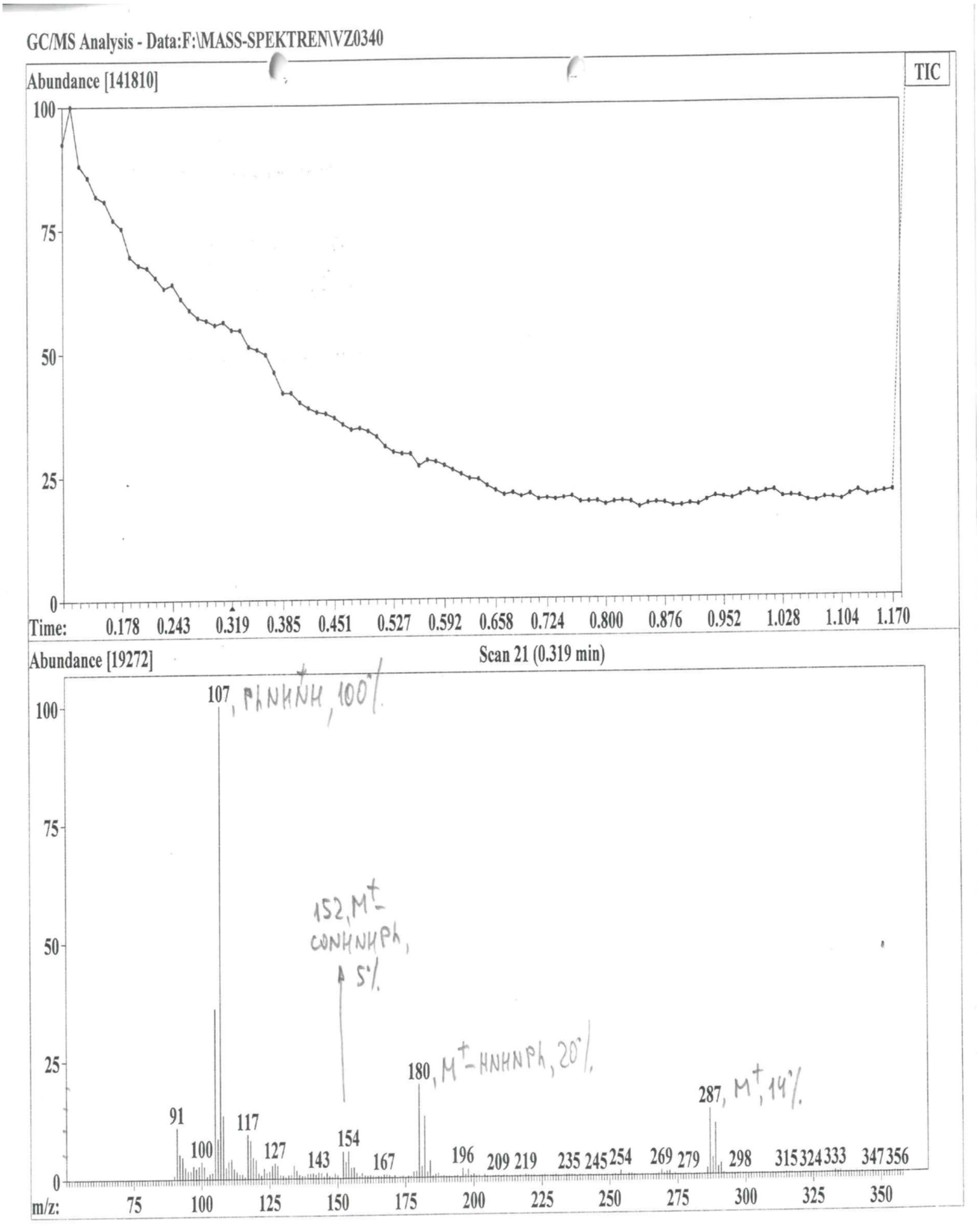

Figure S155. 200 MHz ^{1}H-NMR spectrum in DMSO-*d6* for **44b**.

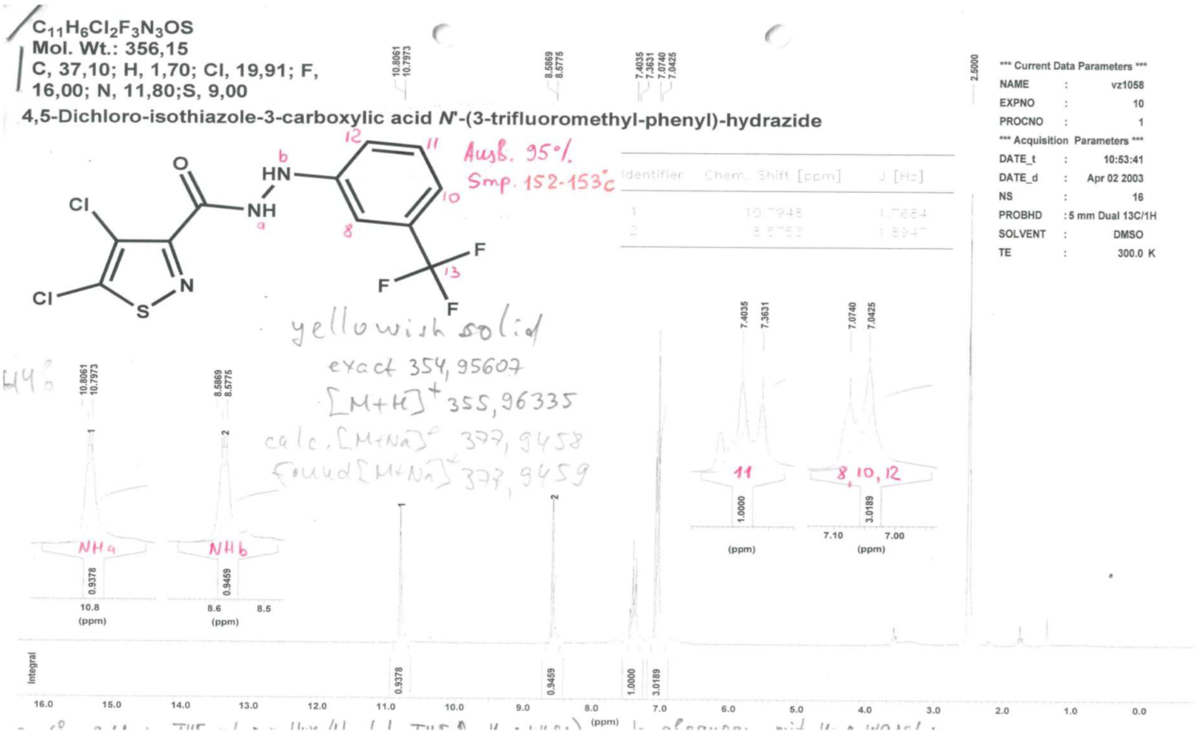

Figure S156. 50 MHz ^{13}C-NMR spectrum in DMSO-*d6* for **44b**.

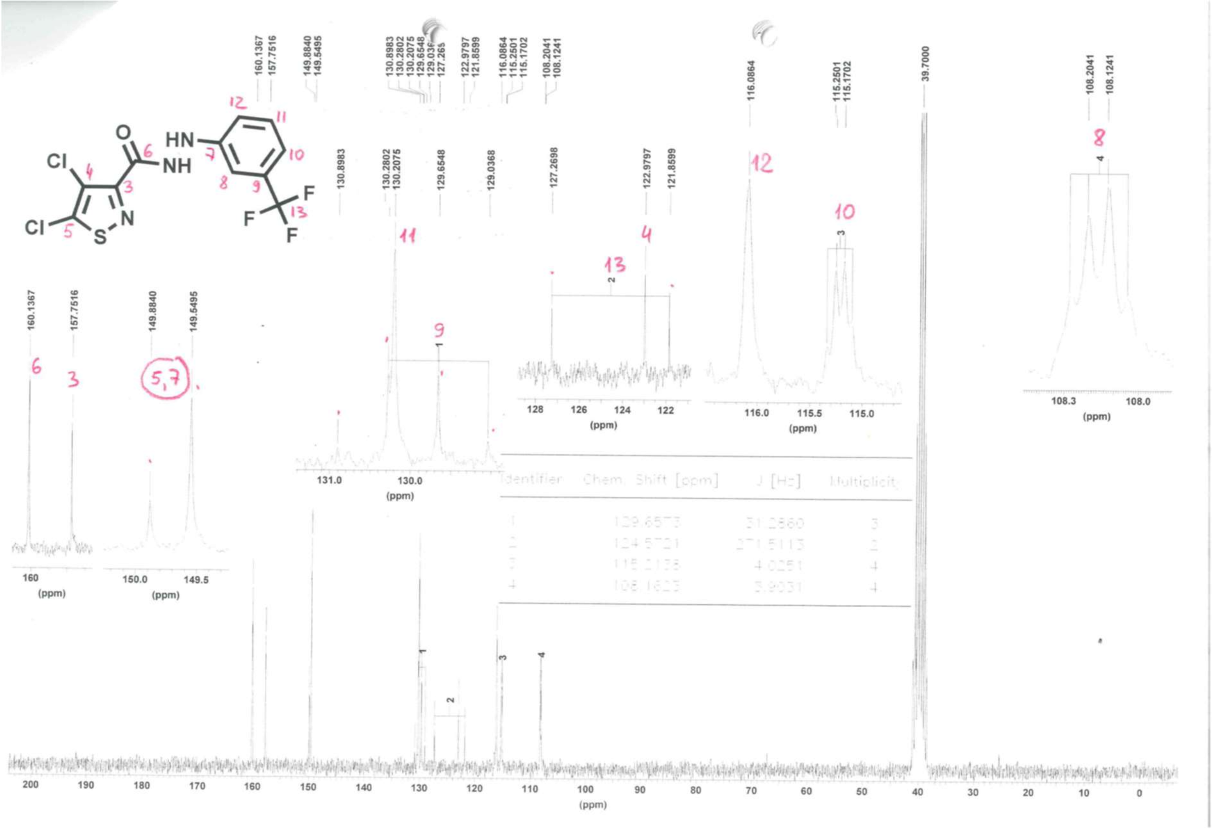

Figure S157. 200 MHz ^{1}H-NMR spectrum in $CDCl_3$ for **44c**.

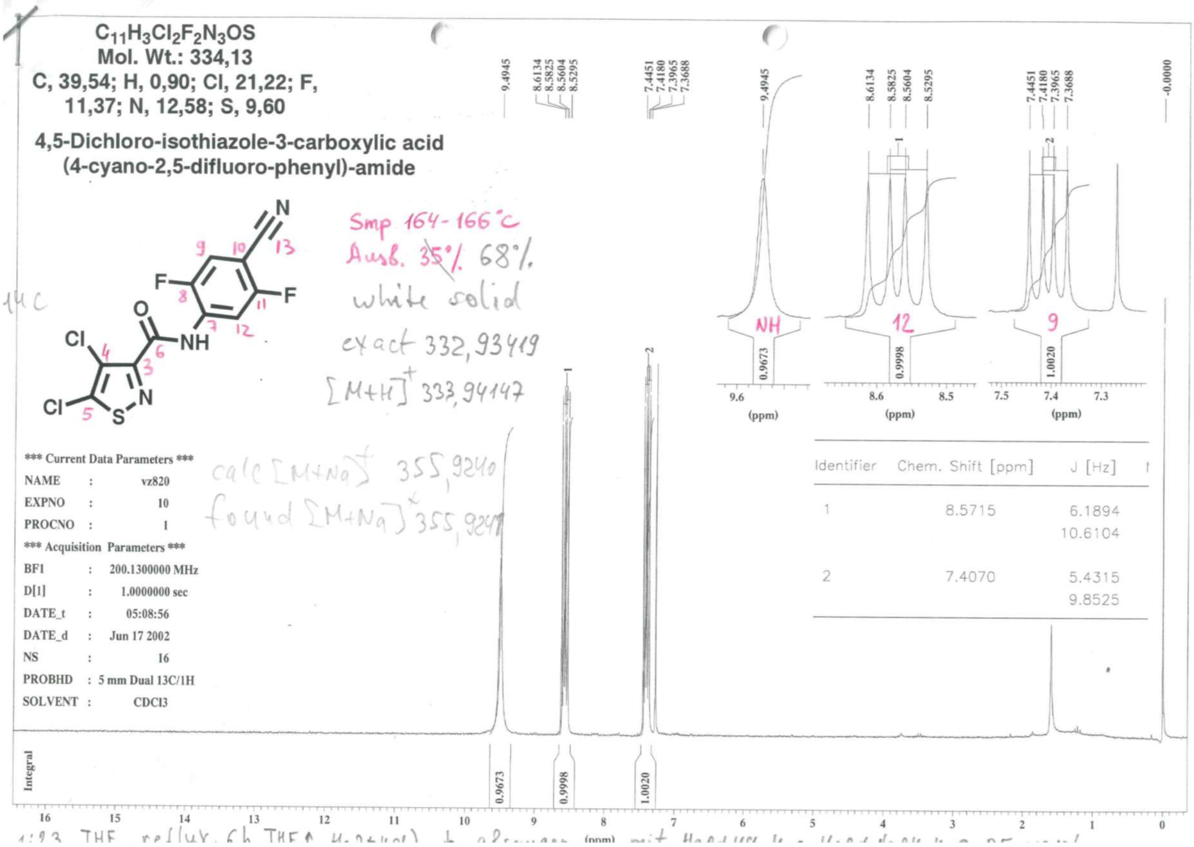

Figure S158. 50 MHz ^{13}C-NMR spectrum in $CDCl_3$ for **44c**.

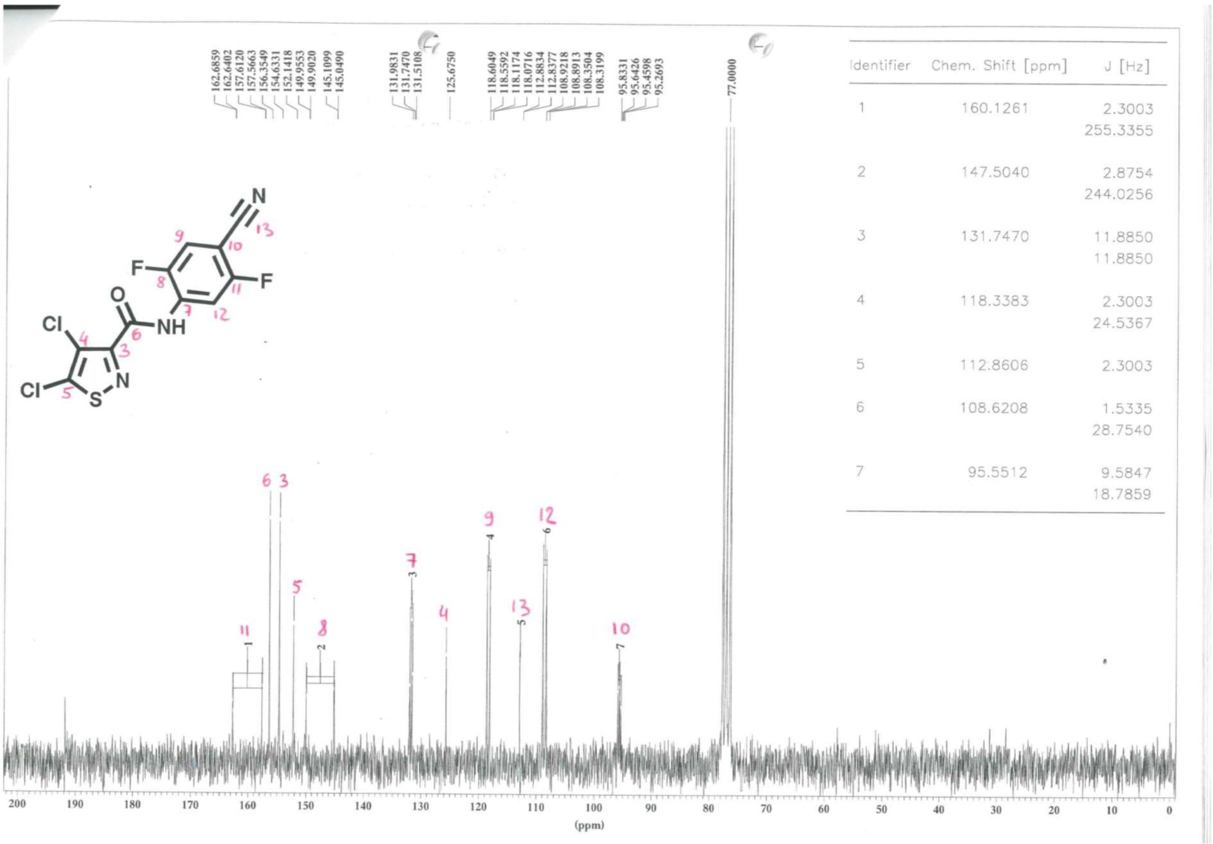

Figure S159. 200 MHz ^{1}H-NMR spectrum in $CDCl_3$ for **44d**.

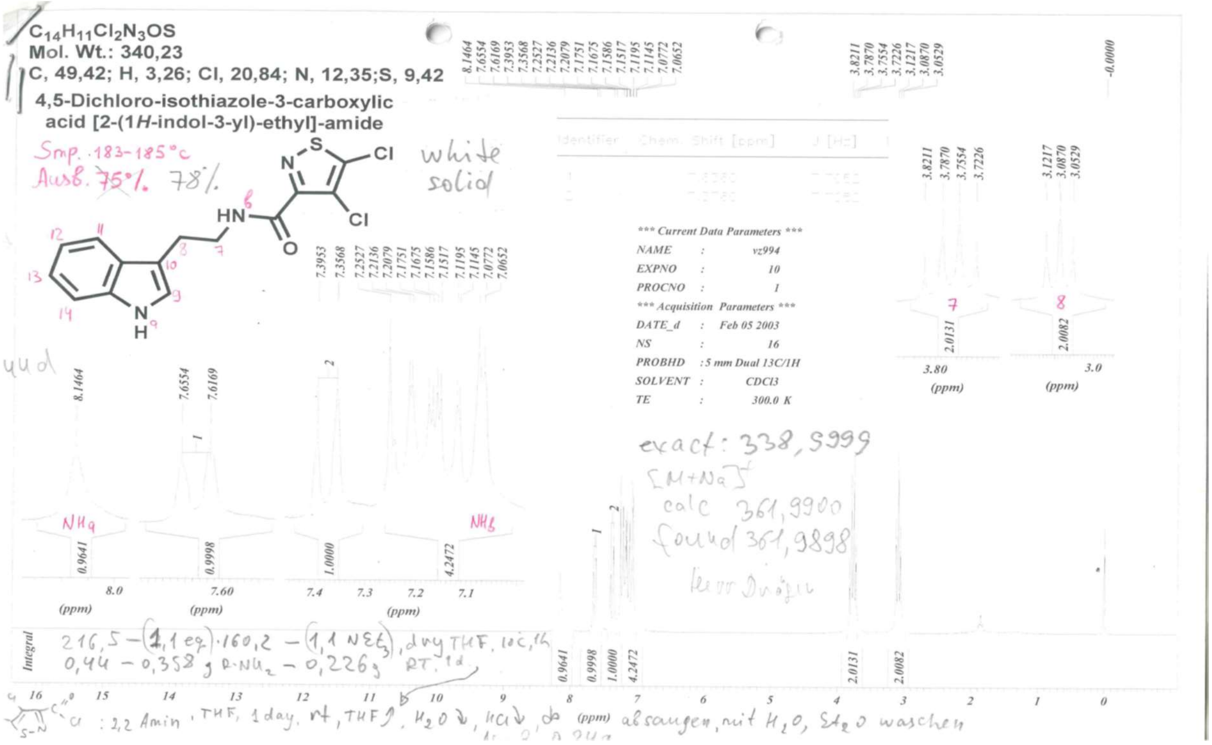

Figure S160. 50 MHz ^{13}C-NMR spectrum in $CDCl_3$ for **44d**.

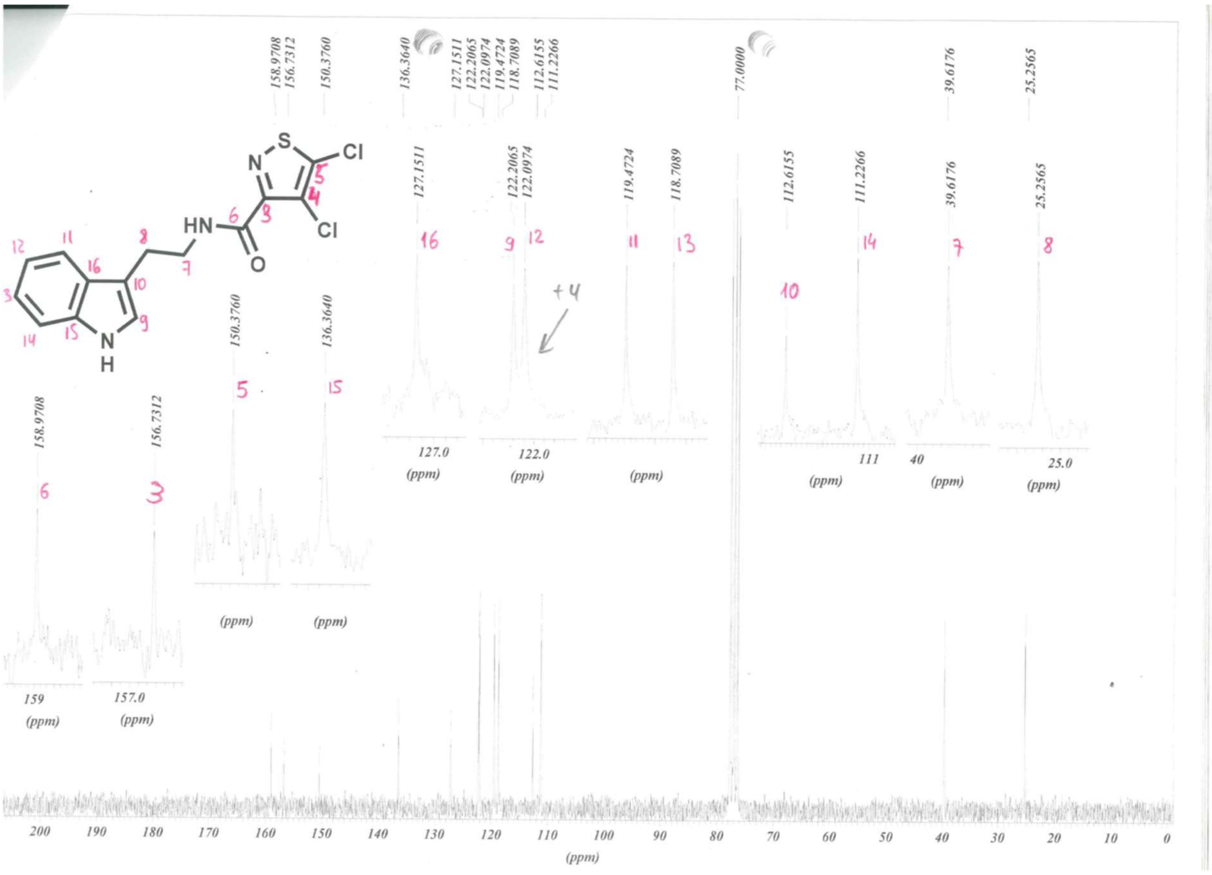

Figure S161. MS spectrum for 4,5-dichloro-*N*-[2-(1*H*-indol-3-yl)ethyl]-1,2-thiazole-3-carboxamide (**44d**).

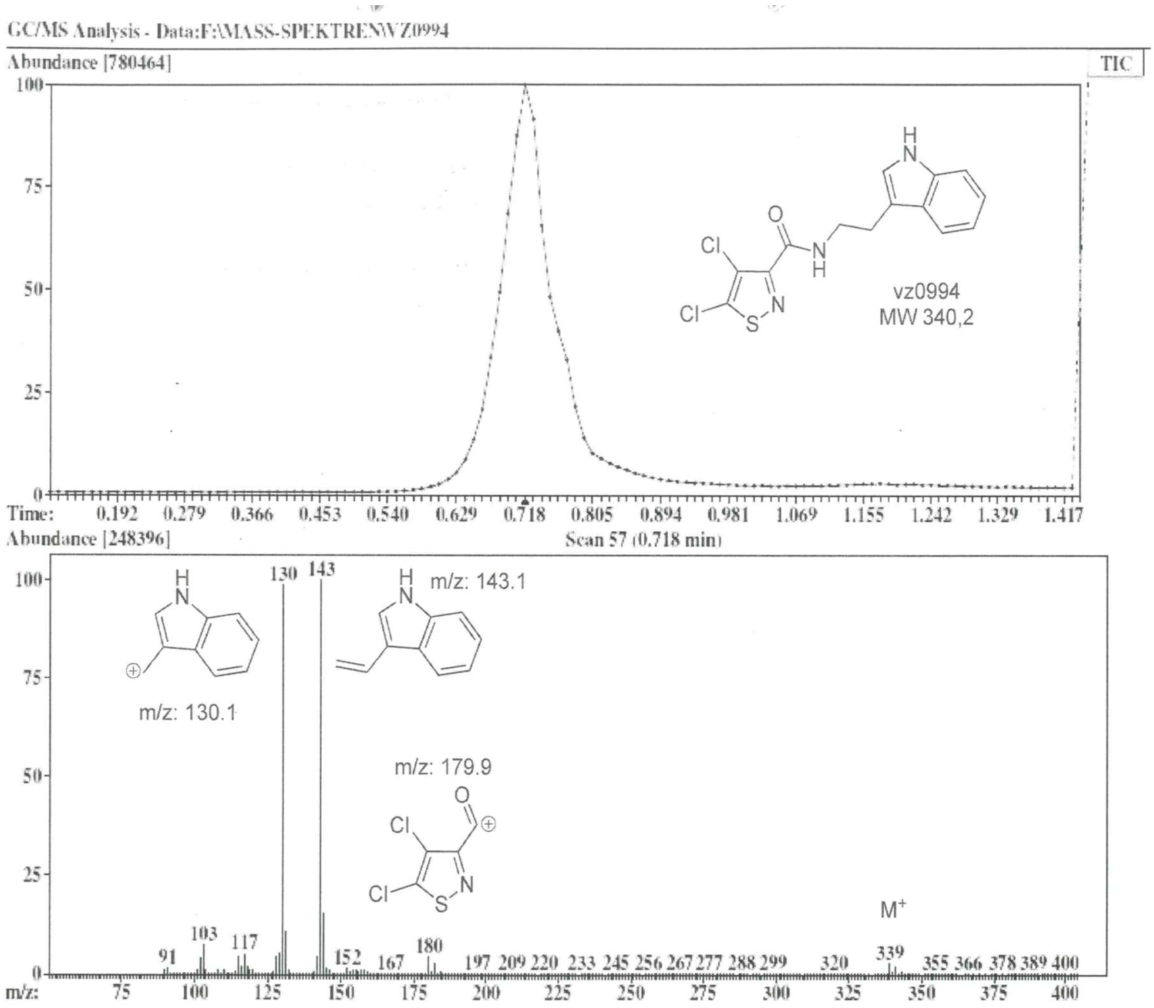

Figure S162. HR-MS spectrum for 4,5-dichloro-*N*-[2-(1*H*-indol-3-yl)ethyl]-1,2-thiazole-3-carboxamide (**44d**).

Elemental Composition Report

Single Mass Analysis (displaying only valid results)

Tolerance = 10.0 PPM / DBE: min = -1.5, max = 50.0
Selected filters: None

Monoisotopic Mass, Even Electron Ions
2476 formula(e) evaluated with 27 results within limits (up to 80 closest results for each mass)
Elements Used:
C: 0-50 H: 0-60 N: 0-3 O: 0-7 Na: 0-1 S: 0-2 Cl: 0-3

Zapolski LCT Premier KD070
VZ 0994 17 (0.388) AM (Cen,4, 95.00, Ar,11000.0,556.28,0.70,LS 5) 1: TOF MS ES+ 893

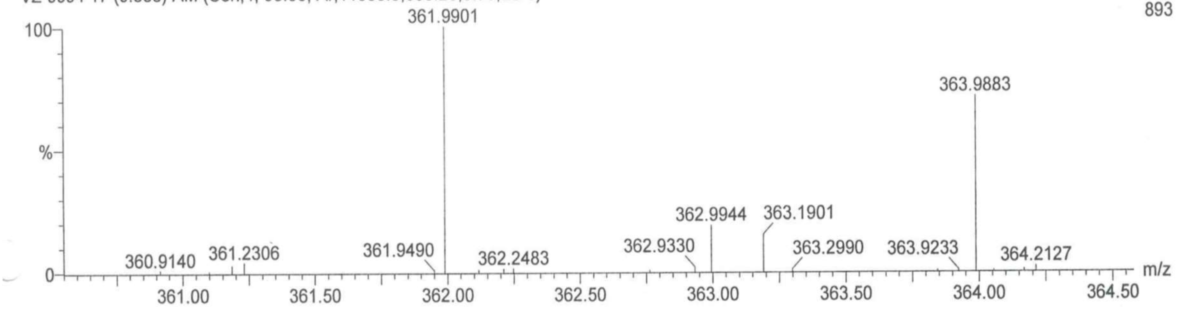

Mass	Calc. Mass	mDa	PPM	DBE	i-FIT	Formula
Minimum:				-1.5		
Maximum:		5.0	10.0	50.0		
361.9901	361.9900	0.1	0.3	4.5	40.3	C11 H14 N O5 Na S2 Cl
	361.9900	0.1	0.3	3.5	38.9	C10 H15 N3 O3 S Cl3
	361.9902	-0.1	-0.3	-1.5	18.4	C7 H18 N O7 S2 Cl2
	361.9898	0.3	0.8	9.5	0.3	C14 H11 N3 O Na S Cl2
	361.9905	-0.4	-1.1	12.5	218.1	C14 H8 N3 O5 S2
	361.9906	-0.5	-1.4	12.5	20.6	C18 H11 N O Cl3
	361.9890	1.1	3.0	12.5	54.0	C16 H9 N O5 S Cl
	361.9912	-1.1	-3.0	21.5	258.3	C22 H4 N O3 S
	361.9888	1.3	3.6	17.5	4.2	C19 H6 N3 O Cl2
	361.9888	1.3	3.6	18.5	256.5	C20 H5 N O3 Na S
	361.9916	-1.5	-4.1	4.5	32.2	C13 H16 N O Na S Cl3
	361.9883	1.8	5.0	3.5	45.1	C8 H13 N3 O7 S2 Cl
	361.9882	1.9	5.2	9.5	22.2	C16 H12 N O Na Cl3
	361.9881	2.0	5.5	9.5	221.0	C12 H9 N3 O5 Na S2
	361.9922	-2.1	-5.8	13.5	220.6	C17 H9 N O3 Na S2
	361.9922	-2.1	-5.8	12.5	0.3	C16 H10 N3 O S Cl2
	361.9923	-2.2	-6.1	5.5	8.7	C10 H11 N3 O6 Na Cl2
	361.9924	-2.3	-6.4	7.5	38.2	C13 H13 N O5 S2 Cl
	361.9878	2.3	6.4	26.5	296.1	C25 N O3
	361.9876	2.5	6.9	0.5	46.3	C8 H16 N3 O3 Na S Cl3
	361.9872	2.9	8.0	17.5	253.0	C17 H4 N3 O5 S
	361.9931	-3.0	-8.3	4.5	4.1	C11 H15 N3 O Na S2 Cl2
	361.9933	-3.2	-8.8	-1.5	54.4	C7 H19 N3 O3 S2 Cl3
	361.9868	3.3	9.1	3.5	8.8	C10 H14 N O7 S Cl2
	361.9866	3.5	9.7	8.5	26.8	C13 H11 N3 O3 Cl3
	361.9866	3.5	9.7	9.5	53.9	C14 H10 N O5 Na S Cl
	361.9865	3.6	9.9	16.5	51.4	C20 H9 N S2 Cl

Figure S163. 600 MHz ^{1}H-NMR spectrum in $CDCl_3$ for **44e**.

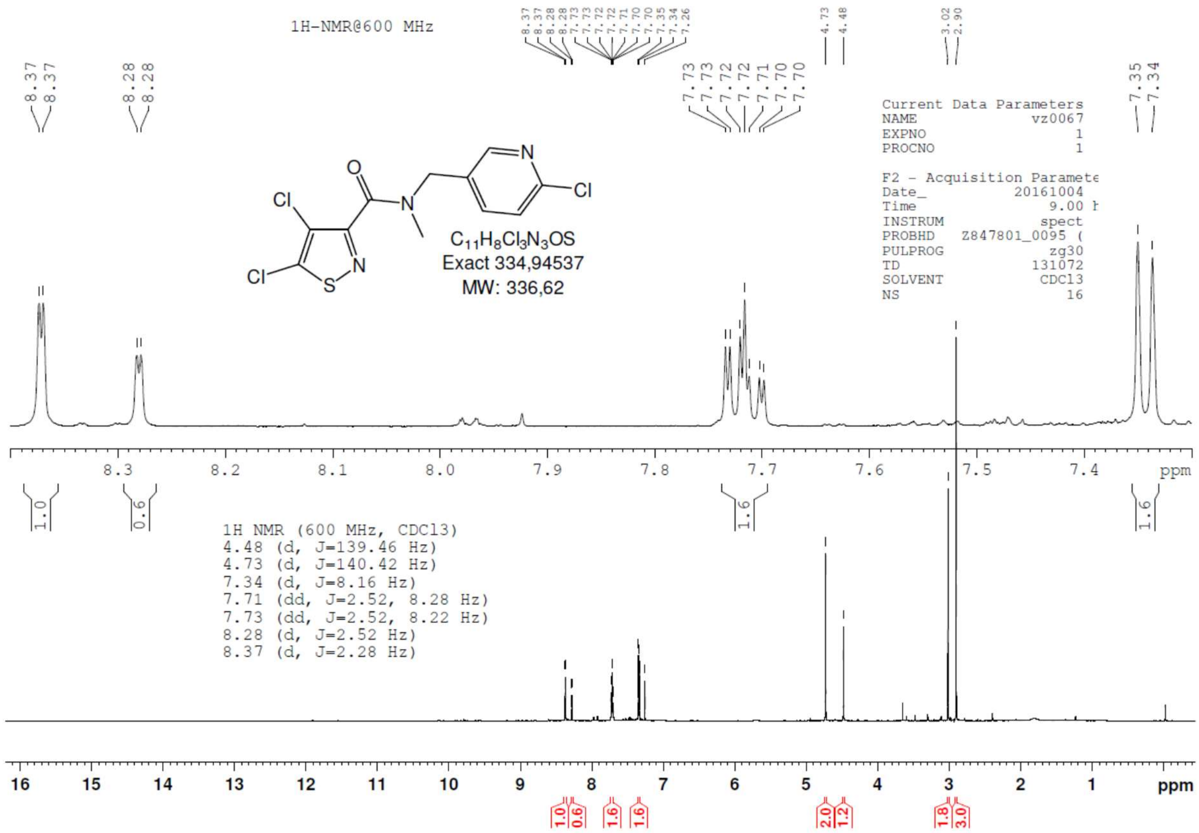

Figure S164. 150 MHz ^{13}C-NMR spectrum in $CDCl_3$ for **44e**.

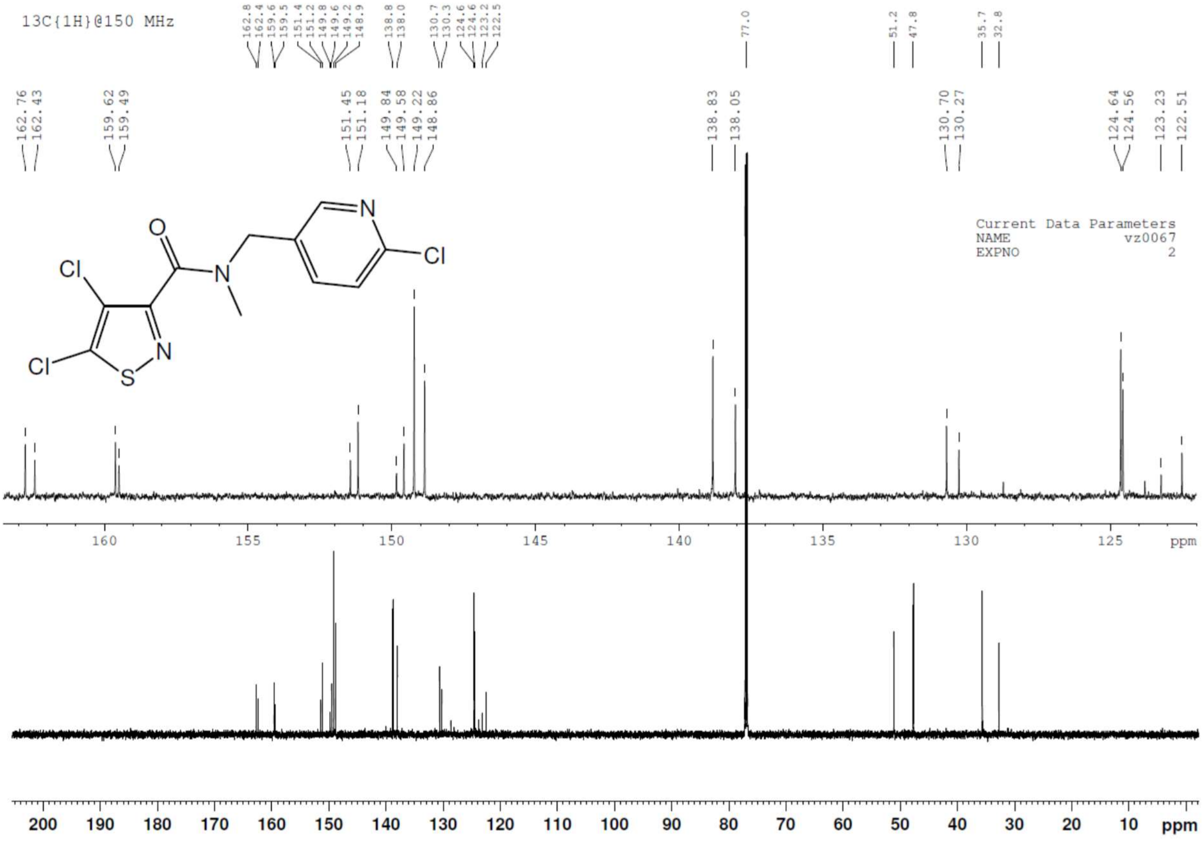

Figure S165. 400 MHz ^{1}H-NMR spectrum in $CDCl_3$ for **45**.

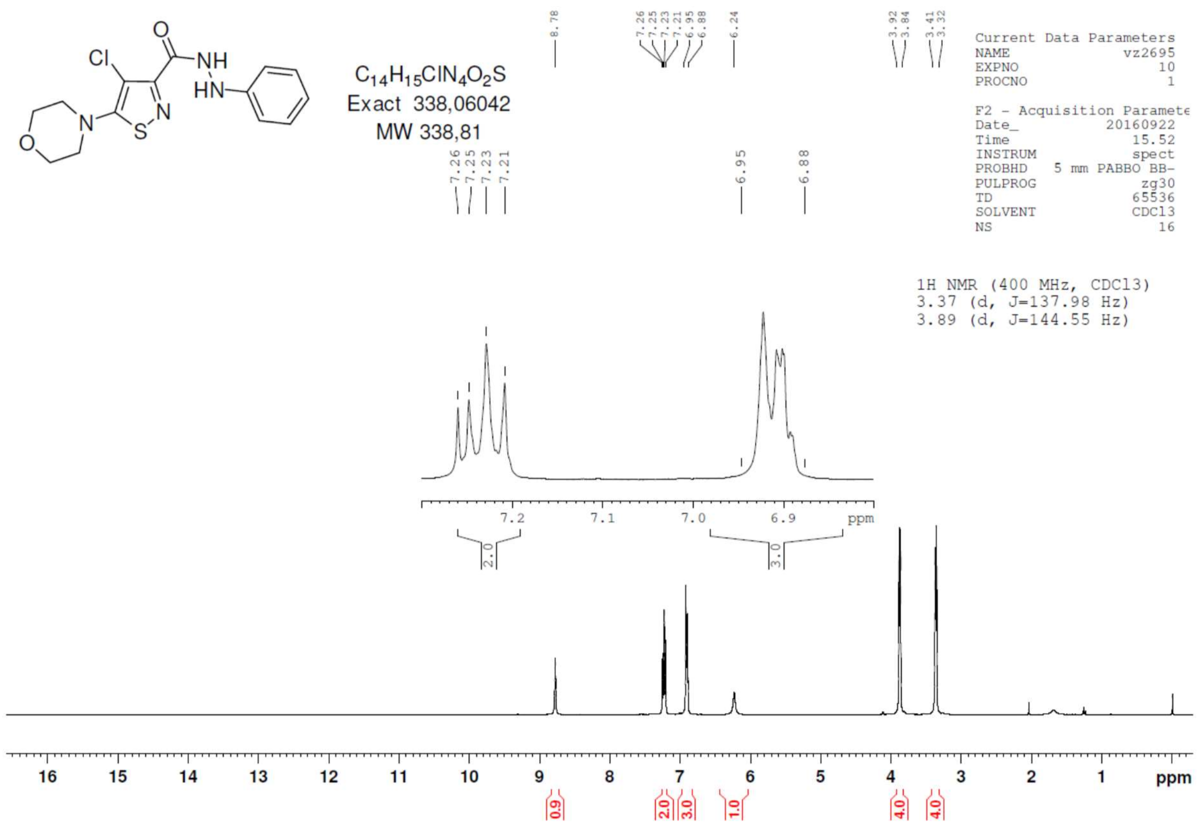

Figure S166. 100 MHz ^{13}C-NMR spectrum in $CDCl_3$ for **45**.

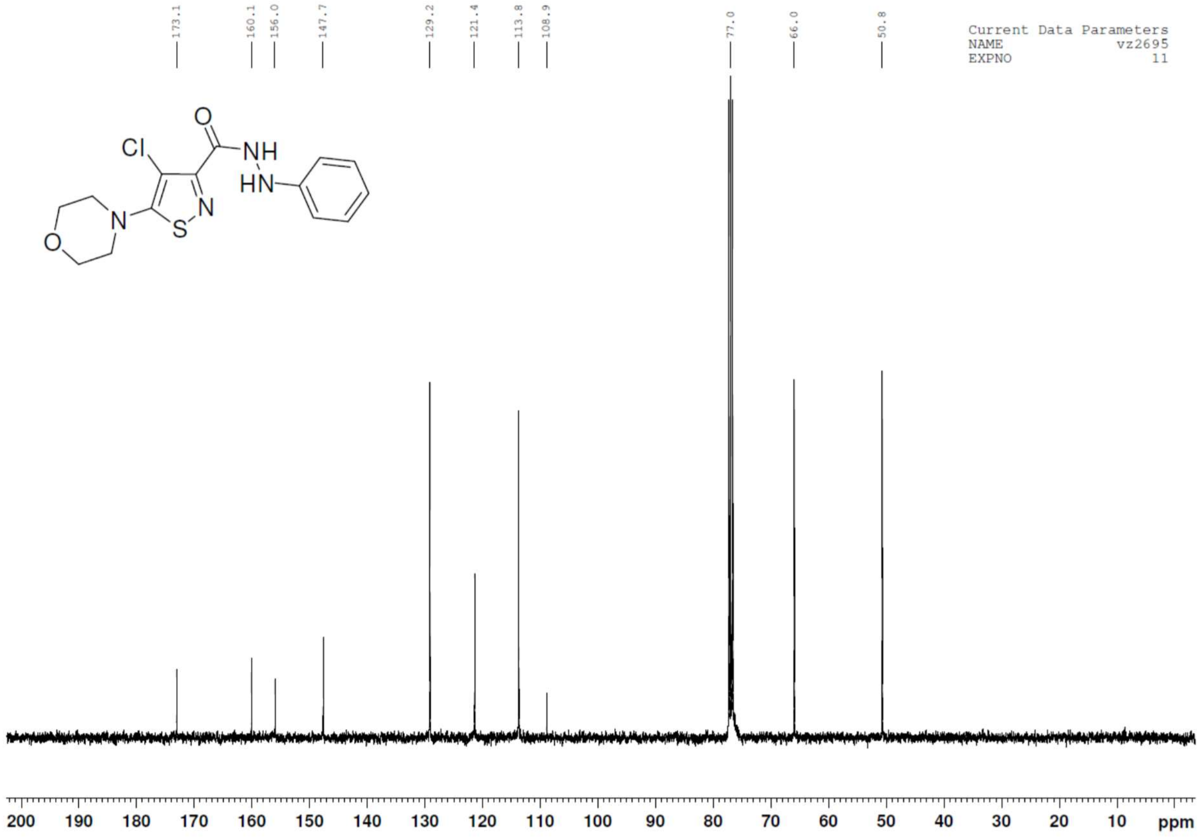

Figure S167. 400 MHz ^{1}H-NMR spectrum in $CDCl_3$ for **46a**.

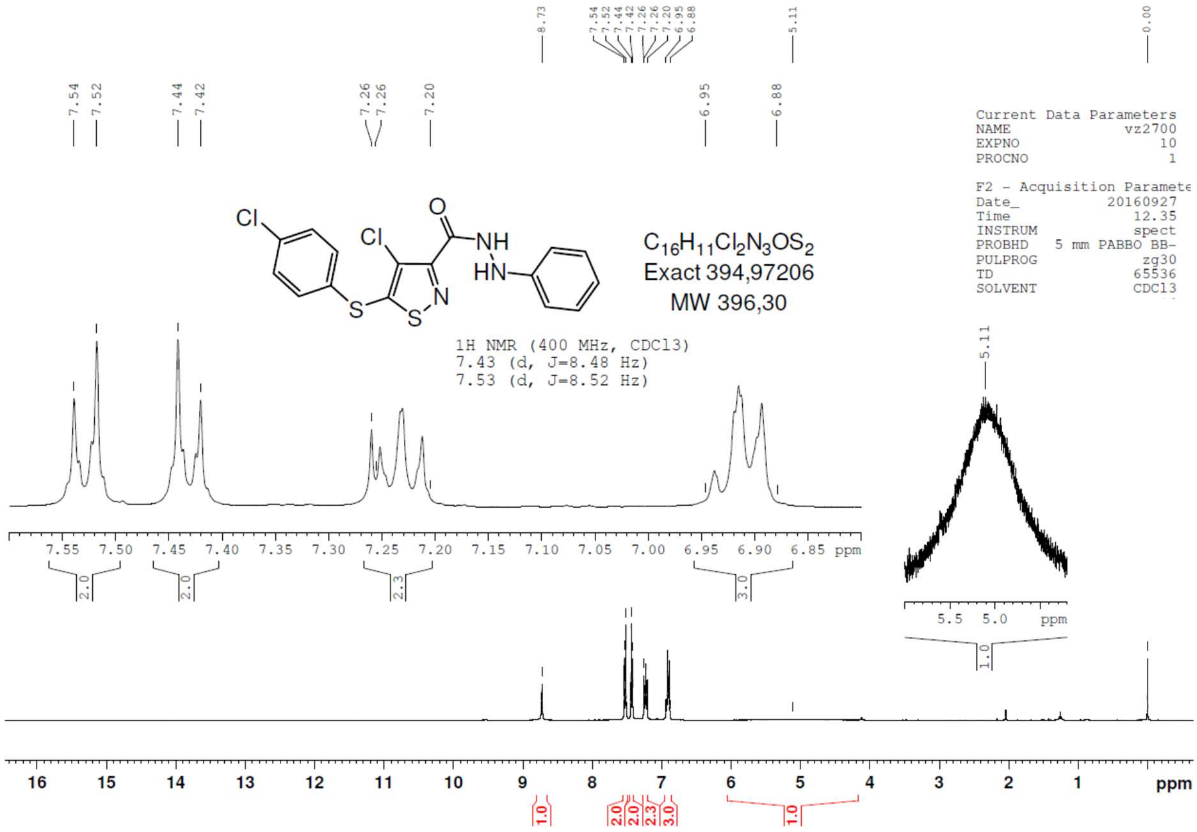

Figure S168. 100 MHz ^{13}C-NMR spectrum in $CDCl_3$ for **46a**.

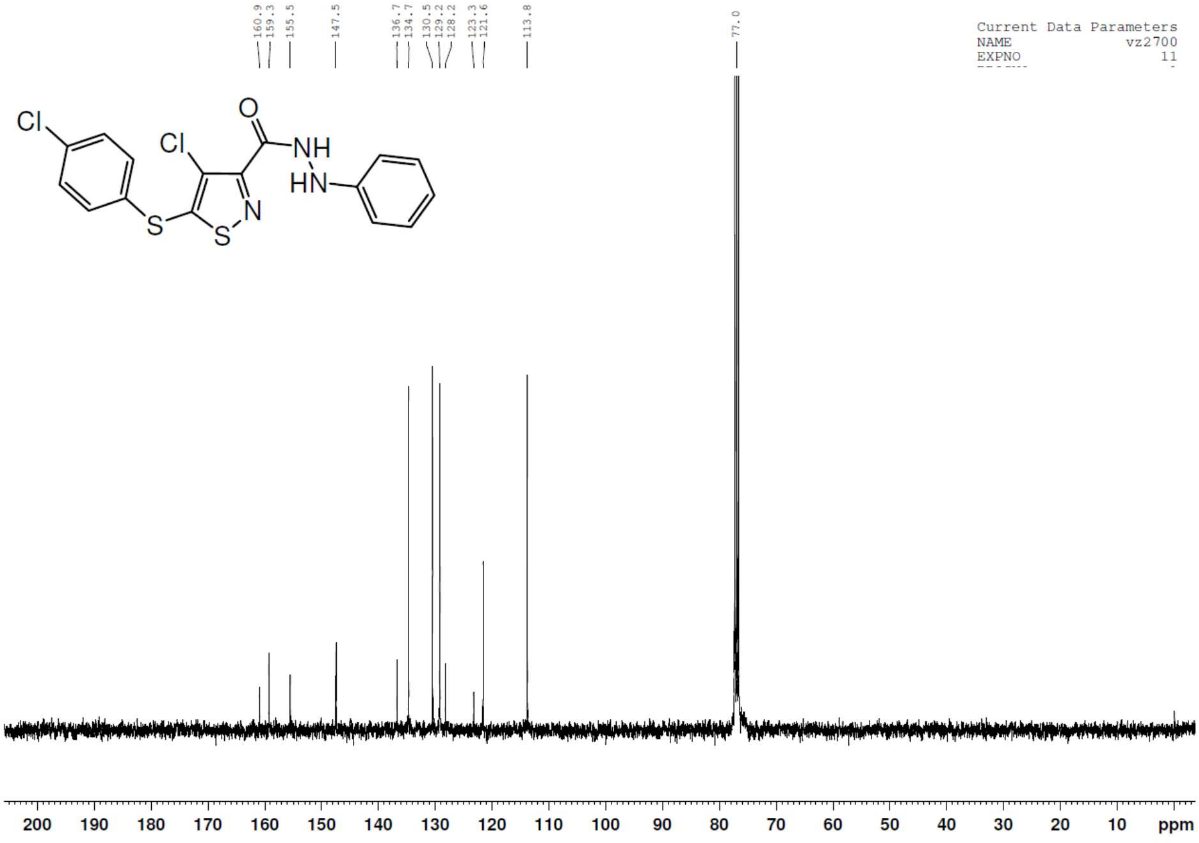

Figure S169. 400 MHz ^{1}H-NMR spectrum in $CDCl_3$ for **46b**.

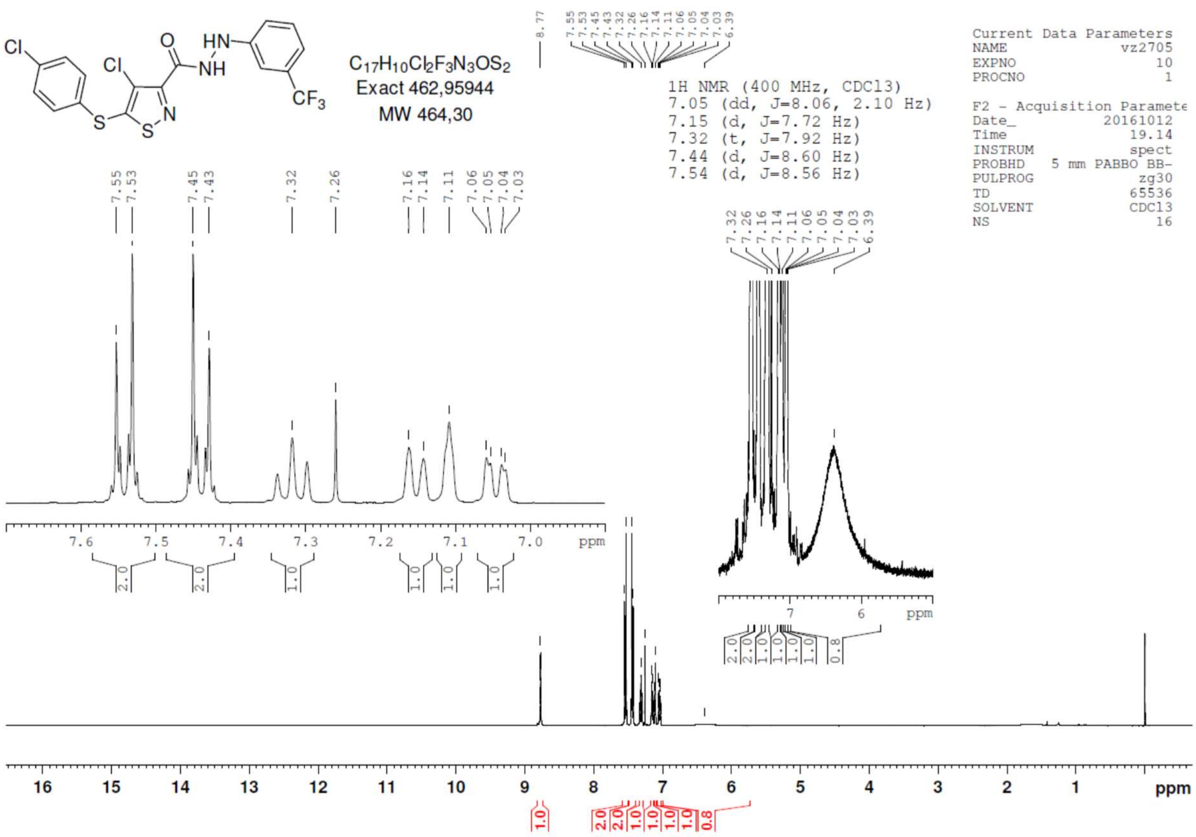

Figure S170. 100 MHz ^{13}C-NMR spectrum in $CDCl_3$ for **46b**.

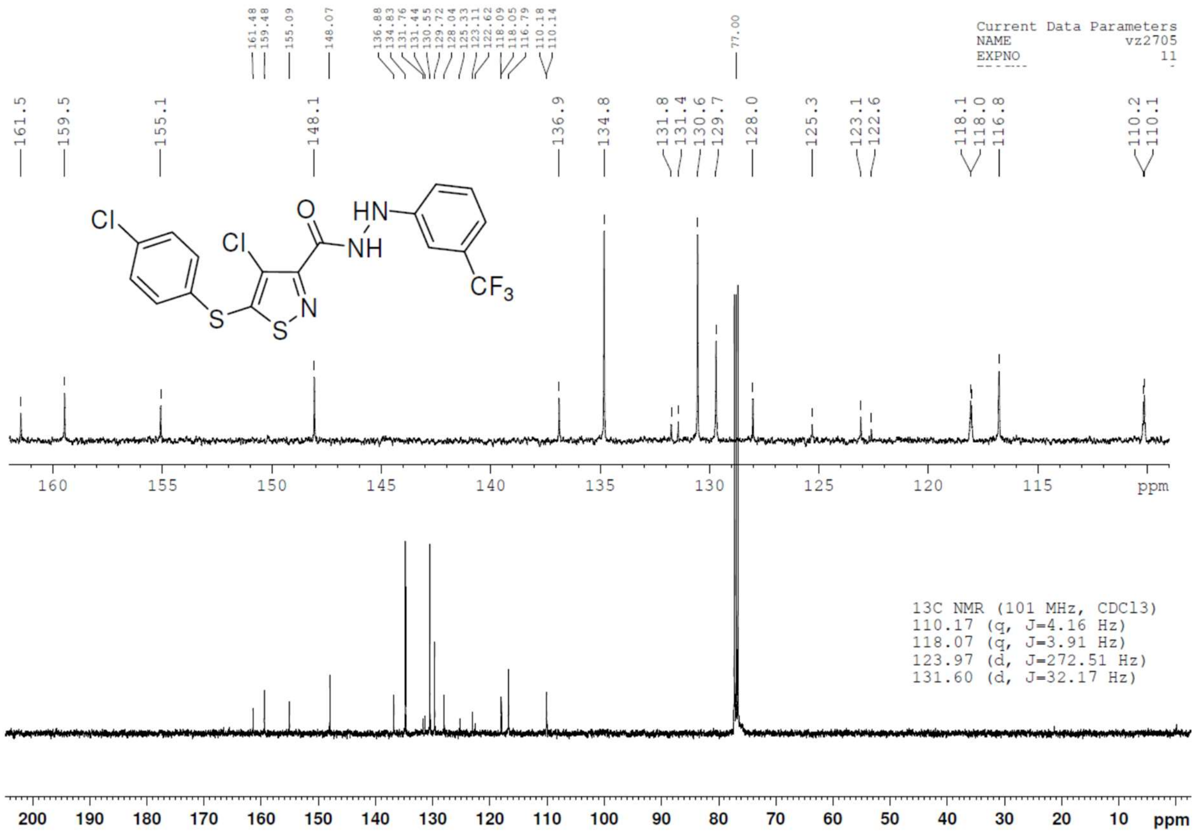

Figure S171. 400 MHz ^{1}H-NMR spectrum in $CDCl_3$ for **46c**.

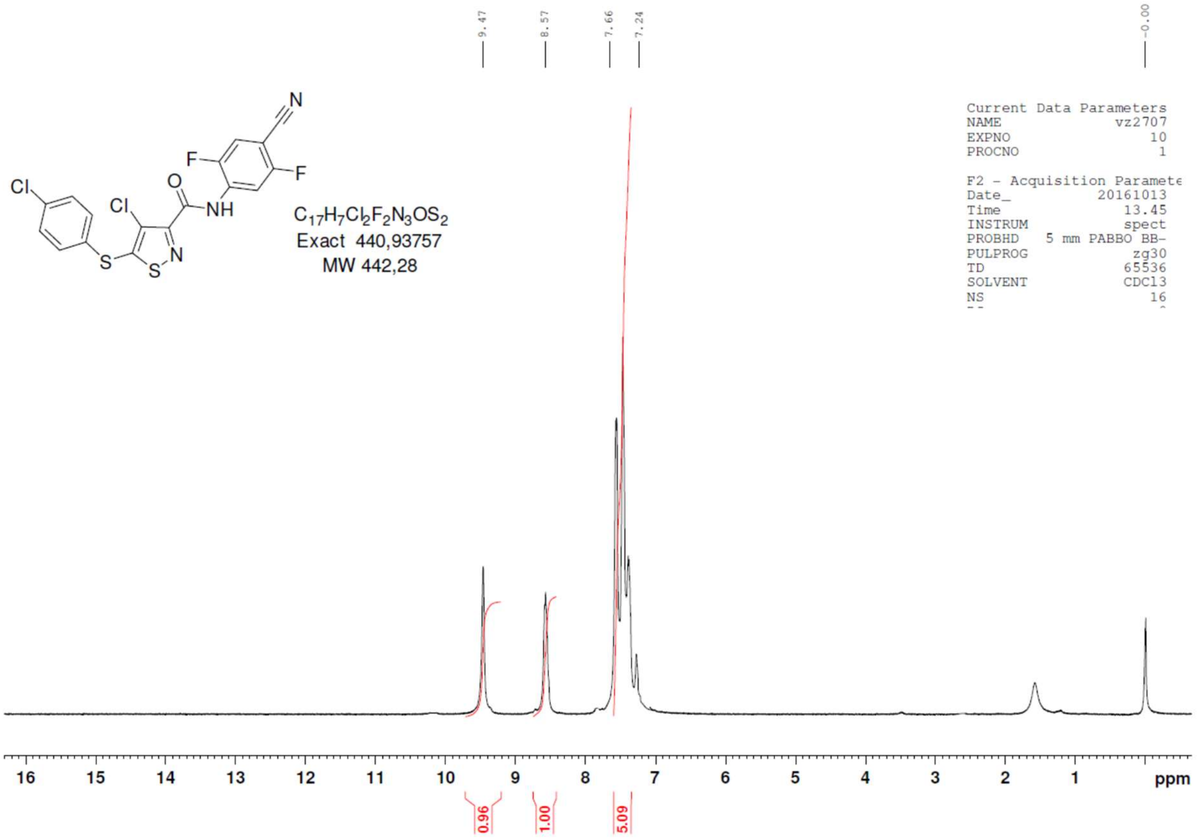

Figure S172. 100 MHz ^{13}C-NMR spectrum in $CDCl_3$ for **46c**.

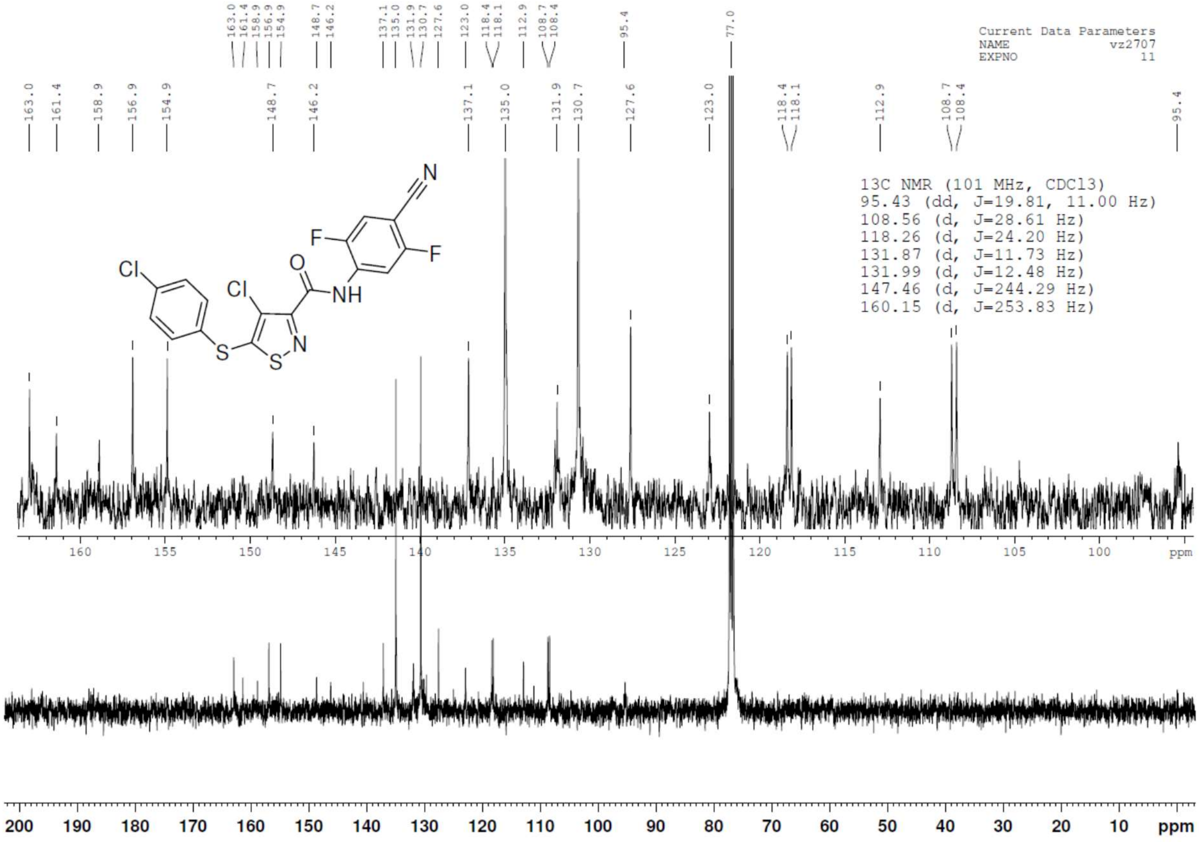

Figure S173. 400 MHz ¹H-NMR spectrum in DMSO-*d6* for **46d**.

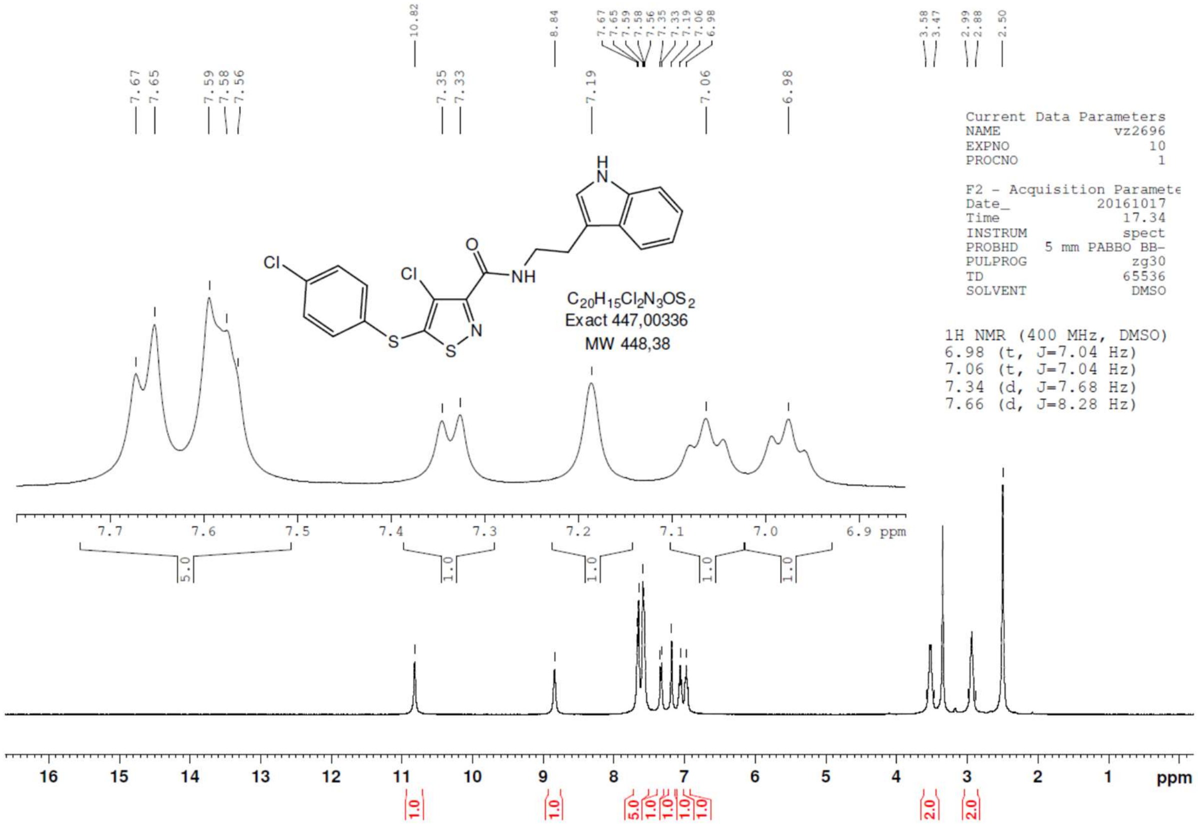

Figure S174. 100 MHz ¹³C-NMR spectrum in DMSO-*d6* for **46d**.

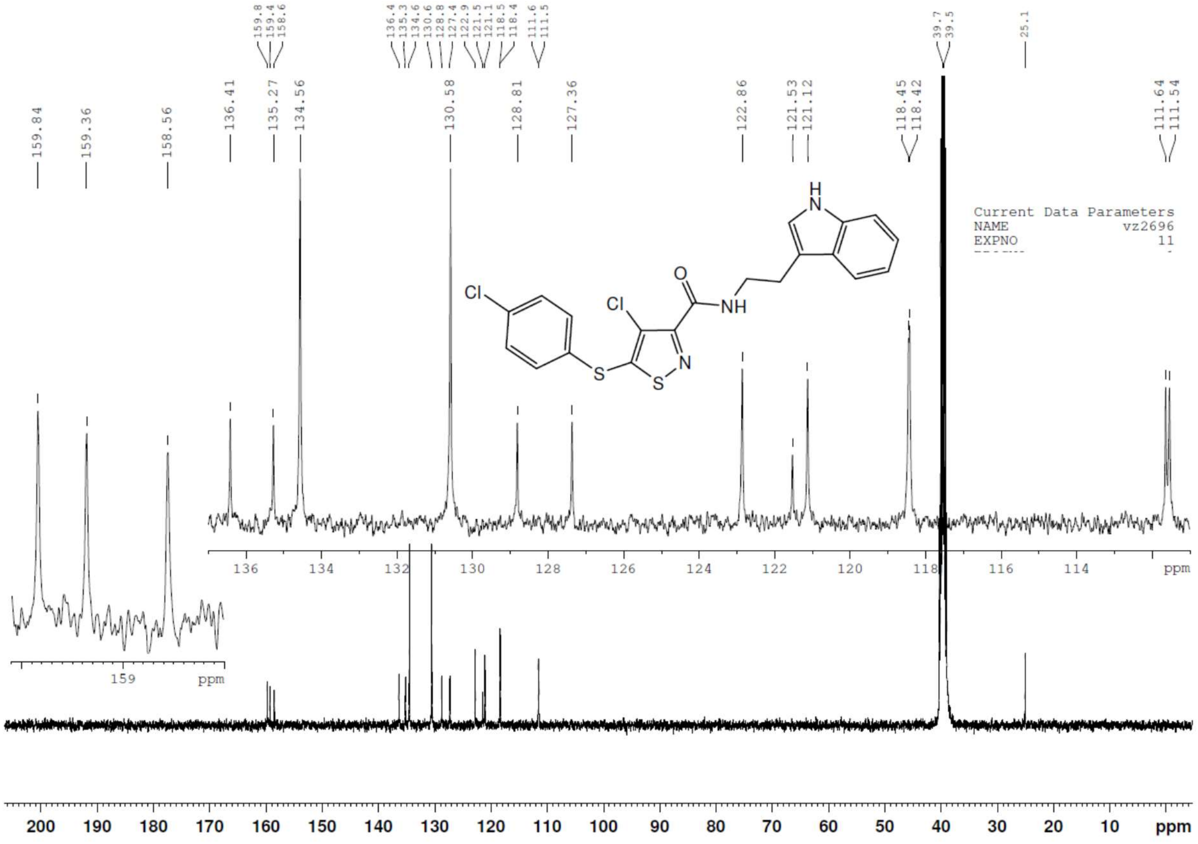

Figure S175. 400 MHz ^{1}H-NMR spectrum in $CDCl_3$ for **46e**.

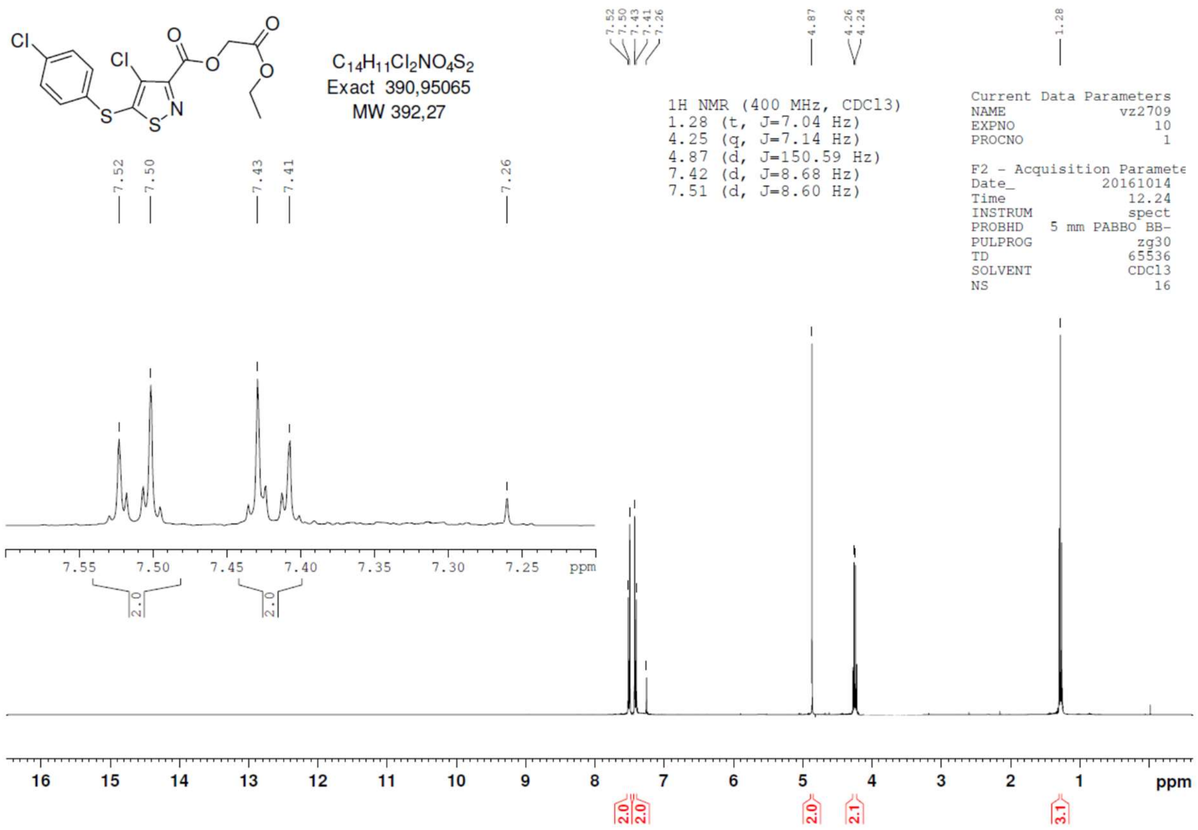

Figure S176. 100 MHz ^{13}C-NMR spectrum in $CDCl_3$ for **46e**.

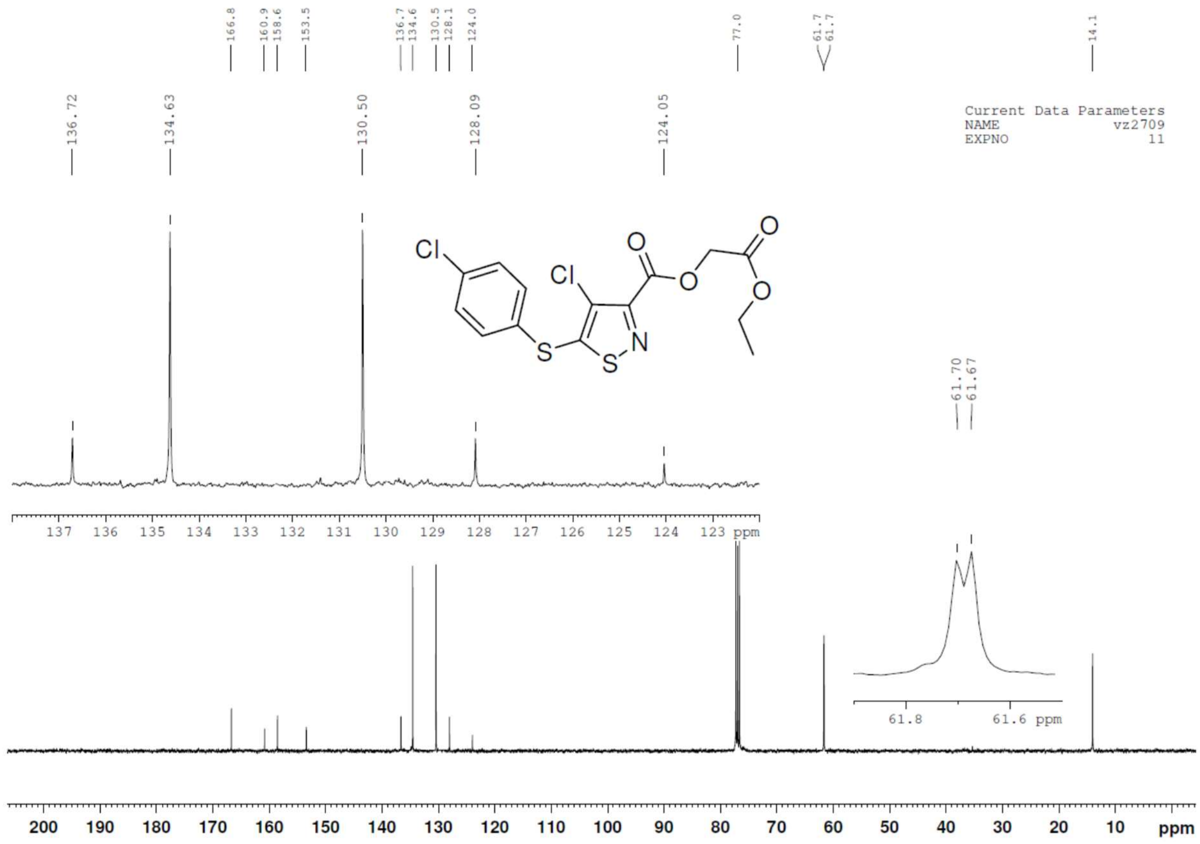

Figure S177. 400 MHz ¹H-NMR spectrum in $CDCl_3$ for **47**.

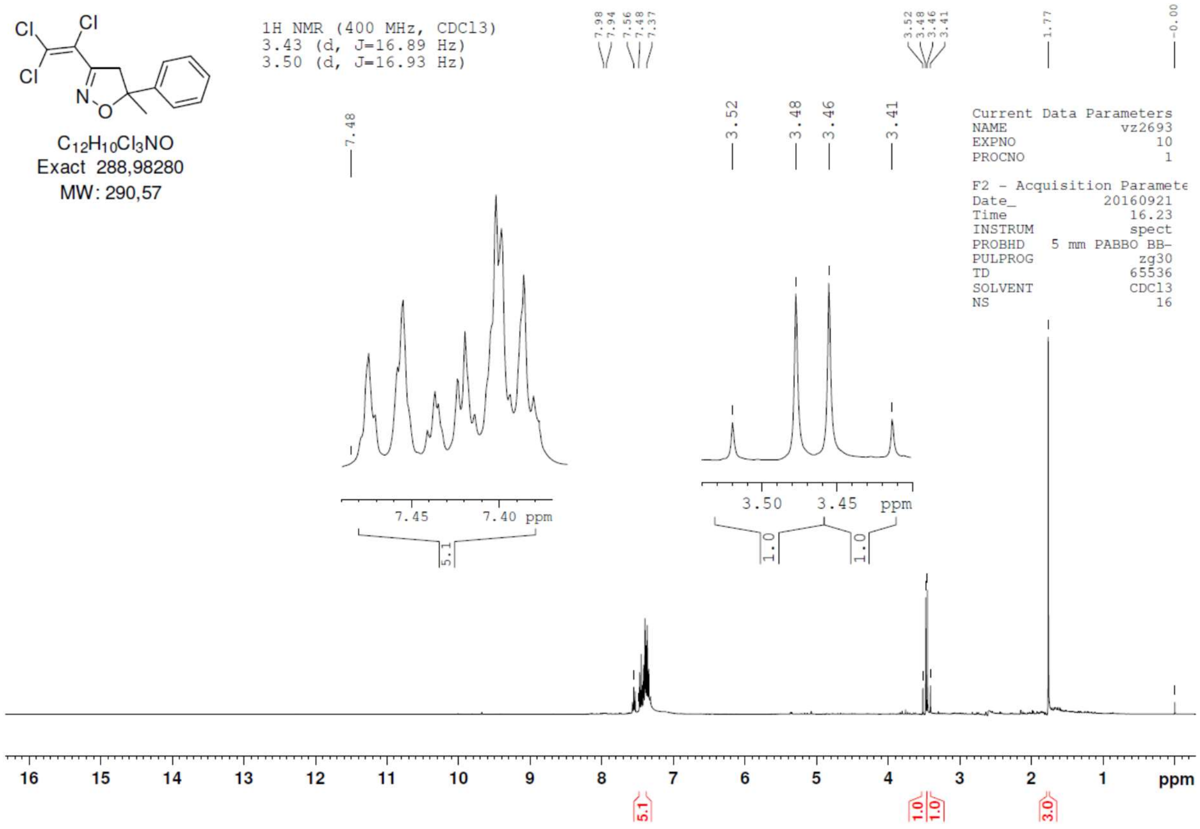

Figure S178. 100 MHz ¹³C-NMR spectrum in $CDCl_3$ for **47**.

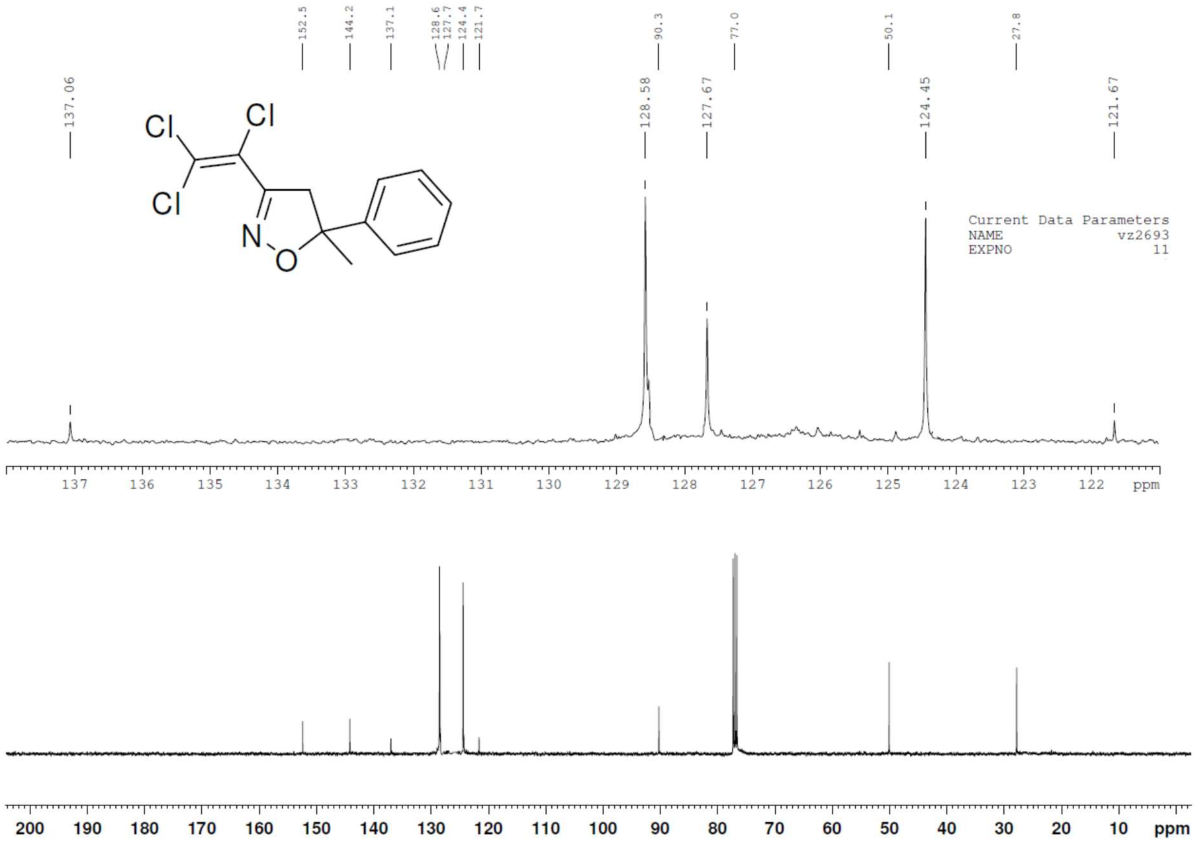

Figure S179. 400 MHz ¹H-NMR spectrum in CDCl₃ for **48**.

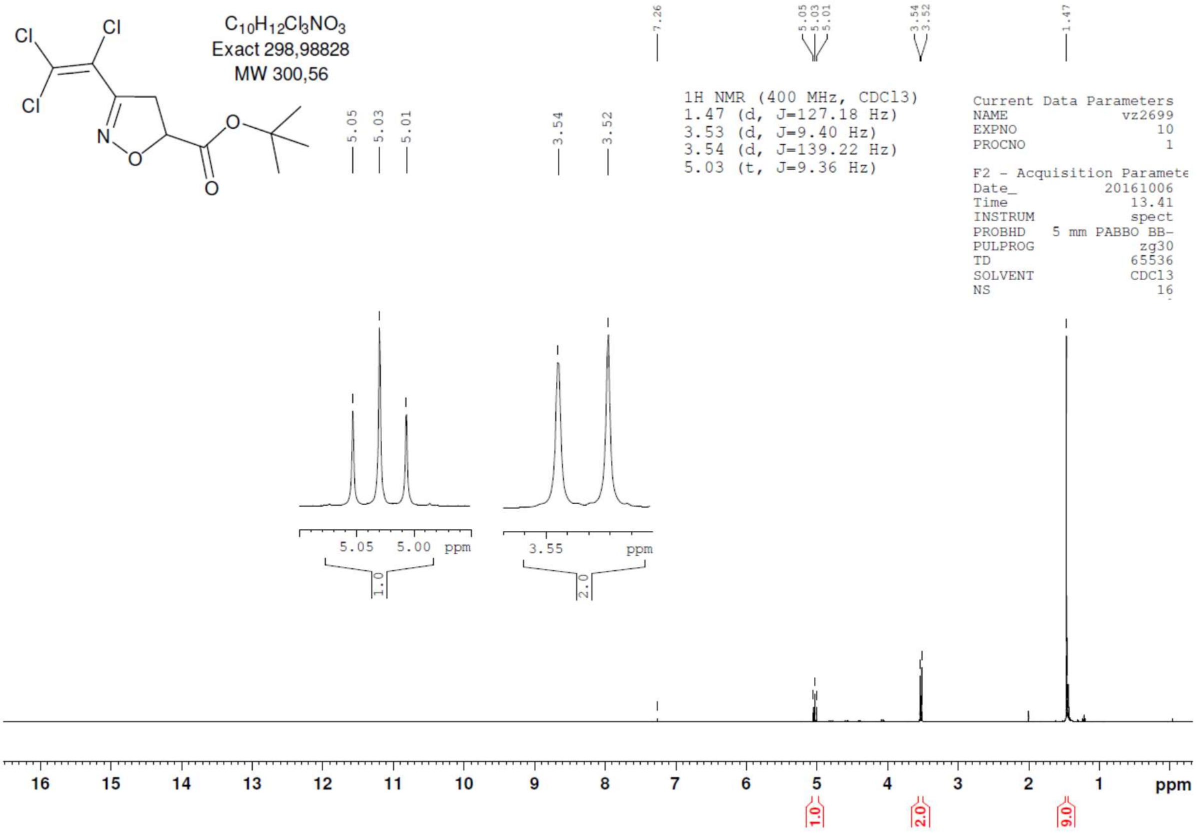

Figure S180. 100 MHz ¹³C-NMR spectrum in CDCl₃ for **48**.

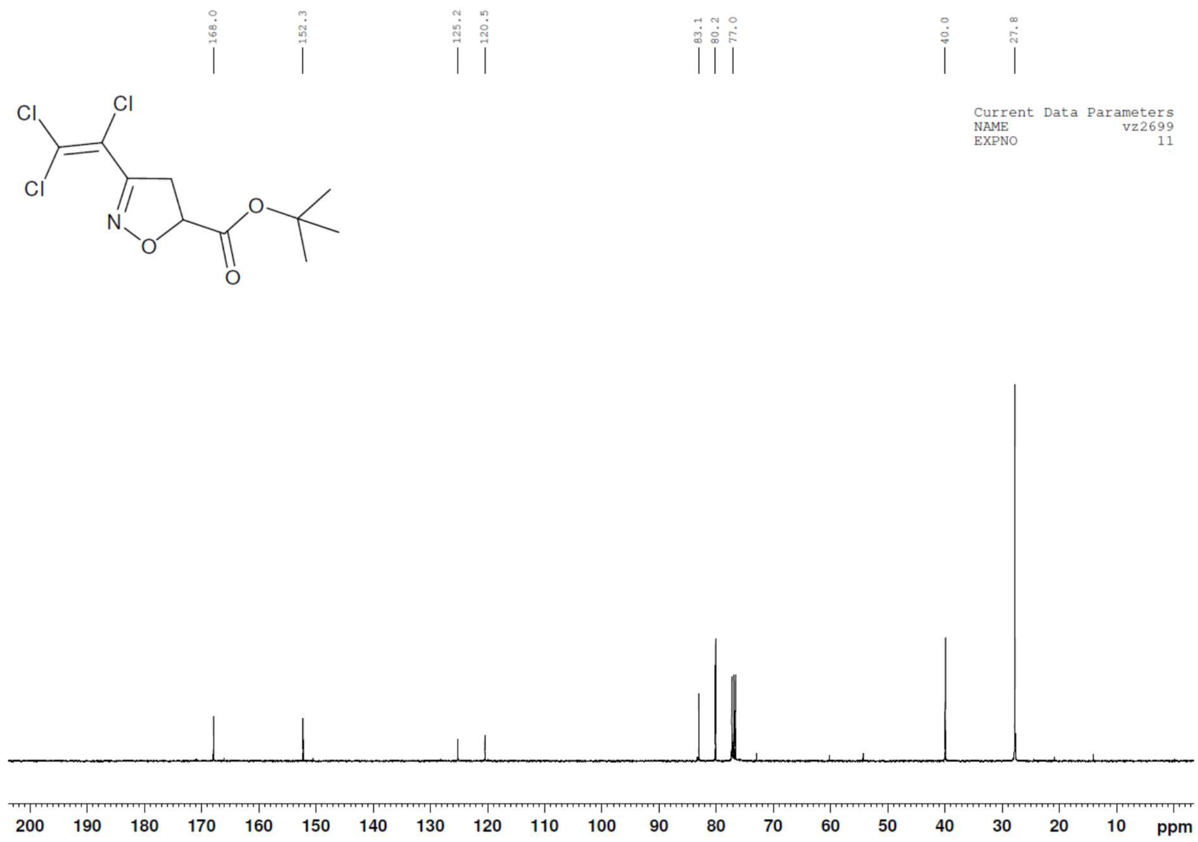

Figure S181. 600 MHz ^{15}N, ^{1}H-HMBC spectrum in $CDCl_3$ for **48**.

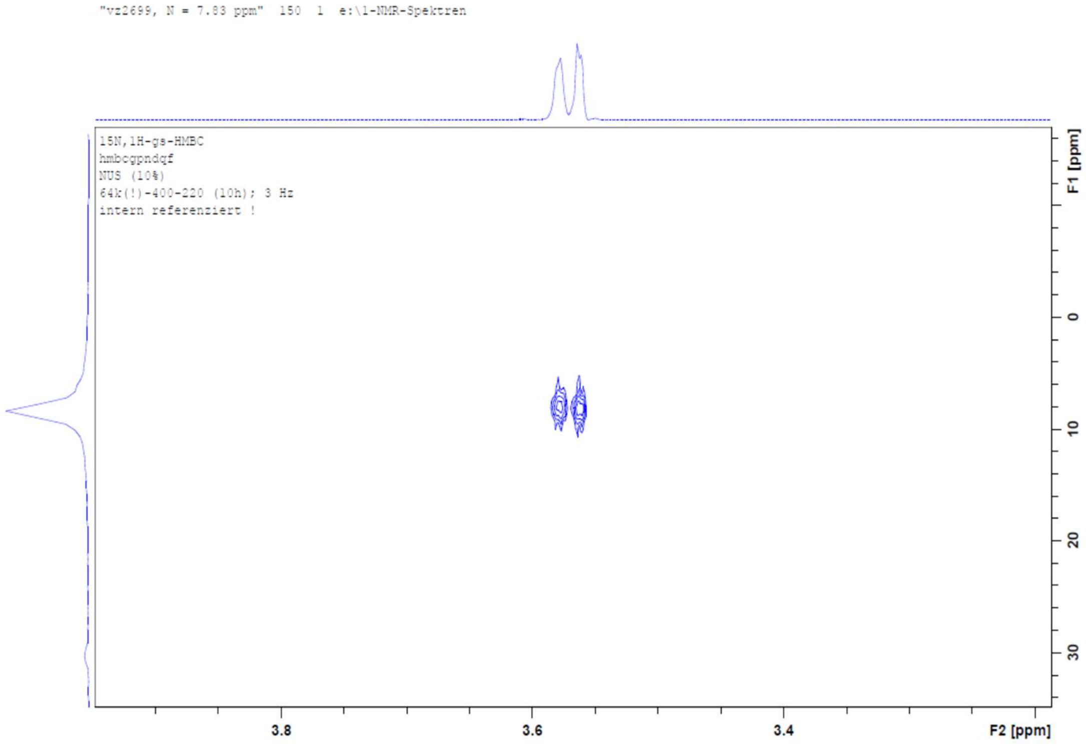

Figure S182. 600 MHz HMBC spectrum in $CDCl_3$ for **48**.

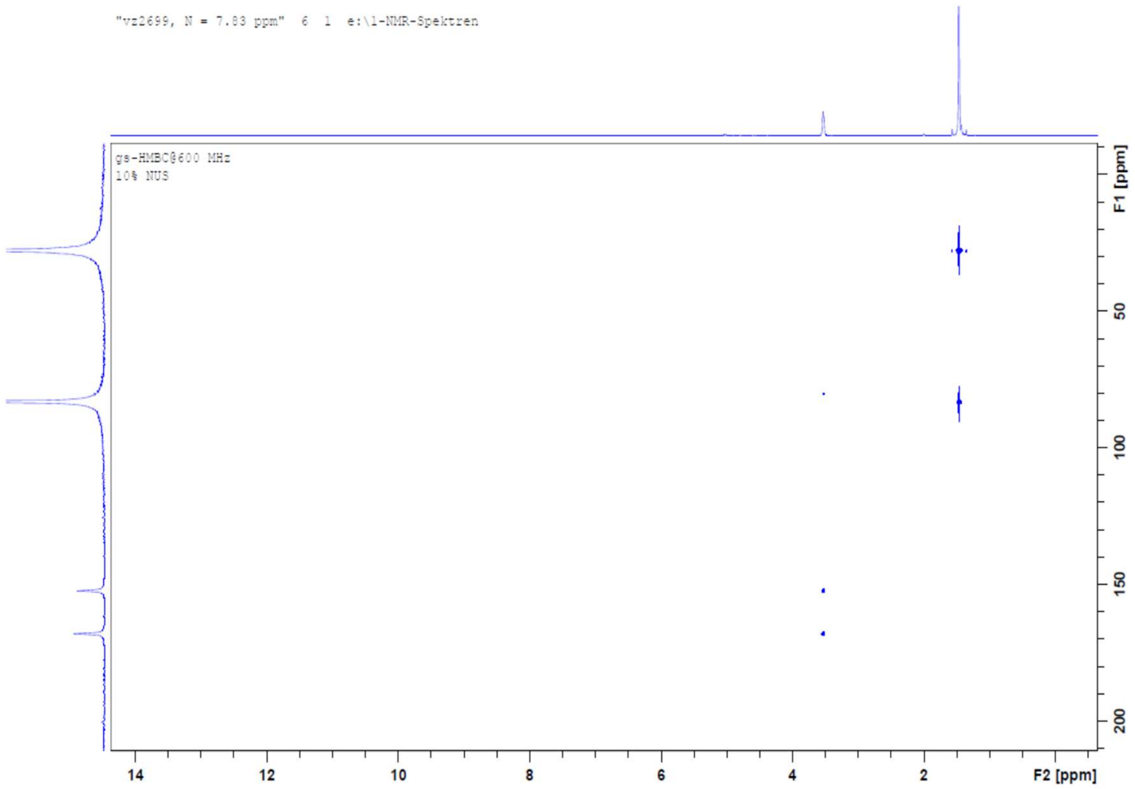

Figure S183. 400 MHz ^{1}H-NMR spectrum in $CDCl_3$ for **49**.

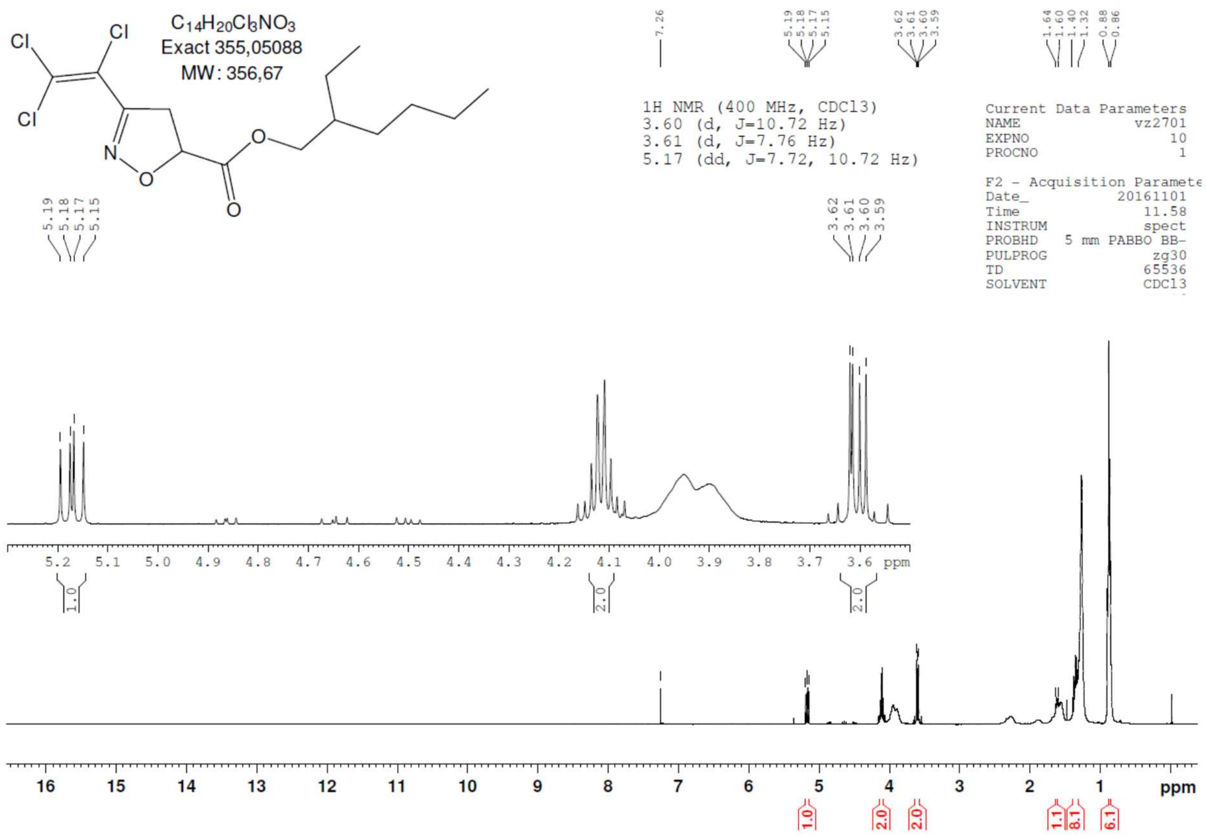

Figure S184. 100 MHz ^{13}C-NMR spectrum in $CDCl_3$ for **49**.

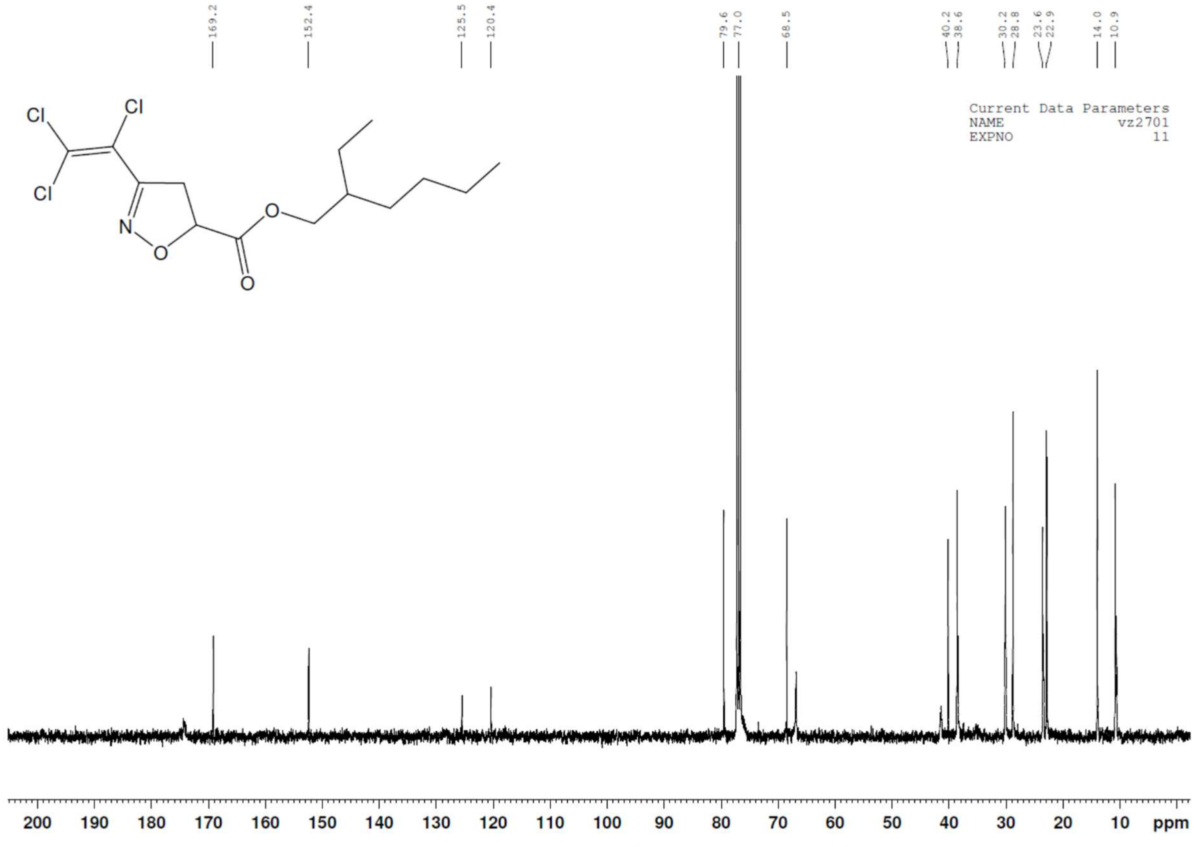

Figure S185. 400 MHz ^{1}H-NMR spectrum in $CDCl_3$ for **50**.

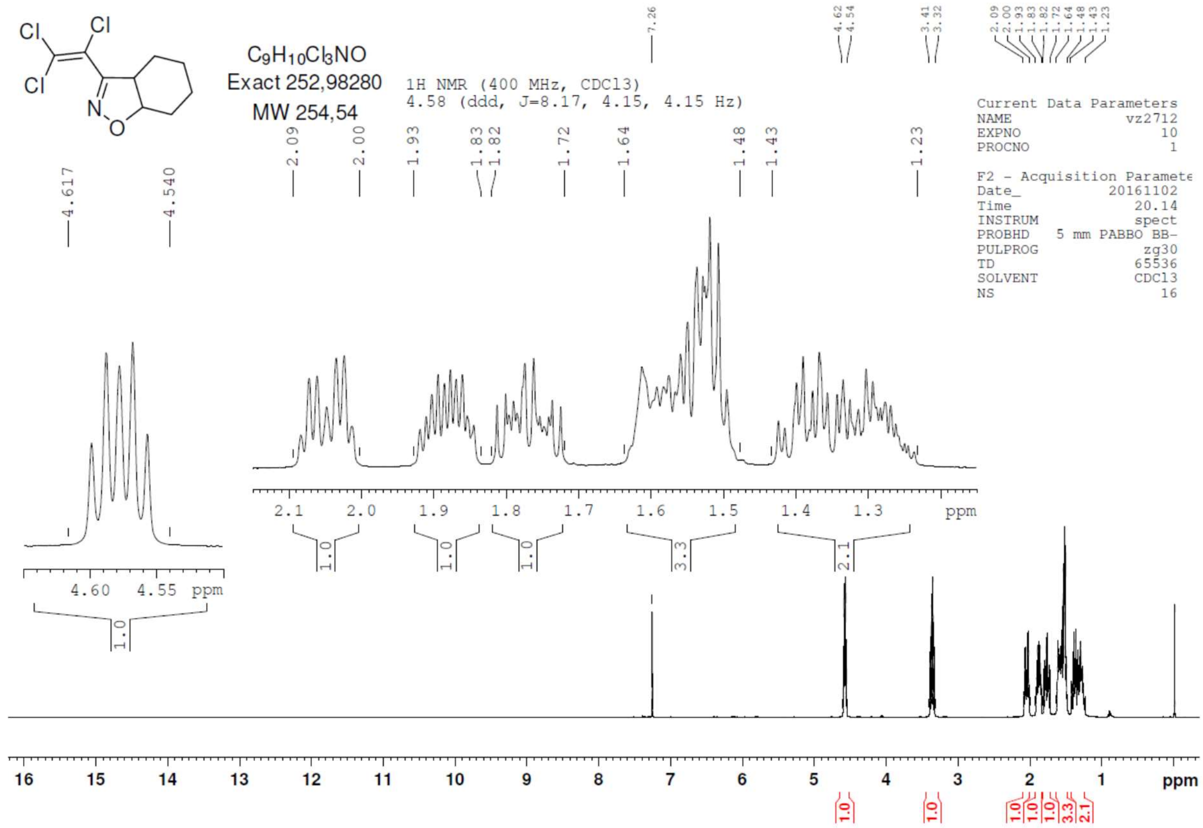

Figure S186. 100 MHz ^{13}C-NMR spectrum in $CDCl_3$ for **50**.

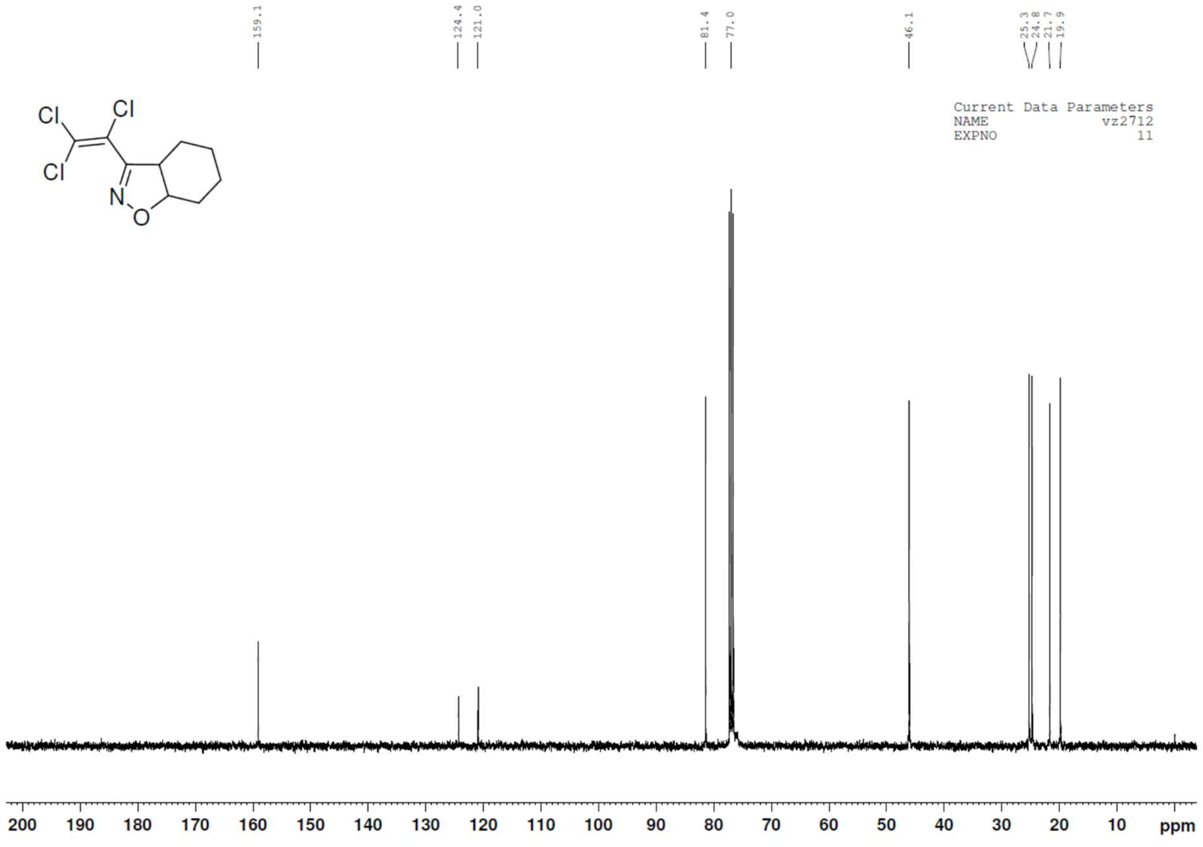

Figure S187. 200 MHz ^{1}H-NMR spectrum in $CDCl_3$ for **52**.

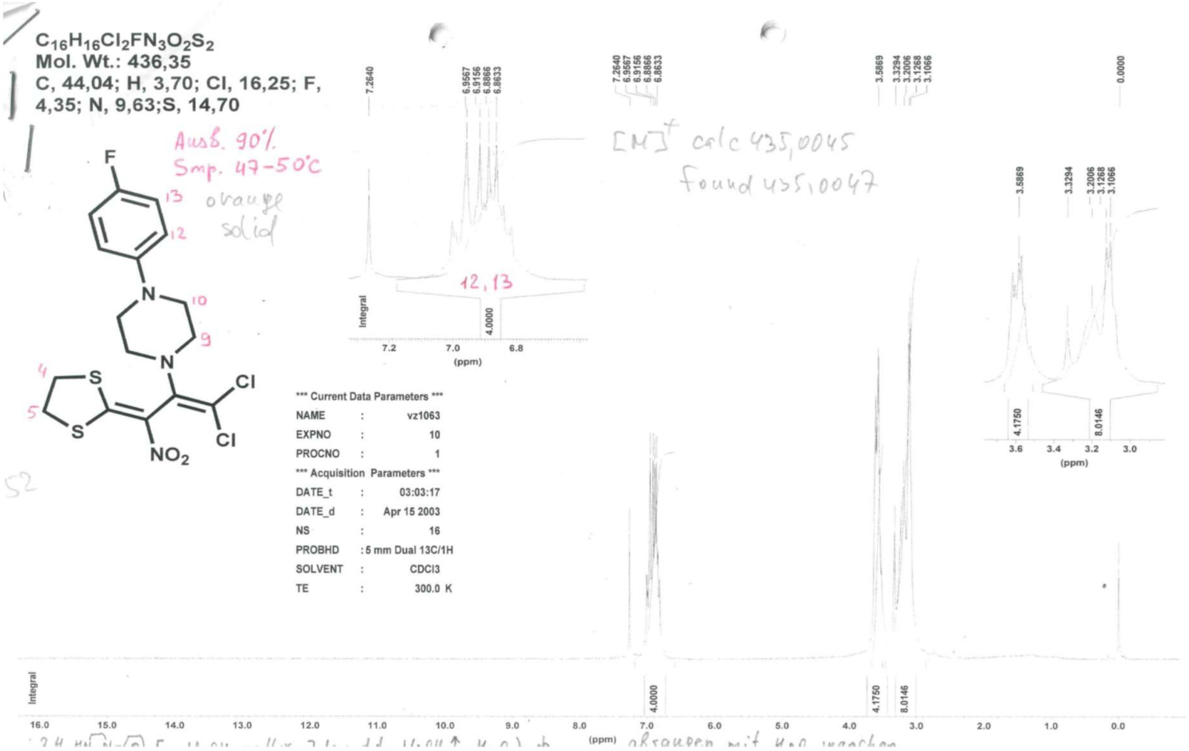

Figure S188. 50 MHz ^{13}C-NMR spectrum in $CDCl_3$ for **52**.

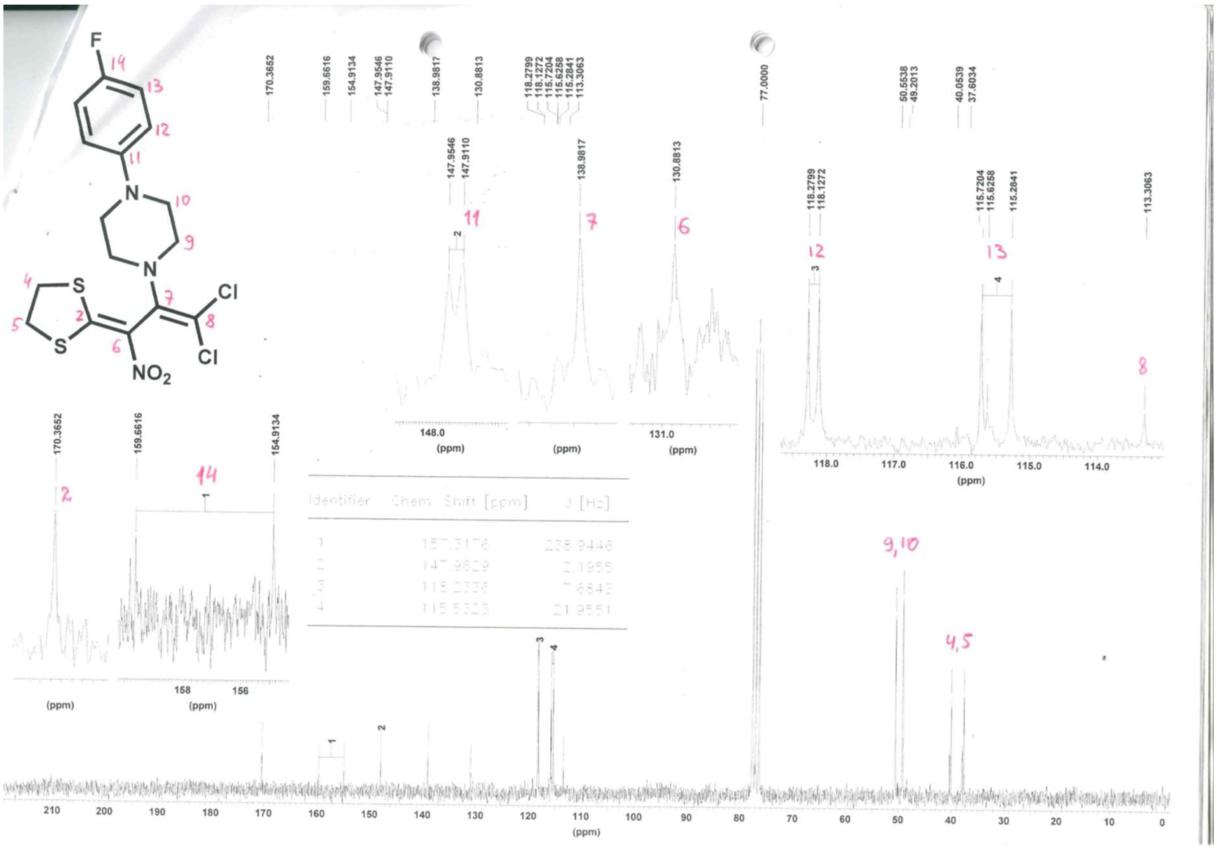

Figure S189. 100 MHz ^{1}H-NMR spectrum in $CDCl_3$ for **53**.

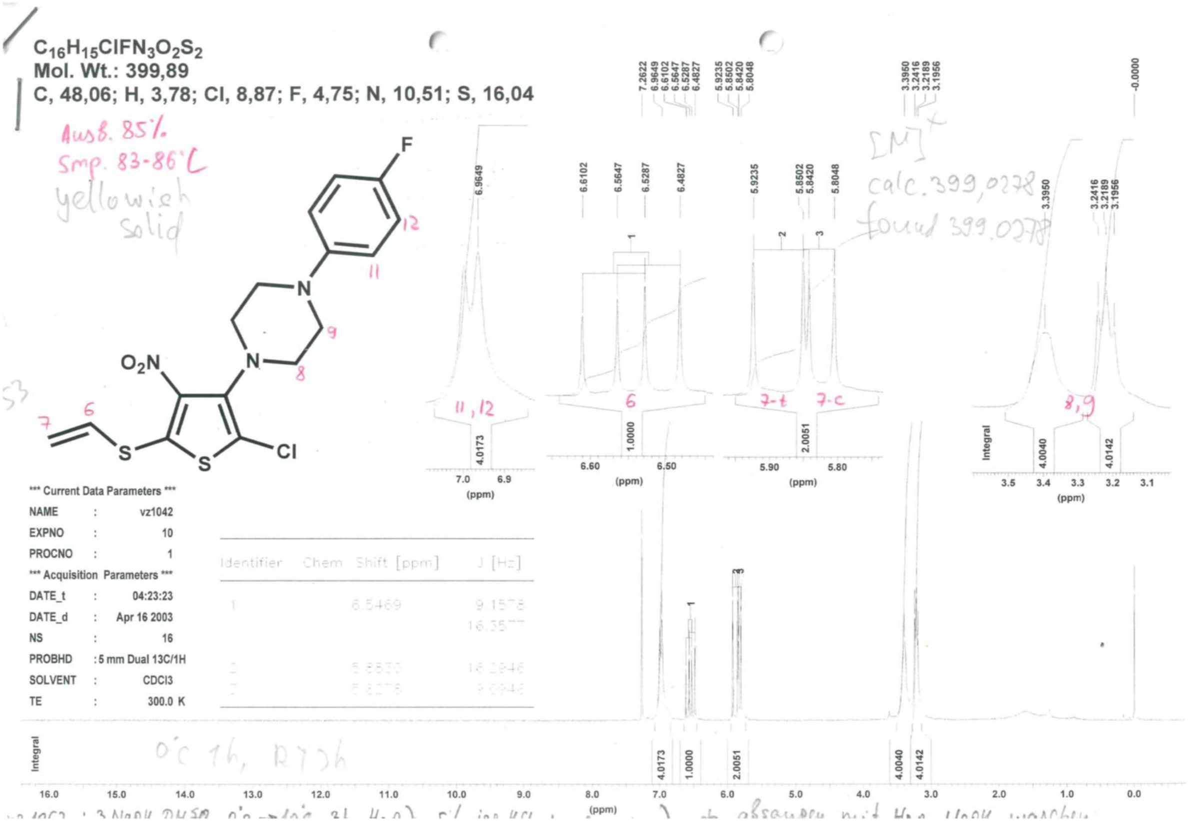

Figure S190. 50 MHz ^{13}C-NMR spectrum in $CDCl_3$ for **53**.

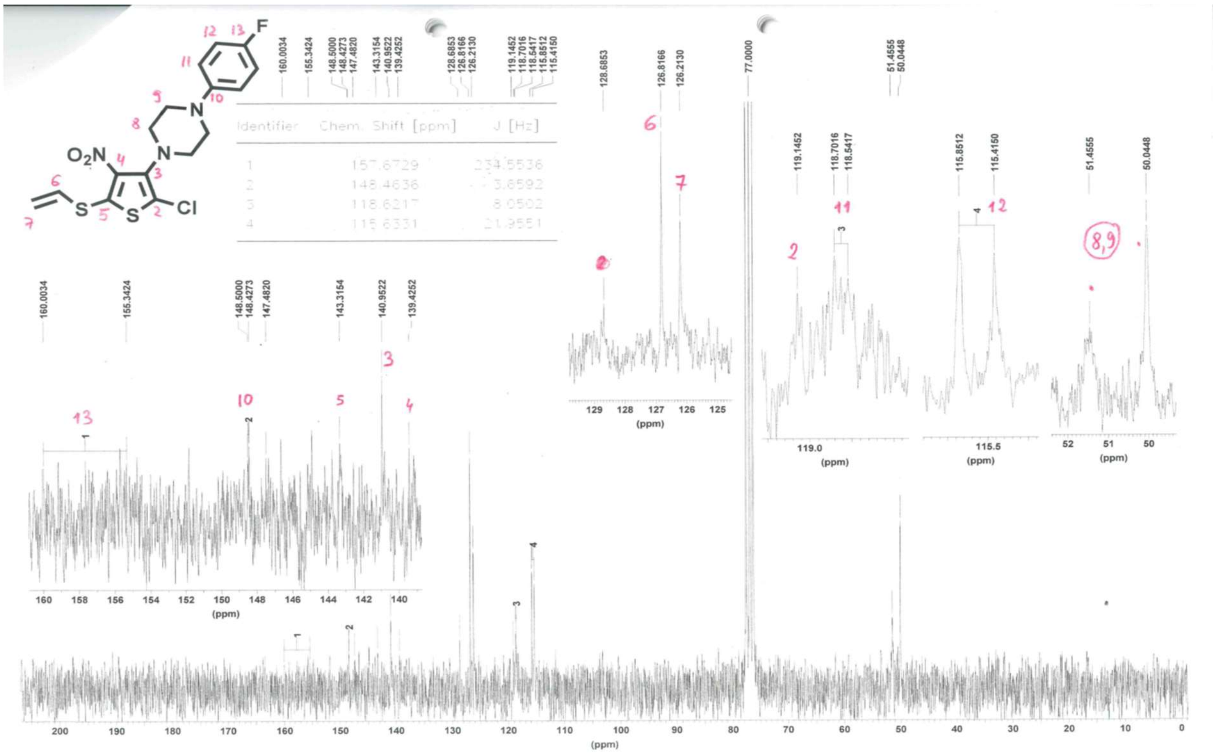

Figure S191. 400 MHz ^{1}H-NMR spectrum in $CDCl_3$ for **54**.

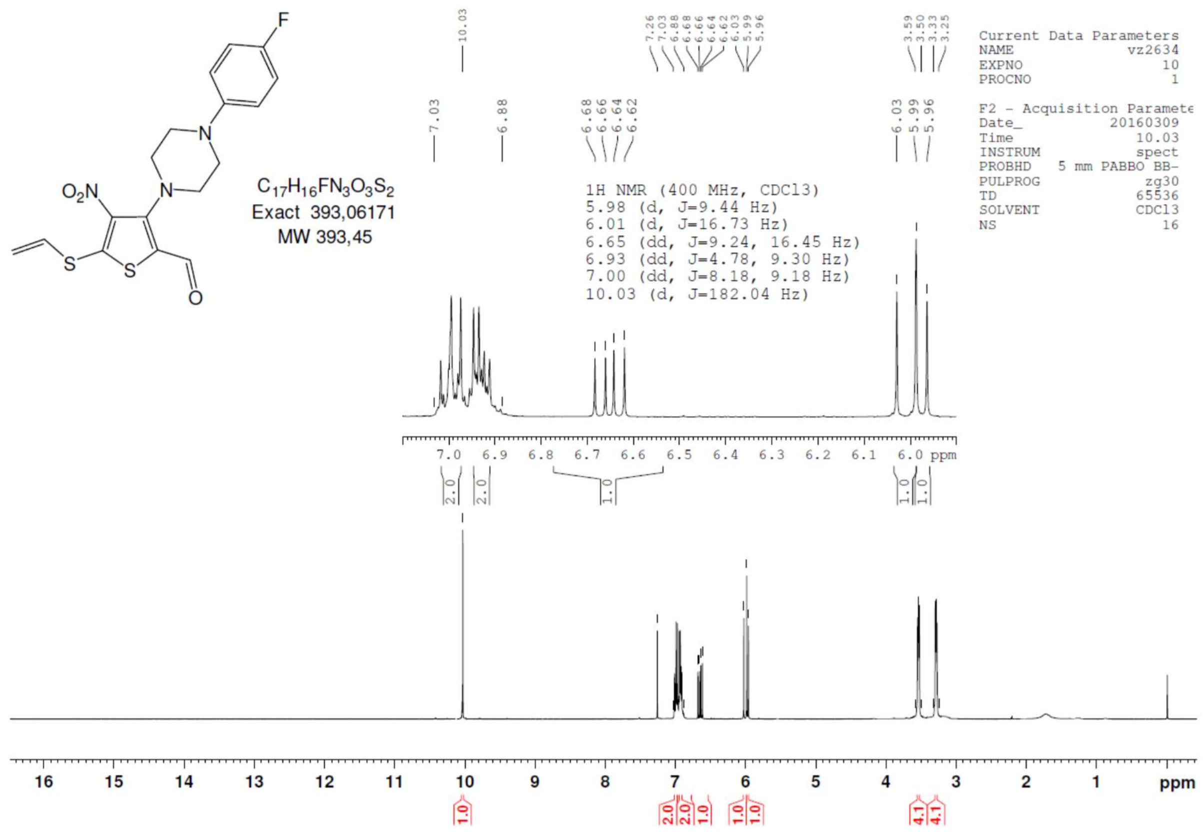

Figure S192. 100 MHz ^{13}C-NMR spectrum in $CDCl_3$ for **54**.

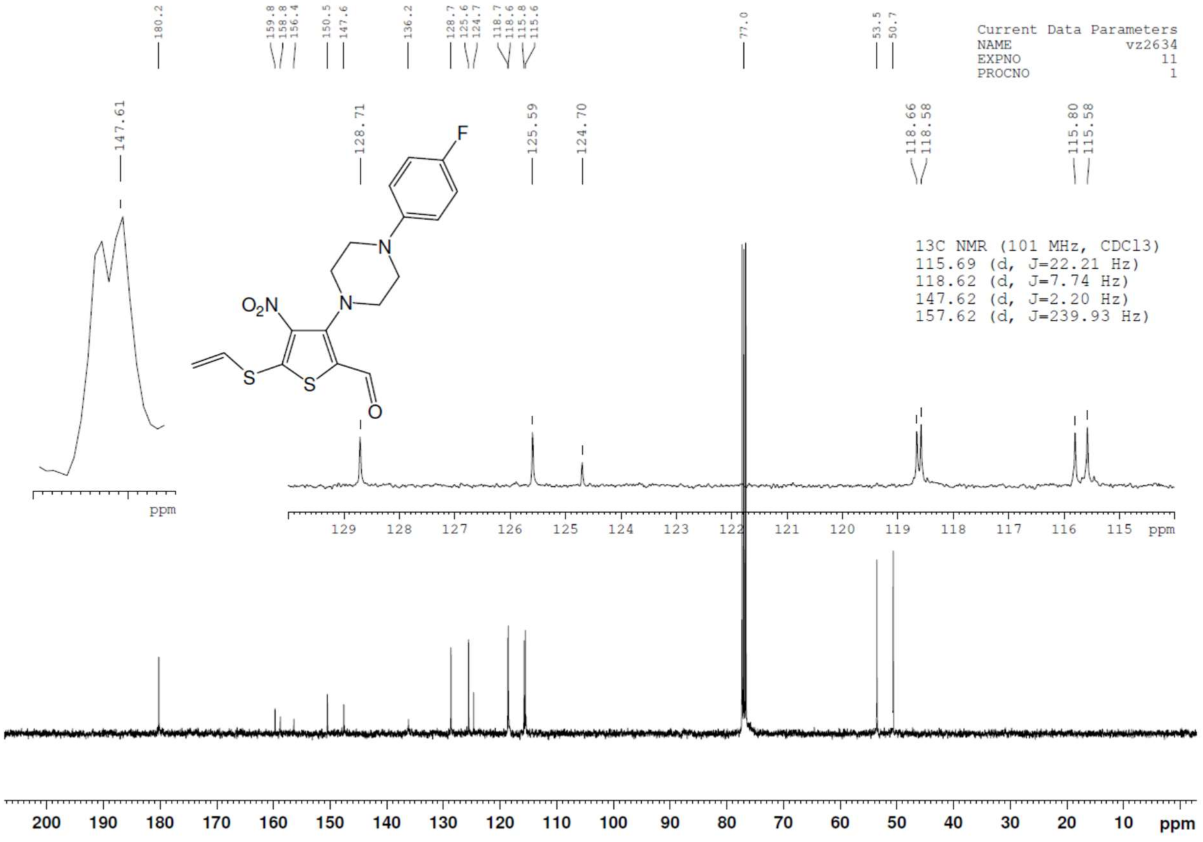

Figure S193. 200 MHz ¹H-NMR spectrum in CDCl₃ for **57**.

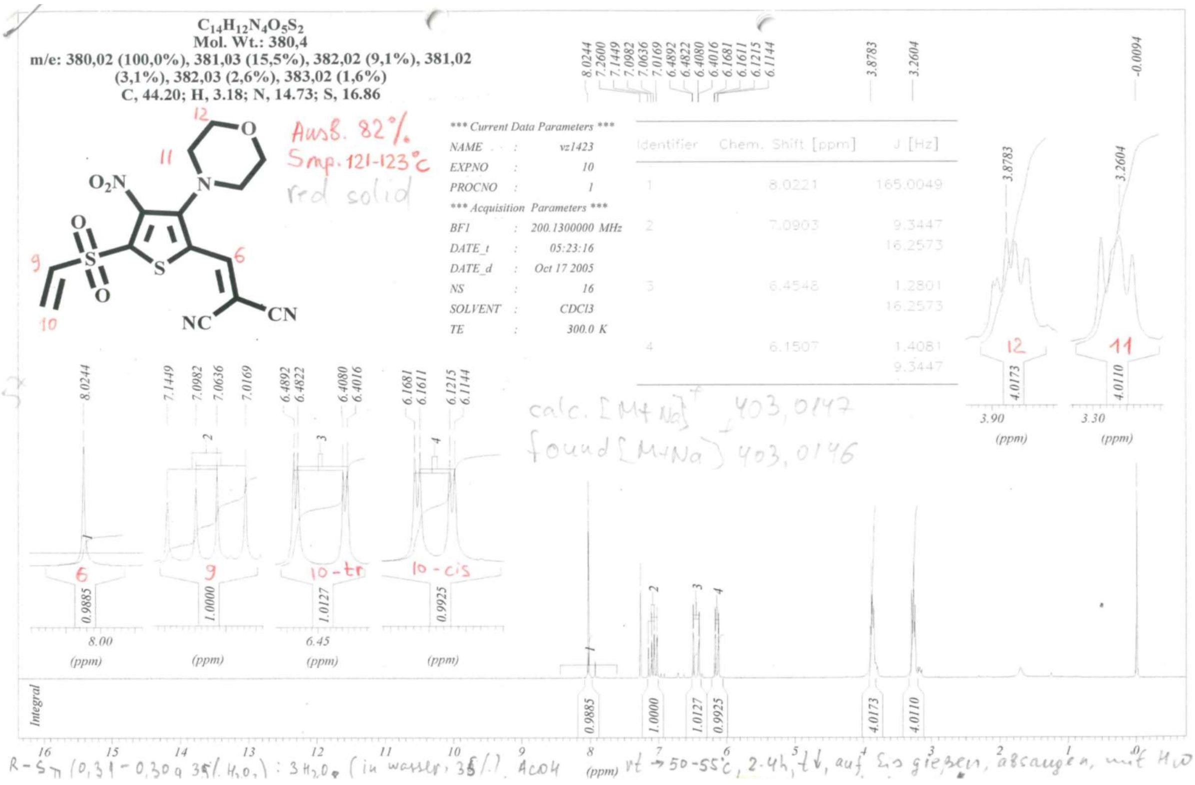

Figure S194. 50 MHz ¹³C-NMR spectrum in CDCl₃ for **57**.

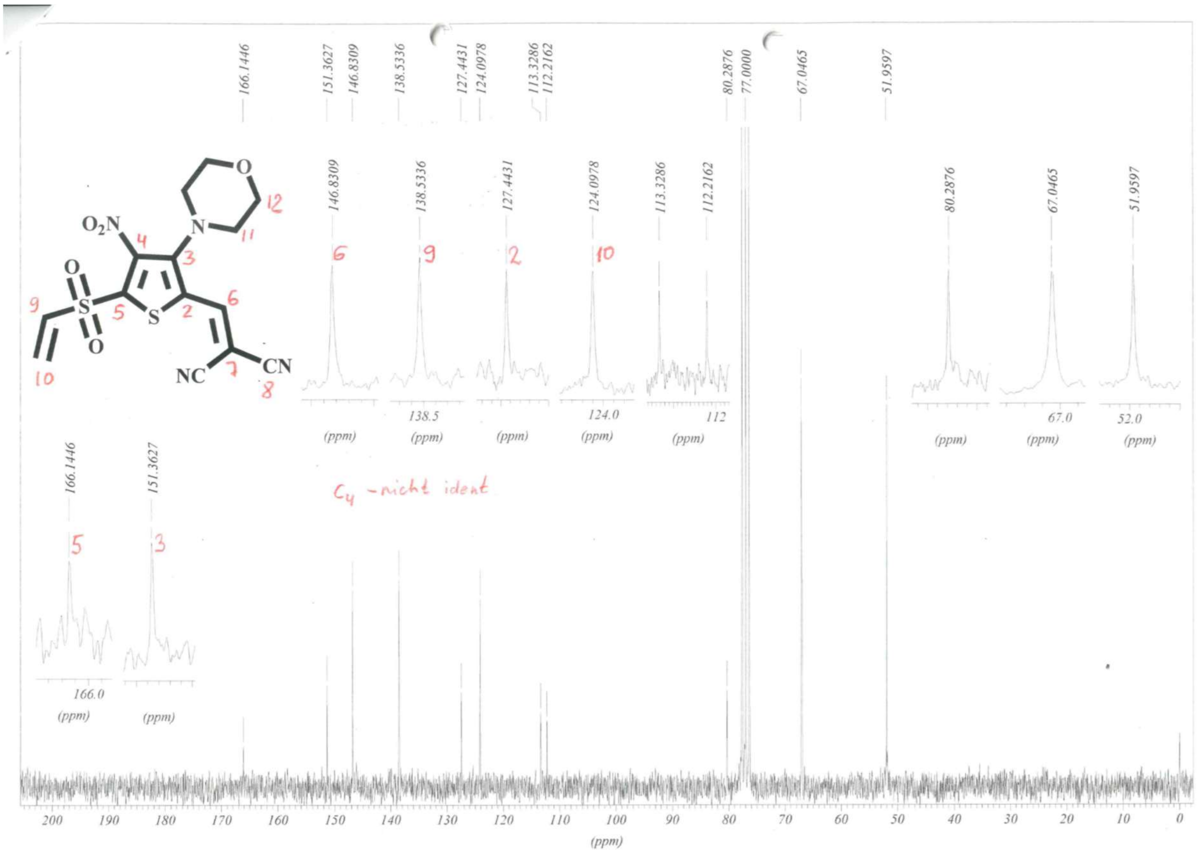

Figure S195. 200 MHz ^{1}H-NMR spectrum in $CDCl_3$ for **58**.

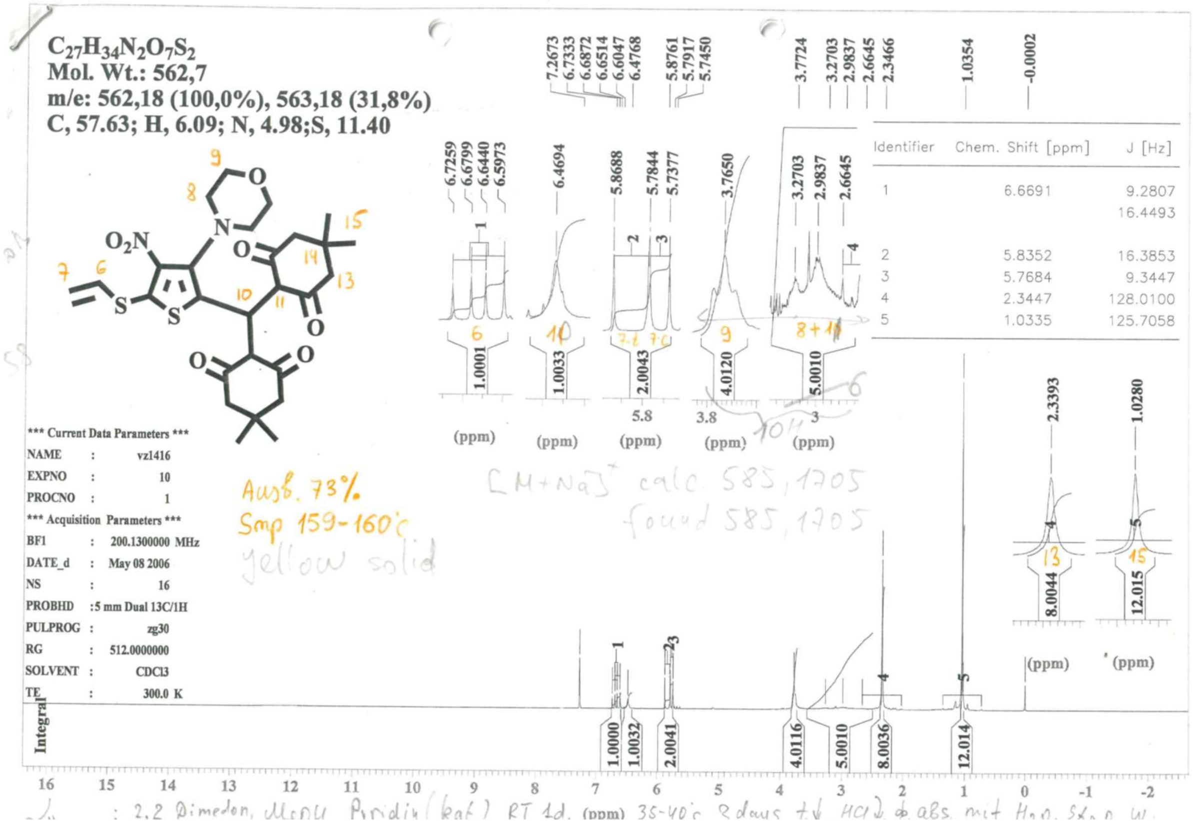

Figure S196. 50 MHz ^{13}C-NMR spectrum in $CDCl_3$ for **58**.

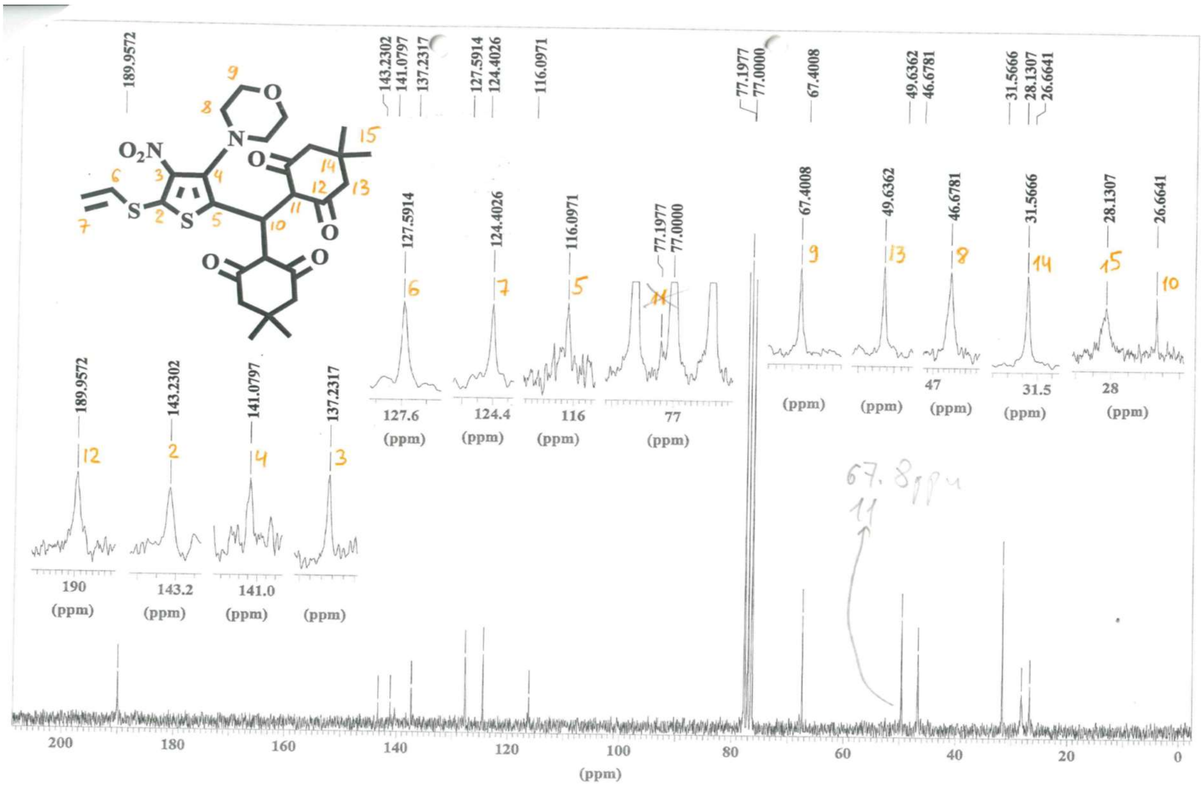

Figure S197. MS spectrum for 2,2'-{[5-(ethenylsulfanyl)-3-(morpholin-4-yl)-4-nitrothiophen-2-yl]methanediyl}bis(5,5-dimethylcyclohexane-1,3-dione (**58**).

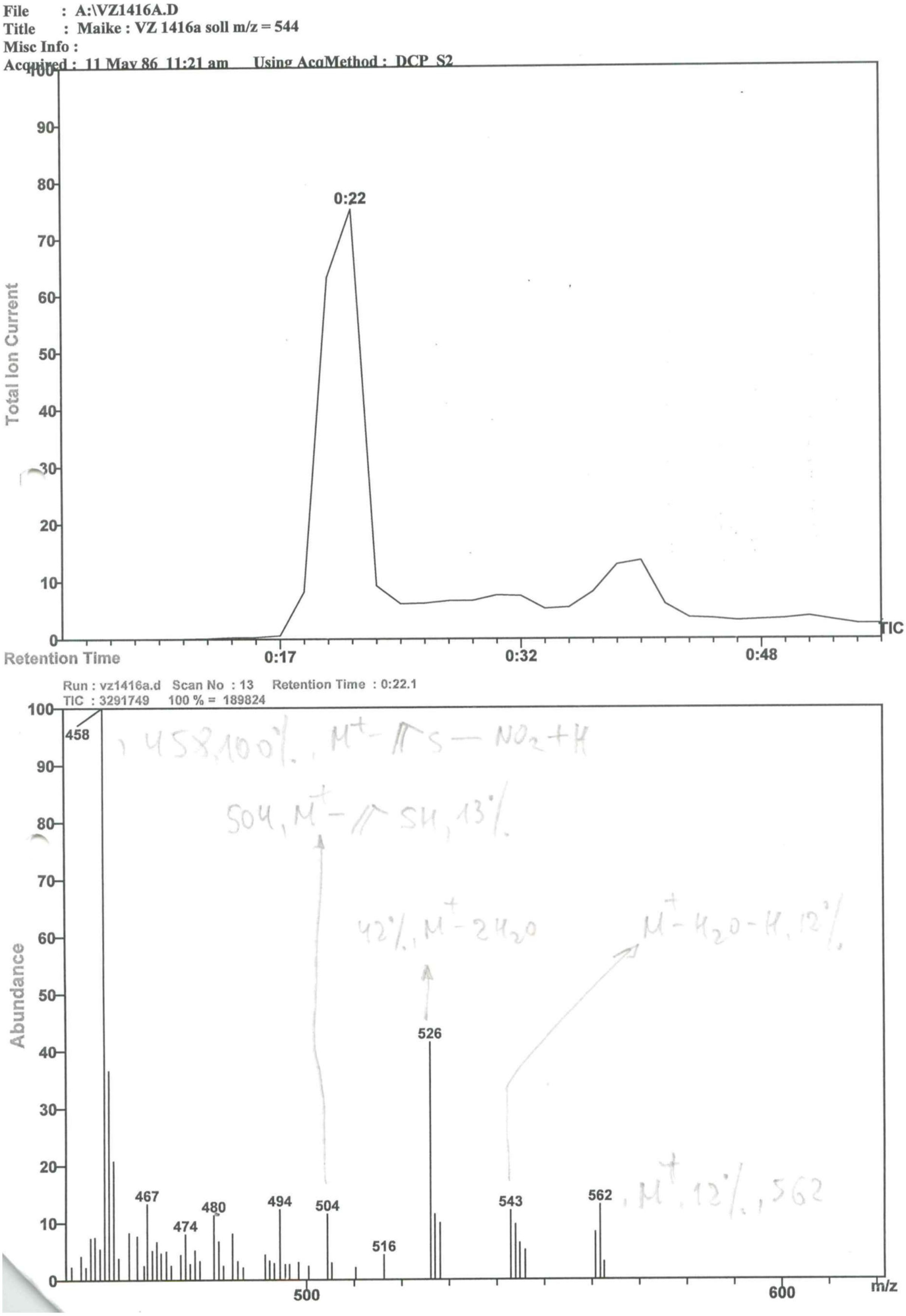

Figure S199. 200 MHz ^{1}H-NMR spectrum in $CDCl_3$ for **59**.

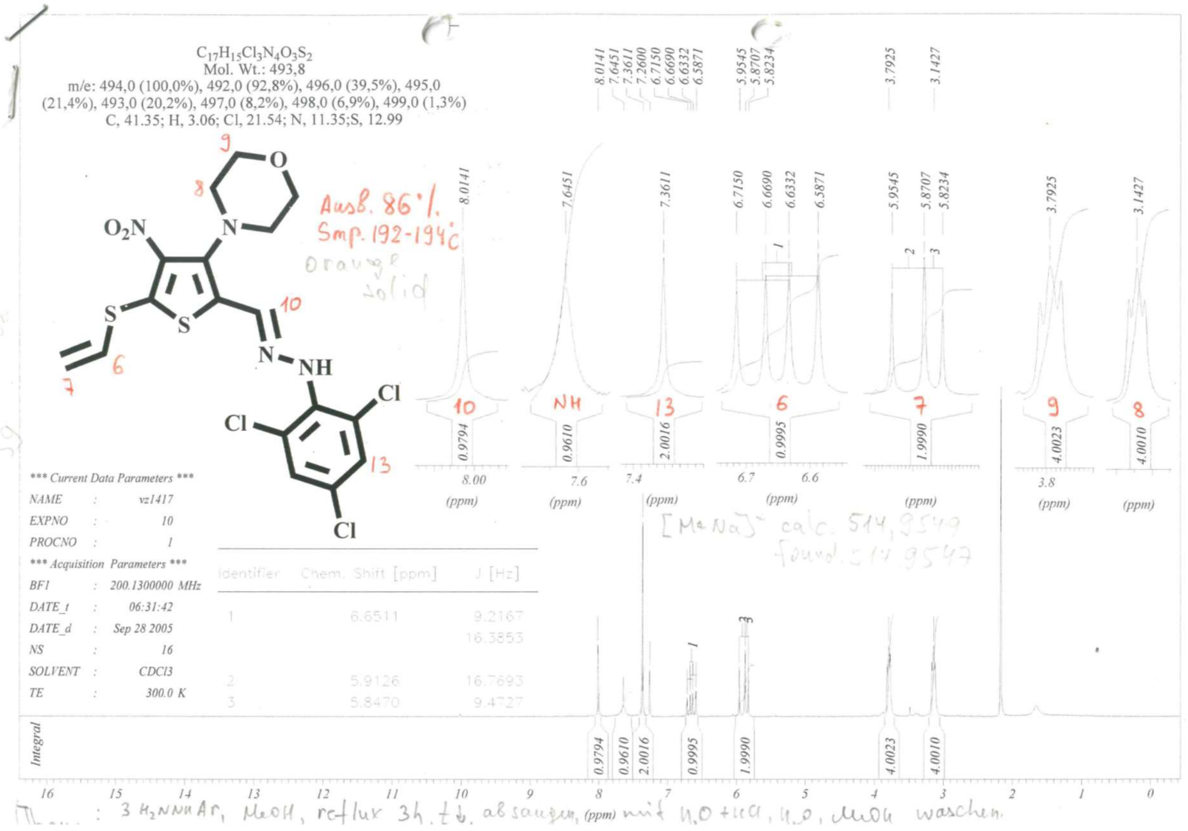

Figure S190. 50 MHz ^{13}C-NMR spectrum in $CDCl_3$ for **59**.

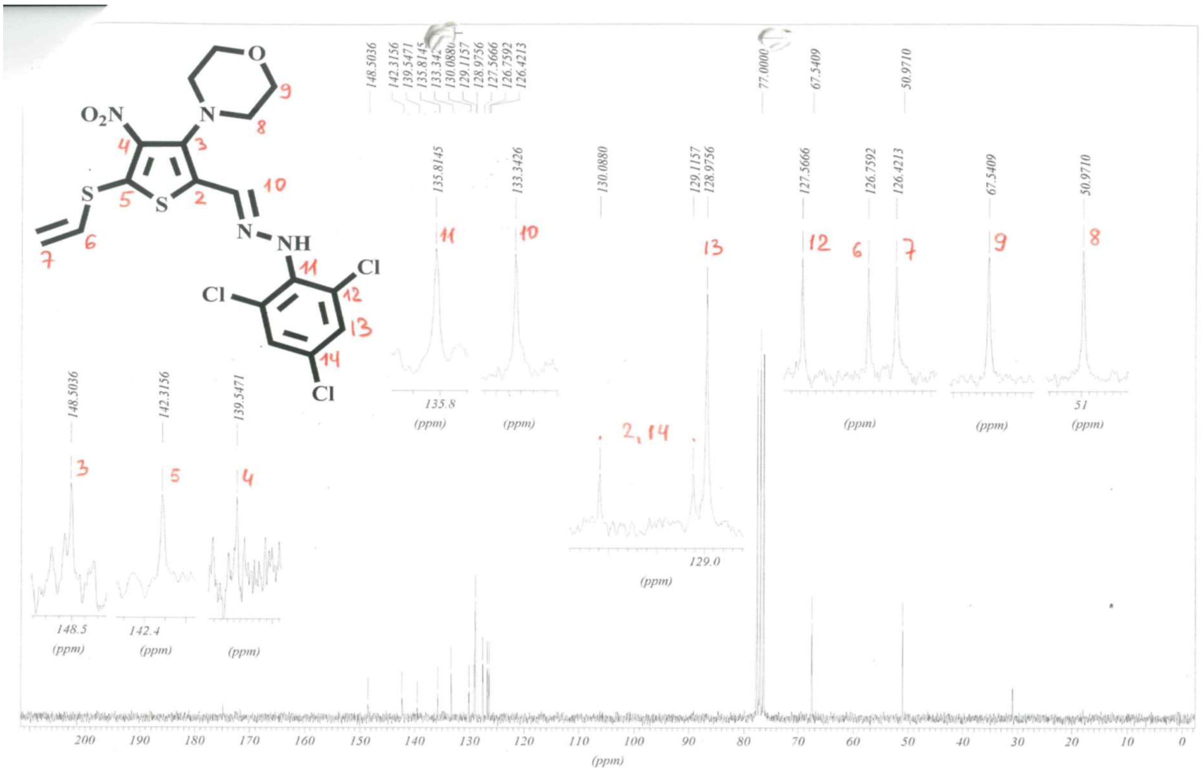

Figure S200. MS spectrum for 4-[5-(ethenylsulfanyl)-4-nitro-2-{[2-(2,4,6-trichlorophenyl)hydrazinylidene]methyl}thiophen-3-yl]morpholine (**59**).

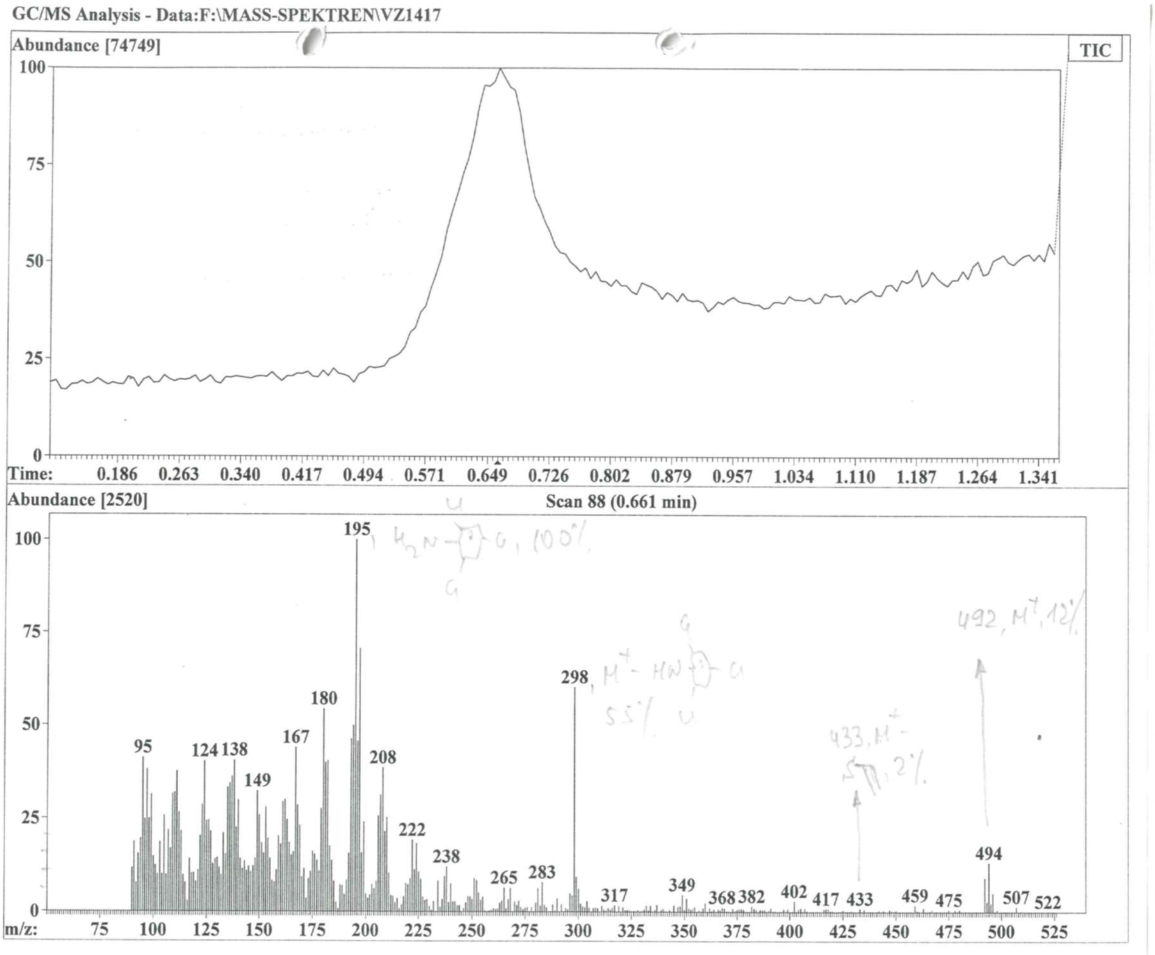

Figure S201. 400 MHz ^{1}H-NMR spectrum in $CDCl_3$ for **60**.

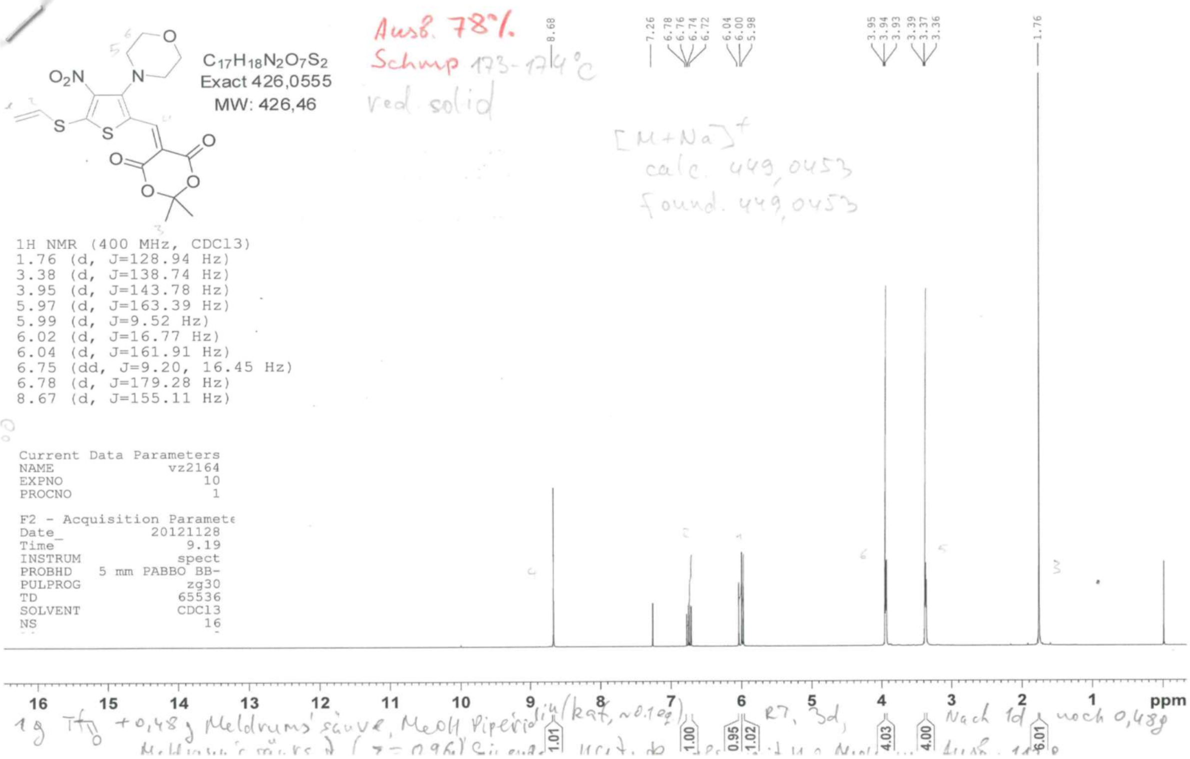

Figure S202. 100 MHz ^{13}C-NMR spectrum in $CDCl_3$ for **60**.

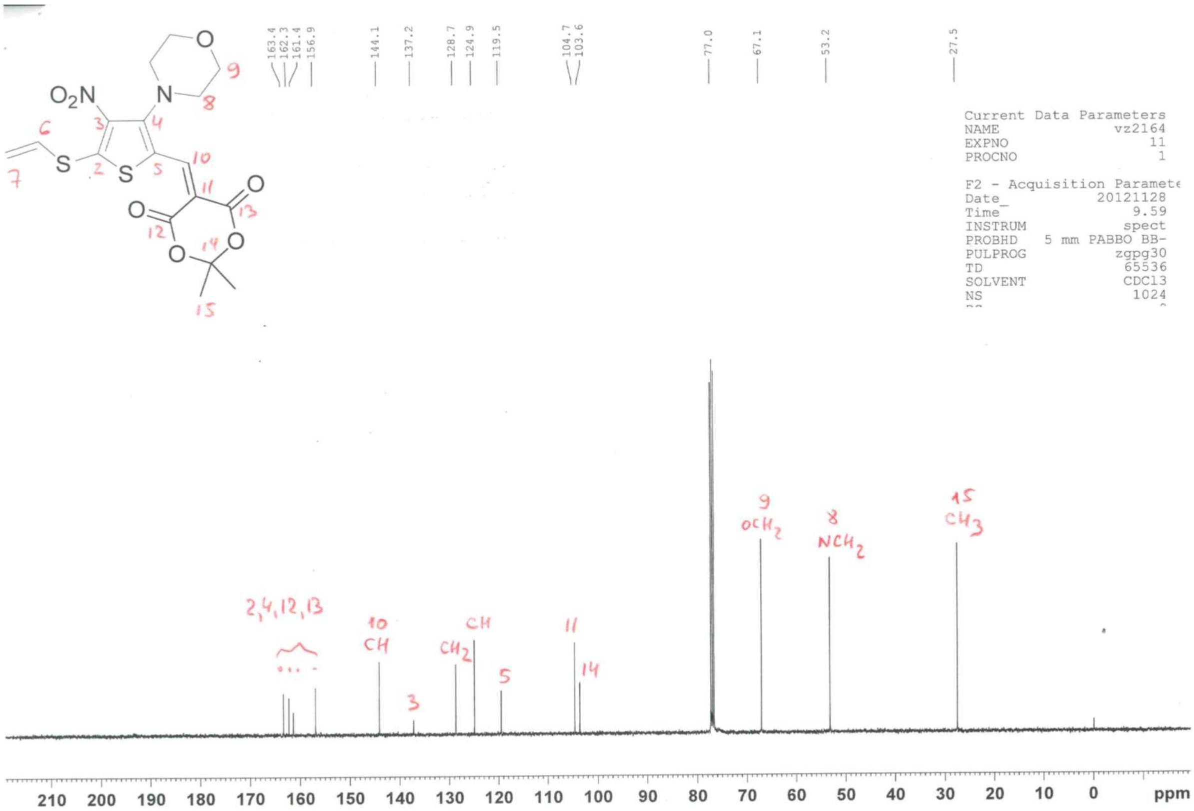

Figure S203. MS spectrum for 5-{[5-(ethenylsulfanyl)-3-(morpholin-4-yl)-4-nitrothiophen-2-yl]methylidene}-2,2-dimethyl- 1,3-dioxane-4,6-dione (**60**).

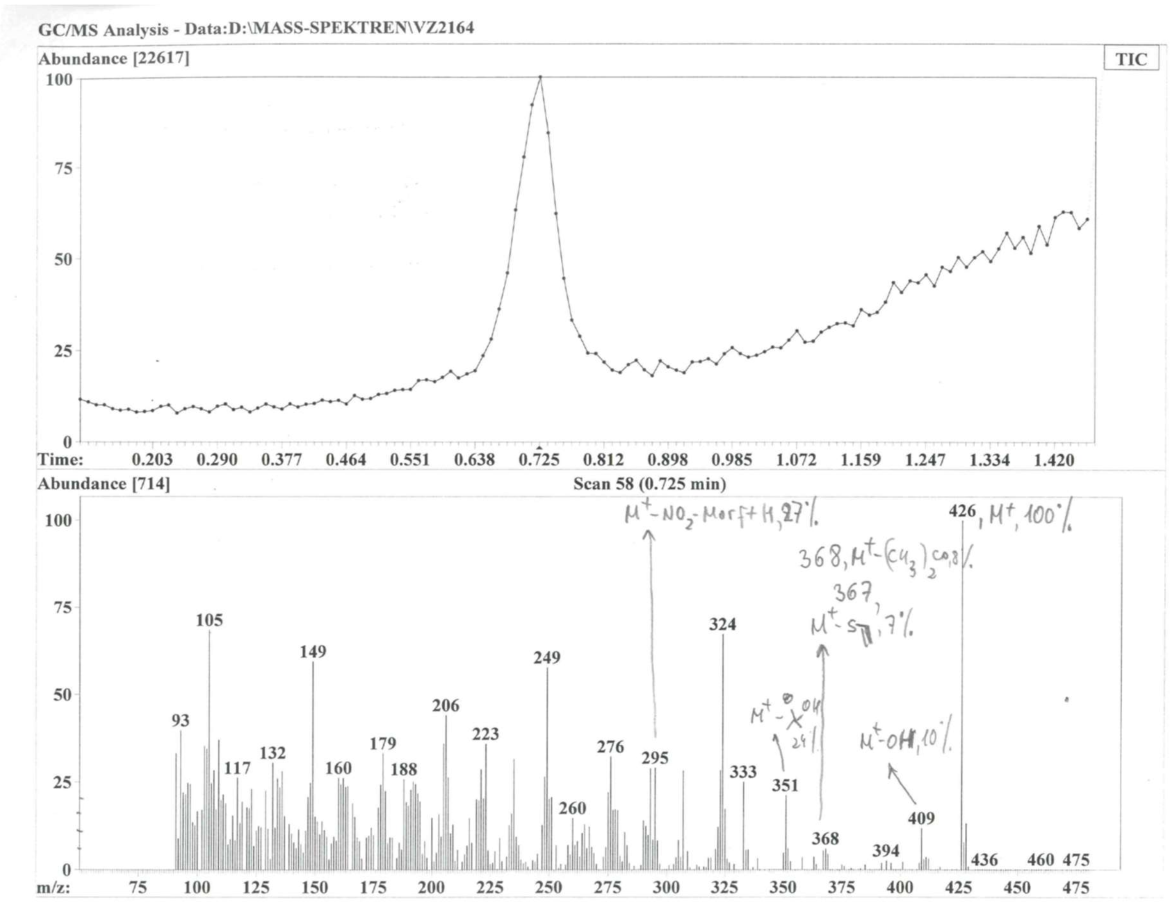

Figure S204. Biological profiling of compounds **4a-32b**.

Compd. No.	S. aureus [% growth]	UPEC796 [% growth]	L929 [viability %]	L929 IC_{50} [µM]	S. aureus IC_{50} [µM]
**4a**	140,46	101,56	85,22	nd	
**4b**	125,95	96,32	86,92	nd	
**5a**	130,28	104,67	86,00	nd	
**5b**	157,76	103,80	85,93	nd	
**5c**	134,35	104,17	84,36	nd	
**5d**	130,28	102,80	88,10	nd	
**7a**	208,40	66,04	82,71	nd	
**7b**	88,30	93,21	98,20	nd	
**7c**	114,50	100,06	94,98	nd	
**8c**	-6,87	72,90	82,52	> 50	
**19a**	105,85	96,07	84,17	nd	
**19b**	123,16	98,57	86,72	nd	
**20a**	38,17	63,80	71,03	44.4 +/- 16.5	
**20b**	-11,20	70,90	81,32	> 50	
**21a**	69,72	98,44	82,90	nd	
**21b**	110,94	90,59	84,12	nd	
**21c**	70,99	82,12	88,46	nd	
**21d**	72,52	95,45	82,11	nd	
**21e**	86,26	86,36	84,67	nd	
**21f**	90,08	89,84	81,65	nd	
**23a**	76,34	87,23	55,83	nd	
**23b**	27,48	87,60	35,39	6.2 +/- 1.8	
**26a**	112,72	93,46	82,96	nd	
**26b**	112,21	98,57	84,98	nd	
**27f**	79,39	96,95	68,26	nd	
**27c**	90,33	86,23	-0,55	1.5 +/- 0.4	
**27d**	89,31	96,82	41,55	nd	
**27e**	152,67	99,19	75,45	nd	
**28**	-17,81	75,02	-0,29	1.05 +/- 0.2	
**29a**	67,43	87,10	83,13	nd	
**29b**	141,73	94,45	92,16	nd	
**29c**	140,46	92,83	83,48	nd	
**30**	2,29	94,33	89,23	> 50	
**31a**	98,98	95,70	3,98	6.0 +/- 1.1	
**31b**	77,61	81,00	1,62	5.7 +/-1.2	
**32a**	132,82	99,07	95,05	nd	
**32b**	115,01	87,73	90,10	nd	

Figure S205. Biological profiling of compounds **33a-60**.

Compd. No.	S. aureus [% growth]	UPEC796 [% growth]	L929 [viability %]	L929 IC_{50} [µM]	S. aureus IC_{50} [µM]
**33a**	106,87	98,57	87,44	nd	
**33b**	106,11	104,67	92,48	nd	
**35a**	1,02	102,93	75,36	56.2 +/- 8.7	37,2
**35b**	-6,87	90,59	53,31	13.9 +/- 2.6	8,6
**36a**	160,05	105,67	91,74	nd	
**36b**	119,59	98,82	78,94	nd	
**38**	167,43	96,07	80,91	nd	
**41**	122,65	103,93	89,85	nd	
**43**	135,62	102,80	88,95	nd	
**44a**	112,98	86,11	78,73	nd	
**44b**	101,78	98,07	84,00	nd	
**44c**	173,79	120,37	86,30	nd	
**44d**	98,73	89,10	85,17	nd	
**44e**	126,97	102,43	87,64	nd	
**45**	151,15	104,17	76,49	nd	
**46a**	102,29	84,36	81,64	nd	
**46b**	91,86	89,72	78,40	nd	
**46c**	110,69	92,21	88,45	nd	
**46d**	122,90	96,82	83,96	nd	
**46e**	115,78	86,36	89,24	nd	
**48**	123,41	108,29	91,06	nd	
**50**	110,43	94,45	90,76	nd	
**52**	167,94	91,34	96,93	nd	
**53**	163,87	92,96	102,82	nd	
**58**	9,16	94,21	-0,42	3.1 +/- 0.4	34.6 +/- 5.0
**59**	133,84	90,72	77,92	nd	
**60**	15,52	88,47	79,45	20.1+/-3.9	

All compounds were used in single concentrations, which was 100 µM for the application to the bacterial strains *Staphylococcus aureus* SH-1000 and uropathogenic *Escherichis coli* UPEC 796, and 10 µM for the evaluation of cytotoxicity with the murine fibroblast cell line L929. The percentages of growth and of viability respectively are given, with the values obtained with the untreated organisms being 100%. The compounds were selected for the IC_{50} – determinations on the basis of these primary data: 50% residual growth or viability was chosen as threshold.
